# Resequencing of the common marmoset genome improves genome assemblies and gene-coding sequence analysis

**DOI:** 10.1038/srep16894

**Published:** 2015-11-20

**Authors:** Kengo Sato, Yoko Kuroki, Wakako Kumita, Asao Fujiyama, Atsushi Toyoda, Jun Kawai, Atsushi Iriki, Erika Sasaki, Hideyuki Okano, Yasubumi Sakakibara

**Affiliations:** 1 Department of Biosciences and Informatics, Keio University, Japan; 2 Department of Genome Medicine, National Research Institute for Child Health and Development, NCCHD, Japan; 3 Department of Applied Developmental Biology, Central Institute for Experimental Animals, Japan; 4 Principles of Informatics Research Division, National Institute of Informatics, Japan; 5 Center for Information Biology, National Institute of Genetics, Japan; 6 Preventive Medicine and Diagnosis Innovation Program, RIEKN, Japan; 7 Laboratory for Symbolic Cognitive Development, Brain Science Institute RIKEN, Japan; 8 Department of Physiology, Keio University School of Medicine, Japan; 9 Laboratory for Marmoset Neural Architecture, RIKEN Brain Science Institute; 10 Keio Advanced Research Center, Japan

## Abstract

The first draft of the common marmoset (*Callithrix jacchus*) genome was published by the Marmoset Genome Sequencing and Analysis Consortium. The draft was based on whole-genome shotgun sequencing, and the current assembly version is Callithrix_jacches-3.2.1, but there still exist 187,214 undetermined gap regions and supercontigs and relatively short contigs that are unmapped to chromosomes in the draft genome. We performed resequencing and assembly of the genome of common marmoset by deep sequencing with high-throughput sequencing technology. Several different sequence runs using Illumina sequencing platforms were executed, and 181 Gbp of high-quality bases including mate-pairs with long insert lengths of 3, 8, 20, and 40 Kbp were obtained, that is, approximately 60× coverage. The resequencing significantly improved the MGSAC draft genome sequence. The N50 of the contigs, which is a statistical measure used to evaluate assembly quality, doubled. As a result, 51% of the contigs (total length: 299 Mbp) that were unmapped to chromosomes in the MGSAC draft were merged with chromosomal contigs, and the improved genome sequence helped to detect 5,288 new genes that are homologous to human cDNAs and the gaps in 5,187 transcripts of the Ensembl gene annotations were completely filled.

The common marmoset (*Callithrix jacchus*) is a New World monkey native to northeast Brazil that has received much attention as an alternative to traditionally used non-human primate species. The marmoset has numerous advantages as a model animal in biomedical research, including regenerative medicine and drug development, because of its unique biological characteristics, such as its similar physiology to humans, its small body size, and the availability of transgenic technologies^1^,^2^,^3^. Furthermore, its well-developed frontal cortex and compact brain size are suitable for studies of the brain^3^,^4^,^5^,^6^.

The recent development of genome editing technologies enables the production of target gene knock-out animals without the use of pluripotent stem cells that can contribute to germ cells^7^,^8^,^9^,^10^. These genome editing technologies are also expected to be applied to the marmoset for the generation of target gene knock-out models to understand genes with unknown functions that specifically exist in primates and to create human disease models^11^. However, to utilize these technologies in the marmoset, a well-defined and more complete genome sequence is required.

The Marmoset Genome Sequencing and Analysis Consortium (MGSAC)^12^ reported the whole-genome sequence of the common marmoset (Callithrix jacchus). The 2.26-Gb genome of a female marmoset was assembled using Sanger read data (6×) using a Sanger sequencer (ABI3730 instrument) and a whole-genome shotgun strategy. However, there still exist 187,214 undetermined gap regions and supercontigs and relatively short contigs that are unmapped to chromosomes in the MGSAC draft genome.

In the present study, we performed resequencing and assembly of the genome of common marmoset being bred at the Central Institute for Experimental Animals (CIEA) in Japan. The marmoset genome was sequenced by deep sequencing with high-throughput sequencing technology using Illumina GAIIx and HiSeq 2000 sequencer. 181 Gbp of high-quality bases including mate-pairs with long insert lengths of 3, 8, 20, and 40 Kbp were obtained, and the coverage depth was approximately 60×. The resequencing significantly improved the MGSAC draft genome sequence and gene-coding annotations. The BAC clone library consisting of 76,410 BAC clones was also constructed and the BAC end sequences were determined and mapped to the improved genome sequence.

## Results

### Genome reassembly using Illumina mate-pair and paired-end sequence reads

A summary of the genomic DNA sequences of the CIEA common marmoset (Animal I2075 male) generated using an Illumina sequencing platform is shown in Table 1.

A total of 1,336 million (M) resequenced single-end (SE) and paired-end (PE) reads with two different insert sizes of 500 and 700 bp was generated using an Illumina GAIIx sequencer, and after quality filtering, 1,276 M reads were obtained for assembly, corresponding to 141 Gbp, and the coverage depth was 47×. In addition, 500 M mate-pair (MP) reads with long inserts of 3, 8, 20, and 40 Kbp were generated using an Illumina HiSeq 2000 sequencer.

To recover the missing regions from the MGSAC draft genome, we mapped the resequenced reads to the draft genome using BWA-MEM^13^, and then collected 143 M unmapped reads. These were assembled into 4,459 contigs using SOAPdenovo2^14^. The total length of the new contigs is 7.3 Mbp and the N50 (a statistical measure used to evaluate assembly quality), is 1,512 bp. These new assembled contigs are used for improving the draft genome sequence.

We utilized the long insert MP reads to improve the genome according to the strategy proposed by van Heesch *et al.*^15^. New scaffolds were generated from the MGSAC contigs and the resequenced new contigs by SSPACE 3.0^16^ with the MP reads. The gaps remaining from the scaffolding were filled using GapCloser v1.12-r6^14^ with the Illumina short reads.

To reconstruct chromosomes, we mapped the contigs generated from the new scaffolds to the MGSAC draft genome using LAST^17^, and replaced the original genome sequences with the mapped contigs. Then, we called single nucleotide polymorphisms (SNPs) using the CIEA short reads to generate the CIEA-based genome. Finally, we obtained an improved genome sequence containing 104,312 contigs with an N50 of 61,143 bp. This improved assembly result contrasts with the previous MGSAC assembly where the number of contigs in the *C. jacchus* (caljac)-3.2 draft genome is 201,371 with an N50 of 29,273 bp. This implied that 97,059 contigs in caljac-3.2 were merged so that the N50 of the contigs doubled. As a result, among the 187,214 gap regions comprising 162,452,744 bp in the draft genome, 65,384 gap regions covering 32,773,613 bp were filled by the improved contigs. The statistics of assembly results is summarized in Table 2 and Supplemental Table 5. Thus, a significant improvement of the draft genome sequence was achieved.

**Figure 1:**
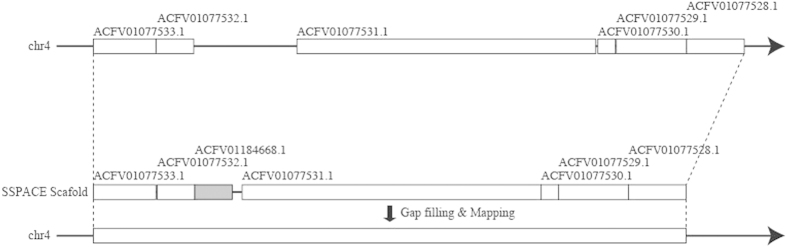
An example of an improved region. (**a**) The region “chr4: 69196273–69307838” in the MGSAC draft genome consisted of 6 contigs, which includes 5 gaps, 1 with a length > 10 Kbp. (**b**) SSPACE generated a scaffold using MP reads, in which the region corresponding to the large gap was filled with the contig ACFV01184668.1 (gray), which was part of the non-chromosomal scaffolds in the MGSAC draft genome. (**c**) The remaining gaps were filled by GapCloser using Illumina short sequence reads. Finally, the genome was updated by mapping the gap-filled scaffolds.

Further, by scaffolding using the MP reads, 51% of the contigs (total length: 299 Mbp) that were unmapped to chromosomes in the MGSAC draft were merged to chromosomal contigs. Figure 1 shows an example of an improved region where an unmapped contig was used to fill a large gap. In Supplemental Table 1, the number and location of the 51% of MGSAC contigs that have been mapped to each chromosome in the improved genome are shown.

### BAC-end sequences and physical map construction

A bacterial artificial chromosome (BAC) library called CJB1 consisting of 76,410 BAC clones was constructed from a CIEA female marmoset (Animal I992 female that is different from the CIEA marmoset (Animal I2075 male) for the whole genome resequence), and BAC end sequences were generated by Sanger sequencing. The BAC-end sequences were mapped to the improved genome using BLAT^18^ with the default parameters. We filtered out improperly mapped pairs of BAC-ends that do not satisfy the following conditions: both ends are mapped on the same chromosome; the mapped direction of both ends is either forward-reverse or reverse-forward; and the insert size is within 350 kb. The result was that 61,654 ends among 76,410 was mapped properly (mapping rate: 80.28%). The distribution of insert sizes is shown in Supplemental Figure 1.

### Revised gene content in the improved regions

A total of 52,754 out of 55,116 transcripts in the Ensembl gene annotations were successfully updated by converting the genomic coordinates from the MGSAC draft genome into the improved genome. Among the 28,471 updated transcripts that contained gaps in the draft genome, 5,187 transcripts were completely filled. These completely filled transcripts are listed in Supplemental Table 2, in which the first, second, and third columns represent Ensembl transcript_id, gene_id, and gene_name, respectively.

We aligned marmoset and human cDNAs downloaded from Ensembl 78^19^ on the improved genome sequence using BLAT, resulting in the prediction of 45,436 and 116,826 transcripts, respectively. Supplemental Figure 2 shows the mapping rate of length for marmoset and human cDNAs to the improved genome. Among the 13,200 alignments of human cDNAs that did not overlap with marmoset cDNAs, 5,288 alignments were located in the gap regions of the MGSAC draft genome that were filled in the improved genome, meaning that the improved genome sequence helped to detect new genes that are homologous to human cDNAs. These 13,200 newly found transcripts are listed in Supplemental Table 3, in which the first, second, and third columns represent Ensembl transcript_id, gene_id, and gene_name, respectively. Further, the 5,228 genes located in the filled gaps in the improved genome sequence are annotated “filled” in Supplemental Table 3.

We predicted 78,227 transcripts using Cufflinks^20^ from RNA-seq data of 5 organs produced by the non-human primate reference transcriptome resource (NHPRTR)^21^ after mapping them using STAR^22^.

We performed *ab initio* gene prediction using AUGUSTUS^23^, which predicted a total of 32,464 genes. Among the 18,706 predicted genes that did not overlap with the mapped marmoset cDNAs, 12,209 were located in the gap regions that were filled in the improved genome. Among 19,199 predicted genes that were not overlapped with the mapped human cDNAs, 11,739 predicted genes contain the gap regions that have been filled in the improved genome. The statistics of gene annotations in the improved genome sequence is summarized in Table 3 and Supplemental Figure 3. To compare gene annotations between both genomes, we also show the summary of gene annotations in the original genome in Supplemental Table 4.

### Variation analysis: genetic distance based on SNPs

**Figure 2:**
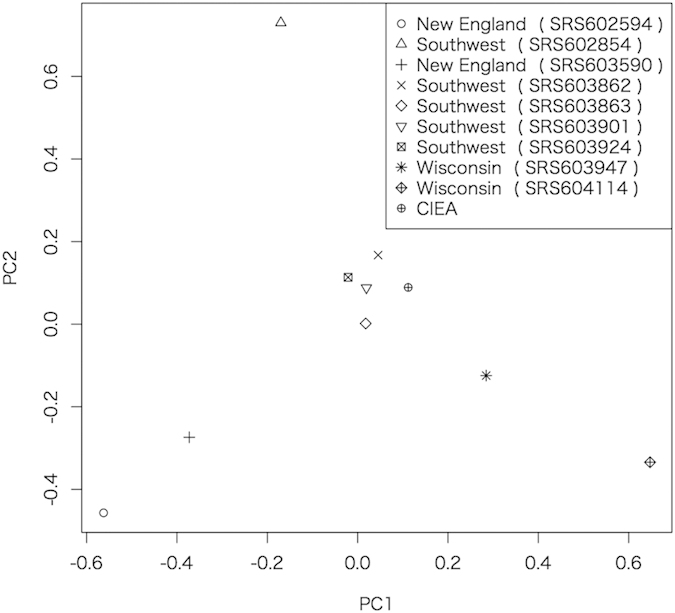
Principal component analysis based on the pairwise allele-sharing distance among the CIEA marmoset and 9 MGSAC marmosets. The contribution rate of PC1 and PC2 is 13.95% and 13.44%, respectively.

From autosomal SNPs, genetic distance and genome sequence diversity were investigated among the CIEA marmoset (Animal I2075 male) and 9 marmosets analyzed by MGSAC. We performed principal component analysis (PCA) based on the pairwise allele-sharing genetic distances in the same manner as the MGSAC analysis (Fig. 2). The results surprisingly showed that according to the pairwise allele-sharing genetic distances the CIEA marmoset was genetically close to the Southwest NPRC colony, from which the sample for the MGSAC draft genome originated, suggesting our reassembly approach combining the CIEA and MGSAC contigs is reasonable.

## Discussion

The MP reads with long inserts exhibited significant effects to complete rather long gaps and merge unmapped contigs. Table 4 shows the total number of new (CIEA) contigs and original (MGSAC) contigs mapped to each chromosome in the improved genome, and the number of original (MGSAC) contigs newly mapped to each chromosome in the improved genome but remained unmapped in the MGSAC draft genome. Significant improvements specifically at the sex chromosomes “X” and “Y” were achieved by the resequencing and the improved genome assembly. Especially, the “Y” chromosome was well reconstructed while the previous assembly of the “Y” chromosome was very poor.

Supplemental table 5 shows the total length of each chromosome in MGSAC genome and the improved (CIEA) genome. As shown in the table, the length of every chromosome in the CIEA genome becomes shorter than the one in MGSAC. We consider that this is mainly because the insert lengths of scaffolds in MGSCA genome were overestimated and the lengths were modified to the correct lengths by gap filling in the CIEA genome.

Figure 1 shows an example of completing long gap region. The region “chr4: 69196273–69307838” in the MGSAC draft genome consisted of 6 contigs, which includes 5 gaps, 1 with a length > 10 Kbp. SSPACE generated a scaffold using MP reads, in which the region corresponding to the large gap was filled with the contig ACFV01184668.1 (gray), which was part of the non-chromosomal scaffolds in the MGSAC draft genome. The remaining gaps were filled by GapCloser using Illumina short sequence reads.

5,187 transcripts containing gaps in the Ensembl gene annotations for the common marmoset were completely filled in the improved genome. Many important genes such as FOX family, E2F family of transcription factors, kinesin family are included in the completed transcripts. For example, GDF9 was reported as a strong candidate for influencing diminutive body size and had a functional nonsynonymous substitution in the common marmoset^12^. SOX9 is a transcription factor related to male sexual development encoded in the “Y” chromosome in SOX family.

**Figure 3:**
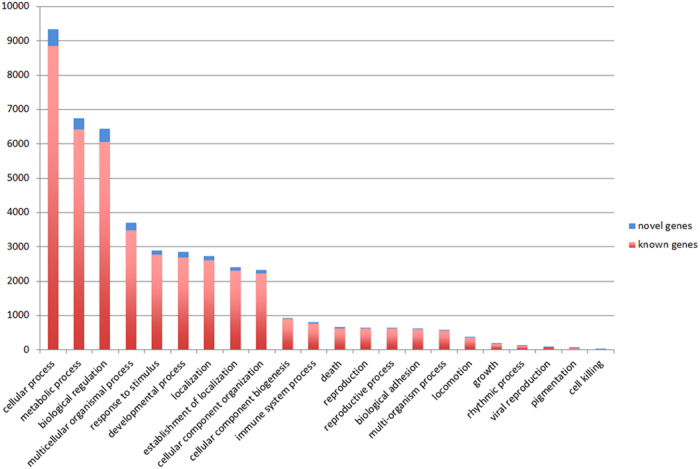
Gene Ontology (GO) category analysis for the transcripts of human cDNA mapped to the MGSAC draft genome and the transcripts of human cDNA newly mapped to the improved genome.

For the transcripts of human cDNA mapped to the MGSAC draft genome and the transcripts of human cDNA newly mapped to the improved genome, the Gene Ontology (GO) categories were analyzed. As shown in Fig. 3, there are no significant differences and no biases between the GO category distributions for the known genes mapped to the MGSAC draft genome and the novel genes newly mapped to the improved genome. This result implied that our resequencing improved the draft genome uniformly and comprehensively.

## Materials and Methods

### Marmoset sample, genomic DNA preparation, and sequencing

The marmoset colony was housed in stainless steel cages (409 × 610 × 1,578 mm) in pairs or family groups at 25–26 °C with a relative humidity of 45–55% and a 12/12 h light/dark cycle. For environmental enrichment, a wood perch was placed for locomotion and gouging, and a platform for a bed was installed in each cage. The marmosets were fed, healthy, and well-nourished and received balanced diet pellets (CMS-1M; CLEA Japan, Inc., Kawasaki, Japan), including L (+)-ascorbic acid (Nacalai Tesque, Tokyo, Japan), vitamins A, D3, and E (Duphasol AE3D; Kyoritsu Seiyaku Co., Ltd., Tokyo, Japan), and honey (Nihonhatimitsu Co., Ltd., Gifu, Japan). In addition, chicken boiled liver (DBF Pet Co., Ltd., Niigata, Japan) was given as a supporting meal once a week. The animals were supplied with tap water *ad libitum* from feed valves.

All animal experiments were approved by the Institutional Animal Care and Use Committee (CIEA ref. nos 12025 and 13071). The study was conducted in accordance with the guidelines of CIEA that comply with the Guidelines for Proper Conduct of Animal Experiments published by the Science Council of Japan. Animal care was conducted in accordance with the Guide for the Care and Use of Laboratory Animals (Institute for Laboratory Animal Resources, 2011).

The marmoset genome was extracted from 8-year, 4-month-old male marmoset (Animal I2075 male) liver by the phenol-chloroform-isoamyl alcohol extraction method. Liver sample collection was performed after euthanasia by exsanguination under ketamine (60 mg/kg) and isoflurane deep anesthesia. The genomic DNA was extracted from liver using Wizard Genomic DNA Purification Kit (Promega, USA) according to manufacturer’s instruction. We performed complete blood-letting at dissection to avoid blood contamination.

The marmoset genome was sequenced with a whole-genome shotgun strategy. DNA library preparation and sequencing were performed according to the manufacturers’ instructions. Briefly, a short insert paired-end DNA library was prepared from 1 μg of genomic DNA following fragmentation to an average size of approximately 500 and 700 bp with an M220 ultrasonicator (Covaris, Woburn, MA) and was sequenced with an Illumina Genome Analyzer IIx (Illumina Inc.). Library generation and sequencing were performed at the Genome Network Analysis Support Facility, RIKEN CLST (Yokohama, Japan).

The genomic DNA for large insert sequencing libraries was extracted from the same animal described above. The genomic DNA was extracted from kidney (Animal I2075 male) using QIAGEN Genomic-tip 500/G (Qiagen, Hilden, Germany) according to manufacturer’s instruction. The genomic DNA was fragmented into target size 40 kb, 20 kb, 8 kb, and 3 kb respectively, and fragmentations of 20 kb, 8 kb, and 3 kb were done twice in order to obtain wide variety of breaking points. These fragments were end-repaired, ligated with biotinated adapters, and circularized. These circularized DNA were re-fragmented into around 300 bp which is acceptable size for Illumina HiSeq 2000 sequencing, and then fragments were selected by streptavidin purification. These jumping fragments (mate-pair (MP) libraries), which excluded normal shotgun fragments, were end-repaired and were ligated with library adapters for Illumina sequencing.

### BAC-end preparation and sequencing

BAC library called CJB1 was constructed according to the procedures previously described^24^. Cultured embryonic stem cells, which were established from CIEA marmosets (Animals I992 female and IH554 male), were embedded in 1% agarose gel, treated with SacI, and subjected to pulse-fielded electrophoresis. The DNA fragments ranging from 125 to 225 kb were isolated and ligated with pKS146 vector. Transformation was carried out electronically using E. coli DH10B as a host strain. Ampicillin-resistant transformants were collected and stored in 384-format plates. The end sequences of BAC clones were determined by a capillary sequencer 3730xl with the Sanger method. The BAC DNA from the BAC library was extracted by using the PI-1100 and 1200 (KURABO). Cycle sequencing reaction was performed by using the BigDye Terminator v3.1 Cycle Sequencing Kit according to manufacturer’s instructions (Applied Biosystems). The sequencing reaction solution was purified by isopropanol precipitation and loaded on the ABI 3730xl DNA Analyzers (Applied Biosystems). Base-calling was performed by the KB basecaller v1.4.

### Genome assembly

The resequenced reads from CIEA marmoset were mapped to the MGSAC draft genome using Bowtie 2.2.2^25^. Unmapped reads were extracted using the SAMtools 0.1.18^26^ “view” command with the “-f 4” option. The unmapped reads were assembled into contigs that are not contained in the MGSAC draft genome using SOAPdenovo2 r240^14^ with the multi k-mer size ranging from 55 to 77.

The contigs that were used to assemble the MGSAC draft genome were downloaded from NCBI (Accession ID: ACFV00000000.1) and have estimated coverage of only 6×. Therefore, both sets of contigs were combined to fill the gaps in the draft genome with our reads generated by high-throughput sequencers.

Scaffolds were generated from both sets of contigs using SSPACE 3.0^16^ with MP reads with insert lengths of 3, 8, 20, and 40 K. Since the MP reads were high coverage, more contigs could be joined so that longer scaffolds could be obtained.

The high coverage Illumina reads can be used to fill the gaps in the scaffolds; GapColoser v1.12-r6^14^ was employed for this purpose. If a gap in a scaffold is filled by short reads, then a longer contig can be obtained. Otherwise, the scaffold was split into several contigs at the remaining gaps. In either case, the contigs could be improved in comparison with the original MGSAC contigs.

The improved contigs were mapped to the MGSAC draft genome using LAST 531^17^ to reconstruct chromosomes. Every region in the draft genome in which a contig was mapped was replaced by the mapped contig. If both ends of a contig were mapped to the upstream and downstream regions of a gap, the gap could be filled by the contig.

The improved contigs originated from 2 common marmosets, meaning that the generated genome is chimeric. To fix the chimeric genome, all CIEA short reads were mapped to the chimeric genome using BWA-MEM 0.7.9a^13^, and variation analysis was performed with the SAMtools 0.1.18 “mpileup” command with “-q 20” (to skip alignments with map Q < 20) and the VCFutils “varFilter” command with “-D100” (to filter out sites with a depth > 100) to call SNPs and insertions-deletions. The detected positions of variations were replaced by the CIEA variations. Thus, the CIEA-based common marmoset genome was generated.

### Gene feature annotation

Gene features were annotated in the improved genome sequence using 3 approaches: *ab initio*, evidence-based, and homology-based predictions. For the *ab initio* prediction, AUGUSTUS 3.0.3^23^ was employed with the predefined parameter set for the human genome, which is the nearest species among the predefined parameter sets.

For the evidence-based prediction of transcripts, STAR 2.4.0-f1^22^ and Cufflinks 2.2.1^20^ were employed with the default parameters. STAR was used to map RNA-seq reads from 5 organs (SRA accession IDs: SRX285538, SRX285591, SRX285592, SRX285593, and SRX285594) produced by NHPRTR. Cufflinks predicted transcripts for each organ and all sets of transcripts were merged using Cuffmerge.

For the homology-based prediction of transcripts, marmoset and human cDNAs downloaded from Ensembl 78 were used^19^. BLAT 35^18^ was employed with the default parameters to map cDNA sequences to the improved genome.

### SNP detection and genetic distance calculation

To estimate the genetic distance between the CIEA marmoset (Animal I2075 male) and 9 marmosets analyzed by MGSAC (SRA accession IDs: SRS602594 and SRS603590 from the New England Regional Primate Research Center; SRS603947 and SRS604114 from the Wisconsin National Primate Research Center; and SRS602854, SRS603862, SRS603863, SRS603901, and SRS603924 from the Southwest NPRC), the pairwise allele-sharing distance was calculated using the SNPRelate package^27^. Our resequenced read data and data deposited in NCBI BioProject 13630 were used for calling SNPs. All reads were mapped to the MGSAC draft genome by BWA-MEM. Biallelic SNPs were called by the SAMtools 0.1.18 “mpileup” command with “-q 20” and the VCFutils “varFilter” command with “-D100”. SNPs with linkage disequilibrium (r^2^) > 0.2 were filtered out with a 500-Kbp sliding window, leaving 16,735 autosomal SNPs. The pairwise allele-sharing distance matrix was calculated from the detected SNPs using the SNPRelate package, and principal component analysis was performed.

## Additional Information

**Accession code:** All sequencing data used in this work are available from the DNA DataBank of Japan (DDBJ) Sequence Read Archive (DRA) under the accession number DRA003594, and the improved genome sequences are available from accession numbers BBXK01000001-BBXK01109198 (contigs) and DG000097-DG000120 (scaffolds). The BAC-end sequences are available from accession numbers LB274659-LB427105.

**How to cite this article**: Sato, K. *et al.* Resequencing of the common marmoset genome improves genome assemblies and gene-coding sequence analysis. *Sci. Rep.* **5**, 16894; doi: 10.1038/srep16894 (2015).

## Supplementary Material

Supplementary Information

## Figures and Tables

**Table 1:** Summary of sequence reads.

	length	insert	raw			quality filter		
			# of reads	total bases	coverage	# of reads	total bases	coverage
Illumina GAIIx								
SE	115		71 M	8 G	2.7×	71 M	7 G	2.4×
PE	150	500	363 M	55 G	18×	337 M	39 G	13×
PE	115	500	441 M	51 G	17×	423 M	45 G	15×
PE	115	700	460 M	53 G	18×	445 M	49 G	16×
Illumina HiSeq2000								
PE	100	3 K				219 M	18 G	5.9×
PE	100	8 K				118 M	10 G	3.2×
PE	100	20 K				122 M	10 G	3.3×
PE	100	40 K				41 M	3 G	1.1×

**Table 2:** Statistics of assembly results with Illumina SE, PE and MP reads.

	# of contigs	N50	# of gaps	total gap length
Our improved draft genome	104 K	61,143	122 K	129,679,131 bp
MGSAC draft (caljac-3.2)	201 K	29,273	187 K	162,452,744 bp

**Table 3:** Statistics of gene annotations in the improved genome sequence.

	Ensembl annotations from MGSAC	marmoset cDNA	human cDNA	ab initio by AUGUSTUS	RNA-seq
# of transcripts	52,754	45,432	116,826	32,464	78,227
# of completed	5,187	0	5,288	12,209	8,316

**Table 4:** The total number of the improved (CIEA) contigs, the total number of MGSAC contigs mapped to each chromosome in the improved genome, and the number of MGSAC contigs newly mapped to each chromosome in the improved genome but remained unmapped in the MGSAC draft genome.

	chr 1	chr 2	chr 3	chr 4	chr 5	chr 6	chr 7	chr 8
# of CIEA contigs	5,310	4,406	3,877	3,866	5,366	3,581	4,472	3,685
# of MGSAC contigs	12,214	10,822	9,257	8,957	11,671	8,369	10,135	7,482
# of MGSAC contigs newly mapped	876	519	845	792	1,020	732	881	1,452
	**chr 9**	**chr 10**	**chr 11**	**chr 12**	**chr 13**	**chr 14**	**chr 15**	**chr 16**
	3,928	2,931	3,571	3,352	2,459	2,519	2,242	2,208
	8,364	7,513	7,836	7,766	6,050	5,988	5,248	5,028
	1,138	328	586	419	456	429	458	437
	**chr 17**	**chr 18**	**chr 19**	**chr 20**	**chr 21**	**chr 22**	**chr X**	**chr Y**
	1,450	1,630	1,288	1,331	1,312	3,480	12,410	4,758
	3,562	3,186	2,961	3,018	2,781	6,192	17,389	5,290
	262	564	246	193	293	282	7,893	5,042
