# Supplementary material for: Resequencing of the common marmoset genome improves genome assemblies and gene-coding sequence analysis
Source: Sci Rep. 2015 Nov 20;5:16894. doi: 10.1038/srep16894 (PMC4653617; doi:10.1038/srep16894)
Supplement: Supplementary Information [file srep16894-s1.pdf]

Supplementary information:

“Resequencing of the common marmoset genome improves genome assemblies and gene-coding sequence analysis”

Kengo Sato, Yoko Kuroki, Wakako Kumita, Asao Fujiyama, Atsushi Toyoda, Jun Kawai, Atsushi Iriki, Erika Sasaki, Hideyuki Okano, Yasubumi Sakakibara*

Supplemental Figure

Supplemental figure 1. The distribution of insert sizes in the mapped BAC-ends.

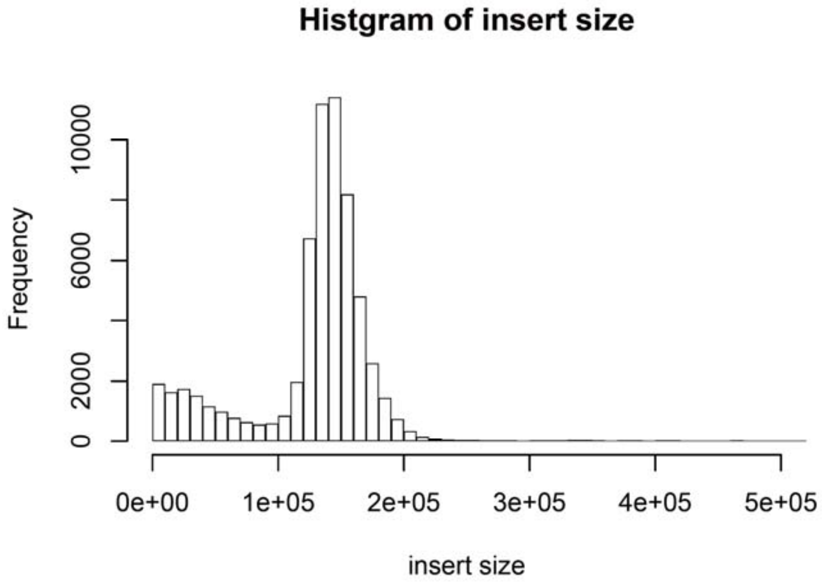

Supplemental figure 2. Histgrams of mapping rate of length for marmoset and human cDNAs to the improved genome sequence. We discarded cDNAs of which mapping rate is <80%.

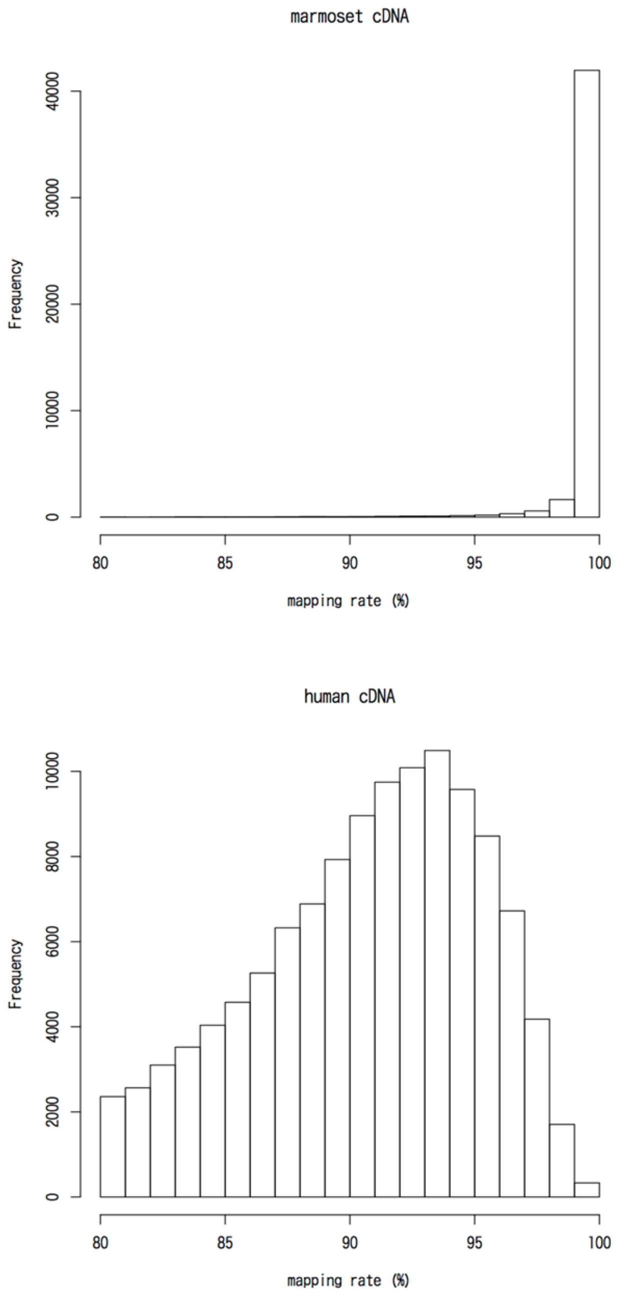

Supplemental figure 3. The Venn diagram that shows how human cDNA transcripts mapped into the improved (CIEA) genome sequence overlap with the other four annotations.

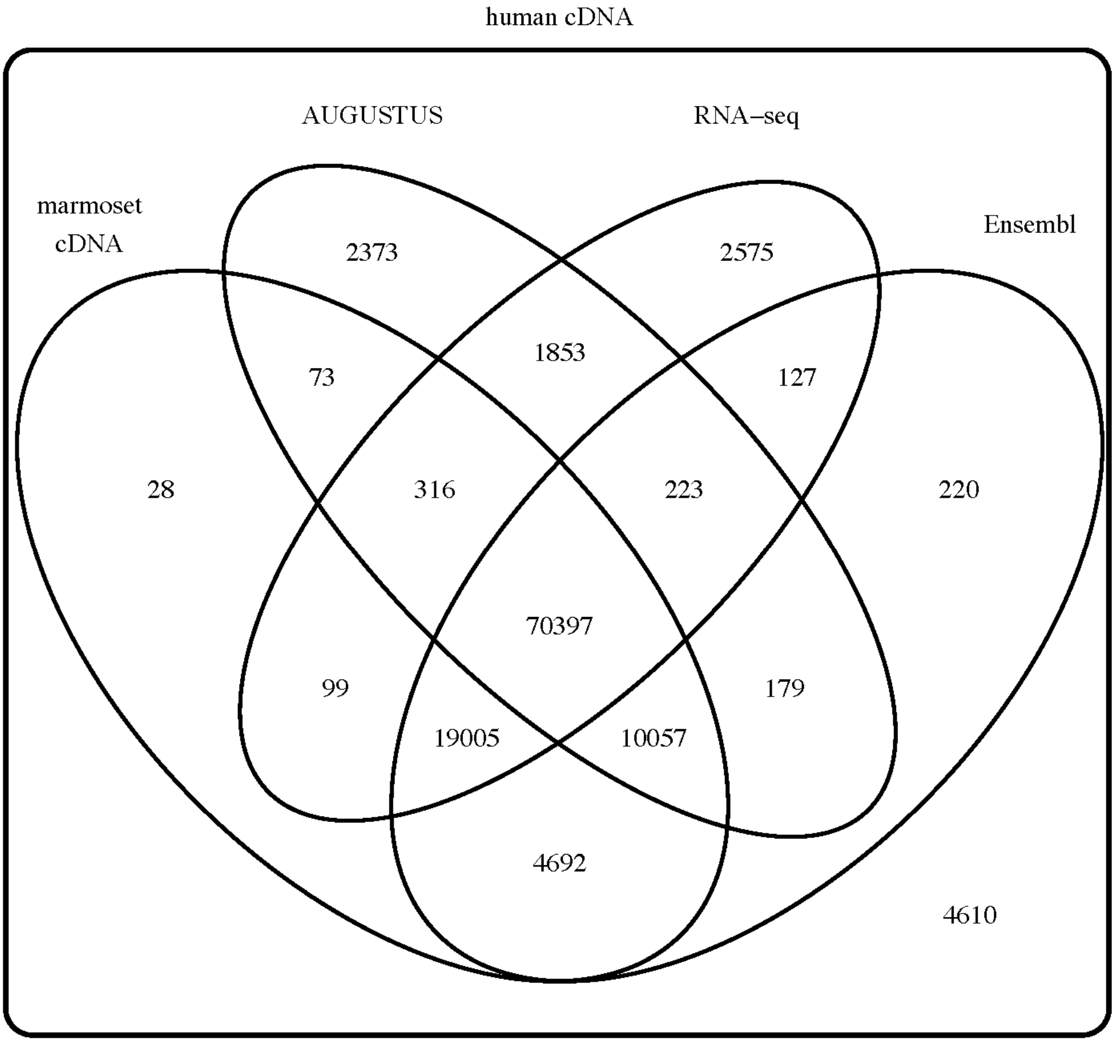

Supplemental Tables

Supplemental table 1. The number and location of the MGSAC contigs that have been previously unmapped to chromosomes in the MGSAC draft but mapped to each chromosome in the improved genome.

		The relative positions on chromosome from 5'-end (binned by every 10% of the total length of chromosome)									
chr	# of contigs	0%-10%	10%-20%	20%-30%	30%-40%	40%-50%	50%-60%	60%-70%	70%-80%	80%-90%	90%-100%
chr1	12214	906	1063	897	1141	1022	850	1034	1116	2044	2141
chr2	10822	1983	1058	1169	1257	791	768	959	793	878	1166
chr3	9257	981	838	843	851	799	858	827	1075	804	1381
chr4	8957	1081	1146	1165	742	806	695	811	658	743	1110
chr5	11671	1145	1106	1193	1204	1282	1169	1249	1232	1197	894
chr6	8369	1050	993	755	676	643	730	720	783	844	1175
chr7	10135	941	848	1378	1514	1311	1043	711	652	772	965
chr8	7482	789	756	597	555	882	697	632	812	784	978
chr9	8364	802	858	671	1145	576	539	690	710	1062	1311
chr10	7513	908	758	684	671	640	607	676	739	629	1201
chr11	7836	667	801	562	554	702	937	545	584	861	1623
chr12	7766	1164	888	903	719	719	455	650	711	622	935
chr13	6050	717	583	558	750	593	557	439	591	580	682
chr14	5988	605	716	477	614	512	515	586	671	520	772
chr15	5248	636	479	770	483	425	349	636	592	450	428
chr16	5028	637	429	405	473	436	756	509	478	409	496
chr17	3562	376	337	353	300	333	346	300	312	343	562
chr18	3186	416	465	392	431	214	343	202	229	257	237
chr19	2961	338	231	292	299	316	350	323	286	259	267
chr20	3018	290	229	321	145	313	337	280	245	356	502
chr21	2781	272	214	221	148	192	251	241	316	338	588
chr22	6192	898	687	688	620	373	423	557	637	685	624
chrX	17389	1396	1414	1408	1870	2342	1752	1794	2087	1612	1714
chrY	5290	437	591	72	229	313	127	1735	473	534	779

Supplemental table 2. The list of the completely filled transcripts. The first, second, and third columns represent Ensembl transcript_id, gene_id, and gene_name, respectively.

ENSCJAT00000061333	ENSCJAG00000003206	unknown
ENSCJAT00000059015	ENSCJAG00000003206	unknown
ENSCJAT00000006167	ENSCJAG00000003211	GPC5
ENSCJAT00000006561	ENSCJAG00000003402	DNAJC3
ENSCJAT00000040975	ENSCJAG00000020832	SLC15A1
ENSCJAT00000040942	ENSCJAG00000020832	SLC15A1
ENSCJAT00000041309	ENSCJAG00000021010	GGACT
ENSCJAT00000039563	ENSCJAG00000020115	TPP2
ENSCJAT00000066098	ENSCJAG00000020115	TPP2
ENSCJAT00000039568	ENSCJAG00000020115	TPP2
ENSCJAT00000028646	ENSCJAG00000014720	DAOA
ENSCJAT00000028573	ENSCJAG00000014685	ABHD13
ENSCJAT00000028570	ENSCJAG00000014666	TNFSF13B
ENSCJAT00000028558	ENSCJAG00000014666	TNFSF13B
ENSCJAT00000027358	ENSCJAG00000014083	TEX29
ENSCJAT00000013163	ENSCJAG00000006736	F10
ENSCJAT00000013194	ENSCJAG00000006758	PROZ
ENSCJAT00000063506	ENSCJAG00000006758	PROZ
ENSCJAT00000013456	ENSCJAG00000006831	CUL4A
ENSCJAT00000053496	ENSCJAG00000006950	TFDP1
ENSCJAT00000052822	ENSCJAG00000033547	unknown
ENSCJAT00000037452	ENSCJAG00000019102	unknown
ENSCJAT00000037468	ENSCJAG00000019102	unknown
ENSCJAT00000037466	ENSCJAG00000019102	unknown
ENSCJAT00000058958	ENSCJAG00000019102	unknown
ENSCJAT00000041838	ENSCJAG00000021261	RBM26
ENSCJAT00000041848	ENSCJAG00000021261	RBM26
ENSCJAT00000041711	ENSCJAG00000021196	MYCBP2
ENSCJAT00000041651	ENSCJAG00000021176	UCHL3
ENSCJAT00000055488	ENSCJAG00000021175	COMMD6
ENSCJAT00000041650	ENSCJAG00000021175	COMMD6
ENSCJAT00000041500	ENSCJAG00000021109	MZT1
ENSCJAT00000016732	ENSCJAG00000008585	RNASEH2B
ENSCJAT00000016745	ENSCJAG00000008585	RNASEH2B
ENSCJAT00000037812	ENSCJAG00000008616	DLEU7
ENSCJAT00000016762	ENSCJAG00000008619	DLEU1
ENSCJAT00000016943	ENSCJAG00000008641	unknown
ENSCJAT00000017008	ENSCJAG00000008764	EBPL
ENSCJAT00000017112	ENSCJAG00000008772	RCBTB1
ENSCJAT00000017124	ENSCJAG00000008839	PHF11
ENSCJAT00000017219	ENSCJAG00000008864	SETDB2
ENSCJAT00000017212	ENSCJAG00000008864	SETDB2
ENSCJAT00000017340	ENSCJAG00000008925	CDADC1
ENSCJAT00000063077	ENSCJAG00000008925	CDADC1
ENSCJAT00000017354	ENSCJAG00000008970	MLNR
ENSCJAT00000017359	ENSCJAG00000008970	MLNR
ENSCJAT00000017413	ENSCJAG00000008978	FNDC3A
ENSCJAT00000017899	ENSCJAG00000009158	ITM2B
ENSCJAT00000032669	ENSCJAG00000016797	CPB2
ENSCJAT00000032680	ENSCJAG00000016797	CPB2
ENSCJAT00000011021	ENSCJAG00000005666	SYK
ENSCJAT00000011025	ENSCJAG00000005666	SYK
ENSCJAT00000015244	ENSCJAG00000007792	unknown
ENSCJAT00000055867	ENSCJAG00000007883	SEMA4D
ENSCJAT00000015415	ENSCJAG00000007883	SEMA4D
ENSCJAT00000040204	ENSCJAG00000020481	NOL8
ENSCJAT00000060984	ENSCJAG00000008862	ERCC6L2
ENSCJAT00000017213	ENSCJAG00000008862	ERCC6L2
ENSCJAT00000055669	ENSCJAG00000008862	ERCC6L2

ENSCJAT00000017273	ENSCJAG00000008899	HSD17B3
ENSCJAT00000017267	ENSCJAG00000008899	HSD17B3
ENSCJAT00000007475	ENSCJAG00000003868	SLC28A3
ENSCJAT00000053138	ENSCJAG00000003868	SLC28A3
ENSCJAT00000006375	ENSCJAG00000003319	PSAT1
ENSCJAT00000006348	ENSCJAG00000003289	CEP78
ENSCJAT00000006344	ENSCJAG00000003289	CEP78
ENSCJAT00000026800	ENSCJAG00000003289	CEP78
ENSCJAT00000063826	ENSCJAG00000003147	VPS13A
ENSCJAT00000006237	ENSCJAG00000003147	VPS13A
ENSCJAT00000061396	ENSCJAG00000003147	VPS13A
ENSCJAT00000024799	ENSCJAG00000012794	NMRK1
ENSCJAT00000024809	ENSCJAG00000012794	NMRK1
ENSCJAT00000002297	ENSCJAG00000001216	RORB
ENSCJAT00000002300	ENSCJAG00000001216	RORB
ENSCJAT00000004070	ENSCJAG00000001441	C9orf85
ENSCJAT00000038340	ENSCJAG00000010690	DMRT2
ENSCJAT00000058429	ENSCJAG00000010690	DMRT2
ENSCJAT00000020842	ENSCJAG00000010690	DMRT2
ENSCJAT00000020825	ENSCJAG00000010690	DMRT2
ENSCJAT00000020814	ENSCJAG00000010690	DMRT2
ENSCJAT00000021260	ENSCJAG00000010914	KCNV2
ENSCJAT00000021255	ENSCJAG00000010914	KCNV2
ENSCJAT00000021723	ENSCJAG00000011136	SPATA6L
ENSCJAT00000021717	ENSCJAG00000011136	SPATA6L
ENSCJAT00000059118	ENSCJAG00000011136	SPATA6L
ENSCJAT00000052659	ENSCJAG00000011136	SPATA6L
ENSCJAT00000021773	ENSCJAG00000011155	CDC37L1
ENSCJAT00000022085	ENSCJAG00000011358	INSL6
ENSCJAT00000061953	ENSCJAG00000011388	CD274
ENSCJAT00000022190	ENSCJAG00000011388	CD274
ENSCJAT00000022181	ENSCJAG00000011388	CD274
ENSCJAT00000022160	ENSCJAG00000011388	CD274
ENSCJAT00000022516	ENSCJAG00000011596	MLANA
ENSCJAT00000023073	ENSCJAG00000011840	PTPRD
ENSCJAT00000023170	ENSCJAG00000011941	LURAP1L
ENSCJAT00000023167	ENSCJAG00000011941	LURAP1L
ENSCJAT00000052887	ENSCJAG00000011950	MPDZ
ENSCJAT00000055177	ENSCJAG00000011950	MPDZ
ENSCJAT00000031630	ENSCJAG00000011950	MPDZ
ENSCJAT00000052328	ENSCJAG00000011950	MPDZ
ENSCJAT00000023552	ENSCJAG00000011950	MPDZ
ENSCJAT00000023516	ENSCJAG00000011950	MPDZ
ENSCJAT00000023541	ENSCJAG00000011950	MPDZ
ENSCJAT00000023637	ENSCJAG00000012155	NFIB
ENSCJAT00000023658	ENSCJAG00000012155	NFIB
ENSCJAT00000023620	ENSCJAG00000012155	NFIB
ENSCJAT00000023628	ENSCJAG00000012155	NFIB
ENSCJAT00000023572	ENSCJAG00000012155	NFIB
ENSCJAT00000024362	ENSCJAG00000012578	BNC2
ENSCJAT00000024374	ENSCJAG00000012578	BNC2
ENSCJAT00000024698	ENSCJAG00000012733	unknown
ENSCJAT00000015663	ENSCJAG00000008021	unknown
ENSCJAT00000035986	ENSCJAG00000018391	LRRC19
ENSCJAT00000015761	ENSCJAG00000008074	BAG1
ENSCJAT00000015953	ENSCJAG00000008175	unknown
ENSCJAT00000016164	ENSCJAG00000008222	NOL6
ENSCJAT00000016160	ENSCJAG00000008222	NOL6
ENSCJAT00000016158	ENSCJAG00000008222	NOL6

ENSCJAT00000016099	ENSCJAG00000008222	NOL6
ENSCJAT00000016466	ENSCJAG00000008463	DCAF12
ENSCJAT00000017373	ENSCJAG00000008968	DNAJB5
ENSCJAT00000056193	ENSCJAG00000008968	DNAJB5
ENSCJAT00000017368	ENSCJAG00000008968	DNAJB5
ENSCJAT00000061402	ENSCJAG00000008968	DNAJB5
ENSCJAT00000053650	ENSCJAG00000008968	DNAJB5
ENSCJAT00000018537	ENSCJAG00000009538	CCDC107
ENSCJAT00000018577	ENSCJAG00000009573	ARHGEF39
ENSCJAT00000018636	ENSCJAG00000009601	CA9
ENSCJAT00000059925	ENSCJAG00000009601	CA9
ENSCJAT00000019240	ENSCJAG00000009895	GBA2
ENSCJAT00000019564	ENSCJAG00000010068	HINT2
ENSCJAT00000019656	ENSCJAG00000010090	TMEM8B
ENSCJAT00000064681	ENSCJAG00000037770	unknown
ENSCJAT00000020134	ENSCJAG00000010299	RNF38
ENSCJAT00000020114	ENSCJAG00000010299	RNF38
ENSCJAT00000020119	ENSCJAG00000010299	RNF38
ENSCJAT00000020464	ENSCJAG00000010502	ZBTB5
ENSCJAT00000020881	ENSCJAG00000013514	unknown
ENSCJAT00000021100	ENSCJAG00000010818	TSTD2
ENSCJAT00000021270	ENSCJAG00000010918	FOXE1
ENSCJAT00000063022	ENSCJAG00000010940	HEMGN
ENSCJAT00000059517	ENSCJAG00000010940	HEMGN
ENSCJAT00000054527	ENSCJAG00000010940	HEMGN
ENSCJAT00000021321	ENSCJAG00000010940	HEMGN
ENSCJAT00000021373	ENSCJAG00000010946	ANP32B
ENSCJAT00000021355	ENSCJAG00000010946	ANP32B
ENSCJAT00000057896	ENSCJAG00000010946	ANP32B
ENSCJAT00000021407	ENSCJAG00000010966	NANS
ENSCJAT00000021400	ENSCJAG00000010966	NANS
ENSCJAT00000022220	ENSCJAG00000011404	INVS
ENSCJAT00000031692	ENSCJAG00000016292	BAAT
ENSCJAT00000031973	ENSCJAG00000016354	RNF20
ENSCJAT00000031995	ENSCJAG00000016354	RNF20
ENSCJAT00000032112	ENSCJAG00000016490	CYLC2
ENSCJAT00000032090	ENSCJAG00000016490	CYLC2
ENSCJAT00000032071	ENSCJAG00000016490	CYLC2
ENSCJAT00000032583	ENSCJAG00000016653	ABCA1
ENSCJAT00000032609	ENSCJAG00000016777	EIF4A3
ENSCJAT00000032614	ENSCJAG00000016777	EIF4A3
ENSCJAT00000032814	ENSCJAG00000016868	TMEM38B
ENSCJAT00000039474	ENSCJAG00000016908	unknown
ENSCJAT00000011901	ENSCJAG00000016908	unknown
ENSCJAT00000032945	ENSCJAG00000016908	unknown
ENSCJAT00000032941	ENSCJAG00000016908	unknown
ENSCJAT00000055539	ENSCJAG00000016966	IKBKAP
ENSCJAT00000033118	ENSCJAG00000016966	IKBKAP
ENSCJAT00000033111	ENSCJAG00000016966	IKBKAP
ENSCJAT00000033109	ENSCJAG00000016966	IKBKAP
ENSCJAT00000033315	ENSCJAG00000017065	CTNNAL1
ENSCJAT00000033685	ENSCJAG00000017317	C9orf152
ENSCJAT00000033714	ENSCJAG00000017329	TXNDC8
ENSCJAT00000033737	ENSCJAG00000017337	unknown
ENSCJAT00000034213	ENSCJAG00000017561	unknown
ENSCJAT00000053244	ENSCJAG00000033590	unknown
ENSCJAT00000034609	ENSCJAG00000017757	SLC46A2
ENSCJAT00000034783	ENSCJAG00000017793	FKBP15
ENSCJAT00000034775	ENSCJAG00000017793	FKBP15

ENSCJAT00000034936	ENSCJAG00000017894	ALAD
ENSCJAT00000034941	ENSCJAG00000017894	ALAD
ENSCJAT00000034944	ENSCJAG00000017894	ALAD
ENSCJAT00000035194	ENSCJAG00000018027	unknown
ENSCJAT00000040213	ENSCJAG00000020473	AKNA
ENSCJAT00000052356	ENSCJAG00000020461	ATP6V1G1
ENSCJAT00000040156	ENSCJAG00000020453	TNFSF8
ENSCJAT00000039944	ENSCJAG00000020344	MEGF9
ENSCJAT00000039858	ENSCJAG00000020298	TRAF1
ENSCJAT00000039691	ENSCJAG00000020202	STOM
ENSCJAT00000063324	ENSCJAG00000020202	STOM
ENSCJAT00000039673	ENSCJAG00000020202	STOM
ENSCJAT00000039685	ENSCJAG00000020202	STOM
ENSCJAT00000039690	ENSCJAG00000020202	STOM
ENSCJAT00000058112	ENSCJAG00000019997	STRBP
ENSCJAT00000039332	ENSCJAG00000019997	STRBP
ENSCJAT00000013132	ENSCJAG00000006730	ARPC5L
ENSCJAT00000013818	ENSCJAG00000007059	MVB12B
ENSCJAT00000062647	ENSCJAG00000007059	MVB12B
ENSCJAT00000035349	ENSCJAG00000018088	LRSAM1
ENSCJAT00000038931	ENSCJAG00000019817	STXBP1
ENSCJAT00000038939	ENSCJAG00000019835	C9orf117
ENSCJAT00000039117	ENSCJAG00000019920	FAM102A
ENSCJAT00000061121	ENSCJAG00000019948	CIZ1
ENSCJAT00000052859	ENSCJAG00000019948	CIZ1
ENSCJAT00000039208	ENSCJAG00000019948	CIZ1
ENSCJAT00000057054	ENSCJAG00000019948	CIZ1
ENSCJAT00000057588	ENSCJAG00000019948	CIZ1
ENSCJAT00000039271	ENSCJAG00000019967	DNM1
ENSCJAT00000039316	ENSCJAG00000020009	TRUB2
ENSCJAT00000039314	ENSCJAG00000020009	TRUB2
ENSCJAT00000039361	ENSCJAG00000020036	URM1
ENSCJAT00000039642	ENSCJAG00000020165	PKN3
ENSCJAT00000039403	ENSCJAG00000020054	PHYHD1
ENSCJAT00000039394	ENSCJAG00000020054	PHYHD1
ENSCJAT00000039860	ENSCJAG00000020303	PRRX2
ENSCJAT00000016474	ENSCJAG00000008470	FAM78A
ENSCJAT00000018968	ENSCJAG00000009774	REXO4
ENSCJAT00000063450	ENSCJAG00000009774	REXO4
ENSCJAT00000054317	ENSCJAG00000009791	ADAMTS13
ENSCJAT00000019630	ENSCJAG00000010099	unknown
ENSCJAT00000019817	ENSCJAG00000010186	FCN2
ENSCJAT00000019804	ENSCJAG00000010186	FCN2
ENSCJAT00000020839	ENSCJAG00000010692	LHX3
ENSCJAT00000020835	ENSCJAG00000010692	LHX3
ENSCJAT00000059302	ENSCJAG00000011179	TRAF2
ENSCJAT00000024846	ENSCJAG00000012814	CLIC3
ENSCJAT00000025345	ENSCJAG00000013053	SSNA1
ENSCJAT00000025838	ENSCJAG00000013219	NSMF
ENSCJAT00000025839	ENSCJAG00000013219	NSMF
ENSCJAT00000025895	ENSCJAG00000013219	NSMF
ENSCJAT00000025837	ENSCJAG00000013219	NSMF
ENSCJAT00000025896	ENSCJAG00000013219	NSMF
ENSCJAT00000025856	ENSCJAG00000013219	NSMF
ENSCJAT00000033518	ENSCJAG00000017229	unknown
ENSCJAT00000033860	ENSCJAG00000017386	TUBA8
ENSCJAT00000028387	ENSCJAG00000037354	GNB1L
ENSCJAT00000033799	ENSCJAG00000015834	unknown
ENSCJAT00000030822	ENSCJAG00000015834	unknown

ENSCJAT00000055339	ENSCJAG00000015818	unknown
ENSCJAT00000030643	ENSCJAG00000015743	MAPK1
ENSCJAT00000018927	ENSCJAG00000009762	RAB36
ENSCJAT00000018917	ENSCJAG00000009762	RAB36
ENSCJAT00000059558	ENSCJAG00000009592	unknown
ENSCJAT00000059220	ENSCJAG00000009592	unknown
ENSCJAT00000058396	ENSCJAG00000009592	unknown
ENSCJAT00000018116	ENSCJAG00000009348	CRYBB3
ENSCJAT00000039622	ENSCJAG00000020170	MN1
ENSCJAT00000039527	ENSCJAG00000020131	unknown
ENSCJAT00000060601	ENSCJAG00000020088	CCDC117
ENSCJAT00000039451	ENSCJAG00000020088	CCDC117
ENSCJAT00000055235	ENSCJAG00000020088	CCDC117
ENSCJAT00000039459	ENSCJAG00000020088	CCDC117
ENSCJAT00000038929	ENSCJAG00000019829	CABP7
ENSCJAT00000056004	ENSCJAG00000010370	SF3A1
ENSCJAT00000020289	ENSCJAG00000010413	CCDC157
ENSCJAT00000057660	ENSCJAG00000010413	CCDC157
ENSCJAT00000020712	ENSCJAG00000010617	PES1
ENSCJAT00000020889	ENSCJAG00000010729	MORC2
ENSCJAT00000052587	ENSCJAG00000010729	MORC2
ENSCJAT00000020946	ENSCJAG00000010736	SMTN
ENSCJAT00000021395	ENSCJAG00000010973	PIK3IP1
ENSCJAT00000059919	ENSCJAG00000010973	PIK3IP1
ENSCJAT00000054100	ENSCJAG00000010973	PIK3IP1
ENSCJAT00000021492	ENSCJAG00000011009	DRG1
ENSCJAT00000021733	ENSCJAG00000011154	PISD
ENSCJAT00000021747	ENSCJAG00000011163	MA_M004_JSM71693R
ENSCJAT00000038549	ENSCJAG00000035365	unknown
ENSCJAT00000022144	ENSCJAG00000011369	SLC5A1
ENSCJAT00000012947	ENSCJAG00000011369	SLC5A1
ENSCJAT00000002048	ENSCJAG00000011369	SLC5A1
ENSCJAT00000000966	ENSCJAG00000000519	TOM1
ENSCJAT00000052334	ENSCJAG00000000519	TOM1
ENSCJAT00000000940	ENSCJAG00000000516	HMOX1
ENSCJAT00000027126	ENSCJAG00000013961	IFT27
ENSCJAT00000004051	ENSCJAG00000002140	C1QTNF6
ENSCJAT00000061179	ENSCJAG00000002140	C1QTNF6
ENSCJAT00000004076	ENSCJAG00000002144	RAC2
ENSCJAT00000056551	ENSCJAG00000000513	GGA1
ENSCJAT00000024266	ENSCJAG00000012524	APOBEC3H
ENSCJAT00000024260	ENSCJAG00000012524	APOBEC3H
ENSCJAT00000023457	ENSCJAG00000012085	unknown
ENSCJAT00000065393	ENSCJAG00000012085	unknown
ENSCJAT00000023487	ENSCJAG00000012085	unknown
ENSCJAT00000062281	ENSCJAG00000011999	PLA2G6
ENSCJAT00000022797	ENSCJAG00000011737	CBY1
ENSCJAT00000022759	ENSCJAG00000011713	JOSD1
ENSCJAT00000009266	ENSCJAG00000004770	EP300
ENSCJAT00000009314	ENSCJAG00000004823	CHADL
ENSCJAT00000009331	ENSCJAG00000004823	CHADL
ENSCJAT00000009321	ENSCJAG00000004823	CHADL
ENSCJAT00000018748	ENSCJAG00000009668	RRP7A
ENSCJAT00000018754	ENSCJAG00000009668	RRP7A
ENSCJAT00000037505	ENSCJAG00000019118	MIOX
ENSCJAT00000037481	ENSCJAG00000019118	MIOX
ENSCJAT00000037626	ENSCJAG00000019187	KLHDC7B
ENSCJAT00000035965	ENSCJAG00000018368	MAPK8IP2
ENSCJAT00000036021	ENSCJAG00000018406	unknown

ENSCJAT00000019938	ENSCJAG00000015228	ZNF107
ENSCJAT00000019945	ENSCJAG00000033954	unknown
ENSCJAT00000014686	ENSCJAG00000033954	unknown
ENSCJAT00000008170	ENSCJAG00000004252	RABGEF1
ENSCJAT00000008158	ENSCJAG00000004252	RABGEF1
ENSCJAT00000025172	ENSCJAG00000004252	RABGEF1
ENSCJAT00000014981	ENSCJAG00000007674	unknown
ENSCJAT00000032586	ENSCJAG00000016763	WBSCR27
ENSCJAT00000032975	ENSCJAG00000016956	TRIM50
ENSCJAT00000033081	ENSCJAG00000017018	CCL24
ENSCJAT00000062819	ENSCJAG00000012804	POR
ENSCJAT00000031425	ENSCJAG00000016147	unknown
ENSCJAT00000031538	ENSCJAG00000016203	POLR2J
ENSCJAT00000062737	ENSCJAG00000016164	SH2B2
ENSCJAT00000031235	ENSCJAG00000016061	COL26A1
ENSCJAT00000031220	ENSCJAG00000016058	IFT22
ENSCJAT00000030764	ENSCJAG00000016058	IFT22
ENSCJAT00000029573	ENSCJAG00000015136	TFR2
ENSCJAT00000029569	ENSCJAG00000015136	TFR2
ENSCJAT00000060612	ENSCJAG00000015328	TSC22D4
ENSCJAT00000029839	ENSCJAG00000015328	TSC22D4
ENSCJAT00000029898	ENSCJAG00000015363	ZCWPW1
ENSCJAT00000029928	ENSCJAG00000015373	unknown
ENSCJAT00000029930	ENSCJAG00000015373	unknown
ENSCJAT00000059730	ENSCJAG00000015392	STAG3
ENSCJAT00000030105	ENSCJAG00000015439	GPC2
ENSCJAT00000030091	ENSCJAG00000015439	GPC2
ENSCJAT00000055847	ENSCJAG00000015522	TAF6
ENSCJAT00000030224	ENSCJAG00000015522	TAF6
ENSCJAT00000053381	ENSCJAG00000015522	TAF6
ENSCJAT00000041865	ENSCJAG00000015656	ZNF3
ENSCJAT00000030230	ENSCJAG00000015656	ZNF3
ENSCJAT00000000475	ENSCJAG00000015664	ZKSCAN1
ENSCJAT00000030509	ENSCJAG00000015664	ZKSCAN1
ENSCJAT00000053925	ENSCJAG00000015702	TRIM4
ENSCJAT00000030545	ENSCJAG00000015702	TRIM4
ENSCJAT00000052141	ENSCJAG00000015702	TRIM4
ENSCJAT00000010978	ENSCJAG00000015783	ZSCAN25
ENSCJAT00000030702	ENSCJAG00000015783	ZSCAN25
ENSCJAT00000058284	ENSCJAG00000015815	unknown
ENSCJAT00000030818	ENSCJAG00000015836	BUD31
ENSCJAT00000031060	ENSCJAG00000015943	KPNA7
ENSCJAT00000029908	ENSCJAG00000015370	AIMP2
ENSCJAT00000056627	ENSCJAG00000015372	EIF2AK1
ENSCJAT00000029963	ENSCJAG00000015372	EIF2AK1
ENSCJAT00000030074	ENSCJAG00000015455	RAC1
ENSCJAT00000042287	ENSCJAG00000021500	RBAK
ENSCJAT00000057395	ENSCJAG00000018776	IQCE
ENSCJAT00000037364	ENSCJAG00000019025	RNF145
ENSCJAT00000037366	ENSCJAG00000019025	RNF145
ENSCJAT00000054736	ENSCJAG00000019025	RNF145
ENSCJAT00000037354	ENSCJAG00000019025	RNF145
ENSCJAT00000037590	ENSCJAG00000019165	unknown
ENSCJAT00000029721	ENSCJAG00000015200	GABRA1
ENSCJAT00000003132	ENSCJAG00000015200	GABRA1
ENSCJAT00000012371	ENSCJAG00000015200	GABRA1
ENSCJAT00000037320	ENSCJAG00000015200	GABRA1
ENSCJAT00000007335	ENSCJAG00000015200	GABRA1
ENSCJAT00000030046	ENSCJAG00000015401	MAT2B

ENSCJAT00000030049	ENSCJAG00000015401	MAT2B
ENSCJAT00000030042	ENSCJAG00000015401	MAT2B
ENSCJAT00000030012	ENSCJAG00000015401	MAT2B
ENSCJAT00000030246	ENSCJAG00000015548	FBLL1
ENSCJAT00000009270	ENSCJAG00000004796	UBE2B
ENSCJAT00000011469	ENSCJAG00000005904	SLC25A48
ENSCJAT00000011480	ENSCJAG00000005904	SLC25A48
ENSCJAT00000011481	ENSCJAG00000005904	SLC25A48
ENSCJAT00000012533	ENSCJAG00000006418	CDC23
ENSCJAT00000056751	ENSCJAG00000006418	CDC23
ENSCJAT00000013296	ENSCJAG00000006776	ETF1
ENSCJAT00000055629	ENSCJAG00000006776	ETF1
ENSCJAT00000013130	ENSCJAG00000006643	HSPA9
ENSCJAT00000013129	ENSCJAG00000006643	HSPA9
ENSCJAT00000057107	ENSCJAG00000006643	HSPA9
ENSCJAT00000059910	ENSCJAG00000006643	HSPA9
ENSCJAT00000013843	ENSCJAG00000007068	SPATA24
ENSCJAT00000032717	ENSCJAG00000016825	PURA
ENSCJAT00000032703	ENSCJAG00000016823	CYSTM1
ENSCJAT00000032319	ENSCJAG00000016613	SRA1
ENSCJAT00000052780	ENSCJAG00000034409	unknown
ENSCJAT00000032116	ENSCJAG00000016366	IK
ENSCJAT00000032086	ENSCJAG00000016366	IK
ENSCJAT00000032023	ENSCJAG00000016366	IK
ENSCJAT00000035312	ENSCJAG00000018086	HDAC3
ENSCJAT00000035447	ENSCJAG00000018098	ARAP3
ENSCJAT00000060118	ENSCJAG00000018220	RNF14
ENSCJAT00000035655	ENSCJAG00000018220	RNF14
ENSCJAT00000059937	ENSCJAG00000018220	RNF14
ENSCJAT00000035669	ENSCJAG00000018220	RNF14
ENSCJAT00000035756	ENSCJAG00000018271	NDFIP1
ENSCJAT00000035782	ENSCJAG00000018293	SPRY4
ENSCJAT00000035893	ENSCJAG00000018337	NR3C1
ENSCJAT00000035942	ENSCJAG00000018337	NR3C1
ENSCJAT00000055771	ENSCJAG00000018337	NR3C1
ENSCJAT00000035946	ENSCJAG00000018337	NR3C1
ENSCJAT00000041954	ENSCJAG00000021314	LARS
ENSCJAT00000041596	ENSCJAG00000021140	AFAP1L1
ENSCJAT00000041592	ENSCJAG00000021140	AFAP1L1
ENSCJAT00000041597	ENSCJAG00000021140	AFAP1L1
ENSCJAT00000057864	ENSCJAG00000021113	PPARGC1B
ENSCJAT00000041471	ENSCJAG00000021088	HMGXB3
ENSCJAT00000056647	ENSCJAG00000021088	HMGXB3
ENSCJAT00000041442	ENSCJAG00000021077	CSF1R
ENSCJAT00000058609	ENSCJAG00000020985	CD74
ENSCJAT00000041261	ENSCJAG00000020985	CD74
ENSCJAT00000063766	ENSCJAG00000020985	CD74
ENSCJAT00000041260	ENSCJAG00000020985	CD74
ENSCJAT00000041262	ENSCJAG00000020985	CD74
ENSCJAT00000041258	ENSCJAG00000020985	CD74
ENSCJAT00000027137	ENSCJAG00000020969	SYNPO
ENSCJAT00000041206	ENSCJAG00000020969	SYNPO
ENSCJAT00000055010	ENSCJAG00000020967	MYOZ3
ENSCJAT00000058582	ENSCJAG00000020967	MYOZ3
ENSCJAT00000041196	ENSCJAG00000020963	RBM22
ENSCJAT00000041182	ENSCJAG00000020943	DCTN4
ENSCJAT00000041151	ENSCJAG00000020942	SMIM3
ENSCJAT00000060947	ENSCJAG00000020930	ZNF300
ENSCJAT00000041130	ENSCJAG00000020930	ZNF300

ENSCJAT00000041121	ENSCJAG00000020924	GPX3
ENSCJAT00000030394	ENSCJAG00000015590	SLC36A1
ENSCJAT00000030371	ENSCJAG00000015590	SLC36A1
ENSCJAT00000030389	ENSCJAG00000015590	SLC36A1
ENSCJAT00000030327	ENSCJAG00000015562	FAT2
ENSCJAT00000030174	ENSCJAG00000015505	ATOX1
ENSCJAT00000030156	ENSCJAG00000015407	G3BP1
ENSCJAT00000030068	ENSCJAG00000015407	G3BP1
ENSCJAT00000008572	ENSCJAG00000004454	LARP1
ENSCJAT00000008559	ENSCJAG00000004454	LARP1
ENSCJAT00000008574	ENSCJAG00000004454	LARP1
ENSCJAT00000014521	ENSCJAG00000007336	GEMIN5
ENSCJAT00000014270	ENSCJAG00000007257	TTYH3
ENSCJAT00000002661	ENSCJAG00000001423	CHST12
ENSCJAT00000002743	ENSCJAG00000001457	FTSJ2
ENSCJAT00000002741	ENSCJAG00000001457	FTSJ2
ENSCJAT00000002762	ENSCJAG00000001468	ELFN1
ENSCJAT00000030691	ENSCJAG00000015780	C7orf50
ENSCJAT00000030755	ENSCJAG00000015785	CYP2W1
ENSCJAT00000030745	ENSCJAG00000015785	CYP2W1
ENSCJAT00000030798	ENSCJAG00000038117	COX19
ENSCJAT00000030812	ENSCJAG00000015829	GET4
ENSCJAT00000030853	ENSCJAG00000015835	SUN1
ENSCJAT00000063270	ENSCJAG00000009795	CNOT6
ENSCJAT00000036773	ENSCJAG00000018733	TRIM41
ENSCJAT00000036774	ENSCJAG00000018733	TRIM41
ENSCJAT00000036767	ENSCJAG00000018733	TRIM41
ENSCJAT00000036931	ENSCJAG00000018803	BTNL9
ENSCJAT00000036930	ENSCJAG00000018803	BTNL9
ENSCJAT00000036933	ENSCJAG00000018803	BTNL9
ENSCJAT00000036952	ENSCJAG00000018845	MGAT1
ENSCJAT00000059828	ENSCJAG00000018845	MGAT1
ENSCJAT00000063357	ENSCJAG00000018845	MGAT1
ENSCJAT00000013117	ENSCJAG00000006729	DBN1
ENSCJAT00000013404	ENSCJAG00000006845	SLC34A1
ENSCJAT00000054720	ENSCJAG00000006845	SLC34A1
ENSCJAT00000013486	ENSCJAG00000006873	RGS14
ENSCJAT00000013458	ENSCJAG00000006873	RGS14
ENSCJAT00000013483	ENSCJAG00000006873	RGS14
ENSCJAT00000013565	ENSCJAG00000006943	MXD3
ENSCJAT00000055613	ENSCJAG00000012089	CLK4
ENSCJAT00000053063	ENSCJAG00000012089	CLK4
ENSCJAT00000023559	ENSCJAG00000012089	CLK4
ENSCJAT00000053593	ENSCJAG00000012089	CLK4
ENSCJAT00000010388	ENSCJAG00000005367	CPLX2
ENSCJAT00000010417	ENSCJAG00000005384	HRH2
ENSCJAT00000010405	ENSCJAG00000005384	HRH2
ENSCJAT00000037624	ENSCJAG00000019186	BNIP1
ENSCJAT00000037625	ENSCJAG00000019186	BNIP1
ENSCJAT00000037621	ENSCJAG00000019186	BNIP1
ENSCJAT00000037759	ENSCJAG00000019267	UBTD2
ENSCJAT00000065311	ENSCJAG00000019324	SMIM23
ENSCJAT00000038087	ENSCJAG00000019417	GABRP
ENSCJAT00000038070	ENSCJAG00000019417	GABRP
ENSCJAT00000038096	ENSCJAG00000019417	GABRP
ENSCJAT00000038112	ENSCJAG00000019417	GABRP
ENSCJAT00000038105	ENSCJAG00000019417	GABRP
ENSCJAT00000026562	ENSCJAG00000013634	PPP2CA
ENSCJAT00000059073	ENSCJAG00000013297	GDF9

ENSCJAT00000053486	ENSCJAG00000013297	GDF9
ENSCJAT00000025818	ENSCJAG00000013297	GDF9
ENSCJAT00000027990	ENSCJAG00000014398	unknown
ENSCJAT00000037851	ENSCJAG00000019309	ISOC1
ENSCJAT00000043264	ENSCJAG00000022279	CTXN3
ENSCJAT00000057143	ENSCJAG00000022279	CTXN3
ENSCJAT00000037571	ENSCJAG00000019170	C5orf63
ENSCJAT00000016013	ENSCJAG00000008214	unknown
ENSCJAT00000054785	ENSCJAG00000017348	CSNK1G3
ENSCJAT00000052544	ENSCJAG00000017348	CSNK1G3
ENSCJAT00000033887	ENSCJAG00000017348	CSNK1G3
ENSCJAT00000033859	ENSCJAG00000017348	CSNK1G3
ENSCJAT00000033841	ENSCJAG00000017348	CSNK1G3
ENSCJAT00000033514	ENSCJAG00000017196	SNCAIP
ENSCJAT00000032553	ENSCJAG00000016745	TMED7
ENSCJAT00000032546	ENSCJAG00000016742	FEM1C
ENSCJAT00000032464	ENSCJAG00000016675	TRIM36
ENSCJAT00000057226	ENSCJAG00000016675	TRIM36
ENSCJAT00000032416	ENSCJAG00000016659	KCNN2
ENSCJAT00000060280	ENSCJAG00000016598	YTHDC2
ENSCJAT00000032120	ENSCJAG00000016517	unknown
ENSCJAT00000032079	ENSCJAG00000016440	APC
ENSCJAT00000032094	ENSCJAG00000016440	APC
ENSCJAT00000031634	ENSCJAG00000016257	SLC25A46
ENSCJAT00000031639	ENSCJAG00000016257	SLC25A46
ENSCJAT00000063008	ENSCJAG00000016257	SLC25A46
ENSCJAT00000017926	ENSCJAG00000009127	PPIP5K2
ENSCJAT00000018231	ENSCJAG00000009287	PAM
ENSCJAT00000018232	ENSCJAG00000009287	PAM
ENSCJAT00000018234	ENSCJAG00000009287	PAM
ENSCJAT00000018088	ENSCJAG00000009287	PAM
ENSCJAT00000018343	ENSCJAG00000009287	PAM
ENSCJAT00000018337	ENSCJAG00000009287	PAM
ENSCJAT00000018318	ENSCJAG00000009287	PAM
ENSCJAT00000018501	ENSCJAG00000009554	FAM174A
ENSCJAT00000018717	ENSCJAG00000009559	CHD1
ENSCJAT00000018702	ENSCJAG00000009559	CHD1
ENSCJAT00000019014	ENSCJAG00000009751	ERAP2
ENSCJAT00000019015	ENSCJAG00000009751	ERAP2
ENSCJAT00000020230	ENSCJAG00000010388	ANKRD32
ENSCJAT00000020317	ENSCJAG00000010418	KIAA0825
ENSCJAT00000020297	ENSCJAG00000010418	KIAA0825
ENSCJAT00000020920	ENSCJAG00000010739	LYSMD3
ENSCJAT00000038621	ENSCJAG00000019641	THBS4
ENSCJAT00000038635	ENSCJAG00000019641	THBS4
ENSCJAT00000038638	ENSCJAG00000019641	THBS4
ENSCJAT00000054938	ENSCJAG00000019876	PDE8B
ENSCJAT00000039376	ENSCJAG00000020046	unknown
ENSCJAT00000033121	ENSCJAG00000017020	COL4A3BP
ENSCJAT00000032371	ENSCJAG00000016645	ARHGEF28
ENSCJAT00000032252	ENSCJAG00000016582	TMEM171
ENSCJAT00000063589	ENSCJAG00000016582	TMEM171
ENSCJAT00000032066	ENSCJAG00000016409	TNPO1
ENSCJAT00000032048	ENSCJAG00000016409	TNPO1
ENSCJAT00000032057	ENSCJAG00000016409	TNPO1
ENSCJAT00000052443	ENSCJAG00000016364	PTCD2
ENSCJAT00000031834	ENSCJAG00000016364	PTCD2
ENSCJAT00000054017	ENSCJAG00000016364	PTCD2
ENSCJAT00000055753	ENSCJAG00000016364	PTCD2

ENSCJAT00000031845	ENSCJAG00000016364	PTCD2
ENSCJAT00000031595	ENSCJAG00000016225	OCLN
ENSCJAT00000031588	ENSCJAG00000016225	OCLN
ENSCJAT00000062813	ENSCJAG00000016225	OCLN
ENSCJAT00000031548	ENSCJAG00000016202	MARVELD2
ENSCJAT00000031558	ENSCJAG00000016202	MARVELD2
ENSCJAT00000031286	ENSCJAG00000016075	unknown
ENSCJAT00000031285	ENSCJAG00000016075	unknown
ENSCJAT00000058132	ENSCJAG00000033226	unknown
ENSCJAT00000030807	ENSCJAG00000015812	TRAPPC13
ENSCJAT00000030832	ENSCJAG00000015812	TRAPPC13
ENSCJAT00000030819	ENSCJAG00000015812	TRAPPC13
ENSCJAT00000030663	ENSCJAG00000015759	CENPK
ENSCJAT00000030445	ENSCJAG00000015644	RNF180
ENSCJAT00000053989	ENSCJAG00000015559	IPO11
ENSCJAT00000053837	ENSCJAG00000002120	ANKRD55
ENSCJAT00000003998	ENSCJAG00000002068	IL6ST
ENSCJAT00000003981	ENSCJAG00000002068	IL6ST
ENSCJAT00000003982	ENSCJAG00000002068	IL6ST
ENSCJAT00000003918	ENSCJAG00000002043	IL31RA
ENSCJAT00000003923	ENSCJAG00000002043	IL31RA
ENSCJAT00000003921	ENSCJAG00000002043	IL31RA
ENSCJAT00000003928	ENSCJAG00000002043	IL31RA
ENSCJAT00000003694	ENSCJAG00000001907	SLC38A9
ENSCJAT00000003693	ENSCJAG00000001907	SLC38A9
ENSCJAT00000002818	ENSCJAG00000001490	ISL1
ENSCJAT00000053145	ENSCJAG00000001432	EMB
ENSCJAT00000002644	ENSCJAG00000001413	FGF10
ENSCJAT00000054977	ENSCJAG00000001333	C5orf34
ENSCJAT00000002448	ENSCJAG00000001321	CCL28
ENSCJAT00000059258	ENSCJAG00000001303	HMGCS1
ENSCJAT00000002437	ENSCJAG00000001303	HMGCS1
ENSCJAT00000002351	ENSCJAG00000001260	CCDC152
ENSCJAT00000002344	ENSCJAG00000001260	CCDC152
ENSCJAT00000002228	ENSCJAG00000001211	C5orf51
ENSCJAT00000002226	ENSCJAG00000001211	C5orf51
ENSCJAT00000014348	ENSCJAG00000007279	MROH2B
ENSCJAT00000052938	ENSCJAG00000007279	MROH2B
ENSCJAT00000061523	ENSCJAG00000007279	MROH2B
ENSCJAT00000014437	ENSCJAG00000007341	PRKAA1
ENSCJAT00000014510	ENSCJAG00000007398	TTC33
ENSCJAT00000014503	ENSCJAG00000007398	TTC33
ENSCJAT00000014936	ENSCJAG00000007608	LIFR
ENSCJAT00000035611	ENSCJAG00000018196	SLC1A3
ENSCJAT00000035645	ENSCJAG00000018196	SLC1A3
ENSCJAT00000035640	ENSCJAG00000018196	SLC1A3
ENSCJAT00000036183	ENSCJAG00000018457	PRLR
ENSCJAT00000036181	ENSCJAG00000018457	PRLR
ENSCJAT00000036185	ENSCJAG00000018457	PRLR
ENSCJAT00000036189	ENSCJAG00000018457	PRLR
ENSCJAT00000036236	ENSCJAG00000018495	DNAJC21
ENSCJAT00000036239	ENSCJAG00000018495	DNAJC21
ENSCJAT00000036473	ENSCJAG00000018539	RAI14
ENSCJAT00000027009	ENSCJAG00000018612	unknown
ENSCJAT00000036513	ENSCJAG00000018612	unknown
ENSCJAT00000036819	ENSCJAG00000018740	ZFR
ENSCJAT00000057529	ENSCJAG00000018740	ZFR
ENSCJAT00000036877	ENSCJAG00000018783	MTMR12
ENSCJAT00000036891	ENSCJAG00000018783	MTMR12

ENSCJAT00000036934	ENSCJAG00000018817	GOLPH3
ENSCJAT00000036929	ENSCJAG00000018817	GOLPH3
ENSCJAT00000022784	ENSCJAG00000011702	CDH10
ENSCJAT00000022770	ENSCJAG00000011702	CDH10
ENSCJAT00000058885	ENSCJAG00000033957	unknown
ENSCJAT00000062815	ENSCJAG00000011831	MYO10
ENSCJAT00000021097	ENSCJAG00000010823	FBXL7
ENSCJAT00000017867	ENSCJAG00000009227	DAP
ENSCJAT00000017840	ENSCJAG00000009213	ROPN1L
ENSCJAT00000017814	ENSCJAG00000009091	6-Mar
ENSCJAT00000017819	ENSCJAG00000009091	6-Mar
ENSCJAT00000017783	ENSCJAG00000009091	6-Mar
ENSCJAT00000021920	ENSCJAG00000036709	SLC6A18
ENSCJAT00000034316	ENSCJAG00000017613	AHRR
ENSCJAT00000034276	ENSCJAG00000017583	SDHA
ENSCJAT00000034289	ENSCJAG00000017583	SDHA
ENSCJAT00000021086	ENSCJAG00000010834	ZFP42
ENSCJAT00000054423	ENSCJAG00000020294	F11
ENSCJAT00000039854	ENSCJAG00000020294	F11
ENSCJAT00000039803	ENSCJAG00000020265	CYP4V2
ENSCJAT00000039807	ENSCJAG00000020265	CYP4V2
ENSCJAT00000011768	ENSCJAG00000006057	TLR3
ENSCJAT00000011590	ENSCJAG00000005969	C4orf47
ENSCJAT00000056100	ENSCJAG00000005780	MLCL1
ENSCJAT00000011321	ENSCJAG00000005780	MLCL1
ENSCJAT00000011236	ENSCJAG00000005780	MLCL1
ENSCJAT00000054369	ENSCJAG00000005780	MLCL1
ENSCJAT00000011330	ENSCJAG00000005780	MLCL1
ENSCJAT00000011130	ENSCJAG00000005740	CASP3
ENSCJAT00000055959	ENSCJAG00000005740	CASP3
ENSCJAT00000011114	ENSCJAG00000005731	IRF2
ENSCJAT00000011083	ENSCJAG00000005711	ENPP6
ENSCJAT00000010572	ENSCJAG00000005476	AGA
ENSCJAT00000010530	ENSCJAG00000005434	VEGFC
ENSCJAT00000010508	ENSCJAG00000005434	VEGFC
ENSCJAT00000010458	ENSCJAG00000005400	ASB5
ENSCJAT00000010373	ENSCJAG00000005304	WDR17
ENSCJAT00000058658	ENSCJAG00000005263	ADAM29
ENSCJAT00000054827	ENSCJAG00000005263	ADAM29
ENSCJAT00000010174	ENSCJAG00000005263	ADAM29
ENSCJAT00000010121	ENSCJAG00000005216	HPGD
ENSCJAT00000010114	ENSCJAG00000005216	HPGD
ENSCJAT00000052812	ENSCJAG00000005216	HPGD
ENSCJAT00000010120	ENSCJAG00000005216	HPGD
ENSCJAT00000010093	ENSCJAG00000005216	HPGD
ENSCJAT00000009986	ENSCJAG00000005158	FBXO8
ENSCJAT00000010012	ENSCJAG00000005158	FBXO8
ENSCJAT00000010005	ENSCJAG00000005158	FBXO8
ENSCJAT00000009769	ENSCJAG00000005048	AADAT
ENSCJAT00000018130	ENSCJAG00000005048	AADAT
ENSCJAT00000008995	ENSCJAG00000004671	SPOCK3
ENSCJAT00000002704	ENSCJAG00000001437	PPID
ENSCJAT00000003148	ENSCJAG00000001666	CTSO
ENSCJAT00000003354	ENSCJAG00000001768	RBM46
ENSCJAT00000060170	ENSCJAG00000001768	RBM46
ENSCJAT00000003353	ENSCJAG00000001768	RBM46
ENSCJAT00000004180	ENSCJAG00000002204	PRSS48
ENSCJAT00000004186	ENSCJAG00000002204	PRSS48
ENSCJAT00000004681	ENSCJAG00000002424	EDNRA

ENSCJAT00000004641	ENSCJAG00000002424	EDNRA
ENSCJAT00000004673	ENSCJAG00000002424	EDNRA
ENSCJAT00000004674	ENSCJAG00000002424	EDNRA
ENSCJAT00000004819	ENSCJAG00000002484	ZNF827
ENSCJAT00000004834	ENSCJAG00000002521	C4orf51
ENSCJAT00000004893	ENSCJAG00000002542	SMAD1
ENSCJAT00000005197	ENSCJAG00000002707	GYPA
ENSCJAT00000005408	ENSCJAG00000002803	GAB1
ENSCJAT00000005387	ENSCJAG00000002803	GAB1
ENSCJAT00000005419	ENSCJAG00000002831	USP38
ENSCJAT00000005428	ENSCJAG00000002831	USP38
ENSCJAT00000056630	ENSCJAG00000003045	CLGN
ENSCJAT00000005846	ENSCJAG00000003045	CLGN
ENSCJAT00000005836	ENSCJAG00000003045	CLGN
ENSCJAT00000005841	ENSCJAG00000003045	CLGN
ENSCJAT00000059986	ENSCJAG00000003045	CLGN
ENSCJAT00000005886	ENSCJAG00000003073	MGST2
ENSCJAT00000006116	ENSCJAG00000003174	ELF2
ENSCJAT00000056386	ENSCJAG00000003174	ELF2
ENSCJAT00000006175	ENSCJAG00000003185	SLC7A11
ENSCJAT00000023828	ENSCJAG00000003185	SLC7A11
ENSCJAT00000006470	ENSCJAG00000003356	C4orf29
ENSCJAT00000006847	ENSCJAG00000003565	FAT4
ENSCJAT00000006849	ENSCJAG00000003579	unknown
ENSCJAT00000058619	ENSCJAG00000003581	ANKRD50
ENSCJAT00000006855	ENSCJAG00000003581	ANKRD50
ENSCJAT00000005252	ENSCJAG00000002744	FGF2
ENSCJAT00000052232	ENSCJAG00000002804	KIAA1109
ENSCJAT00000005985	ENSCJAG00000003110	TNIP3
ENSCJAT00000005972	ENSCJAG00000003110	TNIP3
ENSCJAT00000005998	ENSCJAG00000003128	NDNF
ENSCJAT00000006191	ENSCJAG00000003176	PDE5A
ENSCJAT00000054010	ENSCJAG00000003176	PDE5A
ENSCJAT00000006176	ENSCJAG00000003176	PDE5A
ENSCJAT00000006187	ENSCJAG00000003176	PDE5A
ENSCJAT00000006261	ENSCJAG00000003260	MYOZ2
ENSCJAT00000006476	ENSCJAG00000003361	METTL14
ENSCJAT00000057872	ENSCJAG00000003390	NDST3
ENSCJAT00000006661	ENSCJAG00000003475	ARSJ
ENSCJAT00000006655	ENSCJAG00000003475	ARSJ
ENSCJAT00000058159	ENSCJAG00000003856	ENPEP
ENSCJAT00000007591	ENSCJAG00000003856	ENPEP
ENSCJAT00000007764	ENSCJAG00000004041	GAR1
ENSCJAT00000031409	ENSCJAG00000016082	SEC24B
ENSCJAT00000031379	ENSCJAG00000016082	SEC24B
ENSCJAT00000031401	ENSCJAG00000016082	SEC24B
ENSCJAT00000010939	ENSCJAG00000016082	SEC24B
ENSCJAT00000053106	ENSCJAG00000016082	SEC24B
ENSCJAT00000030927	ENSCJAG00000015903	DKK2
ENSCJAT00000030634	ENSCJAG00000015747	unknown
ENSCJAT00000030630	ENSCJAG00000015747	unknown
ENSCJAT00000054591	ENSCJAG00000015737	TET2
ENSCJAT00000030474	ENSCJAG00000015594	CENPE
ENSCJAT00000029905	ENSCJAG00000015331	SLC39A8
ENSCJAT00000029878	ENSCJAG00000015331	SLC39A8
ENSCJAT00000029926	ENSCJAG00000015331	SLC39A8
ENSCJAT00000029676	ENSCJAG00000015235	unknown
ENSCJAT00000014046	ENSCJAG00000007151	EIF4E
ENSCJAT00000014030	ENSCJAG00000007151	EIF4E

ENSCJAT00000014044	ENSCJAG00000007151	EIF4E
ENSCJAT00000061027	ENSCJAG00000006866	HPGDS
ENSCJAT00000013386	ENSCJAG00000006778	SMARCAD1
ENSCJAT00000013392	ENSCJAG00000006778	SMARCAD1
ENSCJAT00000060638	ENSCJAG00000006778	SMARCAD1
ENSCJAT00000013325	ENSCJAG00000006778	SMARCAD1
ENSCJAT00000012670	ENSCJAG00000006474	HERC5
ENSCJAT00000054805	ENSCJAG00000006474	HERC5
ENSCJAT00000012564	ENSCJAG00000006474	HERC5
ENSCJAT00000012252	ENSCJAG00000006299	SPP1
ENSCJAT00000012275	ENSCJAG00000006299	SPP1
ENSCJAT00000012256	ENSCJAG00000006299	SPP1
ENSCJAT00000057665	ENSCJAG00000006192	SPARCL1
ENSCJAT00000012040	ENSCJAG00000006192	SPARCL1
ENSCJAT00000011930	ENSCJAG00000006150	HSD17B11
ENSCJAT00000011913	ENSCJAG00000006120	HSD17B13
ENSCJAT00000011580	ENSCJAG00000005723	PTPN13
ENSCJAT00000011598	ENSCJAG00000005723	PTPN13
ENSCJAT00000057139	ENSCJAG00000005110	LIN54
ENSCJAT00000009915	ENSCJAG00000005110	LIN54
ENSCJAT00000009955	ENSCJAG00000005110	LIN54
ENSCJAT00000009932	ENSCJAG00000005110	LIN54
ENSCJAT00000059917	ENSCJAG00000015156	NCAPG2
ENSCJAT00000029522	ENSCJAG00000015156	NCAPG2
ENSCJAT00000029511	ENSCJAG00000015154	unknown
ENSCJAT00000029321	ENSCJAG00000015024	ANTXR2
ENSCJAT00000044095	ENSCJAG00000023110	NAA11
ENSCJAT00000029246	ENSCJAG00000015010	PAQR3
ENSCJAT00000029213	ENSCJAG00000014993	BMP2K
ENSCJAT00000029203	ENSCJAG00000014993	BMP2K
ENSCJAT00000028537	ENSCJAG00000014647	CCDC158
ENSCJAT00000027971	ENSCJAG00000014374	NAAA
ENSCJAT00000039056	ENSCJAG00000019852	USO1
ENSCJAT00000039065	ENSCJAG00000019852	USO1
ENSCJAT00000039170	ENSCJAG00000019942	THAP6
ENSCJAT00000039175	ENSCJAG00000019942	THAP6
ENSCJAT00000027162	ENSCJAG00000013971	AFP
ENSCJAT00000027252	ENSCJAG00000013986	ALB
ENSCJAT00000061184	ENSCJAG00000014529	RUFY3
ENSCJAT00000027789	ENSCJAG00000014305	SMR3A
ENSCJAT00000027725	ENSCJAG00000014268	unknown
ENSCJAT00000027714	ENSCJAG00000014257	unknown
ENSCJAT00000027706	ENSCJAG00000014257	unknown
ENSCJAT00000027675	ENSCJAG00000014226	SULT1E1
ENSCJAT00000026946	ENSCJAG00000013877	unknown
ENSCJAT00000055794	ENSCJAG00000009860	TMPRSS11A
ENSCJAT00000019155	ENSCJAG00000009860	TMPRSS11A
ENSCJAT00000019147	ENSCJAG00000009860	TMPRSS11A
ENSCJAT00000038244	ENSCJAG00000035154	unknown
ENSCJAT00000007900	ENSCJAG00000004102	NOA1
ENSCJAT00000007877	ENSCJAG00000004102	NOA1
ENSCJAT00000010577	ENSCJAG00000034129	unknown
ENSCJAT00000024776	ENSCJAG00000012772	HOPX
ENSCJAT00000035227	ENSCJAG00000018022	PAICS
ENSCJAT00000035212	ENSCJAG00000018022	PAICS
ENSCJAT00000035225	ENSCJAG00000018022	PAICS
ENSCJAT00000035495	ENSCJAG00000018119	CEP135
ENSCJAT00000035848	ENSCJAG00000018261	CLOCK
ENSCJAT00000035855	ENSCJAG00000018261	CLOCK

ENSCJAT00000035987	ENSCJAG00000018352	KDR
ENSCJAT00000037012	ENSCJAG00000018872	TXK
ENSCJAT00000037125	ENSCJAG00000018893	NFXL1
ENSCJAT00000037122	ENSCJAG00000018893	NFXL1
ENSCJAT00000037097	ENSCJAG00000018893	NFXL1
ENSCJAT00000037214	ENSCJAG00000018967	COMMD8
ENSCJAT00000037311	ENSCJAG00000018998	GABRA2
ENSCJAT00000037305	ENSCJAG00000018998	GABRA2
ENSCJAT00000037302	ENSCJAG00000018998	GABRA2
ENSCJAT00000037410	ENSCJAG00000019056	GUF1
ENSCJAT00000037400	ENSCJAG00000019056	GUF1
ENSCJAT00000001492	ENSCJAG00000000820	FAM114A1
ENSCJAT00000041100	ENSCJAG00000020913	KLF3
ENSCJAT00000041107	ENSCJAG00000020913	KLF3
ENSCJAT00000057004	ENSCJAG00000020921	TBC1D1
ENSCJAT00000041239	ENSCJAG00000020975	ARAP2
ENSCJAT00000060962	ENSCJAG00000021059	SMIM20
ENSCJAT00000041400	ENSCJAG00000021059	SMIM20
ENSCJAT00000060799	ENSCJAG00000021059	SMIM20
ENSCJAT00000041418	ENSCJAG00000021061	SEL1L3
ENSCJAT00000062419	ENSCJAG00000013366	SLC34A2
ENSCJAT00000026022	ENSCJAG00000013366	SLC34A2
ENSCJAT00000026013	ENSCJAG00000013366	SLC34A2
ENSCJAT00000025761	ENSCJAG00000013241	ZCCHC4
ENSCJAT00000058464	ENSCJAG00000013241	ZCCHC4
ENSCJAT00000025723	ENSCJAG00000013241	ZCCHC4
ENSCJAT00000025707	ENSCJAG00000013218	PI4K2B
ENSCJAT00000055638	ENSCJAG00000013178	SEPSECS
ENSCJAT00000025658	ENSCJAG00000013178	SEPSECS
ENSCJAT00000025651	ENSCJAG00000013178	SEPSECS
ENSCJAT00000025578	ENSCJAG00000013142	LGI2
ENSCJAT00000025515	ENSCJAG00000013142	LGI2
ENSCJAT00000025512	ENSCJAG00000013127	CCDC149
ENSCJAT00000057830	ENSCJAG00000013127	CCDC149
ENSCJAT00000025507	ENSCJAG00000013127	CCDC149
ENSCJAT00000025468	ENSCJAG00000013069	DHX15
ENSCJAT00000025472	ENSCJAG00000013069	DHX15
ENSCJAT00000059296	ENSCJAG00000013069	DHX15
ENSCJAT00000024326	ENSCJAG00000012541	TAPT1
ENSCJAT00000024192	ENSCJAG00000012476	BST1
ENSCJAT00000024202	ENSCJAG00000012476	BST1
ENSCJAT00000024151	ENSCJAG00000012393	FBXL5
ENSCJAT00000063661	ENSCJAG00000012263	C1QTNF7
ENSCJAT00000023772	ENSCJAG00000012263	C1QTNF7
ENSCJAT00000063799	ENSCJAG00000012119	HS3ST1
ENSCJAT00000023515	ENSCJAG00000012119	HS3ST1
ENSCJAT00000022052	ENSCJAG00000011293	WDR1
ENSCJAT00000022084	ENSCJAG00000011293	WDR1
ENSCJAT00000022074	ENSCJAG00000011293	WDR1
ENSCJAT00000000189	ENSCJAG00000000107	OTOP1
ENSCJAT00000000192	ENSCJAG00000000107	OTOP1
ENSCJAT00000020778	ENSCJAG00000010666	C4orf50
ENSCJAT00000001860	ENSCJAG00000001022	SORCS2
ENSCJAT00000001844	ENSCJAG00000001022	SORCS2
ENSCJAT00000060080	ENSCJAG00000014004	DOK7
ENSCJAT00000028162	ENSCJAG00000014447	POLN
ENSCJAT00000018332	ENSCJAG00000009461	UVSSA
ENSCJAT00000024154	ENSCJAG00000012473	unknown
ENSCJAT00000003764	ENSCJAG00000001975	MFSD7

ENSCJAT00000059977	ENSCJAG00000001984	MYL5
ENSCJAT00000036474	ENSCJAG00000018596	GPX6
ENSCJAT00000036471	ENSCJAG00000018596	GPX6
ENSCJAT00000036639	ENSCJAG00000018671	unknown
ENSCJAT00000036668	ENSCJAG00000036509	unknown
ENSCJAT00000021877	ENSCJAG00000011235	ZNF184
ENSCJAT00000021846	ENSCJAG00000011186	PRSS16
ENSCJAT00000021829	ENSCJAG00000011186	PRSS16
ENSCJAT00000021834	ENSCJAG00000011186	PRSS16
ENSCJAT00000021759	ENSCJAG00000011175	ZNF322
ENSCJAT00000032984	ENSCJAG00000011175	ZNF322
ENSCJAT00000058552	ENSCJAG00000011068	unknown
ENSCJAT00000021661	ENSCJAG00000011068	unknown
ENSCJAT00000021708	ENSCJAG00000011068	unknown
ENSCJAT00000063726	ENSCJAG00000011068	unknown
ENSCJAT00000021524	ENSCJAG00000011048	unknown
ENSCJAT00000021466	ENSCJAG00000022177	HIST1H2BI
ENSCJAT00000021506	ENSCJAG00000011039	unknown
ENSCJAT00000043416	ENSCJAG00000011039	unknown
ENSCJAT00000021418	ENSCJAG00000011039	unknown
ENSCJAT00000011923	ENSCJAG00000006136	C6orf62
ENSCJAT00000011932	ENSCJAG00000006136	C6orf62
ENSCJAT00000011882	ENSCJAG00000006130	ACOT13
ENSCJAT00000011832	ENSCJAG00000006084	KIAA0319
ENSCJAT00000011603	ENSCJAG00000005978	NRSN1
ENSCJAT00000014528	ENSCJAG00000007416	SOX4
ENSCJAT00000013591	ENSCJAG00000006964	RNF144B
ENSCJAT00000013580	ENSCJAG00000006952	DEK
ENSCJAT00000061401	ENSCJAG00000006952	DEK
ENSCJAT00000013549	ENSCJAG00000006934	KDM1B
ENSCJAT00000013144	ENSCJAG00000006685	NUP153
ENSCJAT00000038888	ENSCJAG00000019809	GFOD1
ENSCJAT00000041916	ENSCJAG00000021312	TFAP2A
ENSCJAT00000053491	ENSCJAG00000021312	TFAP2A
ENSCJAT00000041948	ENSCJAG00000021330	SNRNP48
ENSCJAT00000041983	ENSCJAG00000021351	CAGE1
ENSCJAT00000041985	ENSCJAG00000021351	CAGE1
ENSCJAT00000041986	ENSCJAG00000021351	CAGE1
ENSCJAT00000041993	ENSCJAG00000021354	SSR1
ENSCJAT00000041994	ENSCJAG00000021354	SSR1
ENSCJAT00000057236	ENSCJAG00000021354	SSR1
ENSCJAT00000053876	ENSCJAG00000021354	SSR1
ENSCJAT00000059892	ENSCJAG00000021354	SSR1
ENSCJAT00000042002	ENSCJAG00000021358	RREB1
ENSCJAT00000041999	ENSCJAG00000021358	RREB1
ENSCJAT00000042004	ENSCJAG00000021358	RREB1
ENSCJAT00000011153	ENSCJAG00000035473	unknown
ENSCJAT00000034335	ENSCJAG00000017623	PSMG4
ENSCJAT00000053700	ENSCJAG00000017623	PSMG4
ENSCJAT00000060147	ENSCJAG00000017623	PSMG4
ENSCJAT00000034328	ENSCJAG00000017623	PSMG4
ENSCJAT00000034526	ENSCJAG00000017717	NQO2
ENSCJAT00000038403	ENSCJAG00000019533	EXOC2
ENSCJAT00000063125	ENSCJAG00000019533	EXOC2
ENSCJAT00000057234	ENSCJAG00000019533	EXOC2
ENSCJAT00000038392	ENSCJAG00000019533	EXOC2
ENSCJAT00000038300	ENSCJAG00000019524	IRF4
ENSCJAT00000038302	ENSCJAG00000019524	IRF4
ENSCJAT00000049638	ENSCJAG00000019949	MRPS18B

ENSCJAT00000039185	ENSCJAG00000019949	MRPS18B
ENSCJAT00000063069	ENSCJAG00000019874	FLOT1
ENSCJAT00000056090	ENSCJAG00000019874	FLOT1
ENSCJAT00000039066	ENSCJAG00000019874	FLOT1
ENSCJAT00000039071	ENSCJAG00000019874	FLOT1
ENSCJAT00000038999	ENSCJAG00000019860	DDR1
ENSCJAT00000039005	ENSCJAG00000019860	DDR1
ENSCJAT00000038887	ENSCJAG00000019806	unknown
ENSCJAT00000006188	ENSCJAG00000003152	DDX39B
ENSCJAT00000060333	ENSCJAG00000003129	NFKBIL1
ENSCJAT00000006036	ENSCJAG00000003129	NFKBIL1
ENSCJAT00000004670	ENSCJAG00000002416	unknown
ENSCJAT00000060452	ENSCJAG00000002416	unknown
ENSCJAT00000060603	ENSCJAG00000002416	unknown
ENSCJAT00000004490	ENSCJAG00000002263	SKIV2L
ENSCJAT00000063763	ENSCJAG00000002263	SKIV2L
ENSCJAT00000004479	ENSCJAG00000002263	SKIV2L
ENSCJAT00000054662	ENSCJAG00000002263	SKIV2L
ENSCJAT00000062577	ENSCJAG00000002263	SKIV2L
ENSCJAT00000004205	ENSCJAG00000002138	unknown
ENSCJAT00000004189	ENSCJAG00000002138	unknown
ENSCJAT00000004200	ENSCJAG00000002138	unknown
ENSCJAT00000004217	ENSCJAG00000002138	unknown
ENSCJAT00000004156	ENSCJAG00000002138	unknown
ENSCJAT00000003549	ENSCJAG00000001854	AGPAT1
ENSCJAT00000003557	ENSCJAG00000001854	AGPAT1
ENSCJAT00000003721	ENSCJAG00000001948	unknown
ENSCJAT00000003740	ENSCJAG00000001948	unknown
ENSCJAT00000042381	ENSCJAG00000001629	CAJA-DQA1
ENSCJAT00000042383	ENSCJAG00000021551	PSMB8
ENSCJAT00000042384	ENSCJAG00000021551	PSMB8
ENSCJAT00000042649	ENSCJAG00000021681	KIFC1
ENSCJAT00000063005	ENSCJAG00000021681	KIFC1
ENSCJAT00000042665	ENSCJAG00000021691	SYNGAP1
ENSCJAT00000042664	ENSCJAG00000021691	SYNGAP1
ENSCJAT00000042663	ENSCJAG00000021691	SYNGAP1
ENSCJAT00000023655	ENSCJAG00000012184	IP6K3
ENSCJAT00000023641	ENSCJAG00000012184	IP6K3
ENSCJAT00000063420	ENSCJAG00000012184	IP6K3
ENSCJAT00000053054	ENSCJAG00000012184	IP6K3
ENSCJAT00000052212	ENSCJAG00000015226	unknown
ENSCJAT00000029766	ENSCJAG00000015294	SPDEF
ENSCJAT00000059665	ENSCJAG00000015294	SPDEF
ENSCJAT00000029789	ENSCJAG00000015304	C6orf106
ENSCJAT00000030280	ENSCJAG00000015519	ZNF76
ENSCJAT00000030288	ENSCJAG00000015519	ZNF76
ENSCJAT00000030299	ENSCJAG00000015519	ZNF76
ENSCJAT00000030298	ENSCJAG00000015519	ZNF76
ENSCJAT00000030361	ENSCJAG00000015599	unknown
ENSCJAT00000063054	ENSCJAG00000015604	PPARD
ENSCJAT00000030390	ENSCJAG00000015604	PPARD
ENSCJAT00000030550	ENSCJAG00000015661	TEAD3
ENSCJAT00000030531	ENSCJAG00000015661	TEAD3
ENSCJAT00000030576	ENSCJAG00000015713	TULP1
ENSCJAT00000061031	ENSCJAG00000015713	TULP1
ENSCJAT00000059546	ENSCJAG00000015713	TULP1
ENSCJAT00000031131	ENSCJAG00000016001	C6orf222
ENSCJAT00000031130	ENSCJAG00000016001	C6orf222
ENSCJAT00000062606	ENSCJAG00000016033	KCTD20

ENSCJAT00000054187	ENSCJAG00000016033	KCTD20
ENSCJAT00000031188	ENSCJAG00000016033	KCTD20
ENSCJAT00000030770	ENSCJAG00000016033	KCTD20
ENSCJAT00000031182	ENSCJAG00000016033	KCTD20
ENSCJAT00000031231	ENSCJAG00000016062	unknown
ENSCJAT00000031241	ENSCJAG00000016062	unknown
ENSCJAT00000063075	ENSCJAG00000016062	unknown
ENSCJAT00000010177	ENSCJAG00000005267	unknown
ENSCJAT00000010854	ENSCJAG00000005567	CMTR1
ENSCJAT00000057370	ENSCJAG00000005567	CMTR1
ENSCJAT00000063055	ENSCJAG00000005701	unknown
ENSCJAT00000011065	ENSCJAG00000005701	unknown
ENSCJAT00000020371	ENSCJAG00000010452	DAAM2
ENSCJAT00000061869	ENSCJAG00000004246	unknown
ENSCJAT00000008130	ENSCJAG00000036721	unknown
ENSCJAT00000008781	ENSCJAG00000004580	PRPH2
ENSCJAT00000054225	ENSCJAG00000004594	GLTSCR1L
ENSCJAT00000008935	ENSCJAG00000004662	CNPY3
ENSCJAT00000008975	ENSCJAG00000004675	GNMT
ENSCJAT00000009194	ENSCJAG00000004684	PEX6
ENSCJAT00000009202	ENSCJAG00000004684	PEX6
ENSCJAT00000009138	ENSCJAG00000004684	PEX6
ENSCJAT00000009518	ENSCJAG00000004911	KLHDC3
ENSCJAT00000009512	ENSCJAG00000004911	KLHDC3
ENSCJAT00000010303	ENSCJAG00000005337	DNPH1
ENSCJAT00000010300	ENSCJAG00000005337	DNPH1
ENSCJAT00000010475	ENSCJAG00000005420	ZNF318
ENSCJAT00000010469	ENSCJAG00000005420	ZNF318
ENSCJAT00000010818	ENSCJAG00000005594	POLR1C
ENSCJAT00000011238	ENSCJAG00000005770	POLH
ENSCJAT00000011233	ENSCJAG00000005770	POLH
ENSCJAT00000023551	ENSCJAG00000012123	CLIC5
ENSCJAT00000023353	ENSCJAG00000012035	TDRD6
ENSCJAT00000023356	ENSCJAG00000012035	TDRD6
ENSCJAT00000054970	ENSCJAG00000012000	MEP1A
ENSCJAT00000023309	ENSCJAG00000012000	MEP1A
ENSCJAT00000023313	ENSCJAG00000012000	MEP1A
ENSCJAT00000023003	ENSCJAG00000011845	GPR115
ENSCJAT00000022607	ENSCJAG00000011651	CRISP2
ENSCJAT00000022672	ENSCJAG00000011651	CRISP2
ENSCJAT00000063577	ENSCJAG00000011651	CRISP2
ENSCJAT00000054673	ENSCJAG00000011651	CRISP2
ENSCJAT00000022475	ENSCJAG00000011561	TFAP2B
ENSCJAT00000022470	ENSCJAG00000011561	TFAP2B
ENSCJAT00000061998	ENSCJAG00000034823	unknown
ENSCJAT00000021948	ENSCJAG00000011268	GSTA4
ENSCJAT00000056189	ENSCJAG00000011268	GSTA4
ENSCJAT00000054063	ENSCJAG00000010723	COL21A1
ENSCJAT00000020771	ENSCJAG00000010349	DST
ENSCJAT00000003330	ENSCJAG00000001759	LGSN
ENSCJAT00000019466	ENSCJAG00000010003	MTO1
ENSCJAT00000020127	ENSCJAG00000010325	TMEM30A
ENSCJAT00000020121	ENSCJAG00000010325	TMEM30A
ENSCJAT00000020806	ENSCJAG00000010684	IRAK1BP1
ENSCJAT00000018164	ENSCJAG00000009155	DOPEY1
ENSCJAT00000018134	ENSCJAG00000009155	DOPEY1
ENSCJAT00000018015	ENSCJAG00000009155	DOPEY1
ENSCJAT00000004515	ENSCJAG00000015128	SNAP91
ENSCJAT00000029557	ENSCJAG00000015171	MRAP2

ENSCJAT00000001179	ENSCJAG00000000648	unknown
ENSCJAT00000001176	ENSCJAG00000000648	unknown
ENSCJAT00000001184	ENSCJAG00000000651	C6orf163
ENSCJAT00000001209	ENSCJAG00000036299	SLC35A1
ENSCJAT00000001195	ENSCJAG00000036299	SLC35A1
ENSCJAT00000065030	ENSCJAG00000036299	SLC35A1
ENSCJAT00000065522	ENSCJAG00000036299	SLC35A1
ENSCJAT00000065396	ENSCJAG00000036299	SLC35A1
ENSCJAT00000001206	ENSCJAG00000036299	SLC35A1
ENSCJAT00000001317	ENSCJAG00000000692	RARS2
ENSCJAT00000001405	ENSCJAG00000000775	CNR1
ENSCJAT00000001397	ENSCJAG00000000775	CNR1
ENSCJAT00000001964	ENSCJAG00000001026	MAP3K7
ENSCJAT00000001968	ENSCJAG00000001026	MAP3K7
ENSCJAT00000001951	ENSCJAG00000001026	MAP3K7
ENSCJAT00000002066	ENSCJAG00000001086	EPHA7
ENSCJAT00000063778	ENSCJAG00000001179	UFL1
ENSCJAT00000002422	ENSCJAG00000001288	FBXL4
ENSCJAT00000059721	ENSCJAG00000001288	FBXL4
ENSCJAT00000002461	ENSCJAG00000001326	COQ3
ENSCJAT00000002452	ENSCJAG00000001326	COQ3
ENSCJAT00000002536	ENSCJAG00000001356	USP45
ENSCJAT00000002766	ENSCJAG00000001421	PRDM13
ENSCJAT00000002689	ENSCJAG00000001421	PRDM13
ENSCJAT00000003221	ENSCJAG00000001700	POPDC3
ENSCJAT00000012353	ENSCJAG00000006332	PRDM1
ENSCJAT00000054915	ENSCJAG00000006332	PRDM1
ENSCJAT00000012341	ENSCJAG00000006332	PRDM1
ENSCJAT00000012226	ENSCJAG00000006246	AIM1
ENSCJAT00000012211	ENSCJAG00000006246	AIM1
ENSCJAT00000012047	ENSCJAG00000006200	QRSL1
ENSCJAT00000012044	ENSCJAG00000006200	QRSL1
ENSCJAT00000011533	ENSCJAG00000005929	FOXO3
ENSCJAT00000062128	ENSCJAG00000005929	FOXO3
ENSCJAT00000026545	ENSCJAG00000005929	FOXO3
ENSCJAT00000057908	ENSCJAG00000005929	FOXO3
ENSCJAT00000011396	ENSCJAG00000005825	CEP57L1
ENSCJAT00000011331	ENSCJAG00000005825	CEP57L1
ENSCJAT00000010147	ENSCJAG00000005248	GTF3C6
ENSCJAT00000009689	ENSCJAG00000004951	TRAF3IP2
ENSCJAT00000008825	ENSCJAG00000004595	FRK
ENSCJAT00000008657	ENSCJAG00000004524	FAM26F
ENSCJAT00000008667	ENSCJAG00000004524	FAM26F
ENSCJAT00000008647	ENSCJAG00000004513	TRAPPC3L
ENSCJAT00000056112	ENSCJAG00000004474	unknown
ENSCJAT00000008586	ENSCJAG00000004474	unknown
ENSCJAT00000008599	ENSCJAG00000004474	unknown
ENSCJAT00000008610	ENSCJAG00000004474	unknown
ENSCJAT00000063051	ENSCJAG00000004474	unknown
ENSCJAT00000008475	ENSCJAG00000004394	KPNA5
ENSCJAT00000008189	ENSCJAG00000004257	DCBLD1
ENSCJAT00000055896	ENSCJAG00000004242	GOPC
ENSCJAT00000008137	ENSCJAG00000004242	GOPC
ENSCJAT00000008136	ENSCJAG00000004242	GOPC
ENSCJAT00000007747	ENSCJAG00000004029	TBC1D32
ENSCJAT00000007757	ENSCJAG00000004029	TBC1D32
ENSCJAT00000007725	ENSCJAG00000004018	GJA1
ENSCJAT00000007720	ENSCJAG00000004018	GJA1
ENSCJAT00000007707	ENSCJAG00000003956	HSF2

ENSCJAT00000007701	ENSCJAG00000003956	HSF2
ENSCJAT00000007544	ENSCJAG00000003930	PKIB
ENSCJAT00000057645	ENSCJAG00000003930	PKIB
ENSCJAT00000007469	ENSCJAG00000003876	CLVS2
ENSCJAT00000007341	ENSCJAG00000003808	RNF217
ENSCJAT00000062850	ENSCJAG00000003808	RNF217
ENSCJAT00000007332	ENSCJAG00000003808	RNF217
ENSCJAT00000007315	ENSCJAG00000003808	RNF217
ENSCJAT00000053990	ENSCJAG00000003686	TRMT11
ENSCJAT00000007098	ENSCJAG00000003686	TRMT11
ENSCJAT00000007028	ENSCJAG00000003669	RSPO3
ENSCJAT00000007034	ENSCJAG00000003669	RSPO3
ENSCJAT00000006989	ENSCJAG00000003649	ECHDC1
ENSCJAT00000006992	ENSCJAG00000003649	ECHDC1
ENSCJAT00000006931	ENSCJAG00000003616	unknown
ENSCJAT00000057841	ENSCJAG00000003496	PTPRK
ENSCJAT00000006111	ENSCJAG00000003171	EPB41L2
ENSCJAT00000006104	ENSCJAG00000003171	EPB41L2
ENSCJAT00000005820	ENSCJAG00000003010	ENPP1
ENSCJAT00000005595	ENSCJAG00000002925	TAAR8
ENSCJAT00000005592	ENSCJAG00000002925	TAAR8
ENSCJAT00000005563	ENSCJAG00000002912	TAAR2
ENSCJAT00000053863	ENSCJAG00000036205	VNN1
ENSCJAT00000062872	ENSCJAG00000002893	VNN3
ENSCJAT00000005550	ENSCJAG00000002893	VNN3
ENSCJAT00000005556	ENSCJAG00000002893	VNN3
ENSCJAT00000053908	ENSCJAG00000002893	VNN3
ENSCJAT00000015686	ENSCJAG00000007798	MYB
ENSCJAT00000062451	ENSCJAG00000007798	MYB
ENSCJAT00000054125	ENSCJAG00000007798	MYB
ENSCJAT00000055730	ENSCJAG00000007798	MYB
ENSCJAT00000061880	ENSCJAG00000007798	MYB
ENSCJAT00000062789	ENSCJAG00000007798	MYB
ENSCJAT00000015975	ENSCJAG00000008189	MTFR2
ENSCJAT00000016531	ENSCJAG00000008383	MAP3K5
ENSCJAT00000060250	ENSCJAG00000033044	unknown
ENSCJAT00000053201	ENSCJAG00000033044	unknown
ENSCJAT00000037351	ENSCJAG00000019042	NHSL1
ENSCJAT00000037352	ENSCJAG00000019042	NHSL1
ENSCJAT00000037347	ENSCJAG00000019042	NHSL1
ENSCJAT00000037417	ENSCJAG00000019079	ECT2L
ENSCJAT00000037412	ENSCJAG00000019079	ECT2L
ENSCJAT00000037723	ENSCJAG00000019230	PEX3
ENSCJAT00000017833	ENSCJAG00000009197	LATS1
ENSCJAT00000063441	ENSCJAG00000009197	LATS1
ENSCJAT00000057926	ENSCJAG00000012842	SYNE1
ENSCJAT00000025054	ENSCJAG00000012842	SYNE1
ENSCJAT00000024874	ENSCJAG00000012833	MYCT1
ENSCJAT00000024867	ENSCJAG00000012833	MYCT1
ENSCJAT00000024816	ENSCJAG00000012799	FBXO5
ENSCJAT00000024833	ENSCJAG00000012799	FBXO5
ENSCJAT00000056574	ENSCJAG00000012787	MTRF1L
ENSCJAT00000059158	ENSCJAG00000012787	MTRF1L
ENSCJAT00000024798	ENSCJAG00000012787	MTRF1L
ENSCJAT00000024759	ENSCJAG00000012747	OPRM1
ENSCJAT00000024656	ENSCJAG00000012720	unknown
ENSCJAT00000024304	ENSCJAG00000012540	TFB1M
ENSCJAT00000024274	ENSCJAG00000012516	NOX3
ENSCJAT00000061522	ENSCJAG00000012516	NOX3

ENSCJAT00000006974	ENSCJAG00000003638	GTF2H5
ENSCJAT00000007094	ENSCJAG00000034779	unknown
ENSCJAT00000022540	ENSCJAG00000011616	TAGAP
ENSCJAT00000023921	ENSCJAG00000012323	AGPAT4
ENSCJAT00000057567	ENSCJAG00000012323	AGPAT4
ENSCJAT00000052550	ENSCJAG00000012323	AGPAT4
ENSCJAT00000061768	ENSCJAG00000012323	AGPAT4
ENSCJAT00000055696	ENSCJAG00000012323	AGPAT4
ENSCJAT00000023971	ENSCJAG00000012368	QKI
ENSCJAT00000024252	ENSCJAG00000012508	T
ENSCJAT00000024288	ENSCJAG00000012533	SFT2D1
ENSCJAT00000000850	ENSCJAG00000000469	unknown
ENSCJAT00000000852	ENSCJAG00000000469	unknown
ENSCJAT00000063003	ENSCJAG00000033004	KIF25
ENSCJAT00000058158	ENSCJAG00000003481	WDR27
ENSCJAT00000006645	ENSCJAG00000003436	PHF10
ENSCJAT00000002486	ENSCJAG00000001339	SLA2
ENSCJAT00000002490	ENSCJAG00000001339	SLA2
ENSCJAT00000034484	ENSCJAG00000017642	RBM12
ENSCJAT00000034490	ENSCJAG00000017642	RBM12
ENSCJAT00000034486	ENSCJAG00000017642	RBM12
ENSCJAT00000034493	ENSCJAG00000017642	RBM12
ENSCJAT00000034483	ENSCJAG00000017642	RBM12
ENSCJAT00000035528	ENSCJAG00000018152	PIGU
ENSCJAT00000035526	ENSCJAG00000018152	PIGU
ENSCJAT00000035509	ENSCJAG00000018152	PIGU
ENSCJAT00000035833	ENSCJAG00000018303	unknown
ENSCJAT00000036344	ENSCJAG00000018544	BPIFA2
ENSCJAT00000036426	ENSCJAG00000018580	BPIFB3
ENSCJAT00000055782	ENSCJAG00000018580	BPIFB3
ENSCJAT00000056000	ENSCJAG00000020772	DUSP15
ENSCJAT00000040816	ENSCJAG00000020772	DUSP15
ENSCJAT00000040814	ENSCJAG00000020772	DUSP15
ENSCJAT00000060855	ENSCJAG00000020772	DUSP15
ENSCJAT00000053047	ENSCJAG00000020772	DUSP15
ENSCJAT00000063838	ENSCJAG00000020772	DUSP15
ENSCJAT00000040818	ENSCJAG00000020773	MYLK2
ENSCJAT00000040821	ENSCJAG00000020773	MYLK2
ENSCJAT00000040836	ENSCJAG00000020774	TPX2
ENSCJAT00000040848	ENSCJAG00000020785	COX4I2
ENSCJAT00000040905	ENSCJAG00000020818	DEFB125
ENSCJAT00000040972	ENSCJAG00000020847	TBC1D20
ENSCJAT00000041023	ENSCJAG00000020873	SLC52A3
ENSCJAT00000041028	ENSCJAG00000020876	FAM110A
ENSCJAT00000041050	ENSCJAG00000020892	C20orf202
ENSCJAT00000041167	ENSCJAG00000020949	STK35
ENSCJAT00000041164	ENSCJAG00000020949	STK35
ENSCJAT00000041587	ENSCJAG00000021125	C20orf194
ENSCJAT00000041561	ENSCJAG00000021125	C20orf194
ENSCJAT00000041689	ENSCJAG00000021194	C20orf27
ENSCJAT00000041688	ENSCJAG00000021194	C20orf27
ENSCJAT00000041696	ENSCJAG00000021195	SPEF1
ENSCJAT00000041708	ENSCJAG00000021199	CDC25B
ENSCJAT00000041710	ENSCJAG00000021199	CDC25B
ENSCJAT00000052370	ENSCJAG00000021199	CDC25B
ENSCJAT00000041726	ENSCJAG00000021211	MAVS
ENSCJAT00000041730	ENSCJAG00000021211	MAVS
ENSCJAT00000041800	ENSCJAG00000021256	RASSF2
ENSCJAT00000041804	ENSCJAG00000021256	RASSF2

ENSCJAT00000041867	ENSCJAG00000021287	GPCPD1
ENSCJAT00000041864	ENSCJAG00000021287	GPCPD1
ENSCJAT00000041898	ENSCJAG00000021302	CRLS1
ENSCJAT00000041899	ENSCJAG00000021302	CRLS1
ENSCJAT00000041920	ENSCJAG00000021316	TMX4
ENSCJAT00000041998	ENSCJAG00000021357	JAG1
ENSCJAT00000041997	ENSCJAG00000021357	JAG1
ENSCJAT00000008806	ENSCJAG00000004583	ISM1
ENSCJAT00000009685	ENSCJAG00000004947	SNX5
ENSCJAT00000009692	ENSCJAG00000004947	SNX5
ENSCJAT00000009693	ENSCJAG00000004947	SNX5
ENSCJAT00000009929	ENSCJAG00000005123	DZANK1
ENSCJAT00000009934	ENSCJAG00000005123	DZANK1
ENSCJAT00000009940	ENSCJAG00000005123	DZANK1
ENSCJAT00000009923	ENSCJAG00000005123	DZANK1
ENSCJAT00000060073	ENSCJAG00000005393	CRNKL1
ENSCJAT00000010488	ENSCJAG00000005393	CRNKL1
ENSCJAT00000022832	ENSCJAG00000011698	PYGB
ENSCJAT00000042316	ENSCJAG00000021513	MANBAL
ENSCJAT00000042290	ENSCJAG00000021501	BPI
ENSCJAT00000034937	ENSCJAG00000017888	SRSF6
ENSCJAT00000034927	ENSCJAG00000017888	SRSF6
ENSCJAT00000034883	ENSCJAG00000017869	SGK2
ENSCJAT00000034867	ENSCJAG00000017869	SGK2
ENSCJAT00000034873	ENSCJAG00000017869	SGK2
ENSCJAT00000034644	ENSCJAG00000017746	HNF4A
ENSCJAT00000034573	ENSCJAG00000017736	TTPAL
ENSCJAT00000034557	ENSCJAG00000017722	SERINC3
ENSCJAT00000053625	ENSCJAG00000017722	SERINC3
ENSCJAT00000034550	ENSCJAG00000017722	SERINC3
ENSCJAT00000055495	ENSCJAG00000017722	SERINC3
ENSCJAT00000033863	ENSCJAG00000017392	unknown
ENSCJAT00000033864	ENSCJAG00000017392	unknown
ENSCJAT00000033842	ENSCJAG00000017387	WFDC9
ENSCJAT00000033637	ENSCJAG00000017262	PLTP
ENSCJAT00000033627	ENSCJAG00000017262	PLTP
ENSCJAT00000024621	ENSCJAG00000017149	SLC12A5
ENSCJAT00000033445	ENSCJAG00000017149	SLC12A5
ENSCJAT00000033345	ENSCJAG00000017149	SLC12A5
ENSCJAT00000033425	ENSCJAG00000017149	SLC12A5
ENSCJAT00000024504	ENSCJAG00000017149	SLC12A5
ENSCJAT00000032964	ENSCJAG00000016959	OCSTAMP
ENSCJAT00000032024	ENSCJAG00000016296	CSE1L
ENSCJAT00000031999	ENSCJAG00000016296	CSE1L
ENSCJAT00000032006	ENSCJAG00000016296	CSE1L
ENSCJAT00000031944	ENSCJAG00000016296	CSE1L
ENSCJAT00000031586	ENSCJAG00000016233	STAU1
ENSCJAT00000031684	ENSCJAG00000016233	STAU1
ENSCJAT00000031572	ENSCJAG00000016135	DDX27
ENSCJAT00000001027	ENSCJAG00000000559	PARD6B
ENSCJAT00000001029	ENSCJAG00000000559	PARD6B
ENSCJAT00000004987	ENSCJAG00000002600	DOK5
ENSCJAT00000021912	ENSCJAG00000011258	FAM210B
ENSCJAT00000037522	ENSCJAG00000019142	unknown
ENSCJAT00000037337	ENSCJAG00000019027	VAPB
ENSCJAT00000037093	ENSCJAG00000018918	CTSZ
ENSCJAT00000037096	ENSCJAG00000018918	CTSZ
ENSCJAT00000037076	ENSCJAG00000018914	TUBB1
ENSCJAT00000037043	ENSCJAG00000018885	EDN3

ENSCJAT00000037042	ENSCJAG00000018885	EDN3
ENSCJAT00000037010	ENSCJAG00000018836	SYCP2
ENSCJAT00000053021	ENSCJAG00000014438	TAF4
ENSCJAT00000057867	ENSCJAG00000014438	TAF4
ENSCJAT00000028082	ENSCJAG00000014438	TAF4
ENSCJAT00000027774	ENSCJAG00000014274	unknown
ENSCJAT00000027745	ENSCJAG00000014274	unknown
ENSCJAT00000027561	ENSCJAG00000014173	RBBP8NL
ENSCJAT00000027557	ENSCJAG00000014173	RBBP8NL
ENSCJAT00000027529	ENSCJAG00000014152	GATA5
ENSCJAT00000027513	ENSCJAG00000014152	GATA5
ENSCJAT00000019374	ENSCJAG00000009980	TCFL5
ENSCJAT00000019524	ENSCJAG00000010056	YTHDF1
ENSCJAT00000019514	ENSCJAG00000010056	YTHDF1
ENSCJAT00000061259	ENSCJAG00000010518	UCKL1
ENSCJAT00000028776	ENSCJAG00000014782	PCMTD2
ENSCJAT00000024122	ENSCJAG00000012412	SGSM2
ENSCJAT00000024099	ENSCJAG00000012412	SGSM2
ENSCJAT00000024113	ENSCJAG00000012412	SGSM2
ENSCJAT00000024157	ENSCJAG00000012456	TSR1
ENSCJAT00000063556	ENSCJAG00000001911	PRPF8
ENSCJAT00000013977	ENSCJAG00000007138	TAX1BP3
ENSCJAT00000014021	ENSCJAG00000007148	unknown
ENSCJAT00000022497	ENSCJAG00000011552	CAMKK1
ENSCJAT00000002456	ENSCJAG00000001307	SLC47A1
ENSCJAT00000002470	ENSCJAG00000001307	SLC47A1
ENSCJAT00000055423	ENSCJAG00000001307	SLC47A1
ENSCJAT00000002431	ENSCJAG00000001307	SLC47A1
ENSCJAT00000002468	ENSCJAG00000001307	SLC47A1
ENSCJAT00000002462	ENSCJAG00000001307	SLC47A1
ENSCJAT00000001764	ENSCJAG00000000974	ALKBH5
ENSCJAT00000001771	ENSCJAG00000000974	ALKBH5
ENSCJAT00000017536	ENSCJAG00000009042	LRRC48
ENSCJAT00000061803	ENSCJAG00000004522	COX10
ENSCJAT00000056048	ENSCJAG00000004522	COX10
ENSCJAT00000055425	ENSCJAG00000004522	COX10
ENSCJAT00000008675	ENSCJAG00000004522	COX10
ENSCJAT00000008811	ENSCJAG00000004542	ELAC2
ENSCJAT00000008777	ENSCJAG00000004542	ELAC2
ENSCJAT00000009114	ENSCJAG00000004683	MYOCD
ENSCJAT00000009119	ENSCJAG00000004683	MYOCD
ENSCJAT00000025527	ENSCJAG00000012860	MYH4
ENSCJAT00000025219	ENSCJAG00000012860	MYH4
ENSCJAT00000025539	ENSCJAG00000012860	MYH4
ENSCJAT00000025283	ENSCJAG00000012860	MYH4
ENSCJAT00000024893	ENSCJAG00000012860	MYH4
ENSCJAT00000025566	ENSCJAG00000012860	MYH4
ENSCJAT00000024718	ENSCJAG00000012758	RCVRN
ENSCJAT00000035704	ENSCJAG00000018264	SPDYE4
ENSCJAT00000028543	ENSCJAG00000014652	ALOX12B
ENSCJAT00000028538	ENSCJAG00000014652	ALOX12B
ENSCJAT00000028571	ENSCJAG00000014652	ALOX12B
ENSCJAT00000028634	ENSCJAG00000014688	GUCY2D
ENSCJAT00000028622	ENSCJAG00000014688	GUCY2D
ENSCJAT00000028728	ENSCJAG00000014760	KCNAB3
ENSCJAT00000028734	ENSCJAG00000014760	KCNAB3
ENSCJAT00000029610	ENSCJAG00000015157	POLR2A
ENSCJAT00000032961	ENSCJAG00000015157	POLR2A
ENSCJAT00000029601	ENSCJAG00000015157	POLR2A

ENSCJAT00000052904	ENSCJAG00000015157	POLR2A
ENSCJAT00000001362	ENSCJAG00000000751	ZBTB4
ENSCJAT00000001357	ENSCJAG00000000751	ZBTB4
ENSCJAT00000001359	ENSCJAG00000000751	ZBTB4
ENSCJAT00000031964	ENSCJAG00000016438	TNK1
ENSCJAT00000031970	ENSCJAG00000016438	TNK1
ENSCJAT00000032170	ENSCJAG00000016494	unknown
ENSCJAT00000032472	ENSCJAG00000016678	DVL2
ENSCJAT00000060152	ENSCJAG00000016759	DLG4
ENSCJAT00000032733	ENSCJAG00000016819	CLEC10A
ENSCJAT00000032777	ENSCJAG00000016819	CLEC10A
ENSCJAT00000032763	ENSCJAG00000016819	CLEC10A
ENSCJAT00000032755	ENSCJAG00000016819	CLEC10A
ENSCJAT00000032901	ENSCJAG00000016905	BCL6B
ENSCJAT00000032898	ENSCJAG00000016905	BCL6B
ENSCJAT00000055558	ENSCJAG00000005086	HSD17B1
ENSCJAT00000054053	ENSCJAG00000004971	FAM134C
ENSCJAT00000009616	ENSCJAG00000004971	FAM134C
ENSCJAT00000009626	ENSCJAG00000004971	FAM134C
ENSCJAT00000025806	ENSCJAG00000013276	VPS25
ENSCJAT00000025622	ENSCJAG00000013195	unknown
ENSCJAT00000025432	ENSCJAG00000013097	G6PC
ENSCJAT00000025442	ENSCJAG00000013097	G6PC
ENSCJAT00000021021	ENSCJAG00000010785	ARL4D
ENSCJAT00000021015	ENSCJAG00000010785	ARL4D
ENSCJAT00000020551	ENSCJAG00000010482	MPP3
ENSCJAT00000020600	ENSCJAG00000010482	MPP3
ENSCJAT00000020587	ENSCJAG00000010482	MPP3
ENSCJAT00000020571	ENSCJAG00000010482	MPP3
ENSCJAT00000019285	ENSCJAG00000009903	GRN
ENSCJAT00000019327	ENSCJAG00000009903	GRN
ENSCJAT00000019278	ENSCJAG00000009903	GRN
ENSCJAT00000019286	ENSCJAG00000009903	GRN
ENSCJAT00000019302	ENSCJAG00000009903	GRN
ENSCJAT00000019179	ENSCJAG00000009865	FAM171A2
ENSCJAT00000019163	ENSCJAG00000009865	FAM171A2
ENSCJAT00000022789	ENSCJAG00000011685	ALDH3A2
ENSCJAT00000022798	ENSCJAG00000011685	ALDH3A2
ENSCJAT00000023663	ENSCJAG00000012193	TTC19
ENSCJAT00000023677	ENSCJAG00000012208	ZSWIM7
ENSCJAT00000023675	ENSCJAG00000012208	ZSWIM7
ENSCJAT00000023691	ENSCJAG00000012224	C17orf104
ENSCJAT00000023710	ENSCJAG00000012229	CCDC43
ENSCJAT00000023715	ENSCJAG00000012229	CCDC43
ENSCJAT00000023712	ENSCJAG00000012229	CCDC43
ENSCJAT00000023892	ENSCJAG00000012274	ADAM11
ENSCJAT00000024660	ENSCJAG00000012703	PLCD3
ENSCJAT00000024646	ENSCJAG00000012703	PLCD3
ENSCJAT00000024682	ENSCJAG00000012732	ACBD4
ENSCJAT00000056210	ENSCJAG00000012775	MAP3K14
ENSCJAT00000024865	ENSCJAG00000012810	ARHGAP27
ENSCJAT00000061212	ENSCJAG00000019994	NSF
ENSCJAT00000038961	ENSCJAG00000019846	EFCAB3
ENSCJAT00000038963	ENSCJAG00000019846	EFCAB3
ENSCJAT00000054611	ENSCJAG00000033545	unknown
ENSCJAT00000052244	ENSCJAG00000019827	TLK2
ENSCJAT00000038755	ENSCJAG00000019745	DCAF7
ENSCJAT00000038758	ENSCJAG00000019745	DCAF7
ENSCJAT00000038757	ENSCJAG00000019745	DCAF7

ENSCJAT00000038636	ENSCJAG00000019683	CCDC47
ENSCJAT00000038639	ENSCJAG00000019683	CCDC47
ENSCJAT00000038595	ENSCJAG00000019649	DDX42
ENSCJAT00000037950	ENSCJAG00000019350	POLG2
ENSCJAT00000057070	ENSCJAG00000019350	POLG2
ENSCJAT00000037928	ENSCJAG00000019310	DDX5
ENSCJAT00000037901	ENSCJAG00000019310	DDX5
ENSCJAT00000037926	ENSCJAG00000019310	DDX5
ENSCJAT00000003282	ENSCJAG00000001738	PPM1E
ENSCJAT00000003037	ENSCJAG00000001554	DHX40
ENSCJAT00000003021	ENSCJAG00000001554	DHX40
ENSCJAT00000002922	ENSCJAG00000001505	CLTC
ENSCJAT00000002918	ENSCJAG00000001505	CLTC
ENSCJAT00000061231	ENSCJAG00000001479	VMP1
ENSCJAT00000056477	ENSCJAG00000001479	VMP1
ENSCJAT00000054300	ENSCJAG00000001479	VMP1
ENSCJAT00000062003	ENSCJAG00000001479	VMP1
ENSCJAT00000058118	ENSCJAG00000001479	VMP1
ENSCJAT00000055621	ENSCJAG00000032128	RNFT1
ENSCJAT00000058690	ENSCJAG00000032128	RNFT1
ENSCJAT00000002499	ENSCJAG00000001324	INTS2
ENSCJAT00000002493	ENSCJAG00000001324	INTS2
ENSCJAT00000002392	ENSCJAG00000001281	TBX4
ENSCJAT00000002264	ENSCJAG00000001208	PPM1D
ENSCJAT00000002239	ENSCJAG00000001208	PPM1D
ENSCJAT00000002246	ENSCJAG00000001208	PPM1D
ENSCJAT00000002146	ENSCJAG00000001176	C17orf64
ENSCJAT00000030055	ENSCJAG00000015436	C17orf78
ENSCJAT00000029725	ENSCJAG00000015270	DUSP14
ENSCJAT00000029625	ENSCJAG00000015190	DDX52
ENSCJAT00000029619	ENSCJAG00000015190	DDX52
ENSCJAT00000029540	ENSCJAG00000015163	unknown
ENSCJAT00000029206	ENSCJAG00000014961	unknown
ENSCJAT00000029198	ENSCJAG00000014961	unknown
ENSCJAT00000029181	ENSCJAG00000014961	unknown
ENSCJAT00000029104	ENSCJAG00000014952	C17orf50
ENSCJAT00000029054	ENSCJAG00000014853	unknown
ENSCJAT00000029056	ENSCJAG00000014853	unknown
ENSCJAT00000029066	ENSCJAG00000014853	unknown
ENSCJAT00000013020	ENSCJAG00000014853	unknown
ENSCJAT00000028712	ENSCJAG00000014751	FNDC8
ENSCJAT00000028704	ENSCJAG00000014732	RAD51D
ENSCJAT00000028313	ENSCJAG00000014550	TMEM98
ENSCJAT00000058868	ENSCJAG00000014550	TMEM98
ENSCJAT00000005380	ENSCJAG00000014550	TMEM98
ENSCJAT00000027381	ENSCJAG00000014106	unknown
ENSCJAT00000026834	ENSCJAG00000013781	BLMH
ENSCJAT00000026267	ENSCJAG00000013508	NUFIP2
ENSCJAT00000026268	ENSCJAG00000013508	NUFIP2
ENSCJAT00000026225	ENSCJAG00000013358	MYO18A
ENSCJAT00000024734	ENSCJAG00000012729	PIGS
ENSCJAT00000024406	ENSCJAG00000012566	POLDIP2
ENSCJAT00000024263	ENSCJAG00000012529	TMEM97
ENSCJAT00000055299	ENSCJAG00000012529	TMEM97
ENSCJAT00000024133	ENSCJAG00000012425	unknown
ENSCJAT00000024130	ENSCJAG00000012425	unknown
ENSCJAT00000059687	ENSCJAG00000012425	unknown
ENSCJAT00000024137	ENSCJAG00000012425	unknown
ENSCJAT00000024146	ENSCJAG00000012425	unknown

ENSCJAT00000023465	ENSCJAG00000012052	ZMYND15
ENSCJAT00000023456	ENSCJAG00000012052	ZMYND15
ENSCJAT00000022615	ENSCJAG00000011609	CAMTA2
ENSCJAT00000022606	ENSCJAG00000011609	CAMTA2
ENSCJAT00000022619	ENSCJAG00000011609	CAMTA2
ENSCJAT00000022612	ENSCJAG00000011609	CAMTA2
ENSCJAT00000022390	ENSCJAG00000011513	SCIMP
ENSCJAT00000027187	ENSCJAG00000013990	AKAP1
ENSCJAT00000027065	ENSCJAG00000013909	DGKE
ENSCJAT00000021915	ENSCJAG00000011246	PCTP
ENSCJAT00000022029	ENSCJAG00000011311	STXBP4
ENSCJAT00000022752	ENSCJAG00000018448	SPAG9
ENSCJAT00000036553	ENSCJAG00000018627	ANKRD40
ENSCJAT00000036854	ENSCJAG00000018782	RSAD1
ENSCJAT00000036843	ENSCJAG00000018782	RSAD1
ENSCJAT00000036885	ENSCJAG00000018788	ACSF2
ENSCJAT00000036887	ENSCJAG00000018788	ACSF2
ENSCJAT00000036896	ENSCJAG00000018788	ACSF2
ENSCJAT00000037003	ENSCJAG00000018867	TMEM92
ENSCJAT00000013725	ENSCJAG00000007010	TAC4
ENSCJAT00000013747	ENSCJAG00000007010	TAC4
ENSCJAT00000013729	ENSCJAG00000007010	TAC4
ENSCJAT00000013743	ENSCJAG00000007010	TAC4
ENSCJAT00000013738	ENSCJAG00000007010	TAC4
ENSCJAT00000013735	ENSCJAG00000007010	TAC4
ENSCJAT00000014086	ENSCJAG00000007174	unknown
ENSCJAT00000014096	ENSCJAG00000007174	unknown
ENSCJAT00000014118	ENSCJAG00000007206	ZNF652
ENSCJAT00000052794	ENSCJAG00000007395	TTLL6
ENSCJAT00000014588	ENSCJAG00000007442	HOXB9
ENSCJAT00000014593	ENSCJAG00000007454	HOXB8
ENSCJAT00000014731	ENSCJAG00000007520	SNX11
ENSCJAT00000014743	ENSCJAG00000007520	SNX11
ENSCJAT00000014733	ENSCJAG00000007520	SNX11
ENSCJAT00000014799	ENSCJAG00000007542	NFE2L1
ENSCJAT00000015006	ENSCJAG00000007692	PRR15L
ENSCJAT00000015037	ENSCJAG00000007693	PNPO
ENSCJAT00000058835	ENSCJAG00000007693	PNPO
ENSCJAT00000061531	ENSCJAG00000007693	PNPO
ENSCJAT00000015043	ENSCJAG00000007693	PNPO
ENSCJAT00000015173	ENSCJAG00000007753	SCRN2
ENSCJAT00000015223	ENSCJAG00000007787	MRPL10
ENSCJAT00000008718	ENSCJAG00000007787	MRPL10
ENSCJAT00000015766	ENSCJAG00000008080	MRPL45
ENSCJAT00000059023	ENSCJAG00000008215	PCGF2
ENSCJAT00000059600	ENSCJAG00000008215	PCGF2
ENSCJAT00000016201	ENSCJAG00000008311	RPL23
ENSCJAT00000016505	ENSCJAG00000008419	CACNB1
ENSCJAT00000055469	ENSCJAG00000008957	IKZF3
ENSCJAT00000052276	ENSCJAG00000008957	IKZF3
ENSCJAT00000060070	ENSCJAG00000008957	IKZF3
ENSCJAT00000058849	ENSCJAG00000008957	IKZF3
ENSCJAT00000063670	ENSCJAG00000008957	IKZF3
ENSCJAT00000061371	ENSCJAG00000008957	IKZF3
ENSCJAT00000017363	ENSCJAG00000008957	IKZF3
ENSCJAT00000017332	ENSCJAG00000008957	IKZF3
ENSCJAT00000060881	ENSCJAG00000008957	IKZF3
ENSCJAT00000058307	ENSCJAG00000008957	IKZF3
ENSCJAT00000031159	ENSCJAG00000016012	MSL1

ENSCJAT00000031166	ENSCJAG00000016012	MSL1
ENSCJAT00000026092	ENSCJAG00000013421	CDC6
ENSCJAT00000026329	ENSCJAG00000013478	TOP2A
ENSCJAT00000026300	ENSCJAG00000013478	TOP2A
ENSCJAT00000043198	ENSCJAG00000031345	unknown
ENSCJAT00000061230	ENSCJAG00000002348	unknown
ENSCJAT00000004454	ENSCJAG00000002348	unknown
ENSCJAT00000065626	ENSCJAG00000002348	unknown
ENSCJAT00000004600	ENSCJAG00000029756	KRT19
ENSCJAT00000000740	ENSCJAG00000000406	PTRF
ENSCJAT00000057795	ENSCJAG00000000265	GNA13
ENSCJAT00000000476	ENSCJAG00000000265	GNA13
ENSCJAT00000052286	ENSCJAG00000000265	GNA13
ENSCJAT00000000353	ENSCJAG00000000190	ABCA8
ENSCJAT00000000355	ENSCJAG00000000190	ABCA8
ENSCJAT00000000331	ENSCJAG00000000190	ABCA8
ENSCJAT00000000342	ENSCJAG00000000190	ABCA8
ENSCJAT00000000362	ENSCJAG00000000190	ABCA8
ENSCJAT00000000254	ENSCJAG00000000122	MAP2K6
ENSCJAT00000058294	ENSCJAG00000023169	KCNJ16
ENSCJAT00000044154	ENSCJAG00000023169	KCNJ16
ENSCJAT00000000199	ENSCJAG00000000109	SOX9
ENSCJAT00000000197	ENSCJAG00000000109	SOX9
ENSCJAT00000039094	ENSCJAG00000019911	CPSF4L
ENSCJAT00000039088	ENSCJAG00000019909	CDC42EP4
ENSCJAT00000029129	ENSCJAG00000014960	BTBD17
ENSCJAT00000028997	ENSCJAG00000014894	CD300A
ENSCJAT00000029000	ENSCJAG00000014894	CD300A
ENSCJAT00000028955	ENSCJAG00000014867	CD300C
ENSCJAT00000028175	ENSCJAG00000014468	GGA3
ENSCJAT00000028163	ENSCJAG00000014468	GGA3
ENSCJAT00000058887	ENSCJAG00000014303	CASKIN2
ENSCJAT00000027820	ENSCJAG00000014303	CASKIN2
ENSCJAT00000027844	ENSCJAG00000014303	CASKIN2
ENSCJAT00000027748	ENSCJAG00000014278	TSEN54
ENSCJAT00000027778	ENSCJAG00000014278	TSEN54
ENSCJAT00000027121	ENSCJAG00000013928	FBF1
ENSCJAT00000026783	ENSCJAG00000013738	SRP68
ENSCJAT00000026685	ENSCJAG00000013734	ZACN
ENSCJAT00000026525	ENSCJAG00000013649	UBALD2
ENSCJAT00000026530	ENSCJAG00000013649	UBALD2
ENSCJAT00000055406	ENSCJAG00000013500	ST6GALNAC2
ENSCJAT00000062626	ENSCJAG00000013500	ST6GALNAC2
ENSCJAT00000025878	ENSCJAG00000013261	SEC14L1
ENSCJAT00000025833	ENSCJAG00000013261	SEC14L1
ENSCJAT00000006454	ENSCJAG00000003364	unknown
ENSCJAT00000059480	ENSCJAG00000018041	CCDC57
ENSCJAT00000034955	ENSCJAG00000017923	FOXK2
ENSCJAT00000034911	ENSCJAG00000017895	FN3K
ENSCJAT00000058131	ENSCJAG00000004525	MPHOSPH8
ENSCJAT00000017893	ENSCJAG00000004525	MPHOSPH8
ENSCJAT00000058904	ENSCJAG00000004621	ZMYM2
ENSCJAT00000009415	ENSCJAG00000004815	IFT88
ENSCJAT00000009366	ENSCJAG00000004815	IFT88
ENSCJAT00000036712	ENSCJAG00000018710	MICU2
ENSCJAT00000036991	ENSCJAG00000018861	unknown
ENSCJAT00000036999	ENSCJAG00000018861	unknown
ENSCJAT00000036987	ENSCJAG00000018861	unknown
ENSCJAT00000037170	ENSCJAG00000018946	CENPJ

ENSCJAT00000037921	ENSCJAG00000019338	GPR12
ENSCJAT00000037969	ENSCJAG00000019369	RASL11A
ENSCJAT00000038022	ENSCJAG00000019388	GSX1
ENSCJAT00000036444	ENSCJAG00000018570	B3GALTL
ENSCJAT00000035488	ENSCJAG00000018144	CCNA1
ENSCJAT00000035482	ENSCJAG00000018144	CCNA1
ENSCJAT00000035480	ENSCJAG00000018144	CCNA1
ENSCJAT00000035432	ENSCJAG00000018138	unknown
ENSCJAT00000035428	ENSCJAG00000018134	unknown
ENSCJAT00000035426	ENSCJAG00000018134	unknown
ENSCJAT00000059537	ENSCJAG00000017833	COG6
ENSCJAT00000034722	ENSCJAG00000017805	MRPS31
ENSCJAT00000034720	ENSCJAG00000017805	MRPS31
ENSCJAT00000055801	ENSCJAG00000017805	MRPS31
ENSCJAT00000034679	ENSCJAG00000017791	unknown
ENSCJAT00000054982	ENSCJAG00000017791	unknown
ENSCJAT00000053974	ENSCJAG00000017782	ELF1
ENSCJAT00000059450	ENSCJAG00000019220	GTF2F2
ENSCJAT00000007168	ENSCJAG00000003751	unknown
ENSCJAT00000007324	ENSCJAG00000003764	UBE3A
ENSCJAT00000037342	ENSCJAG00000009977	GABRG3
ENSCJAT00000033983	ENSCJAG00000017370	TJP1
ENSCJAT00000033875	ENSCJAG00000017370	TJP1
ENSCJAT00000033575	ENSCJAG00000017258	SELS
ENSCJAT00000033544	ENSCJAG00000017247	CHSY1
ENSCJAT00000033548	ENSCJAG00000017247	CHSY1
ENSCJAT00000033541	ENSCJAG00000017247	CHSY1
ENSCJAT00000033179	ENSCJAG00000017029	MEF2A
ENSCJAT00000033169	ENSCJAG00000017029	MEF2A
ENSCJAT00000033150	ENSCJAG00000017029	MEF2A
ENSCJAT00000057536	ENSCJAG00000016887	FAM169B
ENSCJAT00000040111	ENSCJAG00000020434	unknown
ENSCJAT00000018404	ENSCJAG00000009482	SEMA4B
ENSCJAT00000059347	ENSCJAG00000009678	MAN2A2
ENSCJAT00000061898	ENSCJAG00000009678	MAN2A2
ENSCJAT00000058042	ENSCJAG00000009678	MAN2A2
ENSCJAT00000018860	ENSCJAG00000009678	MAN2A2
ENSCJAT00000018850	ENSCJAG00000009678	MAN2A2
ENSCJAT00000018967	ENSCJAG00000009738	FES
ENSCJAT00000018951	ENSCJAG00000009738	FES
ENSCJAT00000018961	ENSCJAG00000009738	FES
ENSCJAT00000024641	ENSCJAG00000012709	PLIN1
ENSCJAT00000024680	ENSCJAG00000012723	KIF7
ENSCJAT00000024820	ENSCJAG00000012773	POLG
ENSCJAT00000024967	ENSCJAG00000012881	RLBP1
ENSCJAT00000025168	ENSCJAG00000012944	ACAN
ENSCJAT00000031532	ENSCJAG00000016173	ZNF592
ENSCJAT00000031510	ENSCJAG00000016173	ZNF592
ENSCJAT00000031442	ENSCJAG00000016146	SEC11A
ENSCJAT00000031352	ENSCJAG00000016119	ZSCAN2
ENSCJAT00000031344	ENSCJAG00000016119	ZSCAN2
ENSCJAT00000003865	ENSCJAG00000015877	C15orf40
ENSCJAT00000030882	ENSCJAG00000015841	BTBD1
ENSCJAT00000055173	ENSCJAG00000015841	BTBD1
ENSCJAT00000030854	ENSCJAG00000015841	BTBD1
ENSCJAT00000030800	ENSCJAG00000015800	TM6SF1
ENSCJAT00000030768	ENSCJAG00000015800	TM6SF1
ENSCJAT00000030820	ENSCJAG00000015800	TM6SF1
ENSCJAT00000030763	ENSCJAG00000015800	TM6SF1

ENSCJAT00000030501	ENSCJAG00000015607	EFTUD1
ENSCJAT00000061660	ENSCJAG00000015607	EFTUD1
ENSCJAT00000015551	ENSCJAG00000007965	STARD5
ENSCJAT00000015533	ENSCJAG00000007965	STARD5
ENSCJAT00000015327	ENSCJAG00000007830	C15orf26
ENSCJAT00000015278	ENSCJAG00000007811	MESDC2
ENSCJAT00000015154	ENSCJAG00000007708	CEMIP
ENSCJAT00000056819	ENSCJAG00000007705	ABHD17C
ENSCJAT00000014797	ENSCJAG00000007560	unknown
ENSCJAT00000008845	ENSCJAG00000004585	PTPN18
ENSCJAT00000008832	ENSCJAG00000004585	PTPN18
ENSCJAT00000021499	ENSCJAG00000011033	PLEKHB2
ENSCJAT00000062885	ENSCJAG00000011033	PLEKHB2
ENSCJAT00000053237	ENSCJAG00000011033	PLEKHB2
ENSCJAT00000021151	ENSCJAG00000010759	SAP130
ENSCJAT00000021137	ENSCJAG00000010759	SAP130
ENSCJAT00000021130	ENSCJAG00000010759	SAP130
ENSCJAT00000020950	ENSCJAG00000010743	AMMECR1L
ENSCJAT00000020686	ENSCJAG00000010560	IWS1
ENSCJAT00000063103	ENSCJAG00000010560	IWS1
ENSCJAT00000040910	ENSCJAG00000010560	IWS1
ENSCJAT00000059593	ENSCJAG00000011104	NUP35
ENSCJAT00000021746	ENSCJAG00000011160	DUSP19
ENSCJAT00000021956	ENSCJAG00000011255	FRZB
ENSCJAT00000058845	ENSCJAG00000011295	DNAJC10
ENSCJAT00000042352	ENSCJAG00000021531	unknown
ENSCJAT00000042349	ENSCJAG00000021531	unknown
ENSCJAT00000042351	ENSCJAG00000021531	unknown
ENSCJAT00000042334	ENSCJAG00000021522	DFNB59
ENSCJAT00000042717	ENSCJAG00000021732	unknown
ENSCJAT00000042718	ENSCJAG00000021732	unknown
ENSCJAT00000054003	ENSCJAG00000008340	NFE2L2
ENSCJAT00000016259	ENSCJAG00000008340	NFE2L2
ENSCJAT00000016140	ENSCJAG00000008284	HOXD8
ENSCJAT00000016050	ENSCJAG00000008226	KIAA1715
ENSCJAT00000015578	ENSCJAG00000007947	GPR155
ENSCJAT00000015573	ENSCJAG00000007947	GPR155
ENSCJAT00000053109	ENSCJAG00000007507	PDK1
ENSCJAT00000052306	ENSCJAG00000007507	PDK1
ENSCJAT00000014726	ENSCJAG00000007507	PDK1
ENSCJAT00000014085	ENSCJAG00000007191	CYBRD1
ENSCJAT00000013964	ENSCJAG00000007075	TLK1
ENSCJAT00000040952	ENSCJAG00000006777	METTL5
ENSCJAT00000013211	ENSCJAG00000006777	METTL5
ENSCJAT00000013198	ENSCJAG00000006710	SSB
ENSCJAT00000012767	ENSCJAG00000006532	KLHL41
ENSCJAT00000055275	ENSCJAG00000006532	KLHL41
ENSCJAT00000012638	ENSCJAG00000006471	XIRP2
ENSCJAT00000055424	ENSCJAG00000006448	SCN7A
ENSCJAT00000012574	ENSCJAG00000006448	SCN7A
ENSCJAT00000012318	ENSCJAG00000006296	TTC21B
ENSCJAT00000012309	ENSCJAG00000006296	TTC21B
ENSCJAT00000012205	ENSCJAG00000006274	GALNT3
ENSCJAT00000012335	ENSCJAG00000006141	SCN2A
ENSCJAT00000011686	ENSCJAG00000006024	FIGN
ENSCJAT00000011621	ENSCJAG00000005956	IFIH1
ENSCJAT00000011611	ENSCJAG00000005956	IFIH1
ENSCJAT00000010797	ENSCJAG00000005572	PSMD14
ENSCJAT00000029034	ENSCJAG00000014862	ITGB6

ENSCJAT00000063630	ENSCJAG00000014862	ITGB6
ENSCJAT00000029027	ENSCJAG00000014862	ITGB6
ENSCJAT00000029484	ENSCJAG00000015117	WDSUB1
ENSCJAT00000029668	ENSCJAG00000015222	unknown
ENSCJAT00000029653	ENSCJAG00000015222	unknown
ENSCJAT00000030059	ENSCJAG00000015426	GALNT5
ENSCJAT00000030222	ENSCJAG00000015533	ARL6IP6
ENSCJAT00000030502	ENSCJAG00000015629	FMNL2
ENSCJAT00000030478	ENSCJAG00000015629	FMNL2
ENSCJAT00000006445	ENSCJAG00000003346	STAM2
ENSCJAT00000006438	ENSCJAG00000003346	STAM2
ENSCJAT00000006360	ENSCJAG00000003311	ARL5A
ENSCJAT00000016137	ENSCJAG00000003311	ARL5A
ENSCJAT00000006080	ENSCJAG00000003138	RIF1
ENSCJAT00000006089	ENSCJAG00000003138	RIF1
ENSCJAT00000006015	ENSCJAG00000003134	TNFAIP6
ENSCJAT00000005926	ENSCJAG00000003086	MMADHC
ENSCJAT00000005931	ENSCJAG00000003086	MMADHC
ENSCJAT00000024566	ENSCJAG00000012637	EPC2
ENSCJAT00000024586	ENSCJAG00000012637	EPC2
ENSCJAT00000024452	ENSCJAG00000012594	MBD5
ENSCJAT00000024308	ENSCJAG00000012496	ACVR2A
ENSCJAT00000063259	ENSCJAG00000012496	ACVR2A
ENSCJAT00000023371	ENSCJAG00000011967	DARS
ENSCJAT00000023337	ENSCJAG00000011967	DARS
ENSCJAT00000023019	ENSCJAG00000011842	UBXN4
ENSCJAT00000023030	ENSCJAG00000011842	UBXN4
ENSCJAT00000022660	ENSCJAG00000011643	RAB3GAP1
ENSCJAT00000022648	ENSCJAG00000011643	RAB3GAP1
ENSCJAT00000027571	ENSCJAG00000014139	SLC35F5
ENSCJAT00000027594	ENSCJAG00000014139	SLC35F5
ENSCJAT00000058787	ENSCJAG00000014139	SLC35F5
ENSCJAT00000027461	ENSCJAG00000014091	ACTR3
ENSCJAT00000027418	ENSCJAG00000014091	ACTR3
ENSCJAT00000027441	ENSCJAG00000014091	ACTR3
ENSCJAT00000027451	ENSCJAG00000014091	ACTR3
ENSCJAT00000038555	ENSCJAG00000014091	ACTR3
ENSCJAT00000027459	ENSCJAG00000014091	ACTR3
ENSCJAT00000042102	ENSCJAG00000021414	DDX18
ENSCJAT00000056610	ENSCJAG00000032138	TMEM37
ENSCJAT00000042152	ENSCJAG00000021440	CFAP221
ENSCJAT00000026014	ENSCJAG00000013386	NIFK
ENSCJAT00000000569	ENSCJAG00000013386	NIFK
ENSCJAT00000011510	ENSCJAG00000005883	ITGAV
ENSCJAT00000062732	ENSCJAG00000005883	ITGAV
ENSCJAT00000058248	ENSCJAG00000005883	ITGAV
ENSCJAT00000063500	ENSCJAG00000005883	ITGAV
ENSCJAT00000010201	ENSCJAG00000005210	COL3A1
ENSCJAT00000031253	ENSCJAG00000005210	COL3A1
ENSCJAT00000038095	ENSCJAG00000005579	unknown
ENSCJAT00000010777	ENSCJAG00000005579	unknown
ENSCJAT00000045190	ENSCJAG00000005590	PMS1
ENSCJAT00000062763	ENSCJAG00000005706	INPP1
ENSCJAT00000011078	ENSCJAG00000005706	INPP1
ENSCJAT00000006897	ENSCJAG00000003591	STK17B
ENSCJAT00000007595	ENSCJAG00000003902	SF3B1
ENSCJAT00000060810	ENSCJAG00000004173	SPATS2L
ENSCJAT00000055739	ENSCJAG00000004319	CLK1
ENSCJAT00000038634	ENSCJAG00000004505	CFLAR

ENSCJAT00000042409	ENSCJAG00000004505	CFLAR	
ENSCJAT00000008676	ENSCJAG00000004505	CFLAR	
ENSCJAT00000054974	ENSCJAG00000004505	CFLAR	
ENSCJAT00000008685	ENSCJAG00000004505	CFLAR	
ENSCJAT00000008668	ENSCJAG00000004505	CFLAR	
ENSCJAT00000060460	ENSCJAG00000004551	CASP8	
ENSCJAT00000008721	ENSCJAG00000004551	CASP8	
ENSCJAT00000053740	ENSCJAG00000004568	TRAK2	
ENSCJAT00000008910	ENSCJAG00000004568	TRAK2	
ENSCJAT00000009476	ENSCJAG00000004892	CDK15	
ENSCJAT00000061463	ENSCJAG00000004892	CDK15	
ENSCJAT00000053243	ENSCJAG00000005224	CARF	
ENSCJAT00000010148	ENSCJAG00000005224	CARF	
ENSCJAT00000010158	ENSCJAG00000005224	CARF	
ENSCJAT00000010507	ENSCJAG00000005399	ABI2	
ENSCJAT00000010499	ENSCJAG00000005399	ABI2	
ENSCJAT00000060924	ENSCJAG00000005399	ABI2	
ENSCJAT00000010378	ENSCJAG00000005339	RAPH1	
ENSCJAT00000010350	ENSCJAG00000005339	RAPH1	
ENSCJAT00000010583	ENSCJAG00000005457	CD28	
ENSCJAT00000010585	ENSCJAG00000005457	CD28	
ENSCJAT00000010573	ENSCJAG00000005457	CD28	
ENSCJAT00000010568	ENSCJAG00000005457	CD28	
ENSCJAT00000010636	ENSCJAG00000005516	ICOS	
ENSCJAT00000030617	ENSCJAG00000005577	NRP2	
ENSCJAT00000058040	ENSCJAG00000022889	GPR1	
ENSCJAT00000061984	ENSCJAG00000006039	CREB1	
ENSCJAT00000011764	ENSCJAG00000006039	CREB1	
ENSCJAT00000052666	ENSCJAG00000006039	CREB1	
ENSCJAT00000062625	ENSCJAG00000002726	unknown	
ENSCJAT00000005235	ENSCJAG00000002726	unknown	
ENSCJAT00000005228	ENSCJAG00000002726	unknown	
ENSCJAT00000020978	ENSCJAG00000010689	MAP2	
ENSCJAT00000021253	ENSCJAG00000010851	KANSL1L	
ENSCJAT00000021222	ENSCJAG00000010851	KANSL1L	
ENSCJAT00000021325	ENSCJAG00000010924	MYL1	
ENSCJAT00000060081	ENSCJAG00000010985	CPS1	
ENSCJAT00000065027	ENSCJAG00000036269	unknown	
ENSCJAT00000022711	ENSCJAG00000011695	ATIC	
ENSCJAT00000022723	ENSCJAG00000011695	ATIC	
ENSCJAT00000023965	ENSCJAG00000012374		4-Mar
ENSCJAT00000013027	ENSCJAG00000012374		4-Mar
ENSCJAT00000057992	ENSCJAG00000022440	GPBAR1	
ENSCJAT00000054542	ENSCJAG00000022440	GPBAR1	
ENSCJAT00000043425	ENSCJAG00000022440	GPBAR1	
ENSCJAT00000021585	ENSCJAG00000010983	USP37	
ENSCJAT00000021564	ENSCJAG00000010983	USP37	
ENSCJAT00000021860	ENSCJAG00000011192	RNF25	
ENSCJAT00000061049	ENSCJAG00000011192	RNF25	
ENSCJAT00000052557	ENSCJAG00000011294	TTLL4	
ENSCJAT00000022062	ENSCJAG00000011342	CYP27A1	
ENSCJAT00000022082	ENSCJAG00000011342	CYP27A1	
ENSCJAT00000022320	ENSCJAG00000011478	SLC23A3	
ENSCJAT00000061277	ENSCJAG00000011478	SLC23A3	
ENSCJAT00000022387	ENSCJAG00000011512	ABCB6	
ENSCJAT00000060221	ENSCJAG00000011512	ABCB6	
ENSCJAT00000022917	ENSCJAG00000011802	SPEG	
ENSCJAT00000052992	ENSCJAG00000011802	SPEG	
ENSCJAT00000060578	ENSCJAG00000011802	SPEG	

ENSCJAT00000023151	ENSCJAG00000011934	INHA
ENSCJAT00000023339	ENSCJAG00000011943	SLC4A3
ENSCJAT00000061354	ENSCJAG00000011943	SLC4A3
ENSCJAT00000019531	ENSCJAG00000011943	SLC4A3
ENSCJAT00000019520	ENSCJAG00000011943	SLC4A3
ENSCJAT00000023584	ENSCJAG00000012088	PAX3
ENSCJAT00000023568	ENSCJAG00000012088	PAX3
ENSCJAT00000023904	ENSCJAG00000012308	ACSL3
ENSCJAT00000014396	ENSCJAG00000012308	ACSL3
ENSCJAT00000024097	ENSCJAG00000012404	SERPINE2
ENSCJAT00000024086	ENSCJAG00000012404	SERPINE2
ENSCJAT00000059716	ENSCJAG00000012449	CUL3
ENSCJAT00000024239	ENSCJAG00000012449	CUL3
ENSCJAT00000057891	ENSCJAG00000012449	CUL3
ENSCJAT00000057587	ENSCJAG00000012449	CUL3
ENSCJAT00000024523	ENSCJAG00000012521	DOCK10
ENSCJAT00000024654	ENSCJAG00000012708	NYAP2
ENSCJAT00000056888	ENSCJAG00000012973	MFF
ENSCJAT00000025212	ENSCJAG00000012973	MFF
ENSCJAT00000061150	ENSCJAG00000012973	MFF
ENSCJAT00000058657	ENSCJAG00000012973	MFF
ENSCJAT00000025207	ENSCJAG00000012973	MFF
ENSCJAT00000025197	ENSCJAG00000012973	MFF
ENSCJAT00000025325	ENSCJAG00000013039	SLC19A3
ENSCJAT00000025738	ENSCJAG00000013244	SLC16A14
ENSCJAT00000025831	ENSCJAG00000013289	SP140
ENSCJAT00000052195	ENSCJAG00000013289	SP140
ENSCJAT00000059871	ENSCJAG00000013354	ITM2C
ENSCJAT00000062943	ENSCJAG00000013354	ITM2C
ENSCJAT00000057312	ENSCJAG00000013354	ITM2C
ENSCJAT00000025957	ENSCJAG00000013354	ITM2C
ENSCJAT00000026243	ENSCJAG00000013379	PSMD1
ENSCJAT00000026240	ENSCJAG00000013379	PSMD1
ENSCJAT00000026258	ENSCJAG00000013503	HTR2B
ENSCJAT00000052386	ENSCJAG00000013503	HTR2B
ENSCJAT00000063550	ENSCJAG00000013596	NMUR1
ENSCJAT00000058348	ENSCJAG00000013596	NMUR1
ENSCJAT00000026426	ENSCJAG00000013596	NMUR1
ENSCJAT00000026450	ENSCJAG00000013602	unknown
ENSCJAT00000026458	ENSCJAG00000013611	unknown
ENSCJAT00000063120	ENSCJAG00000013611	unknown
ENSCJAT00000025568	ENSCJAG00000013611	unknown
ENSCJAT00000026485	ENSCJAG00000013615	PDE6D
ENSCJAT00000053337	ENSCJAG00000013630	COPS7B
ENSCJAT00000026569	ENSCJAG00000013630	COPS7B
ENSCJAT00000026710	ENSCJAG00000013711	ECEL1
ENSCJAT00000026704	ENSCJAG00000013711	ECEL1
ENSCJAT00000063646	ENSCJAG00000013756	CHRND
ENSCJAT00000052112	ENSCJAG00000013756	CHRND
ENSCJAT00000026735	ENSCJAG00000013756	CHRND
ENSCJAT00000026727	ENSCJAG00000013756	CHRND
ENSCJAT00000026766	ENSCJAG00000013767	CHRNG
ENSCJAT00000026769	ENSCJAG00000013767	CHRNG
ENSCJAT00000052843	ENSCJAG00000014074	MA_R131_JSME7C4R
ENSCJAT00000027398	ENSCJAG00000014074	MA_R131_JSME7C4R
ENSCJAT00000066085	ENSCJAG00000014142	MROH2A
ENSCJAT00000027717	ENSCJAG00000014263	SPP2
ENSCJAT00000000908	ENSCJAG00000000491	LRRFIP1
ENSCJAT00000000882	ENSCJAG00000000484	RBM44

ENSCJAT00000058551	ENSCJAG00000000476	UBE2F
ENSCJAT00000055083	ENSCJAG00000000476	UBE2F
ENSCJAT00000000864	ENSCJAG00000000476	UBE2F
ENSCJAT00000054782	ENSCJAG00000000476	UBE2F
ENSCJAT00000000839	ENSCJAG00000000459	KLHL30
ENSCJAT00000000841	ENSCJAG00000000459	KLHL30
ENSCJAT00000000834	ENSCJAG00000000459	KLHL30
ENSCJAT00000000823	ENSCJAG00000000456	FAM132B
ENSCJAT00000000820	ENSCJAG00000000443	ILKAP
ENSCJAT00000000758	ENSCJAG00000000414	ASB1
ENSCJAT00000037413	ENSCJAG00000019077	MYEOV2
ENSCJAT00000056824	ENSCJAG00000006565	CAPN10
ENSCJAT00000012804	ENSCJAG00000006565	CAPN10
ENSCJAT00000012800	ENSCJAG00000006565	CAPN10
ENSCJAT00000012798	ENSCJAG00000006565	CAPN10
ENSCJAT00000061305	ENSCJAG00000006565	CAPN10
ENSCJAT00000056338	ENSCJAG00000006565	CAPN10
ENSCJAT00000064364	ENSCJAG00000036468	unknown
ENSCJAT00000040186	ENSCJAG00000020469	AKR1C4
ENSCJAT00000040219	ENSCJAG00000020489	TUBAL3
ENSCJAT00000040251	ENSCJAG00000020501	ASB13
ENSCJAT00000040299	ENSCJAG00000020527	ANKRD16
ENSCJAT00000052308	ENSCJAG00000020527	ANKRD16
ENSCJAT00000040622	ENSCJAG00000020679	unknown
ENSCJAT00000057227	ENSCJAG00000020685	UPF2
ENSCJAT00000018671	ENSCJAG00000009623	CAMK1D
ENSCJAT00000018366	ENSCJAG00000009474	BEND7
ENSCJAT00000060386	ENSCJAG00000009401	FAM107B
ENSCJAT00000017982	ENSCJAG00000009261	DCLRE1C
ENSCJAT00000017979	ENSCJAG00000009261	DCLRE1C
ENSCJAT00000018005	ENSCJAG00000009261	DCLRE1C
ENSCJAT00000017990	ENSCJAG00000009261	DCLRE1C
ENSCJAT00000017477	ENSCJAG00000009020	PTER
ENSCJAT00000017454	ENSCJAG00000009016	C1QL3
ENSCJAT00000017344	ENSCJAG00000008967	CUBN
ENSCJAT00000061114	ENSCJAG00000008967	CUBN
ENSCJAT00000062318	ENSCJAG00000008937	TRDMT1
ENSCJAT00000017321	ENSCJAG00000008937	TRDMT1
ENSCJAT00000058470	ENSCJAG00000008937	TRDMT1
ENSCJAT00000016695	ENSCJAG00000008561	TMEM236
ENSCJAT00000016483	ENSCJAG00000008439	SLC39A12
ENSCJAT00000016480	ENSCJAG00000008439	SLC39A12
ENSCJAT00000016428	ENSCJAG00000008439	SLC39A12
ENSCJAT00000059250	ENSCJAG00000008439	SLC39A12
ENSCJAT00000060316	ENSCJAG00000008439	SLC39A12
ENSCJAT00000016149	ENSCJAG00000008278	ARL5B
ENSCJAT00000053694	ENSCJAG00000008267	unknown
ENSCJAT00000057573	ENSCJAG00000007956	SPAG6
ENSCJAT00000015479	ENSCJAG00000007919	PIP4K2A
ENSCJAT00000015343	ENSCJAG00000007860	unknown
ENSCJAT00000015337	ENSCJAG00000007850	C10orf67
ENSCJAT00000015248	ENSCJAG00000007722	KIAA1217
ENSCJAT00000016219	ENSCJAG00000007722	KIAA1217
ENSCJAT00000014846	ENSCJAG00000007593	THNSL1
ENSCJAT00000054600	ENSCJAG00000004775	ACBD5
ENSCJAT00000061038	ENSCJAG00000004775	ACBD5
ENSCJAT00000053395	ENSCJAG00000004775	ACBD5
ENSCJAT00000053743	ENSCJAG00000004775	ACBD5
ENSCJAT00000009256	ENSCJAG00000004775	ACBD5

ENSCJAT00000009261	ENSCJAG00000004775	ACBD5
ENSCJAT00000009467	ENSCJAG00000004830	YME1L1
ENSCJAT00000053934	ENSCJAG00000004830	YME1L1
ENSCJAT00000058874	ENSCJAG00000004830	YME1L1
ENSCJAT00000009427	ENSCJAG00000004830	YME1L1
ENSCJAT00000025227	ENSCJAG00000013002	PTCHD3
ENSCJAT00000025318	ENSCJAG00000013033	MKX
ENSCJAT00000025440	ENSCJAG00000013079	MPP7
ENSCJAT00000025841	ENSCJAG00000013306	unknown
ENSCJAT00000025883	ENSCJAG00000013316	ZNF438
ENSCJAT00000060653	ENSCJAG00000013332	ZEB1
ENSCJAT00000026497	ENSCJAG00000013540	KIF5B
ENSCJAT00000026412	ENSCJAG00000013540	KIF5B
ENSCJAT00000026698	ENSCJAG00000013701	ITGB1
ENSCJAT00000055652	ENSCJAG00000013701	ITGB1
ENSCJAT00000026706	ENSCJAG00000013701	ITGB1
ENSCJAT00000026888	ENSCJAG00000013814	PARD3
ENSCJAT00000063211	ENSCJAG00000014005	CUL2
ENSCJAT00000021496	ENSCJAG00000011020	VWA1
ENSCJAT00000021358	ENSCJAG00000010957	SSU72
ENSCJAT00000021029	ENSCJAG00000010782	unknown
ENSCJAT00000021023	ENSCJAG00000010782	unknown
ENSCJAT00000021011	ENSCJAG00000010782	unknown
ENSCJAT00000054948	ENSCJAG00000002454	unknown
ENSCJAT00000004719	ENSCJAG00000002454	unknown
ENSCJAT00000057703	ENSCJAG00000002471	RER1
ENSCJAT00000004775	ENSCJAG00000002471	RER1
ENSCJAT00000004787	ENSCJAG00000002487	PEX10
ENSCJAT00000004822	ENSCJAG00000002498	PLCH2
ENSCJAT00000004863	ENSCJAG00000002520	PANK4
ENSCJAT00000061301	ENSCJAG00000002648	TTC34
ENSCJAT00000005297	ENSCJAG00000002757	WRAP73
ENSCJAT00000005290	ENSCJAG00000002757	WRAP73
ENSCJAT00000005281	ENSCJAG00000002757	WRAP73
ENSCJAT00000000299	ENSCJAG00000000170	ESPN
ENSCJAT00000000675	ENSCJAG00000000359	unknown
ENSCJAT00000000681	ENSCJAG00000000376	CA6
ENSCJAT00000000686	ENSCJAG00000000376	CA6
ENSCJAT00000000845	ENSCJAG00000000442	PIK3CD
ENSCJAT00000000832	ENSCJAG00000000442	PIK3CD
ENSCJAT00000000892	ENSCJAG00000000490	CTNNBIP1
ENSCJAT00000010281	ENSCJAG00000005184	KIF1B
ENSCJAT00000009950	ENSCJAG00000005090	PGD
ENSCJAT00000009906	ENSCJAG00000005090	PGD
ENSCJAT00000009984	ENSCJAG00000005090	PGD
ENSCJAT00000056964	ENSCJAG00000005090	PGD
ENSCJAT00000009702	ENSCJAG00000005030	C1orf127
ENSCJAT00000009708	ENSCJAG00000005030	C1orf127
ENSCJAT00000004586	ENSCJAG00000002381	TARDBP
ENSCJAT00000004549	ENSCJAG00000002381	TARDBP
ENSCJAT00000004572	ENSCJAG00000002381	TARDBP
ENSCJAT00000025865	ENSCJAG00000013319	AGTRAP
ENSCJAT00000026106	ENSCJAG00000013347	CLCN6
ENSCJAT00000026653	ENSCJAG00000013719	AADACL4
ENSCJAT00000026678	ENSCJAG00000013722	AADACL3
ENSCJAT00000026736	ENSCJAG00000013763	unknown
ENSCJAT00000026797	ENSCJAG00000013782	PRDM2
ENSCJAT00000026818	ENSCJAG00000013782	PRDM2
ENSCJAT00000017031	ENSCJAG00000008732	CASP9

ENSCJAT00000057183	ENSCJAG00000008732	CASP9
ENSCJAT00000016680	ENSCJAG00000008527	SPEN
ENSCJAT00000062420	ENSCJAG00000008527	SPEN
ENSCJAT00000016625	ENSCJAG00000008527	SPEN
ENSCJAT00000016036	ENSCJAG00000008196	SPATA21
ENSCJAT00000015535	ENSCJAG00000007927	SDHB
ENSCJAT00000015526	ENSCJAG00000007927	SDHB
ENSCJAT00000014036	ENSCJAG00000006886	UBR4
ENSCJAT00000013923	ENSCJAG00000006886	UBR4
ENSCJAT00000013414	ENSCJAG00000006824	EMC1
ENSCJAT00000013424	ENSCJAG00000006824	EMC1
ENSCJAT00000013419	ENSCJAG00000006824	EMC1
ENSCJAT00000013280	ENSCJAG00000006796	unknown
ENSCJAT00000013051	ENSCJAG00000006699	unknown
ENSCJAT00000013057	ENSCJAG00000006699	unknown
ENSCJAT00000012860	ENSCJAG00000006603	PLA2G5
ENSCJAT00000054887	ENSCJAG00000006603	PLA2G5
ENSCJAT00000012777	ENSCJAG00000006551	VWA5B1
ENSCJAT00000012736	ENSCJAG00000006542	CAMK2N1
ENSCJAT00000012496	ENSCJAG00000006426	KIF17
ENSCJAT00000012483	ENSCJAG00000006422	SH2D5
ENSCJAT00000061168	ENSCJAG00000006422	SH2D5
ENSCJAT00000012393	ENSCJAG00000006258	EIF4G3
ENSCJAT00000012358	ENSCJAG00000006258	EIF4G3
ENSCJAT00000011553	ENSCJAG00000005940	HSPG2
ENSCJAT00000011421	ENSCJAG00000005869	ZBTB40
ENSCJAT00000055705	ENSCJAG00000005512	unknown
ENSCJAT00000010792	ENSCJAG00000005512	unknown
ENSCJAT00000010787	ENSCJAG00000005512	unknown
ENSCJAT00000010717	ENSCJAG00000005512	unknown
ENSCJAT00000010767	ENSCJAG00000005512	unknown
ENSCJAT00000010785	ENSCJAG00000005512	unknown
ENSCJAT00000041195	ENSCJAG00000020962	E2F2
ENSCJAT00000041166	ENSCJAG00000020934	TCEB3
ENSCJAT00000059322	ENSCJAG00000033442	CNR2
ENSCJAT00000041036	ENSCJAG00000020880	MYOM3
ENSCJAT00000040979	ENSCJAG00000020848	NIPAL3
ENSCJAT00000039667	ENSCJAG00000020848	NIPAL3
ENSCJAT00000052982	ENSCJAG00000018575	TMEM50A
ENSCJAT00000036416	ENSCJAG00000018575	TMEM50A
ENSCJAT00000036501	ENSCJAG00000018582	unknown
ENSCJAT00000036578	ENSCJAG00000018625	TMEM57
ENSCJAT00000019578	ENSCJAG00000010081	FAM46B
ENSCJAT00000018055	ENSCJAG00000009319	IFI6
ENSCJAT00000055216	ENSCJAG00000009319	IFI6
ENSCJAT00000018277	ENSCJAG00000009406	PPP1R8
ENSCJAT00000060474	ENSCJAG00000009406	PPP1R8
ENSCJAT00000018256	ENSCJAG00000009406	PPP1R8
ENSCJAT00000018283	ENSCJAG00000009406	PPP1R8
ENSCJAT00000062983	ENSCJAG00000009490	SMPDL3B
ENSCJAT00000018386	ENSCJAG00000009490	SMPDL3B
ENSCJAT00000062833	ENSCJAG00000009490	SMPDL3B
ENSCJAT00000018739	ENSCJAG00000009642	PHACTR4
ENSCJAT00000019031	ENSCJAG00000009804	YTHDF2
ENSCJAT00000019009	ENSCJAG00000009804	YTHDF2
ENSCJAT00000062070	ENSCJAG00000009804	YTHDF2
ENSCJAT00000038919	ENSCJAG00000009804	YTHDF2
ENSCJAT00000019416	ENSCJAG00000009994	MECR
ENSCJAT00000019449	ENSCJAG00000009994	MECR

ENSCJAT00000011691	ENSCJAG00000006019	SDC3
ENSCJAT00000011681	ENSCJAG00000006019	SDC3
ENSCJAT00000012770	ENSCJAG00000006539	PTP4A2
ENSCJAT00000012889	ENSCJAG00000006580	unknown
ENSCJAT00000012877	ENSCJAG00000006580	unknown
ENSCJAT00000002057	ENSCJAG00000001105	unknown
ENSCJAT00000002045	ENSCJAG00000001105	unknown
ENSCJAT00000002335	ENSCJAG00000001247	ZSCAN20
ENSCJAT00000002558	ENSCJAG00000001370	C1orf94
ENSCJAT00000002566	ENSCJAG00000001380	GJB3
ENSCJAT00000002620	ENSCJAG00000001408	unknown
ENSCJAT00000002694	ENSCJAG00000001431	SFPQ
ENSCJAT00000054596	ENSCJAG00000001719	ADPRHL2
ENSCJAT00000060020	ENSCJAG00000001719	ADPRHL2
ENSCJAT00000003245	ENSCJAG00000001719	ADPRHL2
ENSCJAT00000003248	ENSCJAG00000001719	ADPRHL2
ENSCJAT00000003259	ENSCJAG00000001730	COL8A2
ENSCJAT00000003256	ENSCJAG00000001730	COL8A2
ENSCJAT00000003442	ENSCJAG00000001818	STK40
ENSCJAT00000003484	ENSCJAG00000001826	OSCP1
ENSCJAT00000003487	ENSCJAG00000001826	OSCP1
ENSCJAT00000057233	ENSCJAG00000001963	SNIP1
ENSCJAT00000003756	ENSCJAG00000001963	SNIP1
ENSCJAT00000055741	ENSCJAG00000001963	SNIP1
ENSCJAT00000003760	ENSCJAG00000001976	DNALI1
ENSCJAT00000003788	ENSCJAG00000001980	unknown
ENSCJAT00000061972	ENSCJAG00000001980	unknown
ENSCJAT00000003802	ENSCJAG00000001998	RSPO1
ENSCJAT00000003808	ENSCJAG00000001998	RSPO1
ENSCJAT00000056861	ENSCJAG00000001998	RSPO1
ENSCJAT00000003829	ENSCJAG00000002008	CDCA8
ENSCJAT00000003832	ENSCJAG00000002008	CDCA8
ENSCJAT00000055222	ENSCJAG00000002008	CDCA8
ENSCJAT00000003820	ENSCJAG00000002008	CDCA8
ENSCJAT00000004332	ENSCJAG00000036558	unknown
ENSCJAT00000033526	ENSCJAG00000017243	unknown
ENSCJAT00000064189	ENSCJAG00000038041	unknown
ENSCJAT00000004407	ENSCJAG00000002317	unknown
ENSCJAT00000059675	ENSCJAG00000002323	MACF1
ENSCJAT00000004807	ENSCJAG00000002509	unknown
ENSCJAT00000004892	ENSCJAG00000002546	unknown
ENSCJAT00000005555	ENSCJAG00000002851	COL9A2
ENSCJAT00000055517	ENSCJAG00000002961	EXO5
ENSCJAT00000005653	ENSCJAG00000002961	EXO5
ENSCJAT00000005787	ENSCJAG00000003003	CTPS1
ENSCJAT00000060013	ENSCJAG00000003003	CTPS1
ENSCJAT00000061051	ENSCJAG00000003003	CTPS1
ENSCJAT00000005966	ENSCJAG00000003111	ZMYND12
ENSCJAT00000006229	ENSCJAG00000003249	CCDC23
ENSCJAT00000006268	ENSCJAG00000003267	ZNF691
ENSCJAT00000006257	ENSCJAG00000003267	ZNF691
ENSCJAT00000006346	ENSCJAG00000003315	FAM183A
ENSCJAT00000006407	ENSCJAG00000003341	unknown
ENSCJAT00000006536	ENSCJAG00000003389	MPL
ENSCJAT00000006686	ENSCJAG00000003469	ELOVL1
ENSCJAT00000055460	ENSCJAG00000003469	ELOVL1
ENSCJAT00000006702	ENSCJAG00000003469	ELOVL1
ENSCJAT00000032226	ENSCJAG00000003517	unknown
ENSCJAT00000033771	ENSCJAG00000003578	PTPRF

ENSCJAT00000007513	ENSCJAG00000003910	KLF17
ENSCJAT00000007800	ENSCJAG00000004057	RNF220
ENSCJAT00000008383	ENSCJAG00000004303	PTCH2
ENSCJAT00000026620	ENSCJAG00000013641	NASP
ENSCJAT00000026613	ENSCJAG00000013641	NASP
ENSCJAT00000026630	ENSCJAG00000013641	NASP
ENSCJAT00000026640	ENSCJAG00000013641	NASP
ENSCJAT00000041273	ENSCJAG00000020964	MAST2
ENSCJAT00000041441	ENSCJAG00000021078	LURAP1
ENSCJAT00000041496	ENSCJAG00000021080	RAD54L
ENSCJAT00000041480	ENSCJAG00000021080	RAD54L
ENSCJAT00000041491	ENSCJAG00000021080	RAD54L
ENSCJAT00000041585	ENSCJAG00000021141	DMBX1
ENSCJAT00000041617	ENSCJAG00000021159	ATPAF1
ENSCJAT00000041621	ENSCJAG00000021159	ATPAF1
ENSCJAT00000041618	ENSCJAG00000021159	ATPAF1
ENSCJAT00000041738	ENSCJAG00000021204	CYP4Z1
ENSCJAT00000041732	ENSCJAG00000021204	CYP4Z1
ENSCJAT00000041736	ENSCJAG00000021204	CYP4Z1
ENSCJAT00000042405	ENSCJAG00000021568	RNF11
ENSCJAT00000060196	ENSCJAG00000021578	NRD1
ENSCJAT00000063035	ENSCJAG00000021583	ZYG11B
ENSCJAT00000042437	ENSCJAG00000021586	GPX7
ENSCJAT00000042454	ENSCJAG00000021594	PRPF38A
ENSCJAT00000042455	ENSCJAG00000021594	PRPF38A
ENSCJAT00000060658	ENSCJAG00000021597	ZFYVE9
ENSCJAT00000008501	ENSCJAG00000004441	PGAM1
ENSCJAT00000015461	ENSCJAG00000004441	PGAM1
ENSCJAT00000008595	ENSCJAG00000004449	PODN
ENSCJAT00000008589	ENSCJAG00000004449	PODN
ENSCJAT00000008575	ENSCJAG00000004449	PODN
ENSCJAT00000008579	ENSCJAG00000004449	PODN
ENSCJAT00000008577	ENSCJAG00000004449	PODN
ENSCJAT00000009216	ENSCJAG00000004707	NDC1
ENSCJAT00000009264	ENSCJAG00000004707	NDC1
ENSCJAT00000058670	ENSCJAG00000004707	NDC1
ENSCJAT00000009299	ENSCJAG00000004707	NDC1
ENSCJAT00000009277	ENSCJAG00000004707	NDC1
ENSCJAT00000009592	ENSCJAG00000004912	TMEM59
ENSCJAT00000009594	ENSCJAG00000004912	TMEM59
ENSCJAT00000053673	ENSCJAG00000015653	DHCR24
ENSCJAT00000062410	ENSCJAG00000015653	DHCR24
ENSCJAT00000030473	ENSCJAG00000015653	DHCR24
ENSCJAT00000030879	ENSCJAG00000015739	USP24
ENSCJAT00000030872	ENSCJAG00000015739	USP24
ENSCJAT00000030717	ENSCJAG00000015739	USP24
ENSCJAT00000030902	ENSCJAG00000015880	PPAP2B
ENSCJAT00000030929	ENSCJAG00000015889	PRKAA2
ENSCJAT00000030984	ENSCJAG00000015926	C8A
ENSCJAT00000030981	ENSCJAG00000015926	C8A
ENSCJAT00000059188	ENSCJAG00000016020	MYSM1
ENSCJAT00000031180	ENSCJAG00000016020	MYSM1
ENSCJAT00000056654	ENSCJAG00000016077	HOOK1
ENSCJAT00000031355	ENSCJAG00000016077	HOOK1
ENSCJAT00000031345	ENSCJAG00000016077	HOOK1
ENSCJAT00000031422	ENSCJAG00000016137	C1orf87
ENSCJAT00000003992	ENSCJAG00000031592	unknown
ENSCJAT00000032145	ENSCJAG00000016521	ALG6
ENSCJAT00000033480	ENSCJAG00000017222	TCTEX1D1

ENSCJAT00000034225	ENSCJAG00000017542	RPE65
ENSCJAT00000012446	ENSCJAG00000006400	unknown
ENSCJAT00000013519	ENSCJAG00000006894	ZZZ3
ENSCJAT00000013511	ENSCJAG00000006894	ZZZ3
ENSCJAT00000013985	ENSCJAG00000007062	FUBP1
ENSCJAT00000013976	ENSCJAG00000007062	FUBP1
ENSCJAT00000013987	ENSCJAG00000007062	FUBP1
ENSCJAT00000013946	ENSCJAG00000007062	FUBP1
ENSCJAT00000013922	ENSCJAG00000007062	FUBP1
ENSCJAT00000014024	ENSCJAG00000007162	PTGFR
ENSCJAT00000014551	ENSCJAG00000007425	unknown
ENSCJAT00000014571	ENSCJAG00000007432	unknown
ENSCJAT00000013624	ENSCJAG00000006895	WDR63
ENSCJAT00000013631	ENSCJAG00000006895	WDR63
ENSCJAT00000013590	ENSCJAG00000006895	WDR63
ENSCJAT00000057055	ENSCJAG00000006895	WDR63
ENSCJAT00000013739	ENSCJAG00000007022	C1orf52
ENSCJAT00000013753	ENSCJAG00000007022	C1orf52
ENSCJAT00000013796	ENSCJAG00000007041	ZNHIT6
ENSCJAT00000014276	ENSCJAG00000007251	CLCA2
ENSCJAT00000014282	ENSCJAG00000007251	CLCA2
ENSCJAT00000052897	ENSCJAG00000007335	SH3GLB1
ENSCJAT00000056165	ENSCJAG00000007335	SH3GLB1
ENSCJAT00000014421	ENSCJAG00000007335	SH3GLB1
ENSCJAT00000014415	ENSCJAG00000007335	SH3GLB1
ENSCJAT00000014617	ENSCJAG00000007409	PKN2
ENSCJAT00000014663	ENSCJAG00000007469	GTF2B
ENSCJAT00000014659	ENSCJAG00000007469	GTF2B
ENSCJAT00000055992	ENSCJAG00000007493	CCBL2
ENSCJAT00000034166	ENSCJAG00000017540	KIAA1107
ENSCJAT00000034272	ENSCJAG00000017591	RPAP2
ENSCJAT00000034635	ENSCJAG00000017759	TMED5
ENSCJAT00000034629	ENSCJAG00000017759	TMED5
ENSCJAT00000034694	ENSCJAG00000017774	CCDC18
ENSCJAT00000034843	ENSCJAG00000017821	FNBP1L
ENSCJAT00000034811	ENSCJAG00000017821	FNBP1L
ENSCJAT00000034834	ENSCJAG00000017821	FNBP1L
ENSCJAT00000034928	ENSCJAG00000017899	GCLM
ENSCJAT00000056734	ENSCJAG00000017910	ABCA4
ENSCJAT00000032599	ENSCJAG00000017910	ABCA4
ENSCJAT00000035499	ENSCJAG00000018172	PTBP2
ENSCJAT00000035676	ENSCJAG00000018231	unknown
ENSCJAT00000035671	ENSCJAG00000018231	unknown
ENSCJAT00000035663	ENSCJAG00000018231	unknown
ENSCJAT00000035751	ENSCJAG00000018277	PALMD
ENSCJAT00000035762	ENSCJAG00000018284	FRRS1
ENSCJAT00000006563	ENSCJAG00000003398	RTCA
ENSCJAT00000013259	ENSCJAG00000003398	RTCA
ENSCJAT00000006745	ENSCJAG00000003508	SLC30A7
ENSCJAT00000017430	ENSCJAG00000008998	OLFM3
ENSCJAT00000017476	ENSCJAG00000008998	OLFM3
ENSCJAT00000052834	ENSCJAG00000031625	unknown
ENSCJAT00000017163	ENSCJAG00000031625	unknown
ENSCJAT00000053942	ENSCJAG00000002510	PRPF38B
ENSCJAT00000004856	ENSCJAG00000002532	FNDC7
ENSCJAT00000005431	ENSCJAG00000002784	SARS
ENSCJAT00000005430	ENSCJAG00000002784	SARS
ENSCJAT00000005649	ENSCJAG00000002949	MYBPHL
ENSCJAT00000063428	ENSCJAG00000003004	PSMA5

ENSCJAT00000005906	ENSCJAG00000003071	GNAT2
ENSCJAT00000006436	ENSCJAG00000003306	STRIP1
ENSCJAT00000006441	ENSCJAG00000003306	STRIP1
ENSCJAT00000025559	ENSCJAG00000013162	LRIF1
ENSCJAT00000054950	ENSCJAG00000013162	LRIF1
ENSCJAT00000060234	ENSCJAG00000013657	RHOC
ENSCJAT00000060820	ENSCJAG00000013657	RHOC
ENSCJAT00000026586	ENSCJAG00000013657	RHOC
ENSCJAT00000058388	ENSCJAG00000013657	RHOC
ENSCJAT00000023476	ENSCJAG00000012063	RSBN1
ENSCJAT00000053982	ENSCJAG00000012063	RSBN1
ENSCJAT00000023450	ENSCJAG00000012063	RSBN1
ENSCJAT00000054085	ENSCJAG00000012107	PTPN22
ENSCJAT00000053565	ENSCJAG00000012107	PTPN22
ENSCJAT00000055639	ENSCJAG00000012107	PTPN22
ENSCJAT00000061975	ENSCJAG00000012107	PTPN22
ENSCJAT00000025829	ENSCJAG00000012221	HIPK1
ENSCJAT00000023814	ENSCJAG00000012221	HIPK1
ENSCJAT00000025860	ENSCJAG00000012221	HIPK1
ENSCJAT00000025864	ENSCJAG00000012221	HIPK1
ENSCJAT00000058426	ENSCJAG00000012333	SYT6
ENSCJAT00000065568	ENSCJAG00000036599	unknown
ENSCJAT00000011554	ENSCJAG00000005944	CD101
ENSCJAT00000011449	ENSCJAG00000005897	TRIM45
ENSCJAT00000011453	ENSCJAG00000005897	TRIM45
ENSCJAT00000011336	ENSCJAG00000005856	FAM46C
ENSCJAT00000061917	ENSCJAG00000005653	WARS2
ENSCJAT00000057151	ENSCJAG00000005653	WARS2
ENSCJAT00000010930	ENSCJAG00000005653	WARS2
ENSCJAT00000010918	ENSCJAG00000005623	HAO2
ENSCJAT00000007682	ENSCJAG00000035427	unknown
ENSCJAT00000007683	ENSCJAG00000035427	unknown
ENSCJAT00000008439	ENSCJAG00000004402	GRPR
ENSCJAT00000008443	ENSCJAG00000004410	MAGEB17
ENSCJAT00000010868	ENSCJAG00000005571	GPR64
ENSCJAT00000010925	ENSCJAG00000005571	GPR64
ENSCJAT00000010938	ENSCJAG00000005571	GPR64
ENSCJAT00000010965	ENSCJAG00000005571	GPR64
ENSCJAT00000010498	ENSCJAG00000005439	CXorf23
ENSCJAT00000010461	ENSCJAG00000005383	MAP7D2
ENSCJAT00000010466	ENSCJAG00000005383	MAP7D2
ENSCJAT00000009958	ENSCJAG00000005121	PHEX
ENSCJAT00000009861	ENSCJAG00000005108	PTCHD1
ENSCJAT00000009855	ENSCJAG00000005108	PTCHD1
ENSCJAT00000009837	ENSCJAG00000005061	PRDX4
ENSCJAT00000009831	ENSCJAG00000005061	PRDX4
ENSCJAT00000009804	ENSCJAG00000005061	PRDX4
ENSCJAT00000009094	ENSCJAG00000004737	ARX
ENSCJAT00000037188	ENSCJAG00000018959	MAGEB10
ENSCJAT00000014890	ENSCJAG00000007615	NR0B1
ENSCJAT00000014901	ENSCJAG00000007627	CXorf21
ENSCJAT00000015164	ENSCJAG00000007710	TAB3
ENSCJAT00000053306	ENSCJAG00000031923	CHDC2
ENSCJAT00000038620	ENSCJAG00000019688	CXorf30
ENSCJAT00000060264	ENSCJAG00000005168	XK
ENSCJAT00000009992	ENSCJAG00000005168	XK
ENSCJAT00000040829	ENSCJAG00000020771	RPGR
ENSCJAT00000021421	ENSCJAG00000010889	KDM6A
ENSCJAT00000034977	ENSCJAG00000011115	CXorf36

ENSCJAT00000021675	ENSCJAG00000011115	CXorf36
ENSCJAT00000061446	ENSCJAG00000011115	CXorf36
ENSCJAT00000021668	ENSCJAG00000011115	CXorf36
ENSCJAT00000023278	ENSCJAG00000011977	unknown
ENSCJAT00000023243	ENSCJAG00000011977	unknown
ENSCJAT00000023467	ENSCJAG00000012090	NDUFB11
ENSCJAT00000004562	ENSCJAG00000002376	SUV39H1
ENSCJAT00000004700	ENSCJAG00000002426	TIMM17B
ENSCJAT00000005120	ENSCJAG00000002656	GPKOW
ENSCJAT00000000802	ENSCJAG00000002945	CLCN5
ENSCJAT00000005707	ENSCJAG00000002945	CLCN5
ENSCJAT00000031006	ENSCJAG00000015936	unknown
ENSCJAT00000034105	ENSCJAG00000017462	MAGED1
ENSCJAT00000034116	ENSCJAG00000017462	MAGED1
ENSCJAT00000025609	ENSCJAG00000013184	unknown
ENSCJAT00000011350	ENSCJAG00000005850	TSPYL2
ENSCJAT00000011354	ENSCJAG00000005850	TSPYL2
ENSCJAT00000011357	ENSCJAG00000005850	TSPYL2
ENSCJAT00000034101	ENSCJAG00000007728	TRO
ENSCJAT00000034021	ENSCJAG00000007728	TRO
ENSCJAT00000054729	ENSCJAG00000007728	TRO
ENSCJAT00000015194	ENSCJAG00000007728	TRO
ENSCJAT00000032261	ENSCJAG00000016584	APEX2
ENSCJAT00000056783	ENSCJAG00000016584	APEX2
ENSCJAT00000032221	ENSCJAG00000016542	ALAS2
ENSCJAT00000032237	ENSCJAG00000016542	ALAS2
ENSCJAT00000044421	ENSCJAG00000023436	USP51
ENSCJAT00000006895	ENSCJAG00000003590	EDA2R
ENSCJAT00000006891	ENSCJAG00000003590	EDA2R
ENSCJAT00000006875	ENSCJAG00000003590	EDA2R
ENSCJAT00000006903	ENSCJAG00000003590	EDA2R
ENSCJAT00000026580	ENSCJAG00000013662	SNX12
ENSCJAT00000026591	ENSCJAG00000013662	SNX12
ENSCJAT00000026588	ENSCJAG00000013662	SNX12
ENSCJAT00000041630	ENSCJAG00000021170	ATRX
ENSCJAT00000037112	ENSCJAG00000018916	MAGT1
ENSCJAT00000041459	ENSCJAG00000021082	unknown
ENSCJAT00000041449	ENSCJAG00000021082	unknown
ENSCJAT00000041462	ENSCJAG00000021082	unknown
ENSCJAT00000052687	ENSCJAG00000021082	unknown
ENSCJAT00000018602	ENSCJAG00000009591	ITM2A
ENSCJAT00000018606	ENSCJAG00000009591	ITM2A
ENSCJAT00000052368	ENSCJAG00000000289	unknown
ENSCJAT00000020345	ENSCJAG00000010422	DACH2
ENSCJAT00000007024	ENSCJAG00000003672	unknown
ENSCJAT00000007064	ENSCJAG00000003672	unknown
ENSCJAT00000007057	ENSCJAG00000003672	unknown
ENSCJAT00000007031	ENSCJAG00000003672	unknown
ENSCJAT00000008298	ENSCJAG00000004321	SRPX2
ENSCJAT00000008745	ENSCJAG00000004565	ARL13A
ENSCJAT00000008748	ENSCJAG00000004565	ARL13A
ENSCJAT00000053750	ENSCJAG00000032260	unknown
ENSCJAT00000052574	ENSCJAG00000004865	GLA
ENSCJAT00000038675	ENSCJAG00000019686	unknown
ENSCJAT00000038672	ENSCJAG00000019686	unknown
ENSCJAT00000038669	ENSCJAG00000019686	unknown
ENSCJAT00000038694	ENSCJAG00000019686	unknown
ENSCJAT00000038687	ENSCJAG00000019686	unknown
ENSCJAT00000043866	ENSCJAG00000022881	RAB40A

ENSCJAT00000053342	ENSCJAG00000003394	MORF4L2
ENSCJAT00000006507	ENSCJAG00000003394	MORF4L2
ENSCJAT00000060076	ENSCJAG00000003394	MORF4L2
ENSCJAT00000004364	ENSCJAG00000003394	MORF4L2
ENSCJAT00000016075	ENSCJAG00000008241	FAM199X
ENSCJAT00000063332	ENSCJAG00000007983	MORC4
ENSCJAT00000015504	ENSCJAG00000007942	NUP62CL
ENSCJAT00000054332	ENSCJAG00000007942	NUP62CL
ENSCJAT00000044419	ENSCJAG00000023434	KCNE1L
ENSCJAT00000014418	ENSCJAG00000007315	ACSL4
ENSCJAT00000032691	ENSCJAG00000001982	PLS3
ENSCJAT00000010108	ENSCJAG00000005231	ZCCHC12
ENSCJAT00000009959	ENSCJAG00000005166	PGRMC1
ENSCJAT00000059433	ENSCJAG00000005166	PGRMC1
ENSCJAT00000009849	ENSCJAG00000005098	UBE2A
ENSCJAT00000009846	ENSCJAG00000005098	UBE2A
ENSCJAT00000053478	ENSCJAG00000005098	UBE2A
ENSCJAT00000005243	ENSCJAG00000002719	XPNPEP2
ENSCJAT00000061592	ENSCJAG00000002703	SASH3
ENSCJAT00000056735	ENSCJAG00000002703	SASH3
ENSCJAT00000052837	ENSCJAG00000002703	SASH3
ENSCJAT00000005192	ENSCJAG00000002703	SASH3
ENSCJAT00000060159	ENSCJAG00000014442	ARHGAP36
ENSCJAT00000028103	ENSCJAG00000014442	ARHGAP36
ENSCJAT00000040060	ENSCJAG00000020403	CT55
ENSCJAT00000040062	ENSCJAG00000020403	CT55
ENSCJAT00000040059	ENSCJAG00000020403	CT55
ENSCJAT00000028216	ENSCJAG00000014469	DDX26B
ENSCJAT00000055671	ENSCJAG00000014469	DDX26B
ENSCJAT00000060382	ENSCJAG00000003818	COL1A1
ENSCJAT00000027436	ENSCJAG00000003630	ARHGEF6
ENSCJAT00000006984	ENSCJAG00000003630	ARHGEF6
ENSCJAT00000007014	ENSCJAG00000003630	ARHGEF6
ENSCJAT00000019586	ENSCJAG00000010089	CXorf66
ENSCJAT00000061016	ENSCJAG00000003848	GABRE
ENSCJAT00000007413	ENSCJAG00000003848	GABRE
ENSCJAT00000022077	ENSCJAG00000011348	BCAP31
ENSCJAT00000058444	ENSCJAG00000011348	BCAP31
ENSCJAT00000044097	ENSCJAG00000023112	TMEM187
ENSCJAT00000008355	ENSCJAG00000004362	CLIC2
ENSCJAT00000008348	ENSCJAG00000004362	CLIC2
ENSCJAT00000044316	ENSCJAG00000023331	FOXB1
ENSCJAT00000007645	ENSCJAG00000003977	ICE2
ENSCJAT00000008524	ENSCJAG00000004355	TPM1
ENSCJAT00000008499	ENSCJAG00000004355	TPM1
ENSCJAT00000008539	ENSCJAG00000004355	TPM1
ENSCJAT00000008527	ENSCJAG00000004355	TPM1
ENSCJAT00000063431	ENSCJAG00000004355	TPM1
ENSCJAT00000009581	ENSCJAG00000004856	SNX1
ENSCJAT00000009600	ENSCJAG00000004856	SNX1
ENSCJAT00000010044	ENSCJAG00000005200	unknown
ENSCJAT00000010192	ENSCJAG00000033960	PLEKHO2
ENSCJAT00000010159	ENSCJAG00000033960	PLEKHO2
ENSCJAT00000040849	ENSCJAG00000033960	PLEKHO2
ENSCJAT00000010200	ENSCJAG00000005280	unknown
ENSCJAT00000010189	ENSCJAG00000031989	ANKDD1A
ENSCJAT00000013354	ENSCJAG00000006848	MTFMT
ENSCJAT00000013379	ENSCJAG00000006852	SLC51B
ENSCJAT00000003999	ENSCJAG00000002088	SMAD3

ENSCJAT00000016668	ENSCJAG00000008560	SPESP1
ENSCJAT00000036373	ENSCJAG00000018552	NR2E3
ENSCJAT00000036388	ENSCJAG00000018552	NR2E3
ENSCJAT00000036385	ENSCJAG00000018552	NR2E3
ENSCJAT00000036251	ENSCJAG00000018473	PKM
ENSCJAT00000036295	ENSCJAG00000018473	PKM
ENSCJAT00000036292	ENSCJAG00000018473	PKM
ENSCJAT00000036272	ENSCJAG00000018473	PKM
ENSCJAT00000036279	ENSCJAG00000018473	PKM
ENSCJAT00000002220	ENSCJAG00000018355	unknown
ENSCJAT00000035963	ENSCJAG00000018355	unknown
ENSCJAT00000035949	ENSCJAG00000018355	unknown
ENSCJAT00000035979	ENSCJAG00000018355	unknown
ENSCJAT00000035974	ENSCJAG00000018355	unknown
ENSCJAT00000035600	ENSCJAG00000018181	BBS4
ENSCJAT00000035592	ENSCJAG00000018181	BBS4
ENSCJAT00000035500	ENSCJAG00000018161	ADPGK
ENSCJAT00000035523	ENSCJAG00000018161	ADPGK
ENSCJAT00000055513	ENSCJAG00000018161	ADPGK
ENSCJAT00000035517	ENSCJAG00000018161	ADPGK
ENSCJAT00000035505	ENSCJAG00000018161	ADPGK
ENSCJAT00000053464	ENSCJAG00000018044	NEO1
ENSCJAT00000035199	ENSCJAG00000018009	NPTN
ENSCJAT00000035198	ENSCJAG00000018009	NPTN
ENSCJAT00000035176	ENSCJAG00000018009	NPTN
ENSCJAT00000035056	ENSCJAG00000017949	LOXL1
ENSCJAT00000035051	ENSCJAG00000017949	LOXL1
ENSCJAT00000035045	ENSCJAG00000017949	LOXL1
ENSCJAT00000052122	ENSCJAG00000017807	SEMA7A
ENSCJAT00000034734	ENSCJAG00000017807	SEMA7A
ENSCJAT00000034653	ENSCJAG00000017758	ARID3B
ENSCJAT00000034646	ENSCJAG00000017758	ARID3B
ENSCJAT00000023933	ENSCJAG00000012359	unknown
ENSCJAT00000023917	ENSCJAG00000012331	FBXO22
ENSCJAT00000059666	ENSCJAG00000012331	FBXO22
ENSCJAT00000023926	ENSCJAG00000012331	FBXO22
ENSCJAT00000003730	ENSCJAG00000001935	CDAN1
ENSCJAT00000003720	ENSCJAG00000001935	CDAN1
ENSCJAT00000005742	ENSCJAG00000002999	TMEM62
ENSCJAT00000005623	ENSCJAG00000037326	EPB42
ENSCJAT00000053242	ENSCJAG00000002654	STRC
ENSCJAT00000005122	ENSCJAG00000002654	STRC
ENSCJAT00000055437	ENSCJAG00000002654	STRC
ENSCJAT00000005104	ENSCJAG00000002654	STRC
ENSCJAT00000053129	ENSCJAG00000002654	STRC
ENSCJAT00000043173	ENSCJAG00000022188	unknown
ENSCJAT00000004511	ENSCJAG00000002369	TRIM69
ENSCJAT00000004506	ENSCJAG00000002369	TRIM69
ENSCJAT00000003945	ENSCJAG00000002067	SPATA5L1
ENSCJAT00000003916	ENSCJAG00000002049	SLC30A4
ENSCJAT00000043170	ENSCJAG00000037327	CTXN2
ENSCJAT00000003138	ENSCJAG00000001637	SECISBP2L
ENSCJAT00000003143	ENSCJAG00000001637	SECISBP2L
ENSCJAT00000003134	ENSCJAG00000001637	SECISBP2L
ENSCJAT00000003128	ENSCJAG00000001637	SECISBP2L
ENSCJAT00000060306	ENSCJAG00000001585	COPS2
ENSCJAT00000003071	ENSCJAG00000001585	COPS2
ENSCJAT00000052921	ENSCJAG00000001585	COPS2
ENSCJAT00000002970	ENSCJAG00000001572	FGF7

ENSCJAT00000002940	ENSCJAG00000001548	DTWD1
ENSCJAT00000002936	ENSCJAG00000001548	DTWD1
ENSCJAT00000002928	ENSCJAG00000001548	DTWD1
ENSCJAT00000002930	ENSCJAG00000001548	DTWD1
ENSCJAT00000002914	ENSCJAG00000001548	DTWD1
ENSCJAT00000002825	ENSCJAG00000001489	HDC
ENSCJAT00000002820	ENSCJAG00000001489	HDC
ENSCJAT00000054495	ENSCJAG00000001217	AP4E1
ENSCJAT00000002156	ENSCJAG00000001160	GLDN
ENSCJAT00000002142	ENSCJAG00000001160	GLDN
ENSCJAT00000028381	ENSCJAG00000001160	GLDN
ENSCJAT00000052906	ENSCJAG00000001016	SCG3
ENSCJAT00000001874	ENSCJAG00000001016	SCG3
ENSCJAT00000001828	ENSCJAG00000001004	LYSMD2
ENSCJAT00000055973	ENSCJAG00000001004	LYSMD2
ENSCJAT00000001831	ENSCJAG00000001004	LYSMD2
ENSCJAT00000001817	ENSCJAG00000000991	TMOD2
ENSCJAT00000001814	ENSCJAG00000000991	TMOD2
ENSCJAT00000001733	ENSCJAG00000000947	GNB5
ENSCJAT00000001709	ENSCJAG00000000926	MYO5C
ENSCJAT00000060801	ENSCJAG00000000823	WDR72
ENSCJAT00000057169	ENSCJAG00000002283	PIGB
ENSCJAT00000004249	ENSCJAG00000002242	PYGO1
ENSCJAT00000004261	ENSCJAG00000002242	PYGO1
ENSCJAT00000004105	ENSCJAG00000002163	RFX7
ENSCJAT00000040713	ENSCJAG00000020713	RPAP1
ENSCJAT00000040762	ENSCJAG00000020743	ITPKA
ENSCJAT00000040764	ENSCJAG00000020743	ITPKA
ENSCJAT00000040788	ENSCJAG00000020755	NUSAP1
ENSCJAT00000040936	ENSCJAG00000020831	CASC5
ENSCJAT00000040976	ENSCJAG00000020852	IVD
ENSCJAT00000041032	ENSCJAG00000020881	ANKRD63
ENSCJAT00000041074	ENSCJAG00000036373	BUB1B
ENSCJAT00000041060	ENSCJAG00000036373	BUB1B
ENSCJAT00000041299	ENSCJAG00000021006	DPH6
ENSCJAT00000041410	ENSCJAG00000021064	GREM1
ENSCJAT00000063788	ENSCJAG00000021064	GREM1
ENSCJAT00000041420	ENSCJAG00000021067	FMN1
ENSCJAT00000041493	ENSCJAG00000021103	EMC7
ENSCJAT00000041511	ENSCJAG00000021110	unknown
ENSCJAT00000056366	ENSCJAG00000021110	unknown
ENSCJAT00000041629	ENSCJAG00000021166	NUTM1
ENSCJAT00000059089	ENSCJAG00000009650	RNASE4
ENSCJAT00000018698	ENSCJAG00000009650	RNASE4
ENSCJAT00000018262	ENSCJAG00000009398	ARHGEF40
ENSCJAT00000062889	ENSCJAG00000008981	RAB2B
ENSCJAT00000017406	ENSCJAG00000008981	RAB2B
ENSCJAT00000017311	ENSCJAG00000008893	METTL3
ENSCJAT00000061004	ENSCJAG00000008714	TRDC
ENSCJAT00000052522	ENSCJAG00000008714	TRDC
ENSCJAT00000016490	ENSCJAG00000008409	PRMT5
ENSCJAT00000060513	ENSCJAG00000008409	PRMT5
ENSCJAT00000062284	ENSCJAG00000008409	PRMT5
ENSCJAT00000000504	ENSCJAG00000000278	unknown
ENSCJAT00000014730	ENSCJAG00000007523	ZFHX2
ENSCJAT00000014718	ENSCJAG00000007486	AP1G2
ENSCJAT00000014704	ENSCJAG00000007486	AP1G2
ENSCJAT00000014608	ENSCJAG00000007462	unknown
ENSCJAT00000014580	ENSCJAG00000007382	LRRC16B

ENSCJAT00000014526	ENSCJAG00000007382	LRRC16B
ENSCJAT00000013719	ENSCJAG00000006937	IPO4
ENSCJAT00000013655	ENSCJAG00000006937	IPO4
ENSCJAT00000012470	ENSCJAG00000006414	NFATC4
ENSCJAT00000012561	ENSCJAG00000006414	NFATC4
ENSCJAT00000012544	ENSCJAG00000006414	NFATC4
ENSCJAT00000005099	ENSCJAG00000006414	NFATC4
ENSCJAT00000012553	ENSCJAG00000006414	NFATC4
ENSCJAT00000012449	ENSCJAG00000006385	NYNRIN
ENSCJAT00000011892	ENSCJAG00000006106	G2E3
ENSCJAT00000011965	ENSCJAG00000006106	G2E3
ENSCJAT00000010921	ENSCJAG00000005631	HECTD1
ENSCJAT00000010837	ENSCJAG00000005593	HEATR5A
ENSCJAT00000061209	ENSCJAG00000005424	EGLN3
ENSCJAT00000010478	ENSCJAG00000005424	EGLN3
ENSCJAT00000010470	ENSCJAG00000005424	EGLN3
ENSCJAT00000010447	ENSCJAG00000005416	KPNA2
ENSCJAT00000013640	ENSCJAG00000006938	MBIP
ENSCJAT00000013609	ENSCJAG00000006938	MBIP
ENSCJAT00000031744	ENSCJAG00000016314	TRAPPC6B
ENSCJAT00000031745	ENSCJAG00000016314	TRAPPC6B
ENSCJAT00000031741	ENSCJAG00000016314	TRAPPC6B
ENSCJAT00000031596	ENSCJAG00000016105	unknown
ENSCJAT00000031575	ENSCJAG00000016105	unknown
ENSCJAT00000031574	ENSCJAG00000016105	unknown
ENSCJAT00000059706	ENSCJAG00000016105	unknown
ENSCJAT00000031561	ENSCJAG00000016105	unknown
ENSCJAT00000062552	ENSCJAG00000016105	unknown
ENSCJAT00000031568	ENSCJAG00000016105	unknown
ENSCJAT00000031273	ENSCJAG00000016071	C14orf28
ENSCJAT00000031251	ENSCJAG00000016063	KLHL28
ENSCJAT00000031093	ENSCJAG00000015972	unknown
ENSCJAT00000044428	ENSCJAG00000034412	unknown
ENSCJAT00000034049	ENSCJAG00000017431	POLE2
ENSCJAT00000054325	ENSCJAG00000017431	POLE2
ENSCJAT00000034047	ENSCJAG00000017431	POLE2
ENSCJAT00000011808	ENSCJAG00000006021	KLHDC2
ENSCJAT00000012029	ENSCJAG00000006093	NEMF
ENSCJAT00000012053	ENSCJAG00000006093	NEMF
ENSCJAT00000011983	ENSCJAG00000006093	NEMF
ENSCJAT00000012274	ENSCJAG00000006308	L2HGDH
ENSCJAT00000012280	ENSCJAG00000006308	L2HGDH
ENSCJAT00000012304	ENSCJAG00000006325	ATP5S
ENSCJAT00000012581	ENSCJAG00000006357	MAP4K5
ENSCJAT00000012857	ENSCJAG00000006608	unknown
ENSCJAT00000013441	ENSCJAG00000006884	TRIM9
ENSCJAT00000013444	ENSCJAG00000006884	TRIM9
ENSCJAT00000013482	ENSCJAG00000006907	FRMD6
ENSCJAT00000013570	ENSCJAG00000006926	C14orf166
ENSCJAT00000013943	ENSCJAG00000007097	ERO1L
ENSCJAT00000014335	ENSCJAG00000007306	CNIH1
ENSCJAT00000014377	ENSCJAG00000007328	CGRRF1
ENSCJAT00000035577	ENSCJAG00000018205	GCH1
ENSCJAT00000062901	ENSCJAG00000018208	WDHD1
ENSCJAT00000035761	ENSCJAG00000018289	SOCS4
ENSCJAT00000035770	ENSCJAG00000018292	unknown
ENSCJAT00000035936	ENSCJAG00000018372	TBPL2
ENSCJAT00000036359	ENSCJAG00000018523	TMEM260
ENSCJAT00000036572	ENSCJAG00000018629	NAA30

ENSCJAT00000036567	ENSCJAG00000018629	NAA30
ENSCJAT00000036631	ENSCJAG00000018672	C14orf37
ENSCJAT00000036629	ENSCJAG00000018672	C14orf37
ENSCJAT00000063673	ENSCJAG00000018764	ARID4A
ENSCJAT00000053231	ENSCJAG00000018764	ARID4A
ENSCJAT00000036865	ENSCJAG00000018764	ARID4A
ENSCJAT00000052191	ENSCJAG00000018764	ARID4A
ENSCJAT00000036873	ENSCJAG00000018798	TIMM9
ENSCJAT00000037189	ENSCJAG00000018962	GPR135
ENSCJAT00000037201	ENSCJAG00000018964	L3HYPDH
ENSCJAT00000037378	ENSCJAG00000019055	PCNXL4
ENSCJAT00000037381	ENSCJAG00000019055	PCNXL4
ENSCJAT00000037753	ENSCJAG00000019255	KCNH5
ENSCJAT00000037804	ENSCJAG00000019287	RHOJ
ENSCJAT00000021389	ENSCJAG00000019287	RHOJ
ENSCJAT00000038276	ENSCJAG00000019502	ESR2
ENSCJAT00000059246	ENSCJAG00000019526	MTHFD1
ENSCJAT00000038376	ENSCJAG00000019526	MTHFD1
ENSCJAT00000060362	ENSCJAG00000019591	ZBTB1
ENSCJAT00000038426	ENSCJAG00000019591	ZBTB1
ENSCJAT00000007306	ENSCJAG00000003756	FUT8
ENSCJAT00000030150	ENSCJAG00000015489	FAM71D
ENSCJAT00000030161	ENSCJAG00000015489	FAM71D
ENSCJAT00000030143	ENSCJAG00000015489	FAM71D
ENSCJAT00000030018	ENSCJAG00000015396	ATP6V1D
ENSCJAT00000056636	ENSCJAG00000015396	ATP6V1D
ENSCJAT00000029874	ENSCJAG00000015358	TMEM229B
ENSCJAT00000029743	ENSCJAG00000015277	ARG2
ENSCJAT00000029708	ENSCJAG00000015232	RDH11
ENSCJAT00000029700	ENSCJAG00000015232	RDH11
ENSCJAT00000029436	ENSCJAG00000015109	unknown
ENSCJAT00000029061	ENSCJAG00000014931	PLEKHD1
ENSCJAT00000028958	ENSCJAG00000014870	SLC10A1
ENSCJAT00000028963	ENSCJAG00000014870	SLC10A1
ENSCJAT00000022286	ENSCJAG00000014823	SLC8A3
ENSCJAT00000054568	ENSCJAG00000014823	SLC8A3
ENSCJAT00000028795	ENSCJAG00000014786	TTC9
ENSCJAT00000028798	ENSCJAG00000014786	TTC9
ENSCJAT00000028792	ENSCJAG00000014786	TTC9
ENSCJAT00000028251	ENSCJAG00000014522	DPF3
ENSCJAT00000028260	ENSCJAG00000014522	DPF3
ENSCJAT00000028165	ENSCJAG00000014441	unknown
ENSCJAT00000061093	ENSCJAG00000014441	unknown
ENSCJAT00000053030	ENSCJAG00000014441	unknown
ENSCJAT00000028054	ENSCJAG00000014387	PSEN1
ENSCJAT00000019261	ENSCJAG00000009922	DNAL1
ENSCJAT00000039985	ENSCJAG00000020363	FAM161B
ENSCJAT00000039965	ENSCJAG00000020308	ZNF410
ENSCJAT00000040112	ENSCJAG00000020412	ALDH6A1
ENSCJAT00000040176	ENSCJAG00000020468	VRTN
ENSCJAT00000040193	ENSCJAG00000020476	NPC2
ENSCJAT00000063274	ENSCJAG00000020476	NPC2
ENSCJAT00000061043	ENSCJAG00000020476	NPC2
ENSCJAT00000040191	ENSCJAG00000020476	NPC2
ENSCJAT00000056330	ENSCJAG00000020497	AREL1
ENSCJAT00000040281	ENSCJAG00000020497	AREL1
ENSCJAT00000053765	ENSCJAG00000020497	AREL1
ENSCJAT00000040234	ENSCJAG00000037246	unknown
ENSCJAT00000040286	ENSCJAG00000020518	FCF1

ENSCJAT00000040437	ENSCJAG00000020577	EIF2B2
ENSCJAT00000040431	ENSCJAG00000020577	EIF2B2
ENSCJAT00000040507	ENSCJAG00000020610	NEK9
ENSCJAT00000040506	ENSCJAG00000020610	NEK9
ENSCJAT00000040579	ENSCJAG00000020659	C14orf1
ENSCJAT00000040640	ENSCJAG00000020680	TGFB3
ENSCJAT00000058727	ENSCJAG00000020726	ANGEL1
ENSCJAT00000040738	ENSCJAG00000020726	ANGEL1
ENSCJAT00000040736	ENSCJAG00000020726	ANGEL1
ENSCJAT00000040729	ENSCJAG00000020726	ANGEL1
ENSCJAT00000032474	ENSCJAG00000016701	ZDHHC22
ENSCJAT00000032860	ENSCJAG00000016880	ISM2
ENSCJAT00000032849	ENSCJAG00000016880	ISM2
ENSCJAT00000032862	ENSCJAG00000016880	ISM2
ENSCJAT00000032935	ENSCJAG00000016936	ALKBH1
ENSCJAT00000033084	ENSCJAG00000016947	SNW1
ENSCJAT00000055236	ENSCJAG00000034908	unknown
ENSCJAT00000033424	ENSCJAG00000017169	GTF2A1
ENSCJAT00000033426	ENSCJAG00000017169	GTF2A1
ENSCJAT00000033406	ENSCJAG00000017169	GTF2A1
ENSCJAT00000056755	ENSCJAG00000017259	GALC
ENSCJAT00000033573	ENSCJAG00000017259	GALC
ENSCJAT00000033581	ENSCJAG00000017259	GALC
ENSCJAT00000033579	ENSCJAG00000017259	GALC
ENSCJAT00000033585	ENSCJAG00000017259	GALC
ENSCJAT00000033690	ENSCJAG00000017294	SPATA7
ENSCJAT00000033691	ENSCJAG00000017294	SPATA7
ENSCJAT00000055251	ENSCJAG00000017711	NRDE2
ENSCJAT00000034570	ENSCJAG00000017711	NRDE2
ENSCJAT00000055176	ENSCJAG00000017711	NRDE2
ENSCJAT00000034564	ENSCJAG00000017711	NRDE2
ENSCJAT00000035336	ENSCJAG00000018069	CPSF2
ENSCJAT00000035345	ENSCJAG00000018069	CPSF2
ENSCJAT00000035324	ENSCJAG00000018069	CPSF2
ENSCJAT00000035619	ENSCJAG00000018184	GOLGA5
ENSCJAT00000035607	ENSCJAG00000018184	GOLGA5
ENSCJAT00000035672	ENSCJAG00000018227	CHGA
ENSCJAT00000035660	ENSCJAG00000018227	CHGA
ENSCJAT00000059260	ENSCJAG00000009634	PRIMA1
ENSCJAT00000059078	ENSCJAG00000009634	PRIMA1
ENSCJAT00000053110	ENSCJAG00000009634	PRIMA1
ENSCJAT00000018660	ENSCJAG00000009634	PRIMA1
ENSCJAT00000019074	ENSCJAG00000009829	SERPINA5
ENSCJAT00000019065	ENSCJAG00000009829	SERPINA5
ENSCJAT00000060837	ENSCJAG00000006225	DICER1
ENSCJAT00000058986	ENSCJAG00000006225	DICER1
ENSCJAT00000059728	ENSCJAG00000006225	DICER1
ENSCJAT00000012387	ENSCJAG00000006351	SYNE3
ENSCJAT00000012381	ENSCJAG00000006351	SYNE3
ENSCJAT00000012438	ENSCJAG00000006399	BDKRB2
ENSCJAT00000013148	ENSCJAG00000006738	MA_R322_JSMB1F6EF
ENSCJAT00000013142	ENSCJAG00000006738	MA_R322_JSMB1F6EF
ENSCJAT00000013157	ENSCJAG00000006738	MA_R322_JSMB1F6EF
ENSCJAT00000006315	ENSCJAG00000003281	AMN
ENSCJAT00000006307	ENSCJAG00000003281	AMN
ENSCJAT00000058797	ENSCJAG00000017071	EXOC3L4
ENSCJAT00000033186	ENSCJAG00000017071	EXOC3L4
ENSCJAT00000033243	ENSCJAG00000017104	unknown
ENSCJAT00000033435	ENSCJAG00000017190	ZFYVE21

ENSCJAT00000033421	ENSCJAG00000017190	ZFYVE21
ENSCJAT00000033439	ENSCJAG00000017190	ZFYVE21
ENSCJAT00000033497	ENSCJAG00000017204	PPP1R13B
ENSCJAT00000054434	ENSCJAG00000032464	unknown
ENSCJAT00000022983	ENSCJAG00000011834	NUDT14
ENSCJAT00000007284	ENSCJAG00000003801	TMEM123
ENSCJAT00000007251	ENSCJAG00000003781	MMP20
ENSCJAT00000052491	ENSCJAG00000009842	CASP1
ENSCJAT00000019123	ENSCJAG00000009842	CASP1
ENSCJAT00000019090	ENSCJAG00000009842	CASP1
ENSCJAT00000059001	ENSCJAG00000009842	CASP1
ENSCJAT00000019103	ENSCJAG00000009842	CASP1
ENSCJAT00000019116	ENSCJAG00000009842	CASP1
ENSCJAT00000019128	ENSCJAG00000009842	CASP1
ENSCJAT00000054223	ENSCJAG00000009957	ALKBH8
ENSCJAT00000052486	ENSCJAG00000009957	ALKBH8
ENSCJAT00000019673	ENSCJAG00000010127	ACAT1
ENSCJAT00000019719	ENSCJAG00000010132	NPAT
ENSCJAT00000020326	ENSCJAG00000010405	ARHGAP20
ENSCJAT00000021048	ENSCJAG00000010811	C11orf52
ENSCJAT00000024244	ENSCJAG00000012499	IL18
ENSCJAT00000024072	ENSCJAG00000012429	unknown
ENSCJAT00000053295	ENSCJAG00000012306	TTC12
ENSCJAT00000054501	ENSCJAG00000012053	USP28
ENSCJAT00000024346	ENSCJAG00000012570	BUD13
ENSCJAT00000024354	ENSCJAG00000012570	BUD13
ENSCJAT00000062957	ENSCJAG00000012570	BUD13
ENSCJAT00000059110	ENSCJAG00000013044	unknown
ENSCJAT00000053553	ENSCJAG00000013044	unknown
ENSCJAT00000060384	ENSCJAG00000013044	unknown
ENSCJAT00000062516	ENSCJAG00000013044	unknown
ENSCJAT00000025334	ENSCJAG00000013044	unknown
ENSCJAT00000025409	ENSCJAG00000013084	IL10RA
ENSCJAT00000025414	ENSCJAG00000013084	IL10RA
ENSCJAT00000057894	ENSCJAG00000013084	IL10RA
ENSCJAT00000059332	ENSCJAG00000013084	IL10RA
ENSCJAT00000025406	ENSCJAG00000013084	IL10RA
ENSCJAT00000025790	ENSCJAG00000013281	unknown
ENSCJAT00000026196	ENSCJAG00000013463	PHLDB1
ENSCJAT00000026459	ENSCJAG00000013591	DDX6
ENSCJAT00000059107	ENSCJAG00000013591	DDX6
ENSCJAT00000026461	ENSCJAG00000013591	DDX6
ENSCJAT00000055179	ENSCJAG00000013703	TRAPPC4
ENSCJAT00000026646	ENSCJAG00000013703	TRAPPC4
ENSCJAT00000027120	ENSCJAG00000013942	HINFP
ENSCJAT00000027200	ENSCJAG00000014001	NLRX1
ENSCJAT00000062671	ENSCJAG00000014001	NLRX1
ENSCJAT00000027336	ENSCJAG00000014068	unknown
ENSCJAT00000027339	ENSCJAG00000014068	unknown
ENSCJAT00000014495	ENSCJAG00000007368	TBCEL
ENSCJAT00000014452	ENSCJAG00000007368	TBCEL
ENSCJAT00000015315	ENSCJAG00000007804	VWA5A
ENSCJAT00000015332	ENSCJAG00000007804	VWA5A
ENSCJAT00000015368	ENSCJAG00000007804	VWA5A
ENSCJAT00000015357	ENSCJAG00000007804	VWA5A
ENSCJAT00000015822	ENSCJAG00000008040	ROBO3
ENSCJAT00000015731	ENSCJAG00000008040	ROBO3
ENSCJAT00000060473	ENSCJAG00000008040	ROBO3
ENSCJAT00000016451	ENSCJAG00000008400	STT3A

ENSCJAT00000056731	ENSCJAG00000009235	PRDM10
ENSCJAT00000018342	ENSCJAG00000009451	ZBTB44
ENSCJAT00000018348	ENSCJAG00000009451	ZBTB44
ENSCJAT00000018349	ENSCJAG00000009451	ZBTB44
ENSCJAT00000018416	ENSCJAG00000009481	ADAMTS8
ENSCJAT00000018434	ENSCJAG00000009481	ADAMTS8
ENSCJAT00000038329	ENSCJAG00000019542	THYN1
ENSCJAT00000038328	ENSCJAG00000019542	THYN1
ENSCJAT00000063410	ENSCJAG00000019542	THYN1
ENSCJAT00000001129	ENSCJAG00000000595	TRPC6
ENSCJAT00000008235	ENSCJAG00000004281	CEP57
ENSCJAT00000008081	ENSCJAG00000004213	ENDOD1
ENSCJAT00000007799	ENSCJAG00000003984	MRE11A
ENSCJAT00000052101	ENSCJAG00000003984	MRE11A
ENSCJAT00000056319	ENSCJAG00000003984	MRE11A
ENSCJAT00000007903	ENSCJAG00000004075	PIWIL4
ENSCJAT00000007884	ENSCJAG00000004075	PIWIL4
ENSCJAT00000007407	ENSCJAG00000003839	MED17
ENSCJAT00000007394	ENSCJAG00000003839	MED17
ENSCJAT00000060295	ENSCJAG00000003839	MED17
ENSCJAT00000007207	ENSCJAG00000003748	KIAA1731
ENSCJAT00000007163	ENSCJAG00000003746	SMCO4
ENSCJAT00000007154	ENSCJAG00000003734	CCDC67
ENSCJAT00000007159	ENSCJAG00000003734	CCDC67
ENSCJAT00000007144	ENSCJAG00000003734	CCDC67
ENSCJAT00000007140	ENSCJAG00000003734	CCDC67
ENSCJAT00000021562	ENSCJAG00000011030	SLC36A4
ENSCJAT00000021547	ENSCJAG00000011030	SLC36A4
ENSCJAT00000001647	ENSCJAG00000000903	CHORDC1
ENSCJAT00000001642	ENSCJAG00000000903	CHORDC1
ENSCJAT00000001620	ENSCJAG00000000859	NAALAD2
ENSCJAT00000001576	ENSCJAG00000000859	NAALAD2
ENSCJAT00000053997	ENSCJAG00000000859	NAALAD2
ENSCJAT00000062319	ENSCJAG00000000859	NAALAD2
ENSCJAT00000001270	ENSCJAG00000000698	TYR
ENSCJAT00000001243	ENSCJAG00000000679	CTSC
ENSCJAT00000001262	ENSCJAG00000000679	CTSC
ENSCJAT00000001244	ENSCJAG00000000679	CTSC
ENSCJAT00000023491	ENSCJAG00000012092	CCDC81
ENSCJAT00000028077	ENSCJAG00000014359	SYTL2
ENSCJAT00000028068	ENSCJAG00000014359	SYTL2
ENSCJAT00000028006	ENSCJAG00000014359	SYTL2
ENSCJAT00000063299	ENSCJAG00000014359	SYTL2
ENSCJAT00000028001	ENSCJAG00000014359	SYTL2
ENSCJAT00000062758	ENSCJAG00000014792	ALG8
ENSCJAT00000028832	ENSCJAG00000014792	ALG8
ENSCJAT00000028854	ENSCJAG00000014824	KCTD14
ENSCJAT00000054439	ENSCJAG00000014954	PAK1
ENSCJAT00000029738	ENSCJAG00000015204	C11orf30
ENSCJAT00000056163	ENSCJAG00000015204	C11orf30
ENSCJAT00000058806	ENSCJAG00000015204	C11orf30
ENSCJAT00000029729	ENSCJAG00000015204	C11orf30
ENSCJAT00000058650	ENSCJAG00000015204	C11orf30
ENSCJAT00000058925	ENSCJAG00000015204	C11orf30
ENSCJAT00000029872	ENSCJAG00000015337	DGAT2
ENSCJAT00000029855	ENSCJAG00000015337	DGAT2
ENSCJAT00000030076	ENSCJAG00000015424	RPS3
ENSCJAT00000058206	ENSCJAG00000015573	NEU3
ENSCJAT00000030302	ENSCJAG00000015573	NEU3

ENSCJAT00000030305	ENSCJAG00000015573	NEU3
ENSCJAT00000030657	ENSCJAG00000015734	PPME1
ENSCJAT00000030724	ENSCJAG00000015764	C2CD3
ENSCJAT00000031062	ENSCJAG00000015964	unknown
ENSCJAT00000029323	ENSCJAG00000015038	ARAP1
ENSCJAT00000029687	ENSCJAG00000015246	unknown
ENSCJAT00000053035	ENSCJAG00000032556	unknown
ENSCJAT00000030087	ENSCJAG00000015457	ART1
ENSCJAT00000030360	ENSCJAG00000015591	TRIM21
ENSCJAT00000052362	ENSCJAG00000015591	TRIM21
ENSCJAT00000030419	ENSCJAG00000015632	OLR45
ENSCJAT00000039541	ENSCJAG00000020117	unknown
ENSCJAT00000026792	ENSCJAG00000013790	ZNF214
ENSCJAT00000026779	ENSCJAG00000013765	NLRP14
ENSCJAT00000061770	ENSCJAG00000013765	NLRP14
ENSCJAT00000057667	ENSCJAG00000013765	NLRP14
ENSCJAT00000023080	ENSCJAG00000011854	TUB
ENSCJAT00000022949	ENSCJAG00000011812	LMO1
ENSCJAT00000022907	ENSCJAG00000011801	STK33
ENSCJAT00000022689	ENSCJAG00000011668	TMEM9B
ENSCJAT00000063153	ENSCJAG00000011668	TMEM9B
ENSCJAT00000022641	ENSCJAG00000011630	NRIP3
ENSCJAT00000022626	ENSCJAG00000011630	NRIP3
ENSCJAT00000061476	ENSCJAG00000011395	IPO7
ENSCJAT00000021500	ENSCJAG00000011014	RNF141
ENSCJAT00000019725	ENSCJAG00000010121	BTBD10
ENSCJAT00000056616	ENSCJAG00000010121	BTBD10
ENSCJAT00000019282	ENSCJAG00000038149	COPB1
ENSCJAT00000019472	ENSCJAG00000009850	PSMA1
ENSCJAT00000019477	ENSCJAG00000009850	PSMA1
ENSCJAT00000018877	ENSCJAG00000009705	INSC
ENSCJAT00000013684	ENSCJAG00000007006	UEVLD
ENSCJAT00000059153	ENSCJAG00000006985	SPTY2D1
ENSCJAT00000013646	ENSCJAG00000006985	SPTY2D1
ENSCJAT00000053578	ENSCJAG00000006985	SPTY2D1
ENSCJAT00000013665	ENSCJAG00000006985	SPTY2D1
ENSCJAT00000013343	ENSCJAG00000006811	E2F8
ENSCJAT00000000166	ENSCJAG00000006408	SLC17A6
ENSCJAT00000012261	ENSCJAG00000006285	ANO3
ENSCJAT00000012342	ENSCJAG00000006336	SLC5A12
ENSCJAT00000012184	ENSCJAG00000006265	BBOX1
ENSCJAT00000053449	ENSCJAG00000006191	BDNF
ENSCJAT00000062042	ENSCJAG00000006191	BDNF
ENSCJAT00000011986	ENSCJAG00000006191	BDNF
ENSCJAT00000012005	ENSCJAG00000006191	BDNF
ENSCJAT00000056021	ENSCJAG00000006191	BDNF
ENSCJAT00000012020	ENSCJAG00000006191	BDNF
ENSCJAT00000055566	ENSCJAG00000006191	BDNF
ENSCJAT00000011973	ENSCJAG00000006149	KIF18A
ENSCJAT00000054061	ENSCJAG00000006055	unknown
ENSCJAT00000056798	ENSCJAG00000005942	PAX6
ENSCJAT00000011243	ENSCJAG00000005797	PRRG4
ENSCJAT00000010999	ENSCJAG00000005638	TCP11L1
ENSCJAT00000020329	ENSCJAG00000010403	KIAA1549L
ENSCJAT00000020319	ENSCJAG00000010403	KIAA1549L
ENSCJAT00000020309	ENSCJAG00000010403	KIAA1549L
ENSCJAT00000020355	ENSCJAG00000010443	CD59
ENSCJAT00000020843	ENSCJAG00000010585	NAT10
ENSCJAT00000020840	ENSCJAG00000010585	NAT10

ENSCJAT00000020808	ENSCJAG00000010585	NAT10
ENSCJAT00000052626	ENSCJAG00000010711	ABTB2
ENSCJAT00000018147	ENSCJAG00000010775	ELF5
ENSCJAT00000021197	ENSCJAG00000010844	APIP
ENSCJAT00000021408	ENSCJAG00000010977	PAMR1
ENSCJAT00000021460	ENSCJAG00000011000	TRIM44
ENSCJAT00000021559	ENSCJAG00000011056	PRR5L
ENSCJAT00000058372	ENSCJAG00000011056	PRR5L
ENSCJAT00000060212	ENSCJAG00000011056	PRR5L
ENSCJAT00000053731	ENSCJAG00000011130	API5
ENSCJAT00000021854	ENSCJAG00000011130	API5
ENSCJAT00000021843	ENSCJAG00000011130	API5
ENSCJAT00000058150	ENSCJAG00000011130	API5
ENSCJAT00000021812	ENSCJAG00000011130	API5
ENSCJAT00000059406	ENSCJAG00000011232	TTC17
ENSCJAT00000022090	ENSCJAG00000011353	ALKBH3
ENSCJAT00000053993	ENSCJAG00000011353	ALKBH3
ENSCJAT00000022099	ENSCJAG00000011353	ALKBH3
ENSCJAT00000022270	ENSCJAG00000011453	CD82
ENSCJAT00000022297	ENSCJAG00000011462	TP53I11
ENSCJAT00000039203	ENSCJAG00000019944	LRP4
ENSCJAT00000039193	ENSCJAG00000019944	LRP4
ENSCJAT00000039409	ENSCJAG00000020045	MADD
ENSCJAT00000039406	ENSCJAG00000020045	MADD
ENSCJAT00000052300	ENSCJAG00000020045	MADD
ENSCJAT00000062438	ENSCJAG00000020045	MADD
ENSCJAT00000058029	ENSCJAG00000020045	MADD
ENSCJAT00000059846	ENSCJAG00000009145	OR8U1
ENSCJAT00000017659	ENSCJAG00000009120	OR5M11
ENSCJAT00000017566	ENSCJAG00000009066	LRRC55
ENSCJAT00000017297	ENSCJAG00000036833	PRG2
ENSCJAT00000060710	ENSCJAG00000036833	PRG2
ENSCJAT00000017057	ENSCJAG00000008787	SERPING1
ENSCJAT00000017079	ENSCJAG00000008787	SERPING1
ENSCJAT00000017072	ENSCJAG00000008787	SERPING1
ENSCJAT00000057001	ENSCJAG00000008787	SERPING1
ENSCJAT00000017065	ENSCJAG00000008787	SERPING1
ENSCJAT00000016326	ENSCJAG00000008395	GLYATL1
ENSCJAT00000016323	ENSCJAG00000008395	GLYATL1
ENSCJAT00000031479	ENSCJAG00000016132	OSBP
ENSCJAT00000031473	ENSCJAG00000016132	OSBP
ENSCJAT00000031553	ENSCJAG00000016192	PATL1
ENSCJAT00000031732	ENSCJAG00000016313	GIF
ENSCJAT00000031774	ENSCJAG00000016335	MS4A3
ENSCJAT00000031853	ENSCJAG00000016376	unknown
ENSCJAT00000058630	ENSCJAG00000016376	unknown
ENSCJAT00000031856	ENSCJAG00000016388	unknown
ENSCJAT00000032019	ENSCJAG00000016453	MS4A15
ENSCJAT00000032039	ENSCJAG00000016453	MS4A15
ENSCJAT00000032047	ENSCJAG00000016478	MS4A10
ENSCJAT00000032331	ENSCJAG00000016626	SLC15A3
ENSCJAT00000032353	ENSCJAG00000016626	SLC15A3
ENSCJAT00000037843	ENSCJAG00000019302	CYB561A3
ENSCJAT00000037841	ENSCJAG00000019302	CYB561A3
ENSCJAT00000037778	ENSCJAG00000019276	SDHAF2
ENSCJAT00000037781	ENSCJAG00000019276	SDHAF2
ENSCJAT00000037464	ENSCJAG00000019095	BEST1
ENSCJAT00000037510	ENSCJAG00000019095	BEST1
ENSCJAT00000037507	ENSCJAG00000019095	BEST1

ENSCJAT00000037471	ENSCJAG00000019095	BEST1
ENSCJAT00000037380	ENSCJAG00000019064	SCGB2A1
ENSCJAT00000036732	ENSCJAG00000018709	unknown
ENSCJAT00000037006	ENSCJAG00000018860	B3GAT3
ENSCJAT00000037011	ENSCJAG00000018860	B3GAT3
ENSCJAT00000036902	ENSCJAG00000018789	C11orf48
ENSCJAT00000036208	ENSCJAG00000018481	HRASLS2
ENSCJAT00000036196	ENSCJAG00000018481	HRASLS2
ENSCJAT00000035683	ENSCJAG00000018209	STIP1
ENSCJAT00000005716	ENSCJAG00000002977	SLC22A12
ENSCJAT00000005713	ENSCJAG00000002977	SLC22A12
ENSCJAT00000005415	ENSCJAG00000002780	SF1
ENSCJAT00000055994	ENSCJAG00000002780	SF1
ENSCJAT00000005388	ENSCJAG00000002780	SF1
ENSCJAT00000005410	ENSCJAG00000002780	SF1
ENSCJAT00000056975	ENSCJAG00000002780	SF1
ENSCJAT00000005230	ENSCJAG00000002730	MAP4K2
ENSCJAT00000004714	ENSCJAG00000002441	TM7SF2
ENSCJAT00000004708	ENSCJAG00000002441	TM7SF2
ENSCJAT00000004721	ENSCJAG00000002441	TM7SF2
ENSCJAT00000043178	ENSCJAG00000022193	TIGD3
ENSCJAT00000004329	ENSCJAG00000002274	FRMD8
ENSCJAT00000004288	ENSCJAG00000002235	SCYL1
ENSCJAT00000004303	ENSCJAG00000002235	SCYL1
ENSCJAT00000057456	ENSCJAG00000002115	PCNXL3
ENSCJAT00000003994	ENSCJAG00000002062	SIPA1
ENSCJAT00000004002	ENSCJAG00000002062	SIPA1
ENSCJAT00000053642	ENSCJAG00000002062	SIPA1
ENSCJAT00000004318	ENSCJAG00000002062	SIPA1
ENSCJAT00000063276	ENSCJAG00000001847	EFEMP2
ENSCJAT00000052669	ENSCJAG00000001847	EFEMP2
ENSCJAT00000003459	ENSCJAG00000001795	FIBP
ENSCJAT00000003369	ENSCJAG00000001776	FOSL1
ENSCJAT00000003370	ENSCJAG00000001776	FOSL1
ENSCJAT00000003223	ENSCJAG00000001701	GAL3ST3
ENSCJAT00000003220	ENSCJAG00000001701	GAL3ST3
ENSCJAT00000003033	ENSCJAG00000001599	RAB1B
ENSCJAT00000003030	ENSCJAG00000001599	RAB1B
ENSCJAT00000002841	ENSCJAG00000001508	B3GNT1
ENSCJAT00000002838	ENSCJAG00000001508	B3GNT1
ENSCJAT00000002435	ENSCJAG00000001313	CCDC87
ENSCJAT00000002426	ENSCJAG00000001305	CCS
ENSCJAT00000059933	ENSCJAG00000001294	RBM14
ENSCJAT00000002055	ENSCJAG00000001124	RHOD
ENSCJAT00000001892	ENSCJAG00000001006	ADRBK1
ENSCJAT00000001878	ENSCJAG00000001006	ADRBK1
ENSCJAT00000001942	ENSCJAG00000001059	unknown
ENSCJAT00000001936	ENSCJAG00000001059	unknown
ENSCJAT00000030817	ENSCJAG00000015752	PPP6R3
ENSCJAT00000030851	ENSCJAG00000015858	GAL
ENSCJAT00000054039	ENSCJAG00000000932	FADD
ENSCJAT00000001687	ENSCJAG00000000932	FADD
ENSCJAT00000022383	ENSCJAG00000011520	MRGPRG
ENSCJAT00000022659	ENSCJAG00000011636	NAP1L4
ENSCJAT00000022704	ENSCJAG00000011699	SLC22A18AS
ENSCJAT00000036168	ENSCJAG00000018439	TNNT3
ENSCJAT00000036178	ENSCJAG00000018439	TNNT3
ENSCJAT00000036172	ENSCJAG00000018439	TNNT3
ENSCJAT00000036018	ENSCJAG00000018403	IFITM10

ENSCJAT00000036015	ENSCJAG00000018403	IFITM10
ENSCJAT00000022734	ENSCJAG00000011689	CHID1
ENSCJAT00000023411	ENSCJAG00000012048	DRD4
ENSCJAT00000023644	ENSCJAG00000012183	LMNTD2
ENSCJAT00000023648	ENSCJAG00000012183	LMNTD2
ENSCJAT00000017779	ENSCJAG00000009166	KBTBD2
ENSCJAT00000017772	ENSCJAG00000009166	KBTBD2
ENSCJAT00000017568	ENSCJAG00000009035	NT5C3A
ENSCJAT00000056332	ENSCJAG00000009035	NT5C3A
ENSCJAT00000017500	ENSCJAG00000009030	unknown
ENSCJAT00000017490	ENSCJAG00000009030	unknown
ENSCJAT00000059375	ENSCJAG00000012461	AOAH
ENSCJAT00000033249	ENSCJAG00000007624	YAE1D1
ENSCJAT00000014923	ENSCJAG00000007641	CDK13
ENSCJAT00000015408	ENSCJAG00000007875	UBE2D4
ENSCJAT00000055080	ENSCJAG00000007875	UBE2D4
ENSCJAT00000023317	ENSCJAG00000011984	ZMIZ2
ENSCJAT00000022985	ENSCJAG00000011833	NACAD
ENSCJAT00000028658	ENSCJAG00000014725	SUN3
ENSCJAT00000016716	ENSCJAG00000008589	GGCT
ENSCJAT00000054967	ENSCJAG00000008589	GGCT
ENSCJAT00000016633	ENSCJAG00000008540	ZNRF2
ENSCJAT00000052225	ENSCJAG00000008478	SCRN1
ENSCJAT00000059053	ENSCJAG00000008478	SCRN1
ENSCJAT00000054218	ENSCJAG00000008478	SCRN1
ENSCJAT00000059151	ENSCJAG00000008478	SCRN1
ENSCJAT00000016512	ENSCJAG00000008478	SCRN1
ENSCJAT00000016516	ENSCJAG00000008478	SCRN1
ENSCJAT00000016397	ENSCJAG00000008406	CHN2
ENSCJAT00000016431	ENSCJAG00000008406	CHN2
ENSCJAT00000016393	ENSCJAG00000008406	CHN2
ENSCJAT00000016391	ENSCJAG00000008406	CHN2
ENSCJAT00000016352	ENSCJAG00000008406	CHN2
ENSCJAT00000016385	ENSCJAG00000008406	CHN2
ENSCJAT00000058478	ENSCJAG00000008204	HIBADH
ENSCJAT00000015840	ENSCJAG00000008096	unknown
ENSCJAT00000015908	ENSCJAG00000008096	unknown
ENSCJAT00000015514	ENSCJAG00000007952	unknown
ENSCJAT00000002638	ENSCJAG00000001412	TOMM7
ENSCJAT00000002628	ENSCJAG00000001400	IL6
ENSCJAT00000058926	ENSCJAG00000001400	IL6
ENSCJAT00000063890	ENSCJAG00000036784	RAPGEF5
ENSCJAT00000002595	ENSCJAG00000036784	RAPGEF5
ENSCJAT00000002588	ENSCJAG00000036784	RAPGEF5
ENSCJAT00000002157	ENSCJAG00000001171	ITGB8
ENSCJAT00000002167	ENSCJAG00000001171	ITGB8
ENSCJAT00000001876	ENSCJAG00000001029	AHR
ENSCJAT00000001868	ENSCJAG00000001029	AHR
ENSCJAT00000001837	ENSCJAG00000000997	AGR2
ENSCJAT00000054291	ENSCJAG00000000997	AGR2
ENSCJAT00000001792	ENSCJAG00000000954	BZW2
ENSCJAT00000063592	ENSCJAG00000000954	BZW2
ENSCJAT00000052177	ENSCJAG00000000954	BZW2
ENSCJAT00000001689	ENSCJAG00000000930	MEOX2
ENSCJAT00000001688	ENSCJAG00000000930	MEOX2
ENSCJAT00000001447	ENSCJAG00000000800	VWDE
ENSCJAT00000001273	ENSCJAG00000000686	MIOS
ENSCJAT00000059805	ENSCJAG00000000618	ASNS
ENSCJAT00000054002	ENSCJAG00000000618	ASNS

ENSCJAT00000001149	ENSCJAG00000000618	ASNS
ENSCJAT00000025775	ENSCJAG00000013229	PDK4
ENSCJAT00000056341	ENSCJAG00000013280	ASB4
ENSCJAT00000025961	ENSCJAG00000013355	PON1
ENSCJAT00000026324	ENSCJAG00000013493	CASD1
ENSCJAT00000055960	ENSCJAG00000013493	CASD1
ENSCJAT00000026647	ENSCJAG00000013547	COL1A2
ENSCJAT00000026645	ENSCJAG00000013547	COL1A2
ENSCJAT00000055492	ENSCJAG00000013758	CALCR
ENSCJAT00000026950	ENSCJAG00000013864	HEPACAM2
ENSCJAT00000026980	ENSCJAG00000013864	HEPACAM2
ENSCJAT00000026968	ENSCJAG00000013864	HEPACAM2
ENSCJAT00000052397	ENSCJAG00000013927	PEX1
ENSCJAT00000052715	ENSCJAG00000013927	PEX1
ENSCJAT00000027261	ENSCJAG00000014019	unknown
ENSCJAT00000057373	ENSCJAG00000023041	CLDN12
ENSCJAT00000044026	ENSCJAG00000023041	CLDN12
ENSCJAT00000028654	ENSCJAG00000014724	TMEM243
ENSCJAT00000059311	ENSCJAG00000031605	unknown
ENSCJAT00000059670	ENSCJAG00000034653	unknown
ENSCJAT00000011999	ENSCJAG00000006177	RSBN1L
ENSCJAT00000011991	ENSCJAG00000006177	RSBN1L
ENSCJAT00000056096	ENSCJAG00000006177	RSBN1L
ENSCJAT00000011734	ENSCJAG00000006020	GSAP
ENSCJAT00000056672	ENSCJAG00000021225	LRRC17
ENSCJAT00000041758	ENSCJAG00000021225	LRRC17
ENSCJAT00000041628	ENSCJAG00000012752	SLC26A5
ENSCJAT00000024465	ENSCJAG00000012572	ORC5
ENSCJAT00000055944	ENSCJAG00000012572	ORC5
ENSCJAT00000063197	ENSCJAG00000012572	ORC5
ENSCJAT00000031549	ENSCJAG00000016193	NAMPT
ENSCJAT00000032189	ENSCJAG00000016477	LAMB1
ENSCJAT00000032169	ENSCJAG00000016477	LAMB1
ENSCJAT00000033241	ENSCJAG00000017110	MDFIC
ENSCJAT00000033231	ENSCJAG00000017110	MDFIC
ENSCJAT00000009611	ENSCJAG00000004983	WNT2
ENSCJAT00000009276	ENSCJAG00000004812	LSM8
ENSCJAT00000009267	ENSCJAG00000004809	unknown
ENSCJAT00000027693	ENSCJAG00000014233	ATP1B4
ENSCJAT00000027680	ENSCJAG00000014233	ATP1B4
ENSCJAT00000027708	ENSCJAG00000014233	ATP1B4
ENSCJAT00000008268	ENSCJAG00000004298	UBE3C
ENSCJAT00000055112	ENSCJAG00000004298	UBE3C
ENSCJAT00000008345	ENSCJAG00000004357	RNF32
ENSCJAT00000008349	ENSCJAG00000004357	RNF32
ENSCJAT00000060759	ENSCJAG00000004357	RNF32
ENSCJAT00000061422	ENSCJAG00000000158	HTR5A
ENSCJAT00000000285	ENSCJAG00000000158	HTR5A
ENSCJAT00000038564	ENSCJAG00000032080	XRCC2
ENSCJAT00000055019	ENSCJAG00000019740	GALNTL5
ENSCJAT00000040089	ENSCJAG00000020414	RHEB
ENSCJAT00000040077	ENSCJAG00000020410	CRYGN
ENSCJAT00000040075	ENSCJAG00000020410	CRYGN
ENSCJAT00000039999	ENSCJAG00000020371	ABCF2
ENSCJAT00000040003	ENSCJAG00000020371	ABCF2
ENSCJAT00000039768	ENSCJAG00000020233	ABCB8
ENSCJAT00000039766	ENSCJAG00000020233	ABCB8
ENSCJAT00000039770	ENSCJAG00000020233	ABCB8
ENSCJAT00000039771	ENSCJAG00000020233	ABCB8

ENSCJAT00000014771	ENSCJAG00000020150	KCNH2
ENSCJAT00000039620	ENSCJAG00000020150	KCNH2
ENSCJAT00000055141	ENSCJAG00000020150	KCNH2
ENSCJAT00000019980	ENSCJAG00000031758	ZNF783
ENSCJAT00000019975	ENSCJAG00000010260	ZNF212
ENSCJAT00000019853	ENSCJAG00000035501	ZNF425
ENSCJAT00000019555	ENSCJAG00000010002	CUL1
ENSCJAT00000019563	ENSCJAG00000010002	CUL1
ENSCJAT00000019170	ENSCJAG00000009888	unknown
ENSCJAT00000018445	ENSCJAG00000009471	KEL
ENSCJAT00000018292	ENSCJAG00000009427	TRPV6
ENSCJAT00000026480	ENSCJAG00000013621	KIAA1147
ENSCJAT00000026411	ENSCJAG00000013588	unknown
ENSCJAT00000026302	ENSCJAG00000013524	NDUFB2
ENSCJAT00000057080	ENSCJAG00000013198	unknown
ENSCJAT00000025697	ENSCJAG00000013198	unknown
ENSCJAT00000062056	ENSCJAG00000013198	unknown
ENSCJAT00000059448	ENSCJAG00000013198	unknown
ENSCJAT00000025537	ENSCJAG00000013133	TTC26
ENSCJAT00000025545	ENSCJAG00000013133	TTC26
ENSCJAT00000025581	ENSCJAG00000013133	TTC26
ENSCJAT00000024810	ENSCJAG00000012786	PTN
ENSCJAT00000024480	ENSCJAG00000012599	NUP205
ENSCJAT00000023813	ENSCJAG00000012283	unknown
ENSCJAT00000056814	ENSCJAG00000012283	unknown
ENSCJAT00000055658	ENSCJAG00000016372	PODXL
ENSCJAT00000020525	ENSCJAG00000010520	UBE2H
ENSCJAT00000020224	ENSCJAG00000010323	AHCYL2
ENSCJAT00000020181	ENSCJAG00000010323	AHCYL2
ENSCJAT00000020201	ENSCJAG00000010323	AHCYL2
ENSCJAT00000058734	ENSCJAG00000018061	IRF5
ENSCJAT00000034723	ENSCJAG00000017814	FAM71F2
ENSCJAT00000034176	ENSCJAG00000017546	GPR37
ENSCJAT00000059883	ENSCJAG00000017422	SLC13A1
ENSCJAT00000033923	ENSCJAG00000017422	SLC13A1
ENSCJAT00000033911	ENSCJAG00000017422	SLC13A1
ENSCJAT00000033368	ENSCJAG00000017145	ING3
ENSCJAT00000033336	ENSCJAG00000017145	ING3
ENSCJAT00000033325	ENSCJAG00000017145	ING3
ENSCJAT00000024908	ENSCJAG00000034484	unknown
ENSCJAT00000029144	ENSCJAG00000014975	SLC48A1
ENSCJAT00000020072	ENSCJAG00000014979	HDAC7
ENSCJAT00000029224	ENSCJAG00000014979	HDAC7
ENSCJAT00000029267	ENSCJAG00000015018	VDR
ENSCJAT00000002455	ENSCJAG00000001320	PLEKHA5
ENSCJAT00000025560	ENSCJAG00000021598	EPS8
ENSCJAT00000042468	ENSCJAG00000021598	EPS8
ENSCJAT00000059109	ENSCJAG00000021598	EPS8
ENSCJAT00000025553	ENSCJAG00000021598	EPS8
ENSCJAT00000025557	ENSCJAG00000021598	EPS8
ENSCJAT00000042476	ENSCJAG00000021602	RERG
ENSCJAT00000042474	ENSCJAG00000021602	RERG
ENSCJAT00000042475	ENSCJAG00000021602	RERG
ENSCJAT00000042479	ENSCJAG00000021605	ARHGDIB
ENSCJAT00000042481	ENSCJAG00000021607	MGP
ENSCJAT00000042543	ENSCJAG00000021638	CREBL2
ENSCJAT00000042570	ENSCJAG00000021653	unknown
ENSCJAT00000057446	ENSCJAG00000021661	STYK1
ENSCJAT00000042588	ENSCJAG00000021663	NKG2A

ENSCJAT00000042585	ENSCJAG00000021663	NKG2A
ENSCJAT00000042589	ENSCJAG00000021663	NKG2A
ENSCJAT00000042599	ENSCJAG00000021669	CD94
ENSCJAT00000042601	ENSCJAG00000021669	CD94
ENSCJAT00000056604	ENSCJAG00000021669	CD94
ENSCJAT00000053411	ENSCJAG00000021669	CD94
ENSCJAT00000028554	ENSCJAG00000021669	CD94
ENSCJAT00000042600	ENSCJAG00000021669	CD94
ENSCJAT00000042598	ENSCJAG00000021669	CD94
ENSCJAT00000042603	ENSCJAG00000021670	unknown
ENSCJAT00000042602	ENSCJAG00000021670	unknown
ENSCJAT00000042620	ENSCJAG00000021673	CLEC7A
ENSCJAT00000042621	ENSCJAG00000021673	CLEC7A
ENSCJAT00000042614	ENSCJAG00000021673	CLEC7A
ENSCJAT00000042617	ENSCJAG00000021673	CLEC7A
ENSCJAT00000042618	ENSCJAG00000021673	CLEC7A
ENSCJAT00000042616	ENSCJAG00000021673	CLEC7A
ENSCJAT00000042628	ENSCJAG00000021674	CLEC1A
ENSCJAT00000042625	ENSCJAG00000021674	CLEC1A
ENSCJAT00000042648	ENSCJAG00000021683	unknown
ENSCJAT00000013319	ENSCJAG00000006797	SLC6A12
ENSCJAT00000010376	ENSCJAG00000006797	SLC6A12
ENSCJAT00000013337	ENSCJAG00000006797	SLC6A12
ENSCJAT00000010385	ENSCJAG00000006797	SLC6A12
ENSCJAT00000001294	ENSCJAG00000000716	LRTM2
ENSCJAT00000001300	ENSCJAG00000000716	LRTM2
ENSCJAT00000036796	ENSCJAG00000018746	FKBP4
ENSCJAT00000036867	ENSCJAG00000018790	RHNO1
ENSCJAT00000003026	ENSCJAG00000001601	C12orf5
ENSCJAT00000003091	ENSCJAG00000001628	C12orf4
ENSCJAT00000001258	ENSCJAG00000000689	SCNN1A
ENSCJAT00000001277	ENSCJAG00000000689	SCNN1A
ENSCJAT00000001275	ENSCJAG00000000689	SCNN1A
ENSCJAT00000001301	ENSCJAG00000000719	CD27
ENSCJAT00000014048	ENSCJAG00000037676	IFFO1
ENSCJAT00000060190	ENSCJAG00000037676	IFFO1
ENSCJAT00000015897	ENSCJAG00000008114	CHD4
ENSCJAT00000015426	ENSCJAG00000007835	COPS7A
ENSCJAT00000015267	ENSCJAG00000007790	DYRK4
ENSCJAT00000013961	ENSCJAG00000007120	unknown
ENSCJAT00000061681	ENSCJAG00000034245	unknown
ENSCJAT00000036946	ENSCJAG00000018810	RIMKLB
ENSCJAT00000036569	ENSCJAG00000018610	PZP
ENSCJAT00000036560	ENSCJAG00000018610	PZP
ENSCJAT00000036479	ENSCJAG00000018605	CLEC2B
ENSCJAT00000025106	ENSCJAG00000012877	SLC38A4
ENSCJAT00000025093	ENSCJAG00000012877	SLC38A4
ENSCJAT00000025652	ENSCJAG00000013213	DBX2
ENSCJAT00000034497	ENSCJAG00000017695	PRICKLE1
ENSCJAT00000056459	ENSCJAG00000034165	unknown
ENSCJAT00000035177	ENSCJAG00000017961	KIF21A
ENSCJAT00000063564	ENSCJAG00000018129	YARS2
ENSCJAT00000035438	ENSCJAG00000018129	YARS2
ENSCJAT00000002236	ENSCJAG00000001215	unknown
ENSCJAT00000052775	ENSCJAG00000001215	unknown
ENSCJAT00000001960	ENSCJAG00000001057	IPO8
ENSCJAT00000057207	ENSCJAG00000001057	IPO8
ENSCJAT00000060300	ENSCJAG00000001057	IPO8
ENSCJAT00000040869	ENSCJAG00000020798	unknown

ENSCJAT00000041081	ENSCJAG00000020904	PRPH
ENSCJAT00000041106	ENSCJAG00000020919	DNAJC22
ENSCJAT00000041424	ENSCJAG00000021058	RACGAP1
ENSCJAT00000041409	ENSCJAG00000021058	RACGAP1
ENSCJAT00000041467	ENSCJAG00000021089	GPD1
ENSCJAT00000041522	ENSCJAG00000021115	LIMA1
ENSCJAT00000013376	ENSCJAG00000021115	LIMA1
ENSCJAT00000034232	ENSCJAG00000017564	LETMD1
ENSCJAT00000035664	ENSCJAG00000018242	unknown
ENSCJAT00000053963	ENSCJAG00000017646	POU6F1
ENSCJAT00000034380	ENSCJAG00000017646	POU6F1
ENSCJAT00000034443	ENSCJAG00000017680	SMAGP
ENSCJAT00000034678	ENSCJAG00000017737	SLC4A8
ENSCJAT00000059123	ENSCJAG00000017870	ANKRD33
ENSCJAT00000034858	ENSCJAG00000017870	ANKRD33
ENSCJAT00000034853	ENSCJAG00000017870	ANKRD33
ENSCJAT00000034847	ENSCJAG00000017870	ANKRD33
ENSCJAT00000034979	ENSCJAG00000017929	ATG101
ENSCJAT00000054142	ENSCJAG00000017929	ATG101
ENSCJAT00000035013	ENSCJAG00000017933	KRT80
ENSCJAT00000035009	ENSCJAG00000017933	KRT80
ENSCJAT00000035024	ENSCJAG00000017950	unknown
ENSCJAT00000035130	ENSCJAG00000017960	unknown
ENSCJAT00000035280	ENSCJAG00000038154	KRT75
ENSCJAT00000035380	ENSCJAG00000018016	KRT1
ENSCJAT00000035375	ENSCJAG00000018016	KRT1
ENSCJAT00000035347	ENSCJAG00000018122	KRT76
ENSCJAT00000035159	ENSCJAG00000018122	KRT76
ENSCJAT00000057851	ENSCJAG00000018094	KRT4
ENSCJAT00000035173	ENSCJAG00000018094	KRT4
ENSCJAT00000036088	ENSCJAG00000018408	RARG
ENSCJAT00000037208	ENSCJAG00000018968	GTSF1
ENSCJAT00000037395	ENSCJAG00000019038	PDE1B
ENSCJAT00000037390	ENSCJAG00000019038	PDE1B
ENSCJAT00000037460	ENSCJAG00000019103	TESPA1
ENSCJAT00000037456	ENSCJAG00000019103	TESPA1
ENSCJAT00000037477	ENSCJAG00000019103	TESPA1
ENSCJAT00000022589	ENSCJAG00000011620	MYL6B
ENSCJAT00000022592	ENSCJAG00000011620	MYL6B
ENSCJAT00000022265	ENSCJAG00000011455	NABP2
ENSCJAT00000029750	ENSCJAG00000011455	NABP2
ENSCJAT00000021926	ENSCJAG00000011259	unknown
ENSCJAT00000021549	ENSCJAG00000010984	TIMELESS
ENSCJAT00000021529	ENSCJAG00000010984	TIMELESS
ENSCJAT00000021556	ENSCJAG00000010984	TIMELESS
ENSCJAT00000021017	ENSCJAG00000010774	BAZ2A
ENSCJAT00000021032	ENSCJAG00000010774	BAZ2A
ENSCJAT00000020858	ENSCJAG00000010701	NACA
ENSCJAT00000020868	ENSCJAG00000010701	NACA
ENSCJAT00000062964	ENSCJAG00000010652	HSD17B6
ENSCJAT00000020752	ENSCJAG00000010652	HSD17B6
ENSCJAT00000020748	ENSCJAG00000010652	HSD17B6
ENSCJAT00000059994	ENSCJAG00000010327	STAT6
ENSCJAT00000020261	ENSCJAG00000010327	STAT6
ENSCJAT00000020250	ENSCJAG00000010327	STAT6
ENSCJAT00000020207	ENSCJAG00000010327	STAT6
ENSCJAT00000060660	ENSCJAG00000010327	STAT6
ENSCJAT00000055344	ENSCJAG00000010327	STAT6
ENSCJAT00000062928	ENSCJAG00000010327	STAT6

ENSCJAT00000058230	ENSCJAG00000007356	INHBE
ENSCJAT00000013636	ENSCJAG00000006966	DTX3
ENSCJAT00000013619	ENSCJAG00000006966	DTX3
ENSCJAT00000012819	ENSCJAG00000006549	AVIL
ENSCJAT00000012837	ENSCJAG00000006549	AVIL
ENSCJAT00000058339	ENSCJAG00000006419	SLC16A7
ENSCJAT00000012198	ENSCJAG00000006144	MON2
ENSCJAT00000012214	ENSCJAG00000006144	MON2
ENSCJAT00000012133	ENSCJAG00000006144	MON2
ENSCJAT00000012221	ENSCJAG00000006144	MON2
ENSCJAT00000012232	ENSCJAG00000006144	MON2
ENSCJAT00000028870	ENSCJAG00000014810	C12orf56
ENSCJAT00000028727	ENSCJAG00000014734	TBK1
ENSCJAT00000028352	ENSCJAG00000014576	unknown
ENSCJAT00000028318	ENSCJAG00000014558	TMBIM4
ENSCJAT00000028323	ENSCJAG00000014558	TMBIM4
ENSCJAT00000028319	ENSCJAG00000014558	TMBIM4
ENSCJAT00000028321	ENSCJAG00000014558	TMBIM4
ENSCJAT00000014052	ENSCJAG00000007177	DYRK2
ENSCJAT00000003365	ENSCJAG00000001779	YEATS4
ENSCJAT00000003080	ENSCJAG00000037154	unknown
ENSCJAT00000003077	ENSCJAG00000037154	unknown
ENSCJAT00000003075	ENSCJAG00000037154	unknown
ENSCJAT00000056781	ENSCJAG00000001567	CNOT2
ENSCJAT00000003052	ENSCJAG00000001567	CNOT2
ENSCJAT00000055391	ENSCJAG00000001567	CNOT2
ENSCJAT00000002915	ENSCJAG00000001515	unknown
ENSCJAT00000002660	ENSCJAG00000001384	ZFC3H1
ENSCJAT00000002657	ENSCJAG00000001384	ZFC3H1
ENSCJAT00000002451	ENSCJAG00000001306	TPH2
ENSCJAT00000037876	ENSCJAG00000019271	PPP1R12A
ENSCJAT00000037879	ENSCJAG00000019271	PPP1R12A
ENSCJAT00000037850	ENSCJAG00000019271	PPP1R12A
ENSCJAT00000037869	ENSCJAG00000019271	PPP1R12A
ENSCJAT00000037854	ENSCJAG00000019271	PPP1R12A
ENSCJAT00000037906	ENSCJAG00000019332	unknown
ENSCJAT00000038032	ENSCJAG00000019396	CCDC59
ENSCJAT00000038090	ENSCJAG00000019425	SLC6A15
ENSCJAT00000038117	ENSCJAG00000019425	SLC6A15
ENSCJAT00000038153	ENSCJAG00000019458	ALX1
ENSCJAT00000038238	ENSCJAG00000019495	PLEKHG7
ENSCJAT00000038247	ENSCJAG00000019504	CLLU1OS
ENSCJAT00000038530	ENSCJAG00000019637	POC1B
ENSCJAT00000038513	ENSCJAG00000019637	POC1B
ENSCJAT00000038525	ENSCJAG00000019637	POC1B
ENSCJAT00000038507	ENSCJAG00000019637	POC1B
ENSCJAT00000038630	ENSCJAG00000019685	TMTC3
ENSCJAT00000038763	ENSCJAG00000019698	CEP290
ENSCJAT00000038760	ENSCJAG00000019698	CEP290
ENSCJAT00000038718	ENSCJAG00000019698	CEP290
ENSCJAT00000052941	ENSCJAG00000019698	CEP290
ENSCJAT00000038678	ENSCJAG00000019698	CEP290
ENSCJAT00000053479	ENSCJAG00000032825	MRPL42
ENSCJAT00000055869	ENSCJAG00000032825	MRPL42
ENSCJAT00000005677	ENSCJAG00000032825	MRPL42
ENSCJAT00000062110	ENSCJAG00000002964	SOCS2
ENSCJAT00000005436	ENSCJAG00000002810	NR2C1
ENSCJAT00000005272	ENSCJAG00000002740	METAP2
ENSCJAT00000053091	ENSCJAG00000002740	METAP2

ENSCJAT00000005268	ENSCJAG00000002740	METAP2
ENSCJAT00000005276	ENSCJAG00000002740	METAP2
ENSCJAT00000005170	ENSCJAG00000002685	CCDC38
ENSCJAT00000005131	ENSCJAG00000002672	AMDHD1
ENSCJAT00000005042	ENSCJAG00000002595	LTA4H
ENSCJAT00000063603	ENSCJAG00000002518	C12orf55
ENSCJAT00000004639	ENSCJAG00000002421	TMPO
ENSCJAT00000004632	ENSCJAG00000002392	SLC25A3
ENSCJAT00000004633	ENSCJAG00000002392	SLC25A3
ENSCJAT00000004212	ENSCJAG00000002192	unknown
ENSCJAT00000004033	ENSCJAG00000002087	NR1H4
ENSCJAT00000004018	ENSCJAG00000002087	NR1H4
ENSCJAT00000003940	ENSCJAG00000002065	GAS2L3
ENSCJAT00000003736	ENSCJAG00000001913	UTP20
ENSCJAT00000027829	ENSCJAG00000014307	IGF1
ENSCJAT00000027842	ENSCJAG00000014307	IGF1
ENSCJAT00000027837	ENSCJAG00000014307	IGF1
ENSCJAT00000027879	ENSCJAG00000014328	PAH
ENSCJAT00000057332	ENSCJAG00000014328	PAH
ENSCJAT00000028173	ENSCJAG00000014455	HSP90B1
ENSCJAT00000028200	ENSCJAG00000014455	HSP90B1
ENSCJAT00000062941	ENSCJAG00000014455	HSP90B1
ENSCJAT00000028178	ENSCJAG00000014455	HSP90B1
ENSCJAT00000028333	ENSCJAG00000014542	GLT8D2
ENSCJAT00000060123	ENSCJAG00000014542	GLT8D2
ENSCJAT00000028565	ENSCJAG00000014635	TXNRD1
ENSCJAT00000028560	ENSCJAG00000014635	TXNRD1
ENSCJAT00000028569	ENSCJAG00000014635	TXNRD1
ENSCJAT00000028652	ENSCJAG00000014728	unknown
ENSCJAT00000062528	ENSCJAG00000014728	unknown
ENSCJAT00000029092	ENSCJAG00000014883	APPL2
ENSCJAT00000029090	ENSCJAG00000014883	APPL2
ENSCJAT00000029075	ENSCJAG00000014883	APPL2
ENSCJAT00000029121	ENSCJAG00000014948	NUAK1
ENSCJAT00000029120	ENSCJAG00000014948	NUAK1
ENSCJAT00000029158	ENSCJAG00000014977	TCP11L2
ENSCJAT00000056779	ENSCJAG00000014977	TCP11L2
ENSCJAT00000029382	ENSCJAG00000015061	RIC8B
ENSCJAT00000029377	ENSCJAG00000015061	RIC8B
ENSCJAT00000029371	ENSCJAG00000015061	RIC8B
ENSCJAT00000029379	ENSCJAG00000015061	RIC8B
ENSCJAT00000029702	ENSCJAG00000015164	PWP1
ENSCJAT00000029705	ENSCJAG00000015164	PWP1
ENSCJAT00000029674	ENSCJAG00000015164	PWP1
ENSCJAT00000029915	ENSCJAG00000015330	SART3
ENSCJAT00000029907	ENSCJAG00000015330	SART3
ENSCJAT00000058961	ENSCJAG00000015330	SART3
ENSCJAT00000053587	ENSCJAG00000015499	SVOP
ENSCJAT00000019776	ENSCJAG00000010157	USP30
ENSCJAT00000019675	ENSCJAG00000010041	unknown
ENSCJAT00000019661	ENSCJAG00000010041	unknown
ENSCJAT00000019541	ENSCJAG00000010041	unknown
ENSCJAT00000019421	ENSCJAG00000009995	MYO1H
ENSCJAT00000056732	ENSCJAG00000009995	MYO1H
ENSCJAT00000019174	ENSCJAG00000009881	MMAB
ENSCJAT00000019168	ENSCJAG00000009881	MMAB
ENSCJAT00000018891	ENSCJAG00000009739	TCHP
ENSCJAT00000018905	ENSCJAG00000009739	TCHP
ENSCJAT00000018495	ENSCJAG00000009518	IFT81

ENSCJAT00000063370	ENSCJAG00000009518	IFT81
ENSCJAT00000018244	ENSCJAG00000009364	ANAPC7
ENSCJAT00000018121	ENSCJAG00000009360	unknown
ENSCJAT00000052943	ENSCJAG00000009360	unknown
ENSCJAT00000017763	ENSCJAG00000009147	unknown
ENSCJAT00000022691	ENSCJAG00000011667	MYL2
ENSCJAT00000062236	ENSCJAG00000011944	ACAD10
ENSCJAT00000023224	ENSCJAG00000011944	ACAD10
ENSCJAT00000062384	ENSCJAG00000011944	ACAD10
ENSCJAT00000023521	ENSCJAG00000012112	ERP29
ENSCJAT00000023492	ENSCJAG00000012112	ERP29
ENSCJAT00000024467	ENSCJAG00000012624	OAS1
ENSCJAT00000061616	ENSCJAG00000012624	OAS1
ENSCJAT00000024477	ENSCJAG00000012624	OAS1
ENSCJAT00000024476	ENSCJAG00000012624	OAS1
ENSCJAT00000024542	ENSCJAG00000012666	OAS2
ENSCJAT00000019020	ENSCJAG00000009813	LHX5
ENSCJAT00000017791	ENSCJAG00000009188	unknown
ENSCJAT00000017713	ENSCJAG00000009139	HSPB8
ENSCJAT00000016828	ENSCJAG00000008639	PXN
ENSCJAT00000016788	ENSCJAG00000008631	SIRT4
ENSCJAT00000016776	ENSCJAG00000008624	PLA2G1B
ENSCJAT00000002702	ENSCJAG00000001436	P2RX4
ENSCJAT00000002813	ENSCJAG00000001488	ANAPC5
ENSCJAT00000063430	ENSCJAG00000001488	ANAPC5
ENSCJAT00000039946	ENSCJAG00000020340	unknown
ENSCJAT00000040134	ENSCJAG00000020419	RSRC2
ENSCJAT00000040104	ENSCJAG00000020419	RSRC2
ENSCJAT00000040214	ENSCJAG00000020485	HCAR1
ENSCJAT00000031911	ENSCJAG00000016370	HIP1R
ENSCJAT00000031907	ENSCJAG00000016370	HIP1R
ENSCJAT00000031996	ENSCJAG00000016428	ABCB9
ENSCJAT00000059511	ENSCJAG00000020466	TMED2
ENSCJAT00000040178	ENSCJAG00000020466	TMED2
ENSCJAT00000040180	ENSCJAG00000020466	TMED2
ENSCJAT00000040223	ENSCJAG00000020484	EIF2B1
ENSCJAT00000040296	ENSCJAG00000020528	ZNF664
ENSCJAT00000007130	ENSCJAG00000003720	PUS1
ENSCJAT00000007243	ENSCJAG00000003733	EP400
ENSCJAT00000025410	ENSCJAG00000013090	ZNF26
ENSCJAT00000022471	ENSCJAG00000011572	C16ORF33
ENSCJAT00000022794	ENSCJAG00000037284	unknown
ENSCJAT00000023819	ENSCJAG00000012292	GNG13
ENSCJAT00000025811	ENSCJAG00000033492	unknown
ENSCJAT00000012150	ENSCJAG00000006235	unknown
ENSCJAT00000028393	ENSCJAG00000014588	TMEM204
ENSCJAT00000028620	ENSCJAG00000014712	EME2
ENSCJAT00000028738	ENSCJAG00000014764	FAHD1
ENSCJAT00000028736	ENSCJAG00000014764	FAHD1
ENSCJAT00000029026	ENSCJAG00000014910	unknown
ENSCJAT00000029024	ENSCJAG00000014910	unknown
ENSCJAT00000029834	ENSCJAG00000015323	PRSS21
ENSCJAT00000029840	ENSCJAG00000015323	PRSS21
ENSCJAT00000030016	ENSCJAG00000015415	PKMYT1
ENSCJAT00000057565	ENSCJAG00000015462	CCDC64B
ENSCJAT00000058402	ENSCJAG00000015462	CCDC64B
ENSCJAT00000030103	ENSCJAG00000015462	CCDC64B
ENSCJAT00000038440	ENSCJAG00000019582	MEFV
ENSCJAT00000033552	ENSCJAG00000019582	MEFV

ENSCJAT00000038429	ENSCJAG00000019582	MEFV
ENSCJAT00000038469	ENSCJAG00000036662	ZNF75A
ENSCJAT00000038626	ENSCJAG00000019687	DNASE1
ENSCJAT00000038773	ENSCJAG00000019750	SRL
ENSCJAT00000038780	ENSCJAG00000019750	SRL
ENSCJAT00000038809	ENSCJAG00000019756	TFAP4
ENSCJAT00000038930	ENSCJAG00000019832	NMRAL1
ENSCJAT00000038937	ENSCJAG00000019834	HMOX2
ENSCJAT00000059999	ENSCJAG00000019834	HMOX2
ENSCJAT00000039200	ENSCJAG00000019945	UBN1
ENSCJAT00000039199	ENSCJAG00000019945	UBN1
ENSCJAT00000033753	ENSCJAG00000017349	unknown
ENSCJAT00000010605	ENSCJAG00000005426	KIAA0430
ENSCJAT00000010436	ENSCJAG00000005411	unknown
ENSCJAT00000010190	ENSCJAG00000005268	unknown
ENSCJAT00000030860	ENSCJAG00000015851	BFAR
ENSCJAT00000030856	ENSCJAG00000015851	BFAR
ENSCJAT00000030850	ENSCJAG00000015851	BFAR
ENSCJAT00000031067	ENSCJAG00000015925	MKL2
ENSCJAT00000016324	ENSCJAG00000008375	unknown
ENSCJAT00000052478	ENSCJAG00000008375	unknown
ENSCJAT00000016317	ENSCJAG00000008375	unknown
ENSCJAT00000063349	ENSCJAG00000008403	ZC3H7A
ENSCJAT00000063124	ENSCJAG00000008403	ZC3H7A
ENSCJAT00000031216	ENSCJAG00000016057	LITAF
ENSCJAT00000031214	ENSCJAG00000016057	LITAF
ENSCJAT00000031210	ENSCJAG00000016054	unknown
ENSCJAT00000055532	ENSCJAG00000015997	CIITA
ENSCJAT00000031146	ENSCJAG00000015997	CIITA
ENSCJAT00000031104	ENSCJAG00000015978	NUBP1
ENSCJAT00000031076	ENSCJAG00000015978	NUBP1
ENSCJAT00000031095	ENSCJAG00000015978	NUBP1
ENSCJAT00000018599	ENSCJAG00000009595	GPR139
ENSCJAT00000018578	ENSCJAG00000009544	UMOD
ENSCJAT00000018567	ENSCJAG00000009544	UMOD
ENSCJAT00000018574	ENSCJAG00000009544	UMOD
ENSCJAT00000017497	ENSCJAG00000008918	POLR3E
ENSCJAT00000040414	ENSCJAG00000020582	HS3ST2
ENSCJAT00000040435	ENSCJAG00000020591	SCNN1G
ENSCJAT00000040486	ENSCJAG00000020616	NDUFAB1
ENSCJAT00000040725	ENSCJAG00000020698	ARHGAP17
ENSCJAT00000040726	ENSCJAG00000020698	ARHGAP17
ENSCJAT00000040701	ENSCJAG00000020698	ARHGAP17
ENSCJAT00000040686	ENSCJAG00000020698	ARHGAP17
ENSCJAT00000011514	ENSCJAG00000005921	IL4R
ENSCJAT00000011561	ENSCJAG00000005950	GTF3C1
ENSCJAT00000011558	ENSCJAG00000005950	GTF3C1
ENSCJAT00000011655	ENSCJAG00000006003	GSG1L
ENSCJAT00000061786	ENSCJAG00000033767	LAT
ENSCJAT00000012906	ENSCJAG00000006585	unknown
ENSCJAT00000012866	ENSCJAG00000006585	unknown
ENSCJAT00000012929	ENSCJAG00000006585	unknown
ENSCJAT00000012886	ENSCJAG00000006585	unknown
ENSCJAT00000013312	ENSCJAG00000006809	GDPD3
ENSCJAT00000055668	ENSCJAG00000006809	GDPD3
ENSCJAT00000059707	ENSCJAG00000006809	GDPD3
ENSCJAT00000063222	ENSCJAG00000006809	GDPD3
ENSCJAT00000013339	ENSCJAG00000035904	TBX6
ENSCJAT00000013347	ENSCJAG00000035904	TBX6

ENSCJAT00000013615	ENSCJAG00000006954	INO80E
ENSCJAT00000013607	ENSCJAG00000006954	INO80E
ENSCJAT00000013592	ENSCJAG00000006954	INO80E
ENSCJAT00000026360	ENSCJAG00000006995	TAOK2
ENSCJAT00000013871	ENSCJAG00000007082	TMEM219
ENSCJAT00000013895	ENSCJAG00000007098	KCTD13
ENSCJAT00000052196	ENSCJAG00000033727	unknown
ENSCJAT00000060738	ENSCJAG00000007155	CDIPT
ENSCJAT00000056611	ENSCJAG00000007155	CDIPT
ENSCJAT00000014025	ENSCJAG00000007155	CDIPT
ENSCJAT00000062620	ENSCJAG00000007155	CDIPT
ENSCJAT00000059506	ENSCJAG00000007166	MVP
ENSCJAT00000014057	ENSCJAG00000007166	MVP
ENSCJAT00000014050	ENSCJAG00000007166	MVP
ENSCJAT00000063173	ENSCJAG00000007166	MVP
ENSCJAT00000058669	ENSCJAG00000002878	RNF40
ENSCJAT00000032025	ENSCJAG00000002878	RNF40
ENSCJAT00000005591	ENSCJAG00000002878	RNF40
ENSCJAT00000061216	ENSCJAG00000002878	RNF40
ENSCJAT00000005606	ENSCJAG00000002878	RNF40
ENSCJAT00000005624	ENSCJAG00000002878	RNF40
ENSCJAT00000054681	ENSCJAG00000002878	RNF40
ENSCJAT00000005612	ENSCJAG00000002878	RNF40
ENSCJAT00000005564	ENSCJAG00000002878	RNF40
ENSCJAT00000005806	ENSCJAG00000003030	HSD3B7
ENSCJAT00000006001	ENSCJAG00000003122	KAT8
ENSCJAT00000005995	ENSCJAG00000003122	KAT8
ENSCJAT00000031426	ENSCJAG00000016155	unknown
ENSCJAT00000003578	ENSCJAG00000016273	unknown
ENSCJAT00000031646	ENSCJAG00000016273	unknown
ENSCJAT00000031671	ENSCJAG00000016280	ZNF485
ENSCJAT00000031677	ENSCJAG00000016280	ZNF485
ENSCJAT00000056256	ENSCJAG00000016280	ZNF485
ENSCJAT00000031714	ENSCJAG00000016304	CXCL12
ENSCJAT00000026038	ENSCJAG00000013406	ALOX5
ENSCJAT00000026002	ENSCJAG00000013387	unknown
ENSCJAT00000025978	ENSCJAG00000013377	MSMB
ENSCJAT00000025971	ENSCJAG00000013352	unknown
ENSCJAT00000058224	ENSCJAG00000013352	unknown
ENSCJAT00000055983	ENSCJAG00000013484	C10orf71
ENSCJAT00000026201	ENSCJAG00000013484	C10orf71
ENSCJAT00000026215	ENSCJAG00000013489	C10orf128
ENSCJAT00000053398	ENSCJAG00000013528	WDFY4
ENSCJAT00000054179	ENSCJAG00000013661	IRBP
ENSCJAT00000022837	ENSCJAG00000011770	unknown
ENSCJAT00000022861	ENSCJAG00000011770	unknown
ENSCJAT00000022864	ENSCJAG00000011788	GPRIN2
ENSCJAT00000029849	ENSCJAG00000015338	RGR
ENSCJAT00000029860	ENSCJAG00000015338	RGR
ENSCJAT00000006541	ENSCJAG00000003404	SH2D4B
ENSCJAT00000001812	ENSCJAG00000000976	ZMIZ1
ENSCJAT00000002871	ENSCJAG00000001521	SEC24C
ENSCJAT00000003035	ENSCJAG00000001592	MYOZ1
ENSCJAT00000003096	ENSCJAG00000001615	USP54
ENSCJAT00000003074	ENSCJAG00000001615	USP54
ENSCJAT00000003084	ENSCJAG00000001615	USP54
ENSCJAT00000003235	ENSCJAG00000001711	MSS51
ENSCJAT00000056417	ENSCJAG00000007511	ECD
ENSCJAT00000014721	ENSCJAG00000007511	ECD

ENSCJAT00000061668	ENSCJAG00000007511	ECD
ENSCJAT00000014661	ENSCJAG00000007475	NUDT13
ENSCJAT00000014696	ENSCJAG00000007475	NUDT13
ENSCJAT00000014691	ENSCJAG00000007475	NUDT13
ENSCJAT00000014626	ENSCJAG00000007471	unknown
ENSCJAT00000014546	ENSCJAG00000007420	PLA2G12B
ENSCJAT00000014525	ENSCJAG00000007405	OIT3
ENSCJAT00000014515	ENSCJAG00000007405	OIT3
ENSCJAT00000014215	ENSCJAG00000007252	SPOCK2
ENSCJAT00000014252	ENSCJAG00000007252	SPOCK2
ENSCJAT00000014143	ENSCJAG00000007212	PSAP
ENSCJAT00000031689	ENSCJAG00000016288	NODAL
ENSCJAT00000031793	ENSCJAG00000016344	NPFFR1
ENSCJAT00000031775	ENSCJAG00000016308	unknown
ENSCJAT00000031846	ENSCJAG00000016374	TYSND1
ENSCJAT00000032059	ENSCJAG00000016486	TSPAN15
ENSCJAT00000032064	ENSCJAG00000016486	TSPAN15
ENSCJAT00000025232	ENSCJAG00000012961	HKDC1
ENSCJAT00000025067	ENSCJAG00000012907	VPS26A
ENSCJAT00000025050	ENSCJAG00000012907	VPS26A
ENSCJAT00000025062	ENSCJAG00000012907	VPS26A
ENSCJAT00000063224	ENSCJAG00000012907	VPS26A
ENSCJAT00000025051	ENSCJAG00000012907	VPS26A
ENSCJAT00000025001	ENSCJAG00000012896	KIAA1279
ENSCJAT00000037047	ENSCJAG00000018844	DDX50
ENSCJAT00000024979	ENSCJAG00000018844	DDX50
ENSCJAT00000014018	ENSCJAG00000007142	unknown
ENSCJAT00000014003	ENSCJAG00000007142	unknown
ENSCJAT00000013959	ENSCJAG00000007123	EGR2
ENSCJAT00000013942	ENSCJAG00000007123	EGR2
ENSCJAT00000013932	ENSCJAG00000007119	unknown
ENSCJAT00000013878	ENSCJAG00000007086	RTKN2
ENSCJAT00000013712	ENSCJAG00000007001	CDK1
ENSCJAT00000054308	ENSCJAG00000006912	ANK3
ENSCJAT00000020819	ENSCJAG00000010694	unknown
ENSCJAT00000020699	ENSCJAG00000010625	TFAM
ENSCJAT00000020682	ENSCJAG00000010573	UBE2D1
ENSCJAT00000020591	ENSCJAG00000010565	CISD1
ENSCJAT00000057789	ENSCJAG00000005045	A1CF
ENSCJAT00000009875	ENSCJAG00000005045	A1CF
ENSCJAT00000062687	ENSCJAG00000005045	A1CF
ENSCJAT00000054389	ENSCJAG00000005045	A1CF
ENSCJAT00000010015	ENSCJAG00000005129	unknown
ENSCJAT00000053492	ENSCJAG00000005129	unknown
ENSCJAT00000010025	ENSCJAG00000005129	unknown
ENSCJAT00000010250	ENSCJAG00000005296	ATAD1
ENSCJAT00000010265	ENSCJAG00000005296	ATAD1
ENSCJAT00000010408	ENSCJAG00000005360	LIPF
ENSCJAT00000010420	ENSCJAG00000005360	LIPF
ENSCJAT00000010555	ENSCJAG00000005454	LIPM
ENSCJAT00000055974	ENSCJAG00000008509	5-Mar
ENSCJAT00000053188	ENSCJAG00000008515	IDE
ENSCJAT00000016714	ENSCJAG00000008515	IDE
ENSCJAT00000016582	ENSCJAG00000008515	IDE
ENSCJAT00000016785	ENSCJAG00000008627	HHEX
ENSCJAT00000016779	ENSCJAG00000008627	HHEX
ENSCJAT00000017750	ENSCJAG00000009157	FFAR4
ENSCJAT00000017748	ENSCJAG00000009157	FFAR4
ENSCJAT00000030330	ENSCJAG00000015574	FRA10AC1

ENSCJAT00000062514	ENSCJAG00000015911	unknown
ENSCJAT00000031075	ENSCJAG00000015969	unknown
ENSCJAT00000031078	ENSCJAG00000015969	unknown
ENSCJAT00000031571	ENSCJAG00000016206	TCTN3
ENSCJAT00000058735	ENSCJAG00000016206	TCTN3
ENSCJAT00000055989	ENSCJAG00000016206	TCTN3
ENSCJAT00000053973	ENSCJAG00000016206	TCTN3
ENSCJAT00000032049	ENSCJAG00000016474	PIK3AP1
ENSCJAT00000032068	ENSCJAG00000016484	LCOR
ENSCJAT00000024811	ENSCJAG00000016484	LCOR
ENSCJAT00000056879	ENSCJAG00000016484	LCOR
ENSCJAT00000032538	ENSCJAG00000016726	ANKRD2
ENSCJAT00000032541	ENSCJAG00000016726	ANKRD2
ENSCJAT00000060232	ENSCJAG00000016755	PI4K2A
ENSCJAT00000032594	ENSCJAG00000016755	PI4K2A
ENSCJAT00000032612	ENSCJAG00000016772	AVPI1
ENSCJAT00000052935	ENSCJAG00000016949	HPS1
ENSCJAT00000032968	ENSCJAG00000016949	HPS1
ENSCJAT00000061178	ENSCJAG00000016949	HPS1
ENSCJAT00000033287	ENSCJAG00000017121	CUTC
ENSCJAT00000052646	ENSCJAG00000017121	CUTC
ENSCJAT00000057339	ENSCJAG00000017269	BLOC1S2
ENSCJAT00000033592	ENSCJAG00000017269	BLOC1S2
ENSCJAT00000034020	ENSCJAG00000017458	SEMA4G
ENSCJAT00000034013	ENSCJAG00000017458	SEMA4G
ENSCJAT00000034036	ENSCJAG00000017480	MRPL43
ENSCJAT00000034030	ENSCJAG00000017480	MRPL43
ENSCJAT00000034075	ENSCJAG00000017496	LZTS2
ENSCJAT00000034078	ENSCJAG00000017496	LZTS2
ENSCJAT00000054358	ENSCJAG00000017496	LZTS2
ENSCJAT00000034290	ENSCJAG00000017600	POLL
ENSCJAT00000061717	ENSCJAG00000017600	POLL
ENSCJAT00000060733	ENSCJAG00000017600	POLL
ENSCJAT00000034305	ENSCJAG00000017614	DPCD
ENSCJAT00000034297	ENSCJAG00000017614	DPCD
ENSCJAT00000034313	ENSCJAG00000017614	DPCD
ENSCJAT00000054672	ENSCJAG00000017627	FBXW4
ENSCJAT00000034571	ENSCJAG00000017676	KCNIP2
ENSCJAT00000034705	ENSCJAG00000017777	LDB1
ENSCJAT00000034696	ENSCJAG00000017777	LDB1
ENSCJAT00000021455	ENSCJAG00000017928	NFKB2
ENSCJAT00000035539	ENSCJAG00000018178	unknown
ENSCJAT00000053510	ENSCJAG00000018178	unknown
ENSCJAT00000035688	ENSCJAG00000018253	unknown
ENSCJAT00000035774	ENSCJAG00000018295	CALHM3
ENSCJAT00000036327	ENSCJAG00000018526	SORCS1
ENSCJAT00000037132	ENSCJAG00000018889	GPAM
ENSCJAT00000037174	ENSCJAG00000018943	ACSL5
ENSCJAT00000037177	ENSCJAG00000018943	ACSL5
ENSCJAT00000063780	ENSCJAG00000019284	VWA2
ENSCJAT00000037806	ENSCJAG00000019284	VWA2
ENSCJAT00000037803	ENSCJAG00000019284	VWA2
ENSCJAT00000037791	ENSCJAG00000019284	VWA2
ENSCJAT00000038102	ENSCJAG00000019429	PNLIPRP2
ENSCJAT00000038132	ENSCJAG00000019440	HSPA12A
ENSCJAT00000038256	ENSCJAG00000019505	KCNK18
ENSCJAT00000038299	ENSCJAG00000019511	SLC18A2
ENSCJAT00000038307	ENSCJAG00000019527	PDZD8
ENSCJAT00000061145	ENSCJAG00000019527	PDZD8

ENSCJAT00000038387	ENSCJAG00000019575	PRLHR
ENSCJAT00000038390	ENSCJAG00000019575	PRLHR
ENSCJAT00000038416	ENSCJAG00000019589	NANOS1
ENSCJAT00000038455	ENSCJAG00000019595	EIF3A
ENSCJAT00000038445	ENSCJAG00000019595	EIF3A
ENSCJAT00000060223	ENSCJAG00000019595	EIF3A
ENSCJAT00000059498	ENSCJAG00000034798	SFXN4
ENSCJAT00000012693	ENSCJAG00000006430	WDR11
ENSCJAT00000053205	ENSCJAG00000006430	WDR11
ENSCJAT00000012527	ENSCJAG00000006430	WDR11
ENSCJAT00000002530	ENSCJAG00000001289	TACC2
ENSCJAT00000056492	ENSCJAG00000001117	PSTK
ENSCJAT00000002033	ENSCJAG00000001117	PSTK
ENSCJAT00000062847	ENSCJAG00000001092	ACADSB
ENSCJAT00000061986	ENSCJAG00000001092	ACADSB
ENSCJAT00000062779	ENSCJAG00000012593	METTL10
ENSCJAT00000024409	ENSCJAG00000012593	METTL10
ENSCJAT00000024401	ENSCJAG00000012593	METTL10
ENSCJAT00000024395	ENSCJAG00000012593	METTL10
ENSCJAT00000061054	ENSCJAG00000012581	FAM175B
ENSCJAT00000024373	ENSCJAG00000012581	FAM175B
ENSCJAT00000056946	ENSCJAG00000012481	EDRF1
ENSCJAT00000024238	ENSCJAG00000012481	EDRF1
ENSCJAT00000024096	ENSCJAG00000012411	BCCIP
ENSCJAT00000024110	ENSCJAG00000012411	BCCIP
ENSCJAT00000024094	ENSCJAG00000012411	BCCIP
ENSCJAT00000024075	ENSCJAG00000012411	BCCIP
ENSCJAT00000023174	ENSCJAG00000011914	FBXO25
ENSCJAT00000008757	ENSCJAG00000004569	ARHGEF10
ENSCJAT00000008826	ENSCJAG00000004597	KBTBD11
ENSCJAT00000054757	ENSCJAG00000000204	unknown
ENSCJAT00000007443	ENSCJAG00000003877	DEFB1
ENSCJAT00000007470	ENSCJAG00000003893	unknown
ENSCJAT00000065988	ENSCJAG00000004032	GATA4
ENSCJAT00000007775	ENSCJAG00000004032	GATA4
ENSCJAT00000007987	ENSCJAG00000004167	XKR6
ENSCJAT00000057096	ENSCJAG00000004168	unknown
ENSCJAT00000008007	ENSCJAG00000004168	unknown
ENSCJAT00000008011	ENSCJAG00000004168	unknown
ENSCJAT00000008056	ENSCJAG00000004193	PRSS55
ENSCJAT00000008040	ENSCJAG00000004193	PRSS55
ENSCJAT00000038614	ENSCJAG00000019677	EPHX2
ENSCJAT00000058116	ENSCJAG00000019884	BNIP3L
ENSCJAT00000039074	ENSCJAG00000019884	BNIP3L
ENSCJAT00000039064	ENSCJAG00000019884	BNIP3L
ENSCJAT00000039213	ENSCJAG00000019958	CDCA2
ENSCJAT00000039219	ENSCJAG00000019958	CDCA2
ENSCJAT00000039388	ENSCJAG00000020047	NEFM
ENSCJAT00000039384	ENSCJAG00000020047	NEFM
ENSCJAT00000056273	ENSCJAG00000020135	unknown
ENSCJAT00000039552	ENSCJAG00000020135	unknown
ENSCJAT00000039549	ENSCJAG00000020135	unknown
ENSCJAT00000039626	ENSCJAG00000020166	unknown
ENSCJAT00000039624	ENSCJAG00000020166	unknown
ENSCJAT00000039708	ENSCJAG00000020218	unknown
ENSCJAT00000039712	ENSCJAG00000020218	unknown
ENSCJAT00000051001	ENSCJAG00000020290	BMP1
ENSCJAT00000039901	ENSCJAG00000020317	LGI3
ENSCJAT00000027926	ENSCJAG00000014346	LPL

ENSCJAT00000027565	ENSCJAG00000014144	ASAH1
ENSCJAT00000027575	ENSCJAG00000014144	ASAH1
ENSCJAT00000027566	ENSCJAG00000014144	ASAH1
ENSCJAT00000022574	ENSCJAG00000011583	MTUS1
ENSCJAT00000022554	ENSCJAG00000011583	MTUS1
ENSCJAT00000022720	ENSCJAG00000011665	SLC7A2
ENSCJAT00000022739	ENSCJAG00000011665	SLC7A2
ENSCJAT00000022834	ENSCJAG00000011725	MTMR7
ENSCJAT00000022827	ENSCJAG00000011725	MTMR7
ENSCJAT00000022865	ENSCJAG00000011769	VPS37A
ENSCJAT00000022875	ENSCJAG00000011769	VPS37A
ENSCJAT00000062107	ENSCJAG00000011795	CNOT7
ENSCJAT00000022924	ENSCJAG00000011795	CNOT7
ENSCJAT00000023383	ENSCJAG00000012014	DLC1
ENSCJAT00000043741	ENSCJAG00000022756	PPP1R3B
ENSCJAT00000023928	ENSCJAG00000012338	DCTN6
ENSCJAT00000023975	ENSCJAG00000012366	RBPMS
ENSCJAT00000024030	ENSCJAG00000012399	GTF2E2
ENSCJAT00000005287	ENSCJAG00000002756	PROSC
ENSCJAT00000005025	ENSCJAG00000002619	BAG4
ENSCJAT00000005016	ENSCJAG00000002619	BAG4
ENSCJAT00000004931	ENSCJAG00000002560	PPAPDC1B
ENSCJAT00000004659	ENSCJAG00000002394	FGFR1
ENSCJAT00000004540	ENSCJAG00000002356	TACC1
ENSCJAT00000004223	ENSCJAG00000002206	unknown
ENSCJAT00000004237	ENSCJAG00000002206	unknown
ENSCJAT00000008999	ENSCJAG00000011099	GIPC1
ENSCJAT00000021633	ENSCJAG00000011099	GIPC1
ENSCJAT00000021589	ENSCJAG00000011066	SFRP1
ENSCJAT00000015032	ENSCJAG00000007684	CHRNA6
ENSCJAT00000014990	ENSCJAG00000007684	CHRNA6
ENSCJAT00000015156	ENSCJAG00000007751	AP3M2
ENSCJAT00000057562	ENSCJAG00000007751	AP3M2
ENSCJAT00000055689	ENSCJAG00000008061	SMIM19
ENSCJAT00000055645	ENSCJAG00000008965	RNMT
ENSCJAT00000063489	ENSCJAG00000008965	RNMT
ENSCJAT00000017397	ENSCJAG00000008965	RNMT
ENSCJAT00000058392	ENSCJAG00000008965	RNMT
ENSCJAT00000009784	ENSCJAG00000005028	CEP76
ENSCJAT00000009765	ENSCJAG00000005028	CEP76
ENSCJAT00000059226	ENSCJAG00000004552	VAPA
ENSCJAT00000008766	ENSCJAG00000004552	VAPA
ENSCJAT00000008760	ENSCJAG00000004552	VAPA
ENSCJAT00000008489	ENSCJAG00000004430	RALBP1
ENSCJAT00000008332	ENSCJAG00000004307	NDUFV2
ENSCJAT00000008206	ENSCJAG00000004279	MTCL1
ENSCJAT00000030558	ENSCJAG00000015677	AURKB
ENSCJAT00000030561	ENSCJAG00000015677	AURKB
ENSCJAT00000052462	ENSCJAG00000015677	AURKB
ENSCJAT00000061181	ENSCJAG00000015677	AURKB
ENSCJAT00000025078	ENSCJAG00000012917	MYOM1
ENSCJAT00000025090	ENSCJAG00000012917	MYOM1
ENSCJAT00000025110	ENSCJAG00000012946	LPIN2
ENSCJAT00000000165	ENSCJAG00000000078	NDC80
ENSCJAT00000040322	ENSCJAG00000020543	GATA6
ENSCJAT00000040319	ENSCJAG00000020543	GATA6
ENSCJAT00000040366	ENSCJAG00000020556	RIOK3
ENSCJAT00000040472	ENSCJAG00000020598	LAMA3
ENSCJAT00000040543	ENSCJAG00000020641	unknown

ENSCJAT00000040547	ENSCJAG00000020641	unknown
ENSCJAT00000040603	ENSCJAG00000020648	OSBPL1A
ENSCJAT00000040589	ENSCJAG00000020648	OSBPL1A
ENSCJAT00000040733	ENSCJAG00000020728	KCTD1
ENSCJAT00000040825	ENSCJAG00000020767	DSC2
ENSCJAT00000040822	ENSCJAG00000020767	DSC2
ENSCJAT00000040827	ENSCJAG00000020767	DSC2
ENSCJAT00000040844	ENSCJAG00000020778	DSG1
ENSCJAT00000040864	ENSCJAG00000020783	DSG4
ENSCJAT00000040881	ENSCJAG00000020796	DSG3
ENSCJAT00000058326	ENSCJAG00000020804	DSG2
ENSCJAT00000040890	ENSCJAG00000020804	DSG2
ENSCJAT00000040889	ENSCJAG00000020804	DSG2
ENSCJAT00000041340	ENSCJAG00000021025	INO80C
ENSCJAT00000041539	ENSCJAG00000021123	TPGS2
ENSCJAT00000041535	ENSCJAG00000021123	TPGS2
ENSCJAT00000016726	ENSCJAG00000008484	EPG5
ENSCJAT00000016047	ENSCJAG00000008221	LOXHD1
ENSCJAT00000016072	ENSCJAG00000008221	LOXHD1
ENSCJAT00000009041	ENSCJAG00000004704	ZBTB7C
ENSCJAT00000016688	ENSCJAG00000008555	SKA1
ENSCJAT00000055027	ENSCJAG00000034626	unknown
ENSCJAT00000052640	ENSCJAG00000001841	C18orf54
ENSCJAT00000007739	ENSCJAG00000004012	TXNL1
ENSCJAT00000006718	ENSCJAG00000003500	CPLX4
ENSCJAT00000005501	ENSCJAG00000002859	unknown
ENSCJAT00000052479	ENSCJAG00000002877	SERPINB8
ENSCJAT00000005528	ENSCJAG00000002877	SERPINB8
ENSCJAT00000005531	ENSCJAG00000002877	SERPINB8
ENSCJAT00000006055	ENSCJAG00000003151	FBXO15
ENSCJAT00000015605	ENSCJAG00000007988	GALR1
ENSCJAT00000037067	ENSCJAG00000018883	KCNIP3
ENSCJAT00000036805	ENSCJAG00000018757	ASTL
ENSCJAT00000014996	ENSCJAG00000007660	unknown
ENSCJAT00000014992	ENSCJAG00000007660	unknown
ENSCJAT00000015138	ENSCJAG00000007688	KANSL3
ENSCJAT00000059948	ENSCJAG00000007688	KANSL3
ENSCJAT00000015101	ENSCJAG00000007688	KANSL3
ENSCJAT00000015134	ENSCJAG00000007688	KANSL3
ENSCJAT00000053066	ENSCJAG00000007688	KANSL3
ENSCJAT00000058291	ENSCJAG00000007901	LMAN2L
ENSCJAT00000015495	ENSCJAG00000007901	LMAN2L
ENSCJAT00000015500	ENSCJAG00000007901	LMAN2L
ENSCJAT00000056085	ENSCJAG00000007901	LMAN2L
ENSCJAT00000054047	ENSCJAG00000007901	LMAN2L
ENSCJAT00000058359	ENSCJAG00000007949	CNNM4
ENSCJAT00000054160	ENSCJAG00000007949	CNNM4
ENSCJAT00000015518	ENSCJAG00000007949	CNNM4
ENSCJAT00000013981	ENSCJAG00000007139	COA5
ENSCJAT00000013840	ENSCJAG00000007074	LYG2
ENSCJAT00000013848	ENSCJAG00000007074	LYG2
ENSCJAT00000019743	ENSCJAG00000010160	PDCL3
ENSCJAT00000016362	ENSCJAG00000008399	IL1R2
ENSCJAT00000054123	ENSCJAG00000008399	IL1R2
ENSCJAT00000016171	ENSCJAG00000008271	IL18R1
ENSCJAT00000060233	ENSCJAG00000008255	IL18RAP
ENSCJAT00000057656	ENSCJAG00000008193	SLC9A2
ENSCJAT00000015935	ENSCJAG00000035523	TMEM182
ENSCJAT00000060577	ENSCJAG00000008156	unknown
ENSCJAT00000015891	ENSCJAG00000008156	unknown
ENSCJAT00000039743	ENSCJAG00000037169	SULT1C3
ENSCJAT00000034621	ENSCJAG00000017754	10-Sep
ENSCJAT00000029736	ENSCJAG00000015268	unknown
ENSCJAT00000026251	ENSCJAG00000013468	BUB1
ENSCJAT00000008482	ENSCJAG00000004435	unknown
ENSCJAT00000032143	ENSCJAG00000016518	TMEM87B
ENSCJAT00000062854	ENSCJAG00000016479	ZC3H6
ENSCJAT00000032063	ENSCJAG00000016479	ZC3H6
ENSCJAT00000027067	ENSCJAG00000013930	IL1RN
ENSCJAT00000037956	ENSCJAG00000019362	KRCC1
ENSCJAT00000038056	ENSCJAG00000037995	RNF103
ENSCJAT00000024269	ENSCJAG00000019414	KDM3A
ENSCJAT00000052905	ENSCJAG00000019414	KDM3A
ENSCJAT00000038138	ENSCJAG00000019414	KDM3A
ENSCJAT00000038150	ENSCJAG00000019414	KDM3A
ENSCJAT00000038146	ENSCJAG00000019414	KDM3A
ENSCJAT00000038194	ENSCJAG00000019467	unknown
ENSCJAT00000038185	ENSCJAG00000019467	unknown
ENSCJAT00000038337	ENSCJAG00000019523	PTCD3
ENSCJAT00000038334	ENSCJAG00000019523	PTCD3
ENSCJAT00000038308	ENSCJAG00000019523	PTCD3
ENSCJAT00000038487	ENSCJAG00000019594	USP39
ENSCJAT00000058528	ENSCJAG00000019594	USP39
ENSCJAT00000063784	ENSCJAG00000019594	USP39
ENSCJAT00000054338	ENSCJAG00000019594	USP39
ENSCJAT00000058520	ENSCJAG00000019594	USP39
ENSCJAT00000058777	ENSCJAG00000019594	USP39
ENSCJAT00000032954	ENSCJAG00000019657	GGCX
ENSCJAT00000038593	ENSCJAG00000019657	GGCX
ENSCJAT00000038715	ENSCJAG00000019726	RETSAT
ENSCJAT00000038713	ENSCJAG00000019726	RETSAT
ENSCJAT00000060677	ENSCJAG00000019726	RETSAT
ENSCJAT00000033324	ENSCJAG00000017132	TACR1
ENSCJAT00000033318	ENSCJAG00000017132	TACR1
ENSCJAT00000025226	ENSCJAG00000017161	HK2
ENSCJAT00000033418	ENSCJAG00000017161	HK2
ENSCJAT00000062397	ENSCJAG00000017577	C2orf81
ENSCJAT00000034795	ENSCJAG00000017844	BOLA3
ENSCJAT00000061566	ENSCJAG00000004144	ALMS1
ENSCJAT00000007946	ENSCJAG00000004136	EGR4
ENSCJAT00000007940	ENSCJAG00000004136	EGR4
ENSCJAT00000007890	ENSCJAG00000003978	CCT7
ENSCJAT00000060463	ENSCJAG00000003978	CCT7
ENSCJAT00000053269	ENSCJAG00000003978	CCT7
ENSCJAT00000007893	ENSCJAG00000003978	CCT7
ENSCJAT00000010492	ENSCJAG00000003978	CCT7
ENSCJAT00000062180	ENSCJAG00000003978	CCT7
ENSCJAT00000007586	ENSCJAG00000003959	NOTO
ENSCJAT00000007429	ENSCJAG00000003870	SPR
ENSCJAT00000058081	ENSCJAG00000003816	CYP26B1
ENSCJAT00000038023	ENSCJAG00000019383	ATP6V1B1
ENSCJAT00000062729	ENSCJAG00000019383	ATP6V1B1
ENSCJAT00000037976	ENSCJAG00000019371	CD207
ENSCJAT00000057117	ENSCJAG00000019358	unknown
ENSCJAT00000037955	ENSCJAG00000019358	unknown
ENSCJAT00000062247	ENSCJAG00000019256	SNRNP27
ENSCJAT00000059382	ENSCJAG00000019266	MXD1
ENSCJAT00000037770	ENSCJAG00000019266	MXD1

ENSCJAT00000037386	ENSCJAG00000019067	PROKR1
ENSCJAT00000012813	ENSCJAG00000006564	unknown
ENSCJAT00000064503	ENSCJAG00000035534	unknown
ENSCJAT00000001455	ENSCJAG00000009029	KIAA1841
ENSCJAT00000056339	ENSCJAG00000009029	KIAA1841
ENSCJAT00000017501	ENSCJAG00000009029	KIAA1841
ENSCJAT00000017518	ENSCJAG00000009037	PEX13
ENSCJAT00000017389	ENSCJAG00000008975	REL
ENSCJAT00000017394	ENSCJAG00000008975	REL
ENSCJAT00000004594	ENSCJAG00000009299	VRK2
ENSCJAT00000061532	ENSCJAG00000009299	VRK2
ENSCJAT00000018053	ENSCJAG00000009299	VRK2
ENSCJAT00000018042	ENSCJAG00000009299	VRK2
ENSCJAT00000018741	ENSCJAG00000009663	CCDC85A
ENSCJAT00000018756	ENSCJAG00000009663	CCDC85A
ENSCJAT00000057596	ENSCJAG00000009740	PNPT1
ENSCJAT00000034912	ENSCJAG00000009891	CCDC88A
ENSCJAT00000056718	ENSCJAG00000009891	CCDC88A
ENSCJAT00000019600	ENSCJAG00000010079	RTN4
ENSCJAT00000019596	ENSCJAG00000010079	RTN4
ENSCJAT00000019617	ENSCJAG00000010079	RTN4
ENSCJAT00000056985	ENSCJAG00000010079	RTN4
ENSCJAT00000058109	ENSCJAG00000010079	RTN4
ENSCJAT00000060729	ENSCJAG00000010079	RTN4
ENSCJAT00000019593	ENSCJAG00000010079	RTN4
ENSCJAT00000019612	ENSCJAG00000010079	RTN4
ENSCJAT00000056866	ENSCJAG00000010600	CHAC2
ENSCJAT00000020653	ENSCJAG00000010600	CHAC2
ENSCJAT00000053931	ENSCJAG00000010640	FSHR
ENSCJAT00000001131	ENSCJAG00000000614	KCNK12
ENSCJAT00000056844	ENSCJAG00000031938	unknown
ENSCJAT00000001247	ENSCJAG00000000680	PIGF
ENSCJAT00000001249	ENSCJAG00000000680	PIGF
ENSCJAT00000001263	ENSCJAG00000000685	RHOQ
ENSCJAT00000052350	ENSCJAG00000000685	RHOQ
ENSCJAT00000001526	ENSCJAG00000000833	ABCG8
ENSCJAT00000028365	ENSCJAG00000014571	ZFP36L2
ENSCJAT00000029028	ENSCJAG00000014891	THUMPD2
ENSCJAT00000059971	ENSCJAG00000015185	SRSF7
ENSCJAT00000029648	ENSCJAG00000015185	SRSF7
ENSCJAT00000060023	ENSCJAG00000015185	SRSF7
ENSCJAT00000059765	ENSCJAG00000015185	SRSF7
ENSCJAT00000007653	ENSCJAG00000003980	QPCT
ENSCJAT00000007536	ENSCJAG00000003858	CEBPZ
ENSCJAT00000007518	ENSCJAG00000003858	CEBPZ
ENSCJAT00000007403	ENSCJAG00000003850	SULT6B1
ENSCJAT00000007380	ENSCJAG00000003819	EIF2AK2
ENSCJAT00000007372	ENSCJAG00000003819	EIF2AK2
ENSCJAT00000007377	ENSCJAG00000003819	EIF2AK2
ENSCJAT00000006225	ENSCJAG00000003245	unknown
ENSCJAT00000006220	ENSCJAG00000003245	unknown
ENSCJAT00000006153	ENSCJAG00000003194	YPEL5
ENSCJAT00000062338	ENSCJAG00000003194	YPEL5
ENSCJAT00000006110	ENSCJAG00000003179	LCLAT1
ENSCJAT00000006107	ENSCJAG00000003179	LCLAT1
ENSCJAT00000006120	ENSCJAG00000003179	LCLAT1
ENSCJAT00000022183	ENSCJAG00000003137	EHD3
ENSCJAT00000006032	ENSCJAG00000003137	EHD3
ENSCJAT00000006028	ENSCJAG00000003137	EHD3

ENSCJAT00000056564	ENSCJAG00000008811	CLIP4
ENSCJAT00000061584	ENSCJAG00000008811	CLIP4
ENSCJAT00000017293	ENSCJAG00000008912	FAM179A
ENSCJAT00000017367	ENSCJAG00000008980	TRMT61B
ENSCJAT00000017443	ENSCJAG00000008988	PPP1CB
ENSCJAT00000017449	ENSCJAG00000008988	PPP1CB
ENSCJAT00000017461	ENSCJAG00000009019	FOSL2
ENSCJAT00000017625	ENSCJAG00000009098	unknown
ENSCJAT00000017674	ENSCJAG00000009108	SLC4A1AP
ENSCJAT00000061432	ENSCJAG00000009108	SLC4A1AP
ENSCJAT00000055315	ENSCJAG00000009108	SLC4A1AP
ENSCJAT00000060348	ENSCJAG00000009180	ZNF512
ENSCJAT00000032104	ENSCJAG00000009180	ZNF512
ENSCJAT00000052262	ENSCJAG00000009180	ZNF512
ENSCJAT00000059932	ENSCJAG00000009180	ZNF512
ENSCJAT00000002001	ENSCJAG00000001098	MPV17
ENSCJAT00000002315	ENSCJAG00000033953	CGREF1
ENSCJAT00000062793	ENSCJAG00000001279	AGBL5
ENSCJAT00000002433	ENSCJAG00000001279	AGBL5
ENSCJAT00000002419	ENSCJAG00000001279	AGBL5
ENSCJAT00000058677	ENSCJAG00000001317	TMEM214
ENSCJAT00000057335	ENSCJAG00000001317	TMEM214
ENSCJAT00000002475	ENSCJAG00000001317	TMEM214
ENSCJAT00000052680	ENSCJAG00000001317	TMEM214
ENSCJAT00000002471	ENSCJAG00000001317	TMEM214
ENSCJAT00000002610	ENSCJAG00000001393	CIB4
ENSCJAT00000002658	ENSCJAG00000001409	OTOF
ENSCJAT00000002810	ENSCJAG00000001491	GPR113
ENSCJAT00000062089	ENSCJAG00000031704	unknown
ENSCJAT00000016344	ENSCJAG00000008396	ASXL2
ENSCJAT00000016375	ENSCJAG00000008396	ASXL2
ENSCJAT00000015784	ENSCJAG00000008094	DNAJC27
ENSCJAT00000015789	ENSCJAG00000008094	DNAJC27
ENSCJAT00000014699	ENSCJAG00000007450	PUM2
ENSCJAT00000052437	ENSCJAG00000007450	PUM2
ENSCJAT00000011957	ENSCJAG00000007450	PUM2
ENSCJAT00000060084	ENSCJAG00000007450	PUM2
ENSCJAT00000014705	ENSCJAG00000007450	PUM2
ENSCJAT00000014557	ENSCJAG00000007433	LAPTM4A
ENSCJAT00000014535	ENSCJAG00000007406	MATN3
ENSCJAT00000014552	ENSCJAG00000007406	MATN3
ENSCJAT00000014550	ENSCJAG00000007406	MATN3
ENSCJAT00000014451	ENSCJAG00000007361	unknown
ENSCJAT00000014453	ENSCJAG00000007361	unknown
ENSCJAT00000014248	ENSCJAG00000007262	RAD51AP2
ENSCJAT00000014235	ENSCJAG00000007231	FAM49A
ENSCJAT00000013710	ENSCJAG00000007012	TRIB2
ENSCJAT00000013520	ENSCJAG00000006910	E2F6
ENSCJAT00000013539	ENSCJAG00000006910	E2F6
ENSCJAT00000013505	ENSCJAG00000006910	E2F6
ENSCJAT00000013515	ENSCJAG00000006910	E2F6
ENSCJAT00000013504	ENSCJAG00000006910	E2F6
ENSCJAT00000012828	ENSCJAG00000006576	ODC1
ENSCJAT00000012455	ENSCJAG00000006394	YWHAQ
ENSCJAT00000012460	ENSCJAG00000006394	YWHAQ
ENSCJAT00000055540	ENSCJAG00000006369	ADAM17
ENSCJAT00000012372	ENSCJAG00000006360	IAH1
ENSCJAT00000011884	ENSCJAG00000006054	KIDINS220
ENSCJAT00000012017	ENSCJAG00000006054	KIDINS220

ENSCJAT00000011994	ENSCJAG00000006054	KIDINS220
ENSCJAT00000011997	ENSCJAG00000006054	KIDINS220
ENSCJAT00000011985	ENSCJAG00000006054	KIDINS220
ENSCJAT00000031971	ENSCJAG00000016446	DCDC2C
ENSCJAT00000032035	ENSCJAG00000016462	unknown
ENSCJAT00000032044	ENSCJAG00000016462	unknown
ENSCJAT00000025035	ENSCJAG00000012901	CCDC39
ENSCJAT00000025038	ENSCJAG00000012901	CCDC39
ENSCJAT00000062018	ENSCJAG00000012901	CCDC39
ENSCJAT00000053616	ENSCJAG00000013172	ABCC5
ENSCJAT00000052409	ENSCJAG00000013172	ABCC5
ENSCJAT00000025435	ENSCJAG00000013060	DVL3
ENSCJAT00000025206	ENSCJAG00000012959	ABCF3
ENSCJAT00000029663	ENSCJAG00000015206	VWA5B2
ENSCJAT00000029644	ENSCJAG00000015206	VWA5B2
ENSCJAT00000060785	ENSCJAG00000015132	ECE2
ENSCJAT00000062229	ENSCJAG00000015132	ECE2
ENSCJAT00000029541	ENSCJAG00000015132	ECE2
ENSCJAT00000063166	ENSCJAG00000015132	ECE2
ENSCJAT00000029536	ENSCJAG00000015132	ECE2
ENSCJAT00000029065	ENSCJAG00000014925	CHRD
ENSCJAT00000061302	ENSCJAG00000014925	CHRD
ENSCJAT00000029632	ENSCJAG00000014775	SENP2
ENSCJAT00000060075	ENSCJAG00000014775	SENP2
ENSCJAT00000028802	ENSCJAG00000014775	SENP2
ENSCJAT00000028157	ENSCJAG00000014480	RFC4
ENSCJAT00000027896	ENSCJAG00000014348	TPRG1
ENSCJAT00000027863	ENSCJAG00000014256	TP63
ENSCJAT00000027860	ENSCJAG00000014256	TP63
ENSCJAT00000027535	ENSCJAG00000014165	FGF12
ENSCJAT00000027537	ENSCJAG00000014165	FGF12
ENSCJAT00000027508	ENSCJAG00000014159	unknown
ENSCJAT00000027501	ENSCJAG00000014131	ATP13A5
ENSCJAT00000027292	ENSCJAG00000014044	HES1
ENSCJAT00000056763	ENSCJAG00000013687	PAK2
ENSCJAT00000026628	ENSCJAG00000013687	PAK2
ENSCJAT00000003168	ENSCJAG00000001677	NRROS
ENSCJAT00000056733	ENSCJAG00000001677	NRROS
ENSCJAT00000053529	ENSCJAG00000001693	RNF168
ENSCJAT00000058274	ENSCJAG00000031316	unknown
ENSCJAT00000003238	ENSCJAG00000001716	FAM198A
ENSCJAT00000003272	ENSCJAG00000001727	SNRK
ENSCJAT00000003280	ENSCJAG00000001727	SNRK
ENSCJAT00000054449	ENSCJAG00000001727	SNRK
ENSCJAT00000003279	ENSCJAG00000001727	SNRK
ENSCJAT00000057387	ENSCJAG00000001727	SNRK
ENSCJAT00000062212	ENSCJAG00000001744	ANO10
ENSCJAT00000055986	ENSCJAG00000001744	ANO10
ENSCJAT00000053617	ENSCJAG00000001744	ANO10
ENSCJAT00000063804	ENSCJAG00000001744	ANO10
ENSCJAT00000003339	ENSCJAG00000001760	TOPAZ1
ENSCJAT00000003342	ENSCJAG00000001760	TOPAZ1
ENSCJAT00000003356	ENSCJAG00000001771	TCAIM
ENSCJAT00000057639	ENSCJAG00000001771	TCAIM
ENSCJAT00000055722	ENSCJAG00000001771	TCAIM
ENSCJAT00000003417	ENSCJAG00000001803	ZKSCAN7
ENSCJAT00000003425	ENSCJAG00000001803	ZKSCAN7
ENSCJAT00000003681	ENSCJAG00000001928	EXOSC7
ENSCJAT00000060455	ENSCJAG00000001977	SACM1L

ENSCJAT00000053333	ENSCJAG00000002217	TMIE
ENSCJAT00000004202	ENSCJAG00000002217	TMIE
ENSCJAT00000061085	ENSCJAG00000002303	SETD2
ENSCJAT00000004443	ENSCJAG00000002303	SETD2
ENSCJAT00000004430	ENSCJAG00000002303	SETD2
ENSCJAT00000061576	ENSCJAG00000002303	SETD2
ENSCJAT00000032295	ENSCJAG00000002342	KIF9
ENSCJAT00000056845	ENSCJAG00000002342	KIF9
ENSCJAT00000004799	ENSCJAG00000002499	CSPG5
ENSCJAT00000004805	ENSCJAG00000002499	CSPG5
ENSCJAT00000005199	ENSCJAG00000002710	CAMP
ENSCJAT00000056057	ENSCJAG00000002710	CAMP
ENSCJAT00000005195	ENSCJAG00000002710	CAMP
ENSCJAT00000057952	ENSCJAG00000002710	CAMP
ENSCJAT00000005481	ENSCJAG00000002762	PLXNB1
ENSCJAT00000056069	ENSCJAG00000002762	PLXNB1
ENSCJAT00000005467	ENSCJAG00000002762	PLXNB1
ENSCJAT00000057316	ENSCJAG00000002874	ATRIP
ENSCJAT00000005553	ENSCJAG00000002874	ATRIP
ENSCJAT00000005557	ENSCJAG00000002874	ATRIP
ENSCJAT00000005544	ENSCJAG00000002874	ATRIP
ENSCJAT00000006136	ENSCJAG00000003177	CELSR3
ENSCJAT00000006826	ENSCJAG00000003457	QARS
ENSCJAT00000006819	ENSCJAG00000003457	QARS
ENSCJAT00000047924	ENSCJAG00000035420	unknown
ENSCJAT00000005673	ENSCJAG00000031826	unknown
ENSCJAT00000008530	ENSCJAG00000004415	TRAIP
ENSCJAT00000008815	ENSCJAG00000004579	MON1A
ENSCJAT00000008840	ENSCJAG00000004579	MON1A
ENSCJAT00000011080	ENSCJAG00000032309	IQCF1
ENSCJAT00000011797	ENSCJAG00000006069	unknown
ENSCJAT00000011793	ENSCJAG00000006069	unknown
ENSCJAT00000011924	ENSCJAG00000006114	WDR82
ENSCJAT00000012101	ENSCJAG00000006182	DNAH1
ENSCJAT00000059403	ENSCJAG00000006182	DNAH1
ENSCJAT00000012595	ENSCJAG00000006457	NISCH
ENSCJAT00000012597	ENSCJAG00000006457	NISCH
ENSCJAT00000012608	ENSCJAG00000006457	NISCH
ENSCJAT00000060097	ENSCJAG00000007045	ITIH4
ENSCJAT00000059440	ENSCJAG00000007045	ITIH4
ENSCJAT00000013837	ENSCJAG00000007045	ITIH4
ENSCJAT00000013822	ENSCJAG00000007045	ITIH4
ENSCJAT00000013816	ENSCJAG00000007045	ITIH4
ENSCJAT00000013813	ENSCJAG00000007045	ITIH4
ENSCJAT00000014084	ENSCJAG00000007179	unknown
ENSCJAT00000014381	ENSCJAG00000007253	TKT
ENSCJAT00000014360	ENSCJAG00000007253	TKT
ENSCJAT00000060834	ENSCJAG00000007253	TKT
ENSCJAT00000014461	ENSCJAG00000007337	DCP1A
ENSCJAT00000014473	ENSCJAG00000007337	DCP1A
ENSCJAT00000001315	ENSCJAG00000007337	DCP1A
ENSCJAT00000062400	ENSCJAG00000007392	CACNA1D
ENSCJAT00000021247	ENSCJAG00000010847	FAM208A
ENSCJAT00000039142	ENSCJAG00000019922	ARF4
ENSCJAT00000039114	ENSCJAG00000019910	DENND6A
ENSCJAT00000039047	ENSCJAG00000019871	SLMAP
ENSCJAT00000039049	ENSCJAG00000019871	SLMAP
ENSCJAT00000038772	ENSCJAG00000019749	RPP14
ENSCJAT00000038591	ENSCJAG00000019678	C3orf14

ENSCJAT00000038459	ENSCJAG00000019617	unknown
ENSCJAT00000053647	ENSCJAG00000019487	ADAMTS9
ENSCJAT00000038172	ENSCJAG00000019439	MAGI1
ENSCJAT00000065408	ENSCJAG00000037769	unknown
ENSCJAT00000009455	ENSCJAG00000008143	unknown
ENSCJAT00000024187	ENSCJAG00000013564	EOGT
ENSCJAT00000026409	ENSCJAG00000013564	EOGT
ENSCJAT00000026415	ENSCJAG00000013564	EOGT
ENSCJAT00000058435	ENSCJAG00000013564	EOGT
ENSCJAT00000025988	ENSCJAG00000013370	ARL6IP5
ENSCJAT00000026001	ENSCJAG00000013370	ARL6IP5
ENSCJAT00000025962	ENSCJAG00000013357	LMOD3
ENSCJAT00000059795	ENSCJAG00000013120	EIF4E3
ENSCJAT00000029829	ENSCJAG00000015257	CHL1
ENSCJAT00000030158	ENSCJAG00000015500	LRRN1
ENSCJAT00000030328	ENSCJAG00000015544	ITPR1
ENSCJAT00000030977	ENSCJAG00000015919	RAD18
ENSCJAT00000031500	ENSCJAG00000016178	unknown
ENSCJAT00000031480	ENSCJAG00000016178	unknown
ENSCJAT00000030526	ENSCJAG00000016205	IL17RE
ENSCJAT00000031554	ENSCJAG00000016205	IL17RE
ENSCJAT00000061719	ENSCJAG00000016205	IL17RE
ENSCJAT00000056503	ENSCJAG00000016416	unknown
ENSCJAT00000055034	ENSCJAG00000016416	unknown
ENSCJAT00000031927	ENSCJAG00000016416	unknown
ENSCJAT00000031986	ENSCJAG00000016439	SEC13
ENSCJAT00000031993	ENSCJAG00000016439	SEC13
ENSCJAT00000052248	ENSCJAG00000016632	VGLL4
ENSCJAT00000053635	ENSCJAG00000016632	VGLL4
ENSCJAT00000032352	ENSCJAG00000016632	VGLL4
ENSCJAT00000032364	ENSCJAG00000016643	TAMM41
ENSCJAT00000032525	ENSCJAG00000016687	PPARG
ENSCJAT00000061827	ENSCJAG00000016687	PPARG
ENSCJAT00000053214	ENSCJAG00000016687	PPARG
ENSCJAT00000057491	ENSCJAG00000016687	PPARG
ENSCJAT00000032510	ENSCJAG00000016687	PPARG
ENSCJAT00000032533	ENSCJAG00000016733	MKRN2
ENSCJAT00000053112	ENSCJAG00000016844	HDAC11
ENSCJAT00000060422	ENSCJAG00000016844	HDAC11
ENSCJAT00000032785	ENSCJAG00000016844	HDAC11
ENSCJAT00000060685	ENSCJAG00000016952	XPC
ENSCJAT00000032991	ENSCJAG00000016952	XPC
ENSCJAT00000053019	ENSCJAG00000017014	GRIP2
ENSCJAT00000028188	ENSCJAG00000017014	GRIP2
ENSCJAT00000060321	ENSCJAG00000034449	unknown
ENSCJAT00000033855	ENSCJAG00000017390	H1FX
ENSCJAT00000033849	ENSCJAG00000017390	H1FX
ENSCJAT00000057674	ENSCJAG00000017469	EFCC1
ENSCJAT00000034004	ENSCJAG00000017469	EFCC1
ENSCJAT00000034050	ENSCJAG00000017490	ACAD9
ENSCJAT00000010125	ENSCJAG00000005226	PODXL2
ENSCJAT00000010112	ENSCJAG00000005226	PODXL2
ENSCJAT00000010132	ENSCJAG00000005226	PODXL2
ENSCJAT00000013182	ENSCJAG00000006741	ZNF148
ENSCJAT00000012992	ENSCJAG00000006666	HEG1
ENSCJAT00000015630	ENSCJAG00000008000	PARP9
ENSCJAT00000055195	ENSCJAG00000008000	PARP9
ENSCJAT00000056757	ENSCJAG00000008000	PARP9
ENSCJAT00000015647	ENSCJAG00000008000	PARP9

ENSCJAT00000054444	ENSCJAG00000008000	PARP9
ENSCJAT00000016028	ENSCJAG00000008181	CD86
ENSCJAT00000016014	ENSCJAG00000008181	CD86
ENSCJAT00000016249	ENSCJAG00000008258	SLC15A2
ENSCJAT00000016240	ENSCJAG00000008258	SLC15A2
ENSCJAT00000063764	ENSCJAG00000008258	SLC15A2
ENSCJAT00000016168	ENSCJAG00000008258	SLC15A2
ENSCJAT00000016761	ENSCJAG00000008611	RABL3
ENSCJAT00000016882	ENSCJAG00000008626	HGD
ENSCJAT00000060052	ENSCJAG00000008891	MAATS1
ENSCJAT00000017408	ENSCJAG00000008990	TIMMDC1
ENSCJAT00000017494	ENSCJAG00000008999	POGLUT1
ENSCJAT00000017498	ENSCJAG00000008999	POGLUT1
ENSCJAT00000017464	ENSCJAG00000008999	POGLUT1
ENSCJAT00000018236	ENSCJAG00000009359	GRAMD1C
ENSCJAT00000018254	ENSCJAG00000009359	GRAMD1C
ENSCJAT00000039991	ENSCJAG00000020358	ATG3
ENSCJAT00000039992	ENSCJAG00000020358	ATG3
ENSCJAT00000039959	ENSCJAG00000020341	CCDC80
ENSCJAT00000039952	ENSCJAG00000020341	CCDC80
ENSCJAT00000039962	ENSCJAG00000020341	CCDC80
ENSCJAT00000039915	ENSCJAG00000020325	GTPBP8
ENSCJAT00000062145	ENSCJAG00000020325	GTPBP8
ENSCJAT00000039925	ENSCJAG00000020325	GTPBP8
ENSCJAT00000039750	ENSCJAG00000020242	TMPRSS7
ENSCJAT00000039747	ENSCJAG00000020242	TMPRSS7
ENSCJAT00000055204	ENSCJAG00000020142	TRAT1
ENSCJAT00000039561	ENSCJAG00000020142	TRAT1
ENSCJAT00000000888	ENSCJAG00000000487	NXPE3
ENSCJAT00000000891	ENSCJAG00000000492	unknown
ENSCJAT00000000904	ENSCJAG00000000493	CEP97
ENSCJAT00000000898	ENSCJAG00000000493	CEP97
ENSCJAT00000001021	ENSCJAG00000000553	TFG
ENSCJAT00000001050	ENSCJAG00000000558	GPR128
ENSCJAT00000061487	ENSCJAG00000000558	GPR128
ENSCJAT00000001089	ENSCJAG00000000590	NIT2
ENSCJAT00000001649	ENSCJAG00000000590	NIT2
ENSCJAT00000001111	ENSCJAG00000000607	TMEM30C
ENSCJAT00000028056	ENSCJAG00000014407	TCEA1
ENSCJAT00000028107	ENSCJAG00000014407	TCEA1
ENSCJAT00000061802	ENSCJAG00000014407	TCEA1
ENSCJAT00000028081	ENSCJAG00000014407	TCEA1
ENSCJAT00000032411	ENSCJAG00000016657	TGS1
ENSCJAT00000032409	ENSCJAG00000016657	TGS1
ENSCJAT00000032312	ENSCJAG00000016614	CHCHD7
ENSCJAT00000016792	ENSCJAG00000008610	unknown
ENSCJAT00000016810	ENSCJAG00000008610	unknown
ENSCJAT00000016835	ENSCJAG00000008657	FAM110B
ENSCJAT00000017400	ENSCJAG00000008976	CA8
ENSCJAT00000017409	ENSCJAG00000008976	CA8
ENSCJAT00000017618	ENSCJAG00000009025	CHD7
ENSCJAT00000017735	ENSCJAG00000009122	ASPH
ENSCJAT00000017740	ENSCJAG00000009122	ASPH
ENSCJAT00000063064	ENSCJAG00000009122	ASPH
ENSCJAT00000017724	ENSCJAG00000009122	ASPH
ENSCJAT00000017739	ENSCJAG00000009122	ASPH
ENSCJAT00000017737	ENSCJAG00000009122	ASPH
ENSCJAT00000017863	ENSCJAG00000009217	YTHDF3
ENSCJAT00000017866	ENSCJAG00000009217	YTHDF3

ENSCJAT00000055016	ENSCJAG00000009217	YTHDF3
ENSCJAT00000053161	ENSCJAG00000009217	YTHDF3
ENSCJAT00000059301	ENSCJAG00000009217	YTHDF3
ENSCJAT00000017841	ENSCJAG00000009217	YTHDF3
ENSCJAT00000017844	ENSCJAG00000009217	YTHDF3
ENSCJAT00000017896	ENSCJAG00000009244	CYP7B1
ENSCJAT00000018150	ENSCJAG00000009369	DNAJC5B
ENSCJAT00000018143	ENSCJAG00000009369	DNAJC5B
ENSCJAT00000018190	ENSCJAG00000009384	TRIM55
ENSCJAT00000018199	ENSCJAG00000009384	TRIM55
ENSCJAT00000018209	ENSCJAG00000009384	TRIM55
ENSCJAT00000018198	ENSCJAG00000009384	TRIM55
ENSCJAT00000055627	ENSCJAG00000004434	ADHFE1
ENSCJAT00000062161	ENSCJAG00000004434	ADHFE1
ENSCJAT00000008503	ENSCJAG00000004434	ADHFE1
ENSCJAT00000060376	ENSCJAG00000004434	ADHFE1
ENSCJAT00000058212	ENSCJAG00000004515	VCPIP1
ENSCJAT00000008651	ENSCJAG00000004515	VCPIP1
ENSCJAT00000008661	ENSCJAG00000004515	VCPIP1
ENSCJAT00000052578	ENSCJAG00000004515	VCPIP1
ENSCJAT00000060166	ENSCJAG00000004573	MCMDC2
ENSCJAT00000061318	ENSCJAG00000004573	MCMDC2
ENSCJAT00000008834	ENSCJAG00000004573	MCMDC2
ENSCJAT00000058487	ENSCJAG00000004573	MCMDC2
ENSCJAT00000056615	ENSCJAG00000004573	MCMDC2
ENSCJAT00000062321	ENSCJAG00000004573	MCMDC2
ENSCJAT00000059045	ENSCJAG00000004964	C8orf34
ENSCJAT00000009613	ENSCJAG00000004964	C8orf34
ENSCJAT00000015056	ENSCJAG00000007714	TERF1
ENSCJAT00000015060	ENSCJAG00000007714	TERF1
ENSCJAT00000055318	ENSCJAG00000007632	unknown
ENSCJAT00000014900	ENSCJAG00000007605	unknown
ENSCJAT00000001057	ENSCJAG00000000577	LY96
ENSCJAT00000001019	ENSCJAG00000000555	PI15
ENSCJAT00000001022	ENSCJAG00000000555	PI15
ENSCJAT00000061303	ENSCJAG00000000555	PI15
ENSCJAT00000001024	ENSCJAG00000000555	PI15
ENSCJAT00000001015	ENSCJAG00000000549	CRISPLD1
ENSCJAT00000001014	ENSCJAG00000000549	CRISPLD1
ENSCJAT00000001016	ENSCJAG00000000549	CRISPLD1
ENSCJAT00000000918	ENSCJAG00000000495	ZC2HC1A
ENSCJAT00000061438	ENSCJAG00000000485	STMN2
ENSCJAT00000000893	ENSCJAG00000000485	STMN2
ENSCJAT00000000831	ENSCJAG00000000454	ZBTB10
ENSCJAT00000000836	ENSCJAG00000000454	ZBTB10
ENSCJAT00000000826	ENSCJAG00000000454	ZBTB10
ENSCJAT00000000833	ENSCJAG00000000454	ZBTB10
ENSCJAT00000000787	ENSCJAG00000000433	FABP12
ENSCJAT00000000760	ENSCJAG00000000415	CHMP4C
ENSCJAT00000000714	ENSCJAG00000000393	LRRCC1
ENSCJAT00000000722	ENSCJAG00000000393	LRRCC1
ENSCJAT00000000725	ENSCJAG00000000393	LRRCC1
ENSCJAT00000000665	ENSCJAG00000000369	CA3
ENSCJAT00000000402	ENSCJAG00000000226	RIPK2
ENSCJAT00000000417	ENSCJAG00000000226	RIPK2
ENSCJAT00000000400	ENSCJAG00000000220	OSGIN2
ENSCJAT00000000317	ENSCJAG00000037181	LRRC69
ENSCJAT00000000313	ENSCJAG00000037181	LRRC69
ENSCJAT00000000252	ENSCJAG00000000138	TRIQK

ENSCJAT00000000225	ENSCJAG00000000126	FAM92A1
ENSCJAT00000000222	ENSCJAG00000000126	FAM92A1
ENSCJAT00000000073	ENSCJAG00000000034	DPY19L4
ENSCJAT00000000066	ENSCJAG00000000034	DPY19L4
ENSCJAT00000000049	ENSCJAG00000000015	INTS8
ENSCJAT00000000046	ENSCJAG00000000015	INTS8
ENSCJAT00000042054	ENSCJAG00000036389	ZNF34
ENSCJAT00000042073	ENSCJAG00000021397	LRRC24
ENSCJAT00000042085	ENSCJAG00000021404	GPT
ENSCJAT00000042084	ENSCJAG00000021404	GPT
ENSCJAT00000042083	ENSCJAG00000021404	GPT
ENSCJAT00000062912	ENSCJAG00000021437	BOP1
ENSCJAT00000042103	ENSCJAG00000021413	PYCRL
ENSCJAT00000042113	ENSCJAG00000021419	EEF1D
ENSCJAT00000004733	ENSCJAG00000021419	EEF1D
ENSCJAT00000042247	ENSCJAG00000021475	SLURP1
ENSCJAT00000042252	ENSCJAG00000021484	LY6K
ENSCJAT00000054712	ENSCJAG00000010346	unknown
ENSCJAT00000010126	ENSCJAG00000005232	ST3GAL1
ENSCJAT00000008615	ENSCJAG00000004490	LRRC6
ENSCJAT00000008611	ENSCJAG00000004490	LRRC6
ENSCJAT00000008532	ENSCJAG00000004367	EFR3A
ENSCJAT00000008504	ENSCJAG00000004367	EFR3A
ENSCJAT00000024420	ENSCJAG00000012609	GSDMC
ENSCJAT00000024421	ENSCJAG00000012609	GSDMC
ENSCJAT00000001523	ENSCJAG00000000837	KLHL38
ENSCJAT00000001665	ENSCJAG00000000868	ATAD2
ENSCJAT00000001684	ENSCJAG00000000868	ATAD2
ENSCJAT00000001680	ENSCJAG00000000868	ATAD2
ENSCJAT00000001679	ENSCJAG00000000868	ATAD2
ENSCJAT00000001790	ENSCJAG00000000953	TBC1D31
ENSCJAT00000001794	ENSCJAG00000000953	TBC1D31
ENSCJAT00000001800	ENSCJAG00000000953	TBC1D31
ENSCJAT00000011270	ENSCJAG00000005804	TNFRSF11B
ENSCJAT00000011265	ENSCJAG00000005804	TNFRSF11B
ENSCJAT00000052681	ENSCJAG00000006204	SYBU
ENSCJAT00000052908	ENSCJAG00000006204	SYBU
ENSCJAT00000012420	ENSCJAG00000006282	PKHD1L1
ENSCJAT00000057300	ENSCJAG00000006282	PKHD1L1
ENSCJAT00000012398	ENSCJAG00000006282	PKHD1L1
ENSCJAT00000012432	ENSCJAG00000006389	ENY2
ENSCJAT00000055051	ENSCJAG00000006407	NUDCD1
ENSCJAT00000012495	ENSCJAG00000006407	NUDCD1
ENSCJAT00000012490	ENSCJAG00000006407	NUDCD1
ENSCJAT00000012535	ENSCJAG00000006437	EMC2
ENSCJAT00000013092	ENSCJAG00000006727	unknown
ENSCJAT00000031533	ENSCJAG00000016181	DCAF13
ENSCJAT00000031308	ENSCJAG00000016087	AZIN1
ENSCJAT00000057716	ENSCJAG00000016087	AZIN1
ENSCJAT00000052293	ENSCJAG00000015844	YWHAZ
ENSCJAT00000061048	ENSCJAG00000015844	YWHAZ
ENSCJAT00000030875	ENSCJAG00000015844	YWHAZ
ENSCJAT00000054826	ENSCJAG00000015844	YWHAZ
ENSCJAT00000053058	ENSCJAG00000015844	YWHAZ
ENSCJAT00000056431	ENSCJAG00000015844	YWHAZ
ENSCJAT00000030567	ENSCJAG00000015719	COX6C
ENSCJAT00000059541	ENSCJAG00000015567	POP1
ENSCJAT00000030321	ENSCJAG00000015567	POP1
ENSCJAT00000030278	ENSCJAG00000015560	unknown

ENSCJAT00000053972	ENSCJAG00000032339	unknown
ENSCJAT00000062225	ENSCJAG00000002129	ECT2
ENSCJAT00000062190	ENSCJAG00000002194	NCEH1
ENSCJAT00000004166	ENSCJAG00000002194	NCEH1
ENSCJAT00000004160	ENSCJAG00000002194	NCEH1
ENSCJAT00000004631	ENSCJAG00000002414	SLC2A2
ENSCJAT00000004634	ENSCJAG00000002414	SLC2A2
ENSCJAT00000004889	ENSCJAG00000002517	PHC3
ENSCJAT00000004895	ENSCJAG00000002517	PHC3
ENSCJAT00000004930	ENSCJAG00000002573	GPR160
ENSCJAT00000061201	ENSCJAG00000002651	MECOM
ENSCJAT00000005185	ENSCJAG00000002651	MECOM
ENSCJAT00000005176	ENSCJAG00000002651	MECOM
ENSCJAT00000005163	ENSCJAG00000002651	MECOM
ENSCJAT00000005245	ENSCJAG00000002711	GOLIM4
ENSCJAT00000005238	ENSCJAG00000002711	GOLIM4
ENSCJAT00000005507	ENSCJAG00000002837	SI
ENSCJAT00000054365	ENSCJAG00000002934	B3GALNT1
ENSCJAT00000057103	ENSCJAG00000002934	B3GALNT1
ENSCJAT00000052793	ENSCJAG00000002934	B3GALNT1
ENSCJAT00000005622	ENSCJAG00000002946	unknown
ENSCJAT00000052363	ENSCJAG00000002974	SMC4
ENSCJAT00000000737	ENSCJAG00000000395	MFSD1
ENSCJAT00000000742	ENSCJAG00000000395	MFSD1
ENSCJAT00000000744	ENSCJAG00000000395	MFSD1
ENSCJAT00000000719	ENSCJAG00000000395	MFSD1
ENSCJAT00000009204	ENSCJAG00000004776	C3orf55
ENSCJAT00000009196	ENSCJAG00000004776	C3orf55
ENSCJAT00000009017	ENSCJAG00000004692	CCNL1
ENSCJAT00000009099	ENSCJAG00000004692	CCNL1
ENSCJAT00000009034	ENSCJAG00000004692	CCNL1
ENSCJAT00000001634	ENSCJAG00000000902	SLC33A1
ENSCJAT00000052623	ENSCJAG00000000902	SLC33A1
ENSCJAT00000001785	ENSCJAG00000000982	GPR149
ENSCJAT00000001855	ENSCJAG00000000986	DHX36
ENSCJAT00000001872	ENSCJAG00000000986	DHX36
ENSCJAT00000001867	ENSCJAG00000000986	DHX36
ENSCJAT00000057914	ENSCJAG00000001054	MBNL1
ENSCJAT00000001954	ENSCJAG00000001054	MBNL1
ENSCJAT00000001953	ENSCJAG00000001054	MBNL1
ENSCJAT00000001938	ENSCJAG00000001054	MBNL1
ENSCJAT00000001939	ENSCJAG00000001054	MBNL1
ENSCJAT00000001931	ENSCJAG00000001054	MBNL1
ENSCJAT00000001949	ENSCJAG00000001054	MBNL1
ENSCJAT00000001992	ENSCJAG00000001095	AADACL2
ENSCJAT00000002058	ENSCJAG00000001129	P2RY12
ENSCJAT00000002084	ENSCJAG00000001146	CLRN1
ENSCJAT00000002094	ENSCJAG00000001151	unknown
ENSCJAT00000002175	ENSCJAG00000001182	ERICH6
ENSCJAT00000002173	ENSCJAG00000001182	ERICH6
ENSCJAT00000002492	ENSCJAG00000001343	TM4SF4
ENSCJAT00000002488	ENSCJAG00000001343	TM4SF4
ENSCJAT00000002837	ENSCJAG00000001501	CPA3
ENSCJAT00000002869	ENSCJAG00000001529	ZIC4
ENSCJAT00000002888	ENSCJAG00000001534	PLSCR5
ENSCJAT00000002935	ENSCJAG00000001542	PLSCR1
ENSCJAT00000055257	ENSCJAG00000033152	unknown
ENSCJAT00000003377	ENSCJAG00000001762	PLS1
ENSCJAT00000003371	ENSCJAG00000001762	PLS1

ENSCJAT00000003521	ENSCJAG00000001797	ATR
ENSCJAT00000003699	ENSCJAG00000001924	GK5
ENSCJAT00000003673	ENSCJAG00000001924	GK5
ENSCJAT00000052788	ENSCJAG00000031471	ZBTB38
ENSCJAT00000003953	ENSCJAG00000002072	PXYLP1
ENSCJAT00000004136	ENSCJAG00000002172	RBP1
ENSCJAT00000004377	ENSCJAG00000002299	FAIM
ENSCJAT00000004375	ENSCJAG00000002299	FAIM
ENSCJAT00000004387	ENSCJAG00000002299	FAIM
ENSCJAT00000061018	ENSCJAG00000002299	FAIM
ENSCJAT00000057046	ENSCJAG00000002315	CEP70
ENSCJAT00000057998	ENSCJAG00000002315	CEP70
ENSCJAT00000004455	ENSCJAG00000002315	CEP70
ENSCJAT00000053957	ENSCJAG00000002315	CEP70
ENSCJAT00000004452	ENSCJAG00000002315	CEP70
ENSCJAT00000004694	ENSCJAG00000002446	DZIP1L
ENSCJAT00000004732	ENSCJAG00000002455	IL20RB
ENSCJAT00000005335	ENSCJAG00000002778	AMOTL2
ENSCJAT00000005339	ENSCJAG00000002778	AMOTL2
ENSCJAT00000005451	ENSCJAG00000002845	SRPRB
ENSCJAT00000005586	ENSCJAG00000002848	TF
ENSCJAT00000027903	ENSCJAG00000002848	TF
ENSCJAT00000005791	ENSCJAG00000003027	ACAD11
ENSCJAT00000037487	ENSCJAG00000003027	ACAD11
ENSCJAT00000005789	ENSCJAG00000003027	ACAD11
ENSCJAT00000053346	ENSCJAG00000003031	ACKR4
ENSCJAT00000006006	ENSCJAG00000003118	CPNE4
ENSCJAT00000006004	ENSCJAG00000003118	CPNE4
ENSCJAT00000006052	ENSCJAG00000003143	MRPL3
ENSCJAT00000006056	ENSCJAG00000003143	MRPL3
ENSCJAT00000006066	ENSCJAG00000003162	NUDT16
ENSCJAT00000006365	ENSCJAG00000003293	PIK3R4
ENSCJAT00000006547	ENSCJAG00000003415	METTL6
ENSCJAT00000006544	ENSCJAG00000003415	METTL6
ENSCJAT00000056275	ENSCJAG00000003426	HACL1
ENSCJAT00000006596	ENSCJAG00000003426	HACL1
ENSCJAT00000057109	ENSCJAG00000003426	HACL1
ENSCJAT00000059543	ENSCJAG00000003426	HACL1
ENSCJAT00000052836	ENSCJAG00000003426	HACL1
ENSCJAT00000059163	ENSCJAG00000003426	HACL1
ENSCJAT00000054385	ENSCJAG00000003498	BTD
ENSCJAT00000055505	ENSCJAG00000003498	BTD
ENSCJAT00000007051	ENSCJAG00000003681	KCNH8
ENSCJAT00000007117	ENSCJAG00000003700	EFHB
ENSCJAT00000055351	ENSCJAG00000003700	EFHB
ENSCJAT00000052958	ENSCJAG00000003744	KAT2B
ENSCJAT00000007198	ENSCJAG00000003760	SGOL1
ENSCJAT00000007200	ENSCJAG00000003760	SGOL1
ENSCJAT00000007204	ENSCJAG00000003760	SGOL1
ENSCJAT00000007501	ENSCJAG00000003887	THRB
ENSCJAT00000007733	ENSCJAG00000003963	TOP2B
ENSCJAT00000008174	ENSCJAG00000004272	CMC1
ENSCJAT00000008400	ENSCJAG00000004363	TGFBR2
ENSCJAT00000008410	ENSCJAG00000004363	TGFBR2
ENSCJAT00000008379	ENSCJAG00000004363	TGFBR2
ENSCJAT00000008466	ENSCJAG00000004395	GADL1
ENSCJAT00000008494	ENSCJAG00000004428	STT3B
ENSCJAT00000022960	ENSCJAG00000011783	DYNC1LI1
ENSCJAT00000057813	ENSCJAG00000011783	DYNC1LI1

ENSCJAT00000058460	ENSCJAG00000011986	SUSD5
ENSCJAT00000023259	ENSCJAG00000011986	SUSD5
ENSCJAT00000023799	ENSCJAG00000012204	PDCD6IP
ENSCJAT00000055693	ENSCJAG00000012204	PDCD6IP
ENSCJAT00000023816	ENSCJAG00000012204	PDCD6IP
ENSCJAT00000061046	ENSCJAG00000012602	GOLGA4
ENSCJAT00000019882	ENSCJAG00000010212	unknown
ENSCJAT00000019876	ENSCJAG00000010212	unknown
ENSCJAT00000019806	ENSCJAG00000010173	OXSR1
ENSCJAT00000019770	ENSCJAG00000010167	SLC22A13
ENSCJAT00000019095	ENSCJAG00000009759	WDR48
ENSCJAT00000019098	ENSCJAG00000009759	WDR48
ENSCJAT00000019066	ENSCJAG00000009759	WDR48
ENSCJAT00000058989	ENSCJAG00000009759	WDR48
ENSCJAT00000052242	ENSCJAG00000009759	WDR48
ENSCJAT00000061807	ENSCJAG00000009752	GORASP1
ENSCJAT00000018900	ENSCJAG00000009752	GORASP1
ENSCJAT00000035235	ENSCJAG00000018028	VIPR1
ENSCJAT00000017169	ENSCJAG00000018028	VIPR1
ENSCJAT00000035221	ENSCJAG00000018028	VIPR1
ENSCJAT00000035259	ENSCJAG00000018053	SEC22C
ENSCJAT00000035258	ENSCJAG00000018053	SEC22C
ENSCJAT00000035433	ENSCJAG00000018130	UBXN7
ENSCJAT00000035425	ENSCJAG00000018130	UBXN7
ENSCJAT00000041862	ENSCJAG00000021290	GBAS
ENSCJAT00000028519	ENSCJAG00000021279	CCT6A
ENSCJAT00000028514	ENSCJAG00000021279	CCT6A
ENSCJAT00000041854	ENSCJAG00000021279	CCT6A
ENSCJAT00000041858	ENSCJAG00000021279	CCT6A
ENSCJAT00000016616	ENSCJAG00000008517	CRYBG3
ENSCJAT00000017111	ENSCJAG00000008810	POU1F1
ENSCJAT00000017100	ENSCJAG00000008810	POU1F1
ENSCJAT00000058563	ENSCJAG00000008842	CHMP2B
ENSCJAT00000017144	ENSCJAG00000008842	CHMP2B
ENSCJAT00000006273	ENSCJAG00000003226	unknown
ENSCJAT00000006343	ENSCJAG00000003304	SAMSN1
ENSCJAT00000006339	ENSCJAG00000003304	SAMSN1
ENSCJAT00000006349	ENSCJAG00000003304	SAMSN1
ENSCJAT00000055950	ENSCJAG00000003304	SAMSN1
ENSCJAT00000054222	ENSCJAG00000003304	SAMSN1
ENSCJAT00000006474	ENSCJAG00000003375	unknown
ENSCJAT00000016020	ENSCJAG00000003434	C21orf91
ENSCJAT00000054621	ENSCJAG00000003434	C21orf91
ENSCJAT00000006583	ENSCJAG00000003434	C21orf91
ENSCJAT00000006578	ENSCJAG00000003434	C21orf91
ENSCJAT00000026819	ENSCJAG00000013778	JAM2
ENSCJAT00000026807	ENSCJAG00000013778	JAM2
ENSCJAT00000026785	ENSCJAG00000013778	JAM2
ENSCJAT00000016376	ENSCJAG00000008373	LTN1
ENSCJAT00000016786	ENSCJAG00000008503	CCT8
ENSCJAT00000016754	ENSCJAG00000008503	CCT8
ENSCJAT00000017078	ENSCJAG00000008797	unknown
ENSCJAT00000017125	ENSCJAG00000008840	unknown
ENSCJAT00000017145	ENSCJAG00000008840	unknown
ENSCJAT00000017599	ENSCJAG00000009073	HUNK
ENSCJAT00000061683	ENSCJAG00000009187	SYNJ1
ENSCJAT00000061159	ENSCJAG00000009437	C21orf62
ENSCJAT00000018264	ENSCJAG00000009437	C21orf62
ENSCJAT00000000109	ENSCJAG00000000056	IFNAR1

ENSCJAT00000000108	ENSCJAG00000000056	IFNAR1
ENSCJAT00000006033	ENSCJAG00000003146	TMEM50B
ENSCJAT00000005948	ENSCJAG00000003083	GART
ENSCJAT00000005512	ENSCJAG00000002873	ATP5O
ENSCJAT00000005373	ENSCJAG00000002805	SETD4
ENSCJAT00000005360	ENSCJAG00000038083	CBR1
ENSCJAT00000005357	ENSCJAG00000038083	CBR1
ENSCJAT00000002822	ENSCJAG00000002704	SIM2
ENSCJAT00000015793	ENSCJAG00000008102	unknown
ENSCJAT00000059453	ENSCJAG00000005714	WRB
ENSCJAT00000057259	ENSCJAG00000033400	unknown
ENSCJAT00000021910	ENSCJAG00000011236	TMPRSS2
ENSCJAT00000021894	ENSCJAG00000011236	TMPRSS2
ENSCJAT00000026858	ENSCJAG00000013815	PRDM15
ENSCJAT00000014436	ENSCJAG00000007364	TFF2
ENSCJAT00000052509	ENSCJAG00000001863	COL6A2
ENSCJAT00000003544	ENSCJAG00000001863	COL6A2
ENSCJAT00000003548	ENSCJAG00000001863	COL6A2
ENSCJAT00000063189	ENSCJAG00000001863	COL6A2
ENSCJAT00000020707	ENSCJAG00000008270	CAPN8
ENSCJAT00000016121	ENSCJAG00000008270	CAPN8
ENSCJAT00000057625	ENSCJAG00000022060	TLR5
ENSCJAT00000043045	ENSCJAG00000022060	TLR5
ENSCJAT00000061498	ENSCJAG00000006530	BROX
ENSCJAT00000057214	ENSCJAG00000006530	BROX
ENSCJAT00000012821	ENSCJAG00000006530	BROX
ENSCJAT00000011809	ENSCJAG00000006086	DUSP10
ENSCJAT00000011836	ENSCJAG00000006086	DUSP10
ENSCJAT00000011828	ENSCJAG00000006086	DUSP10
ENSCJAT00000056138	ENSCJAG00000006086	DUSP10
ENSCJAT00000011920	ENSCJAG00000006146	2-Mar
ENSCJAT00000011934	ENSCJAG00000006146	2-Mar
ENSCJAT00000025150	ENSCJAG00000012954	LYPLAL1
ENSCJAT00000029669	ENSCJAG00000015233	unknown
ENSCJAT00000029787	ENSCJAG00000015237	KCTD3
ENSCJAT00000029773	ENSCJAG00000015237	KCTD3
ENSCJAT00000030533	ENSCJAG00000015690	FLVCR1
ENSCJAT00000052321	ENSCJAG00000015748	PPP2R5A
ENSCJAT00000030920	ENSCJAG00000015898	RD3
ENSCJAT00000030954	ENSCJAG00000015904	unknown
ENSCJAT00000030951	ENSCJAG00000015904	unknown
ENSCJAT00000021617	ENSCJAG00000011040	NUP133
ENSCJAT00000021636	ENSCJAG00000011040	NUP133
ENSCJAT00000021650	ENSCJAG00000011040	NUP133
ENSCJAT00000021882	ENSCJAG00000011215	RAB4A
ENSCJAT00000063401	ENSCJAG00000012980	CDC42BPA
ENSCJAT00000024605	ENSCJAG00000012685	SDE2
ENSCJAT00000024597	ENSCJAG00000012685	SDE2
ENSCJAT00000024296	ENSCJAG00000012510	ENAH
ENSCJAT00000024344	ENSCJAG00000012510	ENAH
ENSCJAT00000008936	ENSCJAG00000004629	LBR
ENSCJAT00000055058	ENSCJAG00000004629	LBR
ENSCJAT00000008925	ENSCJAG00000004629	LBR
ENSCJAT00000009223	ENSCJAG00000004680	ASPM
ENSCJAT00000009210	ENSCJAG00000004680	ASPM
ENSCJAT00000009051	ENSCJAG00000004680	ASPM
ENSCJAT00000056870	ENSCJAG00000004916	LHX9
ENSCJAT00000009490	ENSCJAG00000004916	LHX9
ENSCJAT00000039636	ENSCJAG00000005196	CAMSAP2

ENSCJAT00000010134	ENSCJAG00000005196	CAMSAP2
ENSCJAT00000010101	ENSCJAG00000005196	CAMSAP2
ENSCJAT00000010693	ENSCJAG00000005499	TNNI1
ENSCJAT00000010660	ENSCJAG00000005499	TNNI1
ENSCJAT00000010700	ENSCJAG00000005499	TNNI1
ENSCJAT00000011055	ENSCJAG00000005698	unknown
ENSCJAT00000055902	ENSCJAG00000005745	RNPEP
ENSCJAT00000011242	ENSCJAG00000005745	RNPEP
ENSCJAT00000011232	ENSCJAG00000005745	RNPEP
ENSCJAT00000062700	ENSCJAG00000005745	RNPEP
ENSCJAT00000011448	ENSCJAG00000005882	PTPN7
ENSCJAT00000011433	ENSCJAG00000005882	PTPN7
ENSCJAT00000053511	ENSCJAG00000005882	PTPN7
ENSCJAT00000012339	ENSCJAG00000006297	unknown
ENSCJAT00000012322	ENSCJAG00000006297	unknown
ENSCJAT00000012329	ENSCJAG00000006297	unknown
ENSCJAT00000061737	ENSCJAG00000016921	PLEKHA6
ENSCJAT00000033108	ENSCJAG00000017024	LRRN2
ENSCJAT00000033255	ENSCJAG00000017046	NFASC
ENSCJAT00000033239	ENSCJAG00000017046	NFASC
ENSCJAT00000059796	ENSCJAG00000017046	NFASC
ENSCJAT00000033244	ENSCJAG00000017046	NFASC
ENSCJAT00000021322	ENSCJAG00000010943	SLC45A3
ENSCJAT00000021568	ENSCJAG00000011061	PM20D1
ENSCJAT00000021574	ENSCJAG00000011061	PM20D1
ENSCJAT00000036685	ENSCJAG00000018679	IKBKE
ENSCJAT00000036673	ENSCJAG00000018679	IKBKE
ENSCJAT00000035923	ENSCJAG00000018353	C4BPB
ENSCJAT00000035916	ENSCJAG00000018353	C4BPB
ENSCJAT00000035925	ENSCJAG00000018353	C4BPB
ENSCJAT00000035841	ENSCJAG00000018314	CD55
ENSCJAT00000062326	ENSCJAG00000018314	CD55
ENSCJAT00000060322	ENSCJAG00000018314	CD55
ENSCJAT00000035849	ENSCJAG00000018314	CD55
ENSCJAT00000007339	ENSCJAG00000003807	CD46
ENSCJAT00000007334	ENSCJAG00000003807	CD46
ENSCJAT00000007347	ENSCJAG00000003807	CD46
ENSCJAT00000029105	ENSCJAG00000014941	HSD11B1
ENSCJAT00000029320	ENSCJAG00000015049	DIEXF
ENSCJAT00000029329	ENSCJAG00000015049	DIEXF
ENSCJAT00000017217	ENSCJAG00000008884	TRIM58
ENSCJAT00000017222	ENSCJAG00000008884	TRIM58
ENSCJAT00000017156	ENSCJAG00000008869	OR1C1
ENSCJAT00000032503	ENSCJAG00000016671	ARF1
ENSCJAT00000056687	ENSCJAG00000016671	ARF1
ENSCJAT00000040871	ENSCJAG00000016671	ARF1
ENSCJAT00000033086	ENSCJAG00000016914	HNRNPU
ENSCJAT00000033094	ENSCJAG00000016914	HNRNPU
ENSCJAT00000033165	ENSCJAG00000017069	ADSS
ENSCJAT00000033178	ENSCJAG00000017074	C1orf100
ENSCJAT00000033516	ENSCJAG00000017236	MAP1LC3C
ENSCJAT00000034766	ENSCJAG00000017734	HEATR1
ENSCJAT00000062818	ENSCJAG00000018015	GNG4
ENSCJAT00000063454	ENSCJAG00000018055	ARID4B
ENSCJAT00000035536	ENSCJAG00000018183	COA6
ENSCJAT00000035857	ENSCJAG00000018333	unknown
ENSCJAT00000035854	ENSCJAG00000018333	unknown
ENSCJAT00000052568	ENSCJAG00000018333	unknown
ENSCJAT00000035869	ENSCJAG00000018340	SPRTN

ENSCJAT00000035996	ENSCJAG00000018389	C1orf131
ENSCJAT00000036014	ENSCJAG00000018389	C1orf131
ENSCJAT00000059726	ENSCJAG00000011909	COG2
ENSCJAT00000010696	ENSCJAG00000005546	EFNA2
ENSCJAT00000010704	ENSCJAG00000005546	EFNA2
ENSCJAT00000025561	ENSCJAG00000013091	AP3D1
ENSCJAT00000025639	ENSCJAG00000013201	PLEKHJ1
ENSCJAT00000025646	ENSCJAG00000013206	SF3A2
ENSCJAT00000025679	ENSCJAG00000013217	JSRP1
ENSCJAT00000025676	ENSCJAG00000013217	JSRP1
ENSCJAT00000029278	ENSCJAG00000015041	DAPK3
ENSCJAT00000029412	ENSCJAG00000015084	MAP2K2
ENSCJAT00000029473	ENSCJAG00000015120	SIRT6
ENSCJAT00000014581	ENSCJAG00000007418	UBXN6
ENSCJAT00000014584	ENSCJAG00000007418	UBXN6
ENSCJAT00000033498	ENSCJAG00000017205	SAFB2
ENSCJAT00000033477	ENSCJAG00000017205	SAFB2
ENSCJAT00000033089	ENSCJAG00000016997	unknown
ENSCJAT00000033085	ENSCJAG00000016997	unknown
ENSCJAT00000032277	ENSCJAG00000016577	SLC25A23
ENSCJAT00000032204	ENSCJAG00000016543	DENND1C
ENSCJAT00000032198	ENSCJAG00000016543	DENND1C
ENSCJAT00000032191	ENSCJAG00000016543	DENND1C
ENSCJAT00000031454	ENSCJAG00000016161	unknown
ENSCJAT00000016307	ENSCJAG00000008391	unknown
ENSCJAT00000016339	ENSCJAG00000008402	PEX11G
ENSCJAT00000061571	ENSCJAG00000008402	PEX11G
ENSCJAT00000016718	ENSCJAG00000008568	XAB2
ENSCJAT00000020938	ENSCJAG00000010732	KANK3
ENSCJAT00000020887	ENSCJAG00000010732	KANK3
ENSCJAT00000020963	ENSCJAG00000010732	KANK3
ENSCJAT00000020973	ENSCJAG00000010732	KANK3
ENSCJAT00000021019	ENSCJAG00000010778	RAB11B
ENSCJAT00000021005	ENSCJAG00000010778	RAB11B
ENSCJAT00000011745	ENSCJAG00000006008	COL5A3
ENSCJAT00000011148	ENSCJAG00000005726	ICAM5
ENSCJAT00000054240	ENSCJAG00000005716	ZGLP1
ENSCJAT00000011069	ENSCJAG00000037004	unknown
ENSCJAT00000010735	ENSCJAG00000005551	ZNF653
ENSCJAT00000010697	ENSCJAG00000005530	CNN1
ENSCJAT00000062186	ENSCJAG00000005530	CNN1
ENSCJAT00000010481	ENSCJAG00000005418	unknown
ENSCJAT00000022830	ENSCJAG00000005418	unknown
ENSCJAT00000010503	ENSCJAG00000005445	ZNF136
ENSCJAT00000053222	ENSCJAG00000005445	ZNF136
ENSCJAT00000010380	ENSCJAG00000005297	unknown
ENSCJAT00000009697	ENSCJAG00000005020	BEST2
ENSCJAT00000009411	ENSCJAG00000004873	RNASEH2A
ENSCJAT00000009406	ENSCJAG00000004873	RNASEH2A
ENSCJAT00000009092	ENSCJAG00000004733	SYCE2
ENSCJAT00000008880	ENSCJAG00000004639	DAND5
ENSCJAT00000008409	ENSCJAG00000004388	NANOS3
ENSCJAT00000008381	ENSCJAG00000004372	C19orf57
ENSCJAT00000061858	ENSCJAG00000033038	unknown
ENSCJAT00000062884	ENSCJAG00000004666	DNAJB1
ENSCJAT00000060424	ENSCJAG00000004666	DNAJB1
ENSCJAT00000060565	ENSCJAG00000004666	DNAJB1
ENSCJAT00000008948	ENSCJAG00000004666	DNAJB1
ENSCJAT00000008926	ENSCJAG00000004626	unknown

ENSCJAT00000008902	ENSCJAG00000004626	unknown
ENSCJAT00000008719	ENSCJAG00000004543	ZNF333
ENSCJAT00000008690	ENSCJAG00000004537	CJA24
ENSCJAT00000043445	ENSCJAG00000022460	unknown
ENSCJAT00000004213	ENSCJAG00000002212	AKAP8
ENSCJAT00000004215	ENSCJAG00000002212	AKAP8
ENSCJAT00000004287	ENSCJAG00000002255	PGLYRP2
ENSCJAT00000004321	ENSCJAG00000002265	WIZ
ENSCJAT00000004336	ENSCJAG00000002265	WIZ
ENSCJAT00000062362	ENSCJAG00000002265	WIZ
ENSCJAT00000004327	ENSCJAG00000002265	WIZ
ENSCJAT00000027411	ENSCJAG00000014114	HSH2D
ENSCJAT00000027134	ENSCJAG00000013966	C19orf44
ENSCJAT00000027145	ENSCJAG00000013966	C19orf44
ENSCJAT00000027140	ENSCJAG00000013966	C19orf44
ENSCJAT00000018555	ENSCJAG00000009547	USE1
ENSCJAT00000018544	ENSCJAG00000009547	USE1
ENSCJAT00000061720	ENSCJAG00000009547	USE1
ENSCJAT00000018528	ENSCJAG00000009547	USE1
ENSCJAT00000018607	ENSCJAG00000009593	NR2F6
ENSCJAT00000018669	ENSCJAG00000009614	USHBP1
ENSCJAT00000018642	ENSCJAG00000009614	USHBP1
ENSCJAT00000027302	ENSCJAG00000031998	unknown
ENSCJAT00000027372	ENSCJAG00000014094	IFI30
ENSCJAT00000027538	ENSCJAG00000014171	LSM4
ENSCJAT00000038812	ENSCJAG00000019767	MEF2B
ENSCJAT00000038841	ENSCJAG00000019775	RFXANK
ENSCJAT00000038838	ENSCJAG00000019775	RFXANK
ENSCJAT00000001580	ENSCJAG00000000862	ARMC5
ENSCJAT00000001558	ENSCJAG00000000862	ARMC5
ENSCJAT00000055698	ENSCJAG00000000862	ARMC5
ENSCJAT00000054372	ENSCJAG00000000862	ARMC5
ENSCJAT00000001486	ENSCJAG00000000816	C16orf58
ENSCJAT00000001502	ENSCJAG00000000816	C16orf58
ENSCJAT00000001500	ENSCJAG00000000816	C16orf58
ENSCJAT00000057095	ENSCJAG00000000816	C16orf58
ENSCJAT00000001506	ENSCJAG00000000816	C16orf58
ENSCJAT00000001380	ENSCJAG00000000768	unknown
ENSCJAT00000001370	ENSCJAG00000038110	ZNF429
ENSCJAT00000034614	ENSCJAG00000017763	ZNF257
ENSCJAT00000014322	ENSCJAG00000007298	unknown
ENSCJAT00000054968	ENSCJAG00000001887	ISP
ENSCJAT00000003571	ENSCJAG00000001887	ISP
ENSCJAT00000035957	ENSCJAG00000018378	ZNF507
ENSCJAT00000035954	ENSCJAG00000018378	ZNF507
ENSCJAT00000036016	ENSCJAG00000018385	DPY19L3
ENSCJAT00000020145	ENSCJAG00000010352	KCTD15
ENSCJAT00000021243	ENSCJAG00000010896	PDCD2L
ENSCJAT00000021000	ENSCJAG00000010784	unknown
ENSCJAT00000021010	ENSCJAG00000010784	unknown
ENSCJAT00000020190	ENSCJAG00000010377	unknown
ENSCJAT00000019902	ENSCJAG00000010220	RBM42
ENSCJAT00000019898	ENSCJAG00000010220	RBM42
ENSCJAT00000019269	ENSCJAG00000009923	UPK1A
ENSCJAT00000019258	ENSCJAG00000009923	UPK1A
ENSCJAT00000019028	ENSCJAG00000009808	NFKBID
ENSCJAT00000018997	ENSCJAG00000009797	HCST
ENSCJAT00000018989	ENSCJAG00000009797	HCST
ENSCJAT00000018909	ENSCJAG00000009750	SYNE4

ENSCJAT00000018441	ENSCJAG00000009497	ZNF850
ENSCJAT00000058233	ENSCJAG00000009456	ZNF260
ENSCJAT00000026017	ENSCJAG00000013369	ZNF568
ENSCJAT00000026000	ENSCJAG00000013369	ZNF568
ENSCJAT00000026050	ENSCJAG00000013405	ZNF829
ENSCJAT00000026044	ENSCJAG00000013405	ZNF829
ENSCJAT00000026157	ENSCJAG00000013449	unknown
ENSCJAT00000026185	ENSCJAG00000013449	unknown
ENSCJAT00000026353	ENSCJAG00000013557	YIF1B
ENSCJAT00000026958	ENSCJAG00000013880	unknown
ENSCJAT00000027499	ENSCJAG00000014147	MED29
ENSCJAT00000027495	ENSCJAG00000014147	MED29
ENSCJAT00000064257	ENSCJAG00000014147	MED29
ENSCJAT00000027915	ENSCJAG00000014325	DYRK1B
ENSCJAT00000027920	ENSCJAG00000014325	DYRK1B
ENSCJAT00000061524	ENSCJAG00000014563	TTC9B
ENSCJAT00000028335	ENSCJAG00000014563	TTC9B
ENSCJAT00000028897	ENSCJAG00000014833	ADCK4
ENSCJAT00000028910	ENSCJAG00000014833	ADCK4
ENSCJAT00000052818	ENSCJAG00000014833	ADCK4
ENSCJAT00000064240	ENSCJAG00000035992	unknown
ENSCJAT00000028978	ENSCJAG00000014887	EGLN2
ENSCJAT00000039578	ENSCJAG00000020149	TGFB1
ENSCJAT00000057651	ENSCJAG00000020149	TGFB1
ENSCJAT00000039580	ENSCJAG00000020149	TGFB1
ENSCJAT00000024705	ENSCJAG00000012746	ZNF574
ENSCJAT00000024840	ENSCJAG00000012791	unknown
ENSCJAT00000060185	ENSCJAG00000012791	unknown
ENSCJAT00000025028	ENSCJAG00000012914	PRR19
ENSCJAT00000025055	ENSCJAG00000012919	TMEM145
ENSCJAT00000025039	ENSCJAG00000012919	TMEM145
ENSCJAT00000025125	ENSCJAG00000012952	CNFN
ENSCJAT00000053827	ENSCJAG00000012952	CNFN
ENSCJAT00000026516	ENSCJAG00000013643	TEX101
ENSCJAT00000026140	ENSCJAG00000013416	SMG9
ENSCJAT00000025902	ENSCJAG00000032912	ZNF283
ENSCJAT00000025909	ENSCJAG00000013331	unknown
ENSCJAT00000025959	ENSCJAG00000013331	unknown
ENSCJAT00000025965	ENSCJAG00000013321	ZNF226
ENSCJAT00000025946	ENSCJAG00000013321	ZNF226
ENSCJAT00000025886	ENSCJAG00000013321	ZNF226
ENSCJAT00000025882	ENSCJAG00000013321	ZNF226
ENSCJAT00000025873	ENSCJAG00000013321	ZNF226
ENSCJAT00000025935	ENSCJAG00000013321	ZNF226
ENSCJAT00000055310	ENSCJAG00000013239	ZNF180
ENSCJAT00000025734	ENSCJAG00000013239	ZNF180
ENSCJAT00000024715	ENSCJAG00000012716	ERCC2
ENSCJAT00000024701	ENSCJAG00000012716	ERCC2
ENSCJAT00000024712	ENSCJAG00000012716	ERCC2
ENSCJAT00000024323	ENSCJAG00000012560	PPM1N
ENSCJAT00000017168	ENSCJAG00000012422	GIPR
ENSCJAT00000024142	ENSCJAG00000012422	GIPR
ENSCJAT00000023797	ENSCJAG00000012268	RSPH6A
ENSCJAT00000023791	ENSCJAG00000012268	RSPH6A
ENSCJAT00000005139	ENSCJAG00000011891	PPP5C
ENSCJAT00000023315	ENSCJAG00000011891	PPP5C
ENSCJAT00000023289	ENSCJAG00000011891	PPP5C
ENSCJAT00000022959	ENSCJAG00000011828	DACT3
ENSCJAT00000058454	ENSCJAG00000011828	DACT3

ENSCJAT00000022951	ENSCJAG00000011828	DACT3
ENSCJAT00000022935	ENSCJAG00000011814	PRKD2
ENSCJAT00000059076	ENSCJAG00000011601	ZC3H4
ENSCJAT00000000804	ENSCJAG00000000440	GRWD1
ENSCJAT00000000712	ENSCJAG00000000396	KCNJ14
ENSCJAT00000000672	ENSCJAG00000000366	SPHK2
ENSCJAT00000000670	ENSCJAG00000000366	SPHK2
ENSCJAT00000044149	ENSCJAG00000023164	FUT2
ENSCJAT00000044147	ENSCJAG00000023162	NTF4
ENSCJAT00000000269	ENSCJAG00000000136	HRC
ENSCJAT00000000264	ENSCJAG00000000136	HRC
ENSCJAT00000000126	ENSCJAG00000000069	unknown
ENSCJAT00000034365	ENSCJAG00000017641	PRRG2
ENSCJAT00000034361	ENSCJAG00000017641	PRRG2
ENSCJAT00000034371	ENSCJAG00000017641	PRRG2
ENSCJAT00000007004	ENSCJAG00000003663	BCL2L12
ENSCJAT00000053720	ENSCJAG00000003663	BCL2L12
ENSCJAT00000008726	ENSCJAG00000003806	POLD1
ENSCJAT00000008708	ENSCJAG00000003806	POLD1
ENSCJAT00000008019	ENSCJAG00000003943	MED25
ENSCJAT00000008024	ENSCJAG00000003943	MED25
ENSCJAT00000054902	ENSCJAG00000004201	unknown
ENSCJAT00000052996	ENSCJAG00000004201	unknown
ENSCJAT00000008059	ENSCJAG00000004201	unknown
ENSCJAT00000008053	ENSCJAG00000004201	unknown
ENSCJAT00000008438	ENSCJAG00000004417	NAPSA
ENSCJAT00000008450	ENSCJAG00000004417	NAPSA
ENSCJAT00000033469	ENSCJAG00000017216	ASPDH
ENSCJAT00000012229	ENSCJAG00000006295	KLK10
ENSCJAT00000012321	ENSCJAG00000006302	KLK11
ENSCJAT00000012307	ENSCJAG00000006302	KLK11
ENSCJAT00000012316	ENSCJAG00000006302	KLK11
ENSCJAT00000036422	ENSCJAG00000018562	IGLON5
ENSCJAT00000036418	ENSCJAG00000018562	IGLON5
ENSCJAT00000036309	ENSCJAG00000018511	unknown
ENSCJAT00000036301	ENSCJAG00000018511	unknown
ENSCJAT00000036284	ENSCJAG00000018511	unknown
ENSCJAT00000003979	ENSCJAG00000002084	ZNF577
ENSCJAT00000003927	ENSCJAG00000002058	ZNF841
ENSCJAT00000003900	ENSCJAG00000002045	ZNF615
ENSCJAT00000035674	ENSCJAG00000018238	ZNF347
ENSCJAT00000036166	ENSCJAG00000018393	PRKCG
ENSCJAT00000060209	ENSCJAG00000018393	PRKCG
ENSCJAT00000036121	ENSCJAG00000018393	PRKCG
ENSCJAT00000036140	ENSCJAG00000018393	PRKCG
ENSCJAT00000036145	ENSCJAG00000018393	PRKCG
ENSCJAT00000054402	ENSCJAG00000018393	PRKCG
ENSCJAT00000057545	ENSCJAG00000001094	MA_M007_JSM4E6A6F
ENSCJAT00000002009	ENSCJAG00000001094	MA_M007_JSM4E6A6F
ENSCJAT00000002013	ENSCJAG00000001106	LENG1
ENSCJAT00000056187	ENSCJAG00000001106	LENG1
ENSCJAT00000002016	ENSCJAG00000001106	LENG1
ENSCJAT00000034512	ENSCJAG00000017703	unknown
ENSCJAT00000034416	ENSCJAG00000017672	unknown
ENSCJAT00000034420	ENSCJAG00000017672	unknown
ENSCJAT00000034413	ENSCJAG00000017672	unknown
ENSCJAT00000055206	ENSCJAG00000017672	unknown
ENSCJAT00000001555	ENSCJAG00000000858	IL11
ENSCJAT00000058810	ENSCJAG00000000858	IL11

ENSCJAT00000039875	ENSCJAG00000020302	unknown
ENSCJAT00000019507	ENSCJAG00000010044	PEG3
ENSCJAT00000019525	ENSCJAG00000010044	PEG3
ENSCJAT00000000036	ENSCJAG00000000026	unknown
ENSCJAT00000059206	ENSCJAG00000000026	unknown
ENSCJAT00000000041	ENSCJAG00000000026	unknown
ENSCJAT00000000678	ENSCJAG00000000374	ZNF606
ENSCJAT00000026097	ENSCJAG00000000374	ZNF606
ENSCJAT00000000600	ENSCJAG00000000329	ZNF544
ENSCJAT00000056748	ENSCJAG00000000329	ZNF544
ENSCJAT00000039135	ENSCJAG00000019931	NOTCH2
ENSCJAT00000039197	ENSCJAG00000019954	PDE4DIP
ENSCJAT00000039246	ENSCJAG00000019954	PDE4DIP
ENSCJAT00000004144	ENSCJAG00000002179	POLR3GL
ENSCJAT00000004138	ENSCJAG00000002179	POLR3GL
ENSCJAT00000004530	ENSCJAG00000002370	ACP6
ENSCJAT00000004520	ENSCJAG00000002370	ACP6
ENSCJAT00000019623	ENSCJAG00000010097	TBC1D2B
ENSCJAT00000055218	ENSCJAG00000010115	CIB2
ENSCJAT00000052739	ENSCJAG00000010115	CIB2
ENSCJAT00000019677	ENSCJAG00000010115	CIB2
ENSCJAT00000020171	ENSCJAG00000010363	HYKK
ENSCJAT00000020160	ENSCJAG00000010363	HYKK
ENSCJAT00000061104	ENSCJAG00000010437	CHRNA3
ENSCJAT00000057288	ENSCJAG00000010437	CHRNA3
ENSCJAT00000020322	ENSCJAG00000010437	CHRNA3
ENSCJAT00000055174	ENSCJAG00000022181	HIST2H2BF
ENSCJAT00000063400	ENSCJAG00000022181	HIST2H2BF
ENSCJAT00000059182	ENSCJAG00000022181	HIST2H2BF
ENSCJAT00000001570	ENSCJAG00000000867	PLEKHO1
ENSCJAT00000001567	ENSCJAG00000000867	PLEKHO1
ENSCJAT00000019185	ENSCJAG00000009890	C1orf54
ENSCJAT00000019176	ENSCJAG00000009890	C1orf54
ENSCJAT00000019183	ENSCJAG00000009890	C1orf54
ENSCJAT00000012621	ENSCJAG00000006429	TARS2
ENSCJAT00000012563	ENSCJAG00000006429	TARS2
ENSCJAT00000012592	ENSCJAG00000006429	TARS2
ENSCJAT00000012613	ENSCJAG00000006429	TARS2
ENSCJAT00000012971	ENSCJAG00000006621	ENSA
ENSCJAT00000013265	ENSCJAG00000006790	CTSS
ENSCJAT00000013268	ENSCJAG00000006790	CTSS
ENSCJAT00000013534	ENSCJAG00000006839	ARNT
ENSCJAT00000013569	ENSCJAG00000006839	ARNT
ENSCJAT00000013596	ENSCJAG00000006839	ARNT
ENSCJAT00000063095	ENSCJAG00000006972	SETDB1
ENSCJAT00000013835	ENSCJAG00000007043	CERS2
ENSCJAT00000013827	ENSCJAG00000007043	CERS2
ENSCJAT00000013948	ENSCJAG00000007125	C1orf56
ENSCJAT00000015105	ENSCJAG00000007659	CGN
ENSCJAT00000057793	ENSCJAG00000007659	CGN
ENSCJAT00000015454	ENSCJAG00000007924	unknown
ENSCJAT00000015511	ENSCJAG00000007939	OAZ3
ENSCJAT00000058544	ENSCJAG00000010012	TCHH
ENSCJAT00000012498	ENSCJAG00000031716	RPTN
ENSCJAT00000054829	ENSCJAG00000031716	RPTN
ENSCJAT00000052374	ENSCJAG00000031716	RPTN
ENSCJAT00000032271	ENSCJAG00000016606	unknown
ENSCJAT00000031530	ENSCJAG00000036359	unknown
ENSCJAT00000058607	ENSCJAG00000036359	unknown

ENSCJAT00000023403	ENSCJAG00000037822	unknown
ENSCJAT00000011343	ENSCJAG00000005847	S100A12
ENSCJAT00000062313	ENSCJAG00000010417	S100A13
ENSCJAT00000020281	ENSCJAG00000010417	S100A13
ENSCJAT00000020192	ENSCJAG00000010373	SNAPIN
ENSCJAT00000020172	ENSCJAG00000010264	ILF2
ENSCJAT00000020178	ENSCJAG00000010264	ILF2
ENSCJAT00000018779	ENSCJAG00000009677	UBAP2L
ENSCJAT00000017249	ENSCJAG00000008715	THBS3
ENSCJAT00000052307	ENSCJAG00000008715	THBS3
ENSCJAT00000061692	ENSCJAG00000008715	THBS3
ENSCJAT00000017129	ENSCJAG00000008715	THBS3
ENSCJAT00000055690	ENSCJAG00000008486	SCAMP3
ENSCJAT00000062526	ENSCJAG00000008486	SCAMP3
ENSCJAT00000016587	ENSCJAG00000008486	SCAMP3
ENSCJAT00000016569	ENSCJAG00000008486	SCAMP3
ENSCJAT00000016410	ENSCJAG00000008422	HCN3
ENSCJAT00000016407	ENSCJAG00000008422	HCN3
ENSCJAT00000016255	ENSCJAG00000008263	RUSC1
ENSCJAT00000016237	ENSCJAG00000008263	RUSC1
ENSCJAT00000056073	ENSCJAG00000008263	RUSC1
ENSCJAT00000016226	ENSCJAG00000008263	RUSC1
ENSCJAT00000016097	ENSCJAG00000035507	YY1AP1
ENSCJAT00000061249	ENSCJAG00000035507	YY1AP1
ENSCJAT00000015588	ENSCJAG00000035507	YY1AP1
ENSCJAT00000018673	ENSCJAG00000007969	SYT11
ENSCJAT00000015546	ENSCJAG00000007969	SYT11
ENSCJAT00000015259	ENSCJAG00000007755	UBQLN4
ENSCJAT00000015270	ENSCJAG00000007755	UBQLN4
ENSCJAT00000063133	ENSCJAG00000005605	SEMA4A
ENSCJAT00000010862	ENSCJAG00000005605	SEMA4A
ENSCJAT00000010851	ENSCJAG00000005605	SEMA4A
ENSCJAT00000010881	ENSCJAG00000005625	SLC25A44
ENSCJAT00000054447	ENSCJAG00000005776	CCT3
ENSCJAT00000011294	ENSCJAG00000005776	CCT3
ENSCJAT00000062775	ENSCJAG00000005776	CCT3
ENSCJAT00000063707	ENSCJAG00000005776	CCT3
ENSCJAT00000011281	ENSCJAG00000005776	CCT3
ENSCJAT00000011337	ENSCJAG00000005848	unknown
ENSCJAT00000055792	ENSCJAG00000006002	TTC24
ENSCJAT00000011663	ENSCJAG00000006002	TTC24
ENSCJAT00000012319	ENSCJAG00000006311	INSRR
ENSCJAT00000012852	ENSCJAG00000006570	FCRL5
ENSCJAT00000012833	ENSCJAG00000006570	FCRL5
ENSCJAT00000012816	ENSCJAG00000006570	FCRL5
ENSCJAT00000013243	ENSCJAG00000006783	unknown
ENSCJAT00000012059	ENSCJAG00000006175	CFAP45
ENSCJAT00000012038	ENSCJAG00000006175	CFAP45
ENSCJAT00000011783	ENSCJAG00000006022	ATP1A2
ENSCJAT00000011668	ENSCJAG00000005980	CASQ1
ENSCJAT00000011089	ENSCJAG00000005699	SLAMF1
ENSCJAT00000011086	ENSCJAG00000005699	SLAMF1
ENSCJAT00000010983	ENSCJAG00000005659	SLAMF7
ENSCJAT00000010960	ENSCJAG00000005659	SLAMF7
ENSCJAT00000010988	ENSCJAG00000005659	SLAMF7
ENSCJAT00000010980	ENSCJAG00000005659	SLAMF7
ENSCJAT00000010957	ENSCJAG00000005659	SLAMF7
ENSCJAT00000011000	ENSCJAG00000005659	SLAMF7
ENSCJAT00000010966	ENSCJAG00000005659	SLAMF7

ENSCJAT00000013082	ENSCJAG00000005659	SLAMF7
ENSCJAT00000010493	ENSCJAG00000005396	USF1
ENSCJAT00000010462	ENSCJAG00000005396	USF1
ENSCJAT00000010516	ENSCJAG00000005452	PVRL4
ENSCJAT00000010536	ENSCJAG00000005452	PVRL4
ENSCJAT00000010528	ENSCJAG00000005452	PVRL4
ENSCJAT00000036871	ENSCJAG00000004966	NDUFS2
ENSCJAT00000008752	ENSCJAG00000004556	OLFML2B
ENSCJAT00000008734	ENSCJAG00000004556	OLFML2B
ENSCJAT00000011743	ENSCJAG00000006043	PDC
ENSCJAT00000011696	ENSCJAG00000005988	C1orf27
ENSCJAT00000011704	ENSCJAG00000005988	C1orf27
ENSCJAT00000011692	ENSCJAG00000005988	C1orf27
ENSCJAT00000011552	ENSCJAG00000005872	TPR
ENSCJAT00000011335	ENSCJAG00000005872	TPR
ENSCJAT00000011607	ENSCJAG00000005872	TPR
ENSCJAT00000011219	ENSCJAG00000005660	HMCN1
ENSCJAT00000010765	ENSCJAG00000005544	SWT1
ENSCJAT00000010661	ENSCJAG00000005456	TRMT1L
ENSCJAT00000010677	ENSCJAG00000005456	TRMT1L
ENSCJAT00000010575	ENSCJAG00000005456	TRMT1L
ENSCJAT00000062224	ENSCJAG00000005265	RGL1
ENSCJAT00000010269	ENSCJAG00000005265	RGL1
ENSCJAT00000010286	ENSCJAG00000005328	APOBEC4
ENSCJAT00000009838	ENSCJAG00000005053	LAMC2
ENSCJAT00000060119	ENSCJAG00000005053	LAMC2
ENSCJAT00000009825	ENSCJAG00000005053	LAMC2
ENSCJAT00000055158	ENSCJAG00000004821	DHX9
ENSCJAT00000057621	ENSCJAG00000004821	DHX9
ENSCJAT00000009284	ENSCJAG00000004793	NPL
ENSCJAT00000009262	ENSCJAG00000004793	NPL
ENSCJAT00000009265	ENSCJAG00000004793	NPL
ENSCJAT00000009288	ENSCJAG00000004793	NPL
ENSCJAT00000053949	ENSCJAG00000004748	RGS16
ENSCJAT00000009136	ENSCJAG00000004748	RGS16
ENSCJAT00000008905	ENSCJAG00000004652	ZNF648
ENSCJAT00000008430	ENSCJAG00000004391	MR1
ENSCJAT00000008460	ENSCJAG00000004391	MR1
ENSCJAT00000008452	ENSCJAG00000004391	MR1
ENSCJAT00000008295	ENSCJAG00000004339	SH2D1B
ENSCJAT00000008196	ENSCJAG00000004264	UAP1
ENSCJAT00000008200	ENSCJAG00000004264	UAP1
ENSCJAT00000007853	ENSCJAG00000004050	NUF2
ENSCJAT00000057827	ENSCJAG00000008093	FAM163A
ENSCJAT00000057425	ENSCJAG00000008093	FAM163A
ENSCJAT00000015763	ENSCJAG00000008093	FAM163A
ENSCJAT00000016273	ENSCJAG00000008366	TOR3A
ENSCJAT00000016271	ENSCJAG00000008366	TOR3A
ENSCJAT00000017751	ENSCJAG00000009106	RC3H1
ENSCJAT00000017771	ENSCJAG00000009106	RC3H1
ENSCJAT00000017871	ENSCJAG00000009215	ZBTB37
ENSCJAT00000017823	ENSCJAG00000009215	ZBTB37
ENSCJAT00000017884	ENSCJAG00000009215	ZBTB37
ENSCJAT00000017849	ENSCJAG00000009215	ZBTB37
ENSCJAT00000018481	ENSCJAG00000009537	C1orf105
ENSCJAT00000018970	ENSCJAG00000009773	FMO4
ENSCJAT00000019312	ENSCJAG00000009943	METTL11B
ENSCJAT00000019489	ENSCJAG00000009954	KIFAP3
ENSCJAT00000019455	ENSCJAG00000009954	KIFAP3

ENSCJAT00000019475	ENSCJAG00000009954	KIFAP3
ENSCJAT00000063743	ENSCJAG00000009954	KIFAP3
ENSCJAT00000019657	ENSCJAG00000010088	C1orf112
ENSCJAT00000058437	ENSCJAG00000010174	SELL
ENSCJAT00000019790	ENSCJAG00000010174	SELL
ENSCJAT00000020148	ENSCJAG00000010354	DPT
ENSCJAT00000032735	ENSCJAG00000016812	TBX19
ENSCJAT00000062333	ENSCJAG00000017048	CD247
ENSCJAT00000033202	ENSCJAG00000017048	CD247
ENSCJAT00000031152	ENSCJAG00000016018	TADA1
ENSCJAT00000008985	ENSCJAG00000004656	RXRG
ENSCJAT00000008988	ENSCJAG00000004656	RXRG
ENSCJAT00000008979	ENSCJAG00000004656	RXRG
ENSCJAT00000016968	ENSCJAG00000008733	RGS18
ENSCJAT00000016908	ENSCJAG00000008699	RGS13
ENSCJAT00000056782	ENSCJAG00000008699	RGS13
ENSCJAT00000016662	ENSCJAG00000008562	GLRX2
ENSCJAT00000016666	ENSCJAG00000008562	GLRX2
ENSCJAT00000024337	ENSCJAG00000012514	ABCC11
ENSCJAT00000024191	ENSCJAG00000012514	ABCC11
ENSCJAT00000024335	ENSCJAG00000012514	ABCC11
ENSCJAT00000024340	ENSCJAG00000012514	ABCC11
ENSCJAT00000008427	ENSCJAG00000004403	unknown
ENSCJAT00000002792	ENSCJAG00000001445	RPGRIP1L
ENSCJAT00000002809	ENSCJAG00000001445	RPGRIP1L
ENSCJAT00000002805	ENSCJAG00000001445	RPGRIP1L
ENSCJAT00000025123	ENSCJAG00000012929	MMP2
ENSCJAT00000025114	ENSCJAG00000012929	MMP2
ENSCJAT00000025107	ENSCJAG00000012929	MMP2
ENSCJAT00000024999	ENSCJAG00000012830	SLC6A2
ENSCJAT00000024995	ENSCJAG00000012830	SLC6A2
ENSCJAT00000024762	ENSCJAG00000012765	GNAO1
ENSCJAT00000024091	ENSCJAG00000012357	HERPUD1
ENSCJAT00000024054	ENSCJAG00000012357	HERPUD1
ENSCJAT00000024067	ENSCJAG00000012357	HERPUD1
ENSCJAT00000024000	ENSCJAG00000012357	HERPUD1
ENSCJAT00000023528	ENSCJAG00000012116	unknown
ENSCJAT00000023312	ENSCJAG00000011994	DOK4
ENSCJAT00000023010	ENSCJAG00000011825	KATNB1
ENSCJAT00000063466	ENSCJAG00000011752	TEPP
ENSCJAT00000022702	ENSCJAG00000011673	CSNK2A2
ENSCJAT00000022646	ENSCJAG00000011662	CCDC113
ENSCJAT00000022650	ENSCJAG00000011662	CCDC113
ENSCJAT00000056372	ENSCJAG00000011662	CCDC113
ENSCJAT00000022604	ENSCJAG00000011635	GINS3
ENSCJAT00000022596	ENSCJAG00000011635	GINS3
ENSCJAT00000058752	ENSCJAG00000011635	GINS3
ENSCJAT00000061964	ENSCJAG00000011635	GINS3
ENSCJAT00000056243	ENSCJAG00000011285	SLC38A7
ENSCJAT00000022009	ENSCJAG00000011285	SLC38A7
ENSCJAT00000007329	ENSCJAG00000011285	SLC38A7
ENSCJAT00000032747	ENSCJAG00000016827	CMTM2
ENSCJAT00000032749	ENSCJAG00000016827	CMTM2
ENSCJAT00000032678	ENSCJAG00000016771	DYNC1LI2
ENSCJAT00000032673	ENSCJAG00000016771	DYNC1LI2
ENSCJAT00000032668	ENSCJAG00000016771	DYNC1LI2
ENSCJAT00000032626	ENSCJAG00000016771	DYNC1LI2
ENSCJAT00000032119	ENSCJAG00000016510	C16orf70
ENSCJAT00000032074	ENSCJAG00000016502	TRADD

ENSCJAT00000031145	ENSCJAG00000016015	NUTF2
ENSCJAT00000030488	ENSCJAG00000015670	SLC7A6OS
ENSCJAT00000029697	ENSCJAG00000015244	NQO1
ENSCJAT00000004117	ENSCJAG00000015244	NQO1
ENSCJAT00000025641	ENSCJAG00000015244	NQO1
ENSCJAT00000052515	ENSCJAG00000031829	PMFBP1
ENSCJAT00000053147	ENSCJAG00000031829	PMFBP1
ENSCJAT00000059571	ENSCJAG00000031829	PMFBP1
ENSCJAT00000029383	ENSCJAG00000015085	DHODH
ENSCJAT00000029275	ENSCJAG00000015028	AP1G1
ENSCJAT00000035501	ENSCJAG00000015005	PHLPP2
ENSCJAT00000030579	ENSCJAG00000015005	PHLPP2
ENSCJAT00000029250	ENSCJAG00000015005	PHLPP2
ENSCJAT00000029245	ENSCJAG00000015005	PHLPP2
ENSCJAT00000042397	ENSCJAG00000014996	MARVELD3
ENSCJAT00000053083	ENSCJAG00000014972	unknown
ENSCJAT00000029138	ENSCJAG00000014972	unknown
ENSCJAT00000029146	ENSCJAG00000014972	unknown
ENSCJAT00000028894	ENSCJAG00000014831	MTSS1L
ENSCJAT00000058014	ENSCJAG00000014831	MTSS1L
ENSCJAT00000028853	ENSCJAG00000014773	SF3B3
ENSCJAT00000028859	ENSCJAG00000014773	SF3B3
ENSCJAT00000028586	ENSCJAG00000014678	FUK
ENSCJAT00000059954	ENSCJAG00000014678	FUK
ENSCJAT00000027902	ENSCJAG00000014349	ZFP1
ENSCJAT00000027897	ENSCJAG00000014349	ZFP1
ENSCJAT00000027754	ENSCJAG00000014283	unknown
ENSCJAT00000027704	ENSCJAG00000014253	unknown
ENSCJAT00000027652	ENSCJAG00000014213	KARS
ENSCJAT00000027669	ENSCJAG00000014213	KARS
ENSCJAT00000027662	ENSCJAG00000014213	KARS
ENSCJAT00000027255	ENSCJAG00000014027	unknown
ENSCJAT00000032442	ENSCJAG00000016683	CMC2
ENSCJAT00000032234	ENSCJAG00000016571	GAN
ENSCJAT00000007542	ENSCJAG00000003920	HSDL1
ENSCJAT00000007531	ENSCJAG00000003920	HSDL1
ENSCJAT00000007583	ENSCJAG00000003933	DNAAF1
ENSCJAT00000007571	ENSCJAG00000003933	DNAAF1
ENSCJAT00000007578	ENSCJAG00000003933	DNAAF1
ENSCJAT00000007557	ENSCJAG00000003933	DNAAF1
ENSCJAT00000007646	ENSCJAG00000003981	ADAD2
ENSCJAT00000007639	ENSCJAG00000003981	ADAD2
ENSCJAT00000007902	ENSCJAG00000004090	CRISPLD2
ENSCJAT00000007969	ENSCJAG00000004146	FAM92B
ENSCJAT00000062644	ENSCJAG00000004146	FAM92B
ENSCJAT00000056714	ENSCJAG00000017301	GSE1
ENSCJAT00000033288	ENSCJAG00000017133	FOXL1
ENSCJAT00000016954	ENSCJAG00000008702	unknown
ENSCJAT00000025200	ENSCJAG00000012986	unknown
ENSCJAT00000025196	ENSCJAG00000012986	unknown
ENSCJAT00000025353	ENSCJAG00000013021	CBFA2T3
ENSCJAT00000058115	ENSCJAG00000011124	PEAK1
ENSCJAT00000021687	ENSCJAG00000011124	PEAK1
ENSCJAT00000021169	ENSCJAG00000010855	ISL2

Supplemental table 3. The list of the 13,200 newly found transcripts. The first, second, and third columns represent Ensembl transcript_id, gene_id, and gene_name, respectively. Further, the 5,228 genes located in the filled gaps in the improved genome sequence are annotated “filled”.

ENST00000417977	ENSG00000237254	TRBV30	
ENST00000390426	ENSG00000211778	TRAV4	
ENST00000390423	ENSG00000256553	TRAV1-2	
ENST00000390387	ENSG00000237702	TRBV3-1	
ENST00000390353	ENSG00000211706	TRBV6-1	
ENST00000547918	ENSG00000211707	TRBV7-1	
ENST00000390368	ENSG00000211721	TRBV6-5	
ENST00000390362	ENSG00000211715	TRBV5-3	filled
ENST00000390424	ENSG00000211776	TRAV2	
ENST00000390430	ENSG00000211782	TRAV8-1	
ENST00000390376	ENSG00000253534	TRBV6-8	
ENST00000623991	ENSG00000279000	OR10A6	
ENST00000623813	ENSG00000279000	OR10A6	
ENST00000377171	ENSG00000204702	OR2J1	
ENST00000435756	ENSG00000241356	OR5G3	
ENST00000467490	ENSG00000164616	FBXL21	filled
ENST00000498734	ENSG00000164616	FBXL21	filled
ENST00000620812	ENSG00000164616	FBXL21	
ENST00000508166	ENSG00000249948	GBA3	filled
ENST00000503442	ENSG00000249948	GBA3	filled
ENST00000511446	ENSG00000249948	GBA3	
ENST00000508264	ENSG00000249948	GBA3	
ENST00000610628	ENSG00000249948	GBA3	filled
ENST00000613293	ENSG00000249948	GBA3	filled
ENST00000623373	ENSG00000197558	SSPO	filled
ENST00000486824	ENSG00000197558	SSPO	
ENST00000477518	ENSG00000197558	SSPO	
ENST00000493502	ENSG00000197558	SSPO	
ENST00000475488	ENSG00000197558	SSPO	filled
ENST00000378016	ENSG00000197558	SSPO	filled
ENST00000624978	ENSG00000279270	OR52R1	
ENST00000624198	ENSG00000279270	OR52R1	
ENST00000606380	ENSG00000272395	IFNL4	
ENST00000623530	ENSG00000280094	OR1B1	
ENST00000624439	ENSG00000280094	OR1B1	
ENST00000390293	ENSG00000211647	IGLV5-48	
ENST00000390283	ENSG00000211638	IGLV8-61	
ENST00000361272	ENSG00000198791	CNOT7	filled
ENST00000375712	ENSG00000101350	KIF3B	filled
ENST00000359676	ENSG00000197183	NOL4L	filled
ENST00000475781	ENSG00000197183	NOL4L	
ENST00000375678	ENSG00000197183	NOL4L	filled
ENST00000470428	ENSG00000197183	NOL4L	filled
ENST00000621426	ENSG00000197183	NOL4L	filled
ENST00000616976	ENSG00000197183	NOL4L	
ENST00000514088	ENSG00000113494	PRLR	filled
ENST00000509140	ENSG00000113494	PRLR	filled
ENST00000542609	ENSG00000113494	PRLR	filled
ENST00000376873	ENSG00000134873	CLDN10	filled
ENST00000451303	ENSG00000119396	RAB14	filled
ENST00000495360	ENSG00000137074	APTX	filled
ENST00000460945	ENSG00000137074	APTX	filled
ENST00000372520	ENSG00000168612	ZSWIM1	
ENST00000371790	ENSG00000152779	SLC16A12	
ENST00000343986	ENSG00000171858	RPS21	
ENST00000602452	ENSG00000269964	MEI4	filled
ENST00000374296	ENSG00000182749	PAQR7	
ENST00000374045	ENSG00000181634	TNFSF15	filled
ENST00000532250	ENSG00000133805	AMPD3	filled
ENST00000509025	ENSG00000134986	NREP	

ENST00000395634	ENSG00000134986	NREP	filled
ENST00000450761	ENSG00000134986	NREP	filled
ENST00000543153	ENSG00000111445	RFC5	
ENST00000447098	ENSG00000117425	PTCH2	filled
ENST00000481747	ENSG00000125895	TMEM74B	
ENST00000508009	ENSG00000167085	PHB	
ENST00000324631	ENSG00000165995	CACNB2	filled
ENST00000352115	ENSG00000165995	CACNB2	filled
ENST00000377328	ENSG00000165995	CACNB2	filled
ENST00000468177	ENSG00000165995	CACNB2	
ENST00000282343	ENSG00000165995	CACNB2	filled
ENST00000377331	ENSG00000165995	CACNB2	filled
ENST00000621279	ENSG00000275619	C14orf142	filled
ENST00000522370	ENSG00000171530	TBCA	filled
ENST00000513200	ENSG00000185261	KIAA0825	filled
ENST00000427991	ENSG00000185261	KIAA0825	filled
ENST00000532292	ENSG00000254585	MAGEL2	
ENST00000498004	ENSG00000169239	CA5B	filled
ENST00000450036	ENSG00000103202	NME4	
ENST00000423795	ENSG00000106034	CPED1	filled
ENST00000621307	ENSG00000277722	NDUFA3	
ENST00000528048	ENSG00000204370	SDHD	
ENST00000525291	ENSG00000204370	SDHD	
ENST00000597441	ENSG00000167749	KLK4	filled
ENST00000481143	ENSG00000198947	DMD	
ENST00000378677	ENSG00000198947	DMD	filled
ENST00000357033	ENSG00000198947	DMD	filled
ENST00000474231	ENSG00000198947	DMD	filled
ENST00000488902	ENSG00000198947	DMD	filled
ENST00000288447	ENSG00000198947	DMD	filled
ENST00000472681	ENSG00000198947	DMD	filled
ENST00000378707	ENSG00000198947	DMD	filled
ENST00000541735	ENSG00000198947	DMD	filled
ENST00000359836	ENSG00000198947	DMD	filled
ENST00000620040	ENSG00000198947	DMD	filled
ENST00000619831	ENSG00000198947	DMD	filled
ENST00000373457	ENSG00000169155	ZBTB43	
ENST00000497064	ENSG00000169155	ZBTB43	
ENST00000327228	ENSG00000177143	CETN1	
ENST00000562874	ENSG00000189091	SF3B3	
ENST00000476320	ENSG00000186577	C6orf1	
ENST00000481533	ENSG00000186577	C6orf1	
ENST00000413013	ENSG00000186577	C6orf1	
ENST00000394990	ENSG00000186577	C6orf1	
ENST00000335352	ENSG00000186577	C6orf1	
ENST00000468145	ENSG00000186577	C6orf1	
ENST00000316286	ENSG00000197362	ZNF786	filled
ENST00000320451	ENSG00000197961	ZNF121	
ENST00000462769	ENSG00000167614	TTYH1	
ENST00000467939	ENSG00000167614	TTYH1	
ENST00000375050	ENSG00000204304	PBX2	filled
ENST00000467052	ENSG00000204291	COL15A1	
ENST00000416026	ENSG00000197885	NKIRAS1	
ENST00000543050	ENSG00000110200	ANAPC15	filled
ENST00000538117	ENSG00000110200	ANAPC15	filled
ENST00000543587	ENSG00000110200	ANAPC15	filled
ENST00000545680	ENSG00000110200	ANAPC15	filled
ENST00000227618	ENSG00000110200	ANAPC15	filled
ENST00000542531	ENSG00000110200	ANAPC15	filled
ENST00000535234	ENSG00000110200	ANAPC15	filled

ENST00000538393	ENSG00000110200	ANAPC15	filled
ENST00000535503	ENSG00000110200	ANAPC15	filled
ENST00000538919	ENSG00000110200	ANAPC15	filled
ENST00000537644	ENSG00000110200	ANAPC15	filled
ENST00000545333	ENSG00000110200	ANAPC15	filled
ENST00000539395	ENSG00000110200	ANAPC15	
ENST00000523566	ENSG00000171320	ESCO2	
ENST00000305188	ENSG00000171320	ESCO2	filled
ENST00000522378	ENSG00000171320	ESCO2	filled
ENST00000341552	ENSG00000181378	CCDC108	
ENST00000474601	ENSG00000181378	CCDC108	
ENST00000453220	ENSG00000181378	CCDC108	
ENST00000546769	ENSG00000057294	PKP2	
ENST00000507892	ENSG00000204536	CCHCR1	
ENST00000601863	ENSG00000277871	AF186192.2	
ENST00000515764	ENSG00000250317	SMIM20	filled
ENST00000337478	ENSG00000148841	ITPRIP	filled
ENST00000358187	ENSG00000148841	ITPRIP	
ENST00000458723	ENSG00000148841	ITPRIP	
ENST00000278071	ENSG00000148841	ITPRIP	
ENST00000443275	ENSG00000143190	POU2F1	filled
ENST00000463633	ENSG00000163746	PLSCR2	filled
ENST00000470285	ENSG00000005436	GCFC2	
ENST00000605930	ENSG00000096433	ITPR3	filled
ENST00000371927	ENSG00000138134	STAMBPL1	
ENST00000622683	ENSG00000275832	ARHGAP23	filled
ENST00000620417	ENSG00000275832	ARHGAP23	filled
ENST00000616767	ENSG00000275832	ARHGAP23	
ENST00000618942	ENSG00000275832	ARHGAP23	
ENST00000616909	ENSG00000275832	ARHGAP23	filled
ENST00000376634	ENSG00000197969	VPS13A	filled
ENST00000376636	ENSG00000197969	VPS13A	filled
ENST00000360280	ENSG00000197969	VPS13A	filled
ENST00000357409	ENSG00000197969	VPS13A	filled
ENST00000471439	ENSG00000197969	VPS13A	
ENST00000461724	ENSG00000144802	NFKBIZ	filled
ENST00000483180	ENSG00000144802	NFKBIZ	filled
ENST00000394054	ENSG00000144802	NFKBIZ	filled
ENST00000461918	ENSG00000084234	APLP2	filled
ENST00000396057	ENSG00000171956	FOXB1	filled
ENST00000624977	ENSG00000280342	PCDH11Y	filled
ENST00000249075	ENSG00000128342	LIF	
ENST00000497709	ENSG00000155111	CDK19	
ENST00000421422	ENSG00000174306	ZHX3	
ENST00000559296	ENSG00000174306	ZHX3	filled
ENST00000574289	ENSG00000262473	GART	
ENST00000575239	ENSG00000262473	GART	
ENST00000334209	ENSG00000186732	MPPED1	filled
ENST00000447567	ENSG00000186732	MPPED1	
ENST00000480239	ENSG00000186732	MPPED1	
ENST00000587492	ENSG00000131848	ZSCAN5A	
ENST00000593106	ENSG00000131848	ZSCAN5A	
ENST00000592509	ENSG00000131848	ZSCAN5A	
ENST00000588442	ENSG00000131848	ZSCAN5A	filled
ENST00000592679	ENSG00000131848	ZSCAN5A	filled
ENST00000591078	ENSG00000131848	ZSCAN5A	
ENST00000589279	ENSG00000131848	ZSCAN5A	
ENST00000585670	ENSG00000131848	ZSCAN5A	
ENST00000587614	ENSG00000131848	ZSCAN5A	
ENST00000565275	ENSG00000103160	HSDL1	

ENST00000494188	ENSG00000138069	RAB1A	
ENST00000478109	ENSG00000163191	S100A11	
ENST00000381418	ENSG00000168453	HR	
ENST00000522759	ENSG00000168453	HR	
ENST00000518377	ENSG00000168453	HR	
ENST00000369750	ENSG00000146242	TPBG	
ENST00000445060	ENSG00000230062	ANKRD66	
ENST00000479472	ENSG00000196562	SULF2	
ENST00000360774	ENSG00000119121	TRPM6	
ENST00000483186	ENSG00000119121	TRPM6	
ENST00000361255	ENSG00000119121	TRPM6	
ENST00000430725	ENSG00000179364	PACS2	filled
ENST00000547217	ENSG00000179364	PACS2	
ENST00000464984	ENSG00000197321	SVIL	
ENST00000504690	ENSG00000169247	SH3TC2	
ENST00000510350	ENSG00000169247	SH3TC2	
ENST00000515229	ENSG00000169247	SH3TC2	
ENST00000513340	ENSG00000169247	SH3TC2	filled
ENST00000402866	ENSG00000102385	DRP2	
ENST00000395209	ENSG00000102385	DRP2	
ENST00000372916	ENSG00000102385	DRP2	
ENST00000541709	ENSG00000102385	DRP2	
ENST00000593151	ENSG00000267710	AC006116.20	
ENST00000411890	ENSG00000108813	DLX4	
ENST00000611342	ENSG00000108813	DLX4	
ENST00000491978	ENSG00000242685	HLA-DMA	
ENST00000479292	ENSG00000242685	HLA-DMA	
ENST00000404826	ENSG00000146555	SDK1	filled
ENST00000389531	ENSG00000146555	SDK1	filled
ENST00000466611	ENSG00000146555	SDK1	filled
ENST00000615806	ENSG00000146555	SDK1	filled
ENST00000625126	ENSG00000280146	RPS4Y2	
ENST00000586572	ENSG00000267001	AC006538.4	
ENST00000415707	ENSG00000096088	PGC	
ENST00000389709	ENSG00000070476	ZXDC	
ENST00000415757	ENSG00000198838	RYR3	filled
ENST00000389232	ENSG00000198838	RYR3	filled
ENST00000557931	ENSG00000198838	RYR3	
ENST00000558060	ENSG00000198838	RYR3	
ENST00000559333	ENSG00000198838	RYR3	
ENST00000560791	ENSG00000198838	RYR3	
ENST00000622037	ENSG00000198838	RYR3	filled
ENST00000411560	ENSG00000237289	CKMT1B	filled
ENST00000624754	ENSG00000279651	C3orf35	
ENST00000509448	ENSG00000243317	C7orf73	filled
ENST00000515197	ENSG00000243317	C7orf73	filled
ENST00000507606	ENSG00000243317	C7orf73	filled
ENST00000479763	ENSG00000186889	TMEM17	
ENST00000353487	ENSG00000157837	SPPL3	filled
ENST00000536996	ENSG00000157837	SPPL3	filled
ENST00000543181	ENSG00000157837	SPPL3	
ENST00000566855	ENSG00000227868	C1orf234	
ENST00000374316	ENSG00000096433	ITPR3	filled
ENST00000408965	ENSG00000221869	CEBPD	
ENST00000440542	ENSG00000226887	ERVMER34-1	
ENST00000376945	ENSG00000125285	SOX21	
ENST00000521168	ENSG00000104218	CSPP1	
ENST00000521324	ENSG00000104218	CSPP1	
ENST00000399451	ENSG00000206560	ANKRD28	filled
ENST00000525754	ENSG00000173914	RBM4B	filled

ENST00000310046	ENSG00000173914	RBM4B	filled
ENST00000531969	ENSG00000173914	RBM4B	filled
ENST00000534198	ENSG00000173914	RBM4B	
ENST00000528106	ENSG00000173914	RBM4B	
ENST00000529195	ENSG00000173914	RBM4B	
ENST00000315596	ENSG00000083642	PDS5B	filled
ENST00000482955	ENSG00000083642	PDS5B	filled
ENST00000380229	ENSG00000177047	IFNW1	
ENST00000372114	ENSG00000172315	TP53RK	filled
ENST00000557640	ENSG00000227729	RD3L	
ENST00000567051	ENSG00000140675	SLC5A2	
ENST00000564197	ENSG00000140675	SLC5A2	
ENST00000470016	ENSG00000206281	TAPBP	
ENST00000549142	ENSG00000206281	TAPBP	
ENST00000294811	ENSG00000162757	C1orf74	
ENST00000340634	ENSG00000188582	PAQR9	
ENST00000528865	ENSG00000176102	CSTF3	filled
ENST00000393909	ENSG00000115364	MRPL19	
ENST00000358788	ENSG00000115364	MRPL19	filled
ENST00000409374	ENSG00000115364	MRPL19	
ENST00000453233	ENSG00000115364	MRPL19	filled
ENST00000373517	ENSG00000186462	NAP1L2	
ENST00000560802	ENSG00000069956	MAPK6	filled
ENST00000558063	ENSG00000069956	MAPK6	filled
ENST00000560254	ENSG00000069956	MAPK6	filled
ENST00000437998	ENSG00000136267	DGKB	filled
ENST00000287934	ENSG00000157240	FZD1	filled
ENST00000377474	ENSG00000178695	KCTD12	
ENST00000624551	ENSG00000279468	AC114494.1	
ENST00000610754	ENSG00000276194	HNF1B	
ENST00000370035	ENSG00000162636	FAM102B	filled
ENST00000405454	ENSG00000162636	FAM102B	filled
ENST00000483371	ENSG00000162636	FAM102B	
ENST00000557096	ENSG00000165409	TSHR	
ENST00000260408	ENSG00000137845	ADAM10	
ENST00000402627	ENSG00000137845	ADAM10	
ENST00000561288	ENSG00000137845	ADAM10	
ENST00000559053	ENSG00000137845	ADAM10	
ENST00000558733	ENSG00000137845	ADAM10	
ENST00000439637	ENSG00000137845	ADAM10	
ENST00000560608	ENSG00000137845	ADAM10	
ENST00000283254	ENSG00000153253	SCN3A	filled
ENST00000409101	ENSG00000153253	SCN3A	filled
ENST00000453007	ENSG00000153253	SCN3A	filled
ENST00000360093	ENSG00000153253	SCN3A	filled
ENST00000506615	ENSG00000145348	TBCK	filled
ENST00000507866	ENSG00000184985	SORCS2	filled
ENST00000505529	ENSG00000184985	SORCS2	
ENST00000329016	ENSG00000184985	SORCS2	filled
ENST00000297323	ENSG00000164742	ADCY1	filled
ENST00000620650	ENSG00000277773	SMN2	filled
ENST00000477088	ENSG00000148343	FAM73B	filled
ENST00000327783	ENSG00000171189	GRIK1	filled
ENST00000389125	ENSG00000171189	GRIK1	filled
ENST00000399913	ENSG00000171189	GRIK1	filled
ENST00000399914	ENSG00000171189	GRIK1	filled
ENST00000389124	ENSG00000171189	GRIK1	filled
ENST00000399907	ENSG00000171189	GRIK1	filled
ENST00000399909	ENSG00000171189	GRIK1	filled
ENST00000472429	ENSG00000171189	GRIK1	filled

ENST00000600150	ENSG00000163714	U2SURP	
ENST00000492534	ENSG00000117280	RAB29	filled
ENST00000524607	ENSG00000198216	CACNA1E	filled
ENST00000308618	ENSG00000174279	EVX2	
ENST00000426713	ENSG00000175701	LINC00116	
ENST00000300584	ENSG00000167202	TBC1D2B	filled
ENST00000409931	ENSG00000167202	TBC1D2B	filled
ENST00000435468	ENSG00000167202	TBC1D2B	filled
ENST00000482562	ENSG00000167202	TBC1D2B	filled
ENST00000616910	ENSG00000277615	CNOT3	filled
ENST00000589222	ENSG00000141376	BCAS3	filled
ENST00000407086	ENSG00000141376	BCAS3	filled
ENST00000390652	ENSG00000141376	BCAS3	filled
ENST00000588462	ENSG00000141376	BCAS3	filled
ENST00000408905	ENSG00000141376	BCAS3	filled
ENST00000585744	ENSG00000141376	BCAS3	filled
ENST00000588874	ENSG00000141376	BCAS3	filled
ENST00000587002	ENSG00000141376	BCAS3	filled
ENST00000588008	ENSG00000141376	BCAS3	
ENST00000585812	ENSG00000141376	BCAS3	filled
ENST00000587294	ENSG00000141376	BCAS3	filled
ENST00000588569	ENSG00000141376	BCAS3	filled
ENST00000588720	ENSG00000141376	BCAS3	filled
ENST00000302270	ENSG00000172199	OR8U1	
ENST00000263233	ENSG00000102003	SYP	filled
ENST00000560549	ENSG00000138594	TMOD3	filled
ENST00000561438	ENSG00000138594	TMOD3	filled
ENST00000561136	ENSG00000138594	TMOD3	
ENST00000429234	ENSG00000248993	XXbac-BPG181M1	filled
ENST00000418023	ENSG00000232062	HLA-DQA1	filled
ENST00000444296	ENSG00000232062	HLA-DQA1	filled
ENST00000547425	ENSG00000232062	HLA-DQA1	filled
ENST00000334268	ENSG00000186439	TRDN	filled
ENST00000546248	ENSG00000186439	TRDN	filled
ENST00000513691	ENSG00000091490	SEL1L3	
ENST00000514872	ENSG00000091490	SEL1L3	
ENST00000488967	ENSG00000107796	ACTA2	filled
ENST00000259239	ENSG00000136718	IMP4	
ENST00000475074	ENSG00000136718	IMP4	
ENST00000521285	ENSG00000234284	ZNF879	
ENST00000532569	ENSG00000175538	KCNE3	
ENST00000382348	ENSG00000184221	OLIG1	
ENST00000426947	ENSG00000184221	OLIG1	
ENST00000515655	ENSG00000118777	ABCG2	filled
ENST00000370991	ENSG00000124215	CDH26	filled
ENST00000497614	ENSG00000124215	CDH26	
ENST00000244049	ENSG00000124215	CDH26	
ENST00000468915	ENSG00000158296	SLC13A3	filled
ENST00000372121	ENSG00000158296	SLC13A3	filled
ENST00000417157	ENSG00000158296	SLC13A3	filled
ENST00000471694	ENSG00000196776	CD47	
ENST00000491165	ENSG00000176542	KIAA2018	
ENST00000316407	ENSG00000176542	KIAA2018	
ENST00000538986	ENSG00000255837	TAS2R20	
ENST00000268171	ENSG00000140564	FURIN	
ENST00000559353	ENSG00000140564	FURIN	
ENST00000610579	ENSG00000140564	FURIN	
ENST00000618099	ENSG00000140564	FURIN	
ENST00000504881	ENSG00000151304	SRFBP1	filled
ENST00000442101	ENSG00000120690	ELF1	filled

ENST00000374724	ENSG00000070214	SLC44A1	filled
ENST00000560081	ENSG00000140519	RHCG	
ENST00000268122	ENSG00000140519	RHCG	
ENST00000402868	ENSG00000183955	SETD8	filled
ENST00000437519	ENSG00000183955	SETD8	filled
ENST00000537270	ENSG00000183955	SETD8	
ENST00000330479	ENSG00000183955	SETD8	filled
ENST00000575738	ENSG00000261897	OR5T3	
ENST00000487629	ENSG00000132122	SPATA6	
ENST00000374825	ENSG00000204256	BRD2	
ENST00000496118	ENSG00000204256	BRD2	
ENST00000580234	ENSG00000204256	BRD2	
ENST00000584808	ENSG00000204256	BRD2	
ENST00000581002	ENSG00000204256	BRD2	
ENST00000463639	ENSG00000204256	BRD2	
ENST00000455723	ENSG00000158427	TMSB15B	
ENST00000616936	ENSG00000151849	CENPJ	filled
ENST00000538285	ENSG00000204856	FAM216A	
ENST00000373765	ENSG00000162510	MATN1	
ENST00000469039	ENSG00000116337	AMPD2	
ENST00000617377	ENSG00000102053	ZC3H12B	filled
ENST00000338957	ENSG00000102053	ZC3H12B	
ENST00000593731	ENSG00000268173	PIK3R2	filled
ENST00000545279	ENSG00000113658	SMAD5	
ENST00000507118	ENSG00000113658	SMAD5	
ENST00000509297	ENSG00000113658	SMAD5	
ENST00000506223	ENSG00000113658	SMAD5	
ENST00000513418	ENSG00000113658	SMAD5	filled
ENST00000545620	ENSG00000113658	SMAD5	
ENST00000508208	ENSG00000164168	TMEM184C	filled
ENST00000376687	ENSG00000101945	SUV39H1	filled
ENST00000337852	ENSG00000101945	SUV39H1	filled
ENST00000370193	ENSG00000138136	LBX1	
ENST00000483294	ENSG00000198908	BHLHB9	
ENST00000361229	ENSG00000198908	BHLHB9	
ENST00000372735	ENSG00000198908	BHLHB9	
ENST00000447531	ENSG00000198908	BHLHB9	
ENST00000448867	ENSG00000198908	BHLHB9	
ENST00000457056	ENSG00000198908	BHLHB9	
ENST00000296503	ENSG00000164104	HMGB2	
ENST00000340438	ENSG00000189369	GSPT2	
ENST00000624625	ENSG00000280315	AL161784.1	
ENST00000331835	ENSG00000183291	15-Sep	filled
ENST00000370554	ENSG00000183291	15-Sep	filled
ENST00000469566	ENSG00000183291	15-Sep	filled
ENST00000401030	ENSG00000183291	15-Sep	filled
ENST00000497861	ENSG00000183291	15-Sep	filled
ENST00000467557	ENSG00000183291	15-Sep	filled
ENST00000616787	ENSG00000183291	15-Sep	filled
ENST00000611507	ENSG00000183291	15-Sep	filled
ENST00000278836	ENSG00000124920	MYRF	
ENST00000265460	ENSG00000124920	MYRF	
ENST00000305632	ENSG00000106638	TBL2	
ENST00000285238	ENSG00000108846	ABCC3	filled
ENST00000502426	ENSG00000108846	ABCC3	filled
ENST00000505699	ENSG00000108846	ABCC3	filled
ENST00000571981	ENSG00000198336	MYL4	filled
ENST00000377266	ENSG00000204740	MALRD1	filled
ENST00000454679	ENSG00000204740	MALRD1	filled
ENST00000447271	ENSG00000070159	PTPN3	

ENST00000373823	ENSG00000148180	GSN	filled
ENST00000449773	ENSG00000148180	GSN	
ENST00000432226	ENSG00000148180	GSN	
ENST00000477863	ENSG00000148180	GSN	
ENST00000475428	ENSG00000148180	GSN	
ENST00000477104	ENSG00000148180	GSN	
ENST00000373807	ENSG00000148180	GSN	filled
ENST00000545652	ENSG00000148180	GSN	
ENST00000449733	ENSG00000148180	GSN	
ENST00000436847	ENSG00000148180	GSN	
ENST00000532428	ENSG00000149089	APIP	
ENST00000504241	ENSG00000146054	TRIM7	
ENST00000579585	ENSG00000198909	MAP3K3	filled
ENST00000584573	ENSG00000198909	MAP3K3	filled
ENST00000361733	ENSG00000198909	MAP3K3	filled
ENST00000361357	ENSG00000198909	MAP3K3	filled
ENST00000577395	ENSG00000198909	MAP3K3	filled
ENST00000577597	ENSG00000198909	MAP3K3	filled
ENST00000503118	ENSG00000108829	LRRC59	
ENST00000623139	ENSG00000262826	INTS3	
ENST00000599785	ENSG00000124181	PLCG1	
ENST00000608689	ENSG00000124181	PLCG1	
ENST00000608885	ENSG00000124181	PLCG1	
ENST00000607954	ENSG00000124181	PLCG1	
ENST00000370596	ENSG00000162643	WDR63	filled
ENST00000479491	ENSG00000205835	GMNC	
ENST00000482253	ENSG00000117569	PTBP2	filled
ENST00000460706	ENSG00000117569	PTBP2	
ENST00000370198	ENSG00000117569	PTBP2	filled
ENST00000370197	ENSG00000117569	PTBP2	filled
ENST00000476419	ENSG00000117569	PTBP2	filled
ENST00000426398	ENSG00000117569	PTBP2	filled
ENST00000459735	ENSG00000117569	PTBP2	filled
ENST00000476783	ENSG00000117569	PTBP2	filled
ENST00000492905	ENSG00000117569	PTBP2	filled
ENST00000462433	ENSG00000117569	PTBP2	filled
ENST00000420103	ENSG00000163528	CHCHD4	
ENST00000360228	ENSG00000141837	CACNA1A	filled
ENST00000573710	ENSG00000141837	CACNA1A	filled
ENST00000574974	ENSG00000141837	CACNA1A	filled
ENST00000592864	ENSG00000141837	CACNA1A	filled
ENST00000614285	ENSG00000141837	CACNA1A	filled
ENST00000481983	ENSG00000187987	ZSCAN23	
ENST00000289788	ENSG00000187987	ZSCAN23	
ENST00000273283	ENSG00000055957	ITIH1	
ENST00000480409	ENSG00000055957	ITIH1	
ENST00000487686	ENSG00000055957	ITIH1	
ENST00000478667	ENSG00000055957	ITIH1	
ENST00000494603	ENSG00000055957	ITIH1	
ENST00000484844	ENSG00000055957	ITIH1	
ENST00000405128	ENSG00000055957	ITIH1	
ENST00000482836	ENSG00000055957	ITIH1	
ENST00000494705	ENSG00000055957	ITIH1	
ENST00000537050	ENSG00000055957	ITIH1	
ENST00000590414	ENSG00000189144	ZNF573	
ENST00000589632	ENSG00000189144	ZNF573	
ENST00000586155	ENSG00000189144	ZNF573	
ENST00000489148	ENSG00000189144	ZNF573	
ENST00000585724	ENSG00000189144	ZNF573	
ENST00000378445	ENSG00000189144	ZNF573	

ENST00000591516	ENSG00000189144	ZNF573	
ENST00000494605	ENSG00000189144	ZNF573	
ENST00000357309	ENSG00000189144	ZNF573	
ENST00000536220	ENSG00000189144	ZNF573	
ENST00000339503	ENSG00000189144	ZNF573	
ENST00000469481	ENSG00000101972	STAG2	filled
ENST00000479694	ENSG00000164654	MIOS	
ENST00000619253	ENSG00000275605	NDUFA3	
ENST00000549396	ENSG00000139220	PPFIA2	filled
ENST00000550584	ENSG00000139220	PPFIA2	filled
ENST00000549325	ENSG00000139220	PPFIA2	filled
ENST00000548586	ENSG00000139220	PPFIA2	filled
ENST00000552948	ENSG00000139220	PPFIA2	filled
ENST00000552020	ENSG00000139220	PPFIA2	filled
ENST00000551442	ENSG00000139220	PPFIA2	filled
ENST00000547623	ENSG00000139220	PPFIA2	filled
ENST00000547273	ENSG00000139220	PPFIA2	
ENST00000550798	ENSG00000139220	PPFIA2	filled
ENST00000522491	ENSG00000070614	NDST1	filled
ENST00000524161	ENSG00000070614	NDST1	filled
ENST00000424859	ENSG00000187416	LHFPL3	filled
ENST00000401970	ENSG00000187416	LHFPL3	filled
ENST00000608639	ENSG00000170917	NUDT6	
ENST00000520986	ENSG00000105339	DENND3	
ENST00000426055	ENSG00000003436	TFPI	filled
ENST00000409676	ENSG00000003436	TFPI	filled
ENST00000339091	ENSG00000003436	TFPI	filled
ENST00000453013	ENSG00000003436	TFPI	filled
ENST00000526381	ENSG00000136002	ARHGEF4	filled
ENST00000400419	ENSG00000139656	SMIM2	filled
ENST00000621615	ENSG00000273734	LLfos-48D6.2	filled
ENST00000593156	ENSG00000267426	RP11-552F3.12	filled
ENST00000380184	ENSG00000175518	UBQLNL	
ENST00000409324	ENSG00000221994	ZNF630	
ENST00000276054	ENSG00000221994	ZNF630	
ENST00000428686	ENSG00000221994	ZNF630	
ENST00000421903	ENSG00000221994	ZNF630	
ENST00000442455	ENSG00000221994	ZNF630	
ENST00000616492	ENSG00000221994	ZNF630	
ENST00000360426	ENSG00000124214	STAU1	
ENST00000347458	ENSG00000124214	STAU1	
ENST00000340954	ENSG00000124214	STAU1	
ENST00000371802	ENSG00000124214	STAU1	
ENST00000371792	ENSG00000124214	STAU1	
ENST00000371828	ENSG00000124214	STAU1	
ENST00000530451	ENSG00000162298	SYVN1	filled
ENST00000617904	ENSG00000054116	TRAPPC3	filled
ENST00000325404	ENSG00000181449	SOX2	
ENST00000375918	ENSG00000204444	APOM	
ENST00000375920	ENSG00000204444	APOM	
ENST00000373264	ENSG00000136807	CDK9	
ENST00000621029	ENSG00000012171	SEMA3B	
ENST00000456210	ENSG00000012171	SEMA3B	filled
ENST00000485523	ENSG00000171121	KCNMB3	
ENST00000486944	ENSG00000171121	KCNMB3	
ENST00000442398	ENSG00000168014	C2CD3	filled
ENST00000512467	ENSG00000183775	KCTD16	filled
ENST00000507359	ENSG00000183775	KCTD16	filled
ENST00000524801	ENSG00000028277	POU2F2	filled
ENST00000532176	ENSG00000028277	POU2F2	filled

ENST00000481060	ENSG00000163638	ADAMTS9	filled
ENST00000525723	ENSG00000139974	SLC38A6	filled
ENST00000491344	ENSG00000139974	SLC38A6	filled
ENST00000451406	ENSG00000139974	SLC38A6	filled
ENST00000529345	ENSG00000139974	SLC38A6	filled
ENST00000354886	ENSG00000139974	SLC38A6	filled
ENST00000610454	ENSG00000161956	SENP3	
ENST00000581010	ENSG00000161956	SENP3	
ENST00000598704	ENSG00000125648	SLC25A23	filled
ENST00000595810	ENSG00000125648	SLC25A23	filled
ENST00000598908	ENSG00000125648	SLC25A23	filled
ENST00000601322	ENSG00000125648	SLC25A23	filled
ENST00000597307	ENSG00000125648	SLC25A23	
ENST00000242109	ENSG00000122548	KIAA0087	
ENST00000258607	ENSG00000136108	CKAP2	
ENST00000490903	ENSG00000136108	CKAP2	
ENST00000490926	ENSG00000114395	CYB561D2	
ENST00000498662	ENSG00000178199	ZC3H12D	
ENST00000533186	ENSG00000140988	RPS2	filled
ENST00000412532	ENSG00000105281	SLC1A5	
ENST00000593713	ENSG00000105281	SLC1A5	
ENST00000570584	ENSG00000129221	AIPL1	filled
ENST00000378953	ENSG00000197536	C5orf56	filled
ENST00000337752	ENSG00000197536	C5orf56	filled
ENST00000407797	ENSG00000197536	C5orf56	filled
ENST00000621237	ENSG00000197536	C5orf56	filled
ENST00000445281	ENSG00000188729	OSTN	
ENST00000534428	ENSG00000167257	RNF214	filled
ENST00000563919	ENSG00000140386	SCAPER	
ENST00000554738	ENSG00000021645	NRXN3	filled
ENST00000556088	ENSG00000021645	NRXN3	filled
ENST00000553363	ENSG00000021645	NRXN3	filled
ENST00000553631	ENSG00000021645	NRXN3	filled
ENST00000554719	ENSG00000021645	NRXN3	filled
ENST00000557594	ENSG00000021645	NRXN3	filled
ENST00000281127	ENSG00000021645	NRXN3	filled
ENST00000428277	ENSG00000021645	NRXN3	filled
ENST00000555387	ENSG00000021645	NRXN3	filled
ENST00000564543	ENSG00000260272	RP11-20I23.1	filled
ENST00000569317	ENSG00000259784	ATP6C	
ENST00000379923	ENSG00000122729	ACO1	
ENST00000541043	ENSG00000122729	ACO1	
ENST00000487968	ENSG00000186716	BCR	
ENST00000492094	ENSG00000181355	OFCC1	
ENST00000487015	ENSG00000181355	OFCC1	
ENST00000460363	ENSG00000181355	OFCC1	
ENST00000492169	ENSG00000181355	OFCC1	
ENST00000469426	ENSG00000181355	OFCC1	
ENST00000469656	ENSG00000181355	OFCC1	
ENST00000486246	ENSG00000181355	OFCC1	
ENST00000472329	ENSG00000181355	OFCC1	filled
ENST00000491508	ENSG00000181355	OFCC1	
ENST00000561322	ENSG00000171914	TLN2	filled
ENST00000561311	ENSG00000171914	TLN2	filled
ENST00000245957	ENSG00000089101	CFAP61	filled
ENST00000377308	ENSG00000089101	CFAP61	filled
ENST00000469994	ENSG00000089101	CFAP61	filled
ENST00000477735	ENSG00000121879	PIK3CA	
ENST00000468036	ENSG00000121879	PIK3CA	
ENST00000559199	ENSG00000090487	SPG21	filled

ENST00000383075	ENSG00000174963	ZIC4	filled
ENST00000484399	ENSG00000174963	ZIC4	filled
ENST00000473123	ENSG00000174963	ZIC4	filled
ENST00000491672	ENSG00000174963	ZIC4	filled
ENST00000464144	ENSG00000174963	ZIC4	
ENST00000425731	ENSG00000174963	ZIC4	filled
ENST00000525172	ENSG00000174963	ZIC4	filled
ENST00000590564	ENSG00000198597	ZNF536	filled
ENST00000585628	ENSG00000198597	ZNF536	filled
ENST00000591488	ENSG00000198597	ZNF536	filled
ENST00000592773	ENSG00000198597	ZNF536	filled
ENST00000569259	ENSG00000167186	COQ7	
ENST00000586717	ENSG00000188878	FBF1	filled
ENST00000319129	ENSG00000188878	FBF1	filled
ENST00000389570	ENSG00000188878	FBF1	filled
ENST00000566249	ENSG00000065427	KARS	
ENST00000619509	ENSG00000277800	TBX22	
ENST00000563892	ENSG00000103194	USP10	
ENST00000587367	ENSG00000125743	SNRPD2	
ENST00000587579	ENSG00000125743	SNRPD2	
ENST00000528032	ENSG00000180035	ZNF48	
ENST00000612608	ENSG00000180035	ZNF48	
ENST00000622647	ENSG00000180035	ZNF48	
ENST00000623202	ENSG00000280098	AC011155.1	
ENST00000606515	ENSG00000271723	MROH7-TTC4	filled
ENST00000425300	ENSG00000271723	MROH7-TTC4	filled
ENST00000414150	ENSG00000271723	MROH7-TTC4	filled
ENST00000460155	ENSG00000271723	MROH7-TTC4	filled
ENST00000591658	ENSG00000204815	TTC25	filled
ENST00000377540	ENSG00000204815	TTC25	filled
ENST00000617460	ENSG00000204815	TTC25	filled
ENST00000366574	ENSG00000198626	RYR2	filled
ENST00000504482	ENSG00000171195	MUC7	
ENST00000304887	ENSG00000171195	MUC7	
ENST00000456088	ENSG00000171195	MUC7	
ENST00000413702	ENSG00000171195	MUC7	filled
ENST00000373347	ENSG00000116544	DLGAP3	filled
ENST00000495979	ENSG00000116544	DLGAP3	filled
ENST00000531169	ENSG00000197580	BCO2	filled
ENST00000547915	ENSG00000089022	MAPKAPK5	
ENST00000622207	ENSG00000276451	OR10A6	
ENST00000624063	ENSG00000279963	AL049872.1	
ENST00000601453	ENSG00000149311	ATM	
ENST00000624809	ENSG00000278868	AL109927.1	
ENST00000272371	ENSG00000115155	OTOF	filled
ENST00000403946	ENSG00000115155	OTOF	filled
ENST00000612788	ENSG00000273953	OR10A3	
ENST00000486583	ENSG00000116525	TRIM62	
ENST00000411780	ENSG00000166619	BLCAP	
ENST00000447625	ENSG00000166619	BLCAP	
ENST00000613961	ENSG00000166619	BLCAP	
ENST00000467603	ENSG00000166619	BLCAP	
ENST00000624812	ENSG00000279937	AC073657.1	filled
ENST00000623781	ENSG00000279824	AC104389.2	
ENST00000615600	ENSG00000277044	AL121581.1	filled
ENST00000367326	ENSG00000120278	PLEKHG1	filled
ENST00000367328	ENSG00000120278	PLEKHG1	filled
ENST00000269973	ENSG00000124459	ZNF45	
ENST00000505624	ENSG00000205838	TTC23L	filled
ENST00000569691	ENSG00000261652	C15orf65	

ENST00000570794	ENSG00000261652	C15orf65	
ENST00000520388	ENSG00000132837	DMGDH	filled
ENST00000518707	ENSG00000132837	DMGDH	filled
ENST00000623441	ENSG00000279408	AC135068.1	
ENST00000399232	ENSG00000139915	MDGA2	filled
ENST00000372290	ENSG00000178028	DMAP1	
ENST00000605528	ENSG00000270800	RPS10-NUDT3	filled
ENST00000479558	ENSG00000188452	CERKL	
ENST00000460319	ENSG00000188452	CERKL	
ENST00000497337	ENSG00000188452	CERKL	
ENST00000485330	ENSG00000163607	GTPBP8	filled
ENST00000464908	ENSG00000100297	MCM5	
ENST00000599335	ENSG00000196235	SUPT5H	
ENST00000600818	ENSG00000196235	SUPT5H	
ENST00000484068	ENSG00000173889	PHC3	
ENST00000297784	ENSG00000165091	TMC1	filled
ENST00000316939	ENSG00000179271	GADD45GIP1	
ENST00000462618	ENSG00000107099	DOCK8	
ENST00000593138	ENSG00000168517	HEXIM2	
ENST00000591070	ENSG00000168517	HEXIM2	
ENST00000546592	ENSG00000111231	GPN3	
ENST00000377602	ENSG00000148488	ST8SIA6	filled
ENST00000616297	ENSG00000273713	TAS2R9	
ENST00000620101	ENSG00000274161	AF181898.1	filled
ENST00000620226	ENSG00000274161	AF181898.1	filled
ENST00000473141	ENSG00000163634	THOC7	
ENST00000469153	ENSG00000163634	THOC7	
ENST00000487570	ENSG00000163634	THOC7	
ENST00000481897	ENSG00000127328	RAB3IP	
ENST00000560783	ENSG00000140471	LINS	
ENST00000561073	ENSG00000140471	LINS	
ENST00000559827	ENSG00000140471	LINS	
ENST00000466386	ENSG00000174827	PDZK1	
ENST00000429537	ENSG00000174827	PDZK1	
ENST00000465595	ENSG00000174827	PDZK1	
ENST00000443667	ENSG00000174827	PDZK1	
ENST00000451928	ENSG00000174827	PDZK1	
ENST00000417171	ENSG00000174827	PDZK1	
ENST00000568510	ENSG00000065457	ADAT1	
ENST00000564657	ENSG00000065457	ADAT1	
ENST00000461655	ENSG00000162407	PPAP2B	filled
ENST00000481743	ENSG00000107485	GATA3	
ENST00000379328	ENSG00000107485	GATA3	filled
ENST00000346208	ENSG00000107485	GATA3	
ENST00000461472	ENSG00000107485	GATA3	filled
ENST00000469019	ENSG00000136720	HS6ST1	filled
ENST00000373173	ENSG00000155008	APOOL	
ENST00000578424	ENSG00000169710	FASN	
ENST00000558014	ENSG00000137872	SEMA6D	filled
ENST00000559184	ENSG00000137872	SEMA6D	filled
ENST00000520821	ENSG00000243978	RGAG1	filled
ENST00000465301	ENSG00000243978	RGAG1	
ENST00000540313	ENSG00000243978	RGAG1	
ENST00000533757	ENSG00000135365	PHF21A	
ENST00000525438	ENSG00000135365	PHF21A	
ENST00000527782	ENSG00000135365	PHF21A	
ENST00000532010	ENSG00000135365	PHF21A	
ENST00000528893	ENSG00000135365	PHF21A	
ENST00000369768	ENSG00000125522	NPBWR2	
ENST00000424550	ENSG00000170054	SERPINA9	filled

ENST00000337425	ENSG00000170054	SERPINA9	filled
ENST00000538527	ENSG00000170054	SERPINA9	filled
ENST00000546329	ENSG00000170054	SERPINA9	filled
ENST00000539349	ENSG00000170054	SERPINA9	filled
ENST00000314259	ENSG00000181903	OR4C6	
ENST00000334504	ENSG00000197565	COL4A6	filled
ENST00000372216	ENSG00000197565	COL4A6	filled
ENST00000468338	ENSG00000197565	COL4A6	filled
ENST00000538570	ENSG00000197565	COL4A6	filled
ENST00000394872	ENSG00000197565	COL4A6	filled
ENST00000621266	ENSG00000197565	COL4A6	filled
ENST00000545689	ENSG00000197565	COL4A6	filled
ENST00000481381	ENSG00000068903	SIRT2	
ENST00000254803	ENSG00000132467	UTP3	
ENST00000558506	ENSG00000100664	EIF5	
ENST00000561406	ENSG00000100664	EIF5	
ENST00000308158	ENSG00000175309	PHYKPL	filled
ENST00000481436	ENSG00000175309	PHYKPL	filled
ENST00000474052	ENSG00000175309	PHYKPL	filled
ENST00000393488	ENSG00000175309	PHYKPL	filled
ENST00000493197	ENSG00000175309	PHYKPL	filled
ENST00000510991	ENSG00000175309	PHYKPL	filled
ENST00000377291	ENSG00000113749	HRH2	filled
ENST00000471600	ENSG00000076864	RAP1GAP	filled
ENST00000495204	ENSG00000076864	RAP1GAP	filled
ENST00000374757	ENSG00000076864	RAP1GAP	filled
ENST00000290101	ENSG00000076864	RAP1GAP	filled
ENST00000616581	ENSG00000275600	PIGW	
ENST00000375155	ENSG00000040487	PQLC2	filled
ENST00000375153	ENSG00000040487	PQLC2	filled
ENST00000469076	ENSG00000040487	PQLC2	filled
ENST00000400548	ENSG00000040487	PQLC2	filled
ENST00000437504	ENSG00000132623	ANKEF1	filled
ENST00000475662	ENSG00000115163	CENPA	filled
ENST00000241436	ENSG00000122008	POLK	filled
ENST00000503479	ENSG00000122008	POLK	filled
ENST00000505774	ENSG00000122008	POLK	filled
ENST00000504315	ENSG00000197757	HOXC6	filled
ENST00000262032	ENSG00000123411	IKZF4	
ENST00000548601	ENSG00000123411	IKZF4	
ENST00000550860	ENSG00000123411	IKZF4	
ENST00000552689	ENSG00000123411	IKZF4	
ENST00000549519	ENSG00000123411	IKZF4	
ENST00000478425	ENSG00000109689	STIM2	
ENST00000532354	ENSG00000170955	PRKCDBP	
ENST00000303927	ENSG00000170955	PRKCDBP	
ENST00000524852	ENSG00000170955	PRKCDBP	
ENST00000530979	ENSG00000170955	PRKCDBP	
ENST00000012049	ENSG00000011478	QPCTL	filled
ENST00000366382	ENSG00000011478	QPCTL	filled
ENST00000584040	ENSG00000174448	STARD6	
ENST00000625158	ENSG00000279576	MALAT1	
ENST00000519356	ENSG00000129696	TTI2	
ENST00000575734	ENSG00000166292	TMEM100	
ENST00000575806	ENSG00000166292	TMEM100	
ENST00000624710	ENSG00000279232	DKFZP434P228	
ENST00000480932	ENSG00000215522	DDR1	
ENST00000273610	ENSG00000145040	UCN2	
ENST00000453513	ENSG00000224319	OR2H2	
ENST00000262605	ENSG00000124120	TTPAL	filled

ENST00000372904	ENSG00000124120	TTPAL	filled
ENST00000461134	ENSG00000124120	TTPAL	
ENST00000370156	ENSG00000117620	SLC35A3	
ENST00000451080	ENSG00000138395	CDK15	filled
ENST00000493754	ENSG00000138395	CDK15	
ENST00000616467	ENSG00000175470	PPP2R2D	filled
ENST00000422256	ENSG00000175470	PPP2R2D	filled
ENST00000468884	ENSG00000177683	THAP5	
ENST00000415914	ENSG00000177683	THAP5	
ENST00000294072	ENSG00000162144	CYB561A3	filled
ENST00000536452	ENSG00000162144	CYB561A3	
ENST00000540755	ENSG00000162144	CYB561A3	filled
ENST00000539128	ENSG00000162144	CYB561A3	filled
ENST00000538263	ENSG00000162144	CYB561A3	filled
ENST00000542411	ENSG00000162144	CYB561A3	
ENST00000537680	ENSG00000162144	CYB561A3	
ENST00000426130	ENSG00000162144	CYB561A3	filled
ENST00000442633	ENSG00000273331	TM4SF19-TCTEX1D2	
ENST00000625138	ENSG00000117859	OSBPL9	
ENST00000417572	ENSG00000093183	SEC22C	filled
ENST00000441821	ENSG00000185666	SYN3	filled
ENST00000412575	ENSG00000185666	SYN3	
ENST00000557497	ENSG00000013523	ANGEL1	filled
ENST00000398904	ENSG00000146122	DAAM2	filled
ENST00000538976	ENSG00000146122	DAAM2	filled
ENST00000445218	ENSG00000225859	DDX39B	filled
ENST00000557114	ENSG00000100814	CCNB1IP1	filled
ENST00000231061	ENSG00000113140	SPARC	
ENST00000538026	ENSG00000113140	SPARC	
ENST00000521569	ENSG00000113140	SPARC	
ENST00000539687	ENSG00000113140	SPARC	
ENST00000521327	ENSG00000113140	SPARC	
ENST00000522348	ENSG00000113140	SPARC	
ENST00000620972	ENSG00000113140	SPARC	
ENST00000344606	ENSG00000121895	TMEM156	
ENST00000372489	ENSG00000121895	TMEM156	
ENST00000495029	ENSG00000121895	TMEM156	
ENST00000391857	ENSG00000142541	RPL13A	
ENST00000624069	ENSG00000142541	RPL13A	
ENST00000472481	ENSG00000142541	RPL13A	
ENST00000484279	ENSG00000142541	RPL13A	
ENST00000479992	ENSG00000142541	RPL13A	
ENST00000477613	ENSG00000142541	RPL13A	
ENST00000621674	ENSG00000142541	RPL13A	
ENST00000349938	ENSG00000135931	ARMC9	filled
ENST00000469789	ENSG00000135931	ARMC9	filled
ENST00000444285	ENSG00000135931	ARMC9	
ENST00000436339	ENSG00000135931	ARMC9	filled
ENST00000467698	ENSG00000135931	ARMC9	filled
ENST00000424740	ENSG00000135931	ARMC9	filled
ENST00000612844	ENSG00000110203	FOLR3	filled
ENST00000611028	ENSG00000110203	FOLR3	filled
ENST00000546166	ENSG00000110203	FOLR3	filled
ENST00000521478	ENSG00000106006	HOXA6	
ENST00000435520	ENSG00000197586	ENTPD6	
ENST00000490320	ENSG00000180044	C3orf80	
ENST00000610477	ENSG00000180044	C3orf80	
ENST00000326474	ENSG00000180044	C3orf80	
ENST00000491526	ENSG00000135540	NHSL1	filled
ENST00000468095	ENSG00000135540	NHSL1	filled

ENST00000454243	ENSG00000168490	PHYHIP	
ENST00000307961	ENSG00000174444	RPL4	
ENST00000577647	ENSG00000264813	ACE	filled
ENST00000421982	ENSG00000264813	ACE	filled
ENST00000560898	ENSG00000137804	NUSAP1	
ENST00000389908	ENSG00000102445	KIAA0226L	filled
ENST00000378787	ENSG00000102445	KIAA0226L	filled
ENST00000439642	ENSG00000102445	KIAA0226L	filled
ENST00000378797	ENSG00000102445	KIAA0226L	filled
ENST00000429979	ENSG00000102445	KIAA0226L	filled
ENST00000378781	ENSG00000102445	KIAA0226L	filled
ENST00000543469	ENSG00000213186	TRIM59	
ENST00000539036	ENSG00000008394	MGST1	filled
ENST00000542256	ENSG00000008394	MGST1	
ENST00000433552	ENSG00000224952	PBX2	filled
ENST00000596436	ENSG00000130307	USHBP1	filled
ENST00000403633	ENSG00000106245	BUD31	filled
ENST00000222969	ENSG00000106245	BUD31	filled
ENST00000376497	ENSG00000011021	CLCN6	filled
ENST00000588873	ENSG00000267360	CTC-454I21.3	filled
ENST00000426401	ENSG00000227567	APOM	
ENST00000439902	ENSG00000227567	APOM	
ENST00000379108	ENSG00000180332	KCTD4	
ENST00000612192	ENSG00000273889	FAM83H	
ENST00000395032	ENSG00000149516	MS4A3	filled
ENST00000528298	ENSG00000149516	MS4A3	
ENST00000278865	ENSG00000149516	MS4A3	filled
ENST00000526199	ENSG00000149516	MS4A3	
ENST00000528952	ENSG00000149516	MS4A3	
ENST00000358152	ENSG00000149516	MS4A3	filled
ENST00000366707	ENSG00000154358	OBSCN	filled
ENST00000570156	ENSG00000154358	OBSCN	filled
ENST00000422127	ENSG00000154358	OBSCN	filled
ENST00000394511	ENSG00000174607	UGT8	filled
ENST00000520739	ENSG00000104660	LEPROTL1	filled
ENST00000423645	ENSG00000125999	BPIFB1	
ENST00000477335	ENSG00000102144	PGK1	filled
ENST00000491291	ENSG00000102144	PGK1	filled
ENST00000228741	ENSG00000111145	ELK3	filled
ENST00000326756	ENSG00000178163	ZNF518B	
ENST00000507515	ENSG00000178163	ZNF518B	
ENST00000515072	ENSG00000178163	ZNF518B	
ENST00000555159	ENSG00000025423	HSD17B6	filled
ENST00000555805	ENSG00000025423	HSD17B6	filled
ENST00000554643	ENSG00000025423	HSD17B6	filled
ENST00000554150	ENSG00000025423	HSD17B6	filled
ENST00000554155	ENSG00000025423	HSD17B6	filled
ENST00000542705	ENSG00000177575	CD163	
ENST00000379445	ENSG00000205279	CTXN3	filled
ENST00000295896	ENSG00000163632	C3orf49	
ENST00000482662	ENSG00000115486	GGCX	filled
ENST00000415090	ENSG00000241674	HLA-DMB	filled
ENST00000419996	ENSG00000206409	APOM	
ENST00000400157	ENSG00000206409	APOM	
ENST00000446620	ENSG00000236398	TAS2R39	
ENST00000567309	ENSG00000261052	SULT1A3	filled
ENST00000563322	ENSG00000261052	SULT1A3	filled
ENST00000569485	ENSG00000261052	SULT1A3	filled
ENST00000561549	ENSG00000261052	SULT1A3	filled
ENST00000561533	ENSG00000261052	SULT1A3	filled

ENST00000623310	ENSG00000101901	ALG13	
ENST00000605067	ENSG00000145990	GFOD1	
ENST00000379278	ENSG00000145990	GFOD1	
ENST00000612338	ENSG00000145990	GFOD1	filled
ENST00000239878	ENSG00000120686	UFM1	
ENST00000619935	ENSG00000277354	CRIM1	
ENST00000570574	ENSG00000262315	OR8U9	
ENST00000513567	ENSG00000163288	GABRB1	filled
ENST00000474953	ENSG00000054267	ARID4B	filled
ENST00000450494	ENSG00000139514	SLC7A1	filled
ENST00000462832	ENSG00000163806	SPDYA	
ENST00000547605	ENSG00000078328	RBFOX1	filled
ENST00000550418	ENSG00000078328	RBFOX1	filled
ENST00000547372	ENSG00000078328	RBFOX1	filled
ENST00000548749	ENSG00000078328	RBFOX1	filled
ENST00000422070	ENSG00000078328	RBFOX1	filled
ENST00000535565	ENSG00000078328	RBFOX1	filled
ENST00000547427	ENSG00000078328	RBFOX1	filled
ENST00000551752	ENSG00000078328	RBFOX1	filled
ENST00000512188	ENSG00000164106	SCRG1	
ENST00000545719	ENSG00000135048	TMEM2	filled
ENST00000361124	ENSG00000198885	ITPRIPL1	
ENST00000519760	ENSG00000170312	CDK1	
ENST00000615108	ENSG00000276416	AL136338.1	filled
ENST00000502461	ENSG00000151725	CENPU	
ENST00000490264	ENSG00000168301	KCTD6	
ENST00000479179	ENSG00000168301	KCTD6	
ENST00000531214	ENSG00000177542	SLC25A22	
ENST00000529351	ENSG00000177542	SLC25A22	
ENST00000559847	ENSG00000181827	RFX7	filled
ENST00000559447	ENSG00000181827	RFX7	filled
ENST00000560792	ENSG00000181827	RFX7	filled
ENST00000298894	ENSG00000165943	MOAP1	
ENST00000556883	ENSG00000165943	MOAP1	
ENST00000375915	ENSG00000204442	FAM155A	filled
ENST00000586178	ENSG00000011258	MBTD1	filled
ENST00000405860	ENSG00000011258	MBTD1	filled
ENST00000593259	ENSG00000011258	MBTD1	filled
ENST00000596188	ENSG00000179542	SLITRK4	filled
ENST00000495160	ENSG00000163666	HESX1	filled
ENST00000554140	ENSG00000183092	BEGAIN	filled
ENST00000529024	ENSG00000162139	NEU3	filled
ENST00000244221	ENSG00000124374	PAIP2B	
ENST00000399398	ENSG00000189051	RNF222	
ENST00000344001	ENSG00000189051	RNF222	
ENST00000460386	ENSG00000130150	MOSPD2	filled
ENST00000517562	ENSG00000253250	C8orf88	filled
ENST00000409327	ENSG00000186684	CYP27C1	
ENST00000548759	ENSG00000135144	DTX1	
ENST00000561216	ENSG00000156206	C15orf26	
ENST00000311104	ENSG00000173250	GPR151	
ENST00000614620	ENSG00000082515	MRPL22	
ENST00000579242	ENSG00000180901	KCTD2	
ENST00000584767	ENSG00000180901	KCTD2	
ENST00000508795	ENSG00000172262	ZNF131	
ENST00000507393	ENSG00000172262	ZNF131	filled
ENST00000513741	ENSG00000138738	PRDM5	
ENST00000392321	ENSG00000196458	ZNF605	filled
ENST00000331711	ENSG00000196458	ZNF605	filled
ENST00000412621	ENSG00000196458	ZNF605	filled

ENST00000513209	ENSG00000273049	RP11-834C11.12	filled
ENST00000501038	ENSG00000004399	PLXND1	
ENST00000504689	ENSG00000004399	PLXND1	
ENST00000504524	ENSG00000004399	PLXND1	
ENST00000506979	ENSG00000004399	PLXND1	
ENST00000512807	ENSG00000004399	PLXND1	
ENST00000504979	ENSG00000004399	PLXND1	
ENST00000505237	ENSG00000004399	PLXND1	filled
ENST00000435164	ENSG00000090372	STRN4	
ENST00000593979	ENSG00000090372	STRN4	
ENST00000596012	ENSG00000090372	STRN4	
ENST00000473954	ENSG00000204569	PPP1R10	
ENST00000589410	ENSG00000176136	MC5R	
ENST00000271850	ENSG00000143549	TPM3	
ENST00000515609	ENSG00000143549	TPM3	
ENST00000540590	ENSG00000048540	LMO3	
ENST00000332914	ENSG00000048540	LMO3	
ENST00000537568	ENSG00000048540	LMO3	
ENST00000338257	ENSG00000142347	MYO1F	filled
ENST00000596245	ENSG00000142347	MYO1F	
ENST00000598005	ENSG00000142347	MYO1F	filled
ENST00000598797	ENSG00000142347	MYO1F	
ENST00000597222	ENSG00000142347	MYO1F	filled
ENST00000601502	ENSG00000142347	MYO1F	
ENST00000600825	ENSG00000142347	MYO1F	
ENST00000613525	ENSG00000142347	MYO1F	filled
ENST00000262626	ENSG00000105707	HPN	filled
ENST00000600390	ENSG00000105707	HPN	filled
ENST00000597419	ENSG00000105707	HPN	filled
ENST00000361050	ENSG00000197629	MPEG1	
ENST00000535191	ENSG00000114904	NEK4	filled
ENST00000417712	ENSG00000100221	JOSD1	
ENST00000456626	ENSG00000100221	JOSD1	
ENST00000462610	ENSG00000100221	JOSD1	
ENST00000493939	ENSG00000100221	JOSD1	
ENST00000591163	ENSG00000267796	LIN37	
ENST00000390015	ENSG00000171298	GAA	filled
ENST00000302262	ENSG00000171298	GAA	filled
ENST00000394709	ENSG00000126803	HSPA2	
ENST00000247207	ENSG00000126803	HSPA2	
ENST00000488517	ENSG00000235291	PPP1R10	
ENST00000330949	ENSG00000182968	SOX1	
ENST00000296591	ENSG00000164176	EDIL3	filled
ENST00000406775	ENSG00000158321	AUTS2	filled
ENST00000342771	ENSG00000158321	AUTS2	filled
ENST00000403018	ENSG00000158321	AUTS2	filled
ENST00000476695	ENSG00000158321	AUTS2	filled
ENST00000475660	ENSG00000158321	AUTS2	
ENST00000615871	ENSG00000158321	AUTS2	filled
ENST00000611706	ENSG00000158321	AUTS2	filled
ENST00000262891	ENSG00000007047	MARK4	filled
ENST00000300843	ENSG00000007047	MARK4	filled
ENST00000592762	ENSG00000007047	MARK4	
ENST00000588533	ENSG00000007047	MARK4	
ENST00000588621	ENSG00000007047	MARK4	
ENST00000592207	ENSG00000007047	MARK4	
ENST00000590897	ENSG00000007047	MARK4	
ENST00000622871	ENSG00000007047	MARK4	filled
ENST00000620044	ENSG00000007047	MARK4	filled
ENST00000359373	ENSG00000186635	ARAP1	filled

ENST00000492749	ENSG00000239974	EGFL8	filled
ENST00000508322	ENSG00000007062	PROM1	
ENST00000508940	ENSG00000007062	PROM1	
ENST00000514967	ENSG00000007062	PROM1	
ENST00000599174	ENSG00000267909	CCDC177	
ENST00000489665	ENSG00000174945	AMZ1	filled
ENST00000238497	ENSG00000119541	VPS4B	
ENST00000588059	ENSG00000119541	VPS4B	
ENST00000591519	ENSG00000119541	VPS4B	
ENST00000564071	ENSG00000186118	TEX38	
ENST00000620457	ENSG00000276495	APBA2	filled
ENST00000612449	ENSG00000276495	APBA2	filled
ENST00000549920	ENSG00000063177	RPL18	
ENST00000550973	ENSG00000063177	RPL18	
ENST00000586501	ENSG00000150477	KIAA1328	filled
ENST00000405709	ENSG00000184903	IMMP2L	filled
ENST00000331762	ENSG00000184903	IMMP2L	filled
ENST00000489381	ENSG00000184903	IMMP2L	filled
ENST00000452895	ENSG00000184903	IMMP2L	filled
ENST00000447215	ENSG00000184903	IMMP2L	filled
ENST00000492938	ENSG00000184903	IMMP2L	filled
ENST00000452753	ENSG00000184903	IMMP2L	filled
ENST00000437687	ENSG00000184903	IMMP2L	filled
ENST00000589214	ENSG00000012061	ERCC1	
ENST00000525734	ENSG00000110274	CEP164	filled
ENST00000533153	ENSG00000110274	CEP164	filled
ENST00000360759	ENSG00000170683	OR10A3	
ENST00000378614	ENSG00000086475	SEPHS1	
ENST00000562370	ENSG00000103326	CAPN15	
ENST00000568988	ENSG00000103326	CAPN15	
ENST00000550502	ENSG00000185432	METTL7A	
ENST00000547104	ENSG00000185432	METTL7A	
ENST00000550097	ENSG00000185432	METTL7A	
ENST00000608781	ENSG00000163006	CCDC138	filled
ENST00000609740	ENSG00000163006	CCDC138	
ENST00000534100	ENSG00000254445	HSPB2-C11orf52	filled
ENST00000436711	ENSG00000158863	FAM160B2	
ENST00000558530	ENSG00000259399	TGIF2-C20orf24	filled
ENST00000301618	ENSG00000167889	MGAT5B	filled
ENST00000569840	ENSG00000167889	MGAT5B	filled
ENST00000565675	ENSG00000167889	MGAT5B	
ENST00000428789	ENSG00000167889	MGAT5B	filled
ENST00000565043	ENSG00000167889	MGAT5B	filled
ENST00000481801	ENSG00000152127	MGAT5	
ENST00000468758	ENSG00000152127	MGAT5	
ENST00000409645	ENSG00000152127	MGAT5	filled
ENST00000488365	ENSG00000152127	MGAT5	
ENST00000612327	ENSG00000276220	NDUFA3	
ENST00000377942	ENSG00000136099	PCDH8	
ENST00000518711	ENSG00000164808	SPIDR	
ENST00000381873	ENSG00000101298	SNPH	filled
ENST00000381867	ENSG00000101298	SNPH	filled
ENST00000614659	ENSG00000101298	SNPH	filled
ENST00000382461	ENSG00000171044	XKR6	filled
ENST00000416569	ENSG00000171044	XKR6	filled
ENST00000529336	ENSG00000171044	XKR6	
ENST00000577138	ENSG00000262302	RP1-4G17.5	
ENST00000395733	ENSG00000100253	MIOX	filled
ENST00000216075	ENSG00000100253	MIOX	filled
ENST00000395732	ENSG00000100253	MIOX	filled

ENST00000307522	ENSG00000169515	CCDC8	
ENST00000614237	ENSG00000277957	SENP3-EIF4A1	
ENST00000472720	ENSG00000144229	THSD7B	filled
ENST00000256509	ENSG00000134121	CHL1	filled
ENST00000453040	ENSG00000134121	CHL1	filled
ENST00000435603	ENSG00000134121	CHL1	filled
ENST00000486881	ENSG00000134121	CHL1	
ENST00000449294	ENSG00000134121	CHL1	filled
ENST00000533475	ENSG00000109846	CRYAB	
ENST00000531198	ENSG00000109846	CRYAB	
ENST00000527899	ENSG00000109846	CRYAB	
ENST00000529647	ENSG00000109846	CRYAB	
ENST00000528628	ENSG00000109846	CRYAB	
ENST00000533879	ENSG00000109846	CRYAB	
ENST00000616970	ENSG00000109846	CRYAB	
ENST00000485616	ENSG00000106686	SPATA6L	filled
ENST00000379706	ENSG00000145777	TSLP	
ENST00000369683	ENSG00000108039	XPNPEP1	filled
ENST00000502935	ENSG00000108039	XPNPEP1	filled
ENST00000509646	ENSG00000108039	XPNPEP1	filled
ENST00000460523	ENSG00000108039	XPNPEP1	filled
ENST00000403138	ENSG00000108039	XPNPEP1	filled
ENST00000430337	ENSG00000108039	XPNPEP1	filled
ENST00000443078	ENSG00000108039	XPNPEP1	filled
ENST00000506777	ENSG00000108039	XPNPEP1	filled
ENST00000490740	ENSG00000108039	XPNPEP1	filled
ENST00000451592	ENSG00000108039	XPNPEP1	filled
ENST00000508275	ENSG00000108039	XPNPEP1	filled
ENST00000322238	ENSG00000108039	XPNPEP1	filled
ENST00000523601	ENSG00000104375	STK3	filled
ENST00000523567	ENSG00000104375	STK3	
ENST00000519420	ENSG00000104375	STK3	
ENST00000368710	ENSG00000196754	S100A2	
ENST00000590148	ENSG00000231256	C17orf105	
ENST00000399075	ENSG00000164096	C4orf3	
ENST00000405659	ENSG00000187860	CCDC157	filled
ENST00000338306	ENSG00000187860	CCDC157	filled
ENST00000589413	ENSG00000188283	ZNF383	filled
ENST00000619966	ENSG00000276766	NDUFA3	
ENST00000553654	ENSG00000166881	TMEM194A	filled
ENST00000248564	ENSG00000127920	GNG11	
ENST00000409514	ENSG00000135930	EIF4E2	
ENST00000409098	ENSG00000135930	EIF4E2	
ENST00000409394	ENSG00000135930	EIF4E2	
ENST00000478878	ENSG00000135930	EIF4E2	
ENST00000459714	ENSG00000135930	EIF4E2	
ENST00000444113	ENSG00000135930	EIF4E2	
ENST00000376131	ENSG00000102466	FGF14	filled
ENST00000418923	ENSG00000102466	FGF14	
ENST00000606589	ENSG00000272104	XXcos-LUCA11.5	
ENST00000476279	ENSG00000090266	NDUFB2	
ENST00000472523	ENSG00000152977	ZIC1	filled
ENST00000474034	ENSG00000152977	ZIC1	filled
ENST00000481840	ENSG00000152977	ZIC1	
ENST00000569360	ENSG00000261459	ZNF747	
ENST00000367696	ENSG00000135870	RC3H1	filled
ENST00000402452	ENSG00000244588	RAD21L1	filled
ENST00000524144	ENSG00000134013	LOXL2	
ENST00000520871	ENSG00000134013	LOXL2	
ENST00000503326	ENSG00000184432	COPB2	

ENST00000356455	ENSG00000175063	UBE2C	
ENST00000335046	ENSG00000175063	UBE2C	
ENST00000243893	ENSG00000175063	UBE2C	
ENST00000352551	ENSG00000175063	UBE2C	
ENST00000496085	ENSG00000175063	UBE2C	
ENST00000405520	ENSG00000175063	UBE2C	
ENST00000617055	ENSG00000175063	UBE2C	
ENST00000566850	ENSG00000103544	C16orf62	
ENST00000615008	ENSG00000089063	TMEM230	filled
ENST00000612323	ENSG00000089063	TMEM230	filled
ENST00000594268	ENSG00000104901	DKKL1	
ENST00000554677	ENSG00000100714	MTHFD1	filled
ENST00000610551	ENSG00000273780	ARHGAP23	filled
ENST00000462348	ENSG00000146223	RPL7L1	
ENST00000459829	ENSG00000146223	RPL7L1	
ENST00000528559	ENSG00000110697	PITPNM1	filled
ENST00000037869	ENSG00000035141	FAM136A	
ENST00000457458	ENSG00000102225	CDK16	
ENST00000517426	ENSG00000102225	CDK16	
ENST00000518391	ENSG00000102225	CDK16	
ENST00000520141	ENSG00000102225	CDK16	
ENST00000462827	ENSG00000102225	CDK16	
ENST00000276052	ENSG00000102225	CDK16	
ENST00000473854	ENSG00000145075	CCDC39	
ENST00000489868	ENSG00000145075	CCDC39	
ENST00000476379	ENSG00000145075	CCDC39	filled
ENST00000457914	ENSG00000114302	PRKAR2A	
ENST00000296446	ENSG00000114302	PRKAR2A	filled
ENST00000537873	ENSG00000139197	PEX5	
ENST00000351960	ENSG00000137497	NUMA1	
ENST00000393695	ENSG00000137497	NUMA1	
ENST00000537217	ENSG00000137497	NUMA1	
ENST00000543009	ENSG00000137497	NUMA1	
ENST00000537930	ENSG00000137497	NUMA1	
ENST00000535947	ENSG00000137497	NUMA1	
ENST00000613205	ENSG00000137497	NUMA1	
ENST00000517981	ENSG00000147613	PSKH2	filled
ENST00000523010	ENSG00000147613	PSKH2	
ENST00000572893	ENSG00000169992	NLGN2	
ENST00000592437	ENSG00000263002	ZNF234	filled
ENST00000423994	ENSG00000007402	CACNA2D2	filled
ENST00000429770	ENSG00000007402	CACNA2D2	filled
ENST00000266039	ENSG00000007402	CACNA2D2	filled
ENST00000360963	ENSG00000007402	CACNA2D2	filled
ENST00000483620	ENSG00000007402	CACNA2D2	
ENST00000424201	ENSG00000007402	CACNA2D2	filled
ENST00000479441	ENSG00000007402	CACNA2D2	filled
ENST00000327259	ENSG00000179292	TMEM151A	
ENST00000586012	ENSG00000267157	CTB-54O9.9	
ENST00000541638	ENSG00000067221	STOML1	
ENST00000561656	ENSG00000067221	STOML1	
ENST00000619177	ENSG00000110944	IL23A	
ENST00000460974	ENSG00000035115	SH3YL1	
ENST00000376759	ENSG00000102317	RBM3	
ENST00000483183	ENSG00000240592	EGFL8	filled
ENST00000563303	ENSG00000159708	LRRC36	filled
ENST00000570075	ENSG00000159708	LRRC36	filled
ENST00000569499	ENSG00000159708	LRRC36	filled
ENST00000329956	ENSG00000159708	LRRC36	filled
ENST00000561948	ENSG00000159708	LRRC36	filled

ENST00000569228	ENSG00000159708	LRRC36	filled
ENST00000565019	ENSG00000159708	LRRC36	filled
ENST00000568804	ENSG00000159708	LRRC36	filled
ENST00000566558	ENSG00000159708	LRRC36	
ENST00000568010	ENSG00000159708	LRRC36	filled
ENST00000569552	ENSG00000159708	LRRC36	filled
ENST00000567723	ENSG00000159708	LRRC36	filled
ENST00000563189	ENSG00000159708	LRRC36	filled
ENST00000567823	ENSG00000159708	LRRC36	filled
ENST00000561821	ENSG00000159708	LRRC36	filled
ENST00000435835	ENSG00000159708	LRRC36	filled
ENST00000567195	ENSG00000140365	COMMD4	
ENST00000564815	ENSG00000140365	COMMD4	
ENST00000562610	ENSG00000140365	COMMD4	
ENST00000338995	ENSG00000140365	COMMD4	
ENST00000561662	ENSG00000140365	COMMD4	
ENST00000568034	ENSG00000140365	COMMD4	
ENST00000567377	ENSG00000140365	COMMD4	
ENST00000562789	ENSG00000140365	COMMD4	
ENST00000568301	ENSG00000140365	COMMD4	
ENST00000282020	ENSG00000152208	GRID2	filled
ENST00000505687	ENSG00000152208	GRID2	filled
ENST00000510992	ENSG00000152208	GRID2	filled
ENST00000515744	ENSG00000152208	GRID2	filled
ENST00000506929	ENSG00000158985	CDC42SE2	
ENST00000502639	ENSG00000158985	CDC42SE2	
ENST00000511432	ENSG00000158985	CDC42SE2	filled
ENST00000502582	ENSG00000158985	CDC42SE2	
ENST00000620529	ENSG00000185619	PCGF3	
ENST00000246895	ENSG00000126549	STATH	filled
ENST00000615994	ENSG00000126549	STATH	filled
ENST00000370994	ENSG00000142864	SERBP1	
ENST00000040584	ENSG00000037965	HOXC8	
ENST00000565592	ENSG00000167964	RAB26	filled
ENST00000417257	ENSG00000171877	FRMD5	filled
ENST00000402883	ENSG00000171877	FRMD5	filled
ENST00000484674	ENSG00000171877	FRMD5	filled
ENST00000458630	ENSG00000171877	FRMD5	filled
ENST00000421674	ENSG00000171877	FRMD5	filled
ENST00000451277	ENSG00000171877	FRMD5	filled
ENST00000490337	ENSG00000169282	KCNAB1	filled
ENST00000389636	ENSG00000169282	KCNAB1	filled
ENST00000471742	ENSG00000169282	KCNAB1	filled
ENST00000591946	ENSG00000130175	PRKCSH	
ENST00000588269	ENSG00000130175	PRKCSH	
ENST00000530722	ENSG00000158816	VWA5B1	
ENST00000534075	ENSG00000158816	VWA5B1	
ENST00000622766	ENSG00000162601	MYSM1	filled
ENST00000302053	ENSG00000171560	FGA	filled
ENST00000549855	ENSG00000196531	NACA	filled
ENST00000266000	ENSG00000204209	DAXX	
ENST00000374542	ENSG00000204209	DAXX	
ENST00000468536	ENSG00000204209	DAXX	
ENST00000477162	ENSG00000204209	DAXX	
ENST00000477370	ENSG00000204209	DAXX	
ENST00000446403	ENSG00000204209	DAXX	
ENST00000453407	ENSG00000204209	DAXX	filled
ENST00000498030	ENSG00000204209	DAXX	
ENST00000446511	ENSG00000204209	DAXX	
ENST00000494082	ENSG00000204209	DAXX	

ENST00000414083	ENSG00000204209	DAXX	
ENST00000620164	ENSG00000204209	DAXX	
ENST00000405402	ENSG00000131149	GSE1	filled
ENST00000411612	ENSG00000131149	GSE1	filled
ENST00000253458	ENSG00000131149	GSE1	filled
ENST00000223129	ENSG00000106399	RPA3	
ENST00000463632	ENSG00000106399	RPA3	
ENST00000589258	ENSG00000267140	RP11-322E11.6	filled
ENST00000411503	ENSG00000173641	HSPB7	
ENST00000442459	ENSG00000173641	HSPB7	
ENST00000311890	ENSG00000173641	HSPB7	
ENST00000375718	ENSG00000173641	HSPB7	
ENST00000541464	ENSG00000103534	TMC5	filled
ENST00000381414	ENSG00000103534	TMC5	filled
ENST00000542583	ENSG00000103534	TMC5	filled
ENST00000396229	ENSG00000103534	TMC5	filled
ENST00000616359	ENSG00000274616	CNOT3	filled
ENST00000511449	ENSG00000173542	MOB1B	filled
ENST00000548312	ENSG00000122986	HVCN1	filled
ENST00000489738	ENSG00000135241	PNPLA8	filled
ENST00000380252	ENSG00000196565	HBG2	filled
ENST00000535042	ENSG00000134825	TMEM258	
ENST00000496961	ENSG00000174151	CYB561D1	
ENST00000310611	ENSG00000174151	CYB561D1	
ENST00000420578	ENSG00000174151	CYB561D1	
ENST00000528785	ENSG00000174151	CYB561D1	
ENST00000430195	ENSG00000174151	CYB561D1	
ENST00000496083	ENSG00000184307	ZDHHC23	
ENST00000506482	ENSG00000163116	STPG2	filled
ENST00000358344	ENSG00000197451	HNRNPAB	
ENST00000506339	ENSG00000197451	HNRNPAB	
ENST00000355836	ENSG00000197451	HNRNPAB	
ENST00000514633	ENSG00000197451	HNRNPAB	
ENST00000515193	ENSG00000197451	HNRNPAB	
ENST00000506259	ENSG00000197451	HNRNPAB	
ENST00000504898	ENSG00000197451	HNRNPAB	
ENST00000504796	ENSG00000197451	HNRNPAB	
ENST00000615932	ENSG00000197451	HNRNPAB	
ENST00000237642	ENSG00000118804	FAM47E-STBD1	
ENST00000519266	ENSG00000108515	ENO3	
ENST00000520221	ENSG00000108515	ENO3	
ENST00000522798	ENSG00000108515	ENO3	
ENST00000523008	ENSG00000034239	EFCAB1	
ENST00000435773	ENSG00000162972	C2orf47	
ENST00000356303	ENSG00000156508	EEF1A1	
ENST00000455918	ENSG00000156508	EEF1A1	
ENST00000511575	ENSG00000180818	HOXC10	
ENST00000510197	ENSG00000189157	FAM47E	filled
ENST00000515589	ENSG00000189157	FAM47E	filled
ENST00000316149	ENSG00000123545	NDUFAF4	
ENST00000589627	ENSG00000125753	VASP	
ENST00000245932	ENSG00000125753	VASP	
ENST00000592139	ENSG00000125753	VASP	
ENST00000590603	ENSG00000125753	VASP	
ENST00000586014	ENSG00000125753	VASP	
ENST00000586619	ENSG00000125753	VASP	
ENST00000442995	ENSG00000242028	HYPK	
ENST00000477628	ENSG00000154997	14-Sep	
ENST00000477267	ENSG00000107821	KAZALD1	
ENST00000608812	ENSG00000107821	KAZALD1	

ENST00000525350	ENSG00000236287	ZBED5	filled
ENST00000533903	ENSG00000236287	ZBED5	filled
ENST00000533925	ENSG00000236287	ZBED5	
ENST00000563567	ENSG00000179361	ARID3B	
ENST00000513455	ENSG00000109680	TBC1D19	
ENST00000555141	ENSG00000118308	LRMP	
ENST00000554942	ENSG00000118308	LRMP	
ENST00000555885	ENSG00000118308	LRMP	
ENST00000263033	ENSG00000102362	SYTL4	filled
ENST00000372981	ENSG00000102362	SYTL4	filled
ENST00000276141	ENSG00000102362	SYTL4	filled
ENST00000330175	ENSG00000167100	SAMD14	filled
ENST00000508892	ENSG00000167100	SAMD14	filled
ENST00000503131	ENSG00000167100	SAMD14	filled
ENST00000507043	ENSG00000167100	SAMD14	
ENST00000511964	ENSG00000167100	SAMD14	
ENST00000513474	ENSG00000167100	SAMD14	
ENST00000008876	ENSG00000008735	MAPK8IP2	filled
ENST00000594199	ENSG00000269743	SLC25A53	
ENST00000503008	ENSG00000145416	1-Mar	filled
ENST00000514618	ENSG00000145416	1-Mar	filled
ENST00000507270	ENSG00000145416	1-Mar	filled
ENST00000510696	ENSG00000145416	1-Mar	filled
ENST00000503104	ENSG00000145416	1-Mar	filled
ENST00000508725	ENSG00000145416	1-Mar	filled
ENST00000505391	ENSG00000145416	1-Mar	filled
ENST00000515471	ENSG00000145416	1-Mar	filled
ENST00000339438	ENSG00000009307	CSDE1	filled
ENST00000438362	ENSG00000009307	CSDE1	filled
ENST00000358528	ENSG00000009307	CSDE1	filled
ENST00000261443	ENSG00000009307	CSDE1	filled
ENST00000530886	ENSG00000009307	CSDE1	filled
ENST00000369530	ENSG00000009307	CSDE1	filled
ENST00000534699	ENSG00000009307	CSDE1	filled
ENST00000529046	ENSG00000009307	CSDE1	filled
ENST00000525132	ENSG00000009307	CSDE1	filled
ENST00000525878	ENSG00000009307	CSDE1	filled
ENST00000525970	ENSG00000009307	CSDE1	filled
ENST00000533818	ENSG00000009307	CSDE1	filled
ENST00000524652	ENSG00000009307	CSDE1	filled
ENST00000610726	ENSG00000009307	CSDE1	filled
ENST00000399074	ENSG00000233209	HLA-DQB1	filled
ENST00000422950	ENSG00000233209	HLA-DQB1	
ENST00000486521	ENSG00000233209	HLA-DQB1	filled
ENST00000391859	ENSG00000104894	CD37	
ENST00000598095	ENSG00000104894	CD37	
ENST00000426897	ENSG00000104894	CD37	
ENST00000595725	ENSG00000104894	CD37	
ENST00000323906	ENSG00000104894	CD37	
ENST00000597852	ENSG00000104894	CD37	
ENST00000535669	ENSG00000104894	CD37	
ENST00000594743	ENSG00000104894	CD37	
ENST00000283148	ENSG00000115652	UXS1	filled
ENST00000409501	ENSG00000115652	UXS1	filled
ENST00000473338	ENSG00000115652	UXS1	
ENST00000457835	ENSG00000115652	UXS1	filled
ENST00000436241	ENSG00000115652	UXS1	
ENST00000483426	ENSG00000115652	UXS1	filled
ENST00000569592	ENSG00000155666	KDM8	
ENST00000570760	ENSG00000262526	CTD-2545G14.7	

ENST00000599578	ENSG00000269823	AL513523.1	
ENST00000506724	ENSG00000157514	TSC22D3	
ENST00000505965	ENSG00000157514	TSC22D3	
ENST00000502961	ENSG00000157514	TSC22D3	
ENST00000596542	ENSG00000269307	CTD-2278I10.6	
ENST00000477244	ENSG00000160014	CALM3	
ENST00000598871	ENSG00000160014	CALM3	
ENST00000594523	ENSG00000160014	CALM3	
ENST00000303400	ENSG00000090316	MAEA	filled
ENST00000505177	ENSG00000090316	MAEA	filled
ENST00000513301	ENSG00000090316	MAEA	filled
ENST00000512308	ENSG00000090316	MAEA	filled
ENST00000264750	ENSG00000090316	MAEA	filled
ENST00000509254	ENSG00000090316	MAEA	filled
ENST00000502558	ENSG00000090316	MAEA	filled
ENST00000512842	ENSG00000090316	MAEA	
ENST00000261888	ENSG00000138617	PARP16	filled
ENST00000444347	ENSG00000138617	PARP16	filled
ENST00000374429	ENSG00000107562	CXCL12	filled
ENST00000571081	ENSG00000167264	DUS2	filled
ENST00000570709	ENSG00000167264	DUS2	filled
ENST00000576994	ENSG00000167264	DUS2	filled
ENST00000573699	ENSG00000170291	ELP5	
ENST00000555449	ENSG00000183032	SLC25A21	filled
ENST00000331299	ENSG00000183032	SLC25A21	filled
ENST00000557611	ENSG00000183032	SLC25A21	filled
ENST00000348035	ENSG00000136238	RAC1	filled
ENST00000356142	ENSG00000136238	RAC1	filled
ENST00000495499	ENSG00000136238	RAC1	filled
ENST00000457089	ENSG00000258543	CSNK2B-LY6G5B-1181	
ENST00000611721	ENSG00000258543	CSNK2B-LY6G5B-1181	
ENST00000620322	ENSG00000258543	CSNK2B-LY6G5B-1181	
ENST00000622393	ENSG00000258543	CSNK2B-LY6G5B-1181	
ENST00000553310	ENSG00000170442	KRT86	filled
ENST00000535837	ENSG00000276665	AC245078.2	
ENST00000491186	ENSG00000213123	TCTEX1D2	
ENST00000465757	ENSG00000213123	TCTEX1D2	
ENST00000517710	ENSG00000176623	RMDN1	
ENST00000519247	ENSG00000176623	RMDN1	
ENST00000424383	ENSG00000225968	ELFN1	filled
ENST00000561626	ENSG00000225968	ELFN1	filled
ENST00000497735	ENSG00000047849	MAP4	filled
ENST00000505197	ENSG00000001084	GCLC	filled
ENST00000563887	ENSG00000260548	RP6-24A23.6	
ENST00000398333	ENSG00000250021	C15orf38-AP3S2	filled
ENST00000560224	ENSG00000250021	C15orf38-AP3S2	filled
ENST00000431322	ENSG00000228867	NRM	
ENST00000599286	ENSG00000099330	OCEL1	
ENST00000570667	ENSG00000262771	SSBP1	
ENST00000575059	ENSG00000262771	SSBP1	
ENST00000269205	ENSG00000141437	SLC25A52	
ENST00000374112	ENSG00000178645	C10orf53	filled
ENST00000535836	ENSG00000178645	C10orf53	filled
ENST00000523954	ENSG00000172817	CYP7B1	filled
ENST00000368219	ENSG00000143319	ISG20L2	filled
ENST00000380956	ENSG00000137265	IRF4	filled
ENST00000540433	ENSG00000155974	GRIP1	filled
ENST00000541947	ENSG00000155974	GRIP1	filled
ENST00000538373	ENSG00000155974	GRIP1	filled
ENST00000470718	ENSG00000092010	PSME1	

ENST00000297107	ENSG00000164574	GALNT10	filled
ENST00000520647	ENSG00000164574	GALNT10	filled
ENST00000377661	ENSG00000164574	GALNT10	filled
ENST00000521781	ENSG00000164574	GALNT10	filled
ENST00000380051	ENSG00000124787	RPP40	
ENST00000496876	ENSG00000162992	NEUROD1	
ENST00000295108	ENSG00000162992	NEUROD1	
ENST00000498444	ENSG00000196233	LCOR	filled
ENST00000291547	ENSG00000160199	PKNOX1	filled
ENST00000418336	ENSG00000160199	PKNOX1	
ENST00000431254	ENSG00000241837	ATP5O	
ENST00000418933	ENSG00000241837	ATP5O	
ENST00000595393	ENSG00000105393	BABAM1	filled
ENST00000601043	ENSG00000105393	BABAM1	
ENST00000523475	ENSG00000188343	FAM92A1	
ENST00000587257	ENSG00000126243	LRFN3	
ENST00000535388	ENSG00000174606	ANGEL2	filled
ENST00000374140	ENSG00000138835	RGS3	filled
ENST00000317613	ENSG00000138835	RGS3	filled
ENST00000369893	ENSG00000156398	SFXN2	
ENST00000483377	ENSG00000124813	RUNX2	filled
ENST00000483243	ENSG00000124813	RUNX2	
ENST00000465038	ENSG00000124813	RUNX2	filled
ENST00000371438	ENSG00000124813	RUNX2	filled
ENST00000371436	ENSG00000124813	RUNX2	filled
ENST00000576263	ENSG00000124813	RUNX2	filled
ENST00000473041	ENSG00000124813	RUNX2	
ENST00000371432	ENSG00000124813	RUNX2	filled
ENST00000490407	ENSG00000235956	PRRT1	
ENST00000462634	ENSG00000235956	PRRT1	
ENST00000464533	ENSG00000235956	PRRT1	
ENST00000465667	ENSG00000100325	ASCC2	filled
ENST00000495681	ENSG00000100325	ASCC2	filled
ENST00000453160	ENSG00000100325	ASCC2	filled
ENST00000460313	ENSG00000100325	ASCC2	filled
ENST00000468598	ENSG00000116560	SFPQ	
ENST00000460428	ENSG00000116560	SFPQ	filled
ENST00000470472	ENSG00000116560	SFPQ	filled
ENST00000466745	ENSG00000116560	SFPQ	
ENST00000466213	ENSG00000116560	SFPQ	
ENST00000490668	ENSG00000116560	SFPQ	
ENST00000485365	ENSG00000116560	SFPQ	
ENST00000485454	ENSG00000116560	SFPQ	
ENST00000568599	ENSG00000103066	PLA2G15	
ENST00000517561	ENSG00000132842	AP3B1	filled
ENST00000623013	ENSG00000279473	AC009065.4	
ENST00000278409	ENSG00000149133	OR5F1	
ENST00000515458	ENSG00000175766	EIF4E1B	
ENST00000504597	ENSG00000175766	EIF4E1B	
ENST00000318682	ENSG00000175766	EIF4E1B	
ENST00000296795	ENSG00000164342	TLR3	filled
ENST00000453905	ENSG00000172943	PHF8	
ENST00000352385	ENSG00000103742	IGDCC4	filled
ENST00000558048	ENSG00000103742	IGDCC4	
ENST00000467127	ENSG00000204006	C1orf185	
ENST00000373705	ENSG00000121764	HCRTR1	
ENST00000293780	ENSG00000108556	CHRNE	
ENST00000342247	ENSG00000099954	CECR2	filled
ENST00000262608	ENSG00000099954	CECR2	filled
ENST00000369541	ENSG00000116752	BCAS2	

ENST00000383251	ENSG00000206305	HLA-DQA1	filled
ENST00000399675	ENSG00000206305	HLA-DQA1	filled
ENST00000399678	ENSG00000206305	HLA-DQA1	filled
ENST00000548014	ENSG00000206305	HLA-DQA1	filled
ENST00000359128	ENSG00000167984	NLRC3	filled
ENST00000615877	ENSG00000167984	NLRC3	filled
ENST00000324659	ENSG00000167984	NLRC3	filled
ENST00000603055	ENSG00000167984	NLRC3	
ENST00000525452	ENSG00000231738	TSPAN19	
ENST00000529820	ENSG00000231738	TSPAN19	
ENST00000532498	ENSG00000231738	TSPAN19	
ENST00000532628	ENSG00000231738	TSPAN19	
ENST00000552392	ENSG00000231738	TSPAN19	
ENST00000461347	ENSG00000115053	NCL	
ENST00000366695	ENSG00000181218	HIST3H2A	
ENST00000479547	ENSG00000147459	DOCK5	
ENST00000456402	ENSG00000168032	ENTPD3	filled
ENST00000328600	ENSG00000182261	NLRP10	
ENST00000372950	ENSG00000213551	DNAJC9	
ENST00000417551	ENSG00000091140	DLD	
ENST00000399060	ENSG00000206206	DAXX	
ENST00000383062	ENSG00000206206	DAXX	
ENST00000484131	ENSG00000206206	DAXX	
ENST00000461796	ENSG00000206206	DAXX	
ENST00000459766	ENSG00000206206	DAXX	
ENST00000428268	ENSG00000206206	DAXX	
ENST00000414272	ENSG00000206206	DAXX	filled
ENST00000474819	ENSG00000206206	DAXX	
ENST00000457553	ENSG00000206206	DAXX	
ENST00000478948	ENSG00000206206	DAXX	
ENST00000612888	ENSG00000206206	DAXX	
ENST00000613912	ENSG00000206206	DAXX	
ENST00000509952	ENSG00000144908	ALDH1L1	filled
ENST00000511283	ENSG00000144908	ALDH1L1	filled
ENST00000472599	ENSG00000151576	QTRTD1	
ENST00000462966	ENSG00000151576	QTRTD1	
ENST00000546556	ENSG00000135678	CPM	filled
ENST00000551897	ENSG00000135678	CPM	filled
ENST00000546373	ENSG00000135678	CPM	filled
ENST00000379535	ENSG00000185787	MORF4L1	filled
ENST00000463624	ENSG00000164091	WDR82	
ENST00000303459	ENSG00000169519	METTL15	
ENST00000380834	ENSG00000138092	CENPO	filled
ENST00000473706	ENSG00000138092	CENPO	filled
ENST00000464156	ENSG00000138092	CENPO	
ENST00000395845	ENSG00000138092	CENPO	
ENST00000568059	ENSG00000140398	NEIL1	
ENST00000561643	ENSG00000140398	NEIL1	
ENST00000565051	ENSG00000140398	NEIL1	
ENST00000274849	ENSG00000146109	ABT1	
ENST00000527445	ENSG00000154319	FAM167A	
ENST00000531804	ENSG00000154319	FAM167A	
ENST00000349607	ENSG00000145113	MUC4	filled
ENST00000424132	ENSG00000144468	RHBDD1	
ENST00000441049	ENSG00000188010	MORN2	filled
ENST00000532967	ENSG00000250741	NT5C1B-RDH14	filled
ENST00000444297	ENSG00000250741	NT5C1B-RDH14	filled
ENST00000587885	ENSG00000160888	IER2	
ENST00000554992	ENSG00000136319	TTC5	
ENST00000521788	ENSG00000184374	COLEC10	

ENST00000370532	ENSG00000184258	CDR1	
ENST00000548197	ENSG00000089169	RPH3A	filled
ENST00000543106	ENSG00000089169	RPH3A	filled
ENST00000551593	ENSG00000089169	RPH3A	filled
ENST00000361531	ENSG00000143314	MRPL24	
ENST00000368211	ENSG00000143314	MRPL24	
ENST00000434558	ENSG00000143314	MRPL24	
ENST00000420938	ENSG00000143314	MRPL24	
ENST00000620573	ENSG00000276082	CNOT3	filled
ENST00000267484	ENSG00000139970	RTN1	filled
ENST00000611068	ENSG00000139970	RTN1	filled
ENST00000328747	ENSG00000146757	ZNF92	
ENST00000431504	ENSG00000146757	ZNF92	
ENST00000450302	ENSG00000146757	ZNF92	filled
ENST00000357512	ENSG00000146757	ZNF92	
ENST00000395067	ENSG00000066697	MSANTD3	filled
ENST00000398977	ENSG00000066697	MSANTD3	filled
ENST00000374885	ENSG00000066697	MSANTD3	filled
ENST00000374886	ENSG00000066697	MSANTD3	filled
ENST00000622639	ENSG00000066697	MSANTD3	filled
ENST00000613183	ENSG00000066697	MSANTD3	filled
ENST00000240727	ENSG00000121413	ZSCAN18	filled
ENST00000594191	ENSG00000121413	ZSCAN18	
ENST00000595944	ENSG00000121413	ZSCAN18	
ENST00000521564	ENSG00000147614	ATP6V0D2	filled
ENST00000592957	ENSG00000141759	TXNL4A	
ENST00000624399	ENSG00000279988	AL022578.1	
ENST00000398221	ENSG00000214376	VSTM5	
ENST00000610966	ENSG00000166111	SVOP	filled
ENST00000550436	ENSG00000166111	SVOP	filled
ENST00000546618	ENSG00000166111	SVOP	filled
ENST00000548229	ENSG00000166111	SVOP	filled
ENST00000625176	ENSG00000279909	AC110615.1	
ENST00000369851	ENSG00000065135	GNAI3	filled
ENST00000601808	ENSG00000186272	ZNF17	
ENST00000602050	ENSG00000186272	ZNF17	
ENST00000376185	ENSG00000254870	ATP6V1G2-DDX39	filled
ENST00000480131	ENSG00000254870	ATP6V1G2-DDX39B	
ENST00000475917	ENSG00000254870	ATP6V1G2-DDX39B	
ENST00000523975	ENSG00000170791	CHCHD7	filled
ENST00000263257	ENSG00000104967	NOVA2	
ENST00000504251	ENSG00000113638	TTC33	
ENST00000524391	ENSG00000164794	KCNV1	
ENST00000239174	ENSG00000120094	HOXB1	
ENST00000454552	ENSG00000249590	RP4-539M6.19	
ENST00000439838	ENSG00000249590	RP4-539M6.19	
ENST00000439023	ENSG00000249590	RP4-539M6.19	
ENST00000470096	ENSG00000115998	C2orf42	filled
ENST00000460663	ENSG00000204420	C6orf25	
ENST00000331664	ENSG00000179270	C2orf71	
ENST00000394123	ENSG00000145907	G3BP1	filled
ENST00000449505	ENSG00000163939	PBRM1	
ENST00000450271	ENSG00000163939	PBRM1	
ENST00000439181	ENSG00000163939	PBRM1	
ENST00000458294	ENSG00000163939	PBRM1	
ENST00000424867	ENSG00000163939	PBRM1	
ENST00000407886	ENSG00000206069	TMEM211	
ENST00000423535	ENSG00000206069	TMEM211	
ENST00000376476	ENSG00000175206	NPPA	
ENST00000498088	ENSG00000172458	IL17D	

ENST00000449707	ENSG00000115568	ZNF142	filled
ENST00000433921	ENSG00000115568	ZNF142	filled
ENST00000450765	ENSG00000115568	ZNF142	filled
ENST00000450560	ENSG00000115568	ZNF142	
ENST00000432460	ENSG00000115568	ZNF142	
ENST00000411696	ENSG00000115568	ZNF142	filled
ENST00000263054	ENSG00000108018	SORCS1	filled
ENST00000344440	ENSG00000108018	SORCS1	filled
ENST00000622431	ENSG00000108018	SORCS1	filled
ENST00000612154	ENSG00000108018	SORCS1	filled
ENST00000434158	ENSG00000146828	SLC12A9	
ENST00000527308	ENSG00000172367	PDZD3	
ENST00000526279	ENSG00000172367	PDZD3	
ENST00000531114	ENSG00000172367	PDZD3	
ENST00000526836	ENSG00000172367	PDZD3	
ENST00000528730	ENSG00000172367	PDZD3	
ENST00000529573	ENSG00000172367	PDZD3	
ENST00000483565	ENSG00000231618	PPT2	
ENST00000317201	ENSG00000105997	HOXA3	
ENST00000522788	ENSG00000105997	HOXA3	
ENST00000521401	ENSG00000105997	HOXA3	
ENST00000518451	ENSG00000105997	HOXA3	filled
ENST00000496384	ENSG00000157764	BRAF	filled
ENST00000619012	ENSG00000106123	EPHB6	filled
ENST00000497095	ENSG00000106123	EPHB6	
ENST00000616380	ENSG00000106123	EPHB6	filled
ENST00000614832	ENSG00000106123	EPHB6	filled
ENST00000355280	ENSG00000141503	MINK1	filled
ENST00000572330	ENSG00000141503	MINK1	filled
ENST00000347992	ENSG00000141503	MINK1	filled
ENST00000574453	ENSG00000141503	MINK1	filled
ENST00000453408	ENSG00000141503	MINK1	filled
ENST00000555551	ENSG00000135517	MIP	filled
ENST00000359326	ENSG00000000460	C1orf112	filled
ENST00000459772	ENSG00000000460	C1orf112	filled
ENST00000286031	ENSG00000000460	C1orf112	filled
ENST00000413811	ENSG00000000460	C1orf112	filled
ENST00000565857	ENSG00000166780	C16orf45	filled
ENST00000368323	ENSG00000143622	RIT1	
ENST00000614467	ENSG00000112624	GLTSCR1L	filled
ENST00000296125	ENSG00000163810	TGM4	filled
ENST00000471637	ENSG00000163810	TGM4	
ENST00000422219	ENSG00000163810	TGM4	filled
ENST00000459830	ENSG00000163810	TGM4	
ENST00000463892	ENSG00000107863	ARHGAP21	
ENST00000506786	ENSG00000176293	ZNF135	
ENST00000568075	ENSG00000197272	IL27	
ENST00000366691	ENSG00000116574	RHOU	
ENST00000556415	ENSG00000182379	NXPH4	
ENST00000555154	ENSG00000182379	NXPH4	
ENST00000525427	ENSG00000019144	PHLDB1	filled
ENST00000600882	ENSG00000019144	PHLDB1	filled
ENST00000535299	ENSG00000185652	NTF3	filled
ENST00000544836	ENSG00000185652	NTF3	
ENST00000541234	ENSG00000185652	NTF3	
ENST00000486776	ENSG00000125850	OVOL2	filled
ENST00000586402	ENSG00000161091	MFSD12	
ENST00000504352	ENSG00000132570	PCBD2	filled
ENST00000543743	ENSG00000101493	ZNF516	
ENST00000532857	ENSG00000101493	ZNF516	filled

ENST00000513706	ENSG00000164209	SLC25A46	
ENST00000624408	ENSG00000279291	AC069547.1	
ENST00000593226	ENSG00000104881	PPP1R13L	
ENST00000420148	ENSG00000163939	PBRM1	
ENST00000489476	ENSG00000144034	TPRKB	
ENST00000396904	ENSG00000272968	RBAK-RBAKDN	filled
ENST00000169298	ENSG00000073849	ST6GAL1	filled
ENST00000487031	ENSG00000073849	ST6GAL1	
ENST00000455441	ENSG00000073849	ST6GAL1	filled
ENST00000427315	ENSG00000073849	ST6GAL1	filled
ENST00000468614	ENSG00000073849	ST6GAL1	
ENST00000457772	ENSG00000073849	ST6GAL1	filled
ENST00000508190	ENSG00000180806	HOXC9	
ENST00000519842	ENSG00000122592	HOXA7	
ENST00000409469	ENSG00000055130	CUL1	filled
ENST00000602748	ENSG00000055130	CUL1	filled
ENST00000454212	ENSG00000164048	ZNF589	
ENST00000395472	ENSG00000180008	SOCS4	filled
ENST00000339298	ENSG00000180008	SOCS4	filled
ENST00000486255	ENSG00000102287	GABRE	
ENST00000466387	ENSG00000066032	CTNNA2	filled
ENST00000496251	ENSG00000066032	CTNNA2	filled
ENST00000467488	ENSG00000066032	CTNNA2	
ENST00000497804	ENSG00000066032	CTNNA2	filled
ENST00000496558	ENSG00000066032	CTNNA2	filled
ENST00000409971	ENSG00000066032	CTNNA2	filled
ENST00000409266	ENSG00000066032	CTNNA2	filled
ENST00000402739	ENSG00000066032	CTNNA2	filled
ENST00000343114	ENSG00000066032	CTNNA2	filled
ENST00000361291	ENSG00000066032	CTNNA2	filled
ENST00000540488	ENSG00000066032	CTNNA2	filled
ENST00000422650	ENSG00000229684	TUBB	
ENST00000454454	ENSG00000229684	TUBB	
ENST00000396825	ENSG00000197296	FITM2	
ENST00000361673	ENSG00000198796	ALPK2	filled
ENST00000534482	ENSG00000172922	RNASEH2C	
ENST00000308418	ENSG00000172922	RNASEH2C	
ENST00000533698	ENSG00000172922	RNASEH2C	
ENST00000531596	ENSG00000172922	RNASEH2C	
ENST00000498228	ENSG00000162390	ACOT11	filled
ENST00000411588	ENSG00000231676	OR2J2	
ENST00000255198	ENSG00000132846	ZBED3	
ENST00000554858	ENSG00000100626	GALNT16	
ENST00000556829	ENSG00000100626	GALNT16	
ENST00000479335	ENSG00000182185	RAD51B	filled
ENST00000487861	ENSG00000182185	RAD51B	filled
ENST00000471583	ENSG00000182185	RAD51B	filled
ENST00000487270	ENSG00000182185	RAD51B	filled
ENST00000488612	ENSG00000182185	RAD51B	filled
ENST00000553595	ENSG00000182185	RAD51B	filled
ENST00000497460	ENSG00000182185	RAD51B	filled
ENST00000492236	ENSG00000182185	RAD51B	filled
ENST00000554244	ENSG00000182185	RAD51B	filled
ENST00000478014	ENSG00000182185	RAD51B	filled
ENST00000460526	ENSG00000182185	RAD51B	filled
ENST00000469165	ENSG00000182185	RAD51B	filled
ENST00000468382	ENSG00000182185	RAD51B	filled
ENST00000554183	ENSG00000182185	RAD51B	
ENST00000554575	ENSG00000182185	RAD51B	
ENST00000555782	ENSG00000182185	RAD51B	
ENST00000556251	ENSG00000182185	RAD51B	filled
ENST00000367012	ENSG00000082497	SERTAD4	
ENST00000482421	ENSG00000082497	SERTAD4	
ENST00000483884	ENSG00000082497	SERTAD4	
ENST00000611448	ENSG00000274965	TRPM1	filled
ENST00000302945	ENSG00000169840	GSX1	filled
ENST00000328705	ENSG00000180881	CAPS2	filled
ENST00000493070	ENSG00000180881	CAPS2	
ENST00000399161	ENSG00000186487	MYT1L	filled
ENST00000428368	ENSG00000186487	MYT1L	filled
ENST00000479156	ENSG00000186487	MYT1L	filled
ENST00000476547	ENSG00000186487	MYT1L	
ENST00000428880	ENSG00000074582	BCS1L	
ENST00000430322	ENSG00000074582	BCS1L	
ENST00000554229	ENSG00000205669	ACOT6	
ENST00000559833	ENSG00000103671	TRIP4	filled
ENST00000559565	ENSG00000103671	TRIP4	filled
ENST00000261884	ENSG00000103671	TRIP4	filled
ENST00000558820	ENSG00000103671	TRIP4	filled
ENST00000560567	ENSG00000103671	TRIP4	filled
ENST00000561265	ENSG00000103671	TRIP4	filled
ENST00000560475	ENSG00000103671	TRIP4	filled
ENST00000490980	ENSG00000165879	FRAT1	
ENST00000420475	ENSG00000136826	KLF4	
ENST00000352511	ENSG00000114739	ACVR2B	
ENST00000562340	ENSG00000175267	VWA3A	
ENST00000567131	ENSG00000175267	VWA3A	
ENST00000338888	ENSG00000020633	RUNX3	filled
ENST00000479341	ENSG00000020633	RUNX3	filled
ENST00000425357	ENSG00000188958	UTS2B	
ENST00000463450	ENSG00000188958	UTS2B	
ENST00000490825	ENSG00000188958	UTS2B	
ENST00000464814	ENSG00000188958	UTS2B	
ENST00000512982	ENSG00000082074	FYB	filled
ENST00000512138	ENSG00000082074	FYB	filled
ENST00000396652	ENSG00000171243	SOSTDC1	
ENST00000243103	ENSG00000123407	HOXC12	
ENST00000596411	ENSG00000160460	SPTBN4	
ENST00000376592	ENSG00000177000	MTHFR	
ENST00000376590	ENSG00000177000	MTHFR	
ENST00000376486	ENSG00000177000	MTHFR	
ENST00000376583	ENSG00000177000	MTHFR	
ENST00000526462	ENSG00000182208	MOB2	filled
ENST00000372242	ENSG00000126106	TMEM53	
ENST00000372243	ENSG00000126106	TMEM53	
ENST00000372244	ENSG00000126106	TMEM53	
ENST00000355327	ENSG00000187720	THSD4	filled
ENST00000595857	ENSG00000225950	NTF4	filled
ENST00000594938	ENSG00000225950	NTF4	
ENST00000381249	ENSG00000240857	RDH14	
ENST00000415248	ENSG00000186230	ZNF749	
ENST00000467791	ENSG00000215126	CBWD7	
ENST00000513201	ENSG00000218336	TENM3	filled
ENST00000512480	ENSG00000218336	TENM3	filled
ENST00000511685	ENSG00000218336	TENM3	filled
ENST00000510504	ENSG00000218336	TENM3	filled
ENST00000396863	ENSG00000101057	MYBL2	
ENST00000592726	ENSG00000108443	RPS6KB1	filled
ENST00000587061	ENSG00000108443	RPS6KB1	
ENST00000515820	ENSG00000163623	NKX6-1	

ENST00000295886	ENSG00000163623	NKX6-1	
ENST00000466801	ENSG00000187514	PTMA	filled
ENST00000486911	ENSG00000155621	C9orf85	filled
ENST00000530112	ENSG00000185670	ZBTB3	
ENST00000575785	ENSG00000262621	LA16c-306E5.2	filled
ENST00000618352	ENSG00000262621	LA16c-306E5.2	
ENST00000170447	ENSG00000075975	MKRN2	filled
ENST00000519774	ENSG00000100099	HPS4	filled
ENST00000493455	ENSG00000100099	HPS4	filled
ENST00000624173	ENSG00000279968	RP3-509I19.11	filled
ENST00000279451	ENSG00000149970	CNKSR2	filled
ENST00000379510	ENSG00000149970	CNKSR2	filled
ENST00000425654	ENSG00000149970	CNKSR2	filled
ENST00000543067	ENSG00000149970	CNKSR2	filled
ENST00000572428	ENSG00000166676	TVP23A	
ENST00000481920	ENSG00000143303	RRNAD1	filled
ENST00000561673	ENSG00000179335	CLK3	
ENST00000397898	ENSG00000184900	SUMO3	
ENST00000332859	ENSG00000184900	SUMO3	
ENST00000479153	ENSG00000184900	SUMO3	
ENST00000411651	ENSG00000184900	SUMO3	
ENST00000470018	ENSG00000084072	PPIE	
ENST00000382584	ENSG00000163728	TTC14	
ENST00000490617	ENSG00000176896	TCEANC	
ENST00000380600	ENSG00000176896	TCEANC	
ENST00000467590	ENSG00000176896	TCEANC	
ENST00000463321	ENSG00000176896	TCEANC	
ENST00000597576	ENSG00000188785	ZNF548	filled
ENST00000421388	ENSG00000235711	ANKRD34C	
ENST00000616800	ENSG00000188690	UROS	
ENST00000622016	ENSG00000188690	UROS	
ENST00000455486	ENSG00000276238	AL133373.1	
ENST00000429617	ENSG00000165806	CASP7	filled
ENST00000345633	ENSG00000165806	CASP7	filled
ENST00000614447	ENSG00000165806	CASP7	filled
ENST00000369321	ENSG00000165806	CASP7	filled
ENST00000621345	ENSG00000165806	CASP7	filled
ENST00000443952	ENSG00000258589	CSNK2B-LY6G5B-1181	
ENST00000614071	ENSG00000258589	CSNK2B-LY6G5B-1181	
ENST00000615363	ENSG00000258589	CSNK2B-LY6G5B-1181	
ENST00000612713	ENSG00000258589	CSNK2B-LY6G5B-1181	
ENST00000528911	ENSG00000141971	MVB12A	filled
ENST00000528604	ENSG00000141971	MVB12A	filled
ENST00000529490	ENSG00000141971	MVB12A	filled
ENST00000600514	ENSG00000141971	MVB12A	filled
ENST00000606738	ENSG00000269113	TRABD2B	filled
ENST00000435576	ENSG00000269113	TRABD2B	filled
ENST00000460862	ENSG00000161203	AP2M1	
ENST00000465180	ENSG00000143321	HDGF	filled
ENST00000555664	ENSG00000133958	UNC79	filled
ENST00000553484	ENSG00000133958	UNC79	filled
ENST00000393151	ENSG00000133958	UNC79	filled
ENST00000554549	ENSG00000133958	UNC79	
ENST00000621021	ENSG00000133958	UNC79	filled
ENST00000438445	ENSG00000138031	ADCY3	
ENST00000473987	ENSG00000135175	OCM2	
ENST00000357457	ENSG00000113163	COL4A3BP	
ENST00000604926	ENSG00000113163	COL4A3BP	
ENST00000380494	ENSG00000113163	COL4A3BP	filled
ENST00000509935	ENSG00000123213	NLN	filled

ENST00000301831	ENSG00000168038	ULK4	filled
ENST00000615274	ENSG00000236884	HLA-DRB1	filled
ENST00000321895	ENSG00000177352	CCDC71	filled
ENST00000344836	ENSG00000187555	USP7	filled
ENST00000563961	ENSG00000187555	USP7	filled
ENST00000566273	ENSG00000187555	USP7	
ENST00000589162	ENSG00000126247	CAPNS1	
ENST00000554581	ENSG00000142208	AKT1	filled
ENST00000402615	ENSG00000142208	AKT1	filled
ENST00000555528	ENSG00000142208	AKT1	filled
ENST00000555458	ENSG00000142208	AKT1	filled
ENST00000371989	ENSG00000148248	SURF4	
ENST00000613129	ENSG00000148248	SURF4	
ENST00000545297	ENSG00000148248	SURF4	
ENST00000618229	ENSG00000148248	SURF4	
ENST00000523148	ENSG00000055950	MRPL43	filled
ENST00000438949	ENSG00000106415	GLCCI1	
ENST00000459638	ENSG00000114541	FRMD4B	filled
ENST00000497880	ENSG00000114541	FRMD4B	filled
ENST00000497757	ENSG00000114541	FRMD4B	filled
ENST00000544387	ENSG00000256269	HMBS	
ENST00000539045	ENSG00000256269	HMBS	
ENST00000525144	ENSG00000149571	KIRREL3	filled
ENST00000529097	ENSG00000149571	KIRREL3	filled
ENST00000525704	ENSG00000149571	KIRREL3	filled
ENST00000533026	ENSG00000149571	KIRREL3	filled
ENST00000547738	ENSG00000149571	KIRREL3	filled
ENST00000549874	ENSG00000149571	KIRREL3	filled
ENST00000416561	ENSG00000149571	KIRREL3	filled
ENST00000409978	ENSG00000214694	ARHGEF33	filled
ENST00000338146	ENSG00000176422	SPRYD4	
ENST00000238647	ENSG00000119669	IRF2BPL	
ENST00000624731	ENSG00000280316	AL365181.2	
ENST00000612955	ENSG00000132938	MTUS2	filled
ENST00000380808	ENSG00000132938	MTUS2	filled
ENST00000431530	ENSG00000132938	MTUS2	filled
ENST00000542829	ENSG00000132938	MTUS2	filled
ENST00000482108	ENSG00000242265	PEG10	
ENST00000488574	ENSG00000242265	PEG10	
ENST00000612748	ENSG00000242265	PEG10	
ENST00000617526	ENSG00000242265	PEG10	
ENST00000615790	ENSG00000242265	PEG10	
ENST00000612941	ENSG00000242265	PEG10	
ENST00000465184	ENSG00000242265	PEG10	
ENST00000332995	ENSG00000112137	PHACTR1	filled
ENST00000379350	ENSG00000112137	PHACTR1	filled
ENST00000434980	ENSG00000123243	ITIH5	filled
ENST00000397145	ENSG00000123243	ITIH5	filled
ENST00000624109	ENSG00000279346	AC104389.1	
ENST00000517349	ENSG00000253457	SMIM18	
ENST00000553221	ENSG00000135452	TSPAN31	
ENST00000547311	ENSG00000135452	TSPAN31	
ENST00000550528	ENSG00000135452	TSPAN31	
ENST00000547992	ENSG00000135452	TSPAN31	
ENST00000328041	ENSG00000185052	SLC24A3	filled
ENST00000613834	ENSG00000185052	SLC24A3	filled
ENST00000461628	ENSG00000137203	TFAP2A	filled
ENST00000335080	ENSG00000186280	KDM4D	
ENST00000536741	ENSG00000186280	KDM4D	
ENST00000306336	ENSG00000168887	C2orf68	

ENST00000619808	ENSG00000275650	TTYH1	
ENST00000252137	ENSG00000100056	DGCR14	
ENST00000287042	ENSG00000156486	KCNS2	
ENST00000521839	ENSG00000156486	KCNS2	
ENST00000373115	ENSG00000122863	CHST3	
ENST00000449866	ENSG00000136052	SLC41A2	
ENST00000424946	ENSG00000136052	SLC41A2	
ENST00000417469	ENSG00000136052	SLC41A2	
ENST00000432951	ENSG00000136052	SLC41A2	
ENST00000559405	ENSG00000104047	DTWD1	
ENST00000437875	ENSG00000213160	KLHL23	filled
ENST00000448589	ENSG00000213160	KLHL23	filled
ENST00000616183	ENSG00000160352	ZNF714	filled
ENST00000240100	ENSG00000120875	DUSP4	filled
ENST00000569332	ENSG00000186153	WWOX	filled
ENST00000566662	ENSG00000186153	WWOX	filled
ENST00000408984	ENSG00000186153	WWOX	filled
ENST00000562639	ENSG00000186153	WWOX	filled
ENST00000297267	ENSG00000164694	FNDC1	filled
ENST00000333190	ENSG00000182348	ZNF804B	filled
ENST00000606162	ENSG00000122378	FAM213A	
ENST00000491853	ENSG00000143294	PRCC	filled
ENST00000327171	ENSG00000156958	GALK2	filled
ENST00000558399	ENSG00000156958	GALK2	filled
ENST00000559580	ENSG00000156958	GALK2	filled
ENST00000408903	ENSG00000171444	MCC	filled
ENST00000477985	ENSG00000145919	BOD1	
ENST00000593240	ENSG00000104866	PPP1R37	
ENST00000540059	ENSG00000104866	PPP1R37	
ENST00000313401	ENSG00000176749	CDK5R1	
ENST00000603276	ENSG00000062282	DGAT2	filled
ENST00000475904	ENSG00000102032	RENBP	
ENST00000261249	ENSG00000126814	TRMT5	
ENST00000553903	ENSG00000126814	TRMT5	
ENST00000506888	ENSG00000213204	C6orf165	filled
ENST00000368704	ENSG00000188643	S100A16	
ENST00000368703	ENSG00000188643	S100A16	
ENST00000558625	ENSG00000166069	TMCO5A	
ENST00000559502	ENSG00000166069	TMCO5A	
ENST00000586986	ENSG00000121068	TBX2	
ENST00000624653	ENSG00000279854	RPS4Y2	
ENST00000278742	ENSG00000149418	ST14	filled
ENST00000460737	ENSG00000206404	LY6G5C	
ENST00000494386	ENSG00000206404	LY6G5C	
ENST00000449562	ENSG00000206404	LY6G5C	
ENST00000440737	ENSG00000182568	SATB1	filled
ENST00000415069	ENSG00000182568	SATB1	filled
ENST00000493952	ENSG00000182568	SATB1	filled
ENST00000540583	ENSG00000178878	APOLD1	filled
ENST00000534843	ENSG00000178878	APOLD1	filled
ENST00000326765	ENSG00000178878	APOLD1	filled
ENST00000588943	ENSG00000178878	APOLD1	filled
ENST00000356591	ENSG00000178878	APOLD1	
ENST00000583728	ENSG00000198720	ANKRD13B	
ENST00000487527	ENSG00000198720	ANKRD13B	
ENST00000394859	ENSG00000198720	ANKRD13B	
ENST00000488766	ENSG00000198720	ANKRD13B	
ENST00000614878	ENSG00000198720	ANKRD13B	
ENST00000556818	ENSG00000126804	ZBTB1	
ENST00000448879	ENSG00000075213	SEMA3A	filled

ENST00000424555	ENSG00000075213	SEMA3A	filled
ENST00000471474	ENSG00000075213	SEMA3A	filled
ENST00000490883	ENSG00000075213	SEMA3A	filled
ENST00000495075	ENSG00000175110	MRPS22	filled
ENST00000495225	ENSG00000175110	MRPS22	filled
ENST00000421560	ENSG00000173402	DAG1	filled
ENST00000308775	ENSG00000173402	DAG1	filled
ENST00000431960	ENSG00000173402	DAG1	filled
ENST00000541308	ENSG00000173402	DAG1	filled
ENST00000545947	ENSG00000173402	DAG1	filled
ENST00000539901	ENSG00000173402	DAG1	filled
ENST00000538711	ENSG00000173402	DAG1	filled
ENST00000389082	ENSG00000170456	DENND5B	filled
ENST00000536562	ENSG00000170456	DENND5B	filled
ENST00000439874	ENSG00000229185	OR2H2	
ENST00000589178	ENSG00000186814	ZSCAN30	filled
ENST00000262738	ENSG00000075275	CELSR1	filled
ENST00000454637	ENSG00000075275	CELSR1	
ENST00000263563	ENSG00000107719	PALD1	
ENST00000360657	ENSG00000124564	SLC17A3	filled
ENST00000534649	ENSG00000177595	PIDD1	
ENST00000580392	ENSG00000126858	RHOT1	filled
ENST00000584852	ENSG00000126858	RHOT1	filled
ENST00000582586	ENSG00000126858	RHOT1	filled
ENST00000571908	ENSG00000173821	RNF213	
ENST00000555220	ENSG00000089775	ZBTB25	
ENST00000555424	ENSG00000089775	ZBTB25	
ENST00000541341	ENSG00000072609	CHFR	
ENST00000536843	ENSG00000072609	CHFR	
ENST00000542859	ENSG00000073910	FRY	filled
ENST00000463566	ENSG00000073910	FRY	
ENST00000436046	ENSG00000073910	FRY	filled
ENST00000380250	ENSG00000073910	FRY	filled
ENST00000301744	ENSG00000167981	ZNF597	
ENST00000478662	ENSG00000174640	SLCO2A1	filled
ENST00000479147	ENSG00000136877	FPGS	
ENST00000479375	ENSG00000136877	FPGS	
ENST00000266604	ENSG00000139233	LLPH	
ENST00000480687	ENSG00000173540	GMPPB	
ENST00000308375	ENSG00000173540	GMPPB	
ENST00000606214	ENSG00000145979	TBC1D7	filled
ENST00000421203	ENSG00000145979	TBC1D7	filled
ENST00000616997	ENSG00000277421	AC246793.1	
ENST00000374084	ENSG00000090020	SLC9A1	filled
ENST00000466796	ENSG00000160055	TMEM234	
ENST00000491434	ENSG00000160055	TMEM234	
ENST00000489170	ENSG00000160055	TMEM234	
ENST00000461402	ENSG00000160055	TMEM234	
ENST00000341901	ENSG00000188322	SBK1	filled
ENST00000381486	ENSG00000196208	GREB1	filled
ENST00000488372	ENSG00000204406	MBD5	filled
ENST00000407073	ENSG00000204406	MBD5	filled
ENST00000496158	ENSG00000204406	MBD5	filled
ENST00000478190	ENSG00000204406	MBD5	
ENST00000473478	ENSG00000204406	MBD5	filled
ENST00000470063	ENSG00000204406	MBD5	filled
ENST00000469438	ENSG00000204406	MBD5	
ENST00000478804	ENSG00000204406	MBD5	filled
ENST00000551215	ENSG00000110911	SLC11A2	
ENST00000526090	ENSG00000137747	TMPRSS13	

ENST00000528135	ENSG00000137747	TMPRSS13	
ENST00000472828	ENSG00000163492	CCDC141	
ENST00000443758	ENSG00000163492	CCDC141	filled
ENST00000446116	ENSG00000163492	CCDC141	filled
ENST00000409284	ENSG00000163492	CCDC141	filled
ENST00000420890	ENSG00000163492	CCDC141	filled
ENST00000417601	ENSG00000234258	HSPA1L	
ENST00000410024	ENSG00000144524	COPS7B	filled
ENST00000479430	ENSG00000144524	COPS7B	filled
ENST00000466901	ENSG00000144524	COPS7B	filled
ENST00000508527	ENSG00000164241	C5orf63	
ENST00000606937	ENSG00000164241	C5orf63	
ENST00000606042	ENSG00000164241	C5orf63	
ENST00000535381	ENSG00000164241	C5orf63	
ENST00000455118	ENSG00000154743	TSEN2	filled
ENST00000475595	ENSG00000154743	TSEN2	
ENST00000261700	ENSG00000087302	C14orf166	filled
ENST00000558447	ENSG00000104067	TJP1	filled
ENST00000473741	ENSG00000104067	TJP1	filled
ENST00000422911	ENSG00000141956	PRDM15	filled
ENST00000441787	ENSG00000141956	PRDM15	filled
ENST00000449395	ENSG00000141956	PRDM15	filled
ENST00000398548	ENSG00000141956	PRDM15	filled
ENST00000433067	ENSG00000141956	PRDM15	filled
ENST00000269844	ENSG00000141956	PRDM15	filled
ENST00000584068	ENSG00000270339	PPIAL4C	
ENST00000590532	ENSG00000266997	RP11-886H22.1	
ENST00000486916	ENSG00000112499	SLC22A2	
ENST00000611166	ENSG00000274336	CDIP1	
ENST00000482861	ENSG00000144290	SLC4A10	filled
ENST00000605990	ENSG00000144290	SLC4A10	
ENST00000494935	ENSG00000112320	SOBP	
ENST00000429911	ENSG00000106392	C1GALT1	filled
ENST00000223122	ENSG00000106392	C1GALT1	
ENST00000557222	ENSG00000100749	VRK1	filled
ENST00000483271	ENSG00000162852	CNST	
ENST00000366513	ENSG00000162852	CNST	
ENST00000366512	ENSG00000162852	CNST	
ENST00000366511	ENSG00000162852	CNST	
ENST00000509663	ENSG00000068796	KIF2A	filled
ENST00000455168	ENSG00000134108	ARL8B	
ENST00000419534	ENSG00000134108	ARL8B	
ENST00000532567	ENSG00000118181	RPS25	
ENST00000527791	ENSG00000118181	RPS25	
ENST00000527673	ENSG00000118181	RPS25	
ENST00000527853	ENSG00000118181	RPS25	
ENST00000524864	ENSG00000118181	RPS25	
ENST00000306087	ENSG00000168875	SOX14	filled
ENST00000404234	ENSG00000100095	SEZ6L	filled
ENST00000360929	ENSG00000100095	SEZ6L	filled
ENST00000248933	ENSG00000100095	SEZ6L	filled
ENST00000343706	ENSG00000100095	SEZ6L	filled
ENST00000529632	ENSG00000100095	SEZ6L	filled
ENST00000421544	ENSG00000164418	GRIK2	filled
ENST00000413795	ENSG00000164418	GRIK2	filled
ENST00000369138	ENSG00000164418	GRIK2	filled
ENST00000358361	ENSG00000164418	GRIK2	filled
ENST00000379499	ENSG00000185915	KLHL34	
ENST00000589720	ENSG00000197563	PIGN	
ENST00000590765	ENSG00000197563	PIGN	

ENST00000585458	ENSG00000197563	PIGN	
ENST00000588571	ENSG00000197563	PIGN	
ENST00000589339	ENSG00000197563	PIGN	
ENST00000585344	ENSG00000197563	PIGN	
ENST00000587134	ENSG00000197563	PIGN	
ENST00000593225	ENSG00000197563	PIGN	
ENST00000483649	ENSG00000204147	ASAH2B	
ENST00000371826	ENSG00000119922	IFIT2	
ENST00000611722	ENSG00000119922	IFIT2	
ENST00000377067	ENSG00000179399	GPC5	filled
ENST00000618283	ENSG00000179399	GPC5	
ENST00000455332	ENSG00000036672	USP2	filled
ENST00000260187	ENSG00000036672	USP2	filled
ENST00000531070	ENSG00000036672	USP2	
ENST00000527843	ENSG00000036672	USP2	
ENST00000399065	ENSG00000231286	HLA-DQB1	filled
ENST00000413089	ENSG00000231286	HLA-DQB1	
ENST00000608353	ENSG00000111850	SMIM8	filled
ENST00000229570	ENSG00000111850	SMIM8	
ENST00000608525	ENSG00000111850	SMIM8	
ENST00000608868	ENSG00000111850	SMIM8	filled
ENST00000392863	ENSG00000111850	SMIM8	
ENST00000242576	ENSG00000076248	UNG	
ENST00000336865	ENSG00000076248	UNG	
ENST00000446767	ENSG00000076248	UNG	
ENST00000565558	ENSG00000102904	TSNAXIP1	
ENST00000370766	ENSG00000186376	ZNF75D	filled
ENST00000533756	ENSG00000175115	PACS1	filled
ENST00000527380	ENSG00000175115	PACS1	filled
ENST00000259211	ENSG00000136694	IL36A	
ENST00000553142	ENSG00000135454	B4GALNT1	
ENST00000341156	ENSG00000135454	B4GALNT1	
ENST00000441989	ENSG00000241685	ARPC1A	
ENST00000484662	ENSG00000162616	DNAJB4	
ENST00000452361	ENSG00000274049	INO80B-WBP1	filled
ENST00000441673	ENSG00000274049	INO80B-WBP1	filled
ENST00000484946	ENSG00000189077	TMEM120A	
ENST00000431982	ENSG00000077800	FKBP6	
ENST00000413573	ENSG00000077800	FKBP6	
ENST00000445032	ENSG00000077800	FKBP6	
ENST00000252037	ENSG00000077800	FKBP6	
ENST00000587934	ENSG00000123159	GIPC1	filled
ENST00000256759	ENSG00000134363	FST	
ENST00000308064	ENSG00000175264	CHST1	filled
ENST00000623624	ENSG00000279735	AC124312.1	
ENST00000567662	ENSG00000175318	GRAMD2	
ENST00000357721	ENSG00000139160	METTL20	
ENST00000394718	ENSG00000179841	AKAP5	
ENST00000517878	ENSG00000022567	SLC45A4	filled
ENST00000588084	ENSG00000105325	FZR1	
ENST00000314845	ENSG00000110497	AMBRA1	filled
ENST00000533727	ENSG00000110497	AMBRA1	filled
ENST00000534300	ENSG00000110497	AMBRA1	filled
ENST00000458649	ENSG00000110497	AMBRA1	filled
ENST00000528950	ENSG00000110497	AMBRA1	filled
ENST00000529553	ENSG00000110497	AMBRA1	filled
ENST00000529963	ENSG00000110497	AMBRA1	filled
ENST00000529558	ENSG00000110497	AMBRA1	
ENST00000531542	ENSG00000110497	AMBRA1	filled
ENST00000526606	ENSG00000110497	AMBRA1	filled

ENST00000612728	ENSG00000273611	ZNHIT3	
ENST00000371236	ENSG00000173406	DAB1	filled
ENST00000485760	ENSG00000173406	DAB1	filled
ENST00000371230	ENSG00000173406	DAB1	filled
ENST00000549548	ENSG00000135407	AVIL	filled
ENST00000549994	ENSG00000135407	AVIL	
ENST00000559529	ENSG00000106852	LHX6	
ENST00000612291	ENSG00000276780	NLRP10	
ENST00000480821	ENSG00000125355	TMEM255A	filled
ENST00000521776	ENSG00000253797	UTP14C	
ENST00000469649	ENSG00000065485	PDIA5	
ENST00000367167	ENSG00000174529	TMEM81	
ENST00000466232	ENSG00000005486	RHBDD2	filled
ENST00000247182	ENSG00000126778	SIX1	
ENST00000554986	ENSG00000126778	SIX1	
ENST00000555955	ENSG00000126778	SIX1	
ENST00000553535	ENSG00000126778	SIX1	
ENST00000555627	ENSG00000126778	SIX1	
ENST00000604324	ENSG00000105392	CRX	
ENST00000270112	ENSG00000142149	HUNK	filled
ENST00000618338	ENSG00000274359	NDUFA3	
ENST00000508965	ENSG00000152784	PRDM8	
ENST00000504452	ENSG00000152784	PRDM8	filled
ENST00000508061	ENSG00000152784	PRDM8	
ENST00000515013	ENSG00000152784	PRDM8	
ENST00000507025	ENSG00000152784	PRDM8	
ENST00000415738	ENSG00000152784	PRDM8	filled
ENST00000339711	ENSG00000152784	PRDM8	filled
ENST00000519959	ENSG00000158856	DMTN	filled
ENST00000522340	ENSG00000158856	DMTN	
ENST00000519333	ENSG00000158856	DMTN	
ENST00000574448	ENSG00000103316	CRYM	filled
ENST00000570401	ENSG00000103316	CRYM	filled
ENST00000603974	ENSG00000270011	ZNF559-ZNF177	
ENST00000522269	ENSG00000145863	GABRA6	
ENST00000518888	ENSG00000145863	GABRA6	
ENST00000589171	ENSG00000130165	ELOF1	
ENST00000591674	ENSG00000130165	ELOF1	
ENST00000586120	ENSG00000130165	ELOF1	
ENST00000591912	ENSG00000130165	ELOF1	
ENST00000467035	ENSG00000105971	CAV2	filled
ENST00000484871	ENSG00000105971	CAV2	filled
ENST00000472470	ENSG00000105971	CAV2	filled
ENST00000498493	ENSG00000105971	CAV2	filled
ENST00000533285	ENSG00000149269	PAK1	filled
ENST00000396625	ENSG00000081052	COL4A4	filled
ENST00000427043	ENSG00000105141	CASP14	filled
ENST00000215906	ENSG00000128203	ASPHD2	
ENST00000568049	ENSG00000185716	C16orf52	
ENST00000505905	ENSG00000189308	LIN54	
ENST00000581073	ENSG00000141668	CBLN2	filled
ENST00000580889	ENSG00000141668	CBLN2	filled
ENST00000583570	ENSG00000141668	CBLN2	
ENST00000287912	ENSG00000184863	RBM33	filled
ENST00000392759	ENSG00000184863	RBM33	filled
ENST00000502824	ENSG00000133401	PDZD2	filled
ENST00000513910	ENSG00000133401	PDZD2	filled
ENST00000519409	ENSG00000104450	SPAG1	filled
ENST00000453225	ENSG00000010270	STARD3NL	filled
ENST00000560104	ENSG00000259571	BLID	
ENST00000514258	ENSG00000145708	CRHBP	
ENST00000523993	ENSG00000153310	FAM49B	filled
ENST00000396048	ENSG00000186446	ZNF501	
ENST00000620116	ENSG00000186446	ZNF501	
ENST00000420034	ENSG00000167123	CERCAM	filled
ENST00000372842	ENSG00000167123	CERCAM	filled
ENST00000447915	ENSG00000167123	CERCAM	filled
ENST00000532914	ENSG00000179431	FJX1	
ENST00000609271	ENSG00000007908	SELE	
ENST00000330168	ENSG00000248751	RP1-130H16.18	
ENST00000434987	ENSG00000248751	RP1-130H16.18	
ENST00000434291	ENSG00000248751	RP1-130H16.18	
ENST00000418047	ENSG00000248751	RP1-130H16.18	
ENST00000447976	ENSG00000248751	RP1-130H16.18	
ENST00000346213	ENSG00000108774	RAB5C	
ENST00000551338	ENSG00000108774	RAB5C	filled
ENST00000553521	ENSG00000100650	SRSF5	filled
ENST00000357816	ENSG00000165566	AMER2	
ENST00000489552	ENSG00000146966	DENND2A	filled
ENST00000356860	ENSG00000153956	CACNA2D1	filled
ENST00000464354	ENSG00000153956	CACNA2D1	
ENST00000475237	ENSG00000153956	CACNA2D1	
ENST00000492734	ENSG00000153956	CACNA2D1	
ENST00000423588	ENSG00000153956	CACNA2D1	filled
ENST00000461275	ENSG00000153956	CACNA2D1	filled
ENST00000484706	ENSG00000153956	CACNA2D1	filled
ENST00000356253	ENSG00000153956	CACNA2D1	filled
ENST00000615520	ENSG00000143355	LHX9	
ENST00000367390	ENSG00000143355	LHX9	filled
ENST00000367387	ENSG00000143355	LHX9	filled
ENST00000517490	ENSG00000085719	CPNE3	filled
ENST00000621783	ENSG00000085719	CPNE3	filled
ENST00000522240	ENSG00000085719	CPNE3	filled
ENST00000467442	ENSG00000250588	IQCJ-SCHIP1	filled
ENST00000476809	ENSG00000250588	IQCJ-SCHIP1	filled
ENST00000485419	ENSG00000250588	IQCJ-SCHIP1	filled
ENST00000483486	ENSG00000250588	IQCJ-SCHIP1	filled
ENST00000488898	ENSG00000250588	IQCJ-SCHIP1	filled
ENST00000337808	ENSG00000250588	IQCJ-SCHIP1	filled
ENST00000412423	ENSG00000250588	IQCJ-SCHIP1	filled
ENST00000527095	ENSG00000250588	IQCJ-SCHIP1	filled
ENST00000467377	ENSG00000250588	IQCJ-SCHIP1	
ENST00000421177	ENSG00000168824	D4S234E	filled
ENST00000378993	ENSG00000169306	IL1RAPL1	filled
ENST00000486622	ENSG00000137393	RNF144B	
ENST00000453332	ENSG00000091138	SLC26A3	
ENST00000602662	ENSG00000270136	MINOS1-NBL1	filled
ENST00000602384	ENSG00000270136	MINOS1-NBL1	filled
ENST00000602293	ENSG00000270136	MINOS1-NBL1	filled
ENST00000602450	ENSG00000270136	MINOS1-NBL1	filled
ENST00000500968	ENSG00000247746	USP51	filled
ENST00000432796	ENSG00000128652	HOXD3	
ENST00000489174	ENSG00000119147	C2orf40	
ENST00000381323	ENSG00000197872	FAM49A	filled
ENST00000406434	ENSG00000197872	FAM49A	filled
ENST00000445605	ENSG00000197872	FAM49A	filled
ENST00000451689	ENSG00000197872	FAM49A	filled
ENST00000319074	ENSG00000179933	C14orf119	
ENST00000371564	ENSG00000101096	NFATC2	filled
ENST00000396009	ENSG00000101096	NFATC2	filled

ENST00000609943	ENSG00000101096	NFATC2	filled
ENST00000610033	ENSG00000101096	NFATC2	filled
ENST00000414705	ENSG00000101096	NFATC2	filled
ENST00000609507	ENSG00000101096	NFATC2	filled
ENST00000496054	ENSG00000101096	NFATC2	
ENST00000439593	ENSG00000159147	DONSON	
ENST00000433395	ENSG00000249624	AP000295.9	
ENST00000432231	ENSG00000249624	AP000295.9	
ENST00000328306	ENSG00000065923	SLC9A7	filled
ENST00000427004	ENSG00000112511	PHF1	
ENST00000526960	ENSG00000184007	PTP4A2	filled
ENST00000449679	ENSG00000226264	HLA-DMB	filled
ENST00000507237	ENSG00000269556	TMEM185A	
ENST00000517359	ENSG00000269556	TMEM185A	
ENST00000511776	ENSG00000269556	TMEM185A	
ENST00000377047	ENSG00000183098	GPC6	filled
ENST00000514898	ENSG00000018189	RUFY3	
ENST00000464392	ENSG00000204257	HLA-DMA	
ENST00000368921	ENSG00000213190	MLLT11	
ENST00000599883	ENSG00000105127	AKAP8	
ENST00000375006	ENSG00000158571	PFKFB1	
ENST00000545676	ENSG00000158571	PFKFB1	
ENST00000374992	ENSG00000158571	PFKFB1	
ENST00000342745	ENSG00000100647	KIAA0247	filled
ENST00000556993	ENSG00000100647	KIAA0247	filled
ENST00000619034	ENSG00000275618	BFAR	filled
ENST00000425699	ENSG00000188613	NANOS1	
ENST00000340087	ENSG00000188613	NANOS1	
ENST00000490457	ENSG00000172888	ZNF621	
ENST00000371839	ENSG00000186094	AGBL4	filled
ENST00000371838	ENSG00000186094	AGBL4	filled
ENST00000439374	ENSG00000197622	CDC42SE1	
ENST00000409852	ENSG00000163273	NPPC	filled
ENST00000522112	ENSG00000147676	MAL2	filled
ENST00000571285	ENSG00000140987	ZSCAN32	
ENST00000487286	ENSG00000138032	PPM1B	
ENST00000485769	ENSG00000185630	PBX1	
ENST00000624396	ENSG00000279983	CH17-140K24.8	
ENST00000616205	ENSG00000277069	RXFP3	
ENST00000417834	ENSG00000226882	GNL1	
ENST00000362005	ENSG00000137948	BRDT	
ENST00000370389	ENSG00000137948	BRDT	
ENST00000423434	ENSG00000137948	BRDT	
ENST00000440509	ENSG00000137948	BRDT	
ENST00000427104	ENSG00000137948	BRDT	
ENST00000448194	ENSG00000137948	BRDT	
ENST00000426141	ENSG00000137948	BRDT	
ENST00000450792	ENSG00000137948	BRDT	
ENST00000548992	ENSG00000137948	BRDT	
ENST00000552654	ENSG00000137948	BRDT	
ENST00000394530	ENSG00000137948	BRDT	
ENST00000399546	ENSG00000137948	BRDT	
ENST00000616094	ENSG00000157613	CREB3L1	filled
ENST00000371837	ENSG00000107798	LIPA	filled
ENST00000489359	ENSG00000107798	LIPA	
ENST00000453079	ENSG00000100266	PACSIN2	
ENST00000340736	ENSG00000072071	LPHN1	
ENST00000361434	ENSG00000072071	LPHN1	
ENST00000588320	ENSG00000108788	MLX	filled
ENST00000305963	ENSG00000169446	MMGT1	

ENST00000533357	ENSG00000158887	MPZ	
ENST00000491222	ENSG00000158887	MPZ	
ENST00000526189	ENSG00000158887	MPZ	
ENST00000476410	ENSG00000158887	MPZ	
ENST00000488271	ENSG00000158887	MPZ	
ENST00000331837	ENSG00000182636	NDN	
ENST00000569102	ENSG00000167191	GPRC5B	
ENST00000566822	ENSG00000167191	GPRC5B	
ENST00000550437	ENSG00000258052	AC025263.3	filled
ENST00000448993	ENSG00000182600	C2orf82	
ENST00000409533	ENSG00000182600	C2orf82	
ENST00000613024	ENSG00000277303	C17orf96	
ENST00000393461	ENSG00000158955	WNT9B	filled
ENST00000503191	ENSG00000104723	TUSC3	filled
ENST00000475785	ENSG00000204271	SPIN3	
ENST00000478405	ENSG00000204271	SPIN3	
ENST00000598310	ENSG00000164283	ESM1	
ENST00000601836	ENSG00000164283	ESM1	
ENST00000407714	ENSG00000178623	GPR35	
ENST00000373278	ENSG00000124721	DNAH8	filled
ENST00000511608	ENSG00000164344	KLKB1	filled
ENST00000251691	ENSG00000112379	KIAA1244	filled
ENST00000510806	ENSG00000154447	SH3RF1	
ENST00000483395	ENSG00000175084	DES	
ENST00000567698	ENSG00000171241	SHCBP1	
ENST00000564272	ENSG00000171241	SHCBP1	
ENST00000555989	ENSG00000197555	SIPA1L1	filled
ENST00000554126	ENSG00000197555	SIPA1L1	filled
ENST00000556780	ENSG00000197555	SIPA1L1	filled
ENST00000554362	ENSG00000197555	SIPA1L1	filled
ENST00000555652	ENSG00000197555	SIPA1L1	filled
ENST00000557151	ENSG00000197555	SIPA1L1	filled
ENST00000518471	ENSG00000197217	ENTPD4	
ENST00000356206	ENSG00000197217	ENTPD4	
ENST00000521321	ENSG00000197217	ENTPD4	
ENST00000522255	ENSG00000197217	ENTPD4	filled
ENST00000367661	ENSG00000116183	PAPPA2	filled
ENST00000367662	ENSG00000116183	PAPPA2	filled
ENST00000266643	ENSG00000139266	9-Mar	
ENST00000374550	ENSG00000142676	RPL11	
ENST00000552771	ENSG00000166783	KIAA0430	
ENST00000408967	ENSG00000221870	TMEM257	
ENST00000591168	ENSG00000159905	ZNF221	filled
ENST00000587682	ENSG00000159905	ZNF221	filled
ENST00000251269	ENSG00000159905	ZNF221	filled
ENST00000274024	ENSG00000145384	FABP2	
ENST00000222567	ENSG00000105849	TWISTNB	
ENST00000567514	ENSG00000225526	MKRN2OS	filled
ENST00000561645	ENSG00000225526	MKRN2OS	
ENST00000564146	ENSG00000225526	MKRN2OS	
ENST00000447550	ENSG00000225526	MKRN2OS	
ENST00000316398	ENSG00000179071	CCDC89	
ENST00000616721	ENSG00000275395	FCGBP	
ENST00000524750	ENSG00000085117	CD82	filled
ENST00000395065	ENSG00000147206	NXF3	
ENST00000378933	ENSG00000157625	TAB3	filled
ENST00000378932	ENSG00000157625	TAB3	filled
ENST00000467136	ENSG00000157625	TAB3	filled
ENST00000288422	ENSG00000157625	TAB3	
ENST00000322940	ENSG00000119328	FAM206A	

ENST00000374624	ENSG00000119328	FAM206A	filled
ENST00000257904	ENSG00000135446	CDK4	filled
ENST00000552862	ENSG00000135446	CDK4	
ENST00000613276	ENSG00000273642	NDUFA3	
ENST00000583972	ENSG00000109111	SUPT6H	filled
ENST00000355387	ENSG00000187147	RNF220	filled
ENST00000361799	ENSG00000187147	RNF220	filled
ENST00000487332	ENSG00000187147	RNF220	
ENST00000470498	ENSG00000187147	RNF220	
ENST00000372247	ENSG00000187147	RNF220	filled
ENST00000443020	ENSG00000187147	RNF220	filled
ENST00000272438	ENSG00000144043	TEX261	
ENST00000478068	ENSG00000144043	TEX261	
ENST00000374266	ENSG00000142684	ZNF593	
ENST00000270812	ENSG00000142684	ZNF593	
ENST00000587186	ENSG00000180448	HMHA1	
ENST00000591293	ENSG00000180448	HMHA1	
ENST00000586033	ENSG00000180448	HMHA1	
ENST00000399933	ENSG00000183354	KIAA2026	filled
ENST00000381461	ENSG00000183354	KIAA2026	filled
ENST00000540714	ENSG00000183354	KIAA2026	
ENST00000513355	ENSG00000183354	KIAA2026	filled
ENST00000581347	ENSG00000206432	TMEM200C	filled
ENST00000383490	ENSG00000206432	TMEM200C	
ENST00000573500	ENSG00000141179	PCTP	filled
ENST00000394095	ENSG00000138468	SENP7	filled
ENST00000394091	ENSG00000138468	SENP7	filled
ENST00000394094	ENSG00000138468	SENP7	filled
ENST00000314261	ENSG00000138468	SENP7	filled
ENST00000348610	ENSG00000138468	SENP7	filled
ENST00000256852	ENSG00000134438	RAX	
ENST00000334889	ENSG00000134438	RAX	
ENST00000555288	ENSG00000134438	RAX	filled
ENST00000522369	ENSG00000034677	RNF19A	
ENST00000355689	ENSG00000197557	TTC30A	
ENST00000396249	ENSG00000092208	GEMIN2	
ENST00000412033	ENSG00000092208	GEMIN2	
ENST00000250379	ENSG00000092208	GEMIN2	
ENST00000531684	ENSG00000092208	GEMIN2	
ENST00000534684	ENSG00000092208	GEMIN2	
ENST00000587473	ENSG00000130382	MLLT1	
ENST00000495552	ENSG00000248592	TMEM110-MUSTN1	
ENST00000586725	ENSG00000141429	GALNT1	
ENST00000589189	ENSG00000141429	GALNT1	filled
ENST00000577646	ENSG00000131759	RARA	
ENST00000254066	ENSG00000131759	RARA	filled
ENST00000225726	ENSG00000108588	CCDC47	filled
ENST00000403162	ENSG00000108588	CCDC47	filled
ENST00000582252	ENSG00000108588	CCDC47	filled
ENST00000543114	ENSG00000151067	CACNA1C	filled
ENST00000335762	ENSG00000151067	CACNA1C	filled
ENST00000399655	ENSG00000151067	CACNA1C	filled
ENST00000480911	ENSG00000151067	CACNA1C	filled
ENST00000399595	ENSG00000151067	CACNA1C	filled
ENST00000399644	ENSG00000151067	CACNA1C	filled
ENST00000399638	ENSG00000151067	CACNA1C	filled
ENST00000399597	ENSG00000151067	CACNA1C	filled
ENST00000399621	ENSG00000151067	CACNA1C	filled
ENST00000399637	ENSG00000151067	CACNA1C	filled
ENST00000399591	ENSG00000151067	CACNA1C	filled

ENST00000399641	ENSG00000151067	CACNA1C	filled
ENST00000347598	ENSG00000151067	CACNA1C	filled
ENST00000399606	ENSG00000151067	CACNA1C	filled
ENST00000399601	ENSG00000151067	CACNA1C	filled
ENST00000344100	ENSG00000151067	CACNA1C	filled
ENST00000399629	ENSG00000151067	CACNA1C	filled
ENST00000327702	ENSG00000151067	CACNA1C	filled
ENST00000399649	ENSG00000151067	CACNA1C	filled
ENST00000402845	ENSG00000151067	CACNA1C	filled
ENST00000399603	ENSG00000151067	CACNA1C	filled
ENST00000399634	ENSG00000151067	CACNA1C	filled
ENST00000399617	ENSG00000151067	CACNA1C	filled
ENST00000406454	ENSG00000151067	CACNA1C	filled
ENST00000471733	ENSG00000075711	DLG1	filled
ENST00000562522	ENSG00000140678	ITGAX	filled
ENST00000572274	ENSG00000103426	CORO7-PAM16	
ENST00000574426	ENSG00000132361	CLUH	
ENST00000576885	ENSG00000132361	CLUH	
ENST00000435359	ENSG00000132361	CLUH	
ENST00000519004	ENSG00000070756	PABPC1	filled
ENST00000519596	ENSG00000070756	PABPC1	filled
ENST00000521067	ENSG00000070756	PABPC1	
ENST00000520804	ENSG00000070756	PABPC1	
ENST00000548741	ENSG00000111596	CNOT2	filled
ENST00000547347	ENSG00000179088	C12orf42	filled
ENST00000547470	ENSG00000179088	C12orf42	
ENST00000549927	ENSG00000179088	C12orf42	
ENST00000344147	ENSG00000121766	ZCCHC17	
ENST00000618216	ENSG00000121766	ZCCHC17	
ENST00000616393	ENSG00000121766	ZCCHC17	
ENST00000422613	ENSG00000121766	ZCCHC17	
ENST00000439380	ENSG00000138356	AOX1	
ENST00000360605	ENSG00000105176	URI1	
ENST00000452474	ENSG00000118260	CREB1	
ENST00000414681	ENSG00000118260	CREB1	
ENST00000451164	ENSG00000118260	CREB1	
ENST00000507188	ENSG00000186952	TMEM232	filled
ENST00000508571	ENSG00000186952	TMEM232	filled
ENST00000305428	ENSG00000169330	KIAA1024	
ENST00000559272	ENSG00000169330	KIAA1024	
ENST00000615705	ENSG00000276887	TTYH1	
ENST00000621480	ENSG00000276887	TTYH1	
ENST00000611778	ENSG00000276887	TTYH1	
ENST00000425696	ENSG00000102054	RBBP7	
ENST00000337227	ENSG00000089050	RBBP9	
ENST00000520368	ENSG00000156787	TBC1D31	filled
ENST00000513794	ENSG00000113595	TRIM23	
ENST00000356641	ENSG00000196914	ARHGEF12	
ENST00000397843	ENSG00000196914	ARHGEF12	
ENST00000520552	ENSG00000186106	ANKRD46	
ENST00000561069	ENSG00000034053	APBA2	filled
ENST00000558402	ENSG00000034053	APBA2	filled
ENST00000558330	ENSG00000034053	APBA2	filled
ENST00000560283	ENSG00000034053	APBA2	
ENST00000558259	ENSG00000034053	APBA2	filled
ENST00000411764	ENSG00000034053	APBA2	filled
ENST00000327892	ENSG00000196230	TUBB	
ENST00000330914	ENSG00000196230	TUBB	
ENST00000550331	ENSG00000136098	NEK3	
ENST00000598523	ENSG00000268089	GABRQ	

ENST00000264463	ENSG00000040731	CDH10	filled
ENST00000510477	ENSG00000040731	CDH10	filled
ENST00000564123	ENSG00000125124	BBS2	
ENST00000564459	ENSG00000125124	BBS2	filled
ENST00000372156	ENSG00000108219	TSPAN14	
ENST00000474267	ENSG00000114251	WNT5A	
ENST00000493406	ENSG00000114251	WNT5A	
ENST00000624674	ENSG00000114251	WNT5A	
ENST00000264634	ENSG00000114251	WNT5A	
ENST00000468418	ENSG00000278500	AC009336.19	
ENST00000373359	ENSG00000186675	MAGEE2	
ENST00000505691	ENSG00000125898	FAM110A	
ENST00000589807	ENSG00000267120	AD000671.6	
ENST00000518120	ENSG00000197226	TBC1D9B	
ENST00000489492	ENSG00000138002	IFT172	
ENST00000511842	ENSG00000138002	IFT172	
ENST00000359466	ENSG00000138002	IFT172	
ENST00000416524	ENSG00000138002	IFT172	
ENST00000476264	ENSG00000138002	IFT172	
ENST00000475476	ENSG00000138002	IFT172	
ENST00000528449	ENSG00000084628	NKAIN1	
ENST00000394227	ENSG00000213445	SIPA1	filled
ENST00000561272	ENSG00000100564	PIGH	
ENST00000453504	ENSG00000236418	HLA-DQA1	filled
ENST00000457246	ENSG00000236418	HLA-DQA1	filled
ENST00000442315	ENSG00000236418	HLA-DQA1	filled
ENST00000477036	ENSG00000236418	HLA-DQA1	filled
ENST00000450960	ENSG00000236418	HLA-DQA1	filled
ENST00000461508	ENSG00000236418	HLA-DQA1	filled
ENST00000264657	ENSG00000168610	STAT3	filled
ENST00000585517	ENSG00000168610	STAT3	filled
ENST00000462286	ENSG00000168610	STAT3	filled
ENST00000389272	ENSG00000168610	STAT3	filled
ENST00000588969	ENSG00000168610	STAT3	filled
ENST00000404395	ENSG00000168610	STAT3	filled
ENST00000588065	ENSG00000168610	STAT3	filled
ENST00000306346	ENSG00000186496	ZNF396	
ENST00000589332	ENSG00000186496	ZNF396	
ENST00000586585	ENSG00000186496	ZNF396	
ENST00000586687	ENSG00000186496	ZNF396	
ENST00000452949	ENSG00000224290	APOM	
ENST00000416324	ENSG00000224290	APOM	
ENST00000464522	ENSG00000110436	SLC1A2	
ENST00000533058	ENSG00000196655	TRAPPC4	filled
ENST00000399300	ENSG00000183808	RBM12B	
ENST00000520961	ENSG00000183808	RBM12B	
ENST00000462137	ENSG00000114383	TUSC2	
ENST00000507810	ENSG00000129595	EPB41L4A	
ENST00000593742	ENSG00000109610	SOD3	
ENST00000436627	ENSG00000230669	PSMB8	filled
ENST00000268296	ENSG00000140678	ITGAX	filled
ENST00000366810	ENSG00000185155	MIXL1	
ENST00000557734	ENSG00000185155	MIXL1	
ENST00000335350	ENSG00000107731	UNC5B	filled
ENST00000373192	ENSG00000107731	UNC5B	filled
ENST00000334530	ENSG00000168476	REEP4	
ENST00000306306	ENSG00000168476	REEP4	
ENST00000518664	ENSG00000168476	REEP4	
ENST00000519074	ENSG00000168476	REEP4	
ENST00000282388	ENSG00000152518	ZFP36L2	filled

ENST00000540667	ENSG00000159335	PTMS	
ENST00000389462	ENSG00000159335	PTMS	
ENST00000540874	ENSG00000159335	PTMS	
ENST00000309083	ENSG00000159335	PTMS	
ENST00000540828	ENSG00000159335	PTMS	
ENST00000538057	ENSG00000159335	PTMS	
ENST00000619580	ENSG00000159335	PTMS	
ENST00000478428	ENSG00000008226	DLEC1	
ENST00000532047	ENSG00000152219	ARL14EP	
ENST00000537935	ENSG00000183269	OR52E8	
ENST00000429501	ENSG00000135926	TMBIM1	
ENST00000451181	ENSG00000135926	TMBIM1	
ENST00000479789	ENSG00000006453	BAIAP2L1	
ENST00000595101	ENSG00000053501	USE1	filled
ENST00000557006	ENSG00000198133	TMEM229B	filled
ENST00000557779	ENSG00000198133	TMEM229B	filled
ENST00000357461	ENSG00000198133	TMEM229B	filled
ENST00000614443	ENSG00000277161	PIGW	
ENST00000620233	ENSG00000277161	PIGW	
ENST00000593511	ENSG00000171017	LRRC8E	filled
ENST00000415814	ENSG00000131374	TBC1D5	filled
ENST00000452492	ENSG00000131374	TBC1D5	filled
ENST00000412981	ENSG00000131374	TBC1D5	filled
ENST00000428355	ENSG00000131374	TBC1D5	filled
ENST00000425944	ENSG00000131374	TBC1D5	filled
ENST00000430169	ENSG00000131374	TBC1D5	filled
ENST00000445294	ENSG00000131374	TBC1D5	filled
ENST00000414349	ENSG00000131374	TBC1D5	filled
ENST00000443499	ENSG00000131374	TBC1D5	filled
ENST00000443386	ENSG00000131374	TBC1D5	filled
ENST00000497531	ENSG00000131374	TBC1D5	filled
ENST00000444756	ENSG00000131374	TBC1D5	filled
ENST00000444471	ENSG00000131374	TBC1D5	filled
ENST00000481396	ENSG00000131374	TBC1D5	filled
ENST00000423331	ENSG00000131374	TBC1D5	
ENST00000485432	ENSG00000131374	TBC1D5	
ENST00000471679	ENSG00000131374	TBC1D5	
ENST00000579638	ENSG00000154845	PPP4R1	
ENST00000583903	ENSG00000154845	PPP4R1	
ENST00000579415	ENSG00000154845	PPP4R1	
ENST00000580745	ENSG00000154845	PPP4R1	
ENST00000582933	ENSG00000154845	PPP4R1	
ENST00000370819	ENSG00000124749	COL21A1	filled
ENST00000530348	ENSG00000049449	RCN1	filled
ENST00000532942	ENSG00000049449	RCN1	filled
ENST00000506388	ENSG00000049449	RCN1	
ENST00000054950	ENSG00000049449	RCN1	filled
ENST00000532721	ENSG00000049449	RCN1	
ENST00000531345	ENSG00000049449	RCN1	filled
ENST00000528630	ENSG00000049449	RCN1	
ENST00000317221	ENSG00000188321	ZNF559	
ENST00000592896	ENSG00000188321	ZNF559	
ENST00000605750	ENSG00000188321	ZNF559	
ENST00000461084	ENSG00000114648	KLHL18	filled
ENST00000491575	ENSG00000237495	LY6G5C	
ENST00000478907	ENSG00000237495	LY6G5C	
ENST00000432980	ENSG00000237495	LY6G5C	
ENST00000534526	ENSG00000139132	FGD4	filled
ENST00000434349	ENSG00000128159	TUBGCP6	
ENST00000429960	ENSG00000168384	HLA-DPA1	

ENST00000558604	ENSG00000157766	ACAN	
ENST00000529257	ENSG00000076706	MCAM	
ENST00000529295	ENSG00000076706	MCAM	
ENST00000534522	ENSG00000076706	MCAM	
ENST00000602837	ENSG00000170035	UBE2E3	
ENST00000602970	ENSG00000170035	UBE2E3	
ENST00000419471	ENSG00000235155	B3GALT4	filled
ENST00000522780	ENSG00000165071	TMEM71	
ENST00000623534	ENSG00000278780	ZNF430	
ENST00000600810	ENSG00000269237	CTD-2561J22.3	filled
ENST00000553781	ENSG00000258643	BCL2L2-PABPN1	
ENST00000556100	ENSG00000258643	BCL2L2-PABPN1	
ENST00000557008	ENSG00000258643	BCL2L2-PABPN1	
ENST00000456258	ENSG00000228284	HLA-DQA1	filled
ENST00000450614	ENSG00000228284	HLA-DQA1	filled
ENST00000441297	ENSG00000228284	HLA-DQA1	filled
ENST00000484643	ENSG00000228284	HLA-DQA1	filled
ENST00000614772	ENSG00000228284	HLA-DQA1	filled
ENST00000585836	ENSG00000125740	FOSB	
ENST00000417353	ENSG00000125740	FOSB	
ENST00000353609	ENSG00000125740	FOSB	
ENST00000591858	ENSG00000125740	FOSB	
ENST00000443841	ENSG00000125740	FOSB	
ENST00000592811	ENSG00000125740	FOSB	
ENST00000586615	ENSG00000125740	FOSB	
ENST00000615753	ENSG00000125740	FOSB	
ENST00000503446	ENSG00000124875	CXCL6	
ENST00000505286	ENSG00000168818	STX18	filled
ENST00000306200	ENSG00000168818	STX18	filled
ENST00000512780	ENSG00000168818	STX18	
ENST00000529466	ENSG00000255359	CCDC179	
ENST00000454603	ENSG00000128683	GAD1	
ENST00000618371	ENSG00000242419	PCDHGC4	filled
ENST00000306593	ENSG00000242419	PCDHGC4	filled
ENST00000404924	ENSG00000173960	UBXN2A	filled
ENST00000623174	ENSG00000278899	AL358852.1	
ENST00000544117	ENSG00000167528	ZNF641	
ENST00000301042	ENSG00000167528	ZNF641	
ENST00000441080	ENSG00000101132	PFDN4	
ENST00000418342	ENSG00000169641	LUZP1	
ENST00000394264	ENSG00000187866	FAM122A	
ENST00000462691	ENSG00000197021	CXorf40B	
ENST00000375654	ENSG00000204390	HSPA1L	
ENST00000357874	ENSG00000111554	MDM1	
ENST00000265382	ENSG00000107242	PIP5K1B	filled
ENST00000472907	ENSG00000107242	PIP5K1B	filled
ENST00000474356	ENSG00000107242	PIP5K1B	
ENST00000377284	ENSG00000107242	PIP5K1B	filled
ENST00000437200	ENSG00000107242	PIP5K1B	filled
ENST00000440050	ENSG00000107242	PIP5K1B	filled
ENST00000478500	ENSG00000107242	PIP5K1B	filled
ENST00000541509	ENSG00000107242	PIP5K1B	filled
ENST00000289292	ENSG00000158352	SHROOM4	filled
ENST00000484922	ENSG00000158352	SHROOM4	
ENST00000376020	ENSG00000158352	SHROOM4	filled
ENST00000475838	ENSG00000183778	B3GALT5	
ENST00000613555	ENSG00000276231	PIK3R6	filled
ENST00000611951	ENSG00000276231	PIK3R6	filled
ENST00000619866	ENSG00000276231	PIK3R6	filled
ENST00000586056	ENSG00000141469	SLC14A1	

ENST00000589891	ENSG00000141469	SLC14A1	
ENST00000590246	ENSG00000141469	SLC14A1	
ENST00000461988	ENSG00000127948	POR	filled
ENST00000449920	ENSG00000127948	POR	filled
ENST00000585080	ENSG00000178927	C17orf62	
ENST00000577888	ENSG00000178927	C17orf62	
ENST00000577696	ENSG00000178927	C17orf62	
ENST00000577471	ENSG00000178927	C17orf62	
ENST00000582545	ENSG00000178927	C17orf62	
ENST00000316356	ENSG00000137501	SYTL2	filled
ENST00000527523	ENSG00000137501	SYTL2	filled
ENST00000389960	ENSG00000137501	SYTL2	filled
ENST00000549510	ENSG00000120833	SOCS2	
ENST00000594797	ENSG00000229833	PET100	
ENST00000454337	ENSG00000261732	LA16c-431H6.6	filled
ENST00000435827	ENSG00000090273	NUDC	
ENST00000470481	ENSG00000166295	ANAPC16	filled
ENST00000376663	ENSG00000168283	BMI1	
ENST00000544837	ENSG00000203963	C1orf141	
ENST00000603691	ENSG00000203963	C1orf141	
ENST00000472449	ENSG00000142621	FHAD1	filled
ENST00000587091	ENSG00000147100	SLC16A2	
ENST00000377205	ENSG00000173614	NMNAT1	filled
ENST00000492735	ENSG00000173614	NMNAT1	filled
ENST00000496751	ENSG00000173614	NMNAT1	filled
ENST00000221700	ENSG00000186115	CYP4F2	filled
ENST00000586927	ENSG00000186115	CYP4F2	
ENST00000608168	ENSG00000186115	CYP4F2	
ENST00000539764	ENSG00000175567	UCP2	
ENST00000595900	ENSG00000127511	SIN3B	filled
ENST00000602204	ENSG00000127511	SIN3B	
ENST00000533310	ENSG00000171067	C11orf24	filled
ENST00000440702	ENSG00000116199	FAM20B	filled
ENST00000617282	ENSG00000278365	NDUFA3	
ENST00000472185	ENSG00000147140	NONO	
ENST00000497650	ENSG00000179562	GCC1	filled
ENST00000474330	ENSG00000128536	CDHR3	
ENST00000470188	ENSG00000128536	CDHR3	filled
ENST00000488386	ENSG00000128536	CDHR3	filled
ENST00000326279	ENSG00000131914	LIN28A	
ENST00000254231	ENSG00000131914	LIN28A	
ENST00000379589	ENSG00000183722	LHFP	filled
ENST00000564125	ENSG00000158865	SLC5A11	
ENST00000569520	ENSG00000158865	SLC5A11	
ENST00000561646	ENSG00000087263	OGFOD1	
ENST00000461548	ENSG00000256566	RP4-734P14.4	
ENST00000428683	ENSG00000166526	ZNF3	filled
ENST00000572022	ENSG00000179593	ALOX15B	
ENST00000571240	ENSG00000179593	ALOX15B	
ENST00000573359	ENSG00000179593	ALOX15B	
ENST00000380173	ENSG00000179593	ALOX15B	
ENST00000468472	ENSG00000243696	RP5-966M1.6	filled
ENST00000527924	ENSG00000213906	LTB4R2	filled
ENST00000528054	ENSG00000213906	LTB4R2	
ENST00000607161	ENSG00000268439	RP3-461F17.3	filled
ENST00000261406	ENSG00000268439	RP3-461F17.3	filled
ENST00000546139	ENSG00000173264	GPR137	
ENST00000538244	ENSG00000173264	GPR137	
ENST00000493223	ENSG00000088826	SMOX	filled
ENST00000487159	ENSG00000182606	TRAK1	filled

ENST00000525924	ENSG00000014138	POLA2	
ENST00000521871	ENSG00000163013	FBXO41	
ENST00000520530	ENSG00000163013	FBXO41	
ENST00000520186	ENSG00000163013	FBXO41	
ENST00000295133	ENSG00000163013	FBXO41	
ENST00000565844	ENSG00000103550	KNOP1	
ENST00000506417	ENSG00000154124	OTULIN	
ENST00000496027	ENSG00000244607	CCDC13	filled
ENST00000591855	ENSG00000161265	U2AF1L4	
ENST00000594792	ENSG00000161265	U2AF1L4	
ENST00000378975	ENSG00000161265	U2AF1L4	
ENST00000592913	ENSG00000161265	U2AF1L4	
ENST00000585771	ENSG00000161265	U2AF1L4	
ENST00000587886	ENSG00000161265	U2AF1L4	
ENST00000412391	ENSG00000161265	U2AF1L4	
ENST00000330689	ENSG00000196981	WDR5B	
ENST00000293471	ENSG00000176024	ZNF613	
ENST00000391794	ENSG00000176024	ZNF613	
ENST00000599683	ENSG00000176024	ZNF613	
ENST00000600853	ENSG00000176024	ZNF613	
ENST00000593379	ENSG00000176024	ZNF613	
ENST00000439937	ENSG00000227122	PRRT1	
ENST00000469341	ENSG00000227122	PRRT1	
ENST00000465468	ENSG00000227122	PRRT1	
ENST00000461643	ENSG00000227122	PRRT1	
ENST00000368518	ENSG00000143612	C1orf43	
ENST00000330514	ENSG00000182963	GJC1	filled
ENST00000586347	ENSG00000182963	GJC1	
ENST00000591424	ENSG00000182963	GJC1	filled
ENST00000426078	ENSG00000198590	C3orf35	
ENST00000452017	ENSG00000198590	C3orf35	
ENST00000559369	ENSG00000182346	DAOA	filled
ENST00000488534	ENSG00000182346	DAOA	filled
ENST00000568865	ENSG00000064666	CNN2	filled
ENST00000556347	ENSG00000258989	RP11-47I22.4	filled
ENST00000515211	ENSG00000135824	RGS8	filled
ENST00000592984	ENSG00000004776	HSPB6	
ENST00000308508	ENSG00000173546	CSPG4	filled
ENST00000561908	ENSG00000261611	ZNF23	filled
ENST00000231173	ENSG00000113248	PCDHB15	
ENST00000623671	ENSG00000113248	PCDHB15	
ENST00000402395	ENSG00000198832	SELM	filled
ENST00000221444	ENSG00000104848	KCNA7	
ENST00000445105	ENSG00000114279	FGF12	filled
ENST00000450716	ENSG00000114279	FGF12	filled
ENST00000430714	ENSG00000114279	FGF12	filled
ENST00000448795	ENSG00000114279	FGF12	filled
ENST00000418610	ENSG00000114279	FGF12	filled
ENST00000466144	ENSG00000114279	FGF12	filled
ENST00000310045	ENSG00000171451	DSEL	
ENST00000573745	ENSG00000181885	CLDN7	
ENST00000587708	ENSG00000205155	PSENEN	
ENST00000528697	ENSG00000148948	LRRC4C	filled
ENST00000530763	ENSG00000148948	LRRC4C	filled
ENST00000534577	ENSG00000148948	LRRC4C	filled
ENST00000320431	ENSG00000176020	AMIGO3	
ENST00000372634	ENSG00000166681	NGFRAP1	filled
ENST00000440303	ENSG00000182183	FAM159A	filled
ENST00000401050	ENSG00000182183	FAM159A	filled
ENST00000424164	ENSG00000182183	FAM159A	filled

ENST00000551774	ENSG00000100916	BRMS1L	filled
ENST00000619801	ENSG00000101266	CSNK2A1	
ENST00000471038	ENSG00000271810	RP11-426L16.10	filled
ENST00000607158	ENSG00000271810	RP11-426L16.10	filled
ENST00000606505	ENSG00000271810	RP11-426L16.10	filled
ENST00000605933	ENSG00000271810	RP11-426L16.10	filled
ENST00000606954	ENSG00000271810	RP11-426L16.10	filled
ENST00000529604	ENSG00000134569	LRP4	
ENST00000271751	ENSG00000143473	KCNH1	filled
ENST00000367007	ENSG00000143473	KCNH1	filled
ENST00000428830	ENSG00000149554	CHEK1	filled
ENST00000616281	ENSG00000135638	EMX1	
ENST00000564382	ENSG00000103067	ESRP2	
ENST00000562724	ENSG00000103067	ESRP2	
ENST00000367311	ENSG00000174307	PHLDA3	filled
ENST00000485436	ENSG00000174307	PHLDA3	filled
ENST00000367309	ENSG00000174307	PHLDA3	filled
ENST00000621127	ENSG00000158186	MRAS	
ENST00000515275	ENSG00000248329	APELA	
ENST00000510062	ENSG00000248329	APELA	
ENST00000522782	ENSG00000037749	MFAP3	
ENST00000439768	ENSG00000037749	MFAP3	
ENST00000520327	ENSG00000037749	MFAP3	
ENST00000322602	ENSG00000037749	MFAP3	
ENST00000522177	ENSG00000037749	MFAP3	
ENST00000520899	ENSG00000037749	MFAP3	
ENST00000522440	ENSG00000037749	MFAP3	
ENST00000518497	ENSG00000037749	MFAP3	
ENST00000519325	ENSG00000037749	MFAP3	filled
ENST00000557624	ENSG00000129465	RIPK3	
ENST00000267425	ENSG00000196943	NOP9	filled
ENST00000383800	ENSG00000157087	ATP2B2	filled
ENST00000460129	ENSG00000157087	ATP2B2	filled
ENST00000397077	ENSG00000157087	ATP2B2	filled
ENST00000480680	ENSG00000157087	ATP2B2	filled
ENST00000360273	ENSG00000157087	ATP2B2	filled
ENST00000229824	ENSG00000255587	RAB44	
ENST00000612677	ENSG00000255587	RAB44	filled
ENST00000566742	ENSG00000206053	HN1L	
ENST00000392170	ENSG00000186017	ZNF566	
ENST00000480361	ENSG00000186017	ZNF566	
ENST00000494354	ENSG00000186017	ZNF566	
ENST00000374908	ENSG00000147059	SPIN2A	
ENST00000589713	ENSG00000267127	RP11-795F19.5	
ENST00000518304	ENSG00000214954	LRRC69	
ENST00000331715	ENSG00000185585	OLFML2A	filled
ENST00000373580	ENSG00000185585	OLFML2A	filled
ENST00000288815	ENSG00000185585	OLFML2A	filled
ENST00000261489	ENSG00000102804	TSC22D1	
ENST00000611198	ENSG00000102804	TSC22D1	
ENST00000622051	ENSG00000102804	TSC22D1	
ENST00000416005	ENSG00000243943	ZNF512	filled
ENST00000413371	ENSG00000243943	ZNF512	filled
ENST00000556601	ENSG00000243943	ZNF512	filled
ENST00000369636	ENSG00000155366	RHOC	
ENST00000518778	ENSG00000161013	MGAT4B	
ENST00000519836	ENSG00000161013	MGAT4B	
ENST00000520875	ENSG00000161013	MGAT4B	
ENST00000518702	ENSG00000161013	MGAT4B	
ENST00000521305	ENSG00000161013	MGAT4B	

ENST00000434483	ENSG00000123560	PLP1	
ENST00000530332	ENSG00000170619	COMMD5	
ENST00000416512	ENSG00000225828	FAM229A	
ENST00000428500	ENSG00000225828	FAM229A	
ENST00000415596	ENSG00000225828	FAM229A	
ENST00000476666	ENSG00000102786	INTS6	
ENST00000351797	ENSG00000055147	FAM114A2	filled
ENST00000217133	ENSG00000101162	TUBB1	filled
ENST00000508821	ENSG00000164181	ELOVL7	filled
ENST00000511799	ENSG00000164181	ELOVL7	filled
ENST00000507047	ENSG00000164181	ELOVL7	filled
ENST00000425382	ENSG00000164181	ELOVL7	filled
ENST00000471129	ENSG00000117707	PROX1	filled
ENST00000519541	ENSG00000205236	RP11-514P8.6	filled
ENST00000479987	ENSG00000197415	VEPH1	
ENST00000487753	ENSG00000197415	VEPH1	
ENST00000473907	ENSG00000197415	VEPH1	
ENST00000475050	ENSG00000197415	VEPH1	
ENST00000461299	ENSG00000197415	VEPH1	
ENST00000524041	ENSG00000011083	SLC6A7	filled
ENST00000417185	ENSG00000187091	PLCD1	
ENST00000561103	ENSG00000100911	PSME2	
ENST00000348165	ENSG00000009335	UBE3C	filled
ENST00000430750	ENSG00000009335	UBE3C	filled
ENST00000503565	ENSG00000151466	SCLT1	filled
ENST00000604395	ENSG00000269437	NXF2B	filled
ENST00000506598	ENSG00000178394	HTR1A	
ENST00000394915	ENSG00000138303	ASCC1	filled
ENST00000466624	ENSG00000063660	GPC1	
ENST00000521923	ENSG00000169946	ZFPM2	filled
ENST00000488564	ENSG00000106052	TAX1BP1	
ENST00000460059	ENSG00000106052	TAX1BP1	
ENST00000367135	ENSG00000174502	SLC26A9	
ENST00000367134	ENSG00000174502	SLC26A9	
ENST00000517896	ENSG00000159239	C2orf81	
ENST00000518401	ENSG00000159239	C2orf81	
ENST00000290390	ENSG00000159239	C2orf81	filled
ENST00000393975	ENSG00000145861	C1QTNF2	
ENST00000360945	ENSG00000196860	TOMM20L	
ENST00000411589	ENSG00000149582	TMEM25	
ENST00000442938	ENSG00000149582	TMEM25	
ENST00000354284	ENSG00000149582	TMEM25	
ENST00000354064	ENSG00000149582	TMEM25	
ENST00000533627	ENSG00000149582	TMEM25	
ENST00000533102	ENSG00000149582	TMEM25	
ENST00000524522	ENSG00000149582	TMEM25	
ENST00000313236	ENSG00000149582	TMEM25	
ENST00000524725	ENSG00000149582	TMEM25	
ENST00000359862	ENSG00000149582	TMEM25	
ENST00000325509	ENSG00000178860	MSC	
ENST00000521739	ENSG00000178860	MSC	
ENST00000422444	ENSG00000197121	PGAP1	
ENST00000459896	ENSG00000197121	PGAP1	
ENST00000167106	ENSG00000071246	VASH1	
ENST00000554743	ENSG00000071246	VASH1	
ENST00000622320	ENSG00000052749	RRP12	
ENST00000538043	ENSG00000175782	SLC35E3	
ENST00000310455	ENSG00000173281	PPP1R3B	filled
ENST00000395663	ENSG00000124172	ATP5E	
ENST00000471530	ENSG00000257921	RP11-571M6.15	

ENST00000553083	ENSG00000257921	RP11-571M6.15	
ENST00000546504	ENSG00000257921	RP11-571M6.15	
ENST00000461421	ENSG00000166197	NOLC1	
ENST00000476468	ENSG00000166197	NOLC1	
ENST00000477715	ENSG00000145757	SPATA9	
ENST00000477047	ENSG00000145757	SPATA9	
ENST00000316087	ENSG00000145757	SPATA9	
ENST00000379990	ENSG00000145757	SPATA9	
ENST00000555591	ENSG00000258973	RP11-934B9.3	
ENST00000623963	ENSG00000279906	DKFZP313A047	
ENST00000559168	ENSG00000054690	PLEKHH1	
ENST00000558214	ENSG00000054690	PLEKHH1	
ENST00000356346	ENSG00000178209	PLEC	filled
ENST00000436759	ENSG00000178209	PLEC	filled
ENST00000527096	ENSG00000178209	PLEC	filled
ENST00000528025	ENSG00000178209	PLEC	filled
ENST00000528131	ENSG00000178209	PLEC	filled
ENST00000261655	ENSG00000060709	RIMBP2	filled
ENST00000403346	ENSG00000115694	STK25	filled
ENST00000401869	ENSG00000115694	STK25	filled
ENST00000405883	ENSG00000115694	STK25	filled
ENST00000494699	ENSG00000115694	STK25	
ENST00000487962	ENSG00000115694	STK25	filled
ENST00000405585	ENSG00000115694	STK25	filled
ENST00000478403	ENSG00000115694	STK25	filled
ENST00000470438	ENSG00000115694	STK25	filled
ENST00000465009	ENSG00000115694	STK25	filled
ENST00000543554	ENSG00000115694	STK25	filled
ENST00000535007	ENSG00000115694	STK25	filled
ENST00000497450	ENSG00000088881	EBF4	filled
ENST00000469215	ENSG00000088881	EBF4	filled
ENST00000275732	ENSG00000146830	GIGYF1	
ENST00000358757	ENSG00000258677	RP11-463D19.2	filled
ENST00000424889	ENSG00000156873	PHKG2	
ENST00000467161	ENSG00000165695	AK8	filled
ENST00000418163	ENSG00000250151	ARPC4-TTLL3	
ENST00000424442	ENSG00000250151	ARPC4-TTLL3	
ENST00000397256	ENSG00000250151	ARPC4-TTLL3	filled
ENST00000453882	ENSG00000250151	ARPC4-TTLL3	
ENST00000478993	ENSG00000215301	DDX3X	
ENST00000616050	ENSG00000215301	DDX3X	
ENST00000263083	ENSG00000108963	DPH1	filled
ENST00000575667	ENSG00000108963	DPH1	filled
ENST00000571418	ENSG00000108963	DPH1	filled
ENST00000570833	ENSG00000108963	DPH1	
ENST00000576891	ENSG00000108963	DPH1	
ENST00000572214	ENSG00000108963	DPH1	filled
ENST00000575162	ENSG00000108963	DPH1	
ENST00000549039	ENSG00000175215	CTDSP2	
ENST00000408906	ENSG00000221836	OR2A5	
ENST00000355481	ENSG00000196218	RYR1	filled
ENST00000359596	ENSG00000196218	RYR1	filled
ENST00000594335	ENSG00000196218	RYR1	filled
ENST00000596431	ENSG00000196218	RYR1	filled
ENST00000593322	ENSG00000196218	RYR1	filled
ENST00000360985	ENSG00000196218	RYR1	filled
ENST00000504560	ENSG00000038295	TLL1	filled
ENST00000300403	ENSG00000088325	TPX2	filled
ENST00000340513	ENSG00000088325	TPX2	filled
ENST00000418900	ENSG00000181061	HIGD1A	

ENST00000555997	ENSG00000185650	ZFP36L1	
ENST00000557086	ENSG00000185650	ZFP36L1	filled
ENST00000553375	ENSG00000185650	ZFP36L1	filled
ENST00000383180	ENSG00000181322	NME9	
ENST00000492993	ENSG00000181322	NME9	
ENST00000484930	ENSG00000181322	NME9	
ENST00000317876	ENSG00000181322	NME9	
ENST00000533056	ENSG00000177830	CHID1	
ENST00000533059	ENSG00000177830	CHID1	
ENST00000525840	ENSG00000177830	CHID1	
ENST00000503970	ENSG00000112893	MAN2A1	
ENST00000276204	ENSG00000147251	DOCK11	filled
ENST00000276202	ENSG00000147251	DOCK11	filled
ENST00000261332	ENSG00000172466	ZNF24	
ENST00000589881	ENSG00000172466	ZNF24	
ENST00000399061	ENSG00000172466	ZNF24	
ENST00000347140	ENSG00000179912	R3HDM2	filled
ENST00000402412	ENSG00000179912	R3HDM2	filled
ENST00000403821	ENSG00000179912	R3HDM2	filled
ENST00000466401	ENSG00000179912	R3HDM2	filled
ENST00000441315	ENSG00000004846	ABCB5	filled
ENST00000517969	ENSG00000070718	AP3M2	
ENST00000248248	ENSG00000103111	MON1B	
ENST00000442586	ENSG00000127955	GNAI1	
ENST00000405749	ENSG00000177994	C2orf73	filled
ENST00000491538	ENSG00000177994	C2orf73	filled
ENST00000415322	ENSG00000226936	B3GALT4	filled
ENST00000476214	ENSG00000168452	PPT2	
ENST00000457226	ENSG00000080503	SMARCA2	filled
ENST00000439732	ENSG00000080503	SMARCA2	filled
ENST00000253812	ENSG00000254245	PCDHGA3	filled
ENST00000612467	ENSG00000254245	PCDHGA3	filled
ENST00000585155	ENSG00000079101	CLUL1	
ENST00000557936	ENSG00000069966	GNB5	filled
ENST00000375036	ENSG00000182534	MXRA7	filled
ENST00000449428	ENSG00000182534	MXRA7	filled
ENST00000588114	ENSG00000182534	MXRA7	filled
ENST00000507768	ENSG00000163257	DCAF16	
ENST00000587191	ENSG00000011304	PTBP1	
ENST00000390666	ENSG00000212122	TSSK1B	
ENST00000342203	ENSG00000117155	SSX2IP	
ENST00000481102	ENSG00000117155	SSX2IP	
ENST00000605755	ENSG00000117155	SSX2IP	
ENST00000603677	ENSG00000117155	SSX2IP	
ENST00000422026	ENSG00000117155	SSX2IP	
ENST00000437941	ENSG00000117155	SSX2IP	
ENST00000525951	ENSG00000071203	MS4A12	
ENST00000374160	ENSG00000165383	LRRC18	
ENST00000298124	ENSG00000165383	LRRC18	
ENST00000392842	ENSG00000111783	RFX4	filled
ENST00000546882	ENSG00000111783	RFX4	filled
ENST00000549040	ENSG00000111783	RFX4	filled
ENST00000539967	ENSG00000111783	RFX4	filled
ENST00000357881	ENSG00000111783	RFX4	filled
ENST00000495825	ENSG00000214941	ZSWIM7	filled
ENST00000475498	ENSG00000214941	ZSWIM7	filled
ENST00000432145	ENSG00000198088	NUP62CL	
ENST00000421752	ENSG00000198088	NUP62CL	filled
ENST00000515033	ENSG00000239388	ASB14	
ENST00000555049	ENSG00000090061	CCNK	filled

ENST00000367657	ENSG00000152092	ASTN1	filled
ENST00000361833	ENSG00000152092	ASTN1	filled
ENST00000424564	ENSG00000152092	ASTN1	filled
ENST00000281881	ENSG00000152092	ASTN1	filled
ENST00000615353	ENSG00000276180	HIST1H4I	
ENST00000486967	ENSG00000219626	FAM228B	filled
ENST00000611138	ENSG00000219626	FAM228B	
ENST00000428778	ENSG00000204314	PRRT1	
ENST00000486917	ENSG00000204314	PRRT1	
ENST00000494332	ENSG00000204314	PRRT1	
ENST00000475826	ENSG00000204314	PRRT1	
ENST00000229214	ENSG00000111615	KRR1	
ENST00000556699	ENSG00000129460	NGDN	filled
ENST00000309755	ENSG00000146021	KLHL3	
ENST00000509694	ENSG00000146021	KLHL3	
ENST00000319441	ENSG00000124253	PCK1	
ENST00000611550	ENSG00000163286	ALPPL2	filled
ENST00000531839	ENSG00000258472	RP11-192H23.4	
ENST00000294383	ENSG00000162402	USP24	filled
ENST00000480652	ENSG00000135577	NMBR	filled
ENST00000578207	ENSG00000264343	NOTCH2NL	filled
ENST00000533144	ENSG00000151364	KCTD14	filled
ENST00000614236	ENSG00000151364	KCTD14	filled
ENST00000252087	ENSG00000240764	PCDHGC5	filled
ENST00000381359	ENSG00000140264	SERF2	filled
ENST00000339624	ENSG00000140264	SERF2	
ENST00000281187	ENSG00000151502	VPS26B	
ENST00000525095	ENSG00000151502	VPS26B	
ENST00000527586	ENSG00000151502	VPS26B	
ENST00000532160	ENSG00000151502	VPS26B	
ENST00000531741	ENSG00000151502	VPS26B	
ENST00000396355	ENSG00000072864	NDE1	filled
ENST00000391844	ENSG00000105221	AKT2	
ENST00000565867	ENSG00000102910	LONP2	
ENST00000564259	ENSG00000102910	LONP2	
ENST00000566719	ENSG00000102910	LONP2	
ENST00000565185	ENSG00000102910	LONP2	
ENST00000473915	ENSG00000196305	IARS	
ENST00000430417	ENSG00000196305	IARS	
ENST00000447699	ENSG00000196305	IARS	filled
ENST00000443024	ENSG00000196305	IARS	filled
ENST00000239032	ENSG00000119973	PRLHR	filled
ENST00000494857	ENSG00000003989	SLC7A2	filled
ENST00000522656	ENSG00000003989	SLC7A2	filled
ENST00000470360	ENSG00000003989	SLC7A2	filled
ENST00000491177	ENSG00000189171	S100A13	filled
ENST00000572734	ENSG00000263186	KRTAP16-1	
ENST00000377084	ENSG00000178235	SLITRK1	filled
ENST00000373710	ENSG00000168528	SERINC2	filled
ENST00000487207	ENSG00000168528	SERINC2	filled
ENST00000491976	ENSG00000168528	SERINC2	filled
ENST00000536859	ENSG00000168528	SERINC2	filled
ENST00000600651	ENSG00000105755	ETHE1	filled
ENST00000598330	ENSG00000105755	ETHE1	filled
ENST00000556491	ENSG00000081181	ARG2	
ENST00000470592	ENSG00000065911	MTHFD2	
ENST00000489041	ENSG00000065911	MTHFD2	
ENST00000360079	ENSG00000196419	XRCC6	filled
ENST00000402580	ENSG00000196419	XRCC6	filled
ENST00000405878	ENSG00000196419	XRCC6	filled

ENST00000428575	ENSG00000196419	XRCC6	filled
ENST00000421514	ENSG00000136935	GOLGA1	
ENST00000605438	ENSG00000136935	GOLGA1	
ENST00000263754	ENSG00000114166	KAT2B	
ENST00000367942	ENSG00000118217	ATF6	filled
ENST00000296509	ENSG00000164109	MAD2L1	
ENST00000590258	ENSG00000134779	TPGS2	
ENST00000590652	ENSG00000134779	TPGS2	
ENST00000614939	ENSG00000134779	TPGS2	filled
ENST00000372582	ENSG00000189108	IL1RAPL2	filled
ENST00000585183	ENSG00000178921	PFAS	filled
ENST00000569170	ENSG00000260234	RP11-166N6.3	filled
ENST00000562308	ENSG00000260234	RP11-166N6.3	filled
ENST00000565169	ENSG00000260234	RP11-166N6.3	
ENST00000438257	ENSG00000211448	DIO2	
ENST00000557010	ENSG00000211448	DIO2	
ENST00000554188	ENSG00000211448	DIO2	filled
ENST00000422005	ENSG00000211448	DIO2	
ENST00000367090	ENSG00000146433	TMEM181	filled
ENST00000383768	ENSG00000206559	ZCWPW2	filled
ENST00000457897	ENSG00000206559	ZCWPW2	filled
ENST00000456845	ENSG00000169490	TM2D2	
ENST00000456397	ENSG00000169490	TM2D2	
ENST00000328345	ENSG00000184486	POU3F2	
ENST00000523304	ENSG00000185697	MYBL1	
ENST00000623024	ENSG00000279767	AL513523.2	
ENST00000337318	ENSG00000189319	FAM53B	filled
ENST00000392754	ENSG00000189319	FAM53B	filled
ENST00000280780	ENSG00000189319	FAM53B	filled
ENST00000310343	ENSG00000134909	ARHGAP32	filled
ENST00000526162	ENSG00000134909	ARHGAP32	
ENST00000392657	ENSG00000134909	ARHGAP32	
ENST00000524655	ENSG00000134909	ARHGAP32	filled
ENST00000527272	ENSG00000134909	ARHGAP32	
ENST00000533509	ENSG00000134909	ARHGAP32	filled
ENST00000525234	ENSG00000134909	ARHGAP32	filled
ENST00000476971	ENSG00000170965	PLAC1	filled
ENST00000466797	ENSG00000170965	PLAC1	filled
ENST00000595444	ENSG00000130312	MRPL34	
ENST00000600434	ENSG00000130312	MRPL34	
ENST00000597996	ENSG00000130312	MRPL34	
ENST00000594999	ENSG00000130312	MRPL34	
ENST00000409379	ENSG00000160949	TONSL	filled
ENST00000568305	ENSG00000101440	ASIP	filled
ENST00000586600	ENSG00000104957	CCDC130	
ENST00000498721	ENSG00000138162	TACC2	filled
ENST00000462400	ENSG00000090905	TNRC6A	filled
ENST00000534445	ENSG00000196378	ZNF34	
ENST00000411574	ENSG00000229680	OR2H2	
ENST00000592844	ENSG00000159917	ZNF235	filled
ENST00000589799	ENSG00000159917	ZNF235	filled
ENST00000262198	ENSG00000101544	ADNP2	
ENST00000619854	ENSG00000273943	CNOT3	filled
ENST00000432416	ENSG00000144401	METTL21A	filled
ENST00000458426	ENSG00000144401	METTL21A	filled
ENST00000397518	ENSG00000184708	EIF4ENIF1	
ENST00000397520	ENSG00000184708	EIF4ENIF1	
ENST00000530364	ENSG00000165209	STRBP	filled
ENST00000478973	ENSG00000165209	STRBP	filled
ENST00000471564	ENSG00000165209	STRBP	filled

ENST00000479114	ENSG00000165209	STRBP	filled
ENST00000286301	ENSG00000182578	CSF1R	filled
ENST00000511344	ENSG00000182578	CSF1R	filled
ENST00000503473	ENSG00000213347	MXD3	filled
ENST00000509339	ENSG00000213347	MXD3	
ENST00000427908	ENSG00000213347	MXD3	filled
ENST00000337907	ENSG00000142599	RERE	filled
ENST00000400908	ENSG00000142599	RERE	filled
ENST00000480342	ENSG00000142599	RERE	filled
ENST00000468247	ENSG00000142599	RERE	filled
ENST00000368471	ENSG00000160710	ADAR	
ENST00000463920	ENSG00000160710	ADAR	filled
ENST00000471068	ENSG00000160710	ADAR	
ENST00000368930	ENSG00000168438	CDC40	filled
ENST00000530898	ENSG00000178719	GRINA	
ENST00000332810	ENSG00000181026	AEN	
ENST00000560174	ENSG00000181026	AEN	
ENST00000558327	ENSG00000181026	AEN	
ENST00000583413	ENSG00000108262	GIT1	
ENST00000580271	ENSG00000166685	COG1	filled
ENST00000558289	ENSG00000104177	MYEF2	filled
ENST00000567449	ENSG00000187951	ARHGAP11B	
ENST00000552361	ENSG00000197728	RPS26	
ENST00000503641	ENSG00000159199	ATP5G1	
ENST00000514808	ENSG00000159199	ATP5G1	
ENST00000506855	ENSG00000159199	ATP5G1	
ENST00000521288	ENSG00000198585	NUDT16	
ENST00000537561	ENSG00000198585	NUDT16	filled
ENST00000295463	ENSG00000163295	ALPI	
ENST00000217426	ENSG00000101444	AHCY	
ENST00000375647	ENSG00000184677	ZBTB40	filled
ENST00000404138	ENSG00000184677	ZBTB40	filled
ENST00000374651	ENSG00000184677	ZBTB40	filled
ENST00000472448	ENSG00000153904	DDAH1	
ENST00000598758	ENSG00000105329	TGFB1	filled
ENST00000546609	ENSG00000111012	CYP27B1	
ENST00000373401	ENSG00000112139	MDGA1	
ENST00000534227	ENSG00000151503	NCAPD3	
ENST00000532445	ENSG00000151503	NCAPD3	
ENST00000409972	ENSG00000153250	RBMS1	
ENST00000533715	ENSG00000173137	ADCK5	filled
ENST00000622727	ENSG00000269502	DMRTC1	filled
ENST00000615063	ENSG00000269502	DMRTC1	filled
ENST00000532179	ENSG00000133816	MICAL2	
ENST00000526065	ENSG00000133816	MICAL2	
ENST00000530823	ENSG00000133816	MICAL2	
ENST00000370566	ENSG00000122417	ODF2L	filled
ENST00000294678	ENSG00000122417	ODF2L	filled
ENST00000488879	ENSG00000122417	ODF2L	filled
ENST00000479890	ENSG00000122417	ODF2L	filled
ENST00000394733	ENSG00000122417	ODF2L	filled
ENST00000478286	ENSG00000122417	ODF2L	filled
ENST00000317336	ENSG00000122417	ODF2L	filled
ENST00000555868	ENSG00000182107	TMEM30B	
ENST00000557163	ENSG00000182107	TMEM30B	
ENST00000554497	ENSG00000182107	TMEM30B	
ENST00000422245	ENSG00000161011	SQSTM1	
ENST00000361771	ENSG00000142784	WDTC1	
ENST00000319394	ENSG00000142784	WDTC1	
ENST00000397802	ENSG00000168495	POLR3D	

ENST00000344626	ENSG00000127616	SMARCA4	filled
ENST00000429416	ENSG00000127616	SMARCA4	filled
ENST00000541122	ENSG00000127616	SMARCA4	filled
ENST00000589677	ENSG00000127616	SMARCA4	filled
ENST00000444061	ENSG00000127616	SMARCA4	filled
ENST00000590574	ENSG00000127616	SMARCA4	filled
ENST00000468573	ENSG00000133019	CHRM3	filled
ENST00000448020	ENSG00000133019	CHRM3	filled
ENST00000615928	ENSG00000133019	CHRM3	filled
ENST00000395386	ENSG00000129292	PHF20L1	
ENST00000395390	ENSG00000129292	PHF20L1	
ENST00000460236	ENSG00000129292	PHF20L1	
ENST00000622263	ENSG00000129292	PHF20L1	
ENST00000220847	ENSG00000129292	PHF20L1	
ENST00000478328	ENSG00000106328	FSCN3	
ENST00000467509	ENSG00000162600	OMA1	filled
ENST00000460671	ENSG00000162600	OMA1	filled
ENST00000555185	ENSG00000027075	PRKCH	filled
ENST00000574792	ENSG00000162526	TSSK3	
ENST00000574315	ENSG00000162526	TSSK3	
ENST00000599243	ENSG00000268400	CTD-3214H19.4	
ENST00000595866	ENSG00000268400	CTD-3214H19.4	
ENST00000394196	ENSG00000173575	CHD2	filled
ENST00000622527	ENSG00000253910	PCDHGB2	
ENST00000522605	ENSG00000253910	PCDHGB2	filled
ENST00000529882	ENSG00000112339	HBS1L	
ENST00000505545	ENSG00000034533	ASTE1	
ENST00000506821	ENSG00000164237	CMBL	filled
ENST00000376705	ENSG00000185352	HS6ST3	filled
ENST00000620595	ENSG00000185352	HS6ST3	
ENST00000579309	ENSG00000141068	KSR1	filled
ENST00000491912	ENSG00000064225	ST3GAL6	
ENST00000505478	ENSG00000109756	RAPGEF2	filled
ENST00000504604	ENSG00000109756	RAPGEF2	filled
ENST00000503328	ENSG00000109756	RAPGEF2	
ENST00000471261	ENSG00000049769	PPP1R3F	filled
ENST00000449987	ENSG00000139746	RBM26	
ENST00000397286	ENSG00000131043	AAR2	
ENST00000373932	ENSG00000131043	AAR2	
ENST00000569166	ENSG00000169181	GSG1L	filled
ENST00000562611	ENSG00000169181	GSG1L	filled
ENST00000557623	ENSG00000119705	SLIRP	
ENST00000557431	ENSG00000119705	SLIRP	filled
ENST00000397967	ENSG00000129538	RNASE1	
ENST00000484993	ENSG00000144455	SUMF1	filled
ENST00000445110	ENSG00000077942	FBLN1	
ENST00000450975	ENSG00000077942	FBLN1	
ENST00000600960	ENSG00000167654	ATCAY	filled
ENST00000471660	ENSG00000083937	CHMP2B	filled
ENST00000263780	ENSG00000083937	CHMP2B	filled
ENST00000494980	ENSG00000083937	CHMP2B	filled
ENST00000520790	ENSG00000253305	PCDHGB6	filled
ENST00000477593	ENSG00000051382	PIK3CB	filled
ENST00000462898	ENSG00000051382	PIK3CB	filled
ENST00000483968	ENSG00000051382	PIK3CB	filled
ENST00000461451	ENSG00000051382	PIK3CB	filled
ENST00000265715	ENSG00000091137	SLC26A4	filled
ENST00000519398	ENSG00000161010	C5orf45	
ENST00000455852	ENSG00000168890	TMEM150A	
ENST00000469702	ENSG00000115084	SLC35F5	

ENST00000511166	ENSG00000250709	CCDC169-SOHLH2	filled
ENST00000546878	ENSG00000065357	DGKA	
ENST00000548491	ENSG00000065357	DGKA	
ENST00000571252	ENSG00000262576	PCDHGA4	filled
ENST00000612927	ENSG00000262576	PCDHGA4	
ENST00000576655	ENSG00000261949	GFY	
ENST00000610896	ENSG00000261949	GFY	
ENST00000393913	ENSG00000115363	EVA1A	
ENST00000410113	ENSG00000115363	EVA1A	
ENST00000233712	ENSG00000115363	EVA1A	
ENST00000271715	ENSG00000143442	POGZ	
ENST00000450842	ENSG00000143442	POGZ	
ENST00000554541	ENSG00000100902	PSMA6	
ENST00000261479	ENSG00000100902	PSMA6	
ENST00000554620	ENSG00000100902	PSMA6	
ENST00000554961	ENSG00000100902	PSMA6	
ENST00000553809	ENSG00000100902	PSMA6	
ENST00000555764	ENSG00000100902	PSMA6	
ENST00000556506	ENSG00000100902	PSMA6	
ENST00000622405	ENSG00000100902	PSMA6	
ENST00000475436	ENSG00000182601	HS3ST4	filled
ENST00000576861	ENSG00000183011	NAA38	
ENST00000570555	ENSG00000183011	NAA38	
ENST00000327757	ENSG00000163803	PLB1	filled
ENST00000404858	ENSG00000163803	PLB1	filled
ENST00000479065	ENSG00000163803	PLB1	
ENST00000411743	ENSG00000163803	PLB1	
ENST00000444257	ENSG00000163803	PLB1	
ENST00000422425	ENSG00000163803	PLB1	filled
ENST00000612924	ENSG00000274176	CNOT3	filled
ENST00000358433	ENSG00000188324	OR6C6	
ENST00000541819	ENSG00000166206	GABRB3	filled
ENST00000553306	ENSG00000258466	RP11-1012A1.4	filled
ENST00000553582	ENSG00000258466	RP11-1012A1.4	filled
ENST00000554493	ENSG00000258466	RP11-1012A1.4	filled
ENST00000557564	ENSG00000258466	RP11-1012A1.4	filled
ENST00000402685	ENSG00000165966	PDZRN4	filled
ENST00000432058	ENSG00000115415	STAT1	
ENST00000398637	ENSG00000111371	SLC38A1	
ENST00000549049	ENSG00000111371	SLC38A1	
ENST00000439706	ENSG00000111371	SLC38A1	
ENST00000546893	ENSG00000111371	SLC38A1	
ENST00000549633	ENSG00000111371	SLC38A1	
ENST00000551506	ENSG00000111371	SLC38A1	
ENST00000550173	ENSG00000111371	SLC38A1	
ENST00000468334	ENSG00000169194	IL13	filled
ENST00000487267	ENSG00000169194	IL13	
ENST00000414615	ENSG00000159871	LYPD5	
ENST00000602179	ENSG00000159871	LYPD5	
ENST00000321521	ENSG00000145725	PPIP5K2	filled
ENST00000507921	ENSG00000145725	PPIP5K2	filled
ENST00000502481	ENSG00000145725	PPIP5K2	filled
ENST00000480383	ENSG00000236649	PPT2	
ENST00000399910	ENSG00000215277	RNF212B	filled
ENST00000498441	ENSG00000215277	RNF212B	
ENST00000576936	ENSG00000162104	ADCY9	filled
ENST00000509665	ENSG00000092421	SEMA6A	filled
ENST00000512156	ENSG00000092421	SEMA6A	
ENST00000425953	ENSG00000233757	AC092835.2	
ENST00000336053	ENSG00000092199	HNRNPC	

ENST00000554969	ENSG00000092199	HNRNPC	
ENST00000556142	ENSG00000092199	HNRNPC	
ENST00000554455	ENSG00000092199	HNRNPC	
ENST00000555309	ENSG00000092199	HNRNPC	
ENST00000557442	ENSG00000092199	HNRNPC	
ENST00000556513	ENSG00000092199	HNRNPC	
ENST00000557201	ENSG00000092199	HNRNPC	
ENST00000554383	ENSG00000092199	HNRNPC	
ENST00000555215	ENSG00000092199	HNRNPC	filled
ENST00000555137	ENSG00000092199	HNRNPC	filled
ENST00000554891	ENSG00000092199	HNRNPC	
ENST00000556226	ENSG00000092199	HNRNPC	filled
ENST00000555176	ENSG00000092199	HNRNPC	
ENST00000553614	ENSG00000092199	HNRNPC	filled
ENST00000557336	ENSG00000092199	HNRNPC	filled
ENST00000557768	ENSG00000092199	HNRNPC	filled
ENST00000557033	ENSG00000092199	HNRNPC	filled
ENST00000555127	ENSG00000092199	HNRNPC	
ENST00000506087	ENSG00000170088	TMEM192	filled
ENST00000379426	ENSG00000205269	TMEM170B	
ENST00000497734	ENSG00000197122	SRC	filled
ENST00000373578	ENSG00000197122	SRC	filled
ENST00000477066	ENSG00000197122	SRC	
ENST00000571846	ENSG00000182224	CYB5D1	
ENST00000332439	ENSG00000182224	CYB5D1	
ENST00000561468	ENSG00000100865	CINP	
ENST00000558523	ENSG00000100865	CINP	
ENST00000520509	ENSG00000175305	CCNE2	
ENST00000393705	ENSG00000137496	IL18BP	filled
ENST00000337131	ENSG00000137496	IL18BP	filled
ENST00000531053	ENSG00000137496	IL18BP	filled
ENST00000343898	ENSG00000137496	IL18BP	filled
ENST00000534583	ENSG00000137496	IL18BP	
ENST00000404792	ENSG00000137496	IL18BP	filled
ENST00000620017	ENSG00000137496	IL18BP	filled
ENST00000376302	ENSG00000095777	MYO3A	filled
ENST00000358600	ENSG00000123146	CD97	
ENST00000538617	ENSG00000150991	UBC	
ENST00000542416	ENSG00000150991	UBC	
ENST00000575724	ENSG00000262607	IQSEC3	filled
ENST00000621764	ENSG00000262607	IQSEC3	filled
ENST00000561981	ENSG00000260230	FRRS1L	
ENST00000498398	ENSG00000102313	ITIH6	
ENST00000240587	ENSG00000121297	TSHZ3	
ENST00000560707	ENSG00000121297	TSHZ3	
ENST00000448696	ENSG00000184995	IFNE	
ENST00000611598	ENSG00000254221	PCDHGB1	
ENST00000523390	ENSG00000254221	PCDHGB1	filled
ENST00000427659	ENSG00000226931	OR2J1	
ENST00000585562	ENSG00000267261	CTD-2132N18.3	
ENST00000592248	ENSG00000267261	CTD-2132N18.3	
ENST00000592574	ENSG00000267261	CTD-2132N18.3	
ENST00000580017	ENSG00000189186	DCAF8L2	
ENST00000448590	ENSG00000242386	HLA-DMB	filled
ENST00000533663	ENSG00000183378	OVCH2	
ENST00000612000	ENSG00000183378	OVCH2	
ENST00000410048	ENSG00000186973	FAM183A	filled
ENST00000406697	ENSG00000182326	C1S	
ENST00000232854	ENSG00000114735	HEMK1	
ENST00000448997	ENSG00000114735	HEMK1	

ENST00000396667	ENSG00000106460	TMEM106B	filled
ENST00000588891	ENSG00000267314	AC104532.2	
ENST00000478489	ENSG00000006607	FARP2	filled
ENST00000479427	ENSG00000006607	FARP2	filled
ENST00000547940	ENSG00000257341	CRIP1	
ENST00000553228	ENSG00000257341	CRIP1	
ENST00000278550	ENSG00000149256	TENM4	filled
ENST00000528688	ENSG00000149256	TENM4	filled
ENST00000531583	ENSG00000149256	TENM4	filled
ENST00000505762	ENSG00000146147	MLIP	filled
ENST00000610442	ENSG00000276087	RP11-507M3.1	filled
ENST00000369529	ENSG00000203870	SMIM9	
ENST00000552828	ENSG00000136026	CKAP4	filled
ENST00000383209	ENSG00000206285	B3GALT4	filled
ENST00000559171	ENSG00000100889	PCK2	
ENST00000590974	ENSG00000104903	LYL1	filled
ENST00000590120	ENSG00000104903	LYL1	filled
ENST00000590515	ENSG00000171791	BCL2	
ENST00000589955	ENSG00000171791	BCL2	
ENST00000399753	ENSG00000198736	MSRB1	
ENST00000564908	ENSG00000198736	MSRB1	
ENST00000489198	ENSG00000198736	MSRB1	
ENST00000508366	ENSG00000205464	ATP6AP1L	filled
ENST00000502523	ENSG00000205464	ATP6AP1L	
ENST00000338432	ENSG00000076555	ACACB	filled
ENST00000539864	ENSG00000076555	ACACB	filled
ENST00000470661	ENSG00000112245	PTP4A1	filled
ENST00000550559	ENSG00000123297	TSFM	filled
ENST00000548851	ENSG00000123297	TSFM	filled
ENST00000543727	ENSG00000123297	TSFM	filled
ENST00000481424	ENSG00000197037	ZSCAN25	filled
ENST00000394152	ENSG00000197037	ZSCAN25	filled
ENST00000431485	ENSG00000197037	ZSCAN25	
ENST00000582343	ENSG00000232859	LYRM9	
ENST00000470124	ENSG00000101940	WDR13	filled
ENST00000376729	ENSG00000101940	WDR13	
ENST00000358450	ENSG00000128655	PDE11A	filled
ENST00000610406	ENSG00000275724	NDUFA3	
ENST00000534905	ENSG00000168010	ATG16L2	
ENST00000614461	ENSG00000274523	WBSCR16	
ENST00000347132	ENSG00000117013	KCNQ4	filled
ENST00000509682	ENSG00000117013	KCNQ4	filled
ENST00000465806	ENSG00000196998	WDR45	
ENST00000356463	ENSG00000196998	WDR45	
ENST00000389758	ENSG00000185038	MROH2A	filled
ENST00000480634	ENSG00000185038	MROH2A	filled
ENST00000610772	ENSG00000185038	MROH2A	filled
ENST00000478815	ENSG00000165188	RNF183	
ENST00000297894	ENSG00000165188	RNF183	
ENST00000443976	ENSG00000165188	RNF183	
ENST00000457992	ENSG00000100055	CYTH4	
ENST00000402997	ENSG00000100055	CYTH4	
ENST00000617558	ENSG00000263020	CSNK2B-LY6G5B-1181	
ENST00000622499	ENSG00000263020	CSNK2B-LY6G5B-1181	
ENST00000618754	ENSG00000263020	CSNK2B-LY6G5B-1181	
ENST00000415396	ENSG00000187600	TMEM247	
ENST00000230036	ENSG00000112293	GPLD1	filled
ENST00000474784	ENSG00000112293	GPLD1	filled
ENST00000296218	ENSG00000163879	DNALI1	filled
ENST00000497858	ENSG00000163879	DNALI1	filled

ENST00000316401	ENSG00000179213	SIGLECL1	
ENST00000566339	ENSG00000162032	SPSB3	
ENST00000563741	ENSG00000162032	SPSB3	
ENST00000565550	ENSG00000162032	SPSB3	
ENST00000564070	ENSG00000162032	SPSB3	
ENST00000523486	ENSG00000169122	FAM110B	filled
ENST00000416727	ENSG00000236227	BRD2	
ENST00000491751	ENSG00000236227	BRD2	
ENST00000513079	ENSG00000049860	HEXB	filled
ENST00000503312	ENSG00000049860	HEXB	
ENST00000505859	ENSG00000049860	HEXB	
ENST00000513867	ENSG00000049860	HEXB	
ENST00000483981	ENSG00000161681	SHANK1	
ENST00000379295	ENSG00000123130	ACOT9	filled
ENST00000249363	ENSG00000128594	LRRC4	
ENST00000494115	ENSG00000128594	LRRC4	
ENST00000476782	ENSG00000128594	LRRC4	
ENST00000478726	ENSG00000128594	LRRC4	
ENST00000376358	ENSG00000250232	AF196779.12	
ENST00000373886	ENSG00000122870	BICC1	filled
ENST00000476684	ENSG00000122870	BICC1	
ENST00000492131	ENSG00000263513	FAM72C	
ENST00000382496	ENSG00000112902	SEMA5A	filled
ENST00000514923	ENSG00000112902	SEMA5A	
ENST00000513968	ENSG00000112902	SEMA5A	
ENST00000509486	ENSG00000112902	SEMA5A	
ENST00000551153	ENSG00000178401	DNAJC22	
ENST00000416012	ENSG00000127252	HRASLS	filled
ENST00000565468	ENSG00000261115	TMEM178B	filled
ENST00000563442	ENSG00000261115	TMEM178B	filled
ENST00000610315	ENSG00000261115	TMEM178B	filled
ENST00000370935	ENSG00000165675	ENOX2	filled
ENST00000432489	ENSG00000165675	ENOX2	filled
ENST00000492263	ENSG00000165675	ENOX2	filled
ENST00000338144	ENSG00000165675	ENOX2	filled
ENST00000394363	ENSG00000165675	ENOX2	filled
ENST00000270238	ENSG00000142235	LMTK3	filled
ENST00000515806	ENSG00000109685	WHSC1	
ENST00000495711	ENSG00000203791	METTL10	
ENST00000407040	ENSG00000122687	FTSJ2	
ENST00000607303	ENSG00000050393	MCUR1	filled
ENST00000562332	ENSG00000140873	ADAMTS18	filled
ENST00000515354	ENSG00000205301	MGAT4D	
ENST00000513018	ENSG00000205301	MGAT4D	
ENST00000379919	ENSG00000180660	MAB21L1	
ENST00000616515	ENSG00000278268	MOAP1	
ENST00000443120	ENSG00000213337	ANKRD39	
ENST00000513263	ENSG00000145604	SKP2	
ENST00000580717	ENSG00000125457	MIF4GD	
ENST00000395451	ENSG00000064652	SNX24	filled
ENST00000510914	ENSG00000064652	SNX24	
ENST00000278772	ENSG00000088876	ZNF343	
ENST00000617391	ENSG00000088876	ZNF343	
ENST00000612935	ENSG00000088876	ZNF343	
ENST00000526926	ENSG00000183309	ZNF623	
ENST00000296091	ENSG00000196653	ZNF502	
ENST00000449836	ENSG00000196653	ZNF502	
ENST00000457070	ENSG00000237052	GPSM3	
ENST00000398587	ENSG00000253873	PCDHGA11	filled
ENST00000518882	ENSG00000253873	PCDHGA11	filled

ENST00000456846	ENSG00000137413	TAF8	
ENST00000303059	ENSG00000172489	OR5T3	
ENST00000383640	ENSG00000204657	OR2H2	
ENST00000464326	ENSG00000147872	PLIN2	
ENST00000434144	ENSG00000147872	PLIN2	
ENST00000426480	ENSG00000144381	HSPD1	
ENST00000530656	ENSG00000019485	PRDM11	filled
ENST00000493961	ENSG00000166228	PCBD1	
ENST00000464230	ENSG00000236490	TAPBP	
ENST00000553235	ENSG00000236490	TAPBP	
ENST00000481999	ENSG00000170004	CHD3	filled
ENST00000503658	ENSG00000048342	CC2D2A	filled
ENST00000460562	ENSG00000134882	UBAC2	filled
ENST00000432183	ENSG00000119729	RHOQ	
ENST00000522171	ENSG00000164941	INTS8	filled
ENST00000462692	ENSG00000163126	ANKRD23	
ENST00000360184	ENSG00000197102	DYNC1H1	filled
ENST00000554854	ENSG00000197102	DYNC1H1	
ENST00000556791	ENSG00000197102	DYNC1H1	
ENST00000553423	ENSG00000197102	DYNC1H1	
ENST00000590967	ENSG00000176014	TUBB6	filled
ENST00000586691	ENSG00000176014	TUBB6	filled
ENST00000560875	ENSG00000092098	RNF31	
ENST00000558634	ENSG00000092098	RNF31	
ENST00000557878	ENSG00000092098	RNF31	
ENST00000557991	ENSG00000092098	RNF31	
ENST00000586448	ENSG00000132874	SLC14A2	filled
ENST00000280758	ENSG00000151136	BTBD11	filled
ENST00000420571	ENSG00000151136	BTBD11	filled
ENST00000490090	ENSG00000151136	BTBD11	filled
ENST00000550706	ENSG00000151136	BTBD11	filled
ENST00000245479	ENSG00000125398	SOX9	filled
ENST00000521913	ENSG00000092964	DPYSL2	filled
ENST00000493789	ENSG00000092964	DPYSL2	filled
ENST00000311151	ENSG00000092964	DPYSL2	
ENST00000523027	ENSG00000092964	DPYSL2	
ENST00000523093	ENSG00000092964	DPYSL2	
ENST00000521983	ENSG00000092964	DPYSL2	
ENST00000474808	ENSG00000092964	DPYSL2	
ENST00000372844	ENSG00000116983	HPCAL4	filled
ENST00000612703	ENSG00000116983	HPCAL4	filled
ENST00000617690	ENSG00000116983	HPCAL4	filled
ENST00000622451	ENSG00000167920	TMEM99	filled
ENST00000306011	ENSG00000172238	ATOH1	
ENST00000475121	ENSG00000116254	CHD5	
ENST00000595085	ENSG00000248098	BCKDHA	filled
ENST00000441604	ENSG00000235986	GNL1	
ENST00000311765	ENSG00000172840	PDP2	filled
ENST00000477853	ENSG00000127463	EMC1	filled
ENST00000480380	ENSG00000127463	EMC1	
ENST00000525621	ENSG00000105397	TYK2	
ENST00000529370	ENSG00000105397	TYK2	
ENST00000529317	ENSG00000105397	TYK2	
ENST00000525824	ENSG00000105397	TYK2	
ENST00000381298	ENSG00000134352	IL6ST	filled
ENST00000522633	ENSG00000134352	IL6ST	filled
ENST00000577363	ENSG00000134352	IL6ST	
ENST00000419839	ENSG00000229488	PRRT1	
ENST00000469527	ENSG00000229488	PRRT1	
ENST00000474285	ENSG00000229488	PRRT1	

ENST00000467682	ENSG00000229488	PRRT1	
ENST00000392169	ENSG00000143252	SDHC	
ENST00000513009	ENSG00000143252	SDHC	
ENST00000549068	ENSG00000205352	PRR13	
ENST00000546581	ENSG00000205352	PRR13	
ENST00000549581	ENSG00000205352	PRR13	
ENST00000547368	ENSG00000205352	PRR13	
ENST00000580777	ENSG00000108961	RANGRF	
ENST00000465412	ENSG00000152061	RABGAP1L	filled
ENST00000485114	ENSG00000152061	RABGAP1L	filled
ENST00000392064	ENSG00000152061	RABGAP1L	filled
ENST00000357585	ENSG00000180616	SSTR2	filled
ENST00000489426	ENSG00000184343	SRPK3	
ENST00000252804	ENSG00000130508	PXDN	filled
ENST00000561573	ENSG00000261147	RP11-697E2.6	filled
ENST00000562787	ENSG00000102901	CENPT	filled
ENST00000448642	ENSG00000156515	HK1	filled
ENST00000360289	ENSG00000156515	HK1	filled
ENST00000393797	ENSG00000123329	ARHGAP9	
ENST00000550288	ENSG00000123329	ARHGAP9	
ENST00000466963	ENSG00000168772	CXXC4	filled
ENST00000400128	ENSG00000154645	CHODL	filled
ENST00000400131	ENSG00000154645	CHODL	filled
ENST00000400135	ENSG00000154645	CHODL	filled
ENST00000400127	ENSG00000154645	CHODL	filled
ENST00000369250	ENSG00000151553	FAM160B1	filled
ENST00000369246	ENSG00000151553	FAM160B1	
ENST00000560961	ENSG00000106689	LHX2	filled
ENST00000535844	ENSG00000256861	RP11-512M8.5	
ENST00000503634	ENSG00000151790	TDO2	
ENST00000506072	ENSG00000151790	TDO2	
ENST00000507590	ENSG00000151790	TDO2	
ENST00000598285	ENSG00000105373	GLTSCR2	
ENST00000471765	ENSG00000177674	AGTRAP	filled
ENST00000623359	ENSG00000279566	ZNF43	filled
ENST00000624732	ENSG00000279566	ZNF43	filled
ENST00000623648	ENSG00000279566	ZNF43	filled
ENST00000564233	ENSG00000149927	DOC2A	
ENST00000564979	ENSG00000149927	DOC2A	
ENST00000563378	ENSG00000149927	DOC2A	
ENST00000557082	ENSG00000088808	PPP1R13B	filled
ENST00000412306	ENSG00000112773	FAM46A	filled
ENST00000438194	ENSG00000234507	BRD2	
ENST00000495079	ENSG00000234507	BRD2	
ENST00000546272	ENSG00000157895	C12orf43	
ENST00000539736	ENSG00000157895	C12orf43	
ENST00000592551	ENSG00000130813	C19orf66	filled
ENST00000523149	ENSG00000012232	EXTL3	filled
ENST00000519288	ENSG00000012232	EXTL3	filled
ENST00000220562	ENSG00000012232	EXTL3	
ENST00000518223	ENSG00000012232	EXTL3	
ENST00000434083	ENSG00000242711	PSMB9	
ENST00000425855	ENSG00000242711	PSMB9	filled
ENST00000617770	ENSG00000132965	ALOX5AP	
ENST00000506061	ENSG00000113194	FAF2	
ENST00000510446	ENSG00000113194	FAF2	filled
ENST00000422113	ENSG00000151952	TMEM132D	filled
ENST00000378962	ENSG00000120280	CXorf21	filled
ENST00000572195	ENSG00000262664	OVCA2	
ENST00000326912	ENSG00000178057	NDUFAF3	

ENST00000414440	ENSG00000229496	DDX39B	filled
ENST00000428691	ENSG00000176407	KCMF1	
ENST00000453448	ENSG00000176407	KCMF1	filled
ENST00000506581	ENSG00000163682	RPL9	
ENST00000493092	ENSG00000117362	APH1A	
ENST00000293805	ENSG00000161940	BCL6B	filled
ENST00000537931	ENSG00000161940	BCL6B	filled
ENST00000571729	ENSG00000161940	BCL6B	filled
ENST00000366894	ENSG00000185345	PARK2	filled
ENST00000338468	ENSG00000185345	PARK2	filled
ENST00000479615	ENSG00000185345	PARK2	filled
ENST00000366892	ENSG00000185345	PARK2	filled
ENST00000599537	ENSG00000142546	NOSIP	filled
ENST00000554560	ENSG00000133962	CATSPERB	filled
ENST00000396953	ENSG00000144713	RPL32	
ENST00000458420	ENSG00000158258	CLSTN2	filled
ENST00000511524	ENSG00000158258	CLSTN2	filled
ENST00000303343	ENSG00000169241	SLC50A1	filled
ENST00000368404	ENSG00000169241	SLC50A1	filled
ENST00000368401	ENSG00000169241	SLC50A1	filled
ENST00000368405	ENSG00000169241	SLC50A1	filled
ENST00000622581	ENSG00000169241	SLC50A1	filled
ENST00000561952	ENSG00000181938	GINS3	filled
ENST00000567432	ENSG00000181938	GINS3	
ENST00000567143	ENSG00000181938	GINS3	
ENST00000484757	ENSG00000228883	LY6G5C	
ENST00000481927	ENSG00000228883	LY6G5C	
ENST00000449871	ENSG00000228883	LY6G5C	
ENST00000330877	ENSG00000185100	ADSSL1	
ENST00000556623	ENSG00000185100	ADSSL1	
ENST00000546164	ENSG00000060138	YBX3	
ENST00000559265	ENSG00000255054	RP1-317E23.6	
ENST00000527604	ENSG00000255054	RP1-317E23.6	
ENST00000462733	ENSG00000171953	ATPAF2	
ENST00000551843	ENSG00000257242	C12orf79	
ENST00000549802	ENSG00000257242	C12orf79	
ENST00000552106	ENSG00000257242	C12orf79	
ENST00000551563	ENSG00000257242	C12orf79	
ENST00000546975	ENSG00000257242	C12orf79	
ENST00000551764	ENSG00000257242	C12orf79	
ENST00000611108	ENSG00000257242	C12orf79	
ENST00000463839	ENSG00000136156	ITM2B	
ENST00000524342	ENSG00000081059	TCF7	
ENST00000520699	ENSG00000081059	TCF7	
ENST00000367187	ENSG00000133056	PIK3C2B	filled
ENST00000424712	ENSG00000133056	PIK3C2B	filled
ENST00000582812	ENSG00000166579	NDEL1	
ENST00000579150	ENSG00000166579	NDEL1	
ENST00000580237	ENSG00000166579	NDEL1	
ENST00000581679	ENSG00000166579	NDEL1	
ENST00000420283	ENSG00000187189	TSPYL4	
ENST00000553824	ENSG00000129473	BCL2L2	
ENST00000373097	ENSG00000102290	PCDH11X	filled
ENST00000373094	ENSG00000102290	PCDH11X	filled
ENST00000373088	ENSG00000102290	PCDH11X	filled
ENST00000504220	ENSG00000102290	PCDH11X	filled
ENST00000361655	ENSG00000102290	PCDH11X	filled
ENST00000406881	ENSG00000102290	PCDH11X	filled
ENST00000486145	ENSG00000163689	C3orf67	filled
ENST00000373062	ENSG00000134697	GNL2	

ENST00000528190	ENSG00000137494	ANKRD42	filled
ENST00000587424	ENSG00000263465	SRSF8	
ENST00000414814	ENSG00000214021	TTLL3	
ENST00000496246	ENSG00000214021	TTLL3	filled
ENST00000383827	ENSG00000214021	TTLL3	filled
ENST00000473661	ENSG00000214021	TTLL3	filled
ENST00000478138	ENSG00000142856	ITGB3BP	filled
ENST00000292303	ENSG00000160791	CCR5	
ENST00000556234	ENSG00000185633	NDUFA4L2	
ENST00000557276	ENSG00000185633	NDUFA4L2	
ENST00000419693	ENSG00000106266	SNX8	
ENST00000281043	ENSG00000134323	MYCN	
ENST00000560626	ENSG00000173517	PEAK1	filled
ENST00000558305	ENSG00000173517	PEAK1	filled
ENST00000564328	ENSG00000173517	PEAK1	filled
ENST00000567808	ENSG00000173517	PEAK1	filled
ENST00000312493	ENSG00000173517	PEAK1	filled
ENST00000454607	ENSG00000272897	RP1-309K20.6	filled
ENST00000441563	ENSG00000272897	RP1-309K20.6	filled
ENST00000541176	ENSG00000272897	RP1-309K20.6	filled
ENST00000258888	ENSG00000136383	ALPK3	
ENST00000548514	ENSG00000111331	OAS3	filled
ENST00000549918	ENSG00000111331	OAS3	
ENST00000393240	ENSG00000067715	SYT1	filled
ENST00000261205	ENSG00000067715	SYT1	filled
ENST00000457153	ENSG00000067715	SYT1	filled
ENST00000552744	ENSG00000067715	SYT1	filled
ENST00000428314	ENSG00000169994	MYO7B	filled
ENST00000488428	ENSG00000082512	TRAF5	filled
ENST00000615388	ENSG00000133059	DSTYK	filled
ENST00000614034	ENSG00000275111	ZNF2	
ENST00000622059	ENSG00000275111	ZNF2	
ENST00000611147	ENSG00000275111	ZNF2	
ENST00000588224	ENSG00000165863	C10orf82	filled
ENST00000488788	ENSG00000270299	RP5-850E9.3	
ENST00000412431	ENSG00000113712	CSNK1A1	
ENST00000539841	ENSG00000149452	SLC22A8	
ENST00000552844	ENSG00000137962	ARHGAP29	filled
ENST00000513595	ENSG00000251493	FOXD1	
ENST00000512221	ENSG00000113555	PCDH12	
ENST00000614497	ENSG00000137726	FXYD6	filled
ENST00000382079	ENSG00000273294	C1QTNF3-AMACR	filled
ENST00000591995	ENSG00000187997	C17orf99	filled
ENST00000481846	ENSG00000163681	SLMAP	
ENST00000474301	ENSG00000106952	TNFSF8	
ENST00000618336	ENSG00000106952	TNFSF8	filled
ENST00000377346	ENSG00000171608	PIK3CD	filled
ENST00000536656	ENSG00000171608	PIK3CD	filled
ENST00000550404	ENSG00000076513	ANKRD13A	filled
ENST00000417209	ENSG00000253148	RGS21	filled
ENST00000043402	ENSG00000040608	RTN4R	
ENST00000425986	ENSG00000040608	RTN4R	
ENST00000469601	ENSG00000040608	RTN4R	
ENST00000369183	ENSG00000165669	FAM204A	
ENST00000409476	ENSG00000014641	MDH1	
ENST00000616342	ENSG00000166337	TAF10	
ENST00000299424	ENSG00000166337	TAF10	
ENST00000483718	ENSG00000183628	DGCR6	
ENST00000558973	ENSG00000134146	DPH6	filled
ENST00000602150	ENSG00000105711	SCN1B	filled

ENST00000563281	ENSG00000259956	RBM15B	
ENST00000551765	ENSG00000257218	GATC	
ENST00000589338	ENSG00000133247	SUV420H2	
ENST00000498738	ENSG00000133247	SUV420H2	
ENST00000466522	ENSG00000126790	L3HYPDH	filled
ENST00000331113	ENSG00000184408	KCND2	filled
ENST00000425288	ENSG00000184408	KCND2	
ENST00000473190	ENSG00000184408	KCND2	
ENST00000498349	ENSG00000243897	EGFL8	filled
ENST00000381962	ENSG00000271303	SRXN1	
ENST00000369295	ENSG00000043591	ADRB1	
ENST00000306917	ENSG00000169018	FEM1B	
ENST00000315683	ENSG00000176246	OR4L1	
ENST00000581486	ENSG00000266412	NCOA4	filled
ENST00000621654	ENSG00000273604	C17orf96	
ENST00000617811	ENSG00000275410	HNF1B	
ENST00000398355	ENSG00000046647	GEMIN8	
ENST00000398989	ENSG00000127824	TUBA4A	filled
ENST00000427737	ENSG00000127824	TUBA4A	filled
ENST00000311381	ENSG00000130349	C6orf203	
ENST00000520987	ENSG00000156509	FBXO43	
ENST00000621802	ENSG00000277611	RP1-138B7.6	filled
ENST00000620804	ENSG00000274600	RIMBP3B	filled
ENST00000472833	ENSG00000239463	HLA-DMA	
ENST00000462225	ENSG00000239463	HLA-DMA	
ENST00000546378	ENSG00000123388	HOXC11	filled
ENST00000559898	ENSG00000184508	HDDC3	
ENST00000376804	ENSG00000120942	UBIAD1	
ENST00000486588	ENSG00000120942	UBIAD1	
ENST00000483738	ENSG00000120942	UBIAD1	
ENST00000292729	ENSG00000161133	USP41	
ENST00000470202	ENSG00000161133	USP41	
ENST00000591645	ENSG00000167460	TPM4	
ENST00000526010	ENSG00000174080	CTSF	
ENST00000435303	ENSG00000173567	GPR113	filled
ENST00000333478	ENSG00000173567	GPR113	filled
ENST00000447444	ENSG00000173567	GPR113	filled
ENST00000421466	ENSG00000225553	PHF1	
ENST00000354556	ENSG00000175274	TP53I11	filled
ENST00000371374	ENSG00000172348	RCAN2	filled
ENST00000306764	ENSG00000172348	RCAN2	filled
ENST00000486132	ENSG00000126107	HECTD3	
ENST00000367882	ENSG00000118526	TCF21	
ENST00000237316	ENSG00000118526	TCF21	
ENST00000527336	ENSG00000023445	BIRC3	
ENST00000326035	ENSG00000177453	NIM1K	filled
ENST00000513797	ENSG00000177453	NIM1K	filled
ENST00000512796	ENSG00000177453	NIM1K	filled
ENST00000548946	ENSG00000177889	UBE2N	
ENST00000483270	ENSG00000116138	DNAJC16	filled
ENST00000268035	ENSG00000140443	IGF1R	filled
ENST00000558762	ENSG00000140443	IGF1R	filled
ENST00000559925	ENSG00000140443	IGF1R	filled
ENST00000558355	ENSG00000140443	IGF1R	filled
ENST00000440951	ENSG00000163399	ATP1A1	
ENST00000557727	ENSG00000100888	CHD8	
ENST00000557364	ENSG00000100888	CHD8	filled
ENST00000556833	ENSG00000100888	CHD8	filled
ENST00000483058	ENSG00000198704	GPX6	filled
ENST00000280098	ENSG00000144228	SPOPL	filled

ENST00000408938	ENSG00000221909	FAM200A	
ENST00000389093	ENSG00000067225	PKM	
ENST00000439286	ENSG00000136895	GARNL3	filled
ENST00000444677	ENSG00000136895	GARNL3	filled
ENST00000429629	ENSG00000136895	GARNL3	filled
ENST00000453030	ENSG00000136895	GARNL3	filled
ENST00000446764	ENSG00000136895	GARNL3	filled
ENST00000441134	ENSG00000136895	GARNL3	filled
ENST00000541267	ENSG00000111704	NANOG	
ENST00000526434	ENSG00000111704	NANOG	
ENST00000526286	ENSG00000111704	NANOG	
ENST00000453904	ENSG00000144445	KANSL1L	filled
ENST00000429908	ENSG00000144445	KANSL1L	filled
ENST00000526183	ENSG00000160972	PPP1R16A	filled
ENST00000526643	ENSG00000160972	PPP1R16A	
ENST00000292539	ENSG00000160972	PPP1R16A	filled
ENST00000529009	ENSG00000160972	PPP1R16A	filled
ENST00000314940	ENSG00000177733	HNRNPA0	
ENST00000225927	ENSG00000108784	NAGLU	
ENST00000586516	ENSG00000108784	NAGLU	
ENST00000591587	ENSG00000108784	NAGLU	
ENST00000590358	ENSG00000108784	NAGLU	
ENST00000592454	ENSG00000108784	NAGLU	
ENST00000301732	ENSG00000167972	ABCA3	filled
ENST00000382381	ENSG00000167972	ABCA3	filled
ENST00000563623	ENSG00000167972	ABCA3	filled
ENST00000299275	ENSG00000052126	PLEKHA5	
ENST00000539256	ENSG00000052126	PLEKHA5	
ENST00000560188	ENSG00000258484	SPESP1	filled
ENST00000523582	ENSG00000104321	TRPA1	filled
ENST00000468561	ENSG00000187017	ESPN	
ENST00000304418	ENSG00000169327	OR5AU1	
ENST00000502276	ENSG00000175857	GAPT	
ENST00000396776	ENSG00000175857	GAPT	
ENST00000511930	ENSG00000175857	GAPT	
ENST00000513924	ENSG00000175857	GAPT	
ENST00000511178	ENSG00000175857	GAPT	
ENST00000619056	ENSG00000275038	RP11-546B8.6	
ENST00000419358	ENSG00000164081	TEX264	filled
ENST00000373715	ENSG00000112081	SRSF3	filled
ENST00000426198	ENSG00000106608	URGCP	filled
ENST00000455877	ENSG00000106608	URGCP	filled
ENST00000477768	ENSG00000106608	URGCP	filled
ENST00000449440	ENSG00000197147	LRRC8B	
ENST00000330947	ENSG00000197147	LRRC8B	
ENST00000584294	ENSG00000101577	LPIN2	
ENST00000584915	ENSG00000101577	LPIN2	
ENST00000422861	ENSG00000185513	L3MBTL1	filled
ENST00000471977	ENSG00000185513	L3MBTL1	filled
ENST00000580402	ENSG00000197971	MBP	filled
ENST00000579129	ENSG00000197971	MBP	filled
ENST00000583798	ENSG00000197971	MBP	filled
ENST00000483195	ENSG00000188687	SLC4A5	filled
ENST00000394019	ENSG00000188687	SLC4A5	filled
ENST00000377634	ENSG00000188687	SLC4A5	filled
ENST00000436454	ENSG00000188687	SLC4A5	filled
ENST00000423644	ENSG00000188687	SLC4A5	filled
ENST00000346834	ENSG00000188687	SLC4A5	filled
ENST00000216144	ENSG00000100314	CABP7	filled
ENST00000310018	ENSG00000105929	ATP6V0A4	filled
ENST00000483139	ENSG00000105929	ATP6V0A4	filled
ENST00000231749	ENSG00000004838	ZMYND10	filled
ENST00000503024	ENSG00000164107	HAND2	
ENST00000576073	ENSG00000263310	SALL3	
ENST00000554824	ENSG00000259066	RP11-371E8.4	filled
ENST00000557048	ENSG00000259066	RP11-371E8.4	filled
ENST00000557574	ENSG00000259066	RP11-371E8.4	filled
ENST00000595016	ENSG00000105357	MYH14	filled
ENST00000449241	ENSG00000088543	C3orf18	
ENST00000464080	ENSG00000088543	C3orf18	
ENST00000546466	ENSG00000257727	CNPY2	
ENST00000563778	ENSG00000102879	CORO1A	
ENST00000561815	ENSG00000102879	CORO1A	
ENST00000552695	ENSG00000198855	FICD	
ENST00000549641	ENSG00000198855	FICD	
ENST00000546448	ENSG00000198855	FICD	
ENST00000532155	ENSG00000178795	GDPD4	
ENST00000416564	ENSG00000226201	PSMB8	filled
ENST00000264065	ENSG00000077232	DNAJC10	filled
ENST00000487583	ENSG00000062598	ELMO2	filled
ENST00000421201	ENSG00000171132	PRKCE	filled
ENST00000306156	ENSG00000171132	PRKCE	filled
ENST00000367188	ENSG00000158615	PPP1R15B	
ENST00000376730	ENSG00000187210	GCNT1	
ENST00000444201	ENSG00000187210	GCNT1	
ENST00000494780	ENSG00000196739	COL27A1	
ENST00000490831	ENSG00000196739	COL27A1	
ENST00000243706	ENSG00000214367	HAUS3	filled
ENST00000587368	ENSG00000167670	CHAF1A	
ENST00000460836	ENSG00000137098	SPAG8	
ENST00000463889	ENSG00000137098	SPAG8	
ENST00000340291	ENSG00000137098	SPAG8	
ENST00000475644	ENSG00000137098	SPAG8	
ENST00000489063	ENSG00000137098	SPAG8	
ENST00000620013	ENSG00000137098	SPAG8	
ENST00000522714	ENSG00000178338	ZNF354B	
ENST00000522624	ENSG00000178338	ZNF354B	
ENST00000484157	ENSG00000169241	SLC50A1	
ENST00000490770	ENSG00000169241	SLC50A1	
ENST00000475824	ENSG00000169241	SLC50A1	
ENST00000458073	ENSG00000100226	GTPBP1	
ENST00000462332	ENSG00000100226	GTPBP1	
ENST00000337432	ENSG00000108384	RAD51C	
ENST00000461706	ENSG00000108384	RAD51C	
ENST00000504942	ENSG00000176406	RIMS2	filled
ENST00000262231	ENSG00000176406	RIMS2	filled
ENST00000507740	ENSG00000176406	RIMS2	filled
ENST00000408894	ENSG00000176406	RIMS2	filled
ENST00000436393	ENSG00000176406	RIMS2	filled
ENST00000523362	ENSG00000176406	RIMS2	
ENST00000339750	ENSG00000176406	RIMS2	
ENST00000406091	ENSG00000176406	RIMS2	filled
ENST00000535916	ENSG00000155959	VBP1	filled
ENST00000307845	ENSG00000115756	HPCAL1	filled
ENST00000381765	ENSG00000115756	HPCAL1	filled
ENST00000257100	ENSG00000134668	SPOCD1	
ENST00000473361	ENSG00000134668	SPOCD1	
ENST00000452755	ENSG00000134668	SPOCD1	
ENST00000533231	ENSG00000134668	SPOCD1	
ENST00000531039	ENSG00000134668	SPOCD1	

ENST00000572686	ENSG00000121067	SPOP	
ENST00000570029	ENSG00000015413	DPEP1	
ENST00000565249	ENSG00000015413	DPEP1	filled
ENST00000581033	ENSG00000212901	KRTAP3-1	
ENST00000377981	ENSG00000168828	OR13J1	
ENST00000422904	ENSG00000240508	PSMB9	
ENST00000418861	ENSG00000240508	PSMB9	filled
ENST00000581993	ENSG00000125445	MRPS7	
ENST00000584678	ENSG00000125445	MRPS7	
ENST00000250111	ENSG00000129244	ATP1B2	
ENST00000461444	ENSG00000166263	STXBP4	filled
ENST00000369452	ENSG00000108061	SHOC2	filled
ENST00000489783	ENSG00000108061	SHOC2	filled
ENST00000459903	ENSG00000011454	RABGAP1	filled
ENST00000317419	ENSG00000011454	RABGAP1	filled
ENST00000402311	ENSG00000011454	RABGAP1	filled
ENST00000337343	ENSG00000102780	DGKH	filled
ENST00000261491	ENSG00000102780	DGKH	filled
ENST00000379274	ENSG00000102780	DGKH	filled
ENST00000551806	ENSG00000111780	AL021546.6	filled
ENST00000587120	ENSG00000136448	NMT1	
ENST00000587670	ENSG00000136448	NMT1	
ENST00000442141	ENSG00000138018	EPT1	filled
ENST00000260585	ENSG00000138018	EPT1	
ENST00000613142	ENSG00000138018	EPT1	
ENST00000486568	ENSG00000118855	MFSD1	filled
ENST00000524868	ENSG00000149534	MS4A2	
ENST00000515059	ENSG00000138696	BMPR1B	filled
ENST00000258457	ENSG00000135974	C2orf49	
ENST00000491445	ENSG00000136937	NCBP1	
ENST00000257868	ENSG00000135414	GDF11	
ENST00000546799	ENSG00000135414	GDF11	
ENST00000513019	ENSG00000013561	RNF14	filled
ENST00000356143	ENSG00000013561	RNF14	filled
ENST00000394519	ENSG00000013561	RNF14	filled
ENST00000367302	ENSG00000134369	NAV1	filled
ENST00000491403	ENSG00000134369	NAV1	
ENST00000367296	ENSG00000134369	NAV1	filled
ENST00000494292	ENSG00000085276	MECOM	filled
ENST00000481315	ENSG00000085276	MECOM	filled
ENST00000485957	ENSG00000085276	MECOM	filled
ENST00000486748	ENSG00000085276	MECOM	filled
ENST00000379441	ENSG00000076108	BAZ2A	filled
ENST00000551812	ENSG00000076108	BAZ2A	filled
ENST00000549884	ENSG00000076108	BAZ2A	filled
ENST00000515007	ENSG00000145685	LHFPL2	
ENST00000503686	ENSG00000145685	LHFPL2	filled
ENST00000512759	ENSG00000145685	LHFPL2	filled
ENST00000510949	ENSG00000145685	LHFPL2	
ENST00000380345	ENSG00000145685	LHFPL2	filled
ENST00000381906	ENSG00000130598	TNNI2	
ENST00000368771	ENSG00000186226	LCE1E	
ENST00000560807	ENSG00000140395	WDR61	
ENST00000298925	ENSG00000165973	NELL1	filled
ENST00000357134	ENSG00000165973	NELL1	filled
ENST00000532434	ENSG00000165973	NELL1	filled
ENST00000530672	ENSG00000165973	NELL1	filled
ENST00000325319	ENSG00000165973	NELL1	filled
ENST00000619031	ENSG00000165973	NELL1	filled
ENST00000577155	ENSG00000181045	SLC26A11	

ENST00000306954	ENSG00000170270	C14orf142	filled
ENST00000507401	ENSG00000144285	SCN1A	
ENST00000466239	ENSG00000241404	EGFL8	filled
ENST00000602526	ENSG00000106692	FKTN	filled
ENST00000551337	ENSG00000171759	PAH	filled
ENST00000546844	ENSG00000171759	PAH	
ENST00000547319	ENSG00000171759	PAH	
ENST00000550405	ENSG00000171759	PAH	
ENST00000596678	ENSG00000108474	PIGL	filled
ENST00000431149	ENSG00000108474	PIGL	
ENST00000422142	ENSG00000236279	CLEC2L	filled
ENST00000521281	ENSG00000236279	CLEC2L	filled
ENST00000520413	ENSG00000236279	CLEC2L	
ENST00000622836	ENSG00000240184	PCDHGC3	filled
ENST00000509864	ENSG00000123415	SMUG1	filled
ENST00000243112	ENSG00000123415	SMUG1	
ENST00000514196	ENSG00000123415	SMUG1	
ENST00000534114	ENSG00000118096	IFT46	
ENST00000528378	ENSG00000118096	IFT46	
ENST00000533918	ENSG00000118096	IFT46	
ENST00000586759	ENSG00000099860	GADD45B	
ENST00000215631	ENSG00000099860	GADD45B	
ENST00000520086	ENSG00000164270	HTR4	
ENST00000498807	ENSG00000153558	FBXL2	filled
ENST00000484457	ENSG00000153558	FBXL2	filled
ENST00000497411	ENSG00000153558	FBXL2	filled
ENST00000435207	ENSG00000153558	FBXL2	filled
ENST00000283627	ENSG00000153558	FBXL2	filled
ENST00000432809	ENSG00000153558	FBXL2	filled
ENST00000451636	ENSG00000153558	FBXL2	filled
ENST00000463736	ENSG00000153558	FBXL2	filled
ENST00000425973	ENSG00000153558	FBXL2	filled
ENST00000507198	ENSG00000153558	FBXL2	filled
ENST00000422741	ENSG00000153558	FBXL2	filled
ENST00000421391	ENSG00000153558	FBXL2	filled
ENST00000493778	ENSG00000153558	FBXL2	filled
ENST00000464164	ENSG00000153558	FBXL2	filled
ENST00000538892	ENSG00000153558	FBXL2	filled
ENST00000406028	ENSG00000184113	CLDN5	
ENST00000396573	ENSG00000183454	GRIN2A	filled
ENST00000562109	ENSG00000183454	GRIN2A	filled
ENST00000330684	ENSG00000183454	GRIN2A	filled
ENST00000307767	ENSG00000171928	TVP23B	filled
ENST00000574294	ENSG00000171928	TVP23B	filled
ENST00000571018	ENSG00000171928	TVP23B	filled
ENST00000574226	ENSG00000171928	TVP23B	filled
ENST00000575261	ENSG00000171928	TVP23B	filled
ENST00000298743	ENSG00000180447	GAS1	
ENST00000602430	ENSG00000164080	RAD54L2	
ENST00000440346	ENSG00000119383	PPP2R4	filled
ENST00000440863	ENSG00000140474	ULK3	
ENST00000566479	ENSG00000140474	ULK3	
ENST00000561725	ENSG00000140474	ULK3	
ENST00000569437	ENSG00000140474	ULK3	
ENST00000570276	ENSG00000140474	ULK3	
ENST00000567472	ENSG00000140474	ULK3	
ENST00000568718	ENSG00000140474	ULK3	
ENST00000583548	ENSG00000125450	NUP85	filled
ENST00000578987	ENSG00000125450	NUP85	
ENST00000579838	ENSG00000125450	NUP85	

ENST00000584155	ENSG00000125450	NUP85	
ENST00000441717	ENSG00000166086	JAM3	
ENST00000524969	ENSG00000166086	JAM3	
ENST00000216513	ENSG00000100625	SIX4	
ENST00000340433	ENSG00000187823	ZCCHC16	
ENST00000369774	ENSG00000107957	SH3PXD2A	filled
ENST00000355946	ENSG00000107957	SH3PXD2A	filled
ENST00000398148	ENSG00000088247	KHSRP	filled
ENST00000619396	ENSG00000088247	KHSRP	filled
ENST00000333141	ENSG00000182308	DCAF4L1	
ENST00000401450	ENSG00000100276	RASL10A	
ENST00000216101	ENSG00000100276	RASL10A	
ENST00000608559	ENSG00000100276	RASL10A	
ENST00000610883	ENSG00000274941	CNOT3	filled
ENST00000528644	ENSG00000070081	NUCB2	filled
ENST00000533773	ENSG00000070081	NUCB2	filled
ENST00000529010	ENSG00000070081	NUCB2	filled
ENST00000530964	ENSG00000070081	NUCB2	filled
ENST00000529313	ENSG00000070081	NUCB2	filled
ENST00000532240	ENSG00000070081	NUCB2	
ENST00000395116	ENSG00000181619	GPR135	filled
ENST00000581320	ENSG00000125434	SLC25A35	
ENST00000611864	ENSG00000182050	MGAT4C	
ENST00000621808	ENSG00000182050	MGAT4C	filled
ENST00000552808	ENSG00000182050	MGAT4C	filled
ENST00000547225	ENSG00000182050	MGAT4C	filled
ENST00000552435	ENSG00000182050	MGAT4C	filled
ENST00000550365	ENSG00000182050	MGAT4C	filled
ENST00000518753	ENSG00000265690	RP11-5A19.5	
ENST00000279392	ENSG00000149929	HIRIP3	
ENST00000383450	ENSG00000206412	GNL1	
ENST00000321256	ENSG00000114503	NCBP2	
ENST00000511657	ENSG00000121073	SLC35B1	
ENST00000274710	ENSG00000146005	PSD2	filled
ENST00000548143	ENSG00000185721	DRG1	filled
ENST00000382148	ENSG00000185774	KCNIP4	filled
ENST00000382150	ENSG00000185774	KCNIP4	filled
ENST00000509207	ENSG00000185774	KCNIP4	filled
ENST00000427369	ENSG00000184154	LRTOMT	filled
ENST00000544409	ENSG00000184154	LRTOMT	filled
ENST00000537483	ENSG00000184154	LRTOMT	
ENST00000307198	ENSG00000184154	LRTOMT	filled
ENST00000435085	ENSG00000184154	LRTOMT	filled
ENST00000419228	ENSG00000184154	LRTOMT	filled
ENST00000489408	ENSG00000197385	ZNF860	
ENST00000487694	ENSG00000185008	ROBO2	filled
ENST00000602589	ENSG00000185008	ROBO2	filled
ENST00000475034	ENSG00000185008	ROBO2	filled
ENST00000461745	ENSG00000185008	ROBO2	filled
ENST00000332191	ENSG00000185008	ROBO2	filled
ENST00000473767	ENSG00000185008	ROBO2	filled
ENST00000615701	ENSG00000262911	DNAJC28	
ENST00000431636	ENSG00000196345	ZKSCAN7	filled
ENST00000341840	ENSG00000196345	ZKSCAN7	filled
ENST00000380672	ENSG00000173068	BNC2	filled
ENST00000484726	ENSG00000173068	BNC2	filled
ENST00000380667	ENSG00000173068	BNC2	filled
ENST00000380666	ENSG00000173068	BNC2	filled
ENST00000486514	ENSG00000173068	BNC2	filled
ENST00000613349	ENSG00000173068	BNC2	filled
ENST00000617779	ENSG00000173068	BNC2	filled
ENST00000545497	ENSG00000173068	BNC2	filled
ENST00000548887	ENSG00000166987	MBD6	
ENST00000551351	ENSG00000166987	MBD6	
ENST00000549231	ENSG00000166987	MBD6	
ENST00000546805	ENSG00000166987	MBD6	
ENST00000552255	ENSG00000166987	MBD6	
ENST00000336798	ENSG00000162512	SDC3	filled
ENST00000339394	ENSG00000162512	SDC3	filled
ENST00000371485	ENSG00000138180	CEP55	filled
ENST00000445435	ENSG00000138180	CEP55	filled
ENST00000399096	ENSG00000188522	FAM83G	filled
ENST00000520959	ENSG00000175445	LPL	
ENST00000554107	ENSG00000119596	YLPM1	
ENST00000553381	ENSG00000119596	YLPM1	
ENST00000448864	ENSG00000182899	RPL35A	
ENST00000523893	ENSG00000135722	FBXL8	
ENST00000333274	ENSG00000184349	EFNA5	filled
ENST00000509503	ENSG00000184349	EFNA5	filled
ENST00000504941	ENSG00000184349	EFNA5	filled
ENST00000505499	ENSG00000184349	EFNA5	
ENST00000517434	ENSG00000253731	PCDHGA6	filled
ENST00000525971	ENSG00000133640	LRRIQ1	filled
ENST00000393217	ENSG00000133640	LRRIQ1	filled
ENST00000528777	ENSG00000133640	LRRIQ1	filled
ENST00000526363	ENSG00000133640	LRRIQ1	filled
ENST00000548559	ENSG00000004455	AK2	
ENST00000517913	ENSG00000170624	SGCD	filled
ENST00000394697	ENSG00000121281	ADCY7	filled
ENST00000566433	ENSG00000121281	ADCY7	filled
ENST00000537625	ENSG00000084444	KIAA1467	filled
ENST00000434467	ENSG00000180376	CCDC66	filled
ENST00000484623	ENSG00000180376	CCDC66	
ENST00000376477	ENSG00000102057	KCND1	filled
ENST00000218176	ENSG00000102057	KCND1	filled
ENST00000565267	ENSG00000103342	GSPT1	
ENST00000266673	ENSG00000139291	TMEM19	
ENST00000283916	ENSG00000153802	TMPRSS11D	filled
ENST00000502573	ENSG00000153802	TMPRSS11D	filled
ENST00000509584	ENSG00000153802	TMPRSS11D	filled
ENST00000502822	ENSG00000153802	TMPRSS11D	filled
ENST00000504508	ENSG00000123219	CENPK	
ENST00000488058	ENSG00000137075	RNF38	filled
ENST00000466652	ENSG00000143162	CREG1	
ENST00000270792	ENSG00000142669	SH3BGRL3	
ENST00000614679	ENSG00000142669	SH3BGRL3	
ENST00000216237	ENSG00000100395	L3MBTL2	filled
ENST00000449635	ENSG00000100395	L3MBTL2	filled
ENST00000523898	ENSG00000104626	ERI1	
ENST00000519292	ENSG00000104626	ERI1	
ENST00000520332	ENSG00000104626	ERI1	filled
ENST00000518663	ENSG00000104626	ERI1	filled
ENST00000522612	ENSG00000104626	ERI1	filled
ENST00000250263	ENSG00000104626	ERI1	
ENST00000449191	ENSG00000258984	UBE2F-SCLY	filled
ENST00000489288	ENSG00000171792	RHNO1	
ENST00000623153	ENSG00000171792	RHNO1	
ENST00000522943	ENSG00000145826	LECT2	filled
ENST00000471827	ENSG00000145826	LECT2	filled
ENST00000524196	ENSG00000143801	PSEN2	

ENST00000366783	ENSG00000143801	PSEN2	
ENST00000495488	ENSG00000143801	PSEN2	
ENST00000422240	ENSG00000143801	PSEN2	
ENST00000521431	ENSG00000143801	PSEN2	
ENST00000460775	ENSG00000143801	PSEN2	
ENST00000366782	ENSG00000143801	PSEN2	
ENST00000468814	ENSG00000254778	XXbac-BCX105D18.9	
ENST00000337019	ENSG00000112530	PACRG	filled
ENST00000366889	ENSG00000112530	PACRG	filled
ENST00000366888	ENSG00000112530	PACRG	filled
ENST00000542936	ENSG00000112530	PACRG	
ENST00000611387	ENSG00000112530	PACRG	
ENST00000546651	ENSG00000174456	C12orf76	
ENST00000309050	ENSG00000174456	C12orf76	filled
ENST00000548936	ENSG00000174456	C12orf76	filled
ENST00000601146	ENSG00000105559	PLEKHA4	
ENST00000378105	ENSG00000204956	PCDHGA1	
ENST00000517417	ENSG00000204956	PCDHGA1	filled
ENST00000370453	ENSG00000271949	RP11-302M6.4	filled
ENST00000468818	ENSG00000116459	ATP5F1	
ENST00000479219	ENSG00000135093	USP30	
ENST00000470117	ENSG00000135093	USP30	
ENST00000360655	ENSG00000166833	NAV2	filled
ENST00000327739	ENSG00000173124	ACSM6	filled
ENST00000404473	ENSG00000173124	ACSM6	filled
ENST00000394005	ENSG00000173124	ACSM6	filled
ENST00000341686	ENSG00000173124	ACSM6	filled
ENST00000619918	ENSG00000275793	RIMBP3	filled
ENST00000528209	ENSG00000185187	SIGIRR	
ENST00000525299	ENSG00000185187	SIGIRR	
ENST00000528698	ENSG00000185187	SIGIRR	
ENST00000534217	ENSG00000185187	SIGIRR	
ENST00000530494	ENSG00000185187	SIGIRR	
ENST00000529486	ENSG00000185187	SIGIRR	
ENST00000530683	ENSG00000185187	SIGIRR	
ENST00000528116	ENSG00000185187	SIGIRR	
ENST00000527136	ENSG00000185187	SIGIRR	
ENST00000613128	ENSG00000125813	PAX1	
ENST00000565314	ENSG00000205336	GPR56	filled
ENST00000564912	ENSG00000205336	GPR56	filled
ENST00000568909	ENSG00000205336	GPR56	filled
ENST00000561872	ENSG00000205336	GPR56	filled
ENST00000561988	ENSG00000205336	GPR56	filled
ENST00000569372	ENSG00000205336	GPR56	filled
ENST00000562003	ENSG00000205336	GPR56	filled
ENST00000568487	ENSG00000205336	GPR56	
ENST00000540164	ENSG00000205336	GPR56	filled
ENST00000453487	ENSG00000225987	PBX2	filled
ENST00000529844	ENSG00000254469	RP11-849H4.2	
ENST00000533047	ENSG00000254469	RP11-849H4.2	
ENST00000534704	ENSG00000254469	RP11-849H4.2	
ENST00000528184	ENSG00000254469	RP11-849H4.2	
ENST00000531488	ENSG00000254469	RP11-849H4.2	
ENST00000437006	ENSG00000084693	AGBL5	
ENST00000216034	ENSG00000100216	TOMM22	
ENST00000531537	ENSG00000178685	PARP10	
ENST00000529842	ENSG00000178685	PARP10	
ENST00000530478	ENSG00000178685	PARP10	
ENST00000531707	ENSG00000178685	PARP10	
ENST00000493855	ENSG00000146731	CCT6A	

ENST00000513000	ENSG00000109452	INPP4B	filled
ENST00000509777	ENSG00000109452	INPP4B	filled
ENST00000506788	ENSG00000109452	INPP4B	filled
ENST00000503927	ENSG00000109452	INPP4B	filled
ENST00000508084	ENSG00000109452	INPP4B	
ENST00000369041	ENSG00000143382	ADAMTSL4	
ENST00000271643	ENSG00000143382	ADAMTSL4	
ENST00000369039	ENSG00000143382	ADAMTSL4	
ENST00000433197	ENSG00000178607	ERN1	filled
ENST00000584041	ENSG00000178607	ERN1	filled
ENST00000583028	ENSG00000178607	ERN1	
ENST00000479717	ENSG00000121380	BCL2L14	
ENST00000466531	ENSG00000121380	BCL2L14	
ENST00000308721	ENSG00000121380	BCL2L14	
ENST00000396367	ENSG00000121380	BCL2L14	
ENST00000370996	ENSG00000132825	PPP1R3D	
ENST00000579632	ENSG00000108465	CDK5RAP3	
ENST00000379915	ENSG00000260729	RP11-106M3.2	filled
ENST00000527920	ENSG00000173465	SSSCA1	
ENST00000533115	ENSG00000173465	SSSCA1	
ENST00000526433	ENSG00000173465	SSSCA1	
ENST00000357186	ENSG00000115204	MPV17	filled
ENST00000412106	ENSG00000225073	DDX39B	filled
ENST00000403045	ENSG00000075340	ADD2	
ENST00000367001	ENSG00000170385	SLC30A1	
ENST00000332438	ENSG00000184451	CCR10	
ENST00000591765	ENSG00000184451	CCR10	
ENST00000591568	ENSG00000184451	CCR10	
ENST00000504334	ENSG00000006282	SPATA20	
ENST00000607093	ENSG00000197084	LCE1C	
ENST00000479173	ENSG00000144645	OSBPL10	filled
ENST00000575332	ENSG00000006194	ZNF263	
ENST00000574674	ENSG00000006194	ZNF263	
ENST00000426928	ENSG00000215906	LACTBL1	filled
ENST00000573759	ENSG00000167842	MIS12	
ENST00000381165	ENSG00000167842	MIS12	
ENST00000611091	ENSG00000167842	MIS12	
ENST00000316045	ENSG00000177853	ZNF518A	
ENST00000488700	ENSG00000177853	ZNF518A	
ENST00000539666	ENSG00000177853	ZNF518A	
ENST00000478086	ENSG00000177853	ZNF518A	
ENST00000563195	ENSG00000177853	ZNF518A	
ENST00000484770	ENSG00000177853	ZNF518A	
ENST00000534948	ENSG00000177853	ZNF518A	
ENST00000442635	ENSG00000177853	ZNF518A	
ENST00000624776	ENSG00000177853	ZNF518A	
ENST00000614149	ENSG00000177853	ZNF518A	
ENST00000417761	ENSG00000262560	RP11-296A16.1	filled
ENST00000476242	ENSG00000231325	LY6G5C	
ENST00000473902	ENSG00000231325	LY6G5C	
ENST00000411492	ENSG00000231325	LY6G5C	
ENST00000426290	ENSG00000137218	FRS3	
ENST00000380785	ENSG00000101871	MID1	filled
ENST00000380787	ENSG00000101871	MID1	filled
ENST00000495027	ENSG00000174844	DNAH12	filled
ENST00000351747	ENSG00000174844	DNAH12	filled
ENST00000469090	ENSG00000167549	CORO6	
ENST00000579388	ENSG00000167549	CORO6	
ENST00000617313	ENSG00000177692	DNAJC28	
ENST00000428443	ENSG00000187231	SESTD1	

ENST00000440010	ENSG00000187231	SESTD1	
ENST00000435047	ENSG00000187231	SESTD1	
ENST00000463281	ENSG00000075651	PLD1	
ENST00000358859	ENSG00000197651	CCER1	
ENST00000549707	ENSG00000197651	CCER1	
ENST00000407149	ENSG00000184838	PRR16	filled
ENST00000379551	ENSG00000184838	PRR16	filled
ENST00000509923	ENSG00000184838	PRR16	filled
ENST00000567339	ENSG00000103365	GGA2	
ENST00000566685	ENSG00000103365	GGA2	
ENST00000256389	ENSG00000134007	ADAM20	
ENST00000442510	ENSG00000081237	PTPRC	filled
ENST00000348564	ENSG00000081237	PTPRC	filled
ENST00000382723	ENSG00000163132	MSX1	
ENST00000529857	ENSG00000172732	MUS81	
ENST00000302513	ENSG00000273045	C2orf15	filled
ENST00000409684	ENSG00000273045	C2orf15	
ENST00000496794	ENSG00000273045	C2orf15	
ENST00000311089	ENSG00000119661	DNAL1	filled
ENST00000464177	ENSG00000157600	TMEM164	
ENST00000485577	ENSG00000048405	ZNF800	filled
ENST00000439506	ENSG00000048405	ZNF800	
ENST00000578826	ENSG00000188895	MSL1	filled
ENST00000535644	ENSG00000172613	RAD9A	
ENST00000604036	ENSG00000188352	FOCAD	
ENST00000431696	ENSG00000254706	RP11-565P22.6	
ENST00000367932	ENSG00000254706	RP11-565P22.6	
ENST00000389622	ENSG00000080200	CRYBG3	filled
ENST00000403006	ENSG00000118961	C2orf43	filled
ENST00000381090	ENSG00000118961	C2orf43	filled
ENST00000470099	ENSG00000118961	C2orf43	filled
ENST00000237822	ENSG00000118961	C2orf43	filled
ENST00000432947	ENSG00000118961	C2orf43	
ENST00000412261	ENSG00000118961	C2orf43	
ENST00000440866	ENSG00000118961	C2orf43	filled
ENST00000619656	ENSG00000118961	C2orf43	filled
ENST00000435420	ENSG00000118961	C2orf43	filled
ENST00000541941	ENSG00000118961	C2orf43	filled
ENST00000621735	ENSG00000183035	CYLC1	
ENST00000280979	ENSG00000151320	AKAP6	filled
ENST00000557354	ENSG00000151320	AKAP6	filled
ENST00000557102	ENSG00000151320	AKAP6	filled
ENST00000556037	ENSG00000151320	AKAP6	filled
ENST00000557272	ENSG00000151320	AKAP6	filled
ENST00000554449	ENSG00000151320	AKAP6	
ENST00000553547	ENSG00000151320	AKAP6	filled
ENST00000554410	ENSG00000151320	AKAP6	filled
ENST00000556638	ENSG00000151320	AKAP6	filled
ENST00000420220	ENSG00000239887	C1orf226	
ENST00000458626	ENSG00000239887	C1orf226	
ENST00000354186	ENSG00000160145	KALRN	filled
ENST00000393496	ENSG00000160145	KALRN	filled
ENST00000291478	ENSG00000160145	KALRN	filled
ENST00000360013	ENSG00000160145	KALRN	filled
ENST00000261383	ENSG00000158486	DNAH3	filled
ENST00000379751	ENSG00000125817	CENPB	
ENST00000523207	ENSG00000182674	KCNB2	filled
ENST00000504199	ENSG00000145321	GC	
ENST00000469099	ENSG00000185055	EFCAB10	filled
ENST00000400522	ENSG00000215529	EFCAB8	

ENST00000508440	ENSG00000242441	GTF2A1L	filled
ENST00000569508	ENSG00000149926	FAM57B	filled
ENST00000561666	ENSG00000149926	FAM57B	filled
ENST00000244769	ENSG00000124788	ATXN1	filled
ENST00000483591	ENSG00000124788	ATXN1	filled
ENST00000473388	ENSG00000124788	ATXN1	filled
ENST00000436367	ENSG00000124788	ATXN1	filled
ENST00000371095	ENSG00000087460	GNAS	
ENST00000371085	ENSG00000087460	GNAS	
ENST00000354359	ENSG00000087460	GNAS	
ENST00000265620	ENSG00000087460	GNAS	
ENST00000391946	ENSG00000104892	KLC3	
ENST00000590063	ENSG00000104892	KLC3	
ENST00000251038	ENSG00000100722	ZC3H14	filled
ENST00000610164	ENSG00000164604	GPR85	
ENST00000433855	ENSG00000130035	GALNT8	filled
ENST00000606868	ENSG00000146243	IRAK1BP1	filled
ENST00000606929	ENSG00000146243	IRAK1BP1	
ENST00000582124	ENSG00000170425	ADORA2B	
ENST00000482150	ENSG00000109787	KLF3	filled
ENST00000450169	ENSG00000106355	LSM5	
ENST00000468872	ENSG00000106355	LSM5	
ENST00000377748	ENSG00000171680	PLEKHG5	filled
ENST00000377740	ENSG00000171680	PLEKHG5	filled
ENST00000573079	ENSG00000262795	IFNGR2	
ENST00000571550	ENSG00000262795	IFNGR2	
ENST00000576463	ENSG00000262795	IFNGR2	
ENST00000475831	ENSG00000164900	GBX1	filled
ENST00000547260	ENSG00000174233	ADCY6	
ENST00000550422	ENSG00000174233	ADCY6	
ENST00000307885	ENSG00000174233	ADCY6	filled
ENST00000533589	ENSG00000166135	HIF1AN	
ENST00000299163	ENSG00000166135	HIF1AN	
ENST00000381120	ENSG00000122033	MTIF3	
ENST00000461838	ENSG00000122033	MTIF3	
ENST00000471771	ENSG00000122033	MTIF3	
ENST00000483903	ENSG00000122033	MTIF3	
ENST00000544217	ENSG00000051825	MPHOSPH9	
ENST00000541603	ENSG00000051825	MPHOSPH9	
ENST00000539024	ENSG00000051825	MPHOSPH9	filled
ENST00000544708	ENSG00000256374	PPIAL4D	
ENST00000421999	ENSG00000184220	CMSS1	filled
ENST00000496116	ENSG00000184220	CMSS1	filled
ENST00000489081	ENSG00000184220	CMSS1	filled
ENST00000408899	ENSG00000221938	OR2A14	
ENST00000522837	ENSG00000133943	C14orf159	filled
ENST00000533786	ENSG00000157570	TSPAN18	filled
ENST00000533202	ENSG00000157570	TSPAN18	filled
ENST00000533080	ENSG00000157570	TSPAN18	filled
ENST00000520358	ENSG00000157570	TSPAN18	filled
ENST00000520999	ENSG00000157570	TSPAN18	filled
ENST00000340160	ENSG00000157570	TSPAN18	filled
ENST00000534878	ENSG00000167985	SDHAF2	filled
ENST00000317799	ENSG00000138029	HADHB	
ENST00000405867	ENSG00000138029	HADHB	
ENST00000545822	ENSG00000138029	HADHB	
ENST00000537713	ENSG00000138029	HADHB	
ENST00000619635	ENSG00000183837	PNMA3	
ENST00000424805	ENSG00000183837	PNMA3	
ENST00000570576	ENSG00000141505	ASGR1	

ENST00000573083	ENSG00000141505	ASGR1	
ENST00000373010	ENSG00000164663	USP49	filled
ENST00000373006	ENSG00000164663	USP49	filled
ENST00000423567	ENSG00000164663	USP49	filled
ENST00000418087	ENSG00000075651	PLD1	filled
ENST00000259953	ENSG00000137404	NRM	
ENST00000338121	ENSG00000125812	GZF1	
ENST00000377051	ENSG00000125812	GZF1	
ENST00000316836	ENSG00000176903	PNMA1	
ENST00000332822	ENSG00000183691	NOG	
ENST00000244745	ENSG00000124766	SOX4	filled
ENST00000443535	ENSG00000170734	POLH	
ENST00000369878	ENSG00000148842	CNNM2	filled
ENST00000433628	ENSG00000148842	CNNM2	filled
ENST00000520464	ENSG00000164463	CREBRF	filled
ENST00000565324	ENSG00000157106	SMG1	filled
ENST00000374207	ENSG00000176092	AIM1L	
ENST00000475866	ENSG00000176092	AIM1L	
ENST00000308182	ENSG00000176092	AIM1L	
ENST00000535561	ENSG00000112096	SOD2	
ENST00000401980	ENSG00000112096	SOD2	
ENST00000461790	ENSG00000198771	RCSD1	filled
ENST00000543494	ENSG00000256514	AP003419.11	filled
ENST00000413473	ENSG00000085831	TTC39A	filled
ENST00000262675	ENSG00000085831	TTC39A	filled
ENST00000551733	ENSG00000086159	AQP6	filled
ENST00000615425	ENSG00000086159	AQP6	
ENST00000569167	ENSG00000159625	DRC7	
ENST00000594664	ENSG00000268643	AC006486.9	filled
ENST00000523234	ENSG00000155189	AGPAT5	
ENST00000557850	ENSG00000051180	RAD51	
ENST00000525066	ENSG00000051180	RAD51	
ENST00000522468	ENSG00000147421	HMBOX1	
ENST00000561377	ENSG00000147421	HMBOX1	
ENST00000330909	ENSG00000151276	MAGI1	filled
ENST00000402939	ENSG00000151276	MAGI1	filled
ENST00000483466	ENSG00000151276	MAGI1	filled
ENST00000460329	ENSG00000151276	MAGI1	filled
ENST00000497477	ENSG00000151276	MAGI1	filled
ENST00000470990	ENSG00000151276	MAGI1	filled
ENST00000464060	ENSG00000151276	MAGI1	filled
ENST00000476403	ENSG00000151276	MAGI1	filled
ENST00000611645	ENSG00000151276	MAGI1	filled
ENST00000613799	ENSG00000151276	MAGI1	filled
ENST00000621418	ENSG00000151276	MAGI1	filled
ENST00000596957	ENSG00000076928	ARHGEF1	filled
ENST00000599589	ENSG00000076928	ARHGEF1	
ENST00000481729	ENSG00000135968	GCC2	filled
ENST00000426851	ENSG00000197024	ZNF398	filled
ENST00000589046	ENSG00000251247	ZNF345	filled
ENST00000420450	ENSG00000251247	ZNF345	filled
ENST00000529555	ENSG00000251247	ZNF345	filled
ENST00000331800	ENSG00000251247	ZNF345	filled
ENST00000585396	ENSG00000251247	ZNF345	filled
ENST00000612719	ENSG00000251247	ZNF345	filled
ENST00000491413	ENSG00000151693	ASAP2	
ENST00000484590	ENSG00000151693	ASAP2	filled
ENST00000509602	ENSG00000153113	CAST	
ENST00000510098	ENSG00000153113	CAST	
ENST00000325870	ENSG00000176399	DMRTA1	

ENST00000506189	ENSG00000047365	ARAP2	
ENST00000543826	ENSG00000111452	GPR133	
ENST00000542091	ENSG00000111452	GPR133	filled
ENST00000416247	ENSG00000224531	SMIM13	filled
ENST00000376935	ENSG00000224531	SMIM13	filled
ENST00000464322	ENSG00000117724	CENPF	
ENST00000495259	ENSG00000117724	CENPF	
ENST00000469862	ENSG00000117724	CENPF	
ENST00000492904	ENSG00000238104	PPP1R10	
ENST00000432850	ENSG00000151692	RNF144A	filled
ENST00000512239	ENSG00000113356	POLR3G	
ENST00000504930	ENSG00000113356	POLR3G	
ENST00000399107	ENSG00000113356	POLR3G	
ENST00000549964	ENSG00000135473	PAN2	
ENST00000423427	ENSG00000169760	NLGN1	
ENST00000413821	ENSG00000169760	NLGN1	
ENST00000457714	ENSG00000169760	NLGN1	filled
ENST00000361589	ENSG00000169760	NLGN1	filled
ENST00000415045	ENSG00000169760	NLGN1	filled
ENST00000466350	ENSG00000169760	NLGN1	filled
ENST00000469564	ENSG00000169760	NLGN1	filled
ENST00000490929	ENSG00000169760	NLGN1	filled
ENST00000469727	ENSG00000169760	NLGN1	filled
ENST00000599565	ENSG00000198300	PEG3	
ENST00000543163	ENSG00000158104	HPD	filled
ENST00000323523	ENSG00000181458	TMEM45A	filled
ENST00000403410	ENSG00000181458	TMEM45A	filled
ENST00000449609	ENSG00000181458	TMEM45A	filled
ENST00000343139	ENSG00000196735	HLA-DQA1	filled
ENST00000395363	ENSG00000196735	HLA-DQA1	filled
ENST00000482745	ENSG00000196735	HLA-DQA1	filled
ENST00000487768	ENSG00000238056	PRRT1	
ENST00000494041	ENSG00000238056	PRRT1	
ENST00000463986	ENSG00000238056	PRRT1	
ENST00000587807	ENSG00000091164	TXNL1	filled
ENST00000587613	ENSG00000091164	TXNL1	filled
ENST00000586079	ENSG00000091164	TXNL1	
ENST00000496736	ENSG00000057019	DCBLD2	filled
ENST00000405510	ENSG00000100242	SUN2	filled
ENST00000406622	ENSG00000100242	SUN2	filled
ENST00000505124	ENSG00000164118	CEP44	
ENST00000514712	ENSG00000164118	CEP44	
ENST00000515299	ENSG00000164118	CEP44	
ENST00000522278	ENSG00000134020	PEBP4	filled
ENST00000547525	ENSG00000075035	WSCD2	filled
ENST00000552195	ENSG00000075035	WSCD2	
ENST00000550529	ENSG00000075035	WSCD2	
ENST00000551638	ENSG00000075035	WSCD2	filled
ENST00000332082	ENSG00000075035	WSCD2	filled
ENST00000546811	ENSG00000075035	WSCD2	
ENST00000266078	ENSG00000203880	PCMTD2	
ENST00000256246	ENSG00000133863	TEX15	
ENST00000523186	ENSG00000133863	TEX15	filled
ENST00000614835	ENSG00000133863	TEX15	filled
ENST00000571350	ENSG00000262666	FAM189B	filled
ENST00000526364	ENSG00000134910	STT3A	filled
ENST00000297679	ENSG00000099377	HSD3B7	filled
ENST00000459684	ENSG00000206208	TAPBP	
ENST00000484848	ENSG00000075826	SEC31B	filled
ENST00000430099	ENSG00000234154	HLA-DMB	filled

ENST00000607427	ENSG00000163131	CTSS	
ENST00000331898	ENSG00000183715	OPCML	filled
ENST00000524381	ENSG00000183715	OPCML	filled
ENST00000374778	ENSG00000183715	OPCML	filled
ENST00000525412	ENSG00000183715	OPCML	filled
ENST00000529019	ENSG00000183715	OPCML	filled
ENST00000541867	ENSG00000183715	OPCML	filled
ENST00000612177	ENSG00000183715	OPCML	filled
ENST00000472091	ENSG00000244476	ERVFRD-1	
ENST00000476041	ENSG00000181817	LSM10	filled
ENST00000315732	ENSG00000181817	LSM10	filled
ENST00000489912	ENSG00000181817	LSM10	
ENST00000323061	ENSG00000177432	NAP1L5	
ENST00000334293	ENSG00000161082	CELF5	filled
ENST00000589370	ENSG00000161082	CELF5	filled
ENST00000469190	ENSG00000198753	PLXNB3	
ENST00000472415	ENSG00000198753	PLXNB3	
ENST00000410066	ENSG00000144331	ZNF385B	filled
ENST00000451732	ENSG00000144331	ZNF385B	filled
ENST00000442114	ENSG00000181577	C6orf223	
ENST00000336600	ENSG00000181577	C6orf223	
ENST00000439969	ENSG00000181577	C6orf223	
ENST00000452499	ENSG00000157992	KRTCAP3	
ENST00000543753	ENSG00000157992	KRTCAP3	
ENST00000495706	ENSG00000157593	SLC35B2	
ENST00000393810	ENSG00000157593	SLC35B2	
ENST00000393812	ENSG00000157593	SLC35B2	
ENST00000538577	ENSG00000157593	SLC35B2	
ENST00000619636	ENSG00000157593	SLC35B2	
ENST00000615337	ENSG00000157593	SLC35B2	
ENST00000537814	ENSG00000157593	SLC35B2	
ENST00000543979	ENSG00000255639	RP11-234B24.6	filled
ENST00000544741	ENSG00000255639	RP11-234B24.6	
ENST00000614211	ENSG00000274429	DLG5	filled
ENST00000559763	ENSG00000185088	RPS27L	filled
ENST00000370150	ENSG00000130822	PNCK	
ENST00000466074	ENSG00000130822	PNCK	
ENST00000438984	ENSG00000130822	PNCK	
ENST00000472324	ENSG00000130822	PNCK	
ENST00000370142	ENSG00000130822	PNCK	
ENST00000488994	ENSG00000130822	PNCK	filled
ENST00000422811	ENSG00000130822	PNCK	
ENST00000475172	ENSG00000130822	PNCK	
ENST00000465303	ENSG00000130822	PNCK	
ENST00000466662	ENSG00000130822	PNCK	
ENST00000415927	ENSG00000239329	HLA-DMB	filled
ENST00000536551	ENSG00000230873	STMND1	
ENST00000447566	ENSG00000100372	SLC25A17	
ENST00000420970	ENSG00000100372	SLC25A17	
ENST00000430221	ENSG00000100372	SLC25A17	
ENST00000427084	ENSG00000100372	SLC25A17	
ENST00000458600	ENSG00000100372	SLC25A17	
ENST00000443810	ENSG00000100372	SLC25A17	
ENST00000434193	ENSG00000100372	SLC25A17	
ENST00000449676	ENSG00000100372	SLC25A17	
ENST00000434185	ENSG00000100372	SLC25A17	
ENST00000441808	ENSG00000147996	CBWD5	
ENST00000496364	ENSG00000147996	CBWD5	
ENST00000521150	ENSG00000184347	SLIT3	filled
ENST00000521130	ENSG00000184347	SLIT3	filled

ENST00000445067	ENSG00000064886	CHI3L2	
ENST00000409457	ENSG00000157985	AGAP1	filled
ENST00000336665	ENSG00000157985	AGAP1	filled
ENST00000304032	ENSG00000157985	AGAP1	filled
ENST00000402604	ENSG00000157985	AGAP1	filled
ENST00000359707	ENSG00000138757	G3BP2	
ENST00000511868	ENSG00000138757	G3BP2	filled
ENST00000509561	ENSG00000138757	G3BP2	filled
ENST00000508510	ENSG00000138757	G3BP2	filled
ENST00000499709	ENSG00000138757	G3BP2	filled
ENST00000577298	ENSG00000263961	C1orf186	
ENST00000486669	ENSG00000227804	PPP1R10	
ENST00000573882	ENSG00000141527	CARD14	filled
ENST00000570421	ENSG00000141527	CARD14	
ENST00000400549	ENSG00000215545	DEFB116	
ENST00000520468	ENSG00000164919	COX6C	filled
ENST00000606245	ENSG00000164919	COX6C	
ENST00000380060	ENSG00000188158	NHS	filled
ENST00000525801	ENSG00000149489	ROM1	
ENST00000534093	ENSG00000149489	ROM1	
ENST00000529273	ENSG00000149489	ROM1	
ENST00000300150	ENSG00000166900	STX3	filled
ENST00000469194	ENSG00000117228	GBP1	
ENST00000601319	ENSG00000138641	HERC3	
ENST00000407637	ENSG00000138641	HERC3	filled
ENST00000452979	ENSG00000138641	HERC3	filled
ENST00000555447	ENSG00000140022	STON2	filled
ENST00000553821	ENSG00000140022	STON2	
ENST00000555226	ENSG00000140022	STON2	filled
ENST00000554710	ENSG00000140022	STON2	filled
ENST00000571457	ENSG00000176108	CHMP6	filled
ENST00000424491	ENSG00000241962	C2orf15	filled
ENST00000368318	ENSG00000173080	RXFP4	
ENST00000430073	ENSG00000228662	MCCD1	
ENST00000617050	ENSG00000253537	PCDHGA7	
ENST00000518325	ENSG00000253537	PCDHGA7	filled
ENST00000483634	ENSG00000124532	MRS2	filled
ENST00000555917	ENSG00000198732	SMOC1	filled
ENST00000532355	ENSG00000140798	ABCC12	
ENST00000526251	ENSG00000140798	ABCC12	
ENST00000533185	ENSG00000140798	ABCC12	
ENST00000582126	ENSG00000108424	KPNB1	
ENST00000506123	ENSG00000164294	GPX8	
ENST00000296734	ENSG00000164294	GPX8	
ENST00000373019	ENSG00000183431	SF3A3	filled
ENST00000581598	ENSG00000161973	CCDC42	
ENST00000624695	ENSG00000205174	C7orf66	
ENST00000379007	ENSG00000205174	C7orf66	
ENST00000489551	ENSG00000119535	CSF3R	
ENST00000473144	ENSG00000143512	HHIPL2	
ENST00000342350	ENSG00000172992	DCAKD	filled
ENST00000310604	ENSG00000172992	DCAKD	filled
ENST00000223026	ENSG00000106302	HYAL4	
ENST00000476325	ENSG00000106302	HYAL4	
ENST00000477813	ENSG00000121579	NAA50	
ENST00000611252	ENSG00000275979	CNOT3	filled
ENST00000529618	ENSG00000189057	FAM111B	
ENST00000509176	ENSG00000184305	CCSER1	filled
ENST00000568221	ENSG00000099769	IGFALS	
ENST00000333396	ENSG00000170854	MINA	

ENST00000506099	ENSG00000170854	MINA	
ENST00000479606	ENSG00000099949	LZTR1	
ENST00000215739	ENSG00000099949	LZTR1	
ENST00000414985	ENSG00000099949	LZTR1	
ENST00000493460	ENSG00000099949	LZTR1	
ENST00000443265	ENSG00000099949	LZTR1	
ENST00000480895	ENSG00000099949	LZTR1	
ENST00000497716	ENSG00000099949	LZTR1	
ENST00000461510	ENSG00000099949	LZTR1	
ENST00000492480	ENSG00000099949	LZTR1	
ENST00000415354	ENSG00000099949	LZTR1	
ENST00000495142	ENSG00000099949	LZTR1	
ENST00000491432	ENSG00000099949	LZTR1	
ENST00000439171	ENSG00000099949	LZTR1	
ENST00000464807	ENSG00000099949	LZTR1	
ENST00000452988	ENSG00000099949	LZTR1	
ENST00000498649	ENSG00000099949	LZTR1	
ENST00000389355	ENSG00000099949	LZTR1	
ENST00000486841	ENSG00000127080	IPPK	
ENST00000495031	ENSG00000175984	DENND2C	
ENST00000504491	ENSG00000164066	INTU	
ENST00000594744	ENSG00000267978	MAGEA9	
ENST00000396868	ENSG00000010539	ZNF200	
ENST00000575630	ENSG00000010539	ZNF200	
ENST00000548961	ENSG00000161800	RACGAP1	
ENST00000548247	ENSG00000161800	RACGAP1	filled
ENST00000369550	ENSG00000130826	DKC1	
ENST00000475423	ENSG00000130826	DKC1	
ENST00000413910	ENSG00000130826	DKC1	
ENST00000473552	ENSG00000130826	DKC1	
ENST00000620277	ENSG00000130826	DKC1	
ENST00000483567	ENSG00000164675	IQUB	
ENST00000409354	ENSG00000143951	WDPCP	filled
ENST00000272321	ENSG00000143951	WDPCP	filled
ENST00000409120	ENSG00000143951	WDPCP	filled
ENST00000398544	ENSG00000143951	WDPCP	filled
ENST00000409562	ENSG00000143951	WDPCP	filled
ENST00000490935	ENSG00000143951	WDPCP	filled
ENST00000467687	ENSG00000143951	WDPCP	filled
ENST00000382275	ENSG00000145526	CDH18	filled
ENST00000507958	ENSG00000145526	CDH18	filled
ENST00000506372	ENSG00000145526	CDH18	filled
ENST00000502796	ENSG00000145526	CDH18	filled
ENST00000515257	ENSG00000145526	CDH18	filled
ENST00000511273	ENSG00000145526	CDH18	filled
ENST00000511423	ENSG00000145526	CDH18	filled
ENST00000507632	ENSG00000145526	CDH18	filled
ENST00000503132	ENSG00000145526	CDH18	filled
ENST00000274170	ENSG00000145526	CDH18	filled
ENST00000551533	ENSG00000223793	HLA-DQA2	filled
ENST00000264852	ENSG00000072858	SIDT1	filled
ENST00000491730	ENSG00000072858	SIDT1	filled
ENST00000483946	ENSG00000072858	SIDT1	filled
ENST00000393830	ENSG00000072858	SIDT1	filled
ENST00000468642	ENSG00000138459	SLC35A5	
ENST00000549318	ENSG00000144785	RP11-977G19.10	filled
ENST00000547423	ENSG00000144785	RP11-977G19.10	filled
ENST00000548360	ENSG00000144785	RP11-977G19.10	filled
ENST00000261590	ENSG00000046604	DSG2	filled
ENST00000603700	ENSG00000270617	URGCP-MRPS24	

ENST00000356321	ENSG00000198146	ZNF770	
ENST00000529028	ENSG00000133808	MICALCL	
ENST00000533534	ENSG00000133808	MICALCL	
ENST00000609672	ENSG00000147065	MSN	filled
ENST00000447323	ENSG00000147065	MSN	
ENST00000498744	ENSG00000112146	FBXO9	filled
ENST00000370939	ENSG00000112146	FBXO9	filled
ENST00000461222	ENSG00000112146	FBXO9	filled
ENST00000323557	ENSG00000112146	FBXO9	filled
ENST00000473337	ENSG00000112146	FBXO9	
ENST00000244426	ENSG00000112146	FBXO9	filled
ENST00000468481	ENSG00000112146	FBXO9	
ENST00000480463	ENSG00000112146	FBXO9	
ENST00000484436	ENSG00000112146	FBXO9	
ENST00000473318	ENSG00000112146	FBXO9	
ENST00000461349	ENSG00000136819	C9orf78	filled
ENST00000461762	ENSG00000136819	C9orf78	filled
ENST00000463713	ENSG00000162777	DENND2D	
ENST00000615982	ENSG00000234616	JRK	
ENST00000571961	ENSG00000234616	JRK	
ENST00000414731	ENSG00000234704	BRD2	
ENST00000492825	ENSG00000234704	BRD2	
ENST00000476208	ENSG00000151687	ANKAR	filled
ENST00000507492	ENSG00000188981	MSANTD1	filled
ENST00000438480	ENSG00000188981	MSANTD1	filled
ENST00000619839	ENSG00000149294	NCAM1	filled
ENST00000401611	ENSG00000149294	NCAM1	filled
ENST00000621128	ENSG00000149294	NCAM1	filled
ENST00000613217	ENSG00000149294	NCAM1	filled
ENST00000316851	ENSG00000149294	NCAM1	filled
ENST00000617166	ENSG00000149294	NCAM1	filled
ENST00000620046	ENSG00000149294	NCAM1	filled
ENST00000531044	ENSG00000149294	NCAM1	filled
ENST00000529356	ENSG00000149294	NCAM1	filled
ENST00000615525	ENSG00000149294	NCAM1	filled
ENST00000621850	ENSG00000149294	NCAM1	filled
ENST00000611284	ENSG00000149294	NCAM1	
ENST00000528158	ENSG00000149294	NCAM1	filled
ENST00000618266	ENSG00000149294	NCAM1	filled
ENST00000615112	ENSG00000149294	NCAM1	filled
ENST00000615285	ENSG00000149294	NCAM1	filled
ENST00000621518	ENSG00000149294	NCAM1	filled
ENST00000419137	ENSG00000273303	RP11-944L7.5	filled
ENST00000411912	ENSG00000115464	USP34	filled
ENST00000398571	ENSG00000115464	USP34	filled
ENST00000463046	ENSG00000115464	USP34	filled
ENST00000476716	ENSG00000115464	USP34	
ENST00000467128	ENSG00000115464	USP34	
ENST00000472689	ENSG00000115464	USP34	
ENST00000472706	ENSG00000115464	USP34	filled
ENST00000490527	ENSG00000115464	USP34	
ENST00000483672	ENSG00000115464	USP34	
ENST00000482250	ENSG00000115464	USP34	
ENST00000487547	ENSG00000115464	USP34	
ENST00000468703	ENSG00000115464	USP34	
ENST00000453734	ENSG00000115464	USP34	filled
ENST00000492882	ENSG00000115464	USP34	
ENST00000484179	ENSG00000115464	USP34	filled
ENST00000494867	ENSG00000115464	USP34	
ENST00000299980	ENSG00000166747	AP1G1	filled

ENST00000569748	ENSG00000166747	AP1G1	filled
ENST00000393512	ENSG00000166747	AP1G1	filled
ENST00000565009	ENSG00000166747	AP1G1	filled
ENST00000568327	ENSG00000166747	AP1G1	filled
ENST00000561863	ENSG00000174485	DENND4A	
ENST00000412641	ENSG00000186448	ZNF197	
ENST00000383745	ENSG00000186448	ZNF197	
ENST00000502955	ENSG00000204580	DDR1	
ENST00000505066	ENSG00000204580	DDR1	
ENST00000505534	ENSG00000204580	DDR1	
ENST00000513749	ENSG00000204580	DDR1	
ENST00000503180	ENSG00000204580	DDR1	
ENST00000508317	ENSG00000204580	DDR1	
ENST00000482050	ENSG00000204580	DDR1	
ENST00000507053	ENSG00000145833	DDX46	
ENST00000559306	ENSG00000035664	DAPK2	filled
ENST00000488800	ENSG00000155465	SLC7A7	
ENST00000554741	ENSG00000155465	SLC7A7	
ENST00000553874	ENSG00000155465	SLC7A7	
ENST00000553351	ENSG00000155465	SLC7A7	
ENST00000313732	ENSG00000153786	ZDHHC7	filled
ENST00000344861	ENSG00000153786	ZDHHC7	filled
ENST00000564526	ENSG00000153786	ZDHHC7	
ENST00000564466	ENSG00000153786	ZDHHC7	filled
ENST00000335123	ENSG00000186844	LCE1A	
ENST00000562971	ENSG00000185947	ZNF267	filled
ENST00000441037	ENSG00000166748	AGBL1	filled
ENST00000484834	ENSG00000133063	CHIT1	filled
ENST00000479483	ENSG00000133063	CHIT1	
ENST00000460619	ENSG00000133063	CHIT1	
ENST00000493638	ENSG00000135250	SRPK2	
ENST00000462282	ENSG00000135250	SRPK2	filled
ENST00000569198	ENSG00000167194	C16orf92	
ENST00000567847	ENSG00000167194	C16orf92	
ENST00000591050	ENSG00000105607	GCDH	filled
ENST00000588242	ENSG00000105607	GCDH	
ENST00000555000	ENSG00000054983	GALC	filled
ENST00000616143	ENSG00000278545	8-Mar	
ENST00000587572	ENSG00000130158	DOCK6	
ENST00000308377	ENSG00000172716	SLFN11	
ENST00000394566	ENSG00000172716	SLFN11	
ENST00000588579	ENSG00000172716	SLFN11	
ENST00000239374	ENSG00000120262	CCDC170	filled
ENST00000353214	ENSG00000136950	ARPC5L	filled
ENST00000374791	ENSG00000182667	NTM	filled
ENST00000436745	ENSG00000182667	NTM	filled
ENST00000477098	ENSG00000182667	NTM	filled
ENST00000416661	ENSG00000182667	NTM	filled
ENST00000498764	ENSG00000182667	NTM	filled
ENST00000470371	ENSG00000182667	NTM	
ENST00000374786	ENSG00000182667	NTM	filled
ENST00000425719	ENSG00000182667	NTM	filled
ENST00000615434	ENSG00000274488	LY6H	
ENST00000610554	ENSG00000274488	LY6H	
ENST00000422437	ENSG00000258388	PPT2-EGFL8	
ENST00000259963	ENSG00000137414	FAM8A1	filled
ENST00000482403	ENSG00000240389	EGFL8	filled
ENST00000580248	ENSG00000143183	TMCO1	filled
ENST00000496156	ENSG00000132128	LRRC41	
ENST00000472710	ENSG00000132128	LRRC41	

ENST00000490798	ENSG00000101104	PABPC1L	filled
ENST00000372824	ENSG00000101104	PABPC1L	filled
ENST00000372819	ENSG00000101104	PABPC1L	filled
ENST00000372826	ENSG00000101104	PABPC1L	filled
ENST00000495261	ENSG00000198836	OPA1	
ENST00000544450	ENSG00000196405	EVL	filled
ENST00000554460	ENSG00000196405	EVL	filled
ENST00000370859	ENSG00000137968	SLC44A5	filled
ENST00000370855	ENSG00000137968	SLC44A5	filled
ENST00000469525	ENSG00000137968	SLC44A5	filled
ENST00000566844	ENSG00000137817	PARP6	
ENST00000299977	ENSG00000166750	SLFN5	
ENST00000335624	ENSG00000186806	VSIG10L	
ENST00000511349	ENSG00000152942	RAD17	
ENST00000532833	ENSG00000137699	TRIM29	
ENST00000536130	ENSG00000130921	C12orf65	filled
ENST00000546132	ENSG00000130921	C12orf65	filled
ENST00000474846	ENSG00000152642	GPD1L	
ENST00000383546	ENSG00000206467	OR2H2	
ENST00000624330	ENSG00000279950	RPS4Y1	
ENST00000258711	ENSG00000136213	CHST12	
ENST00000368744	ENSG00000203783	PRR9	
ENST00000450456	ENSG00000203733	GJE1	
ENST00000577913	ENSG00000136492	BRIP1	
ENST00000355854	ENSG00000102547	CAB39L	
ENST00000409308	ENSG00000102547	CAB39L	filled
ENST00000425242	ENSG00000102547	CAB39L	
ENST00000410043	ENSG00000102547	CAB39L	filled
ENST00000457041	ENSG00000102547	CAB39L	
ENST00000413278	ENSG00000102547	CAB39L	filled
ENST00000409082	ENSG00000102547	CAB39L	filled
ENST00000476943	ENSG00000102547	CAB39L	filled
ENST00000347776	ENSG00000102547	CAB39L	filled
ENST00000610540	ENSG00000102547	CAB39L	filled
ENST00000615772	ENSG00000224774	CSNK2B	
ENST00000615589	ENSG00000224774	CSNK2B	
ENST00000622139	ENSG00000224774	CSNK2B	
ENST00000577288	ENSG00000126351	THRA	
ENST00000585047	ENSG00000126351	THRA	
ENST00000578218	ENSG00000126351	THRA	
ENST00000577486	ENSG00000126351	THRA	
ENST00000527010	ENSG00000173898	SPTBN2	
ENST00000537495	ENSG00000255863	AC073610.5	
ENST00000603611	ENSG00000100890	KIAA0391	
ENST00000604073	ENSG00000100890	KIAA0391	
ENST00000614783	ENSG00000206406	CSNK2B	
ENST00000621953	ENSG00000206406	CSNK2B	
ENST00000620798	ENSG00000206406	CSNK2B	
ENST00000606514	ENSG00000189007	ADAT2	
ENST00000237283	ENSG00000189007	ADAT2	
ENST00000571845	ENSG00000262484	CCER2	
ENST00000571838	ENSG00000262484	CCER2	
ENST00000622573	ENSG00000277399	GPR179	
ENST00000553027	ENSG00000134285	FKBP11	
ENST00000479872	ENSG00000143179	UCK2	
ENST00000373827	ENSG00000151150	ANK3	filled
ENST00000510382	ENSG00000151150	ANK3	filled
ENST00000492002	ENSG00000172889	EGFL7	
ENST00000477264	ENSG00000171603	CLSTN1	
ENST00000555360	ENSG00000179627	ZBTB42	

ENST00000342537	ENSG00000179627	ZBTB42	
ENST00000356069	ENSG00000176937	OR52R1	
ENST00000590289	ENSG00000181513	ACBD4	
ENST00000309838	ENSG00000175393	OR10A6	
ENST00000479261	ENSG00000100150	DEPDC5	filled
ENST00000622355	ENSG00000273637	RP5-864K19.7	filled
ENST00000616568	ENSG00000119403	PHF19	filled
ENST00000310454	ENSG00000115159	GPD2	filled
ENST00000409861	ENSG00000115159	GPD2	filled
ENST00000519948	ENSG00000253719	ATXN7L3B	
ENST00000304833	ENSG00000171484	OR1B1	
ENST00000613314	ENSG00000253159	PCDHGA12	
ENST00000252085	ENSG00000253159	PCDHGA12	filled
ENST00000369026	ENSG00000143384	MCL1	
ENST00000620947	ENSG00000143384	MCL1	
ENST00000305479	ENSG00000168634	WFDC13	
ENST00000296452	ENSG00000164061	BSN	filled
ENST00000467456	ENSG00000164061	BSN	
ENST00000442259	ENSG00000229685	HLA-DPA1	
ENST00000306279	ENSG00000168874	ATOH8	
ENST00000463422	ENSG00000168874	ATOH8	
ENST00000473116	ENSG00000168874	ATOH8	
ENST00000493659	ENSG00000243719	HLA-DMA	
ENST00000466446	ENSG00000243719	HLA-DMA	
ENST00000492275	ENSG00000182670	TTC3	
ENST00000463216	ENSG00000182670	TTC3	
ENST00000494243	ENSG00000182670	TTC3	
ENST00000399010	ENSG00000182670	TTC3	
ENST00000484047	ENSG00000182670	TTC3	
ENST00000476784	ENSG00000182670	TTC3	filled
ENST00000613176	ENSG00000278839	SERF1B	
ENST00000430575	ENSG00000129824	RPS4Y1	
ENST00000406117	ENSG00000100344	PNPLA3	
ENST00000412982	ENSG00000072195	SPEG	
ENST00000475104	ENSG00000072195	SPEG	
ENST00000617028	ENSG00000072195	SPEG	
ENST00000530028	ENSG00000255302	EID1	
ENST00000558295	ENSG00000255302	EID1	
ENST00000485988	ENSG00000087053	MTMR2	
ENST00000292174	ENSG00000160683	CXCR5	filled
ENST00000237281	ENSG00000118496	FBXO30	filled
ENST00000555074	ENSG00000259132	RP11-298I3.5	filled
ENST00000557267	ENSG00000126777	KTN1	filled
ENST00000554567	ENSG00000126777	KTN1	filled
ENST00000555498	ENSG00000126777	KTN1	filled
ENST00000438792	ENSG00000126777	KTN1	filled
ENST00000395314	ENSG00000126777	KTN1	filled
ENST00000459737	ENSG00000126777	KTN1	filled
ENST00000395308	ENSG00000126777	KTN1	filled
ENST00000395311	ENSG00000126777	KTN1	filled
ENST00000554294	ENSG00000126777	KTN1	
ENST00000413890	ENSG00000126777	KTN1	filled
ENST00000395309	ENSG00000126777	KTN1	filled
ENST00000550124	ENSG00000257390	RP11-762I7.5	filled
ENST00000546813	ENSG00000257390	RP11-762I7.5	filled
ENST00000548593	ENSG00000257390	RP11-762I7.5	filled
ENST00000552719	ENSG00000257390	RP11-762I7.5	filled
ENST00000439011	ENSG00000177463	NR2C2	
ENST00000413194	ENSG00000177463	NR2C2	
ENST00000543436	ENSG00000175899	A2M	filled

ENST00000582963	ENSG00000141741	MIEN1	
ENST00000413212	ENSG00000076242	MLH1	
ENST00000502401	ENSG00000138758	11-Sep	
ENST00000513373	ENSG00000138758	11-Sep	
ENST00000513697	ENSG00000138758	11-Sep	
ENST00000515671	ENSG00000138758	11-Sep	
ENST00000527808	ENSG00000110330	BIRC2	
ENST00000532832	ENSG00000110330	BIRC2	
ENST00000534646	ENSG00000110330	BIRC2	
ENST00000527465	ENSG00000110330	BIRC2	
ENST00000399518	ENSG00000188089	PLA2G4E	filled
ENST00000472839	ENSG00000257127	CLLU1	filled
ENST00000512817	ENSG00000257127	CLLU1	filled
ENST00000366661	ENSG00000143643	TTC13	
ENST00000366662	ENSG00000143643	TTC13	
ENST00000481976	ENSG00000143643	TTC13	
ENST00000466507	ENSG00000143643	TTC13	
ENST00000534788	ENSG00000110375	UPK2	filled
ENST00000264031	ENSG00000110375	UPK2	filled
ENST00000374706	ENSG00000108100	CCNY	filled
ENST00000312428	ENSG00000118997	DNAH7	filled
ENST00000498097	ENSG00000135951	TSGA10	
ENST00000488600	ENSG00000123684	LPGAT1	
ENST00000460133	ENSG00000158220	ESYT3	filled
ENST00000492130	ENSG00000081087	OSTM1	
ENST00000440575	ENSG00000081087	OSTM1	filled
ENST00000492070	ENSG00000081087	OSTM1	
ENST00000394877	ENSG00000145331	TRMT10A	
ENST00000273962	ENSG00000145331	TRMT10A	
ENST00000394876	ENSG00000145331	TRMT10A	
ENST00000455368	ENSG00000145331	TRMT10A	
ENST00000514547	ENSG00000145331	TRMT10A	
ENST00000521247	ENSG00000066855	MTFR1	
ENST00000527155	ENSG00000066855	MTFR1	
ENST00000518352	ENSG00000066855	MTFR1	
ENST00000523158	ENSG00000066855	MTFR1	
ENST00000520398	ENSG00000066855	MTFR1	
ENST00000527553	ENSG00000066855	MTFR1	
ENST00000462460	ENSG00000116584	ARHGEF2	filled
ENST00000477754	ENSG00000116584	ARHGEF2	filled
ENST00000471589	ENSG00000116584	ARHGEF2	filled
ENST00000497907	ENSG00000116584	ARHGEF2	filled
ENST00000476273	ENSG00000116584	ARHGEF2	filled
ENST00000465079	ENSG00000116584	ARHGEF2	filled
ENST00000495070	ENSG00000116584	ARHGEF2	filled
ENST00000423422	ENSG00000116584	ARHGEF2	filled
ENST00000619863	ENSG00000087085	ACHE	
ENST00000354171	ENSG00000167468	GPX4	
ENST00000589115	ENSG00000167468	GPX4	
ENST00000593032	ENSG00000167468	GPX4	
ENST00000585362	ENSG00000167468	GPX4	
ENST00000622390	ENSG00000167468	GPX4	
ENST00000616066	ENSG00000167468	GPX4	
ENST00000400169	ENSG00000154639	CXADR	
ENST00000553948	ENSG00000165914	TTC7B	
ENST00000436724	ENSG00000142949	PTPRF	filled
ENST00000372405	ENSG00000142949	PTPRF	filled
ENST00000433211	ENSG00000183230	CTNNA3	filled
ENST00000494580	ENSG00000183230	CTNNA3	filled
ENST00000545309	ENSG00000183230	CTNNA3	filled

ENST00000602531	ENSG00000124074	ENKD1	
ENST00000329203	ENSG00000182103	FAM181B	filled
ENST00000547498	ENSG00000229074	HLA-DRB1	filled
ENST00000440503	ENSG00000122733	KIAA1045	
ENST00000576222	ENSG00000262209	PCDHGB3	filled
ENST00000618934	ENSG00000262209	PCDHGB3	
ENST00000395699	ENSG00000146676	PURB	
ENST00000389811	ENSG00000153093	ACOXL	filled
ENST00000439055	ENSG00000153093	ACOXL	filled
ENST00000461340	ENSG00000153093	ACOXL	
ENST00000417074	ENSG00000153093	ACOXL	filled
ENST00000433706	ENSG00000153093	ACOXL	filled
ENST00000443586	ENSG00000153093	ACOXL	filled
ENST00000369770	ENSG00000177301	KCNA2	filled
ENST00000485317	ENSG00000177301	KCNA2	
ENST00000316361	ENSG00000177301	KCNA2	
ENST00000525120	ENSG00000177301	KCNA2	
ENST00000505654	ENSG00000145730	PAM	filled
ENST00000510208	ENSG00000145730	PAM	filled
ENST00000506127	ENSG00000145730	PAM	
ENST00000502472	ENSG00000145730	PAM	filled
ENST00000509523	ENSG00000145730	PAM	
ENST00000506006	ENSG00000145730	PAM	
ENST00000313116	ENSG00000147124	ZNF41	filled
ENST00000377065	ENSG00000147124	ZNF41	filled
ENST00000432977	ENSG00000147124	ZNF41	filled
ENST00000465311	ENSG00000147124	ZNF41	filled
ENST00000596304	ENSG00000221983	UBA52	filled
ENST00000430157	ENSG00000221983	UBA52	filled
ENST00000596273	ENSG00000221983	UBA52	filled
ENST00000595683	ENSG00000221983	UBA52	filled
ENST00000595158	ENSG00000221983	UBA52	
ENST00000598780	ENSG00000221983	UBA52	
ENST00000544880	ENSG00000256591	RP11-286N22.8	filled
ENST00000543044	ENSG00000256591	RP11-286N22.8	filled
ENST00000373012	ENSG00000185668	POU3F1	
ENST00000539751	ENSG00000185958	FAM186A	
ENST00000581849	ENSG00000108349	CASC3	filled
ENST00000583649	ENSG00000108349	CASC3	filled
ENST00000584997	ENSG00000108349	CASC3	filled
ENST00000538399	ENSG00000024422	EHD2	filled
ENST00000262366	ENSG00000126603	GLIS2	filled
ENST00000369408	ENSG00000135299	ANKRD6	filled
ENST00000339746	ENSG00000135299	ANKRD6	filled
ENST00000447838	ENSG00000135299	ANKRD6	filled
ENST00000520458	ENSG00000135299	ANKRD6	
ENST00000482475	ENSG00000198000	NOL8	
ENST00000560234	ENSG00000166803	KIAA0101	
ENST00000514445	ENSG00000109743	BST1	
ENST00000514989	ENSG00000109743	BST1	
ENST00000454628	ENSG00000227044	OR2H2	
ENST00000399139	ENSG00000159261	CLDN14	filled
ENST00000374881	ENSG00000204264	PSMB8	filled
ENST00000622102	ENSG00000106086	PLEKHA8	filled
ENST00000449193	ENSG00000180592	SKIDA1	
ENST00000487107	ENSG00000180592	SKIDA1	
ENST00000450655	ENSG00000113525	IL5	
ENST00000447054	ENSG00000058866	DGKG	filled
ENST00000588606	ENSG00000267795	SMIM22	
ENST00000586005	ENSG00000267795	SMIM22	

ENST00000591870	ENSG00000267795	SMIM22	
ENST00000588500	ENSG00000267795	SMIM22	
ENST00000383269	ENSG00000206314	GPSM3	
ENST00000428002	ENSG00000184277	TM2D3	filled
ENST00000559107	ENSG00000184277	TM2D3	filled
ENST00000558129	ENSG00000184277	TM2D3	filled
ENST00000454222	ENSG00000127989	MTERF1	filled
ENST00000419292	ENSG00000127989	MTERF1	filled
ENST00000610426	ENSG00000164346	NSA2	
ENST00000360388	ENSG00000196376	SLC35F1	filled
ENST00000359591	ENSG00000197324	LRP10	
ENST00000551466	ENSG00000197324	LRP10	
ENST00000585619	ENSG00000123143	PKN1	filled
ENST00000586557	ENSG00000123143	PKN1	filled
ENST00000587429	ENSG00000123143	PKN1	filled
ENST00000590097	ENSG00000123143	PKN1	filled
ENST00000216629	ENSG00000100739	BDKRB1	
ENST00000572154	ENSG00000184697	CLDN6	
ENST00000455089	ENSG00000146648	EGFR	filled
ENST00000344576	ENSG00000146648	EGFR	filled
ENST00000604458	ENSG00000270757	HSPE1-MOB4	filled
ENST00000394597	ENSG00000092871	RFFL	filled
ENST00000447669	ENSG00000092871	RFFL	filled
ENST00000415395	ENSG00000092871	RFFL	
ENST00000413582	ENSG00000092871	RFFL	
ENST00000414419	ENSG00000092871	RFFL	filled
ENST00000454872	ENSG00000177694	NAALADL2	filled
ENST00000485853	ENSG00000177694	NAALADL2	filled
ENST00000473253	ENSG00000177694	NAALADL2	filled
ENST00000369769	ENSG00000177272	KCNA3	
ENST00000615506	ENSG00000100380	ST13	
ENST00000620312	ENSG00000100380	ST13	
ENST00000313349	ENSG00000179833	SERTAD2	
ENST00000476805	ENSG00000179833	SERTAD2	filled
ENST00000608423	ENSG00000179833	SERTAD2	
ENST00000513454	ENSG00000113552	GNPDA1	filled
ENST00000396344	ENSG00000253293	HOXA10	filled
ENST00000558468	ENSG00000259529	IRF9	
ENST00000396862	ENSG00000140993	TIGD7	
ENST00000573608	ENSG00000140993	TIGD7	
ENST00000572297	ENSG00000140993	TIGD7	
ENST00000571748	ENSG00000140993	TIGD7	
ENST00000589742	ENSG00000087903	RFX2	filled
ENST00000586806	ENSG00000087903	RFX2	filled
ENST00000587181	ENSG00000087903	RFX2	filled
ENST00000591793	ENSG00000267022	ZNF223	filled
ENST00000341394	ENSG00000135387	CAPRIN1	
ENST00000389645	ENSG00000135387	CAPRIN1	
ENST00000534042	ENSG00000135387	CAPRIN1	
ENST00000529307	ENSG00000135387	CAPRIN1	
ENST00000526494	ENSG00000135387	CAPRIN1	
ENST00000532755	ENSG00000135387	CAPRIN1	
ENST00000530008	ENSG00000135387	CAPRIN1	
ENST00000465229	ENSG00000107371	EXOSC3	
ENST00000482614	ENSG00000107371	EXOSC3	
ENST00000528330	ENSG00000081853	PCDHGA2	
ENST00000394576	ENSG00000081853	PCDHGA2	filled
ENST00000502598	ENSG00000198055	GRK6	filled
ENST00000506296	ENSG00000198055	GRK6	filled
ENST00000504064	ENSG00000170006	TMEM154	filled

ENST00000517951	ENSG00000135074	ADAM19	filled
ENST00000519718	ENSG00000272772	CTD-2410N18.5	filled
ENST00000559663	ENSG00000167258	CDK12	filled
ENST00000581963	ENSG00000167258	CDK12	
ENST00000559545	ENSG00000167258	CDK12	
ENST00000558240	ENSG00000167258	CDK12	filled
ENST00000490643	ENSG00000163870	TPRA1	
ENST00000544848	ENSG00000246705	H2AFJ	
ENST00000504218	ENSG00000174796	THAP6	
ENST00000579419	ENSG00000109083	IFT20	
ENST00000578009	ENSG00000109083	IFT20	
ENST00000395418	ENSG00000109083	IFT20	
ENST00000585313	ENSG00000109083	IFT20	
ENST00000558623	ENSG00000140323	DISP2	
ENST00000494431	ENSG00000149043	SYT8	
ENST00000487915	ENSG00000129521	EGLN3	filled
ENST00000550114	ENSG00000129521	EGLN3	
ENST00000432179	ENSG00000153094	BCL2L11	
ENST00000526660	ENSG00000137491	SLCO2B1	filled
ENST00000531713	ENSG00000137491	SLCO2B1	filled
ENST00000431917	ENSG00000085978	ATG16L1	filled
ENST00000620060	ENSG00000277114	CNOT3	filled
ENST00000495760	ENSG00000187980	PLA2G2C	
ENST00000373129	ENSG00000196182	STK40	filled
ENST00000373130	ENSG00000196182	STK40	filled
ENST00000373132	ENSG00000196182	STK40	filled
ENST00000460417	ENSG00000196182	STK40	
ENST00000482458	ENSG00000196182	STK40	filled
ENST00000460017	ENSG00000196182	STK40	filled
ENST00000285814	ENSG00000155438	NIFK	filled
ENST00000380736	ENSG00000047648	ARHGAP6	filled
ENST00000337414	ENSG00000047648	ARHGAP6	filled
ENST00000495242	ENSG00000047648	ARHGAP6	filled
ENST00000489330	ENSG00000047648	ARHGAP6	filled
ENST00000380718	ENSG00000047648	ARHGAP6	filled
ENST00000485635	ENSG00000115221	ITGB6	filled
ENST00000498478	ENSG00000115221	ITGB6	
ENST00000459712	ENSG00000115221	ITGB6	
ENST00000477514	ENSG00000115221	ITGB6	
ENST00000324344	ENSG00000188215	DCUN1D3	filled
ENST00000563934	ENSG00000188215	DCUN1D3	
ENST00000441705	ENSG00000234243	GPSM3	
ENST00000354670	ENSG00000104081	BMF	
ENST00000397573	ENSG00000104081	BMF	
ENST00000557870	ENSG00000104081	BMF	
ENST00000560430	ENSG00000104081	BMF	
ENST00000558057	ENSG00000104081	BMF	
ENST00000547702	ENSG00000018236	CNTN1	filled
ENST00000551424	ENSG00000018236	CNTN1	filled
ENST00000551295	ENSG00000018236	CNTN1	filled
ENST00000552248	ENSG00000018236	CNTN1	filled
ENST00000547849	ENSG00000018236	CNTN1	filled
ENST00000552913	ENSG00000018236	CNTN1	filled
ENST00000348761	ENSG00000018236	CNTN1	filled
ENST00000548481	ENSG00000018236	CNTN1	
ENST00000347616	ENSG00000018236	CNTN1	filled
ENST00000560674	ENSG00000182253	SYNM	filled
ENST00000613284	ENSG00000268350	FAM156A	
ENST00000496248	ENSG00000143995	MEIS1	
ENST00000397906	ENSG00000100154	TTC28	filled

ENST00000468807	ENSG00000100154	TTC28	filled
ENST00000454967	ENSG00000203943	SAMD13	
ENST00000574297	ENSG00000161929	SCIMP	filled
ENST00000486744	ENSG00000133619	KRBA1	filled
ENST00000496259	ENSG00000133619	KRBA1	filled
ENST00000319551	ENSG00000133619	KRBA1	filled
ENST00000619947	ENSG00000119608	PROX2	filled
ENST00000282091	ENSG00000152266	PTH	
ENST00000528994	ENSG00000197863	ZNF790	
ENST00000586323	ENSG00000197863	ZNF790	
ENST00000527645	ENSG00000197863	ZNF790	
ENST00000613249	ENSG00000197863	ZNF790	
ENST00000614179	ENSG00000197863	ZNF790	
ENST00000615484	ENSG00000197863	ZNF790	
ENST00000335327	ENSG00000132970	WASF3	filled
ENST00000361042	ENSG00000132970	WASF3	filled
ENST00000477876	ENSG00000139496	NUPL1	
ENST00000619468	ENSG00000224398	CSNK2B	
ENST00000621623	ENSG00000224398	CSNK2B	
ENST00000621203	ENSG00000224398	CSNK2B	
ENST00000475040	ENSG00000144029	MRPS5	filled
ENST00000482796	ENSG00000251184	RP11-101E3.5	filled
ENST00000553525	ENSG00000182732	RGS6	filled
ENST00000555571	ENSG00000182732	RGS6	filled
ENST00000553530	ENSG00000182732	RGS6	filled
ENST00000554474	ENSG00000182732	RGS6	filled
ENST00000556437	ENSG00000182732	RGS6	filled
ENST00000520813	ENSG00000147677	EIF3H	
ENST00000430340	ENSG00000145012	LPP	filled
ENST00000454789	ENSG00000145012	LPP	filled
ENST00000604900	ENSG00000183283	DAZAP2	filled
ENST00000436900	ENSG00000183283	DAZAP2	
ENST00000464461	ENSG00000213203	GIMAP1	
ENST00000598654	ENSG00000269858	EGLN2	
ENST00000601733	ENSG00000269858	EGLN2	
ENST00000422939	ENSG00000104447	TRPS1	filled
ENST00000482608	ENSG00000169085	C8orf46	filled
ENST00000519702	ENSG00000169085	C8orf46	filled
ENST00000510552	ENSG00000174125	TLR1	
ENST00000505744	ENSG00000174125	TLR1	
ENST00000506146	ENSG00000174125	TLR1	filled
ENST00000494813	ENSG00000089820	ARHGAP4	
ENST00000442262	ENSG00000089820	ARHGAP4	
ENST00000445177	ENSG00000160584	SIK3	
ENST00000375300	ENSG00000160584	SIK3	
ENST00000415541	ENSG00000160584	SIK3	
ENST00000446921	ENSG00000160584	SIK3	
ENST00000413553	ENSG00000160584	SIK3	
ENST00000497049	ENSG00000160584	SIK3	
ENST00000543758	ENSG00000198040	ZNF84	filled
ENST00000539686	ENSG00000198040	ZNF84	filled
ENST00000542358	ENSG00000198040	ZNF84	
ENST00000392319	ENSG00000198040	ZNF84	filled
ENST00000585895	ENSG00000116017	ARID3A	
ENST00000592216	ENSG00000116017	ARID3A	
ENST00000556845	ENSG00000139865	TTC6	filled
ENST00000317683	ENSG00000177051	FBXO46	filled
ENST00000359534	ENSG00000164626	KCNK5	
ENST00000570896	ENSG00000174292	TNK1	filled
ENST00000576812	ENSG00000174292	TNK1	filled

ENST00000575268	ENSG00000174292	TNK1	filled
ENST00000605286	ENSG00000196422	PPP1R26	
ENST00000604351	ENSG00000196422	PPP1R26	
ENST00000356818	ENSG00000196422	PPP1R26	
ENST00000401470	ENSG00000196422	PPP1R26	
ENST00000605660	ENSG00000196422	PPP1R26	
ENST00000523262	ENSG00000147408	CSGALNACT1	filled
ENST00000517494	ENSG00000147408	CSGALNACT1	filled
ENST00000563798	ENSG00000075399	VPS9D1	
ENST00000448728	ENSG00000130294	KIF1A	
ENST00000484331	ENSG00000091428	RAPGEF4	filled
ENST00000450780	ENSG00000248235	AC037459.4	filled
ENST00000430850	ENSG00000248235	AC037459.4	
ENST00000447849	ENSG00000248235	AC037459.4	
ENST00000455239	ENSG00000152082	MZT2B	
ENST00000422674	ENSG00000227739	TUBB	
ENST00000435546	ENSG00000227739	TUBB	
ENST00000381196	ENSG00000153707	PTPRD	filled
ENST00000463477	ENSG00000153707	PTPRD	filled
ENST00000334567	ENSG00000186329	TMEM212	filled
ENST00000405008	ENSG00000068781	STON1-GTF2A1L	filled
ENST00000402114	ENSG00000068781	STON1-GTF2A1L	filled
ENST00000446929	ENSG00000100146	SOX10	
ENST00000360880	ENSG00000100146	SOX10	
ENST00000420196	ENSG00000136237	RAPGEF5	filled
ENST00000344041	ENSG00000136237	RAPGEF5	filled
ENST00000615541	ENSG00000080603	SRCAP	
ENST00000395330	ENSG00000240065	PSMB9	
ENST00000329905	ENSG00000185933	CALHM1	
ENST00000554446	ENSG00000092036	HAUS4	
ENST00000557591	ENSG00000092036	HAUS4	
ENST00000382080	ENSG00000185053	SGCZ	filled
ENST00000421524	ENSG00000185053	SGCZ	filled
ENST00000558573	ENSG00000229474	PATL2	filled
ENST00000225777	ENSG00000108639	SYNGR2	
ENST00000585591	ENSG00000108639	SYNGR2	
ENST00000589711	ENSG00000108639	SYNGR2	
ENST00000589168	ENSG00000108639	SYNGR2	
ENST00000590201	ENSG00000108639	SYNGR2	
ENST00000455900	ENSG00000176083	ZNF683	
ENST00000451801	ENSG00000176083	ZNF683	
ENST00000454975	ENSG00000176083	ZNF683	
ENST00000416125	ENSG00000176083	ZNF683	
ENST00000584895	ENSG00000215472	RPL17-C18orf32	
ENST00000332968	ENSG00000215472	RPL17-C18orf32	
ENST00000577910	ENSG00000215472	RPL17-C18orf32	
ENST00000376621	ENSG00000204590	GNL1	
ENST00000380007	ENSG00000178015	GPR150	
ENST00000428189	ENSG00000229470	GNL1	
ENST00000337120	ENSG00000178188	SH2B1	filled
ENST00000524318	ENSG00000168484	SFTPC	
ENST00000357996	ENSG00000171055	FEZ2	filled
ENST00000497993	ENSG00000176142	TMEM39A	
ENST00000481566	ENSG00000065802	ASB1	
ENST00000371497	ENSG00000182463	TSHZ2	filled
ENST00000603338	ENSG00000182463	TSHZ2	filled
ENST00000605656	ENSG00000182463	TSHZ2	filled
ENST00000580380	ENSG00000189159	HN1	
ENST00000584079	ENSG00000189159	HN1	
ENST00000441371	ENSG00000120341	SEC16B	

ENST00000367568	ENSG00000135604	STX11	
ENST00000368172	ENSG00000183853	KIRREL	
ENST00000433765	ENSG00000180720	CHRM4	
ENST00000373043	ENSG00000197982	C1orf122	
ENST00000396113	ENSG00000156170	NDUFAF6	filled
ENST00000519136	ENSG00000156170	NDUFAF6	filled
ENST00000396111	ENSG00000156170	NDUFAF6	filled
ENST00000521840	ENSG00000156170	NDUFAF6	filled
ENST00000523184	ENSG00000156170	NDUFAF6	filled
ENST00000521063	ENSG00000156170	NDUFAF6	
ENST00000264663	ENSG00000112992	NNT	filled
ENST00000573521	ENSG00000261934	PCDHGA9	filled
ENST00000311637	ENSG00000054793	ATP9A	filled
ENST00000338821	ENSG00000054793	ATP9A	filled
ENST00000477492	ENSG00000054793	ATP9A	
ENST00000511792	ENSG00000153071	DAB2	filled
ENST00000507990	ENSG00000153071	DAB2	
ENST00000526143	ENSG00000186174	BCL9L	
ENST00000532899	ENSG00000186174	BCL9L	
ENST00000383836	ENSG00000196220	SRGAP3	filled
ENST00000360413	ENSG00000196220	SRGAP3	filled
ENST00000433332	ENSG00000196220	SRGAP3	filled
ENST00000470951	ENSG00000196220	SRGAP3	filled
ENST00000544190	ENSG00000111490	TBC1D30	filled
ENST00000542120	ENSG00000111490	TBC1D30	filled
ENST00000368090	ENSG00000143315	PIGM	
ENST00000524258	ENSG00000182372	CLN8	
ENST00000587459	ENSG00000267168	RP11-318A15.7	
ENST00000372571	ENSG00000198815	FOXJ3	
ENST00000457052	ENSG00000237452	BHMG1	filled
ENST00000530793	ENSG00000110777	POU2AF1	
ENST00000525662	ENSG00000110777	POU2AF1	
ENST00000526535	ENSG00000110777	POU2AF1	
ENST00000525499	ENSG00000110777	POU2AF1	
ENST00000445628	ENSG00000100151	PICK1	
ENST00000464281	ENSG00000119862	LGALSL	
ENST00000238875	ENSG00000119862	LGALSL	
ENST00000409537	ENSG00000119862	LGALSL	
ENST00000374695	ENSG00000142798	HSPG2	filled
ENST00000368554	ENSG00000165828	PRAP1	filled
ENST00000342788	ENSG00000178568	ERBB4	filled
ENST00000436443	ENSG00000178568	ERBB4	filled
ENST00000484594	ENSG00000178568	ERBB4	filled
ENST00000260943	ENSG00000178568	ERBB4	filled
ENST00000435846	ENSG00000178568	ERBB4	filled
ENST00000459774	ENSG00000178568	ERBB4	
ENST00000402597	ENSG00000178568	ERBB4	filled
ENST00000462520	ENSG00000143458	GABPB2	
ENST00000489549	ENSG00000143458	GABPB2	
ENST00000486076	ENSG00000143458	GABPB2	
ENST00000368865	ENSG00000154473	BUB3	filled
ENST00000525525	ENSG00000117676	RPS6KA1	
ENST00000374168	ENSG00000117676	RPS6KA1	filled
ENST00000524436	ENSG00000117676	RPS6KA1	filled
ENST00000374166	ENSG00000117676	RPS6KA1	filled
ENST00000526792	ENSG00000117676	RPS6KA1	filled
ENST00000529454	ENSG00000117676	RPS6KA1	filled
ENST00000366866	ENSG00000117676	RPS6KA1	filled
ENST00000578982	ENSG00000198933	TBKBP1	
ENST00000480824	ENSG00000198663	C6orf89	

ENST00000586570	ENSG00000041353	RAB27B	filled
ENST00000592334	ENSG00000041353	RAB27B	
ENST00000417496	ENSG00000048052	HDAC9	filled
ENST00000455069	ENSG00000048052	HDAC9	filled
ENST00000433709	ENSG00000048052	HDAC9	filled
ENST00000411993	ENSG00000048052	HDAC9	
ENST00000413509	ENSG00000048052	HDAC9	filled
ENST00000461159	ENSG00000048052	HDAC9	filled
ENST00000474742	ENSG00000048052	HDAC9	filled
ENST00000548802	ENSG00000258064	RP11-293I14.2	
ENST00000338380	ENSG00000124107	SLPI	
ENST00000300005	ENSG00000196678	ERI2	
ENST00000562987	ENSG00000196678	ERI2	
ENST00000564349	ENSG00000196678	ERI2	filled
ENST00000563537	ENSG00000196678	ERI2	
ENST00000467425	ENSG00000196277	GRM7	filled
ENST00000357716	ENSG00000196277	GRM7	filled
ENST00000486284	ENSG00000196277	GRM7	filled
ENST00000440923	ENSG00000196277	GRM7	filled
ENST00000389336	ENSG00000196277	GRM7	filled
ENST00000389335	ENSG00000196277	GRM7	filled
ENST00000435689	ENSG00000196277	GRM7	filled
ENST00000488397	ENSG00000204160	ZDHHC18	
ENST00000471792	ENSG00000118939	UCHL3	filled
ENST00000458054	ENSG00000196950	SLC39A10	filled
ENST00000591864	ENSG00000092931	MFSD11	
ENST00000587661	ENSG00000092931	MFSD11	
ENST00000588031	ENSG00000092931	MFSD11	
ENST00000588670	ENSG00000092931	MFSD11	
ENST00000588768	ENSG00000092931	MFSD11	
ENST00000585584	ENSG00000092931	MFSD11	
ENST00000585958	ENSG00000092931	MFSD11	
ENST00000588647	ENSG00000092931	MFSD11	
ENST00000307201	ENSG00000168792	ABHD15	filled
ENST00000406053	ENSG00000115239	GPR75-ASB3	filled
ENST00000482339	ENSG00000115239	GPR75-ASB3	
ENST00000490794	ENSG00000115239	GPR75-ASB3	
ENST00000526615	ENSG00000149084	HSD17B12	filled
ENST00000532864	ENSG00000149084	HSD17B12	filled
ENST00000533358	ENSG00000149084	HSD17B12	filled
ENST00000511250	ENSG00000173320	STOX2	filled
ENST00000445946	ENSG00000227268	KLLN	
ENST00000502574	ENSG00000249240	AC069368.3	filled
ENST00000437723	ENSG00000249240	AC069368.3	filled
ENST00000552217	ENSG00000198015	MRPL42	
ENST00000487664	ENSG00000107147	KCNT1	filled
ENST00000371757	ENSG00000107147	KCNT1	filled
ENST00000460750	ENSG00000107147	KCNT1	filled
ENST00000473941	ENSG00000107147	KCNT1	filled
ENST00000486577	ENSG00000107147	KCNT1	filled
ENST00000434772	ENSG00000178386	ZNF223	
ENST00000585552	ENSG00000178386	ZNF223	
ENST00000593088	ENSG00000178386	ZNF223	
ENST00000591850	ENSG00000178386	ZNF223	
ENST00000525907	ENSG00000172508	CARNS1	
ENST00000299824	ENSG00000101445	PPP1R16B	filled
ENST00000373331	ENSG00000101445	PPP1R16B	filled
ENST00000540380	ENSG00000186642	PDE2A	filled
ENST00000535701	ENSG00000186642	PDE2A	filled
ENST00000542969	ENSG00000186642	PDE2A	filled

ENST00000546038	ENSG00000186642	PDE2A	filled
ENST00000370078	ENSG00000198890	PRMT6	
ENST00000375099	ENSG00000162543	UBXN10	
ENST00000533072	ENSG00000052841	TTC17	
ENST00000466370	ENSG00000243215	HLA-DMA	
ENST00000486785	ENSG00000243215	HLA-DMA	
ENST00000477571	ENSG00000157800	SLC37A3	
ENST00000591164	ENSG00000197483	ZNF628	
ENST00000391718	ENSG00000197483	ZNF628	
ENST00000619753	ENSG00000131044	TTLL9	
ENST00000545172	ENSG00000143199	ADCY10	filled
ENST00000367851	ENSG00000143199	ADCY10	filled
ENST00000367848	ENSG00000143199	ADCY10	filled
ENST00000476818	ENSG00000143199	ADCY10	
ENST00000529163	ENSG00000161016	RPL8	
ENST00000539554	ENSG00000111196	MAGOHB	
ENST00000368608	ENSG00000189241	TSPYL1	
ENST00000475045	ENSG00000159216	RUNX1	filled
ENST00000468726	ENSG00000159216	RUNX1	
ENST00000467692	ENSG00000159216	RUNX1	filled
ENST00000298705	ENSG00000165807	PPP1R36	
ENST00000467261	ENSG00000165807	PPP1R36	
ENST00000504595	ENSG00000183580	FBXL7	filled
ENST00000510662	ENSG00000183580	FBXL7	filled
ENST00000329673	ENSG00000183580	FBXL7	filled
ENST00000055682	ENSG00000050030	KIAA2022	filled
ENST00000616200	ENSG00000050030	KIAA2022	filled
ENST00000357447	ENSG00000151474	FRMD4A	filled
ENST00000475325	ENSG00000151474	FRMD4A	
ENST00000404989	ENSG00000188677	PARVB	filled
ENST00000477438	ENSG00000188677	PARVB	
ENST00000298552	ENSG00000165699	TSC1	
ENST00000493467	ENSG00000165699	TSC1	
ENST00000403810	ENSG00000165699	TSC1	
ENST00000475903	ENSG00000165699	TSC1	
ENST00000490179	ENSG00000165699	TSC1	
ENST00000370272	ENSG00000117505	DR1	
ENST00000490148	ENSG00000125879	OTOR	
ENST00000584574	ENSG00000108592	FTSJ3	
ENST00000322734	ENSG00000144792	ZNF660	
ENST00000523466	ENSG00000196743	GM2A	filled
ENST00000450434	ENSG00000178809	TRIM73	filled
ENST00000268125	ENSG00000140522	RLBP1	filled
ENST00000567787	ENSG00000140522	RLBP1	filled
ENST00000604534	ENSG00000271092	TMEM56-RWDD3	filled
ENST00000604203	ENSG00000271092	TMEM56-RWDD3	
ENST00000456523	ENSG00000138675	FGF5	filled
ENST00000480406	ENSG00000148688	RPP30	
ENST00000489806	ENSG00000148688	RPP30	
ENST00000378660	ENSG00000176034	CHDC2	filled
ENST00000355621	ENSG00000186591	UBE2H	filled
ENST00000473814	ENSG00000186591	UBE2H	filled
ENST00000496698	ENSG00000186591	UBE2H	filled
ENST00000472396	ENSG00000186591	UBE2H	filled
ENST00000490974	ENSG00000186591	UBE2H	filled
ENST00000589827	ENSG00000129657	SEC14L1	filled
ENST00000392476	ENSG00000129657	SEC14L1	filled
ENST00000569632	ENSG00000129657	SEC14L1	
ENST00000586429	ENSG00000129657	SEC14L1	filled
ENST00000586390	ENSG00000129657	SEC14L1	filled

ENST00000591437	ENSG00000129657	SEC14L1	filled
ENST00000520693	ENSG00000006837	CDKL3	filled
ENST00000519312	ENSG00000006837	CDKL3	filled
ENST00000594321	ENSG00000179954	SSC5D	
ENST00000511204	ENSG00000187695	RP11-723O4.6	filled
ENST00000367658	ENSG00000112406	HECA	filled
ENST00000303596	ENSG00000168286	THAP11	filled
ENST00000577339	ENSG00000167131	CCDC103	
ENST00000518659	ENSG00000145934	TENM2	filled
ENST00000520393	ENSG00000145934	TENM2	filled
ENST00000519204	ENSG00000145934	TENM2	filled
ENST00000520394	ENSG00000145934	TENM2	filled
ENST00000517586	ENSG00000145934	TENM2	filled
ENST00000334289	ENSG00000187151	ANGPTL5	filled
ENST00000617804	ENSG00000274322	RP11-314N13.10	filled
ENST00000381569	ENSG00000061337	LZTS1	filled
ENST00000265801	ENSG00000061337	LZTS1	
ENST00000275235	ENSG00000146416	AIG1	filled
ENST00000594353	ENSG00000118160	SLC8A2	
ENST00000375173	ENSG00000136925	TSTD2	
ENST00000544636	ENSG00000111254	AKAP3	
ENST00000359586	ENSG00000187908	DMBT1	
ENST00000582441	ENSG00000266202	RP1-66C13.4	filled
ENST00000611914	ENSG00000253485	PCDHGA5	
ENST00000518069	ENSG00000253485	PCDHGA5	filled
ENST00000395891	ENSG00000002746	HECW1	filled
ENST00000490954	ENSG00000002746	HECW1	filled
ENST00000453890	ENSG00000002746	HECW1	filled
ENST00000492310	ENSG00000002746	HECW1	filled
ENST00000471527	ENSG00000002746	HECW1	
ENST00000493057	ENSG00000002746	HECW1	
ENST00000464944	ENSG00000002746	HECW1	
ENST00000481031	ENSG00000002746	HECW1	
ENST00000511438	ENSG00000114656	KIAA1257	filled
ENST00000510149	ENSG00000114656	KIAA1257	filled
ENST00000617380	ENSG00000276547	PCDHGB5	filled
ENST00000490550	ENSG00000075945	KIFAP3	filled
ENST00000476529	ENSG00000107929	LARP4B	
ENST00000406525	ENSG00000107929	LARP4B	
ENST00000481118	ENSG00000107929	LARP4B	
ENST00000264933	ENSG00000249915	PDCD6	filled
ENST00000507528	ENSG00000249915	PDCD6	filled
ENST00000507473	ENSG00000249915	PDCD6	filled
ENST00000593531	ENSG00000268861	CTD-2207O23.3	filled
ENST00000617428	ENSG00000268861	CTD-2207O23.3	filled
ENST00000486257	ENSG00000181847	TIGIT	
ENST00000598278	ENSG00000214046	SMIM7	filled
ENST00000593409	ENSG00000214046	SMIM7	filled
ENST00000487416	ENSG00000214046	SMIM7	filled
ENST00000487803	ENSG00000214046	SMIM7	filled
ENST00000593404	ENSG00000214046	SMIM7	filled
ENST00000597711	ENSG00000214046	SMIM7	filled
ENST00000599310	ENSG00000214046	SMIM7	filled
ENST00000602194	ENSG00000214046	SMIM7	filled
ENST00000600740	ENSG00000214046	SMIM7	filled
ENST00000461488	ENSG00000214046	SMIM7	filled
ENST00000369817	ENSG00000147403	RPL10	
ENST00000451365	ENSG00000147403	RPL10	
ENST00000449494	ENSG00000147403	RPL10	
ENST00000427682	ENSG00000147403	RPL10	

ENST00000428169	ENSG00000147403	RPL10	
ENST00000392825	ENSG00000144278	GALNT13	filled
ENST00000434213	ENSG00000144278	GALNT13	filled
ENST00000409237	ENSG00000144278	GALNT13	filled
ENST00000422181	ENSG00000234809	NRM	
ENST00000371030	ENSG00000124203	ZNF831	
ENST00000436604	ENSG00000116133	DHCR24	
ENST00000308022	ENSG00000172663	TMEM134	
ENST00000616646	ENSG00000278053	DDX52	filled
ENST00000309623	ENSG00000173679	OR1L1	
ENST00000373686	ENSG00000173679	OR1L1	
ENST00000608634	ENSG00000183785	TUBA8	filled
ENST00000259021	ENSG00000136504	KAT7	
ENST00000509124	ENSG00000136504	KAT7	
ENST00000460467	ENSG00000154822	PLCL2	filled
ENST00000242462	ENSG00000122859	NEUROG3	
ENST00000437078	ENSG00000154710	RABGEF1	filled
ENST00000510829	ENSG00000154710	RABGEF1	filled
ENST00000562221	ENSG00000111913	FAM65B	
ENST00000538035	ENSG00000111913	FAM65B	filled
ENST00000613507	ENSG00000111913	FAM65B	filled
ENST00000510784	ENSG00000111913	FAM65B	filled
ENST00000579820	ENSG00000177576	C18orf32	
ENST00000485705	ENSG00000156990	RPUSD3	
ENST00000398610	ENSG00000253846	PCDHGA10	filled
ENST00000475157	ENSG00000159388	BTG2	
ENST00000290551	ENSG00000159388	BTG2	
ENST00000561267	ENSG00000092439	TRPM7	filled
ENST00000454782	ENSG00000179165	PXT1	filled
ENST00000480040	ENSG00000147576	ADHFE1	
ENST00000489835	ENSG00000224940	PRRT4	
ENST00000480290	ENSG00000224940	PRRT4	
ENST00000446477	ENSG00000224940	PRRT4	
ENST00000535159	ENSG00000224940	PRRT4	
ENST00000302628	ENSG00000172137	CALB2	filled
ENST00000562305	ENSG00000172137	CALB2	filled
ENST00000349553	ENSG00000172137	CALB2	filled
ENST00000587481	ENSG00000102898	NUTF2	
ENST00000327470	ENSG00000183161	FANCF	
ENST00000319670	ENSG00000104880	ARHGEF18	filled
ENST00000599752	ENSG00000104880	ARHGEF18	filled
ENST00000374719	ENSG00000131080	EDA2R	filled
ENST00000396050	ENSG00000131080	EDA2R	filled
ENST00000521391	ENSG00000113645	WWC1	
ENST00000399560	ENSG00000169981	ZNF35	
ENST00000453164	ENSG00000169981	ZNF35	
ENST00000472766	ENSG00000182533	CAV3	filled
ENST00000496568	ENSG00000178149	DALRD3	
ENST00000492585	ENSG00000178149	DALRD3	
ENST00000528582	ENSG00000148935	GAS2	filled
ENST00000423003	ENSG00000206331	PRRT1	
ENST00000491691	ENSG00000206331	PRRT1	
ENST00000462841	ENSG00000206331	PRRT1	
ENST00000473568	ENSG00000206331	PRRT1	
ENST00000596516	ENSG00000230667	SETSIP	
ENST00000540908	ENSG00000149506	ZP1	
ENST00000537203	ENSG00000149506	ZP1	
ENST00000543020	ENSG00000149506	ZP1	
ENST00000620319	ENSG00000149506	ZP1	
ENST00000455656	ENSG00000152078	TMEM56	

ENST00000503362	ENSG00000129128	SPCS3	
ENST00000503271	ENSG00000145362	ANK2	filled
ENST00000503423	ENSG00000145362	ANK2	filled
ENST00000506722	ENSG00000145362	ANK2	filled
ENST00000504454	ENSG00000145362	ANK2	filled
ENST00000514246	ENSG00000145362	ANK2	
ENST00000394537	ENSG00000145362	ANK2	filled
ENST00000357077	ENSG00000145362	ANK2	filled
ENST00000264366	ENSG00000145362	ANK2	filled
ENST00000508613	ENSG00000145362	ANK2	filled
ENST00000512999	ENSG00000145362	ANK2	
ENST00000511380	ENSG00000145362	ANK2	filled
ENST00000471514	ENSG00000235307	BRD2	
ENST00000552587	ENSG00000235307	BRD2	
ENST00000474657	ENSG00000088832	FKBP1A	
ENST00000562383	ENSG00000166164	BRD7	
ENST00000569774	ENSG00000166164	BRD7	
ENST00000465232	ENSG00000179988	PSTK	filled
ENST00000497219	ENSG00000179988	PSTK	filled
ENST00000496079	ENSG00000179988	PSTK	
ENST00000482853	ENSG00000124596	OARD1	
ENST00000479950	ENSG00000124596	OARD1	
ENST00000488238	ENSG00000124596	OARD1	
ENST00000592326	ENSG00000055483	USP36	
ENST00000604790	ENSG00000269405	NXF2	filled
ENST00000512208	ENSG00000177058	SLC38A9	filled
ENST00000514806	ENSG00000177058	SLC38A9	
ENST00000513993	ENSG00000177058	SLC38A9	
ENST00000513275	ENSG00000177058	SLC38A9	
ENST00000371191	ENSG00000162599	NFIA	filled
ENST00000461975	ENSG00000230995	PPP1R10	
ENST00000600586	ENSG00000186838	SELV	
ENST00000549190	ENSG00000134283	PPHLN1	filled
ENST00000551723	ENSG00000134283	PPHLN1	
ENST00000547440	ENSG00000134283	PPHLN1	filled
ENST00000338051	ENSG00000181904	C5orf24	
ENST00000517581	ENSG00000155096	AZIN1	filled
ENST00000510645	ENSG00000109606	DHX15	filled
ENST00000356020	ENSG00000198685	LINC01565	
ENST00000521475	ENSG00000156011	PSD3	
ENST00000615278	ENSG00000135119	RNFT2	
ENST00000527848	ENSG00000166483	WEE1	filled
ENST00000474125	ENSG00000186862	PDZD7	filled
ENST00000433616	ENSG00000186862	PDZD7	filled
ENST00000476306	ENSG00000186862	PDZD7	filled
ENST00000619208	ENSG00000186862	PDZD7	filled
ENST00000280684	ENSG00000151079	KCNA6	
ENST00000437147	ENSG00000147133	TAF1	filled
ENST00000373775	ENSG00000147133	TAF1	filled
ENST00000462588	ENSG00000147133	TAF1	filled
ENST00000485087	ENSG00000147133	TAF1	filled
ENST00000468167	ENSG00000147133	TAF1	filled
ENST00000461764	ENSG00000147133	TAF1	filled
ENST00000316249	ENSG00000178342	KCNG2	filled
ENST00000568850	ENSG00000103769	RAB11A	filled
ENST00000425975	ENSG00000161180	CCDC116	
ENST00000568288	ENSG00000103275	UBE2I	
ENST00000566587	ENSG00000103275	UBE2I	
ENST00000470310	ENSG00000163710	PCOLCE2	
ENST00000358105	ENSG00000197826	C4orf22	filled

ENST00000512931	ENSG00000197826	C4orf22	filled
ENST00000514249	ENSG00000197826	C4orf22	filled
ENST00000502497	ENSG00000197826	C4orf22	filled
ENST00000508675	ENSG00000197826	C4orf22	filled
ENST00000503883	ENSG00000197826	C4orf22	filled
ENST00000512560	ENSG00000177034	MTX3	
ENST00000617335	ENSG00000177034	MTX3	
ENST00000520717	ENSG00000172164	SNTB1	
ENST00000470747	ENSG00000257184	HOXA10-HOXA9	filled
ENST00000263468	ENSG00000110318	KIAA1377	filled
ENST00000532529	ENSG00000110318	KIAA1377	filled
ENST00000532077	ENSG00000110318	KIAA1377	filled
ENST00000168712	ENSG00000075388	FGF4	
ENST00000564347	ENSG00000080854	IGSF9B	
ENST00000533871	ENSG00000080854	IGSF9B	filled
ENST00000530957	ENSG00000080854	IGSF9B	
ENST00000469954	ENSG00000043093	DCUN1D1	
ENST00000497606	ENSG00000043093	DCUN1D1	
ENST00000460412	ENSG00000043093	DCUN1D1	
ENST00000567908	ENSG00000153048	CARHSP1	
ENST00000570125	ENSG00000153048	CARHSP1	
ENST00000568968	ENSG00000153048	CARHSP1	
ENST00000398594	ENSG00000254122	PCDHGB7	filled
ENST00000487384	ENSG00000078399	HOXA9	filled
ENST00000465941	ENSG00000078399	HOXA9	filled
ENST00000492376	ENSG00000065154	OAT	
ENST00000467711	ENSG00000162735	PEX19	
ENST00000533699	ENSG00000162735	PEX19	
ENST00000375754	ENSG00000165025	SYK	filled
ENST00000375751	ENSG00000165025	SYK	filled
ENST00000375746	ENSG00000165025	SYK	filled
ENST00000478553	ENSG00000101265	RASSF2	filled
ENST00000457587	ENSG00000161057	PSMC2	
ENST00000425206	ENSG00000161057	PSMC2	
ENST00000600078	ENSG00000167578	RAB4B	
ENST00000487284	ENSG00000257923	CUX1	filled
ENST00000309315	ENSG00000122515	ZMIZ2	filled
ENST00000441627	ENSG00000122515	ZMIZ2	filled
ENST00000466424	ENSG00000164054	SHISA5	
ENST00000612611	ENSG00000164054	SHISA5	
ENST00000301149	ENSG00000167588	GPD1	filled
ENST00000281171	ENSG00000151490	PTPRO	filled
ENST00000543886	ENSG00000151490	PTPRO	filled
ENST00000348962	ENSG00000151490	PTPRO	filled
ENST00000606788	ENSG00000064601	CTSA	
ENST00000412402	ENSG00000128573	FOXP2	filled
ENST00000440349	ENSG00000128573	FOXP2	filled
ENST00000441290	ENSG00000128573	FOXP2	filled
ENST00000495516	ENSG00000128573	FOXP2	filled
ENST00000380390	ENSG00000167346	MMP26	filled
ENST00000589400	ENSG00000095066	HOOK2	filled
ENST00000592079	ENSG00000095066	HOOK2	filled
ENST00000590839	ENSG00000095066	HOOK2	filled
ENST00000591641	ENSG00000095066	HOOK2	
ENST00000593143	ENSG00000095066	HOOK2	filled
ENST00000592512	ENSG00000095066	HOOK2	
ENST00000580170	ENSG00000173482	PTPRM	filled
ENST00000332175	ENSG00000173482	PTPRM	filled
ENST00000348120	ENSG00000182901	RGS7	filled
ENST00000366563	ENSG00000182901	RGS7	filled

ENST00000366564	ENSG00000182901	RGS7	filled
ENST00000366565	ENSG00000182901	RGS7	filled
ENST00000421462	ENSG00000144283	PKP4	filled
ENST00000462335	ENSG00000144283	PKP4	filled
ENST00000513748	ENSG00000182575	NXPH3	
ENST00000570453	ENSG00000182575	NXPH3	
ENST00000511871	ENSG00000112246	SIM1	
ENST00000520117	ENSG00000132554	RGS22	
ENST00000485506	ENSG00000177707	PVRL3	filled
ENST00000518838	ENSG00000164841	TMEM74	filled
ENST00000602540	ENSG00000139289	PHLDA1	
ENST00000266671	ENSG00000139289	PHLDA1	
ENST00000619060	ENSG00000139289	PHLDA1	
ENST00000519556	ENSG00000172167	MTBP	
ENST00000519841	ENSG00000172167	MTBP	
ENST00000261937	ENSG00000037280	FLT4	
ENST00000393347	ENSG00000037280	FLT4	
ENST00000502649	ENSG00000037280	FLT4	
ENST00000424276	ENSG00000037280	FLT4	
ENST00000502293	ENSG00000037280	FLT4	
ENST00000513527	ENSG00000037280	FLT4	
ENST00000619105	ENSG00000037280	FLT4	
ENST00000503945	ENSG00000118579	MED28	
ENST00000499786	ENSG00000118579	MED28	
ENST00000572659	ENSG00000262102	DSEL	
ENST00000596022	ENSG00000104951	IL4I1	filled
ENST00000392645	ENSG00000204842	ATXN2	filled
ENST00000250101	ENSG00000129235	TXNDC17	
ENST00000477014	ENSG00000196507	TCEAL3	filled
ENST00000434116	ENSG00000074621	SLC24A1	filled
ENST00000537259	ENSG00000074621	SLC24A1	filled
ENST00000485080	ENSG00000072310	SREBF1	filled
ENST00000578469	ENSG00000072310	SREBF1	filled
ENST00000408926	ENSG00000221947	XKR9	filled
ENST00000520273	ENSG00000221947	XKR9	filled
ENST00000222339	ENSG00000105732	ZNF574	filled
ENST00000558280	ENSG00000129535	NRL	
ENST00000349937	ENSG00000148737	TCF7L2	
ENST00000369397	ENSG00000148737	TCF7L2	
ENST00000369395	ENSG00000148737	TCF7L2	
ENST00000536810	ENSG00000148737	TCF7L2	
ENST00000534894	ENSG00000148737	TCF7L2	
ENST00000355717	ENSG00000148737	TCF7L2	
ENST00000352065	ENSG00000148737	TCF7L2	
ENST00000545257	ENSG00000148737	TCF7L2	
ENST00000355995	ENSG00000148737	TCF7L2	
ENST00000543371	ENSG00000148737	TCF7L2	
ENST00000542695	ENSG00000148737	TCF7L2	
ENST00000538897	ENSG00000148737	TCF7L2	
ENST00000327835	ENSG00000164684	ZNF704	filled
ENST00000517379	ENSG00000164684	ZNF704	
ENST00000519936	ENSG00000164684	ZNF704	filled
ENST00000543077	ENSG00000111241	FGF6	
ENST00000409037	ENSG00000125122	LRRC29	filled
ENST00000371518	ENSG00000020256	ZFP64	filled
ENST00000371523	ENSG00000020256	ZFP64	filled
ENST00000461898	ENSG00000020256	ZFP64	
ENST00000359651	ENSG00000163435	ELF3	
ENST00000498017	ENSG00000163435	ELF3	filled
ENST00000465882	ENSG00000114030	KPNA1	

ENST00000476916	ENSG00000114030	KPNA1	
ENST00000597641	ENSG00000105287	PRKD2	
ENST00000598633	ENSG00000105287	PRKD2	
ENST00000593363	ENSG00000105287	PRKD2	
ENST00000579248	ENSG00000265681	RPL17	
ENST00000314523	ENSG00000188612	SUMO2	filled
ENST00000578238	ENSG00000188612	SUMO2	
ENST00000470908	ENSG00000198625	MDM4	
ENST00000471783	ENSG00000198625	MDM4	
ENST00000507825	ENSG00000198625	MDM4	
ENST00000544050	ENSG00000010219	DYRK4	
ENST00000319119	ENSG00000177125	ZBTB34	
ENST00000373452	ENSG00000177125	ZBTB34	
ENST00000570498	ENSG00000262635	TDO2	
ENST00000574434	ENSG00000262635	TDO2	
ENST00000573644	ENSG00000262635	TDO2	
ENST00000546840	ENSG00000257767	RP11-162P23.2	filled
ENST00000269033	ENSG00000141298	SSH2	filled
ENST00000540801	ENSG00000141298	SSH2	filled
ENST00000394848	ENSG00000141298	SSH2	filled
ENST00000579040	ENSG00000141298	SSH2	filled
ENST00000324677	ENSG00000141298	SSH2	filled
ENST00000577483	ENSG00000141298	SSH2	filled
ENST00000592397	ENSG00000141298	SSH2	filled
ENST00000578411	ENSG00000141298	SSH2	filled
ENST00000582084	ENSG00000141298	SSH2	
ENST00000590153	ENSG00000141298	SSH2	
ENST00000579954	ENSG00000141298	SSH2	
ENST00000532088	ENSG00000129083	COPB1	
ENST00000525214	ENSG00000129083	COPB1	
ENST00000474897	ENSG00000215193	PEX26	filled
ENST00000454109	ENSG00000225556	C2CD4D	
ENST00000459812	ENSG00000254875	DAQB-143M3.10	
ENST00000525403	ENSG00000167323	STIM1	filled
ENST00000527651	ENSG00000167323	STIM1	filled
ENST00000532610	ENSG00000167323	STIM1	
ENST00000532919	ENSG00000167323	STIM1	
ENST00000530554	ENSG00000167323	STIM1	filled
ENST00000524822	ENSG00000167323	STIM1	filled
ENST00000525055	ENSG00000167323	STIM1	filled
ENST00000528656	ENSG00000167323	STIM1	filled
ENST00000373131	ENSG00000126733	DACH2	filled
ENST00000461604	ENSG00000126733	DACH2	filled
ENST00000506327	ENSG00000126733	DACH2	filled
ENST00000373125	ENSG00000126733	DACH2	filled
ENST00000508860	ENSG00000126733	DACH2	filled
ENST00000503338	ENSG00000163781	TOPBP1	filled
ENST00000503464	ENSG00000163781	TOPBP1	filled
ENST00000361505	ENSG00000198805	PNP	
ENST00000294740	ENSG00000162702	ZNF281	
ENST00000523718	ENSG00000129422	MTUS1	filled
ENST00000518891	ENSG00000129422	MTUS1	
ENST00000381869	ENSG00000129422	MTUS1	filled
ENST00000598673	ENSG00000174562	KLK15	
ENST00000495013	ENSG00000136146	MED4	filled
ENST00000344096	ENSG00000127334	DYRK2	filled
ENST00000558325	ENSG00000259371	RP11-468E2.6	
ENST00000391830	ENSG00000204673	AKT1S1	
ENST00000482622	ENSG00000204673	AKT1S1	
ENST00000562116	ENSG00000039523	FAM65A	filled

ENST00000562755	ENSG00000039523	FAM65A	
ENST00000379312	ENSG00000039523	FAM65A	filled
ENST00000566522	ENSG00000039523	FAM65A	
ENST00000042381	ENSG00000039523	FAM65A	filled
ENST00000540839	ENSG00000039523	FAM65A	filled
ENST00000566907	ENSG00000039523	FAM65A	
ENST00000566920	ENSG00000039523	FAM65A	
ENST00000566815	ENSG00000039523	FAM65A	
ENST00000561534	ENSG00000039523	FAM65A	
ENST00000569474	ENSG00000039523	FAM65A	
ENST00000569179	ENSG00000039523	FAM65A	
ENST00000569733	ENSG00000039523	FAM65A	
ENST00000422602	ENSG00000039523	FAM65A	filled
ENST00000409947	ENSG00000169967	MAP3K2	
ENST00000409179	ENSG00000169967	MAP3K2	
ENST00000435819	ENSG00000135218	CD36	filled
ENST00000396252	ENSG00000104320	NBN	filled
ENST00000381980	ENSG00000154274	C4orf19	filled
ENST00000284437	ENSG00000154274	C4orf19	filled
ENST00000578431	ENSG00000082397	EPB41L3	
ENST00000520539	ENSG00000104517	UBR5	filled
ENST00000220959	ENSG00000104517	UBR5	filled
ENST00000521922	ENSG00000104517	UBR5	filled
ENST00000361303	ENSG00000168675	LDLRAD4	filled
ENST00000435606	ENSG00000168675	LDLRAD4	filled
ENST00000427592	ENSG00000100156	SLC16A8	
ENST00000376462	ENSG00000120549	KIAA1217	filled
ENST00000481700	ENSG00000120549	KIAA1217	filled
ENST00000509101	ENSG00000163739	CXCL1	filled
ENST00000515643	ENSG00000143603	KCNN3	
ENST00000491148	ENSG00000153002	CPB1	filled
ENST00000494888	ENSG00000153002	CPB1	filled
ENST00000462345	ENSG00000153002	CPB1	
ENST00000528481	ENSG00000077514	POLD3	filled
ENST00000524752	ENSG00000077514	POLD3	
ENST00000412253	ENSG00000003137	CYP26B1	
ENST00000257621	ENSG00000005981	ASB4	filled
ENST00000261263	ENSG00000080371	RAB21	filled
ENST00000399875	ENSG00000183307	CECR6	filled
ENST00000570941	ENSG00000273092	TAS2R20	
ENST00000588374	ENSG00000108883	EFTUD2	
ENST00000426518	ENSG00000114026	OGG1	filled
ENST00000437574	ENSG00000229071	PRRT1	
ENST00000470230	ENSG00000229071	PRRT1	
ENST00000471346	ENSG00000229071	PRRT1	
ENST00000476447	ENSG00000229071	PRRT1	
ENST00000536742	ENSG00000183273	CCDC60	filled
ENST00000327554	ENSG00000183273	CCDC60	filled
ENST00000539847	ENSG00000183273	CCDC60	filled
ENST00000360997	ENSG00000168309	FAM107A	
ENST00000394481	ENSG00000168309	FAM107A	
ENST00000464064	ENSG00000168309	FAM107A	
ENST00000474531	ENSG00000168309	FAM107A	filled
ENST00000447756	ENSG00000168309	FAM107A	
ENST00000465970	ENSG00000168309	FAM107A	filled
ENST00000497310	ENSG00000168309	FAM107A	
ENST00000593039	ENSG00000267618	RAD51L3-RFFL	filled
ENST00000592181	ENSG00000267618	RAD51L3-RFFL	filled
ENST00000597959	ENSG00000269711	CTD-3214H19.16	
ENST00000436261	ENSG00000178917	ZNF852	

ENST00000489411	ENSG00000178917	ZNF852	
ENST00000463067	ENSG00000178917	ZNF852	
ENST00000616809	ENSG00000178917	ZNF852	
ENST00000426490	ENSG00000175877	WBSCR28	filled
ENST00000469768	ENSG00000144893	MED12L	
ENST00000422248	ENSG00000144893	MED12L	
ENST00000600245	ENSG00000105732	ZNF574	filled
ENST00000597391	ENSG00000105732	ZNF574	
ENST00000359044	ENSG00000105732	ZNF574	filled
ENST00000613064	ENSG00000105732	ZNF574	filled
ENST00000618626	ENSG00000076351	SLC46A1	
ENST00000584995	ENSG00000076351	SLC46A1	
ENST00000584426	ENSG00000076351	SLC46A1	
ENST00000581516	ENSG00000076351	SLC46A1	
ENST00000624166	ENSG00000085224	ATRX	filled
ENST00000624032	ENSG00000085224	ATRX	filled
ENST00000432462	ENSG00000232421	TUBB	
ENST00000427480	ENSG00000232421	TUBB	
ENST00000583104	ENSG00000108666	C17orf75	
ENST00000369864	ENSG00000181754	AMIGO1	
ENST00000495321	ENSG00000116698	SMG7	filled
ENST00000555089	ENSG00000119689	DLST	
ENST00000461327	ENSG00000171848	RRM2	
ENST00000560908	ENSG00000140548	ZNF710	
ENST00000409448	ENSG00000144214	LYG1	filled
ENST00000484894	ENSG00000100142	POLR2F	
ENST00000407936	ENSG00000100142	POLR2F	
ENST00000443002	ENSG00000100142	POLR2F	
ENST00000333418	ENSG00000100142	POLR2F	filled
ENST00000427034	ENSG00000100142	POLR2F	filled
ENST00000553522	ENSG00000176153	GPX2	
ENST00000389614	ENSG00000176153	GPX2	
ENST00000557049	ENSG00000176153	GPX2	
ENST00000557323	ENSG00000176153	GPX2	
ENST00000612794	ENSG00000176153	GPX2	
ENST00000477617	ENSG00000143578	CREB3L4	
ENST00000567078	ENSG00000260342	RP11-1035H13.3	filled
ENST00000517415	ENSG00000181195	PENK	
ENST00000427067	ENSG00000136960	ENPP2	
ENST00000308448	ENSG00000138138	ATAD1	filled
ENST00000328142	ENSG00000138138	ATAD1	filled
ENST00000495903	ENSG00000138138	ATAD1	filled
ENST00000422537	ENSG00000158411	MITD1	
ENST00000477982	ENSG00000005812	FBXL3	
ENST00000443638	ENSG00000077147	TM9SF3	
ENST00000592304	ENSG00000188554	NBR1	
ENST00000585505	ENSG00000188554	NBR1	filled
ENST00000540941	ENSG00000175768	TOMM5	
ENST00000321949	ENSG00000105662	CRTC1	
ENST00000594658	ENSG00000105662	CRTC1	
ENST00000601916	ENSG00000105662	CRTC1	
ENST00000545509	ENSG00000111203	ITFG2	
ENST00000585333	ENSG00000132481	TRIM47	
ENST00000523463	ENSG00000076641	PAG1	
ENST00000359546	ENSG00000145920	CPLX2	filled
ENST00000506642	ENSG00000145920	CPLX2	
ENST00000453258	ENSG00000138185	ENTPD1	filled
ENST00000432059	ENSG00000120519	SLC10A7	filled
ENST00000335472	ENSG00000120519	SLC10A7	filled
ENST00000507030	ENSG00000120519	SLC10A7	filled

ENST00000507560	ENSG00000120519	SLC10A7	filled
ENST00000394059	ENSG00000120519	SLC10A7	
ENST00000511315	ENSG00000120519	SLC10A7	
ENST00000511374	ENSG00000120519	SLC10A7	
ENST00000513583	ENSG00000120519	SLC10A7	
ENST00000409105	ENSG00000180398	MCFD2	
ENST00000409913	ENSG00000180398	MCFD2	
ENST00000319466	ENSG00000180398	MCFD2	
ENST00000409800	ENSG00000180398	MCFD2	
ENST00000467654	ENSG00000143226	FCGR2A	filled
ENST00000536731	ENSG00000143226	FCGR2A	filled
ENST00000497474	ENSG00000143226	FCGR2A	filled
ENST00000482233	ENSG00000143226	FCGR2A	filled
ENST00000561305	ENSG00000166734	CASC4	
ENST00000325602	ENSG00000181631	P2RY13	
ENST00000507402	ENSG00000131732	ZCCHC9	
ENST00000304552	ENSG00000172215	CXCR6	
ENST00000309237	ENSG00000144893	MED12L	
ENST00000474524	ENSG00000144893	MED12L	filled
ENST00000273432	ENSG00000144893	MED12L	filled
ENST00000468305	ENSG00000144893	MED12L	filled
ENST00000399177	ENSG00000166960	CCDC178	
ENST00000485270	ENSG00000151224	MAT1A	
ENST00000480845	ENSG00000151224	MAT1A	
ENST00000391352	ENSG00000212657	KRTAP16-1	
ENST00000570754	ENSG00000214226	C17orf67	filled
ENST00000528396	ENSG00000014216	CAPN1	
ENST00000527739	ENSG00000014216	CAPN1	
ENST00000526966	ENSG00000014216	CAPN1	
ENST00000528739	ENSG00000014216	CAPN1	
ENST00000532285	ENSG00000014216	CAPN1	
ENST00000527469	ENSG00000014216	CAPN1	
ENST00000534373	ENSG00000014216	CAPN1	
ENST00000531068	ENSG00000014216	CAPN1	
ENST00000527699	ENSG00000014216	CAPN1	
ENST00000533909	ENSG00000014216	CAPN1	
ENST00000377868	ENSG00000125864	BFSP1	filled
ENST00000473415	ENSG00000125864	BFSP1	filled
ENST00000536626	ENSG00000125864	BFSP1	filled
ENST00000453441	ENSG00000215018	COL28A1	filled
ENST00000444457	ENSG00000105819	PMPCB	
ENST00000474597	ENSG00000124783	SSR1	filled
ENST00000475213	ENSG00000124783	SSR1	
ENST00000488834	ENSG00000124783	SSR1	
ENST00000424992	ENSG00000118242	MREG	
ENST00000420348	ENSG00000118242	MREG	
ENST00000544838	ENSG00000139437	TCHP	filled
ENST00000549524	ENSG00000139437	TCHP	
ENST00000550780	ENSG00000139437	TCHP	filled
ENST00000512716	ENSG00000138670	RASGEF1B	filled
ENST00000557234	ENSG00000080824	HSP90AA1	filled
ENST00000594136	ENSG00000171570	RAB4B-EGLN2	filled
ENST00000576789	ENSG00000263264	CTB-133G6.1	filled
ENST00000270530	ENSG00000142459	EVI5L	filled
ENST00000472718	ENSG00000132153	DHX30	
ENST00000445061	ENSG00000132153	DHX30	
ENST00000395745	ENSG00000132153	DHX30	
ENST00000348968	ENSG00000132153	DHX30	
ENST00000327946	ENSG00000182771	GRID1	filled
ENST00000464741	ENSG00000182771	GRID1	filled
ENST00000453615	ENSG00000159352	PSMD4	
ENST00000359227	ENSG00000196361	ELAVL3	filled
ENST00000588853	ENSG00000196361	ELAVL3	
ENST00000592218	ENSG00000196361	ELAVL3	
ENST00000300087	ENSG00000166847	DCTN5	filled
ENST00000563614	ENSG00000166847	DCTN5	
ENST00000504330	ENSG00000109794	FAM149A	
ENST00000578289	ENSG00000006042	TMEM98	
ENST00000447607	ENSG00000198125	MB	
ENST00000405858	ENSG00000164631	ZNF12	filled
ENST00000342651	ENSG00000164631	ZNF12	filled
ENST00000576708	ENSG00000108559	NUP88	
ENST00000470688	ENSG00000159409	CELF3	
ENST00000290583	ENSG00000159409	CELF3	
ENST00000303592	ENSG00000168135	KCNJ4	
ENST00000402510	ENSG00000250423	KIAA1210	filled
ENST00000525061	ENSG00000137573	SULF1	
ENST00000528286	ENSG00000137573	SULF1	
ENST00000529134	ENSG00000137573	SULF1	
ENST00000368520	ENSG00000130649	CYP2E1	
ENST00000514302	ENSG00000155269	GPR78	filled
ENST00000392035	ENSG00000160392	C19orf47	filled
ENST00000580606	ENSG00000160392	C19orf47	filled
ENST00000449417	ENSG00000241296	HLA-DMB	filled
ENST00000298351	ENSG00000165548	TMEM63C	filled
ENST00000528274	ENSG00000255561	FDXACB1	
ENST00000471204	ENSG00000116251	RPL22	
ENST00000465335	ENSG00000116251	RPL22	
ENST00000261726	ENSG00000111249	CUX2	filled
ENST00000279488	ENSG00000139318	DUSP6	
ENST00000468888	ENSG00000163539	CLASP2	filled
ENST00000399362	ENSG00000163539	CLASP2	filled
ENST00000480013	ENSG00000163539	CLASP2	filled
ENST00000461133	ENSG00000163539	CLASP2	filled
ENST00000313350	ENSG00000163539	CLASP2	
ENST00000487200	ENSG00000163539	CLASP2	
ENST00000333778	ENSG00000163539	CLASP2	
ENST00000485378	ENSG00000163539	CLASP2	
ENST00000496954	ENSG00000163539	CLASP2	
ENST00000476433	ENSG00000163539	CLASP2	
ENST00000482896	ENSG00000163539	CLASP2	
ENST00000498331	ENSG00000163539	CLASP2	
ENST00000486796	ENSG00000163539	CLASP2	
ENST00000462878	ENSG00000163539	CLASP2	
ENST00000467956	ENSG00000163539	CLASP2	
ENST00000359576	ENSG00000163539	CLASP2	filled
ENST00000373248	ENSG00000042286	AIFM2	filled
ENST00000346807	ENSG00000136695	IL36RN	
ENST00000393200	ENSG00000136695	IL36RN	
ENST00000514072	ENSG00000136695	IL36RN	
ENST00000453129	ENSG00000228607	CLDN25	
ENST00000473790	ENSG00000188001	TPRG1	
ENST00000460613	ENSG00000188001	TPRG1	filled
ENST00000526829	ENSG00000160695	VPS11	filled
ENST00000398604	ENSG00000253767	PCDHGA8	filled
ENST00000529290	ENSG00000146670	CDCA5	
ENST00000525464	ENSG00000146670	CDCA5	
ENST00000533015	ENSG00000146670	CDCA5	filled
ENST00000275517	ENSG00000146670	CDCA5	
ENST00000607283	ENSG00000272305	RP11-894J14.5	filled

ENST00000519382	ENSG00000196459	TRAPPC2	
ENST00000517553	ENSG00000196459	TRAPPC2	
ENST00000519885	ENSG00000196459	TRAPPC2	
ENST00000595863	ENSG00000105143	SLC1A6	filled
ENST00000304954	ENSG00000171209	CSN3	
ENST00000404321	ENSG00000155093	PTPRN2	filled
ENST00000556492	ENSG00000139977	NAA30	filled
ENST00000564704	ENSG00000135740	SLC9A5	
ENST00000321702	ENSG00000177191	B3GNT8	
ENST00000590400	ENSG00000197256	KANK2	
ENST00000306422	ENSG00000171540	OTP	
ENST00000254616	ENSG00000132286	TIMM10B	
ENST00000542678	ENSG00000150967	ABCB9	filled
ENST00000587869	ENSG00000115266	APC2	
ENST00000590877	ENSG00000115266	APC2	
ENST00000587149	ENSG00000115266	APC2	
ENST00000412237	ENSG00000083099	LYRM2	
ENST00000523793	ENSG00000083099	LYRM2	
ENST00000520441	ENSG00000083099	LYRM2	
ENST00000523377	ENSG00000083099	LYRM2	
ENST00000584856	ENSG00000161395	PGAP3	filled
ENST00000619169	ENSG00000161395	PGAP3	
ENST00000420611	ENSG00000138068	SULT6B1	
ENST00000536005	ENSG00000166546	BEAN1	filled
ENST00000562849	ENSG00000166546	BEAN1	filled
ENST00000299694	ENSG00000166546	BEAN1	filled
ENST00000618932	ENSG00000166546	BEAN1	
ENST00000521648	ENSG00000172172	MRPL13	filled
ENST00000393965	ENSG00000135637	CCDC142	filled
ENST00000290418	ENSG00000135637	CCDC142	filled
ENST00000600209	ENSG00000130475	FCHO1	filled
ENST00000597474	ENSG00000130475	FCHO1	filled
ENST00000593833	ENSG00000130475	FCHO1	filled
ENST00000601247	ENSG00000130475	FCHO1	filled
ENST00000585073	ENSG00000132141	CCT6B	
ENST00000564457	ENSG00000102897	LYRM1	
ENST00000612932	ENSG00000274211	SOCS7	
ENST00000377022	ENSG00000130940	CASZ1	filled
ENST00000344008	ENSG00000130940	CASZ1	filled
ENST00000472814	ENSG00000130940	CASZ1	
ENST00000440959	ENSG00000230124	LHX4-AS1	
ENST00000622400	ENSG00000230124	LHX4-AS1	
ENST00000589615	ENSG00000189164	ZNF527	filled
ENST00000264888	ENSG00000138755	CXCL9	
ENST00000490638	ENSG00000137135	ARHGEF39	filled
ENST00000424806	ENSG00000225824	HLA-DQB1	
ENST00000562798	ENSG00000099364	FBXL19	
ENST00000254963	ENSG00000132622	HSPA12B	
ENST00000399701	ENSG00000132622	HSPA12B	
ENST00000470866	ENSG00000132465	IGJ	
ENST00000268616	ENSG00000140948	ZCCHC14	filled
ENST00000568020	ENSG00000140948	ZCCHC14	filled
ENST00000370905	ENSG00000124743	KLHL31	
ENST00000510920	ENSG00000237765	FAM200B	
ENST00000504823	ENSG00000237765	FAM200B	
ENST00000504137	ENSG00000237765	FAM200B	
ENST00000515697	ENSG00000237765	FAM200B	
ENST00000509022	ENSG00000237765	FAM200B	
ENST00000510032	ENSG00000237765	FAM200B	
ENST00000512855	ENSG00000237765	FAM200B	

ENST00000515430	ENSG00000237765	FAM200B	
ENST00000505260	ENSG00000237765	FAM200B	
ENST00000504598	ENSG00000237765	FAM200B	
ENST00000502502	ENSG00000237765	FAM200B	
ENST00000503617	ENSG00000237765	FAM200B	
ENST00000507305	ENSG00000237765	FAM200B	
ENST00000513053	ENSG00000237765	FAM200B	
ENST00000422728	ENSG00000237765	FAM200B	
ENST00000514803	ENSG00000237765	FAM200B	
ENST00000508567	ENSG00000237765	FAM200B	
ENST00000503600	ENSG00000237765	FAM200B	
ENST00000502856	ENSG00000237765	FAM200B	
ENST00000510186	ENSG00000237765	FAM200B	
ENST00000506610	ENSG00000237765	FAM200B	
ENST00000622362	ENSG00000237765	FAM200B	
ENST00000399001	ENSG00000157538	DSCR3	
ENST00000171887	ENSG00000079308	TNS1	filled
ENST00000446903	ENSG00000079308	TNS1	filled
ENST00000413280	ENSG00000079308	TNS1	filled
ENST00000423413	ENSG00000079308	TNS1	filled
ENST00000449814	ENSG00000079308	TNS1	filled
ENST00000611415	ENSG00000079308	TNS1	filled
ENST00000615025	ENSG00000079308	TNS1	filled
ENST00000519479	ENSG00000253953	PCDHGB4	filled
ENST00000496459	ENSG00000147274	RBMX	
ENST00000419968	ENSG00000147274	RBMX	
ENST00000536401	ENSG00000133316	WDR74	
ENST00000607124	ENSG00000124635	HIST1H2BJ	filled
ENST00000369153	ENSG00000178096	BOLA1	
ENST00000621067	ENSG00000276618	RAD17	
ENST00000569547	ENSG00000273025	CELF6	filled
ENST00000547654	ENSG00000139218	SCAF11	
ENST00000550629	ENSG00000139218	SCAF11	
ENST00000464149	ENSG00000013619	MAMLD1	filled
ENST00000599263	ENSG00000105219	CNTD2	
ENST00000593335	ENSG00000105219	CNTD2	
ENST00000491143	ENSG00000119547	ONECUT2	filled
ENST00000522532	ENSG00000147684	NDUFB9	
ENST00000517830	ENSG00000147684	NDUFB9	
ENST00000494105	ENSG00000163424	C3orf30	
ENST00000378711	ENSG00000205086	C2orf91	filled
ENST00000615044	ENSG00000205086	C2orf91	
ENST00000603218	ENSG00000270379	HEATR9	
ENST00000604376	ENSG00000270379	HEATR9	
ENST00000269383	ENSG00000141569	TRIM65	
ENST00000543309	ENSG00000141569	TRIM65	
ENST00000540128	ENSG00000141569	TRIM65	
ENST00000540812	ENSG00000141569	TRIM65	
ENST00000409262	ENSG00000187605	TET3	filled
ENST00000496886	ENSG00000187605	TET3	filled
ENST00000305799	ENSG00000187605	TET3	filled
ENST00000476808	ENSG00000114796	KLHL24	filled
ENST00000493949	ENSG00000107438	PDLIM1	filled
ENST00000558231	ENSG00000128918	ALDH1A2	filled
ENST00000557967	ENSG00000128918	ALDH1A2	filled
ENST00000493385	ENSG00000119929	CUTC	filled
ENST00000261425	ENSG00000109790	KLHL5	filled
ENST00000254941	ENSG00000132603	NIP7	
ENST00000567202	ENSG00000132603	NIP7	
ENST00000562523	ENSG00000132603	NIP7	

ENST00000396166	ENSG00000110786	PTPN5	
ENST00000503687	ENSG00000243335	KCTD7	filled
ENST00000598904	ENSG00000187994	RINL	
ENST00000589111	ENSG00000187994	RINL	
ENST00000588674	ENSG00000189001	SBSN	filled
ENST00000518157	ENSG00000189001	SBSN	filled
ENST00000380701	ENSG00000164989	CCDC171	filled
ENST00000449575	ENSG00000164989	CCDC171	filled
ENST00000432954	ENSG00000164989	CCDC171	filled
ENST00000486641	ENSG00000164989	CCDC171	filled
ENST00000478913	ENSG00000164989	CCDC171	
ENST00000325089	ENSG00000165300	SLITRK5	
ENST00000471707	ENSG00000183597	TANGO2	
ENST00000401886	ENSG00000183597	TANGO2	
ENST00000432198	ENSG00000183597	TANGO2	
ENST00000411907	ENSG00000183597	TANGO2	
ENST00000450019	ENSG00000183597	TANGO2	
ENST00000546361	ENSG00000085872	CHERP	filled
ENST00000198939	ENSG00000085872	CHERP	filled
ENST00000544299	ENSG00000085872	CHERP	filled
ENST00000261558	ENSG00000053770	AP5M1	filled
ENST00000556377	ENSG00000053770	AP5M1	
ENST00000592904	ENSG00000174652	ZNF266	filled
ENST00000436518	ENSG00000099904	ZDHHC8	
ENST00000460936	ENSG00000165028	NIPSNAP3B	
ENST00000374762	ENSG00000165028	NIPSNAP3B	
ENST00000461177	ENSG00000165028	NIPSNAP3B	
ENST00000402886	ENSG00000007866	TEAD3	filled
ENST00000338863	ENSG00000007866	TEAD3	filled
ENST00000340990	ENSG00000159346	ADIPOR1	
ENST00000417068	ENSG00000159346	ADIPOR1	
ENST00000426229	ENSG00000159346	ADIPOR1	
ENST00000367254	ENSG00000159346	ADIPOR1	
ENST00000434923	ENSG00000087495	PHACTR3	
ENST00000492611	ENSG00000087495	PHACTR3	
ENST00000473657	ENSG00000087495	PHACTR3	
ENST00000330910	ENSG00000185662	SMIM23	filled
ENST00000370244	ENSG00000137936	BCAR3	filled
ENST00000509702	ENSG00000109819	PPARGC1A	filled
ENST00000514494	ENSG00000109819	PPARGC1A	
ENST00000318003	ENSG00000132024	CC2D1A	
ENST00000585896	ENSG00000132024	CC2D1A	
ENST00000589606	ENSG00000132024	CC2D1A	
ENST00000587508	ENSG00000132024	CC2D1A	
ENST00000570163	ENSG00000166669	ATF7IP2	
ENST00000457313	ENSG00000135912	TTLL4	filled
ENST00000415717	ENSG00000135912	TTLL4	
ENST00000392102	ENSG00000135912	TTLL4	filled
ENST00000437755	ENSG00000135912	TTLL4	
ENST00000442769	ENSG00000135912	TTLL4	filled
ENST00000225899	ENSG00000108759	KRT32	
ENST00000413714	ENSG00000187616	TMEM8C	filled
ENST00000612775	ENSG00000213588	ZBTB9	
ENST00000449846	ENSG00000239605	C2orf61	
ENST00000445927	ENSG00000239605	C2orf61	
ENST00000619481	ENSG00000009413	REV3L	filled
ENST00000558232	ENSG00000137824	RMDN3	
ENST00000558560	ENSG00000137824	RMDN3	
ENST00000373659	ENSG00000186130	ZBTB6	
ENST00000396073	ENSG00000132464	ENAM	filled

ENST00000528572	ENSG00000187479	C11orf96	
ENST00000339446	ENSG00000187479	C11orf96	
ENST00000373313	ENSG00000204103	MAFB	
ENST00000608091	ENSG00000134851	TMEM165	
ENST00000506103	ENSG00000134851	TMEM165	
ENST00000309180	ENSG00000174946	GPR171	
ENST00000480322	ENSG00000174946	GPR171	
ENST00000490821	ENSG00000116127	ALMS1	
ENST00000409035	ENSG00000141979	CTD-3222D19.2	filled
ENST00000520407	ENSG00000157168	NRG1	filled
ENST00000614767	ENSG00000157168	NRG1	
ENST00000530365	ENSG00000137642	SORL1	
ENST00000625080	ENSG00000176659	C20orf197	
ENST00000313426	ENSG00000176659	C20orf197	
ENST00000526029	ENSG00000129159	KCNC1	
ENST00000531188	ENSG00000149273	RPS3	
ENST00000527446	ENSG00000149273	RPS3	
ENST00000494912	ENSG00000102125	TAZ	
ENST00000498029	ENSG00000102125	TAZ	
ENST00000301364	ENSG00000167721	TSR1	filled
ENST00000464257	ENSG00000206489	PPP1R10	
ENST00000563634	ENSG00000260371	RP11-343C2.9	
ENST00000437535	ENSG00000146809	ASB15	
ENST00000477916	ENSG00000197880	MDS2	filled
ENST00000497864	ENSG00000248487	ABHD14A	
ENST00000494478	ENSG00000248487	ABHD14A	
ENST00000464747	ENSG00000116044	NFE2L2	filled
ENST00000586532	ENSG00000116044	NFE2L2	filled
ENST00000588123	ENSG00000116044	NFE2L2	filled
ENST00000624515	ENSG00000143624	INTS3	
ENST00000601460	ENSG00000239665	RP11-295P9.3	
ENST00000419851	ENSG00000239665	RP11-295P9.3	
ENST00000518864	ENSG00000147481	SNTG1	filled
ENST00000522124	ENSG00000147481	SNTG1	filled
ENST00000517473	ENSG00000147481	SNTG1	filled
ENST00000520825	ENSG00000147481	SNTG1	filled
ENST00000486416	ENSG00000155363	MOV10	
ENST00000489791	ENSG00000152580	IGSF10	filled
ENST00000497472	ENSG00000152580	IGSF10	
ENST00000380079	ENSG00000127954	STEAP4	
ENST00000518261	ENSG00000206579	XKR4	
ENST00000431316	ENSG00000133742	CA1	
ENST00000370606	ENSG00000156925	ZIC3	filled
ENST00000444572	ENSG00000170959	DCDC1	filled
ENST00000483396	ENSG00000170959	DCDC1	filled
ENST00000597505	ENSG00000170959	DCDC1	filled
ENST00000437348	ENSG00000170959	DCDC1	filled
ENST00000342355	ENSG00000170959	DCDC1	filled
ENST00000534722	ENSG00000170959	DCDC1	filled
ENST00000452803	ENSG00000170959	DCDC1	filled
ENST00000401815	ENSG00000178732	GP5	
ENST00000464967	ENSG00000198910	L1CAM	filled
ENST00000534049	ENSG00000150093	ITGB1	
ENST00000475184	ENSG00000150093	ITGB1	
ENST00000221315	ENSG00000256087	ZNF432	filled
ENST00000540654	ENSG00000197901	SLC22A6	
ENST00000446716	ENSG00000198925	ATG9A	filled
ENST00000555076	ENSG00000170633	RNF34	
ENST00000564310	ENSG00000104164	BLOC1S6	
ENST00000492602	ENSG00000157020	SEC13	filled

ENST00000358077	ENSG00000196730	DAPK1	filled
ENST00000472284	ENSG00000196730	DAPK1	filled
ENST00000469067	ENSG00000196730	DAPK1	filled
ENST00000408954	ENSG00000196730	DAPK1	filled
ENST00000491893	ENSG00000196730	DAPK1	filled
ENST00000489291	ENSG00000196730	DAPK1	filled
ENST00000622514	ENSG00000196730	DAPK1	filled
ENST00000469640	ENSG00000196730	DAPK1	filled
ENST00000470521	ENSG00000154305	MIA3	
ENST00000435056	ENSG00000242092	HLA-DMB	filled
ENST00000525810	ENSG00000158813	EDA	filled
ENST00000527388	ENSG00000158813	EDA	filled
ENST00000533317	ENSG00000158813	EDA	filled
ENST00000374548	ENSG00000158813	EDA	filled
ENST00000502251	ENSG00000158813	EDA	filled
ENST00000374553	ENSG00000158813	EDA	filled
ENST00000374552	ENSG00000158813	EDA	filled
ENST00000524573	ENSG00000158813	EDA	filled
ENST00000503592	ENSG00000158813	EDA	filled
ENST00000250863	ENSG00000092345	DAZL	filled
ENST00000399444	ENSG00000092345	DAZL	filled
ENST00000464330	ENSG00000265264	TIMM10B	
ENST00000472836	ENSG00000265264	TIMM10B	
ENST00000597961	ENSG00000269590	CTD-2192J16.24	
ENST00000575594	ENSG00000153930	ANKFN1	filled
ENST00000462155	ENSG00000006652	IFRD1	
ENST00000623481	ENSG00000176087	SLC35A4	
ENST00000471373	ENSG00000013297	CLDN11	
ENST00000477531	ENSG00000013297	CLDN11	
ENST00000488989	ENSG00000013297	CLDN11	
ENST00000579022	ENSG00000206026	SMIM21	
ENST00000382638	ENSG00000206026	SMIM21	
ENST00000584508	ENSG00000206026	SMIM21	
ENST00000409178	ENSG00000222047	C10orf55	
ENST00000376947	ENSG00000206512	OR2H2	
ENST00000479510	ENSG00000197283	SYNGAP1	filled
ENST00000449372	ENSG00000197283	SYNGAP1	filled
ENST00000293748	ENSG00000197283	SYNGAP1	filled
ENST00000486399	ENSG00000197283	SYNGAP1	
ENST00000585482	ENSG00000004139	SARM1	filled
ENST00000402849	ENSG00000100028	SNRPD3	filled
ENST00000361803	ENSG00000144057	ST6GAL2	
ENST00000482640	ENSG00000079257	LXN	
ENST00000353411	ENSG00000113558	SKP1	
ENST00000468913	ENSG00000164008	C1orf50	filled
ENST00000564808	ENSG00000261427	CTD-2349B8.1	
ENST00000568526	ENSG00000261427	CTD-2349B8.1	
ENST00000439040	ENSG00000133657	ATP13A3	filled
ENST00000457986	ENSG00000133657	ATP13A3	filled
ENST00000446356	ENSG00000133657	ATP13A3	
ENST00000369575	ENSG00000160219	GAB3	
ENST00000369568	ENSG00000160219	GAB3	
ENST00000424127	ENSG00000160219	GAB3	
ENST00000284287	ENSG00000196119	OR8A1	
ENST00000431807	ENSG00000144395	CCDC150	filled
ENST00000498512	ENSG00000144395	CCDC150	
ENST00000497159	ENSG00000144395	CCDC150	
ENST00000461243	ENSG00000144395	CCDC150	
ENST00000533490	ENSG00000189167	ZAR1L	
ENST00000430238	ENSG00000235754	APOM	

ENST00000425177	ENSG00000235754	APOM	
ENST00000344642	ENSG00000187942	LDLRAD2	
ENST00000484271	ENSG00000187942	LDLRAD2	
ENST00000456666	ENSG00000230624	DDX39B	filled
ENST00000394670	ENSG00000010244	ZNF207	
ENST00000577908	ENSG00000010244	ZNF207	
ENST00000581531	ENSG00000010244	ZNF207	
ENST00000579810	ENSG00000010244	ZNF207	
ENST00000584416	ENSG00000010244	ZNF207	
ENST00000491607	ENSG00000168273	SMIM4	
ENST00000477703	ENSG00000168273	SMIM4	
ENST00000476842	ENSG00000168273	SMIM4	filled
ENST00000552854	ENSG00000139354	GAS2L3	
ENST00000455860	ENSG00000227046	DAXX	
ENST00000436311	ENSG00000227046	DAXX	
ENST00000486470	ENSG00000227046	DAXX	
ENST00000472399	ENSG00000227046	DAXX	
ENST00000487360	ENSG00000227046	DAXX	
ENST00000440500	ENSG00000227046	DAXX	
ENST00000419855	ENSG00000227046	DAXX	filled
ENST00000491696	ENSG00000227046	DAXX	
ENST00000421874	ENSG00000227046	DAXX	
ENST00000480469	ENSG00000227046	DAXX	
ENST00000612868	ENSG00000227046	DAXX	
ENST00000616312	ENSG00000227046	DAXX	
ENST00000263119	ENSG00000099991	CABIN1	filled
ENST00000405822	ENSG00000099991	CABIN1	filled
ENST00000445422	ENSG00000099991	CABIN1	filled
ENST00000398319	ENSG00000099991	CABIN1	filled
ENST00000617531	ENSG00000099991	CABIN1	filled
ENST00000493917	ENSG00000107262	BAG1	filled
ENST00000531590	ENSG00000173621	LRFN4	
ENST00000393952	ENSG00000173621	LRFN4	
ENST00000556884	ENSG00000119698	PPP4R4	filled
ENST00000493878	ENSG00000157259	GATAD1	filled
ENST00000377049	ENSG00000241563	CORT	
ENST00000216268	ENSG00000100426	ZBED4	
ENST00000401642	ENSG00000184178	SCFD2	filled
ENST00000388940	ENSG00000184178	SCFD2	filled
ENST00000520806	ENSG00000147647	DPYS	filled
ENST00000533874	ENSG00000147647	DPYS	filled
ENST00000520483	ENSG00000147647	DPYS	filled
ENST00000521372	ENSG00000147647	DPYS	filled
ENST00000342213	ENSG00000176435	CLEC14A	
ENST00000542348	ENSG00000119242	CCDC92	
ENST00000544798	ENSG00000119242	CCDC92	
ENST00000539761	ENSG00000119242	CCDC92	
ENST00000438666	ENSG00000133104	SPG20	
ENST00000494062	ENSG00000133104	SPG20	
ENST00000355182	ENSG00000133104	SPG20	filled
ENST00000451493	ENSG00000133104	SPG20	
ENST00000443157	ENSG00000131355	EMR3	filled
ENST00000344373	ENSG00000131355	EMR3	filled
ENST00000422747	ENSG00000236843	NRM	
ENST00000366645	ENSG00000116903	EXOC8	
ENST00000370271	ENSG00000119906	FAM178A	filled
ENST00000406337	ENSG00000083168	KAT6A	filled
ENST00000426524	ENSG00000083168	KAT6A	filled
ENST00000396930	ENSG00000083168	KAT6A	filled
ENST00000454829	ENSG00000232143	GNL1	

ENST00000576064	ENSG00000262845	KRT40	
ENST00000576967	ENSG00000262845	KRT40	
ENST00000533755	ENSG00000179636	TPPP2	
ENST00000534434	ENSG00000179636	TPPP2	
ENST00000472458	ENSG00000179636	TPPP2	
ENST00000481535	ENSG00000179636	TPPP2	
ENST00000529496	ENSG00000179636	TPPP2	
ENST00000495665	ENSG00000179636	TPPP2	
ENST00000555751	ENSG00000179636	TPPP2	
ENST00000537057	ENSG00000111725	PRKAB1	
ENST00000497933	ENSG00000183908	LRRC55	filled
ENST00000546931	ENSG00000170627	GTSF1	filled
ENST00000552397	ENSG00000170627	GTSF1	filled
ENST00000305879	ENSG00000170627	GTSF1	
ENST00000552395	ENSG00000170627	GTSF1	filled
ENST00000552336	ENSG00000170627	GTSF1	
ENST00000546575	ENSG00000170627	GTSF1	
ENST00000548538	ENSG00000170627	GTSF1	filled
ENST00000370192	ENSG00000188641	DPYD	filled
ENST00000468208	ENSG00000168137	SETD5	
ENST00000399686	ENSG00000168137	SETD5	filled
ENST00000591998	ENSG00000196110	ZNF699	
ENST00000548712	ENSG00000111647	UHRF1BP1L	
ENST00000524974	ENSG00000178104	PDE4DIP	filled
ENST00000585156	ENSG00000178104	PDE4DIP	filled
ENST00000618462	ENSG00000178104	PDE4DIP	filled
ENST00000617031	ENSG00000178104	PDE4DIP	filled
ENST00000617527	ENSG00000178104	PDE4DIP	filled
ENST00000617248	ENSG00000178104	PDE4DIP	
ENST00000610852	ENSG00000178104	PDE4DIP	
ENST00000533259	ENSG00000178104	PDE4DIP	
ENST00000619981	ENSG00000178104	PDE4DIP	
ENST00000618504	ENSG00000178104	PDE4DIP	
ENST00000617454	ENSG00000178104	PDE4DIP	filled
ENST00000613995	ENSG00000178104	PDE4DIP	
ENST00000621371	ENSG00000178104	PDE4DIP	filled
ENST00000483528	ENSG00000001460	STPG1	
ENST00000475760	ENSG00000001460	STPG1	
ENST00000585993	ENSG00000176695	OR4F17	
ENST00000418543	ENSG00000123154	WDR83	
ENST00000546754	ENSG00000123154	WDR83	
ENST00000548381	ENSG00000123154	WDR83	
ENST00000553179	ENSG00000123154	WDR83	
ENST00000520490	ENSG00000008988	RPS20	
ENST00000482296	ENSG00000242038	EGFL8	filled
ENST00000460675	ENSG00000188243	COMMD6	filled
ENST00000471682	ENSG00000188243	COMMD6	filled
ENST00000546983	ENSG00000136352	NKX2-1	
ENST00000431660	ENSG00000075142	SRI	filled
ENST00000568170	ENSG00000166548	TK2	
ENST00000520984	ENSG00000120963	ZNF706	filled
ENST00000518071	ENSG00000120963	ZNF706	filled
ENST00000468711	ENSG00000115944	COX7A2L	
ENST00000378669	ENSG00000115944	COX7A2L	
ENST00000349466	ENSG00000067842	ATP2B3	filled
ENST00000370186	ENSG00000067842	ATP2B3	filled
ENST00000496610	ENSG00000067842	ATP2B3	
ENST00000513615	ENSG00000151552	QDPR	filled
ENST00000466368	ENSG00000164889	SLC4A2	
ENST00000443320	ENSG00000225890	HLA-DQA1	filled

ENST00000451947	ENSG00000225890	HLA-DQA1	filled
ENST00000416403	ENSG00000225890	HLA-DQA1	filled
ENST00000498305	ENSG00000225890	HLA-DQA1	filled
ENST00000442680	ENSG00000225890	HLA-DQA1	filled
ENST00000468299	ENSG00000225890	HLA-DQA1	filled
ENST00000244565	ENSG00000124602	UNC5CL	
ENST00000375377	ENSG00000165757	KIAA1462	filled
ENST00000464386	ENSG00000165757	KIAA1462	
ENST00000465712	ENSG00000165757	KIAA1462	filled
ENST00000400457	ENSG00000099715	PCDH11Y	filled
ENST00000495855	ENSG00000230456	DDR1	
ENST00000409039	ENSG00000197653	DNAH10	filled
ENST00000614082	ENSG00000197653	DNAH10	
ENST00000556710	ENSG00000258465	DCAF8	filled
ENST00000485079	ENSG00000258465	DCAF8	filled
ENST00000378302	ENSG00000136144	RCBTB1	filled
ENST00000492232	ENSG00000181090	EHMT1	filled
ENST00000460486	ENSG00000181090	EHMT1	filled
ENST00000511884	ENSG00000169851	PCDH7	filled
ENST00000621961	ENSG00000169851	PCDH7	filled
ENST00000509759	ENSG00000169851	PCDH7	filled
ENST00000509925	ENSG00000169851	PCDH7	filled
ENST00000610830	ENSG00000169851	PCDH7	
ENST00000566735	ENSG00000205730	ITPRIPL2	
ENST00000381440	ENSG00000205730	ITPRIPL2	
ENST00000465252	ENSG00000182473	EXOC7	filled
ENST00000556666	ENSG00000042317	SPATA7	filled
ENST00000556406	ENSG00000042317	SPATA7	filled
ENST00000554802	ENSG00000042317	SPATA7	filled
ENST00000373257	ENSG00000132793	LPIN3	
ENST00000479391	ENSG00000182150	ERCC6L2	filled
ENST00000402838	ENSG00000182150	ERCC6L2	
ENST00000320486	ENSG00000182150	ERCC6L2	filled
ENST00000491418	ENSG00000187144	SPATA21	
ENST00000614739	ENSG00000152207	CYSLTR2	
ENST00000282018	ENSG00000152207	CYSLTR2	
ENST00000261537	ENSG00000101752	MIB1	filled
ENST00000559328	ENSG00000075413	MARK3	
ENST00000560603	ENSG00000075413	MARK3	
ENST00000555235	ENSG00000075413	MARK3	
ENST00000476386	ENSG00000010165	METTL13	filled
ENST00000504577	ENSG00000131183	SLC34A1	
ENST00000453424	ENSG00000165406	8-Mar	
ENST00000395769	ENSG00000165406	8-Mar	
ENST00000319836	ENSG00000165406	8-Mar	filled
ENST00000520132	ENSG00000086589	RBM22	
ENST00000518917	ENSG00000086589	RBM22	
ENST00000497785	ENSG00000114021	NIT2	filled
ENST00000383233	ENSG00000134490	TMEM241	filled
ENST00000477053	ENSG00000134490	TMEM241	filled
ENST00000473688	ENSG00000134490	TMEM241	filled
ENST00000259339	ENSG00000136816	TOR1B	
ENST00000381337	ENSG00000066654	THUMPD1	
ENST00000396083	ENSG00000066654	THUMPD1	
ENST00000288670	ENSG00000157827	FMNL2	filled
ENST00000420432	ENSG00000236353	PBX2	filled
ENST00000588420	ENSG00000125746	EML2	filled
ENST00000461188	ENSG00000125875	TBC1D20	
ENST00000547529	ENSG00000187109	NAP1L1	
ENST00000547479	ENSG00000187109	NAP1L1	

ENST00000299563	ENSG00000166439	RNF169	filled
ENST00000527301	ENSG00000166439	RNF169	
ENST00000419110	ENSG00000029993	HMGB3	
ENST00000370693	ENSG00000112208	BAG2	
ENST00000527521	ENSG00000150687	PRSS23	filled
ENST00000280258	ENSG00000150687	PRSS23	filled
ENST00000532234	ENSG00000150687	PRSS23	filled
ENST00000533902	ENSG00000150687	PRSS23	filled
ENST00000532572	ENSG00000150687	PRSS23	filled
ENST00000528769	ENSG00000150687	PRSS23	filled
ENST00000531521	ENSG00000150687	PRSS23	filled
ENST00000587880	ENSG00000126581	BECN1	filled
ENST00000612631	ENSG00000126581	BECN1	
ENST00000545521	ENSG00000103495	MAZ	
ENST00000563012	ENSG00000103495	MAZ	
ENST00000562594	ENSG00000103495	MAZ	
ENST00000330871	ENSG00000184557	SOCS3	
ENST00000609267	ENSG00000089091	DZANK1	filled
ENST00000591546	ENSG00000255819	KLRC4-KLRK1	filled
ENST00000521466	ENSG00000164587	RPS14	
ENST00000498406	ENSG00000184436	THAP7	
ENST00000361654	ENSG00000130779	CLIP1	filled
ENST00000537178	ENSG00000130779	CLIP1	filled
ENST00000537004	ENSG00000130779	CLIP1	filled
ENST00000535290	ENSG00000130779	CLIP1	filled
ENST00000539080	ENSG00000130779	CLIP1	filled
ENST00000620786	ENSG00000130779	CLIP1	filled
ENST00000358808	ENSG00000130779	CLIP1	filled
ENST00000449706	ENSG00000168955	TM4SF20	
ENST00000335397	ENSG00000186509	OR9Q1	
ENST00000612174	ENSG00000186509	OR9Q1	
ENST00000567270	ENSG00000178741	COX5A	
ENST00000367282	ENSG00000170075	GPR37L1	
ENST00000378078	ENSG00000107185	RGP1	
ENST00000317005	ENSG00000180346	TIGD2	
ENST00000543314	ENSG00000111261	MANSC1	filled
ENST00000334325	ENSG00000186451	SPATA12	
ENST00000470216	ENSG00000218739	CEBPZOS	
ENST00000533448	ENSG00000175868	CALCB	
ENST00000617975	ENSG00000160999	SH2B2	filled
ENST00000368192	ENSG00000117036	ETV3	
ENST00000490633	ENSG00000188186	LAMTOR4	
ENST00000530562	ENSG00000166435	XRRA1	filled
ENST00000560748	ENSG00000156381	ANKRD9	
ENST00000309822	ENSG00000147679	UTP23	
ENST00000521703	ENSG00000147679	UTP23	filled
ENST00000517814	ENSG00000147679	UTP23	
ENST00000521071	ENSG00000147679	UTP23	
ENST00000517820	ENSG00000147679	UTP23	filled
ENST00000520733	ENSG00000147679	UTP23	filled
ENST00000524128	ENSG00000147679	UTP23	filled
ENST00000371657	ENSG00000244687	UBE2V1	filled
ENST00000396059	ENSG00000244687	UBE2V1	filled
ENST00000371674	ENSG00000244687	UBE2V1	filled
ENST00000340309	ENSG00000244687	UBE2V1	filled
ENST00000415862	ENSG00000244687	UBE2V1	filled
ENST00000371677	ENSG00000244687	UBE2V1	filled
ENST00000483534	ENSG00000244687	UBE2V1	
ENST00000492371	ENSG00000244687	UBE2V1	
ENST00000490555	ENSG00000244687	UBE2V1	

ENST00000493090	ENSG00000244687	UBE2V1	
ENST00000467839	ENSG00000244687	UBE2V1	
ENST00000432266	ENSG00000244687	UBE2V1	
ENST00000470565	ENSG00000244687	UBE2V1	
ENST00000490289	ENSG00000244687	UBE2V1	
ENST00000461960	ENSG00000244687	UBE2V1	
ENST00000462217	ENSG00000244687	UBE2V1	
ENST00000473860	ENSG00000244687	UBE2V1	
ENST00000625172	ENSG00000244687	UBE2V1	filled
ENST00000617119	ENSG00000244687	UBE2V1	filled
ENST00000557021	ENSG00000244687	UBE2V1	filled
ENST00000420027	ENSG00000244687	UBE2V1	filled
ENST00000484180	ENSG00000241644	INMT	filled
ENST00000358935	ENSG00000198060	5-Mar	filled
ENST00000309979	ENSG00000166507	NDST2	filled
ENST00000465929	ENSG00000166507	NDST2	
ENST00000398701	ENSG00000166507	NDST2	filled
ENST00000302754	ENSG00000171223	JUNB	
ENST00000497020	ENSG00000105778	AVL9	
ENST00000377503	ENSG00000135083	CCNJL	
ENST00000438502	ENSG00000235773	NRM	
ENST00000490046	ENSG00000131069	ACSS2	filled
ENST00000494727	ENSG00000131069	ACSS2	
ENST00000331569	ENSG00000183779	ZNF703	
ENST00000322110	ENSG00000176925	OR51F2	
ENST00000570805	ENSG00000108561	C1QBP	
ENST00000372903	ENSG00000024048	UBR2	filled
ENST00000372899	ENSG00000024048	UBR2	filled
ENST00000372901	ENSG00000024048	UBR2	filled
ENST00000434700	ENSG00000197520	FAM177B	
ENST00000391880	ENSG00000197520	FAM177B	
ENST00000360827	ENSG00000197520	FAM177B	
ENST00000460763	ENSG00000197520	FAM177B	
ENST00000624272	ENSG00000224186	C5orf66	filled
ENST00000507641	ENSG00000224186	C5orf66	filled
ENST00000505828	ENSG00000224186	C5orf66	filled
ENST00000555438	ENSG00000224186	C5orf66	
ENST00000554670	ENSG00000224186	C5orf66	
ENST00000432382	ENSG00000224186	C5orf66	filled
ENST00000455611	ENSG00000164164	OTUD4	
ENST00000520747	ENSG00000197948	FCHSD1	
ENST00000523856	ENSG00000197948	FCHSD1	
ENST00000341502	ENSG00000150527	CTAGE5	filled
ENST00000579871	ENSG00000141034	GID4	
ENST00000379160	ENSG00000132646	PCNA	filled
ENST00000490786	ENSG00000162949	CAPN13	
ENST00000578794	ENSG00000266086	RP11-159D12.5	
ENST00000402989	ENSG00000163029	SMC6	filled
ENST00000451608	ENSG00000264324	RP11-287D1.3	filled
ENST00000583083	ENSG00000101670	LIPG	
ENST00000261292	ENSG00000101670	LIPG	filled
ENST00000623277	ENSG00000101670	LIPG	
ENST00000596239	ENSG00000105197	TIMM50	
ENST00000377428	ENSG00000132631	SCP2D1	
ENST00000555699	ENSG00000100600	LGMN	filled
ENST00000334869	ENSG00000100600	LGMN	filled
ENST00000557434	ENSG00000100600	LGMN	filled
ENST00000393218	ENSG00000100600	LGMN	filled
ENST00000557609	ENSG00000100600	LGMN	filled
ENST00000556097	ENSG00000100600	LGMN	filled

ENST00000554189	ENSG00000100600	LGMN	
ENST00000557694	ENSG00000100600	LGMN	
ENST00000554397	ENSG00000100600	LGMN	
ENST00000554919	ENSG00000100600	LGMN	
ENST00000553918	ENSG00000100600	LGMN	
ENST00000421812	ENSG00000143067	ZNF697	
ENST00000502564	ENSG00000145388	METTL14	
ENST00000531380	ENSG00000174804	FZD4	
ENST00000460995	ENSG00000197093	GAL3ST4	
ENST00000418521	ENSG00000124678	TCP11	
ENST00000551194	ENSG00000135406	PRPH	
ENST00000257860	ENSG00000135406	PRPH	filled
ENST00000532332	ENSG00000135406	PRPH	
ENST00000530631	ENSG00000135406	PRPH	
ENST00000221232	ENSG00000088038	CNOT3	filled
ENST00000618939	ENSG00000088038	CNOT3	filled
ENST00000564080	ENSG00000260170	RP11-96O20.4	filled
ENST00000464357	ENSG00000160285	LSS	
ENST00000450351	ENSG00000160285	LSS	
ENST00000457828	ENSG00000160285	LSS	
ENST00000584400	ENSG00000108604	SMARCD2	
ENST00000613943	ENSG00000108604	SMARCD2	
ENST00000506591	ENSG00000156463	SH3RF2	
ENST00000470814	ENSG00000184702	5-Sep	
ENST00000368586	ENSG00000171811	CFAP46	filled
ENST00000486104	ENSG00000171811	CFAP46	
ENST00000521746	ENSG00000169499	PLEKHA2	filled
ENST00000616927	ENSG00000169499	PLEKHA2	filled
ENST00000617275	ENSG00000169499	PLEKHA2	filled
ENST00000519640	ENSG00000169499	PLEKHA2	
ENST00000616834	ENSG00000169499	PLEKHA2	filled
ENST00000552924	ENSG00000167552	TUBA1A	filled
ENST00000550811	ENSG00000167552	TUBA1A	filled
ENST00000300850	ENSG00000167395	ZNF646	filled
ENST00000564189	ENSG00000167395	ZNF646	filled
ENST00000428260	ENSG00000167395	ZNF646	filled
ENST00000394979	ENSG00000167395	ZNF646	filled
ENST00000296161	ENSG00000163840	DTX3L	
ENST00000416322	ENSG00000157224	CLDN12	filled
ENST00000478752	ENSG00000157224	CLDN12	
ENST00000476475	ENSG00000157224	CLDN12	
ENST00000427944	ENSG00000228854	NRM	
ENST00000457590	ENSG00000104885	DOT1L	
ENST00000602349	ENSG00000122584	NXPH1	
ENST00000473703	ENSG00000176978	DPP7	
ENST00000509330	ENSG00000118482	PHF3	filled
ENST00000505138	ENSG00000118482	PHF3	filled
ENST00000414286	ENSG00000215183	MSMP	
ENST00000522787	ENSG00000154263	ABCA10	
ENST00000521538	ENSG00000154263	ABCA10	
ENST00000524231	ENSG00000154263	ABCA10	
ENST00000588514	ENSG00000154263	ABCA10	
ENST00000521526	ENSG00000154263	ABCA10	
ENST00000495670	ENSG00000186063	AIDA	
ENST00000538490	ENSG00000196666	FAM180B	
ENST00000326902	ENSG00000180613	GSX2	
ENST00000605275	ENSG00000213930	GALT	filled
ENST00000591922	ENSG00000101654	RNMT	
ENST00000493182	ENSG00000078098	FAP	
ENST00000485999	ENSG00000243189	HLA-DMA	

ENST00000494230	ENSG00000243189	HLA-DMA	
ENST00000465804	ENSG00000018408	WWTR1	filled
ENST00000471586	ENSG00000018408	WWTR1	
ENST00000494754	ENSG00000018408	WWTR1	
ENST00000472417	ENSG00000018408	WWTR1	filled
ENST00000485244	ENSG00000018408	WWTR1	
ENST00000396465	ENSG00000147604	RPL7	
ENST00000518933	ENSG00000132911	NMUR2	filled
ENST00000451097	ENSG00000155254	MARVELD1	
ENST00000434038	ENSG00000155254	MARVELD1	
ENST00000285605	ENSG00000155254	MARVELD1	
ENST00000497837	ENSG00000112493	TAPBP	
ENST00000550330	ENSG00000112493	TAPBP	
ENST00000359856	ENSG00000162704	ARPC5	filled
ENST00000367534	ENSG00000162704	ARPC5	filled
ENST00000562699	ENSG00000175938	ORAI3	
ENST00000371568	ENSG00000085840	ORC1	
ENST00000371566	ENSG00000085840	ORC1	
ENST00000548806	ENSG00000151135	TMEM263	
ENST00000551237	ENSG00000151135	TMEM263	
ENST00000289953	ENSG00000158901	WFDC8	filled
ENST00000486293	ENSG00000170417	TMEM182	filled
ENST00000515137	ENSG00000085871	MGST2	filled
ENST00000616265	ENSG00000085871	MGST2	filled
ENST00000348370	ENSG00000157450	RNF111	
ENST00000557998	ENSG00000157450	RNF111	
ENST00000560080	ENSG00000157450	RNF111	
ENST00000497385	ENSG00000134247	PTGFRN	
ENST00000380232	ENSG00000171855	IFNB1	
ENST00000302517	ENSG00000171604	CXXC5	filled
ENST00000502336	ENSG00000171604	CXXC5	filled
ENST00000520967	ENSG00000171604	CXXC5	filled
ENST00000511048	ENSG00000171604	CXXC5	filled
ENST00000512816	ENSG00000171604	CXXC5	filled
ENST00000509238	ENSG00000171604	CXXC5	filled
ENST00000502716	ENSG00000171604	CXXC5	
ENST00000503511	ENSG00000171604	CXXC5	
ENST00000511457	ENSG00000171604	CXXC5	
ENST00000511591	ENSG00000171604	CXXC5	
ENST00000335798	ENSG00000182247	UBE2E2	filled
ENST00000396703	ENSG00000182247	UBE2E2	filled
ENST00000613545	ENSG00000182247	UBE2E2	filled
ENST00000430474	ENSG00000176956	LY6H	
ENST00000479685	ENSG00000176956	LY6H	
ENST00000342752	ENSG00000176956	LY6H	
ENST00000414417	ENSG00000176956	LY6H	
ENST00000615409	ENSG00000176956	LY6H	
ENST00000554329	ENSG00000232070	TMEM253	
ENST00000554844	ENSG00000232070	TMEM253	
ENST00000556585	ENSG00000232070	TMEM253	
ENST00000555314	ENSG00000232070	TMEM253	
ENST00000553744	ENSG00000232070	TMEM253	
ENST00000418511	ENSG00000232070	TMEM253	
ENST00000335040	ENSG00000186943	OR13C8	
ENST00000389328	ENSG00000167632	TRAPPC9	filled
ENST00000520857	ENSG00000167632	TRAPPC9	filled
ENST00000438773	ENSG00000167632	TRAPPC9	filled
ENST00000521667	ENSG00000167632	TRAPPC9	filled
ENST00000522504	ENSG00000167632	TRAPPC9	filled
ENST00000524162	ENSG00000167632	TRAPPC9	filled

ENST00000523777	ENSG00000167632	TRAPPC9	filled
ENST00000517667	ENSG00000167632	TRAPPC9	filled
ENST00000520532	ENSG00000167632	TRAPPC9	filled
ENST00000452104	ENSG00000115825	PRKD3	
ENST00000443977	ENSG00000115825	PRKD3	
ENST00000556275	ENSG00000140009	ESR2	filled
ENST00000604011	ENSG00000249884	RNF103-CHMP3	filled
ENST00000440757	ENSG00000249884	RNF103-CHMP3	filled
ENST00000503906	ENSG00000271793	RP11-321N4.5	filled
ENST00000533507	ENSG00000213619	NDUFS3	
ENST00000585268	ENSG00000176390	CRLF3	
ENST00000561153	ENSG00000137819	PAQR5	filled
ENST00000515588	ENSG00000205572	SERF1B	
ENST00000282588	ENSG00000213949	ITGA1	filled
ENST00000504086	ENSG00000213949	ITGA1	
ENST00000623635	ENSG00000139304	PTPRQ	
ENST00000551042	ENSG00000139304	PTPRQ	filled
ENST00000547376	ENSG00000139304	PTPRQ	
ENST00000551573	ENSG00000139304	PTPRQ	
ENST00000614701	ENSG00000139304	PTPRQ	filled
ENST00000547485	ENSG00000139304	PTPRQ	filled
ENST00000551624	ENSG00000139304	PTPRQ	
ENST00000547881	ENSG00000139304	PTPRQ	filled
ENST00000549355	ENSG00000139304	PTPRQ	filled
ENST00000616559	ENSG00000139304	PTPRQ	filled
ENST00000377167	ENSG00000204700	OR2J2	
ENST00000377865	ENSG00000184226	PCDH9	filled
ENST00000544246	ENSG00000184226	PCDH9	filled
ENST00000456367	ENSG00000184226	PCDH9	filled
ENST00000617020	ENSG00000184226	PCDH9	
ENST00000614931	ENSG00000184226	PCDH9	filled
ENST00000377861	ENSG00000184226	PCDH9	
ENST00000371471	ENSG00000171940	ZNF217	
ENST00000431687	ENSG00000171940	ZNF217	
ENST00000540425	ENSG00000171940	ZNF217	
ENST00000427329	ENSG00000070950	RAD18	filled
ENST00000585927	ENSG00000133265	HSPBP1	
ENST00000505280	ENSG00000150961	SEC24D	
ENST00000522917	ENSG00000214814	FER1L6	filled
ENST00000400147	ENSG00000170579	DLGAP1	filled
ENST00000400149	ENSG00000170579	DLGAP1	filled
ENST00000400155	ENSG00000170579	DLGAP1	filled
ENST00000400150	ENSG00000170579	DLGAP1	filled
ENST00000315677	ENSG00000170579	DLGAP1	filled
ENST00000534970	ENSG00000170579	DLGAP1	filled
ENST00000539435	ENSG00000170579	DLGAP1	filled
ENST00000581699	ENSG00000170579	DLGAP1	filled
ENST00000400145	ENSG00000170579	DLGAP1	filled
ENST00000581527	ENSG00000170579	DLGAP1	filled
ENST00000498188	ENSG00000170579	DLGAP1	filled
ENST00000478161	ENSG00000170579	DLGAP1	filled
ENST00000484845	ENSG00000170579	DLGAP1	
ENST00000582051	ENSG00000170579	DLGAP1	filled
ENST00000578250	ENSG00000170579	DLGAP1	filled
ENST00000577430	ENSG00000170579	DLGAP1	filled
ENST00000584874	ENSG00000170579	DLGAP1	filled
ENST00000515196	ENSG00000170579	DLGAP1	filled
ENST00000435978	ENSG00000231631	PSMB8	filled
ENST00000442394	ENSG00000197448	GSTK1	filled
ENST00000465879	ENSG00000234078	DDR1	

ENST00000526744	ENSG00000156097	GPR61	
ENST00000307221	ENSG00000172404	DNAJB7	filled
ENST00000416172	ENSG00000180357	ZNF609	filled
ENST00000509647	ENSG00000083857	FAT1	filled
ENST00000423059	ENSG00000005108	THSD7A	filled
ENST00000617773	ENSG00000005108	THSD7A	filled
ENST00000295417	ENSG00000163251	FZD5	
ENST00000509842	ENSG00000177311	ZBTB38	filled
ENST00000513619	ENSG00000177311	ZBTB38	filled
ENST00000512276	ENSG00000177311	ZBTB38	filled
ENST00000507657	ENSG00000177311	ZBTB38	filled
ENST00000507722	ENSG00000177311	ZBTB38	filled
ENST00000512327	ENSG00000177311	ZBTB38	
ENST00000513249	ENSG00000177311	ZBTB38	
ENST00000503809	ENSG00000177311	ZBTB38	filled
ENST00000513258	ENSG00000177311	ZBTB38	filled
ENST00000506623	ENSG00000177311	ZBTB38	
ENST00000509883	ENSG00000177311	ZBTB38	filled
ENST00000514251	ENSG00000177311	ZBTB38	filled
ENST00000510338	ENSG00000177311	ZBTB38	filled
ENST00000504673	ENSG00000177311	ZBTB38	filled
ENST00000513570	ENSG00000177311	ZBTB38	filled
ENST00000512769	ENSG00000177311	ZBTB38	
ENST00000533076	ENSG00000165915	SLC39A13	
ENST00000460103	ENSG00000244444	EGFL8	filled
ENST00000531706	ENSG00000133800	LYVE1	
ENST00000525294	ENSG00000137216	TMEM63B	
ENST00000439351	ENSG00000090520	DNAJB11	
ENST00000551553	ENSG00000139269	INHBE	
ENST00000547970	ENSG00000139269	INHBE	
ENST00000459937	ENSG00000122783	C7orf49	
ENST00000464070	ENSG00000122783	C7orf49	
ENST00000393114	ENSG00000122783	C7orf49	
ENST00000426145	ENSG00000099960	SLC7A4	
ENST00000476571	ENSG00000188580	NKAIN2	filled
ENST00000368416	ENSG00000188580	NKAIN2	filled
ENST00000368417	ENSG00000188580	NKAIN2	filled
ENST00000545433	ENSG00000188580	NKAIN2	filled
ENST00000511031	ENSG00000168685	IL7R	
ENST00000505019	ENSG00000138658	ZGRF1	filled
ENST00000473015	ENSG00000138658	ZGRF1	filled
ENST00000445203	ENSG00000138658	ZGRF1	filled
ENST00000502351	ENSG00000185129	PURA	
ENST00000331327	ENSG00000185129	PURA	filled
ENST00000555327	ENSG00000177108	ZDHHC22	filled
ENST00000413695	ENSG00000113838	TBCCD1	filled
ENST00000424280	ENSG00000113838	TBCCD1	filled
ENST00000361717	ENSG00000145365	TIFA	
ENST00000448427	ENSG00000243147	MRPL33	filled
ENST00000507680	ENSG00000167083	GNGT2	
ENST00000543245	ENSG00000072778	ACADVL	
ENST00000326639	ENSG00000138709	LARP1B	filled
ENST00000508819	ENSG00000138709	LARP1B	filled
ENST00000287022	ENSG00000156467	UQCRB	
ENST00000446546	ENSG00000214655	ZSWIM8	
ENST00000395045	ENSG00000007264	MATK	filled
ENST00000590849	ENSG00000007264	MATK	filled
ENST00000620026	ENSG00000130338	TULP4	
ENST00000600383	ENSG00000124440	HIF3A	filled
ENST00000588898	ENSG00000170832	USP32	

ENST00000608855	ENSG00000163281	GNPDA2	filled
ENST00000609092	ENSG00000163281	GNPDA2	filled
ENST00000552259	ENSG00000139117	CPNE8	
ENST00000469783	ENSG00000163975	MFI2	filled
ENST00000556816	ENSG00000165898	ISCA2	
ENST00000500337	ENSG00000151881	C5orf28	
ENST00000510130	ENSG00000151881	C5orf28	
ENST00000506860	ENSG00000151881	C5orf28	
ENST00000265304	ENSG00000106028	SSBP1	
ENST00000465582	ENSG00000106028	SSBP1	
ENST00000379349	ENSG00000123131	PRDX4	filled
ENST00000415947	ENSG00000116117	PARD3B	filled
ENST00000406610	ENSG00000116117	PARD3B	filled
ENST00000462231	ENSG00000116117	PARD3B	filled
ENST00000471958	ENSG00000116117	PARD3B	
ENST00000358768	ENSG00000116117	PARD3B	filled
ENST00000351153	ENSG00000116117	PARD3B	filled
ENST00000349953	ENSG00000116117	PARD3B	filled
ENST00000529419	ENSG00000189431	RASSF10	
ENST00000399537	ENSG00000182095	TNRC18	filled
ENST00000440081	ENSG00000182095	TNRC18	
ENST00000430969	ENSG00000182095	TNRC18	filled
ENST00000393792	ENSG00000168246	UBTD2	filled
ENST00000370904	ENSG00000147255	IGSF1	
ENST00000409122	ENSG00000180336	C17orf104	filled
ENST00000472403	ENSG00000180336	C17orf104	filled
ENST00000588805	ENSG00000180336	C17orf104	filled
ENST00000226218	ENSG00000109072	VTN	
ENST00000324768	ENSG00000175874	CREG2	filled
ENST00000370648	ENSG00000102239	BRS3	
ENST00000489711	ENSG00000163406	SLC15A2	filled
ENST00000464733	ENSG00000168765	GSTM4	
ENST00000461767	ENSG00000168765	GSTM4	
ENST00000336075	ENSG00000168765	GSTM4	
ENST00000389484	ENSG00000168702	LRP1B	filled
ENST00000434794	ENSG00000168702	LRP1B	filled
ENST00000486364	ENSG00000168702	LRP1B	
ENST00000439940	ENSG00000115561	CHMP3	filled
ENST00000491176	ENSG00000112539	C6orf118	
ENST00000435504	ENSG00000143970	ASXL2	filled
ENST00000336112	ENSG00000143970	ASXL2	filled
ENST00000466658	ENSG00000160746	ANO10	
ENST00000370557	ENSG00000101974	ATP11C	filled
ENST00000303130	ENSG00000170647	TMEM133	
ENST00000295755	ENSG00000163515	RETNLB	
ENST00000600972	ENSG00000130522	JUND	
ENST00000502471	ENSG00000031003	FAM13B	filled
ENST00000446660	ENSG00000085760	MTIF2	filled
ENST00000366137	ENSG00000085760	MTIF2	filled
ENST00000404297	ENSG00000085760	MTIF2	filled
ENST00000420637	ENSG00000085760	MTIF2	filled
ENST00000417363	ENSG00000085760	MTIF2	filled
ENST00000457812	ENSG00000173166	RAPH1	filled
ENST00000616246	ENSG00000148606	POLR3A	filled
ENST00000234195	ENSG00000115841	RMDN2	
ENST00000469469	ENSG00000115841	RMDN2	
ENST00000278317	ENSG00000130595	TNNT3	filled
ENST00000453458	ENSG00000130595	TNNT3	filled
ENST00000381589	ENSG00000130595	TNNT3	filled
ENST00000381579	ENSG00000130595	TNNT3	filled

ENST00000381563	ENSG00000130595	TNNT3	filled
ENST00000344578	ENSG00000130595	TNNT3	filled
ENST00000381558	ENSG00000130595	TNNT3	filled
ENST00000397301	ENSG00000130595	TNNT3	filled
ENST00000394071	ENSG00000156030	ELMSAN1	filled
ENST00000476562	ENSG00000156030	ELMSAN1	filled
ENST00000486739	ENSG00000156030	ELMSAN1	filled
ENST00000564399	ENSG00000132612	VPS4A	filled
ENST00000512784	ENSG00000119715	ESRRB	filled
ENST00000505752	ENSG00000119715	ESRRB	filled
ENST00000507951	ENSG00000119715	ESRRB	filled
ENST00000611036	ENSG00000119715	ESRRB	
ENST00000396838	ENSG00000235109	ZSCAN31	filled
ENST00000414429	ENSG00000235109	ZSCAN31	filled
ENST00000281830	ENSG00000152049	KCNE4	
ENST00000361033	ENSG00000198326	TMEM239	
ENST00000529246	ENSG00000197261	C6orf141	
ENST00000424426	ENSG00000197261	C6orf141	
ENST00000415078	ENSG00000197261	C6orf141	
ENST00000526429	ENSG00000197261	C6orf141	filled
ENST00000503196	ENSG00000118564	FBXL5	filled
ENST00000509314	ENSG00000118564	FBXL5	filled
ENST00000510802	ENSG00000118564	FBXL5	filled
ENST00000507899	ENSG00000118564	FBXL5	filled
ENST00000492902	ENSG00000059915	PSD	
ENST00000579671	ENSG00000160602	NEK8	
ENST00000584342	ENSG00000160602	NEK8	
ENST00000579060	ENSG00000160602	NEK8	
ENST00000482595	ENSG00000135945	REV1	filled
ENST00000465086	ENSG00000135945	REV1	
ENST00000485487	ENSG00000135945	REV1	
ENST00000438366	ENSG00000135945	REV1	
ENST00000477121	ENSG00000135945	REV1	
ENST00000336949	ENSG00000165175	MID1IP1	filled
ENST00000614558	ENSG00000165175	MID1IP1	filled
ENST00000400186	ENSG00000183117	CSMD1	filled
ENST00000602723	ENSG00000183117	CSMD1	filled
ENST00000520002	ENSG00000183117	CSMD1	filled
ENST00000602557	ENSG00000183117	CSMD1	filled
ENST00000537824	ENSG00000183117	CSMD1	filled
ENST00000551946	ENSG00000258311	RP11-644F5.10	filled
ENST00000550412	ENSG00000258311	RP11-644F5.10	filled
ENST00000257899	ENSG00000258311	RP11-644F5.10	filled
ENST00000549424	ENSG00000258311	RP11-644F5.10	filled
ENST00000361328	ENSG00000121903	ZSCAN20	filled
ENST00000373413	ENSG00000121903	ZSCAN20	
ENST00000480917	ENSG00000121903	ZSCAN20	
ENST00000468877	ENSG00000122787	AKR1D1	filled
ENST00000333209	ENSG00000185477	GPRIN3	
ENST00000574474	ENSG00000074755	ZZEF1	filled
ENST00000467933	ENSG00000196369	SRGAP2B	
ENST00000507166	ENSG00000145216	FIP1L1	filled
ENST00000369448	ENSG00000183508	FAM46C	
ENST00000348706	ENSG00000138777	PPA2	filled
ENST00000379096	ENSG00000076554	TPD52	filled
ENST00000518937	ENSG00000076554	TPD52	filled
ENST00000534355	ENSG00000211450	C11orf31	
ENST00000533321	ENSG00000211450	C11orf31	
ENST00000388857	ENSG00000211450	C11orf31	
ENST00000528798	ENSG00000211450	C11orf31	

ENST00000534386	ENSG00000211450	C11orf31	
ENST00000622257	ENSG00000211450	C11orf31	
ENST00000592018	ENSG00000130202	PVRL2	
ENST00000394457	ENSG00000173209	AHSA2	
ENST00000546407	ENSG00000001626	CFTR	
ENST00000600166	ENSG00000001626	CFTR	filled
ENST00000429014	ENSG00000001626	CFTR	
ENST00000610149	ENSG00000001626	CFTR	
ENST00000282111	ENSG00000152284	TCF7L1	
ENST00000494746	ENSG00000110680	CALCA	
ENST00000492580	ENSG00000154258	ABCA9	
ENST00000392499	ENSG00000213139	CRYGS	
ENST00000462989	ENSG00000047579	DTNBP1	filled
ENST00000370862	ENSG00000137252	HCRTR2	filled
ENST00000615358	ENSG00000137252	HCRTR2	filled
ENST00000503358	ENSG00000204661	C5orf60	
ENST00000537475	ENSG00000166535	A2ML1	
ENST00000550574	ENSG00000167553	TUBA1C	filled
ENST00000541364	ENSG00000167553	TUBA1C	filled
ENST00000549818	ENSG00000167553	TUBA1C	filled
ENST00000552448	ENSG00000167553	TUBA1C	filled
ENST00000552125	ENSG00000167553	TUBA1C	filled
ENST00000301072	ENSG00000167553	TUBA1C	
ENST00000548470	ENSG00000167553	TUBA1C	filled
ENST00000551422	ENSG00000157551	KCNJ15	filled
ENST00000534708	ENSG00000142973	CYP4B1	
ENST00000529715	ENSG00000142973	CYP4B1	
ENST00000371919	ENSG00000142973	CYP4B1	
ENST00000614163	ENSG00000142973	CYP4B1	
ENST00000453314	ENSG00000236177	HLA-DPA1	
ENST00000444867	ENSG00000153993	SEMA3D	filled
ENST00000488816	ENSG00000149346	SLX4IP	
ENST00000437419	ENSG00000164818	HEATR2	
ENST00000358299	ENSG00000163596	ICA1L	filled
ENST00000438804	ENSG00000163596	ICA1L	
ENST00000420558	ENSG00000163596	ICA1L	
ENST00000418208	ENSG00000163596	ICA1L	
ENST00000450143	ENSG00000163596	ICA1L	
ENST00000354959	ENSG00000198521	ZNF43	filled
ENST00000598381	ENSG00000198521	ZNF43	filled
ENST00000528029	ENSG00000187066	TMEM262	
ENST00000524632	ENSG00000187066	TMEM262	
ENST00000475380	ENSG00000137332	DDR1	
ENST00000534663	ENSG00000166349	RAG1	filled
ENST00000509479	ENSG00000196782	MAML3	filled
ENST00000502696	ENSG00000196782	MAML3	filled
ENST00000570108	ENSG00000166140	ZFYVE19	
ENST00000564258	ENSG00000166140	ZFYVE19	
ENST00000355341	ENSG00000166140	ZFYVE19	
ENST00000336455	ENSG00000166140	ZFYVE19	
ENST00000299173	ENSG00000166140	ZFYVE19	
ENST00000569057	ENSG00000166140	ZFYVE19	
ENST00000561768	ENSG00000166140	ZFYVE19	
ENST00000560078	ENSG00000166140	ZFYVE19	
ENST00000456772	ENSG00000236251	HSPA1L	
ENST00000423431	ENSG00000114902	SPCS1	
ENST00000531163	ENSG00000180900	SCRIB	
ENST00000361952	ENSG00000198783	ZNF830	
ENST00000578339	ENSG00000198783	ZNF830	
ENST00000484501	ENSG00000174564	IL20RB	

ENST00000236273	ENSG00000117614	SYF2	
ENST00000354361	ENSG00000117614	SYF2	
ENST00000330619	ENSG00000197016	ZNF470	
ENST00000267549	ENSG00000140030	GPR65	
ENST00000247879	ENSG00000127362	TAS2R3	
ENST00000509108	ENSG00000145439	CBR4	filled
ENST00000511593	ENSG00000130844	ZNF331	
ENST00000505949	ENSG00000130844	ZNF331	
ENST00000380393	ENSG00000132670	PTPRA	filled
ENST00000216877	ENSG00000132670	PTPRA	filled
ENST00000318266	ENSG00000132670	PTPRA	filled
ENST00000356147	ENSG00000132670	PTPRA	filled
ENST00000399903	ENSG00000132670	PTPRA	filled
ENST00000529943	ENSG00000197302	ZNF720	filled
ENST00000539915	ENSG00000197302	ZNF720	filled
ENST00000316491	ENSG00000197302	ZNF720	filled
ENST00000533488	ENSG00000197302	ZNF720	
ENST00000398696	ENSG00000197302	ZNF720	filled
ENST00000530881	ENSG00000197302	ZNF720	
ENST00000529515	ENSG00000197302	ZNF720	
ENST00000383564	ENSG00000183311	TUBB	
ENST00000400531	ENSG00000183311	TUBB	
ENST00000375579	ENSG00000188312	CENPP	filled
ENST00000520921	ENSG00000164879	CA3	filled
ENST00000367584	ENSG00000112419	PHACTR2	filled
ENST00000518118	ENSG00000120533	ENY2	
ENST00000540741	ENSG00000181036	FCRL6	
ENST00000536257	ENSG00000181036	FCRL6	
ENST00000321935	ENSG00000181036	FCRL6	
ENST00000541729	ENSG00000181036	FCRL6	
ENST00000339348	ENSG00000181036	FCRL6	
ENST00000392235	ENSG00000181036	FCRL6	
ENST00000368106	ENSG00000181036	FCRL6	
ENST00000588687	ENSG00000180329	CCDC43	filled
ENST00000361566	ENSG00000171345	KRT19	
ENST00000455635	ENSG00000171345	KRT19	
ENST00000533338	ENSG00000168172	HOOK3	filled
ENST00000467345	ENSG00000143324	XPR1	
ENST00000606389	ENSG00000115977	AAK1	
ENST00000623317	ENSG00000115977	AAK1	
ENST00000495239	ENSG00000115977	AAK1	
ENST00000471775	ENSG00000115977	AAK1	
ENST00000461002	ENSG00000115977	AAK1	
ENST00000492192	ENSG00000115977	AAK1	
ENST00000517298	ENSG00000185015	CA13	filled
ENST00000522631	ENSG00000185015	CA13	
ENST00000518392	ENSG00000185015	CA13	
ENST00000583114	ENSG00000108387	4-Sep	
ENST00000426861	ENSG00000108387	4-Sep	
ENST00000580796	ENSG00000108387	4-Sep	
ENST00000583273	ENSG00000108387	4-Sep	
ENST00000317268	ENSG00000108387	4-Sep	
ENST00000577440	ENSG00000108387	4-Sep	
ENST00000317256	ENSG00000108387	4-Sep	
ENST00000584488	ENSG00000108387	4-Sep	
ENST00000581615	ENSG00000108387	4-Sep	
ENST00000578131	ENSG00000108387	4-Sep	
ENST00000585170	ENSG00000108387	4-Sep	
ENST00000583291	ENSG00000108387	4-Sep	
ENST00000584789	ENSG00000108387	4-Sep	

ENST00000581921	ENSG00000108387	4-Sep	
ENST00000584528	ENSG00000108387	4-Sep	
ENST00000580791	ENSG00000108387	4-Sep	
ENST00000582248	ENSG00000108387	4-Sep	
ENST00000578747	ENSG00000108387	4-Sep	
ENST00000581607	ENSG00000108387	4-Sep	
ENST00000582976	ENSG00000108387	4-Sep	
ENST00000393086	ENSG00000108387	4-Sep	
ENST00000422330	ENSG00000144619	CNTN4	filled
ENST00000427741	ENSG00000144619	CNTN4	filled
ENST00000455083	ENSG00000144619	CNTN4	filled
ENST00000418658	ENSG00000144619	CNTN4	filled
ENST00000494222	ENSG00000107679	PLEKHA1	filled
ENST00000368989	ENSG00000107679	PLEKHA1	
ENST00000433307	ENSG00000107679	PLEKHA1	
ENST00000368988	ENSG00000107679	PLEKHA1	
ENST00000583696	ENSG00000141441	GAREM	filled
ENST00000523762	ENSG00000108852	MPP2	filled
ENST00000464761	ENSG00000138443	ABI2	
ENST00000527990	ENSG00000179532	DNHD1	
ENST00000534210	ENSG00000179532	DNHD1	filled
ENST00000254579	ENSG00000179532	DNHD1	
ENST00000455236	ENSG00000100101	RP1-37E16.12	filled
ENST00000437635	ENSG00000189325	C6orf222	filled
ENST00000445873	ENSG00000236311	TLX1NB	filled
ENST00000425505	ENSG00000236311	TLX1NB	filled
ENST00000349457	ENSG00000159618	GPR114	
ENST00000340339	ENSG00000159618	GPR114	
ENST00000419488	ENSG00000187391	MAGI2	filled
ENST00000354212	ENSG00000187391	MAGI2	filled
ENST00000522391	ENSG00000187391	MAGI2	filled
ENST00000571021	ENSG00000015532	XYLT2	
ENST00000493975	ENSG00000013810	TACC3	
ENST00000394266	ENSG00000103037	SETD6	
ENST00000463954	ENSG00000103037	SETD6	filled
ENST00000456860	ENSG00000023171	GRAMD1B	
ENST00000529750	ENSG00000023171	GRAMD1B	
ENST00000322282	ENSG00000023171	GRAMD1B	
ENST00000488524	ENSG00000115386	REG1A	
ENST00000233735	ENSG00000115386	REG1A	
ENST00000625134	ENSG00000274443	C8orf89	
ENST00000348931	ENSG00000186075	ZPBP2	
ENST00000583811	ENSG00000186075	ZPBP2	
ENST00000584588	ENSG00000186075	ZPBP2	
ENST00000377940	ENSG00000186075	ZPBP2	
ENST00000412160	ENSG00000179603	GRM8	
ENST00000274811	ENSG00000146083	RNF44	
ENST00000494767	ENSG00000172053	QARS	
ENST00000481537	ENSG00000178217	SH2D4B	
ENST00000601627	ENSG00000268797	CTC-490E21.12	filled
ENST00000597663	ENSG00000130517	PGPEP1	
ENST00000595552	ENSG00000130517	PGPEP1	
ENST00000430026	ENSG00000096996	IL12RB1	filled
ENST00000598019	ENSG00000096996	IL12RB1	filled
ENST00000594176	ENSG00000096996	IL12RB1	filled
ENST00000418930	ENSG00000133740	E2F5	
ENST00000416274	ENSG00000133740	E2F5	
ENST00000517476	ENSG00000133740	E2F5	
ENST00000521429	ENSG00000133740	E2F5	
ENST00000518234	ENSG00000133740	E2F5	

ENST00000520225	ENSG00000133740	E2F5	
ENST00000442544	ENSG00000187323	DCC	filled
ENST00000511581	ENSG00000205129	C4orf47	
ENST00000523339	ENSG00000181163	NPM1	
ENST00000397456	ENSG00000265818	EEF1E1-BLOC1S5	filled
ENST00000593657	ENSG00000197360	ZNF98	filled
ENST00000299138	ENSG00000069329	VPS35	
ENST00000568784	ENSG00000069329	VPS35	
ENST00000383508	ENSG00000215425	DDX39B	filled
ENST00000619734	ENSG00000277278	HERC2	
ENST00000614263	ENSG00000277278	HERC2	
ENST00000612410	ENSG00000277278	HERC2	
ENST00000460450	ENSG00000114999	TTL	
ENST00000375227	ENSG00000073350	LLGL2	
ENST00000578363	ENSG00000073350	LLGL2	
ENST00000579392	ENSG00000073350	LLGL2	
ENST00000577200	ENSG00000073350	LLGL2	
ENST00000578536	ENSG00000073350	LLGL2	
ENST00000580925	ENSG00000073350	LLGL2	
ENST00000581713	ENSG00000073350	LLGL2	
ENST00000583658	ENSG00000073350	LLGL2	
ENST00000562590	ENSG00000177548	RABEP2	filled
ENST00000566762	ENSG00000177548	RABEP2	filled
ENST00000292114	ENSG00000244045	TMEM199	
ENST00000503581	ENSG00000188107	EYS	filled
ENST00000370621	ENSG00000188107	EYS	filled
ENST00000393380	ENSG00000188107	EYS	filled
ENST00000342421	ENSG00000188107	EYS	filled
ENST00000489873	ENSG00000188107	EYS	filled
ENST00000471279	ENSG00000188107	EYS	filled
ENST00000370618	ENSG00000188107	EYS	filled
ENST00000370616	ENSG00000188107	EYS	filled
ENST00000533866	ENSG00000254415	SIGLEC14	
ENST00000564111	ENSG00000048471	SNX29	
ENST00000352896	ENSG00000111879	FAM184A	filled
ENST00000368475	ENSG00000111879	FAM184A	filled
ENST00000306318	ENSG00000168505	GBX2	
ENST00000592199	ENSG00000008441	NFIX	filled
ENST00000472571	ENSG00000182518	FAM104B	
ENST00000477847	ENSG00000182518	FAM104B	
ENST00000489298	ENSG00000182518	FAM104B	
ENST00000425133	ENSG00000182518	FAM104B	
ENST00000507240	ENSG00000117266	CDK18	filled
ENST00000463212	ENSG00000187961	KLHL17	
ENST00000519179	ENSG00000204344	STK19	filled
ENST00000518568	ENSG00000253598	SLC10A5	
ENST00000381655	ENSG00000132932	ATP8A2	filled
ENST00000470223	ENSG00000186501	TMEM222	
ENST00000369937	ENSG00000107874	CUEDC2	
ENST00000486762	ENSG00000107874	CUEDC2	
ENST00000477994	ENSG00000107874	CUEDC2	
ENST00000477087	ENSG00000111276	CDKN1B	
ENST00000532462	ENSG00000100504	PYGL	
ENST00000344887	ENSG00000129991	TNNI3	
ENST00000355520	ENSG00000079482	OPHN1	filled
ENST00000491714	ENSG00000079482	OPHN1	
ENST00000570713	ENSG00000261972	CES5A	
ENST00000573178	ENSG00000261972	CES5A	filled
ENST00000576682	ENSG00000261972	CES5A	
ENST00000572754	ENSG00000261972	CES5A	filled

ENST00000576219	ENSG00000261972	CES5A	
ENST00000573522	ENSG00000261972	CES5A	
ENST00000576322	ENSG00000261972	CES5A	filled
ENST00000262304	ENSG00000008710	PKD1	filled
ENST00000423118	ENSG00000008710	PKD1	filled
ENST00000487932	ENSG00000008710	PKD1	filled
ENST00000528395	ENSG00000254462	TMX2-CTNND1	filled
ENST00000515559	ENSG00000170464	DNAJC18	
ENST00000257572	ENSG00000135116	HRK	filled
ENST00000561907	ENSG00000183751	TBL3	
ENST00000370836	ENSG00000171004	HS6ST2	filled
ENST00000521489	ENSG00000171004	HS6ST2	filled
ENST00000370833	ENSG00000171004	HS6ST2	filled
ENST00000406696	ENSG00000171004	HS6ST2	filled
ENST00000396976	ENSG00000140265	ZSCAN29	
ENST00000487380	ENSG00000115657	ABCB6	filled
ENST00000485773	ENSG00000115657	ABCB6	filled
ENST00000443805	ENSG00000115657	ABCB6	filled
ENST00000367521	ENSG00000198756	COLGALT2	filled
ENST00000396946	ENSG00000198286	CARD11	filled
ENST00000540096	ENSG00000256825	CTD-2140B24.4	
ENST00000524717	ENSG00000184384	MAML2	filled
ENST00000355502	ENSG00000105650	PDE4C	filled
ENST00000596647	ENSG00000105650	PDE4C	
ENST00000437917	ENSG00000228581	GNL1	
ENST00000439630	ENSG00000243958	PSMB9	
ENST00000423563	ENSG00000243958	PSMB9	filled
ENST00000473497	ENSG00000091831	ESR1	
ENST00000519522	ENSG00000037241	RPL26L1	
ENST00000370251	ENSG00000063587	ZNF275	
ENST00000370249	ENSG00000063587	ZNF275	
ENST00000444315	ENSG00000011105	TSPAN9	filled
ENST00000539631	ENSG00000011105	TSPAN9	
ENST00000585656	ENSG00000267477	CTC-398G3.6	filled
ENST00000452278	ENSG00000106113	CRHR2	filled
ENST00000348438	ENSG00000106113	CRHR2	filled
ENST00000445981	ENSG00000106113	CRHR2	
ENST00000423776	ENSG00000106113	CRHR2	
ENST00000462882	ENSG00000106113	CRHR2	
ENST00000341843	ENSG00000106113	CRHR2	filled
ENST00000496728	ENSG00000166925	TSC22D4	filled
ENST00000573621	ENSG00000262730	RP11-1099M24.7	filled
ENST00000545770	ENSG00000236980	C3orf84	
ENST00000443990	ENSG00000236980	C3orf84	
ENST00000432035	ENSG00000236980	C3orf84	
ENST00000423583	ENSG00000204099	NEU4	
ENST00000467579	ENSG00000213366	GSTM2	
ENST00000430771	ENSG00000111642	CHD4	
ENST00000508235	ENSG00000063978	RNF4	filled
ENST00000486818	ENSG00000106635	BCL7B	
ENST00000223368	ENSG00000106635	BCL7B	filled
ENST00000411832	ENSG00000106635	BCL7B	filled
ENST00000479795	ENSG00000168477	TNXB	
ENST00000277942	ENSG00000148734	NPFFR1	filled
ENST00000536287	ENSG00000141314	RHBDL3	
ENST00000565548	ENSG00000137822	TUBGCP4	
ENST00000339861	ENSG00000187764	SEMA4D	filled
ENST00000492386	ENSG00000187764	SEMA4D	
ENST00000455551	ENSG00000187764	SEMA4D	filled
ENST00000450295	ENSG00000187764	SEMA4D	filled

ENST00000438547	ENSG00000187764	SEMA4D	filled
ENST00000422704	ENSG00000187764	SEMA4D	filled
ENST00000464051	ENSG00000187764	SEMA4D	
ENST00000482128	ENSG00000187764	SEMA4D	
ENST00000420987	ENSG00000187764	SEMA4D	filled
ENST00000356444	ENSG00000187764	SEMA4D	filled
ENST00000478415	ENSG00000165288	BRWD3	
ENST00000532482	ENSG00000134640	MTNR1B	filled
ENST00000529718	ENSG00000254788	CKLF-CMTM1	
ENST00000564978	ENSG00000176953	NFATC2IP	filled
ENST00000546296	ENSG00000111321	LTBR	filled
ENST00000542830	ENSG00000111321	LTBR	filled
ENST00000539925	ENSG00000111321	LTBR	filled
ENST00000390006	ENSG00000141562	NARF	filled
ENST00000345415	ENSG00000141562	NARF	filled
ENST00000374611	ENSG00000141562	NARF	filled
ENST00000457415	ENSG00000141562	NARF	filled
ENST00000584411	ENSG00000141562	NARF	
ENST00000577812	ENSG00000141562	NARF	filled
ENST00000577432	ENSG00000141562	NARF	filled
ENST00000582907	ENSG00000141562	NARF	filled
ENST00000578082	ENSG00000141562	NARF	
ENST00000412079	ENSG00000141562	NARF	filled
ENST00000393330	ENSG00000127324	TSPAN8	filled
ENST00000549421	ENSG00000127324	TSPAN8	filled
ENST00000544974	ENSG00000137821	LRRC49	filled
ENST00000558546	ENSG00000137821	LRRC49	filled
ENST00000535014	ENSG00000073614	KDM5A	
ENST00000536014	ENSG00000073614	KDM5A	
ENST00000373865	ENSG00000119397	CNTRL	
ENST00000471791	ENSG00000166866	MYO1A	
ENST00000558435	ENSG00000166920	C15orf48	
ENST00000592065	ENSG00000131095	GFAP	filled
ENST00000589701	ENSG00000131095	GFAP	
ENST00000592706	ENSG00000131095	GFAP	
ENST00000593179	ENSG00000131095	GFAP	
ENST00000589521	ENSG00000171443	ZNF524	
ENST00000301073	ENSG00000171443	ZNF524	
ENST00000591046	ENSG00000171443	ZNF524	
ENST00000447200	ENSG00000106483	SFRP4	filled
ENST00000420189	ENSG00000144567	FAM134A	
ENST00000580814	ENSG00000074319	TSG101	
ENST00000584526	ENSG00000074319	TSG101	
ENST00000592585	ENSG00000179943	FIZ1	
ENST00000587414	ENSG00000179943	FIZ1	
ENST00000420182	ENSG00000239836	PSMB9	
ENST00000448066	ENSG00000239836	PSMB9	filled
ENST00000473496	ENSG00000138109	CYP2C9	filled
ENST00000568423	ENSG00000198794	SCAMP5	filled
ENST00000425597	ENSG00000198794	SCAMP5	filled
ENST00000562212	ENSG00000198794	SCAMP5	filled
ENST00000562765	ENSG00000198794	SCAMP5	filled
ENST00000361900	ENSG00000198794	SCAMP5	filled
ENST00000567529	ENSG00000198794	SCAMP5	
ENST00000591305	ENSG00000175354	PTPN2	
ENST00000592059	ENSG00000175354	PTPN2	filled
ENST00000378133	ENSG00000204970	PCDHA1	
ENST00000393540	ENSG00000168491	CCDC110	
ENST00000307588	ENSG00000168491	CCDC110	
ENST00000510617	ENSG00000168491	CCDC110	

ENST00000510481	ENSG00000168491	CCDC110	
ENST00000506962	ENSG00000168491	CCDC110	
ENST00000332947	ENSG00000183114	FAM43B	
ENST00000536603	ENSG00000104983	CCDC61	
ENST00000378434	ENSG00000139679	LPAR6	filled
ENST00000345941	ENSG00000139679	LPAR6	
ENST00000620633	ENSG00000139679	LPAR6	
ENST00000254605	ENSG00000132275	RRP8	
ENST00000382292	ENSG00000151835	SACS	filled
ENST00000382298	ENSG00000151835	SACS	filled
ENST00000531096	ENSG00000166394	CYB5R2	filled
ENST00000527542	ENSG00000166394	CYB5R2	filled
ENST00000521468	ENSG00000147689	FAM83A	
ENST00000325421	ENSG00000218891	ZNF579	
ENST00000305596	ENSG00000169217	CD2BP2	
ENST00000451002	ENSG00000254959	INMT-FAM188B	
ENST00000565186	ENSG00000090857	PDPR	filled
ENST00000405592	ENSG00000057935	MTA3	filled
ENST00000461256	ENSG00000057935	MTA3	
ENST00000467925	ENSG00000057935	MTA3	filled
ENST00000572764	ENSG00000179314	WSCD1	filled
ENST00000453731	ENSG00000205639	MFSD2B	filled
ENST00000334478	ENSG00000123349	PFDN5	
ENST00000393892	ENSG00000173786	CNP	
ENST00000592446	ENSG00000173786	CNP	
ENST00000393888	ENSG00000173786	CNP	
ENST00000441615	ENSG00000173786	CNP	
ENST00000585452	ENSG00000173786	CNP	
ENST00000500320	ENSG00000009765	IYD	filled
ENST00000585785	ENSG00000177150	FAM210A	filled
ENST00000588475	ENSG00000177150	FAM210A	filled
ENST00000592976	ENSG00000177150	FAM210A	filled
ENST00000402563	ENSG00000177150	FAM210A	filled
ENST00000409340	ENSG00000138382	METTL5	
ENST00000564472	ENSG00000169783	LINGO1	filled
ENST00000506349	ENSG00000080224	EPHA6	
ENST00000549100	ENSG00000166153	DEPDC4	
ENST00000547823	ENSG00000166153	DEPDC4	
ENST00000468200	ENSG00000180116	C12orf40	filled
ENST00000604414	ENSG00000186188	FFAR4	filled
ENST00000371741	ENSG00000158445	KCNB1	
ENST00000368490	ENSG00000143515	ATP8B2	
ENST00000383699	ENSG00000175161	CADM2	filled
ENST00000473523	ENSG00000175161	CADM2	
ENST00000485126	ENSG00000175161	CADM2	
ENST00000377811	ENSG00000147123	NDUFB11	filled
ENST00000578956	ENSG00000167524	SGK494	
ENST00000494272	ENSG00000167524	SGK494	
ENST00000301037	ENSG00000167524	SGK494	
ENST00000376959	ENSG00000131398	KCNC3	filled
ENST00000474951	ENSG00000131398	KCNC3	filled
ENST00000412759	ENSG00000171943	SRGAP2C	
ENST00000618301	ENSG00000150275	PCDH15	
ENST00000476074	ENSG00000150275	PCDH15	
ENST00000615043	ENSG00000150275	PCDH15	
ENST00000463095	ENSG00000150275	PCDH15	
ENST00000552848	ENSG00000111481	COPZ1	filled
ENST00000548076	ENSG00000111481	COPZ1	filled
ENST00000528910	ENSG00000118369	USP35	
ENST00000494621	ENSG00000075292	ZNF638	filled
ENST00000466975	ENSG00000075292	ZNF638	filled
ENST00000466330	ENSG00000075292	ZNF638	filled
ENST00000512223	ENSG00000139154	AEBP2	filled
ENST00000262426	ENSG00000103241	FOXF1	
ENST00000577588	ENSG00000101773	RBBP8	filled
ENST00000582354	ENSG00000101773	RBBP8	filled
ENST00000581819	ENSG00000101773	RBBP8	filled
ENST00000327155	ENSG00000101773	RBBP8	filled
ENST00000399722	ENSG00000101773	RBBP8	filled
ENST00000399725	ENSG00000101773	RBBP8	filled
ENST00000360790	ENSG00000101773	RBBP8	filled
ENST00000583057	ENSG00000101773	RBBP8	filled
ENST00000581687	ENSG00000101773	RBBP8	
ENST00000434816	ENSG00000164638	SLC29A4	filled
ENST00000512596	ENSG00000176979	TRIM60	filled
ENST00000507119	ENSG00000176979	TRIM60	filled
ENST00000508504	ENSG00000176979	TRIM60	
ENST00000548136	ENSG00000258083	OR9A4	
ENST00000584630	ENSG00000134504	KCTD1	filled
ENST00000442800	ENSG00000180233	ZNRF2	
ENST00000357658	ENSG00000198198	SZT2	filled
ENST00000562955	ENSG00000198198	SZT2	filled
ENST00000470139	ENSG00000198198	SZT2	
ENST00000460536	ENSG00000198198	SZT2	
ENST00000508947	ENSG00000177752	YIPF7	
ENST00000564419	ENSG00000213380	COG8	
ENST00000550459	ENSG00000075188	NUP37	
ENST00000373614	ENSG00000118702	GHRH	
ENST00000586869	ENSG00000073536	NLE1	
ENST00000301886	ENSG00000273003	ARL2-SNX15	
ENST00000240139	ENSG00000120910	PPP3CC	filled
ENST00000289963	ENSG00000120910	PPP3CC	filled
ENST00000397775	ENSG00000120910	PPP3CC	filled
ENST00000611337	ENSG00000278535	DHRS11	
ENST00000608372	ENSG00000153130	SCOC	
ENST00000394201	ENSG00000153130	SCOC	
ENST00000246747	ENSG00000213465	ARL2	
ENST00000529384	ENSG00000213465	ARL2	
ENST00000533729	ENSG00000213465	ARL2	
ENST00000531533	ENSG00000213465	ARL2	
ENST00000529254	ENSG00000213465	ARL2	
ENST00000356542	ENSG00000188112	C6orf132	filled
ENST00000468413	ENSG00000155729	KCTD18	
ENST00000478816	ENSG00000155729	KCTD18	
ENST00000345034	ENSG00000215695	RSC1A1	
ENST00000310528	ENSG00000165416	SUGT1	
ENST00000609175	ENSG00000165416	SUGT1	
ENST00000323345	ENSG00000148303	RPL7A	
ENST00000463740	ENSG00000148303	RPL7A	filled
ENST00000468019	ENSG00000148303	RPL7A	
ENST00000315731	ENSG00000148303	RPL7A	
ENST00000587935	ENSG00000141378	PTRH2	
ENST00000394818	ENSG00000149503	INCENP	filled
ENST00000434415	ENSG00000204946	ZNF783	filled
ENST00000373985	ENSG00000185532	PRKG1	filled
ENST00000373980	ENSG00000185532	PRKG1	filled
ENST00000373976	ENSG00000185532	PRKG1	filled
ENST00000372146	ENSG00000125484	GTF3C4	filled
ENST00000502991	ENSG00000035928	RFC1	
ENST00000504974	ENSG00000035928	RFC1	

ENST00000372557	ENSG00000101470	TNNC2	
ENST00000560079	ENSG00000166716	ZNF592	filled
ENST00000618477	ENSG00000166716	ZNF592	
ENST00000564436	ENSG00000161999	JMJD8	
ENST00000359333	ENSG00000178826	TMEM139	
ENST00000409541	ENSG00000178826	TMEM139	
ENST00000410004	ENSG00000178826	TMEM139	
ENST00000358758	ENSG00000167371	PRRT2	
ENST00000567659	ENSG00000167371	PRRT2	
ENST00000572820	ENSG00000167371	PRRT2	
ENST00000300797	ENSG00000167371	PRRT2	
ENST00000567551	ENSG00000167371	PRRT2	
ENST00000490408	ENSG00000176386	CDC26	
ENST00000454583	ENSG00000080345	RIF1	
ENST00000484077	ENSG00000080345	RIF1	
ENST00000467762	ENSG00000080345	RIF1	
ENST00000488088	ENSG00000130052	STARD8	filled
ENST00000470137	ENSG00000151773	CCDC122	
ENST00000614974	ENSG00000228875	CSNK2B	
ENST00000617578	ENSG00000228875	CSNK2B	
ENST00000613998	ENSG00000228875	CSNK2B	
ENST00000442247	ENSG00000203697	CAPN8	
ENST00000502651	ENSG00000164162	ANAPC10	
ENST00000309439	ENSG00000164162	ANAPC10	
ENST00000448979	ENSG00000267281	RP11-793H13.10	
ENST00000377765	ENSG00000070601	FRMPD1	filled
ENST00000539465	ENSG00000070601	FRMPD1	filled
ENST00000361189	ENSG00000198961	PJA2	
ENST00000431537	ENSG00000232005	PBX2	filled
ENST00000339475	ENSG00000165588	OTX2	
ENST00000408990	ENSG00000165588	OTX2	
ENST00000555006	ENSG00000165588	OTX2	
ENST00000554845	ENSG00000165588	OTX2	
ENST00000554559	ENSG00000165588	OTX2	
ENST00000422021	ENSG00000182983	ZNF662	
ENST00000494744	ENSG00000177138	FAM9B	
ENST00000573108	ENSG00000121060	TRIM25	
ENST00000485545	ENSG00000049541	RFC2	filled
ENST00000461346	ENSG00000135835	KIAA1614	filled
ENST00000496039	ENSG00000187595	ZNF385C	
ENST00000461831	ENSG00000187595	ZNF385C	
ENST00000436535	ENSG00000187595	ZNF385C	
ENST00000524423	ENSG00000175097	RAG2	
ENST00000361987	ENSG00000242689	CNTF	
ENST00000482180	ENSG00000160703	NLRX1	filled
ENST00000409265	ENSG00000160703	NLRX1	filled
ENST00000274643	ENSG00000145949	MYLK4	filled
ENST00000598409	ENSG00000268182	SMIM17	filled
ENST00000600547	ENSG00000268182	SMIM17	filled
ENST00000467540	ENSG00000143222	UFC1	filled
ENST00000452573	ENSG00000236443	PSMB8	filled
ENST00000263174	ENSG00000099260	PALMD	filled
ENST00000605497	ENSG00000099260	PALMD	filled
ENST00000544406	ENSG00000214215	C12orf74	
ENST00000310085	ENSG00000173013	CCDC96	
ENST00000293851	ENSG00000103355	PRSS33	
ENST00000612593	ENSG00000103355	PRSS33	
ENST00000347055	ENSG00000172086	KRCC1	filled
ENST00000376312	ENSG00000204548	DEFB121	
ENST00000427980	ENSG00000224470	ATXN1L	filled
ENST00000569119	ENSG00000224470	ATXN1L	filled
ENST00000565676	ENSG00000224470	ATXN1L	
ENST00000474485	ENSG00000225921	NOL7	
ENST00000297375	ENSG00000164778	EN2	
ENST00000549890	ENSG00000118432	CNR1	
ENST00000583054	ENSG00000154655	L3MBTL4	filled
ENST00000580162	ENSG00000154655	L3MBTL4	filled
ENST00000570054	ENSG00000260914	RP11-343C2.11	
ENST00000375520	ENSG00000204365	C10orf126	
ENST00000614533	ENSG00000204365	C10orf126	
ENST00000397536	ENSG00000188549	C15orf52	
ENST00000382688	ENSG00000188549	C15orf52	
ENST00000513421	ENSG00000072201	LNX1	filled
ENST00000388996	ENSG00000184156	KCNQ3	filled
ENST00000521134	ENSG00000184156	KCNQ3	filled
ENST00000519445	ENSG00000184156	KCNQ3	filled
ENST00000519589	ENSG00000184156	KCNQ3	filled
ENST00000550595	ENSG00000166598	HSP90B1	filled
ENST00000509902	ENSG00000172869	DMXL1	filled
ENST00000593962	ENSG00000268790	CTC-429P9.4	filled
ENST00000601636	ENSG00000268790	CTC-429P9.4	filled
ENST00000593991	ENSG00000268790	CTC-429P9.4	filled
ENST00000595505	ENSG00000268790	CTC-429P9.4	filled
ENST00000593459	ENSG00000268790	CTC-429P9.4	filled
ENST00000594509	ENSG00000268790	CTC-429P9.4	filled
ENST00000600705	ENSG00000268790	CTC-429P9.4	filled
ENST00000416739	ENSG00000111224	PARP11	
ENST00000467314	ENSG00000260027	HOXB7	filled
ENST00000319675	ENSG00000176566	DCAF4L2	
ENST00000368655	ENSG00000143614	GATAD2B	filled
ENST00000332647	ENSG00000179083	FAM133A	
ENST00000322139	ENSG00000179083	FAM133A	
ENST00000492562	ENSG00000068438	FTSJ1	
ENST00000473235	ENSG00000068438	FTSJ1	
ENST00000496365	ENSG00000068438	FTSJ1	
ENST00000467954	ENSG00000068438	FTSJ1	
ENST00000489599	ENSG00000068438	FTSJ1	
ENST00000396894	ENSG00000068438	FTSJ1	
ENST00000563332	ENSG00000089486	CDIP1	
ENST00000399599	ENSG00000089486	CDIP1	
ENST00000566234	ENSG00000089486	CDIP1	
ENST00000412676	ENSG00000174600	CMKLR1	
ENST00000542384	ENSG00000131196	NFATC1	filled
ENST00000427363	ENSG00000131196	NFATC1	filled
ENST00000356415	ENSG00000116473	RAP1A	
ENST00000546961	ENSG00000196876	SCN8A	filled
ENST00000579005	ENSG00000161526	SAP30BP	
ENST00000600067	ENSG00000269403	CTD-2616J11.11	
ENST00000433688	ENSG00000188070	C11orf95	
ENST00000453074	ENSG00000089195	TRMT6	
ENST00000608928	ENSG00000157540	DYRK1A	filled
ENST00000340413	ENSG00000120253	NUP43	filled
ENST00000463048	ENSG00000120253	NUP43	
ENST00000377716	ENSG00000122696	SLC25A51	filled
ENST00000242275	ENSG00000122696	SLC25A51	filled
ENST00000457408	ENSG00000158604	TMED4	
ENST00000289577	ENSG00000158604	TMED4	
ENST00000558829	ENSG00000104043	ATP8B4	
ENST00000512342	ENSG00000109667	SLC2A9	filled
ENST00000503280	ENSG00000109667	SLC2A9	filled

ENST00000618862	ENSG00000274874	RP11-214K3.25	filled
ENST00000314607	ENSG00000146463	ZMYM4	filled
ENST00000262811	ENSG00000099308	MAST3	filled
ENST00000608648	ENSG00000099308	MAST3	
ENST00000409911	ENSG00000176204	LRRTM4	filled
ENST00000409884	ENSG00000176204	LRRTM4	filled
ENST00000409093	ENSG00000176204	LRRTM4	filled
ENST00000587069	ENSG00000129354	AP1M2	
ENST00000564866	ENSG00000261371	PECAM1	filled
ENST00000569967	ENSG00000261371	PECAM1	filled
ENST00000586434	ENSG00000131196	NFATC1	filled
ENST00000441905	ENSG00000003509	NDUFAF7	
ENST00000242208	ENSG00000122641	INHBA	
ENST00000567841	ENSG00000132604	TERF2	filled
ENST00000498634	ENSG00000120068	HOXB8	filled
ENST00000609683	ENSG00000183621	ZNF438	filled
ENST00000449534	ENSG00000237412	PRSS56	
ENST00000602410	ENSG00000237412	PRSS56	
ENST00000617714	ENSG00000237412	PRSS56	
ENST00000515235	ENSG00000133835	HSD17B4	filled
ENST00000551804	ENSG00000152556	PFKM	
ENST00000547587	ENSG00000152556	PFKM	
ENST00000546465	ENSG00000152556	PFKM	
ENST00000550802	ENSG00000152556	PFKM	
ENST00000553055	ENSG00000152556	PFKM	
ENST00000382848	ENSG00000165474	GJB2	
ENST00000382844	ENSG00000165474	GJB2	
ENST00000413325	ENSG00000158528	PPP1R9A	
ENST00000422324	ENSG00000158528	PPP1R9A	
ENST00000461150	ENSG00000169598	DFFB	
ENST00000268489	ENSG00000140836	ZFHX3	filled
ENST00000397992	ENSG00000140836	ZFHX3	filled
ENST00000558842	ENSG00000140836	ZFHX3	
ENST00000399796	ENSG00000131100	ATP6V1E1	
ENST00000399798	ENSG00000131100	ATP6V1E1	
ENST00000608872	ENSG00000119402	FBXW2	
ENST00000456080	ENSG00000229972	IQCF3	filled
ENST00000437810	ENSG00000229972	IQCF3	
ENST00000446775	ENSG00000229972	IQCF3	
ENST00000472485	ENSG00000229972	IQCF3	
ENST00000465028	ENSG00000229972	IQCF3	
ENST00000474242	ENSG00000229972	IQCF3	
ENST00000532403	ENSG00000166788	SAAL1	
ENST00000597304	ENSG00000167785	ZNF558	
ENST00000460779	ENSG00000151577	DRD3	filled
ENST00000371992	ENSG00000085998	POMGNT1	filled
ENST00000396420	ENSG00000085998	POMGNT1	filled
ENST00000598361	ENSG00000006625	GGCT	filled
ENST00000587871	ENSG00000267110	CTD-2587H24.4	
ENST00000375095	ENSG00000181264	TMEM136	
ENST00000531346	ENSG00000181264	TMEM136	
ENST00000529187	ENSG00000181264	TMEM136	
ENST00000373602	ENSG00000160050	CCDC28B	
ENST00000469003	ENSG00000160050	CCDC28B	
ENST00000421922	ENSG00000160050	CCDC28B	
ENST00000461819	ENSG00000160050	CCDC28B	
ENST00000383571	ENSG00000206484	NRM	
ENST00000297625	ENSG00000164976	KIAA1161	
ENST00000451025	ENSG00000124688	MAD2L1BP	
ENST00000429029	ENSG00000105993	DNAJB6	

ENST00000412557	ENSG00000105993	DNAJB6	filled
ENST00000419606	ENSG00000168397	ATG4B	
ENST00000235307	ENSG00000116667	C1orf21	filled
ENST00000249636	ENSG00000033800	PIAS1	
ENST00000564915	ENSG00000033800	PIAS1	
ENST00000562190	ENSG00000033800	PIAS1	
ENST00000545237	ENSG00000033800	PIAS1	
ENST00000567417	ENSG00000033800	PIAS1	filled
ENST00000563996	ENSG00000033800	PIAS1	
ENST00000258403	ENSG00000135917	SLC19A3	
ENST00000399855	ENSG00000198130	HIBCH	
ENST00000409934	ENSG00000198130	HIBCH	filled
ENST00000589774	ENSG00000006125	AP2B1	filled
ENST00000460676	ENSG00000125877	ITPA	
ENST00000417036	ENSG00000134077	THUMPD3	
ENST00000590854	ENSG00000152223	EPG5	
ENST00000463153	ENSG00000011198	ABHD5	
ENST00000388712	ENSG00000135052	GOLM1	
ENST00000388711	ENSG00000135052	GOLM1	
ENST00000486130	ENSG00000135052	GOLM1	
ENST00000466178	ENSG00000135052	GOLM1	filled
ENST00000601520	ENSG00000167378	IRGQ	
ENST00000598324	ENSG00000167378	IRGQ	
ENST00000418046	ENSG00000128610	FEZF1	
ENST00000362032	ENSG00000198829	SUCNR1	
ENST00000503175	ENSG00000175414	ARL10	
ENST00000366935	ENSG00000092978	GPATCH2	filled
ENST00000489246	ENSG00000092978	GPATCH2	
ENST00000470014	ENSG00000092978	GPATCH2	filled
ENST00000485274	ENSG00000092978	GPATCH2	filled
ENST00000366934	ENSG00000092978	GPATCH2	
ENST00000296135	ENSG00000163818	LZTFL1	
ENST00000418700	ENSG00000163818	LZTFL1	
ENST00000448111	ENSG00000163818	LZTFL1	
ENST00000495864	ENSG00000163818	LZTFL1	
ENST00000480156	ENSG00000163818	LZTFL1	
ENST00000445698	ENSG00000163818	LZTFL1	
ENST00000492333	ENSG00000163818	LZTFL1	
ENST00000472635	ENSG00000163818	LZTFL1	
ENST00000536047	ENSG00000163818	LZTFL1	
ENST00000539217	ENSG00000163818	LZTFL1	
ENST00000566625	ENSG00000179965	ZNF771	
ENST00000560629	ENSG00000259753	ITGB3	filled
ENST00000299575	ENSG00000166454	ATMIN	
ENST00000566488	ENSG00000166454	ATMIN	
ENST00000621271	ENSG00000070371	CLTCL1	filled
ENST00000427926	ENSG00000070371	CLTCL1	filled
ENST00000622493	ENSG00000070371	CLTCL1	filled
ENST00000617926	ENSG00000070371	CLTCL1	filled
ENST00000615606	ENSG00000070371	CLTCL1	filled
ENST00000617103	ENSG00000070371	CLTCL1	filled
ENST00000538828	ENSG00000070371	CLTCL1	filled
ENST00000611723	ENSG00000070371	CLTCL1	
ENST00000458188	ENSG00000070371	CLTCL1	
ENST00000540896	ENSG00000070371	CLTCL1	
ENST00000455783	ENSG00000234224	TMEM229A	
ENST00000591346	ENSG00000125910	S1PR4	
ENST00000246115	ENSG00000125910	S1PR4	
ENST00000400331	ENSG00000183742	MACC1	
ENST00000332878	ENSG00000183742	MACC1	

ENST00000471019	ENSG00000183742	MACC1	
ENST00000483317	ENSG00000183742	MACC1	
ENST00000308527	ENSG00000173548	SNX33	
ENST00000322348	ENSG00000176928	GCNT4	filled
ENST00000494072	ENSG00000255275	RP13-279N23.2	filled
ENST00000594144	ENSG00000142230	SAE1	filled
ENST00000483656	ENSG00000094631	HDAC6	
ENST00000477561	ENSG00000094631	HDAC6	
ENST00000441703	ENSG00000094631	HDAC6	
ENST00000426196	ENSG00000094631	HDAC6	
ENST00000312349	ENSG00000172568	FNDC9	
ENST00000587244	ENSG00000166569	CPLX4	filled
ENST00000434353	ENSG00000197620	CXorf40A	
ENST00000313421	ENSG00000013725	CD6	filled
ENST00000538288	ENSG00000013725	CD6	
ENST00000327674	ENSG00000123561	SERPINA7	
ENST00000372563	ENSG00000123561	SERPINA7	
ENST00000487487	ENSG00000123561	SERPINA7	
ENST00000482797	ENSG00000078725	BRINP1	filled
ENST00000394340	ENSG00000136848	DAB2IP	filled
ENST00000436835	ENSG00000136848	DAB2IP	filled
ENST00000489314	ENSG00000136848	DAB2IP	filled
ENST00000408936	ENSG00000136848	DAB2IP	filled
ENST00000465078	ENSG00000136848	DAB2IP	filled
ENST00000309989	ENSG00000136848	DAB2IP	
ENST00000259371	ENSG00000136848	DAB2IP	filled
ENST00000372492	ENSG00000243710	CFAP57	filled
ENST00000610710	ENSG00000243710	CFAP57	filled
ENST00000371375	ENSG00000138193	PLCE1	filled
ENST00000377604	ENSG00000182872	RBM10	
ENST00000345781	ENSG00000182872	RBM10	
ENST00000329236	ENSG00000182872	RBM10	
ENST00000324048	ENSG00000143373	ZNF687	filled
ENST00000548145	ENSG00000257473	HLA-DQA2	filled
ENST00000552745	ENSG00000257473	HLA-DQA2	filled
ENST00000425393	ENSG00000115839	RAB3GAP1	filled
ENST00000584672	ENSG00000141551	CSNK1D	
ENST00000520258	ENSG00000158669	AGPAT6	
ENST00000520223	ENSG00000158669	AGPAT6	
ENST00000521121	ENSG00000158669	AGPAT6	
ENST00000519921	ENSG00000158669	AGPAT6	filled
ENST00000327419	ENSG00000185742	C11orf87	
ENST00000332503	ENSG00000182742	HOXB4	
ENST00000451237	ENSG00000235863	B3GALT4	filled
ENST00000606990	ENSG00000235863	B3GALT4	
ENST00000497554	ENSG00000257335	MGAM	
ENST00000537239	ENSG00000256966	RP11-613M10.8	filled
ENST00000558921	ENSG00000169926	KLF13	filled
ENST00000558673	ENSG00000169926	KLF13	filled
ENST00000558225	ENSG00000169926	KLF13	filled
ENST00000367175	ENSG00000170382	LRRN2	filled
ENST00000367176	ENSG00000170382	LRRN2	filled
ENST00000367177	ENSG00000170382	LRRN2	filled
ENST00000286317	ENSG00000155868	MED7	
ENST00000491456	ENSG00000138061	CYP1B1	
ENST00000610745	ENSG00000138061	CYP1B1	
ENST00000378788	ENSG00000205108	FAM205A	
ENST00000432590	ENSG00000107872	FBXL15	
ENST00000481808	ENSG00000107872	FBXL15	
ENST00000426095	ENSG00000226704	HSPA1L	

ENST00000331173	ENSG00000177613	CSTF2T	
ENST00000409185	ENSG00000152102	FAM168B	filled
ENST00000389915	ENSG00000152102	FAM168B	filled
ENST00000544911	ENSG00000256950	RP11-87C12.2	filled
ENST00000412450	ENSG00000181555	SETD2	filled
ENST00000422787	ENSG00000154646	TMPRSS15	filled
ENST00000455778	ENSG00000099139	PCSK5	
ENST00000291294	ENSG00000160013	PTGIR	
ENST00000597185	ENSG00000160013	PTGIR	
ENST00000361609	ENSG00000144355	DLX1	
ENST00000469444	ENSG00000144355	DLX1	
ENST00000420891	ENSG00000177842	ZNF620	
ENST00000433723	ENSG00000177842	ZNF620	
ENST00000441960	ENSG00000230034	PSMB8	filled
ENST00000412973	ENSG00000106541	AGR2	filled
ENST00000437102	ENSG00000180432	CYP8B1	filled
ENST00000316161	ENSG00000180432	CYP8B1	
ENST00000519126	ENSG00000137648	TMPRSS4	
ENST00000534111	ENSG00000137648	TMPRSS4	filled
ENST00000519236	ENSG00000137648	TMPRSS4	filled
ENST00000522307	ENSG00000137648	TMPRSS4	filled
ENST00000523251	ENSG00000137648	TMPRSS4	filled
ENST00000517483	ENSG00000137648	TMPRSS4	filled
ENST00000437212	ENSG00000137648	TMPRSS4	filled
ENST00000522824	ENSG00000137648	TMPRSS4	filled
ENST00000520063	ENSG00000137648	TMPRSS4	
ENST00000618855	ENSG00000137648	TMPRSS4	filled
ENST00000616579	ENSG00000137648	TMPRSS4	filled
ENST00000610964	ENSG00000137648	TMPRSS4	filled
ENST00000341698	ENSG00000124208	TMEM189-UBE2V1	filled
ENST00000534024	ENSG00000172375	C2CD2L	
ENST00000528271	ENSG00000172375	C2CD2L	
ENST00000529600	ENSG00000172375	C2CD2L	
ENST00000529885	ENSG00000172375	C2CD2L	
ENST00000527854	ENSG00000172375	C2CD2L	
ENST00000315927	ENSG00000180667	YOD1	
ENST00000367084	ENSG00000180667	YOD1	
ENST00000418093	ENSG00000273291	RP11-136C24.3	filled
ENST00000443313	ENSG00000273291	RP11-136C24.3	filled
ENST00000524981	ENSG00000188596	CFAP54	filled
ENST00000449528	ENSG00000058091	CDK14	filled
ENST00000462705	ENSG00000181722	ZBTB20	filled
ENST00000357258	ENSG00000181722	ZBTB20	filled
ENST00000492665	ENSG00000181722	ZBTB20	filled
ENST00000488663	ENSG00000181722	ZBTB20	filled
ENST00000452722	ENSG00000182985	CADM1	filled
ENST00000537140	ENSG00000182985	CADM1	filled
ENST00000542447	ENSG00000182985	CADM1	filled
ENST00000537058	ENSG00000182985	CADM1	filled
ENST00000536727	ENSG00000182985	CADM1	filled
ENST00000545380	ENSG00000182985	CADM1	filled
ENST00000331581	ENSG00000182985	CADM1	filled
ENST00000541434	ENSG00000182985	CADM1	filled
ENST00000545094	ENSG00000182985	CADM1	
ENST00000543249	ENSG00000182985	CADM1	filled
ENST00000540951	ENSG00000182985	CADM1	filled
ENST00000536781	ENSG00000182985	CADM1	filled
ENST00000534420	ENSG00000254673	RP11-598P20.5	filled
ENST00000517894	ENSG00000181790	BAI1	filled
ENST00000521208	ENSG00000181790	BAI1	filled

ENST00000518812	ENSG00000181790	BAI1	
ENST00000323289	ENSG00000181790	BAI1	filled
ENST00000311549	ENSG00000122026	RPL21	
ENST00000473558	ENSG00000122026	RPL21	
ENST00000319826	ENSG00000122026	RPL21	
ENST00000326092	ENSG00000122026	RPL21	
ENST00000493317	ENSG00000122026	RPL21	
ENST00000466550	ENSG00000122026	RPL21	filled
ENST00000483765	ENSG00000122026	RPL21	
ENST00000522037	ENSG00000120896	SORBS3	
ENST00000523941	ENSG00000120896	SORBS3	
ENST00000309384	ENSG00000183542	KLRC4	
ENST00000444368	ENSG00000132405	TBC1D14	filled
ENST00000414347	ENSG00000166897	ELFN2	filled
ENST00000451509	ENSG00000166897	ELFN2	filled
ENST00000415408	ENSG00000166897	ELFN2	filled
ENST00000331251	ENSG00000183729	NPBWR1	
ENST00000595615	ENSG00000178980	SEPW1	
ENST00000599627	ENSG00000178980	SEPW1	
ENST00000508075	ENSG00000069869	NEDD4	filled
ENST00000557845	ENSG00000069869	NEDD4	filled
ENST00000571365	ENSG00000108523	RNF167	
ENST00000531030	ENSG00000141568	FOXK2	filled
ENST00000526383	ENSG00000141568	FOXK2	filled
ENST00000216027	ENSG00000100209	HSCB	filled
ENST00000450178	ENSG00000100209	HSCB	filled
ENST00000485599	ENSG00000100209	HSCB	filled
ENST00000375535	ENSG00000117122	MFAP2	filled
ENST00000490035	ENSG00000185565	LSAMP	filled
ENST00000333617	ENSG00000185565	LSAMP	filled
ENST00000474851	ENSG00000185565	LSAMP	filled
ENST00000369528	ENSG00000052723	SIKE1	
ENST00000543982	ENSG00000073050	XRCC1	
ENST00000597811	ENSG00000073050	XRCC1	
ENST00000416284	ENSG00000198673	FAM19A2	filled
ENST00000549379	ENSG00000198673	FAM19A2	filled
ENST00000551449	ENSG00000198673	FAM19A2	filled
ENST00000552075	ENSG00000198673	FAM19A2	filled
ENST00000549958	ENSG00000198673	FAM19A2	filled
ENST00000548780	ENSG00000198673	FAM19A2	filled
ENST00000549456	ENSG00000198673	FAM19A2	filled
ENST00000547919	ENSG00000198673	FAM19A2	filled
ENST00000550060	ENSG00000198673	FAM19A2	filled
ENST00000547075	ENSG00000198673	FAM19A2	
ENST00000547941	ENSG00000198673	FAM19A2	
ENST00000281496	ENSG00000102763	VWA8	filled
ENST00000421670	ENSG00000111364	DDX55	filled
ENST00000243298	ENSG00000123570	RAB9B	
ENST00000506866	ENSG00000145391	SETD7	filled
ENST00000515101	ENSG00000145391	SETD7	
ENST00000532414	ENSG00000254726	MEX3A	
ENST00000481405	ENSG00000075914	EXOSC7	filled
ENST00000459856	ENSG00000075914	EXOSC7	
ENST00000587092	ENSG00000267757	C19orf83	
ENST00000534108	ENSG00000197165	SULT1A2	filled
ENST00000533150	ENSG00000197165	SULT1A2	filled
ENST00000395630	ENSG00000197165	SULT1A2	filled
ENST00000492747	ENSG00000162814	SPATA17	filled
ENST00000296794	ENSG00000214944	ARHGEF28	filled
ENST00000509848	ENSG00000214944	ARHGEF28	filled

ENST00000296799	ENSG00000214944	ARHGEF28	filled
ENST00000513042	ENSG00000214944	ARHGEF28	filled
ENST00000545377	ENSG00000214944	ARHGEF28	filled
ENST00000409338	ENSG00000257529	RPL36A-HNRNPH2	filled
ENST00000409170	ENSG00000257529	RPL36A-HNRNPH2	filled
ENST00000374049	ENSG00000157693	C9orf91	filled
ENST00000482552	ENSG00000157693	C9orf91	
ENST00000561148	ENSG00000171766	GATM	
ENST00000560538	ENSG00000171766	GATM	
ENST00000458245	ENSG00000171766	GATM	
ENST00000393957	ENSG00000159713	TPPP3	
ENST00000564104	ENSG00000159713	TPPP3	
ENST00000290942	ENSG00000159713	TPPP3	
ENST00000562206	ENSG00000159713	TPPP3	
ENST00000561537	ENSG00000159713	TPPP3	
ENST00000536764	ENSG00000256029	RP11-190A12.7	
ENST00000536779	ENSG00000256029	RP11-190A12.7	
ENST00000544342	ENSG00000256029	RP11-190A12.7	
ENST00000537167	ENSG00000256029	RP11-190A12.7	
ENST00000543372	ENSG00000256029	RP11-190A12.7	
ENST00000505049	ENSG00000164124	TMEM144	
ENST00000505189	ENSG00000164124	TMEM144	
ENST00000511038	ENSG00000164124	TMEM144	
ENST00000561579	ENSG00000159593	NAE1	
ENST00000568581	ENSG00000182149	IST1	filled
ENST00000606369	ENSG00000182149	IST1	
ENST00000424347	ENSG00000148019	CEP78	filled
ENST00000381026	ENSG00000180389	ATP5EP2	
ENST00000465120	ENSG00000120093	HOXB3	
ENST00000464266	ENSG00000120093	HOXB3	
ENST00000463483	ENSG00000163219	ARHGAP25	filled
ENST00000503050	ENSG00000161021	MAML1	filled
ENST00000468628	ENSG00000163788	SNRK	filled
ENST00000476951	ENSG00000156711	MAPK13	
ENST00000373766	ENSG00000156711	MAPK13	
ENST00000467401	ENSG00000165238	WNK2	
ENST00000557530	ENSG00000166165	CKB	
ENST00000478890	ENSG00000133226	SRRM1	filled
ENST00000593639	ENSG00000133226	SRRM1	filled
ENST00000461768	ENSG00000133226	SRRM1	
ENST00000305386	ENSG00000168282	MGAT2	
ENST00000568630	ENSG00000174197	MGA	filled
ENST00000563576	ENSG00000174197	MGA	filled
ENST00000329759	ENSG00000185112	FAM43A	
ENST00000395131	ENSG00000204220	PFDN6	
ENST00000463584	ENSG00000204220	PFDN6	
ENST00000318438	ENSG00000179751	SYCN	
ENST00000431830	ENSG00000235692	PFDN6	
ENST00000468094	ENSG00000235692	PFDN6	
ENST00000443537	ENSG00000048991	R3HDM1	filled
ENST00000369823	ENSG00000134184	GSTM1	
ENST00000490171	ENSG00000134184	GSTM1	
ENST00000549372	ENSG00000089060	SLC8B1	
ENST00000562144	ENSG00000196155	PLEKHG4	
ENST00000565899	ENSG00000196155	PLEKHG4	
ENST00000463997	ENSG00000162909	CAPN2	
ENST00000367577	ENSG00000162783	IER5	
ENST00000422364	ENSG00000149761	NUDT22	filled
ENST00000314686	ENSG00000177873	ZNF619	
ENST00000462961	ENSG00000177873	ZNF619	

ENST00000520737	ENSG00000177873	ZNF619	
ENST00000494154	ENSG00000177873	ZNF619	
ENST00000522736	ENSG00000177873	ZNF619	
ENST00000521353	ENSG00000177873	ZNF619	
ENST00000432264	ENSG00000177873	ZNF619	
ENST00000456778	ENSG00000177873	ZNF619	
ENST00000355790	ENSG00000172731	LRRC20	
ENST00000373224	ENSG00000172731	LRRC20	
ENST00000358141	ENSG00000172731	LRRC20	
ENST00000357631	ENSG00000172731	LRRC20	
ENST00000446961	ENSG00000172731	LRRC20	
ENST00000395011	ENSG00000172731	LRRC20	
ENST00000395010	ENSG00000172731	LRRC20	
ENST00000553038	ENSG00000075089	ACTR6	filled
ENST00000551440	ENSG00000075089	ACTR6	filled
ENST00000449162	ENSG00000106624	AEBP1	
ENST00000487096	ENSG00000116221	MRPL37	filled
ENST00000496333	ENSG00000084676	NCOA1	filled
ENST00000406961	ENSG00000084676	NCOA1	filled
ENST00000405141	ENSG00000084676	NCOA1	filled
ENST00000373212	ENSG00000131171	SH3BGRL	
ENST00000463546	ENSG00000131171	SH3BGRL	
ENST00000466490	ENSG00000144867	SRPRB	filled
ENST00000466636	ENSG00000144867	SRPRB	filled
ENST00000265081	ENSG00000113318	MSH3	filled
ENST00000490145	ENSG00000138495	COX17	
ENST00000468918	ENSG00000138495	COX17	
ENST00000054666	ENSG00000049245	VAMP3	
ENST00000572275	ENSG00000170049	KCNAB3	
ENST00000546455	ENSG00000179715	PCED1B	filled
ENST00000432328	ENSG00000179715	PCED1B	filled
ENST00000592533	ENSG00000171817	ZNF540	filled
ENST00000589117	ENSG00000171817	ZNF540	filled
ENST00000587220	ENSG00000171817	ZNF540	
ENST00000589857	ENSG00000171817	ZNF540	
ENST00000316433	ENSG00000171817	ZNF540	filled
ENST00000590588	ENSG00000171817	ZNF540	
ENST00000586134	ENSG00000171817	ZNF540	filled
ENST00000586792	ENSG00000171817	ZNF540	filled
ENST00000343599	ENSG00000171817	ZNF540	filled
ENST00000589285	ENSG00000171817	ZNF540	filled
ENST00000578189	ENSG00000271383	NBPF14	filled
ENST00000287020	ENSG00000156466	GDF6	
ENST00000531326	ENSG00000179295	PTPN11	
ENST00000556126	ENSG00000259120	SMIM6	
ENST00000569789	ENSG00000168447	SCNN1B	filled
ENST00000469157	ENSG00000145975	FAM217A	filled
ENST00000472595	ENSG00000184911	DMRTC1B	filled
ENST00000334036	ENSG00000184911	DMRTC1B	filled
ENST00000590411	ENSG00000204920	ZNF155	filled
ENST00000469739	ENSG00000177302	TOP3A	filled
ENST00000321105	ENSG00000177302	TOP3A	filled
ENST00000542570	ENSG00000177302	TOP3A	filled
ENST00000548498	ENSG00000211584	SLC48A1	filled
ENST00000552003	ENSG00000211584	SLC48A1	
ENST00000547002	ENSG00000211584	SLC48A1	filled
ENST00000557685	ENSG00000140025	EFCAB11	filled
ENST00000556609	ENSG00000140025	EFCAB11	filled
ENST00000553871	ENSG00000140025	EFCAB11	filled
ENST00000556639	ENSG00000140025	EFCAB11	filled
ENST00000555608	ENSG00000140025	EFCAB11	filled
ENST00000550103	ENSG00000140025	EFCAB11	filled
ENST00000538485	ENSG00000140025	EFCAB11	filled
ENST00000556005	ENSG00000140025	EFCAB11	filled
ENST00000554761	ENSG00000140025	EFCAB11	filled
ENST00000555430	ENSG00000140025	EFCAB11	filled
ENST00000593442	ENSG00000042753	AP2S1	
ENST00000600964	ENSG00000042753	AP2S1	
ENST00000572681	ENSG00000079432	CIC	filled
ENST00000571942	ENSG00000079432	CIC	
ENST00000575839	ENSG00000079432	CIC	
ENST00000440626	ENSG00000138449	SLC40A1	
ENST00000217246	ENSG00000172264	MACROD2	filled
ENST00000477147	ENSG00000172264	MACROD2	filled
ENST00000483997	ENSG00000172264	MACROD2	filled
ENST00000492055	ENSG00000172264	MACROD2	filled
ENST00000462552	ENSG00000172264	MACROD2	filled
ENST00000490428	ENSG00000172264	MACROD2	filled
ENST00000463861	ENSG00000172264	MACROD2	filled
ENST00000497992	ENSG00000172264	MACROD2	filled
ENST00000402914	ENSG00000172264	MACROD2	filled
ENST00000329565	ENSG00000184602	SNN	
ENST00000366131	ENSG00000111319	SCNN1A	filled
ENST00000538957	ENSG00000111319	SCNN1A	
ENST00000545605	ENSG00000111319	SCNN1A	
ENST00000284674	ENSG00000154478	GPR26	
ENST00000538872	ENSG00000120645	IQSEC3	filled
ENST00000398309	ENSG00000150672	DLG2	filled
ENST00000376104	ENSG00000150672	DLG2	filled
ENST00000495362	ENSG00000121577	POPDC2	
ENST00000306117	ENSG00000124134	KCNS1	
ENST00000465495	ENSG00000162461	SLC25A34	
ENST00000583367	ENSG00000134265	NAPG	filled
ENST00000480726	ENSG00000220201	ZGLP1	
ENST00000411529	ENSG00000170144	HNRNPA3	
ENST00000548156	ENSG00000072657	TRHDE	filled
ENST00000380874	ENSG00000054598	FOXC1	
ENST00000480662	ENSG00000138326	RPS24	
ENST00000472746	ENSG00000197816	CCDC180	
ENST00000430971	ENSG00000236802	B3GALT4	filled
ENST00000462736	ENSG00000183943	PRKX	
ENST00000479209	ENSG00000120742	SERP1	filled
ENST00000491195	ENSG00000120742	SERP1	filled
ENST00000463647	ENSG00000120742	SERP1	filled
ENST00000484608	ENSG00000120742	SERP1	filled
ENST00000239944	ENSG00000120742	SERP1	
ENST00000409559	ENSG00000119865	CNRIP1	
ENST00000598265	ENSG00000131126	TEX101	filled
ENST00000250056	ENSG00000129195	FAM64A	
ENST00000573557	ENSG00000129195	FAM64A	
ENST00000572595	ENSG00000129195	FAM64A	
ENST00000331968	ENSG00000184304	PRKD1	filled
ENST00000415220	ENSG00000184304	PRKD1	filled
ENST00000549503	ENSG00000184304	PRKD1	filled
ENST00000616995	ENSG00000184304	PRKD1	filled
ENST00000451862	ENSG00000243067	PSMB9	
ENST00000445315	ENSG00000243067	PSMB9	filled
ENST00000461096	ENSG00000197780	TAF13	
ENST00000338366	ENSG00000197780	TAF13	
ENST00000512868	ENSG00000172058	SERF1A	

ENST00000539240	ENSG00000139187	KLRG1	
ENST00000541957	ENSG00000139187	KLRG1	
ENST00000540557	ENSG00000255872	RP11-613M10.9	filled
ENST00000325719	ENSG00000181963	OR52K2	
ENST00000506237	ENSG00000113387	SUB1	filled
ENST00000512913	ENSG00000113387	SUB1	filled
ENST00000595920	ENSG00000090924	PLEKHG2	
ENST00000438123	ENSG00000090924	PLEKHG2	
ENST00000451354	ENSG00000090924	PLEKHG2	
ENST00000600210	ENSG00000090924	PLEKHG2	
ENST00000506607	ENSG00000249428	CFAP99	
ENST00000616117	ENSG00000249428	CFAP99	
ENST00000322177	ENSG00000178718	RPP25	
ENST00000553232	ENSG00000122965	RBM19	
ENST00000558383	ENSG00000140279	DUOX2	filled
ENST00000392366	ENSG00000197063	MAFG	
ENST00000445968	ENSG00000233948	TRIM27	
ENST00000440169	ENSG00000233948	TRIM27	
ENST00000443804	ENSG00000233948	TRIM27	
ENST00000359321	ENSG00000196584	XRCC2	filled
ENST00000560932	ENSG00000159337	PLA2G4D	
ENST00000290472	ENSG00000159337	PLA2G4D	
ENST00000476620	ENSG00000086289	EPDR1	filled
ENST00000334512	ENSG00000108175	ZMIZ1	filled
ENST00000448684	ENSG00000236697	GPSM3	
ENST00000383390	ENSG00000206383	HSPA1L	
ENST00000431056	ENSG00000161267	BDH1	filled
ENST00000358186	ENSG00000161267	BDH1	filled
ENST00000511644	ENSG00000151247	EIF4E	
ENST00000561562	ENSG00000260300	RP11-505K9.4	filled
ENST00000486811	ENSG00000153266	FEZF2	
ENST00000283268	ENSG00000153266	FEZF2	
ENST00000475839	ENSG00000153266	FEZF2	
ENST00000546654	ENSG00000229117	RPL41	
ENST00000431315	ENSG00000163705	FANCD2OS	
ENST00000436517	ENSG00000163705	FANCD2OS	
ENST00000524279	ENSG00000163705	FANCD2OS	
ENST00000560394	ENSG00000157470	FAM81A	filled
ENST00000424789	ENSG00000170921	TANC2	filled
ENST00000389520	ENSG00000170921	TANC2	filled
ENST00000581424	ENSG00000170921	TANC2	
ENST00000518136	ENSG00000106031	HOXA13	
ENST00000297324	ENSG00000164743	C8orf48	
ENST00000473763	ENSG00000107165	TYRP1	filled
ENST00000519065	ENSG00000196591	HDAC2	filled
ENST00000477760	ENSG00000226404	LY6G5C	
ENST00000479313	ENSG00000226404	LY6G5C	
ENST00000435280	ENSG00000226404	LY6G5C	
ENST00000369535	ENSG00000213281	NRAS	
ENST00000486969	ENSG00000076356	PLXNA2	
ENST00000513107	ENSG00000070193	FGF10	
ENST00000241453	ENSG00000122025	FLT3	filled
ENST00000380987	ENSG00000122025	FLT3	filled
ENST00000359175	ENSG00000196542	SPTSSB	filled
ENST00000497137	ENSG00000196542	SPTSSB	filled
ENST00000497374	ENSG00000196542	SPTSSB	
ENST00000617024	ENSG00000196542	SPTSSB	
ENST00000602926	ENSG00000267228	IER3IP1	
ENST00000588705	ENSG00000267228	IER3IP1	
ENST00000471915	ENSG00000189266	PNRC2	

ENST00000579103	ENSG00000189266	PNRC2	
ENST00000553819	ENSG00000184990	SIVA1	
ENST00000554087	ENSG00000100554	ATP6V1D	filled
ENST00000569461	ENSG00000166451	CENPN	
ENST00000428963	ENSG00000166451	CENPN	
ENST00000483143	ENSG00000160097	FNDC5	
ENST00000481487	ENSG00000160097	FNDC5	
ENST00000373471	ENSG00000160097	FNDC5	
ENST00000497068	ENSG00000160097	FNDC5	
ENST00000316803	ENSG00000157782	CABP1	
ENST00000417056	ENSG00000135842	FAM129A	filled
ENST00000512909	ENSG00000197386	HTT	
ENST00000428566	ENSG00000206240	HLA-DRB1	filled
ENST00000504772	ENSG00000173917	HOXB2	
ENST00000620890	ENSG00000233276	GPX1	
ENST00000588030	ENSG00000099622	CIRBP	
ENST00000588411	ENSG00000099622	CIRBP	
ENST00000534281	ENSG00000110315	RNF141	
ENST00000458269	ENSG00000082146	STRADB	filled
ENST00000368301	ENSG00000160789	LMNA	filled
ENST00000300069	ENSG00000166831	RBPMS2	
ENST00000377321	ENSG00000133895	MEN1	
ENST00000524982	ENSG00000150672	DLG2	filled
ENST00000532653	ENSG00000150672	DLG2	filled
ENST00000529111	ENSG00000150672	DLG2	filled
ENST00000527466	ENSG00000150672	DLG2	filled
ENST00000527088	ENSG00000150672	DLG2	filled
ENST00000530589	ENSG00000150672	DLG2	filled
ENST00000472545	ENSG00000150672	DLG2	filled
ENST00000519833	ENSG00000104432	IL7	filled
ENST00000474694	ENSG00000088205	DDX18	filled
ENST00000415038	ENSG00000088205	DDX18	filled
ENST00000413927	ENSG00000106304	SPAM1	
ENST00000460182	ENSG00000106304	SPAM1	
ENST00000439500	ENSG00000106304	SPAM1	
ENST00000483890	ENSG00000107651	SEC23IP	
ENST00000239451	ENSG00000120329	SLC25A2	filled
ENST00000261658	ENSG00000103429	BFAR	filled
ENST00000566520	ENSG00000103429	BFAR	
ENST00000563971	ENSG00000103429	BFAR	filled
ENST00000570219	ENSG00000103429	BFAR	
ENST00000565478	ENSG00000103429	BFAR	
ENST00000563082	ENSG00000103429	BFAR	
ENST00000514924	ENSG00000154277	UCHL1	
ENST00000503431	ENSG00000154277	UCHL1	
ENST00000505232	ENSG00000154277	UCHL1	
ENST00000508768	ENSG00000154277	UCHL1	
ENST00000421627	ENSG00000198010	DLGAP2	filled
ENST00000612087	ENSG00000198010	DLGAP2	filled
ENST00000500893	ENSG00000247315	ZCCHC3	
ENST00000425067	ENSG00000225141	PRRT1	
ENST00000489899	ENSG00000225141	PRRT1	
ENST00000487441	ENSG00000225141	PRRT1	
ENST00000469199	ENSG00000225141	PRRT1	
ENST00000515489	ENSG00000109381	ELF2	
ENST00000394235	ENSG00000109381	ELF2	filled
ENST00000511184	ENSG00000109381	ELF2	
ENST00000339235	ENSG00000171823	FBXL14	filled
ENST00000543278	ENSG00000171823	FBXL14	filled
ENST00000368464	ENSG00000146350	TBC1D32	filled

ENST00000438528	ENSG00000176209	SMIM19	
ENST00000326737	ENSG00000180423	HARBI1	filled
ENST00000532281	ENSG00000180423	HARBI1	
ENST00000450207	ENSG00000184164	CRELD2	
ENST00000461357	ENSG00000078596	ITM2A	
ENST00000606497	ENSG00000145495	6-Mar	filled
ENST00000394349	ENSG00000135390	ATP5G2	
ENST00000552242	ENSG00000135390	ATP5G2	
ENST00000549164	ENSG00000135390	ATP5G2	
ENST00000338662	ENSG00000135390	ATP5G2	
ENST00000552120	ENSG00000135390	ATP5G2	
ENST00000266581	ENSG00000139211	AMIGO2	
ENST00000374343	ENSG00000204178	TMEM57	filled
ENST00000399766	ENSG00000204178	TMEM57	filled
ENST00000398576	ENSG00000136167	LCP1	filled
ENST00000449645	ENSG00000185973	TMLHE	
ENST00000491547	ENSG00000189337	KAZN	filled
ENST00000376030	ENSG00000189337	KAZN	filled
ENST00000503743	ENSG00000189337	KAZN	filled
ENST00000361144	ENSG00000189337	KAZN	
ENST00000400797	ENSG00000189337	KAZN	
ENST00000400798	ENSG00000189337	KAZN	
ENST00000602185	ENSG00000130755	GMFG	
ENST00000547566	ENSG00000135486	HNRNPA1	
ENST00000547870	ENSG00000135486	HNRNPA1	
ENST00000551702	ENSG00000135486	HNRNPA1	
ENST00000548688	ENSG00000135486	HNRNPA1	
ENST00000436198	ENSG00000129682	FGF13	filled
ENST00000448673	ENSG00000129682	FGF13	filled
ENST00000427721	ENSG00000250644	RP11-295K3.1	filled
ENST00000518297	ENSG00000155506	LARP1	
ENST00000517616	ENSG00000155506	LARP1	
ENST00000369115	ENSG00000143401	ANP32E	
ENST00000534220	ENSG00000143401	ANP32E	
ENST00000409275	ENSG00000042493	CAPG	
ENST00000569638	ENSG00000099365	STX1B	
ENST00000451188	ENSG00000178233	TMEM151B	
ENST00000438774	ENSG00000178233	TMEM151B	filled
ENST00000581184	ENSG00000141434	MEP1B	filled
ENST00000381995	ENSG00000159128	IFNGR2	
ENST00000405436	ENSG00000159128	IFNGR2	
ENST00000613220	ENSG00000276537	TTYH1	
ENST00000612600	ENSG00000276537	TTYH1	
ENST00000615687	ENSG00000276537	TTYH1	
ENST00000470985	ENSG00000111716	LDHB	
ENST00000463287	ENSG00000184381	PLA2G6	
ENST00000594306	ENSG00000184381	PLA2G6	filled
ENST00000305641	ENSG00000168806	LCMT2	
ENST00000505490	ENSG00000183684	ALYREF	
ENST00000504015	ENSG00000183684	ALYREF	filled
ENST00000565555	ENSG00000168803	ADAL	
ENST00000502296	ENSG00000127022	CANX	
ENST00000504734	ENSG00000127022	CANX	
ENST00000247461	ENSG00000127022	CANX	
ENST00000452673	ENSG00000127022	CANX	
ENST00000383596	ENSG00000206492	GNL1	
ENST00000448773	ENSG00000018699	TTC27	
ENST00000435030	ENSG00000168280	KIF5C	filled
ENST00000435505	ENSG00000028116	VRK2	filled
ENST00000417641	ENSG00000028116	VRK2	filled

ENST00000535587	ENSG00000173262	SLC2A14	
ENST00000474874	ENSG00000116285	ERRFI1	filled
ENST00000497970	ENSG00000134253	TRIM45	
ENST00000555119	ENSG00000185046	ANKS1B	filled
ENST00000552245	ENSG00000185046	ANKS1B	filled
ENST00000389017	ENSG00000196544	C17orf59	
ENST00000264233	ENSG00000051341	POLQ	filled
ENST00000488282	ENSG00000051341	POLQ	
ENST00000621776	ENSG00000051341	POLQ	filled
ENST00000423741	ENSG00000214866	DCDC2C	filled
ENST00000399143	ENSG00000214866	DCDC2C	filled
ENST00000555003	ENSG00000137070	IL11RA	filled
ENST00000555247	ENSG00000137070	IL11RA	filled
ENST00000441545	ENSG00000137070	IL11RA	filled
ENST00000318041	ENSG00000137070	IL11RA	filled
ENST00000557298	ENSG00000137070	IL11RA	filled
ENST00000466082	ENSG00000137070	IL11RA	filled
ENST00000302101	ENSG00000171786	NHLH1	
ENST00000313143	ENSG00000161202	DVL3	filled
ENST00000423300	ENSG00000161202	DVL3	filled
ENST00000485704	ENSG00000148356	LRSAM1	
ENST00000603027	ENSG00000272916	RP11-574K11.31	filled
ENST00000603706	ENSG00000272916	RP11-574K11.31	
ENST00000594374	ENSG00000268361	L34079.2	filled
ENST00000572508	ENSG00000108379	WNT3	
ENST00000576471	ENSG00000108379	WNT3	
ENST00000393923	ENSG00000144824	PHLDB2	filled
ENST00000431670	ENSG00000144824	PHLDB2	filled
ENST00000498699	ENSG00000144824	PHLDB2	filled
ENST00000412622	ENSG00000144824	PHLDB2	filled
ENST00000225823	ENSG00000108684	ASIC2	filled
ENST00000359872	ENSG00000108684	ASIC2	filled
ENST00000579816	ENSG00000108684	ASIC2	
ENST00000583395	ENSG00000108684	ASIC2	
ENST00000445426	ENSG00000180834	MAP6D1	filled
ENST00000317802	ENSG00000178021	TSPYL6	
ENST00000449997	ENSG00000136279	DBNL	
ENST00000560444	ENSG00000140451	PIF1	filled
ENST00000369787	ENSG00000116396	KCNC4	
ENST00000412512	ENSG00000116396	KCNC4	
ENST00000375571	ENSG00000101367	MAPRE1	
ENST00000260045	ENSG00000137492	PRKRIR	
ENST00000528993	ENSG00000137492	PRKRIR	
ENST00000585955	ENSG00000168256	NKIRAS2	
ENST00000527343	ENSG00000110651	CD81	
ENST00000492179	ENSG00000205089	CCNI2	
ENST00000373296	ENSG00000122145	TBX22	
ENST00000295269	ENSG00000180251	SLC9A4	filled
ENST00000525057	ENSG00000110169	HPX	
ENST00000520690	ENSG00000104490	NCALD	filled
ENST00000518727	ENSG00000104490	NCALD	filled
ENST00000518166	ENSG00000104490	NCALD	filled
ENST00000517822	ENSG00000104490	NCALD	filled
ENST00000524209	ENSG00000104490	NCALD	filled
ENST00000521964	ENSG00000104490	NCALD	filled
ENST00000523923	ENSG00000104490	NCALD	filled
ENST00000517639	ENSG00000104490	NCALD	filled
ENST00000463327	ENSG00000171433	GLOD5	
ENST00000379089	ENSG00000164970	FAM219A	
ENST00000379087	ENSG00000164970	FAM219A	

ENST00000379084	ENSG00000164970	FAM219A	
ENST00000379081	ENSG00000164970	FAM219A	
ENST00000379080	ENSG00000164970	FAM219A	
ENST00000297620	ENSG00000164970	FAM219A	
ENST00000422409	ENSG00000164970	FAM219A	
ENST00000379078	ENSG00000164970	FAM219A	
ENST00000445726	ENSG00000164970	FAM219A	
ENST00000264433	ENSG00000052795	FNIP2	filled
ENST00000504704	ENSG00000052795	FNIP2	filled
ENST00000276077	ENSG00000147138	GPR174	
ENST00000086933	ENSG00000063515	GSC2	
ENST00000480583	ENSG00000177483	RBM44	filled
ENST00000539115	ENSG00000005889	ZFX	
ENST00000375031	ENSG00000189410	SH2D5	filled
ENST00000444387	ENSG00000189410	SH2D5	filled
ENST00000447746	ENSG00000189410	SH2D5	filled
ENST00000517430	ENSG00000189410	SH2D5	filled
ENST00000318245	ENSG00000178734	LMO7DN	
ENST00000467753	ENSG00000214338	SOGA3	
ENST00000334068	ENSG00000186971	KRTAP13-4	
ENST00000471286	ENSG00000158714	SLAMF8	
ENST00000497141	ENSG00000158714	SLAMF8	
ENST00000481444	ENSG00000111971	LY6G5C	
ENST00000484710	ENSG00000111971	LY6G5C	
ENST00000450798	ENSG00000111971	LY6G5C	
ENST00000392325	ENSG00000141580	WDR45B	
ENST00000572583	ENSG00000141580	WDR45B	
ENST00000573616	ENSG00000141580	WDR45B	
ENST00000571817	ENSG00000141580	WDR45B	
ENST00000414003	ENSG00000224103	HLA-DPA1	
ENST00000458578	ENSG00000185651	UBE2L3	filled
ENST00000493263	ENSG00000125834	STK35	filled
ENST00000322128	ENSG00000180543	TSPYL5	
ENST00000379485	ENSG00000165572	KBTBD6	
ENST00000512441	ENSG00000163683	SMIM14	
ENST00000511809	ENSG00000163683	SMIM14	
ENST00000510628	ENSG00000163683	SMIM14	
ENST00000507613	ENSG00000163683	SMIM14	
ENST00000339020	ENSG00000188725	SMIM15	
ENST00000455584	ENSG00000251537	RP11-385D13.1	filled
ENST00000443687	ENSG00000122691	TWIST1	filled
ENST00000242261	ENSG00000122691	TWIST1	
ENST00000404625	ENSG00000136244	IL6	filled
ENST00000380342	ENSG00000147003	TMEM27	filled
ENST00000583269	ENSG00000182481	KPNA2	
ENST00000584026	ENSG00000182481	KPNA2	
ENST00000582579	ENSG00000257008	GPR142	
ENST00000335666	ENSG00000257008	GPR142	
ENST00000310816	ENSG00000109339	MAPK10	filled
ENST00000361569	ENSG00000109339	MAPK10	filled
ENST00000513839	ENSG00000109339	MAPK10	filled
ENST00000511328	ENSG00000109339	MAPK10	filled
ENST00000502302	ENSG00000109339	MAPK10	filled
ENST00000503911	ENSG00000109339	MAPK10	filled
ENST00000513186	ENSG00000109339	MAPK10	filled
ENST00000504397	ENSG00000109339	MAPK10	filled
ENST00000512046	ENSG00000109339	MAPK10	filled
ENST00000496874	ENSG00000167930	ITFG3	
ENST00000445559	ENSG00000237335	PFDN6	
ENST00000491095	ENSG00000237335	PFDN6	

ENST00000288207	ENSG00000157456	CCNB2	filled
ENST00000561077	ENSG00000157456	CCNB2	filled
ENST00000559622	ENSG00000157456	CCNB2	filled
ENST00000559301	ENSG00000157456	CCNB2	
ENST00000621385	ENSG00000157456	CCNB2	filled
ENST00000370323	ENSG00000124260	MAGEA10	
ENST00000244096	ENSG00000124260	MAGEA10	
ENST00000469124	ENSG00000126561	STAT5A	
ENST00000444283	ENSG00000126561	STAT5A	
ENST00000576040	ENSG00000073969	NSF	filled
ENST00000451246	ENSG00000180822	PSMG4	filled
ENST00000391971	ENSG00000168385	2-Sep	
ENST00000407971	ENSG00000168385	2-Sep	
ENST00000421717	ENSG00000168385	2-Sep	
ENST00000519915	ENSG00000272295	DAQB-331I12.15	filled
ENST00000545614	ENSG00000257065	RP3-468K18.5	filled
ENST00000590169	ENSG00000087152	ATXN7L3	
ENST00000589805	ENSG00000087152	ATXN7L3	
ENST00000286827	ENSG00000156299	TIAM1	filled
ENST00000469412	ENSG00000156299	TIAM1	filled
ENST00000541036	ENSG00000156299	TIAM1	filled
ENST00000589655	ENSG00000105514	RAB3D	
ENST00000351325	ENSG00000266964	FXYD1	
ENST00000589209	ENSG00000266964	FXYD1	
ENST00000588081	ENSG00000266964	FXYD1	
ENST00000589121	ENSG00000266964	FXYD1	
ENST00000590462	ENSG00000266964	FXYD1	
ENST00000612146	ENSG00000266964	FXYD1	
ENST00000504100	ENSG00000138792	ENPEP	
ENST00000315769	ENSG00000165061	ZMAT4	filled
ENST00000522623	ENSG00000165061	ZMAT4	filled
ENST00000463155	ENSG00000054277	OPN3	filled
ENST00000406486	ENSG00000197077	KIAA1671	filled
ENST00000461783	ENSG00000146426	TIAM2	filled
ENST00000535064	ENSG00000146426	TIAM2	filled
ENST00000460692	ENSG00000146426	TIAM2	
ENST00000449545	ENSG00000146426	TIAM2	
ENST00000597186	ENSG00000181894	ZNF329	
ENST00000451311	ENSG00000205542	TMSB4X	
ENST00000229003	ENSG00000111405	ENDOU	filled
ENST00000422538	ENSG00000111405	ENDOU	filled
ENST00000545824	ENSG00000111405	ENDOU	filled
ENST00000480130	ENSG00000119927	GPAM	
ENST00000424834	ENSG00000182957	SPATA13	filled
ENST00000588968	ENSG00000161533	ACOX1	filled
ENST00000587927	ENSG00000161533	ACOX1	filled
ENST00000486112	ENSG00000230678	BRD2	
ENST00000579261	ENSG00000011260	UTP18	
ENST00000540180	ENSG00000120647	CCDC77	
ENST00000339444	ENSG00000170615	SLC26A5	filled
ENST00000393735	ENSG00000170615	SLC26A5	
ENST00000306312	ENSG00000170615	SLC26A5	filled
ENST00000487407	ENSG00000170615	SLC26A5	
ENST00000432958	ENSG00000170615	SLC26A5	filled
ENST00000354356	ENSG00000170615	SLC26A5	filled
ENST00000573224	ENSG00000108433	GOSR2	
ENST00000555216	ENSG00000012983	MAP4K5	filled
ENST00000582520	ENSG00000141522	ARHGDIA	
ENST00000269321	ENSG00000141522	ARHGDIA	
ENST00000400721	ENSG00000141522	ARHGDIA	

ENST00000584461	ENSG00000141522	ARHGDIA	
ENST00000541078	ENSG00000141522	ARHGDIA	
ENST00000580685	ENSG00000141522	ARHGDIA	
ENST00000579121	ENSG00000141522	ARHGDIA	
ENST00000581876	ENSG00000141522	ARHGDIA	
ENST00000583868	ENSG00000141522	ARHGDIA	
ENST00000316673	ENSG00000101076	HNF4A	filled
ENST00000609262	ENSG00000101076	HNF4A	filled
ENST00000619550	ENSG00000101076	HNF4A	filled
ENST00000546760	ENSG00000258315	C17orf49	
ENST00000549498	ENSG00000111077	TENC1	filled
ENST00000475243	ENSG00000124164	VAPB	filled
ENST00000395802	ENSG00000124164	VAPB	filled
ENST00000265619	ENSG00000124164	VAPB	filled
ENST00000569262	ENSG00000103196	CRISPLD2	
ENST00000589780	ENSG00000225190	PLEKHM1	filled
ENST00000586562	ENSG00000225190	PLEKHM1	filled
ENST00000586084	ENSG00000225190	PLEKHM1	filled
ENST00000389982	ENSG00000182896	TMEM95	
ENST00000330767	ENSG00000182896	TMEM95	
ENST00000519274	ENSG00000147364	FBXO25	
ENST00000414316	ENSG00000100129	EIF3L	filled
ENST00000436452	ENSG00000100129	EIF3L	filled
ENST00000375898	ENSG00000139826	ABHD13	filled
ENST00000543740	ENSG00000204954	C12orf73	
ENST00000553183	ENSG00000204954	C12orf73	
ENST00000580358	ENSG00000262068	KRTAP3-1	
ENST00000376006	ENSG00000122824	NUDT10	filled
ENST00000356450	ENSG00000122824	NUDT10	filled
ENST00000322357	ENSG00000178951	ZBTB7A	
ENST00000486377	ENSG00000114125	RNF7	
ENST00000526811	ENSG00000142089	IFITM3	filled
ENST00000442788	ENSG00000256646	PSMA2	
ENST00000433579	ENSG00000256646	PSMA2	
ENST00000343837	ENSG00000188817	SNTN	filled
ENST00000590239	ENSG00000123136	DDX39A	
ENST00000591275	ENSG00000123136	DDX39A	
ENST00000590696	ENSG00000123136	DDX39A	
ENST00000410026	ENSG00000173559	NABP1	
ENST00000380445	ENSG00000215305	VPS16	filled
ENST00000380469	ENSG00000215305	VPS16	filled
ENST00000453689	ENSG00000215305	VPS16	filled
ENST00000417508	ENSG00000215305	VPS16	filled
ENST00000303635	ENSG00000171735	CAMTA1	filled
ENST00000461311	ENSG00000171735	CAMTA1	filled
ENST00000482934	ENSG00000171735	CAMTA1	filled
ENST00000470648	ENSG00000171735	CAMTA1	
ENST00000461580	ENSG00000171735	CAMTA1	
ENST00000467267	ENSG00000171735	CAMTA1	
ENST00000409874	ENSG00000156414	TDRD9	filled
ENST00000526246	ENSG00000174791	RIN1	
ENST00000547943	ENSG00000110871	COQ5	
ENST00000551769	ENSG00000110871	COQ5	
ENST00000624552	ENSG00000148357	HMCN2	filled
ENST00000573845	ENSG00000141086	CTRL	
ENST00000357175	ENSG00000157502	MUM1L1	
ENST00000372552	ENSG00000157502	MUM1L1	filled
ENST00000337685	ENSG00000157502	MUM1L1	filled
ENST00000619455	ENSG00000157502	MUM1L1	
ENST00000345097	ENSG00000053254	FOXN3	filled
ENST00000555353	ENSG00000053254	FOXN3	filled
ENST00000555855	ENSG00000053254	FOXN3	filled
ENST00000555034	ENSG00000053254	FOXN3	filled
ENST00000553904	ENSG00000053254	FOXN3	filled
ENST00000619852	ENSG00000275004	ZNF280B	
ENST00000613655	ENSG00000275004	ZNF280B	
ENST00000337714	ENSG00000121057	AKAP1	filled
ENST00000571629	ENSG00000121057	AKAP1	filled
ENST00000575186	ENSG00000121057	AKAP1	
ENST00000573085	ENSG00000121057	AKAP1	
ENST00000572814	ENSG00000121057	AKAP1	
ENST00000574683	ENSG00000121057	AKAP1	
ENST00000572557	ENSG00000121057	AKAP1	filled
ENST00000481416	ENSG00000121057	AKAP1	filled
ENST00000539273	ENSG00000121057	AKAP1	filled
ENST00000576591	ENSG00000121057	AKAP1	
ENST00000621116	ENSG00000121057	AKAP1	filled
ENST00000314126	ENSG00000121057	AKAP1	
ENST00000295408	ENSG00000153208	MERTK	filled
ENST00000409780	ENSG00000153208	MERTK	filled
ENST00000439966	ENSG00000153208	MERTK	filled
ENST00000421804	ENSG00000153208	MERTK	filled
ENST00000493274	ENSG00000232119	MCTS1	
ENST00000461557	ENSG00000117395	EBNA1BP2	filled
ENST00000410117	ENSG00000115935	WIPF1	filled
ENST00000480400	ENSG00000115935	WIPF1	filled
ENST00000470752	ENSG00000115935	WIPF1	
ENST00000490887	ENSG00000115935	WIPF1	
ENST00000469002	ENSG00000115935	WIPF1	
ENST00000487291	ENSG00000115935	WIPF1	
ENST00000562767	ENSG00000260007	RP11-315D16.2	
ENST00000446974	ENSG00000096070	BRPF3	
ENST00000454960	ENSG00000096070	BRPF3	
ENST00000527515	ENSG00000177570	SAMD12	
ENST00000524555	ENSG00000255071	SAA2-SAA4	
ENST00000515646	ENSG00000173011	TADA2B	
ENST00000514191	ENSG00000119599	DCAF4	filled
ENST00000505361	ENSG00000119599	DCAF4	filled
ENST00000513337	ENSG00000119599	DCAF4	
ENST00000557203	ENSG00000119599	DCAF4	
ENST00000332129	ENSG00000116852	KIF21B	
ENST00000461742	ENSG00000116852	KIF21B	
ENST00000360529	ENSG00000116852	KIF21B	
ENST00000422435	ENSG00000116852	KIF21B	
ENST00000315289	ENSG00000182010	RTKN2	filled
ENST00000233630	ENSG00000115289	PCGF1	
ENST00000218249	ENSG00000102128	RAB40AL	
ENST00000503860	ENSG00000169248	CXCL11	
ENST00000320256	ENSG00000244617	ASPRV1	
ENST00000437811	ENSG00000231389	HLA-DPA1	
ENST00000489603	ENSG00000124831	LRRFIP1	filled
ENST00000298159	ENSG00000165410	CFL2	
ENST00000266594	ENSG00000139223	ANP32D	
ENST00000314218	ENSG00000181004	BBS12	
ENST00000433287	ENSG00000181004	BBS12	
ENST00000542236	ENSG00000181004	BBS12	
ENST00000596851	ENSG00000142303	ADAMTS10	filled
ENST00000596709	ENSG00000142303	ADAMTS10	filled
ENST00000596466	ENSG00000142303	ADAMTS10	filled
ENST00000593534	ENSG00000142303	ADAMTS10	

ENST00000542367	ENSG00000110092	CCND1	
ENST00000589018	ENSG00000120784	ZFP30	
ENST00000443879	ENSG00000163812	ZDHHC3	
ENST00000461414	ENSG00000122194	PLG	
ENST00000322038	ENSG00000179981	TSHZ1	
ENST00000560918	ENSG00000179981	TSHZ1	filled
ENST00000560661	ENSG00000179981	TSHZ1	filled
ENST00000584217	ENSG00000179981	TSHZ1	
ENST00000356317	ENSG00000139629	GALNT6	filled
ENST00000604847	ENSG00000139629	GALNT6	filled
ENST00000604426	ENSG00000139629	GALNT6	filled
ENST00000603563	ENSG00000139629	GALNT6	filled
ENST00000605367	ENSG00000139629	GALNT6	filled
ENST00000605720	ENSG00000139629	GALNT6	filled
ENST00000558992	ENSG00000172183	ISG20	
ENST00000371493	ENSG00000164402	8-Sep	
ENST00000492227	ENSG00000197323	TRIM33	
ENST00000484376	ENSG00000176919	C8G	
ENST00000465773	ENSG00000176919	C8G	
ENST00000621775	ENSG00000273820	USP27X	
ENST00000536837	ENSG00000089053	ANAPC5	
ENST00000410059	ENSG00000175497	DPP10	filled
ENST00000409163	ENSG00000175497	DPP10	filled
ENST00000455903	ENSG00000155636	RBM45	
ENST00000464647	ENSG00000155636	RBM45	
ENST00000494118	ENSG00000151789	ZNF385D	filled
ENST00000494108	ENSG00000151789	ZNF385D	filled
ENST00000474607	ENSG00000151789	ZNF385D	filled
ENST00000252971	ENSG00000130675	MNX1	filled
ENST00000402441	ENSG00000183023	SLC8A1	filled
ENST00000405269	ENSG00000183023	SLC8A1	filled
ENST00000455476	ENSG00000183023	SLC8A1	filled
ENST00000448531	ENSG00000183023	SLC8A1	filled
ENST00000417271	ENSG00000183023	SLC8A1	filled
ENST00000491064	ENSG00000144579	CTDSP1	
ENST00000443891	ENSG00000144579	CTDSP1	
ENST00000492545	ENSG00000144579	CTDSP1	
ENST00000473420	ENSG00000144579	CTDSP1	
ENST00000585493	ENSG00000198551	ZNF627	
ENST00000588651	ENSG00000198551	ZNF627	
ENST00000361113	ENSG00000198551	ZNF627	
ENST00000588174	ENSG00000198551	ZNF627	
ENST00000523674	ENSG00000104408	EIF3E	
ENST00000522088	ENSG00000104408	EIF3E	filled
ENST00000423292	ENSG00000188086	PRSS45	
ENST00000549151	ENSG00000079337	RAPGEF3	filled
ENST00000548919	ENSG00000079337	RAPGEF3	filled
ENST00000530059	ENSG00000157211	CDCP2	
ENST00000401647	ENSG00000175600	SUGCT	filled
ENST00000335693	ENSG00000175600	SUGCT	filled
ENST00000460466	ENSG00000175600	SUGCT	filled
ENST00000509364	ENSG00000187678	SPRY4	
ENST00000515463	ENSG00000153037	SRP19	
ENST00000315033	ENSG00000181656	GPR88	
ENST00000589239	ENSG00000132005	RFX1	filled
ENST00000588885	ENSG00000132005	RFX1	filled
ENST00000269385	ENSG00000141570	CBX8	
ENST00000427800	ENSG00000141570	CBX8	
ENST00000485449	ENSG00000141570	CBX8	
ENST00000558928	ENSG00000166813	KIF7	

ENST00000473006	ENSG00000137285	TUBB2B	
ENST00000380367	ENSG00000182070	OR52A1	
ENST00000475614	ENSG00000186409	CCDC30	filled
ENST00000477155	ENSG00000186409	CCDC30	filled
ENST00000495044	ENSG00000186409	CCDC30	filled
ENST00000507855	ENSG00000186409	CCDC30	filled
ENST00000509712	ENSG00000186409	CCDC30	filled
ENST00000428554	ENSG00000186409	CCDC30	filled
ENST00000266126	ENSG00000119718	EIF2B2	filled
ENST00000376495	ENSG00000165312	OTUD1	
ENST00000622112	ENSG00000149646	CNBD2	filled
ENST00000451997	ENSG00000100092	SH3BP1	filled
ENST00000469947	ENSG00000100092	SH3BP1	filled
ENST00000412289	ENSG00000224782	PFDN6	
ENST00000474406	ENSG00000224782	PFDN6	
ENST00000423054	ENSG00000155265	GOLGA7B	
ENST00000478113	ENSG00000129007	CALML4	
ENST00000395465	ENSG00000129007	CALML4	
ENST00000540479	ENSG00000129007	CALML4	
ENST00000452582	ENSG00000162961	DPY30	
ENST00000414013	ENSG00000162961	DPY30	
ENST00000482018	ENSG00000121769	FABP3	
ENST00000342983	ENSG00000123728	RAP2C	filled
ENST00000620646	ENSG00000123728	RAP2C	filled
ENST00000526757	ENSG00000166444	ST5	filled
ENST00000534127	ENSG00000166444	ST5	filled
ENST00000313726	ENSG00000166444	ST5	filled
ENST00000532734	ENSG00000166444	ST5	filled
ENST00000526057	ENSG00000166444	ST5	filled
ENST00000528196	ENSG00000166444	ST5	filled
ENST00000530580	ENSG00000166444	ST5	filled
ENST00000532651	ENSG00000166444	ST5	filled
ENST00000531093	ENSG00000166444	ST5	filled
ENST00000533225	ENSG00000166444	ST5	filled
ENST00000526126	ENSG00000166444	ST5	filled
ENST00000529940	ENSG00000166444	ST5	filled
ENST00000528523	ENSG00000166444	ST5	
ENST00000533681	ENSG00000166444	ST5	filled
ENST00000526241	ENSG00000166444	ST5	filled
ENST00000533580	ENSG00000166444	ST5	filled
ENST00000526155	ENSG00000166444	ST5	filled
ENST00000534248	ENSG00000166444	ST5	filled
ENST00000527347	ENSG00000166444	ST5	filled
ENST00000533016	ENSG00000166444	ST5	filled
ENST00000527516	ENSG00000166444	ST5	filled
ENST00000527473	ENSG00000166444	ST5	filled
ENST00000533471	ENSG00000166444	ST5	filled
ENST00000524757	ENSG00000166444	ST5	filled
ENST00000527392	ENSG00000166444	ST5	filled
ENST00000526828	ENSG00000166444	ST5	filled
ENST00000525169	ENSG00000166444	ST5	filled
ENST00000534665	ENSG00000166444	ST5	filled
ENST00000531578	ENSG00000166444	ST5	filled
ENST00000357665	ENSG00000166444	ST5	filled
ENST00000477089	ENSG00000067369	TP53BP1	filled
ENST00000509400	ENSG00000120725	SIL1	filled
ENST00000377698	ENSG00000137124	ALDH1B1	
ENST00000563387	ENSG00000169609	C15orf40	
ENST00000512091	ENSG00000150471	LPHN3	filled
ENST00000514591	ENSG00000150471	LPHN3	filled

ENST00000509896	ENSG00000150471	LPHN3	filled
ENST00000511324	ENSG00000150471	LPHN3	filled
ENST00000506700	ENSG00000150471	LPHN3	filled
ENST00000507164	ENSG00000150471	LPHN3	filled
ENST00000508693	ENSG00000150471	LPHN3	filled
ENST00000507625	ENSG00000150471	LPHN3	filled
ENST00000514157	ENSG00000150471	LPHN3	filled
ENST00000504896	ENSG00000150471	LPHN3	filled
ENST00000506720	ENSG00000150471	LPHN3	filled
ENST00000506746	ENSG00000150471	LPHN3	filled
ENST00000509089	ENSG00000150471	LPHN3	filled
ENST00000587521	ENSG00000105220	GPI	filled
ENST00000592277	ENSG00000105220	GPI	filled
ENST00000495135	ENSG00000142920	AZIN2	
ENST00000322002	ENSG00000176887	SOX11	
ENST00000521452	ENSG00000253304	TMEM200B	
ENST00000420504	ENSG00000253304	TMEM200B	
ENST00000523023	ENSG00000104695	PPP2CB	
ENST00000586764	ENSG00000175832	ETV4	filled
ENST00000555145	ENSG00000066629	EML1	filled
ENST00000513488	ENSG00000198740	ZNF652	
ENST00000299178	ENSG00000166148	AVPR1A	
ENST00000475304	ENSG00000231925	TAPBP	
ENST00000509970	ENSG00000163319	MRPS18C	
ENST00000509571	ENSG00000163319	MRPS18C	
ENST00000381020	ENSG00000165556	CDX2	filled
ENST00000367734	ENSG00000177468	OLIG3	
ENST00000477428	ENSG00000106459	NRF1	
ENST00000223190	ENSG00000106459	NRF1	filled
ENST00000311967	ENSG00000106459	NRF1	filled
ENST00000393230	ENSG00000106459	NRF1	filled
ENST00000393232	ENSG00000106459	NRF1	filled
ENST00000353868	ENSG00000106459	NRF1	filled
ENST00000465077	ENSG00000183570	PCBP3	filled
ENST00000400314	ENSG00000183570	PCBP3	filled
ENST00000472191	ENSG00000183570	PCBP3	
ENST00000400310	ENSG00000183570	PCBP3	filled
ENST00000400309	ENSG00000183570	PCBP3	filled
ENST00000400308	ENSG00000183570	PCBP3	filled
ENST00000449640	ENSG00000183570	PCBP3	filled
ENST00000453381	ENSG00000254899	DASS-161H22.6	filled
ENST00000607564	ENSG00000161939	RNASEK-C17orf49	
ENST00000378700	ENSG00000172201	ID4	filled
ENST00000618493	ENSG00000172201	ID4	filled
ENST00000277905	ENSG00000148704	VAX1	
ENST00000529049	ENSG00000166689	PLEKHA7	
ENST00000533901	ENSG00000166689	PLEKHA7	
ENST00000382141	ENSG00000273167	RP11-307N16.6	filled
ENST00000360295	ENSG00000063015	SEZ6	filled
ENST00000317338	ENSG00000063015	SEZ6	filled
ENST00000442608	ENSG00000063015	SEZ6	filled
ENST00000585644	ENSG00000063015	SEZ6	
ENST00000335960	ENSG00000063015	SEZ6	filled
ENST00000493152	ENSG00000136143	SUCLA2	filled
ENST00000417784	ENSG00000175193	PARL	
ENST00000449306	ENSG00000175193	PARL	
ENST00000615922	ENSG00000163378	EOGT	
ENST00000561648	ENSG00000102935	ZNF423	filled
ENST00000563137	ENSG00000102935	ZNF423	filled
ENST00000562520	ENSG00000102935	ZNF423	filled

ENST00000262383	ENSG00000102935	ZNF423	filled
ENST00000302548	ENSG00000171657	GPR82	
ENST00000497180	ENSG00000171657	GPR82	
ENST00000428025	ENSG00000171453	POLR1C	filled
ENST00000492679	ENSG00000135253	KCP	
ENST00000521180	ENSG00000008513	ST3GAL1	filled
ENST00000522652	ENSG00000008513	ST3GAL1	filled
ENST00000523854	ENSG00000008513	ST3GAL1	filled
ENST00000517668	ENSG00000008513	ST3GAL1	filled
ENST00000523634	ENSG00000008513	ST3GAL1	
ENST00000519924	ENSG00000008513	ST3GAL1	
ENST00000523855	ENSG00000008513	ST3GAL1	
ENST00000519435	ENSG00000008513	ST3GAL1	filled
ENST00000518298	ENSG00000008513	ST3GAL1	filled
ENST00000520020	ENSG00000008513	ST3GAL1	
ENST00000522873	ENSG00000008513	ST3GAL1	filled
ENST00000399640	ENSG00000008513	ST3GAL1	filled
ENST00000405846	ENSG00000132975	GPR12	filled
ENST00000495546	ENSG00000187699	C2orf88	filled
ENST00000490033	ENSG00000187699	C2orf88	filled
ENST00000396974	ENSG00000187699	C2orf88	filled
ENST00000409545	ENSG00000187699	C2orf88	
ENST00000456318	ENSG00000163882	POLR2H	
ENST00000438240	ENSG00000163882	POLR2H	
ENST00000460083	ENSG00000163882	POLR2H	
ENST00000317775	ENSG00000089250	NOS1	filled
ENST00000618760	ENSG00000089250	NOS1	filled
ENST00000491573	ENSG00000124608	AARS2	
ENST00000357985	ENSG00000183844	FAM3B	
ENST00000398646	ENSG00000183844	FAM3B	
ENST00000323703	ENSG00000163795	ZNF513	
ENST00000436006	ENSG00000163795	ZNF513	
ENST00000265538	ENSG00000067560	RHOA	
ENST00000404433	ENSG00000163793	DNAJC5G	
ENST00000543580	ENSG00000166073	GPR176	
ENST00000361226	ENSG00000198853	RUSC2	filled
ENST00000477171	ENSG00000152601	MBNL1	
ENST00000373448	ENSG00000101407	TTI1	filled
ENST00000417253	ENSG00000198265	HELZ	filled
ENST00000592360	ENSG00000256294	ZNF225	filled
ENST00000547832	ENSG00000139644	TMBIM6	
ENST00000547187	ENSG00000139644	TMBIM6	
ENST00000552699	ENSG00000139644	TMBIM6	
ENST00000549385	ENSG00000139644	TMBIM6	
ENST00000550445	ENSG00000139644	TMBIM6	
ENST00000423828	ENSG00000139644	TMBIM6	
ENST00000341267	ENSG00000185559	DLK1	
ENST00000224721	ENSG00000107736	CDH23	filled
ENST00000622827	ENSG00000107736	CDH23	filled
ENST00000374392	ENSG00000184454	NCMAP	
ENST00000486262	ENSG00000184454	NCMAP	
ENST00000579439	ENSG00000198844	ARHGEF15	
ENST00000498697	ENSG00000182329	KIAA2012	filled
ENST00000541917	ENSG00000182329	KIAA2012	filled
ENST00000277415	ENSG00000130558	OLFM1	filled
ENST00000510997	ENSG00000136436	CALCOCO2	
ENST00000543233	ENSG00000179242	CDH4	filled
ENST00000590753	ENSG00000108861	DUSP3	
ENST00000378719	ENSG00000164402	8-Sep	
ENST00000490034	ENSG00000117245	KIF17	

ENST00000375044	ENSG00000117245	KIF17	filled
ENST00000400463	ENSG00000117245	KIF17	filled
ENST00000247986	ENSG00000117245	KIF17	filled
ENST00000414546	ENSG00000134339	SAA2	
ENST00000530400	ENSG00000134339	SAA2	
ENST00000508053	ENSG00000138829	FBN2	filled
ENST00000230859	ENSG00000112941	PAPD7	
ENST00000602657	ENSG00000213523	SRA1	
ENST00000423313	ENSG00000235750	KIAA0040	
ENST00000444639	ENSG00000235750	KIAA0040	filled
ENST00000545251	ENSG00000235750	KIAA0040	
ENST00000619513	ENSG00000235750	KIAA0040	
ENST00000454747	ENSG00000119335	SET	
ENST00000372692	ENSG00000119335	SET	
ENST00000409104	ENSG00000119335	SET	
ENST00000322030	ENSG00000119335	SET	
ENST00000372686	ENSG00000119335	SET	
ENST00000466009	ENSG00000119335	SET	
ENST00000477806	ENSG00000119335	SET	
ENST00000480217	ENSG00000119335	SET	
ENST00000480536	ENSG00000119335	SET	
ENST00000523638	ENSG00000254004	ZNF260	filled
ENST00000592282	ENSG00000254004	ZNF260	filled
ENST00000409883	ENSG00000155755	TMEM237	
ENST00000495329	ENSG00000155755	TMEM237	
ENST00000455734	ENSG00000136931	NR5A1	
ENST00000379491	ENSG00000124827	GCM2	filled
ENST00000527029	ENSG00000166199	ALKBH3	
ENST00000534171	ENSG00000166199	ALKBH3	
ENST00000524742	ENSG00000166199	ALKBH3	
ENST00000281317	ENSG00000151611	MMAA	
ENST00000506919	ENSG00000151611	MMAA	
ENST00000497341	ENSG00000115194	SLC30A3	
ENST00000409223	ENSG00000168427	KLHL30	filled
ENST00000395089	ENSG00000162620	LRRIQ3	filled
ENST00000415760	ENSG00000162620	LRRIQ3	filled
ENST00000468759	ENSG00000162620	LRRIQ3	filled
ENST00000444984	ENSG00000162620	LRRIQ3	
ENST00000354431	ENSG00000162620	LRRIQ3	filled
ENST00000496970	ENSG00000214595	EML6	
ENST00000319194	ENSG00000166938	DIS3L	filled
ENST00000319212	ENSG00000166938	DIS3L	filled
ENST00000530537	ENSG00000166938	DIS3L	filled
ENST00000370548	ENSG00000267561	RP5-1052I5.2	filled
ENST00000341259	ENSG00000111252	SH2B3	filled
ENST00000462702	ENSG00000085449	WDFY1	filled
ENST00000479284	ENSG00000177679	SRRM3	
ENST00000612155	ENSG00000177679	SRRM3	
ENST00000556551	ENSG00000258653	RP5-1021I20.4	filled
ENST00000618318	ENSG00000101413	RPRD1B	filled
ENST00000446558	ENSG00000197756	RPL37A	filled
ENST00000460670	ENSG00000161298	ZNF382	
ENST00000463910	ENSG00000161298	ZNF382	
ENST00000434573	ENSG00000110066	SUV420H1	
ENST00000617982	ENSG00000277600	CNOT3	filled
ENST00000612029	ENSG00000277600	CNOT3	filled
ENST00000379800	ENSG00000180138	CSNK1A1L	
ENST00000555280	ENSG00000178974	FBXO34	
ENST00000431984	ENSG00000183166	CALN1	filled
ENST00000457514	ENSG00000237353	PATE4	

ENST00000494101	ENSG00000156860	FBRS	
ENST00000399529	ENSG00000215077	BRD2	
ENST00000484278	ENSG00000215077	BRD2	
ENST00000601093	ENSG00000104960	PTOV1	
ENST00000604629	ENSG00000271271	UGT2A2	filled
ENST00000604021	ENSG00000271271	UGT2A2	filled
ENST00000306243	ENSG00000169105	CHST14	
ENST00000335071	ENSG00000186790	FOXE3	
ENST00000371884	ENSG00000162367	TAL1	
ENST00000294339	ENSG00000162367	TAL1	
ENST00000481091	ENSG00000162367	TAL1	
ENST00000493396	ENSG00000181744	C3orf58	filled
ENST00000409429	ENSG00000115325	DOK1	
ENST00000485132	ENSG00000115325	DOK1	
ENST00000377110	ENSG00000083067	TRPM3	filled
ENST00000377111	ENSG00000083067	TRPM3	filled
ENST00000357533	ENSG00000083067	TRPM3	filled
ENST00000354500	ENSG00000083067	TRPM3	filled
ENST00000366630	ENSG00000116991	SIPA1L2	
ENST00000393934	ENSG00000240891	PLCXD2	filled
ENST00000477665	ENSG00000240891	PLCXD2	filled
ENST00000538204	ENSG00000076770	MBNL3	filled
ENST00000394311	ENSG00000076770	MBNL3	filled
ENST00000286604	ENSG00000173610	UGT2A1	filled
ENST00000505512	ENSG00000173610	UGT2A1	
ENST00000561204	ENSG00000105419	MEIS3	filled
ENST00000560245	ENSG00000105419	MEIS3	filled
ENST00000561096	ENSG00000105419	MEIS3	filled
ENST00000560253	ENSG00000105419	MEIS3	filled
ENST00000558555	ENSG00000105419	MEIS3	filled
ENST00000294829	ENSG00000162771	FAM71A	
ENST00000361727	ENSG00000174469	CNTNAP2	filled
ENST00000455301	ENSG00000174469	CNTNAP2	filled
ENST00000556733	ENSG00000165792	METTL17	
ENST00000591818	ENSG00000079805	DNM2	
ENST00000274605	ENSG00000145911	N4BP3	
ENST00000600981	ENSG00000268714	CTD-2287O16.3	
ENST00000591145	ENSG00000141664	ZCCHC2	
ENST00000518814	ENSG00000127743	IL17B	
ENST00000490386	ENSG00000163815	CLEC3B	filled
ENST00000273153	ENSG00000144655	CSRNP1	
ENST00000514182	ENSG00000144655	CSRNP1	
ENST00000394735	ENSG00000138785	INTS12	
ENST00000451321	ENSG00000138785	INTS12	
ENST00000416543	ENSG00000138785	INTS12	
ENST00000433009	ENSG00000138785	INTS12	
ENST00000510876	ENSG00000138785	INTS12	filled
ENST00000515819	ENSG00000138785	INTS12	
ENST00000261749	ENSG00000086666	ZFAND6	filled
ENST00000561060	ENSG00000086666	ZFAND6	filled
ENST00000559157	ENSG00000086666	ZFAND6	filled
ENST00000561012	ENSG00000086666	ZFAND6	filled
ENST00000560470	ENSG00000086666	ZFAND6	filled
ENST00000613266	ENSG00000086666	ZFAND6	filled
ENST00000608736	ENSG00000125841	NRSN2	filled
ENST00000539664	ENSG00000255690	TRIL	
ENST00000511032	ENSG00000228716	DHFR	
ENST00000504396	ENSG00000228716	DHFR	
ENST00000513048	ENSG00000228716	DHFR	
ENST00000412264	ENSG00000155657	TTN	

ENST00000504780	ENSG00000013375	PGM3	
ENST00000539839	ENSG00000111432	FZD10	
ENST00000229030	ENSG00000111432	FZD10	
ENST00000463747	ENSG00000136240	KDELR2	
ENST00000343666	ENSG00000187713	TMEM203	
ENST00000570112	ENSG00000179455	MKRN3	
ENST00000564592	ENSG00000179455	MKRN3	
ENST00000588564	ENSG00000108309	RUNDC3A	
ENST00000416431	ENSG00000146215	CRIP3	
ENST00000505618	ENSG00000168421	RHOH	
ENST00000503941	ENSG00000168421	RHOH	
ENST00000381799	ENSG00000168421	RHOH	
ENST00000513894	ENSG00000168421	RHOH	
ENST00000515702	ENSG00000168421	RHOH	
ENST00000515718	ENSG00000168421	RHOH	
ENST00000515503	ENSG00000168421	RHOH	
ENST00000514143	ENSG00000168421	RHOH	
ENST00000504638	ENSG00000168421	RHOH	
ENST00000503978	ENSG00000168421	RHOH	
ENST00000615577	ENSG00000168421	RHOH	
ENST00000617441	ENSG00000168421	RHOH	
ENST00000622175	ENSG00000168421	RHOH	
ENST00000619474	ENSG00000168421	RHOH	
ENST00000615083	ENSG00000168421	RHOH	
ENST00000610353	ENSG00000168421	RHOH	
ENST00000614836	ENSG00000168421	RHOH	
ENST00000481764	ENSG00000187736	NHEJ1	
ENST00000403733	ENSG00000151718	WWC2	filled
ENST00000422489	ENSG00000093000	NUP50	
ENST00000570539	ENSG00000167978	SRRM2	
ENST00000619904	ENSG00000206190	ATP10A	
ENST00000439510	ENSG00000179604	CDC42EP4	
ENST00000399385	ENSG00000206283	PFDN6	
ENST00000482292	ENSG00000206283	PFDN6	
ENST00000507065	ENSG00000164393	GPR111	
ENST00000296862	ENSG00000164393	GPR111	
ENST00000467205	ENSG00000164393	GPR111	filled
ENST00000590846	ENSG00000101746	NOL4	
ENST00000588355	ENSG00000101746	NOL4	
ENST00000609256	ENSG00000180535	BHLHA15	
ENST00000537592	ENSG00000256463	SALL3	
ENST00000572928	ENSG00000256463	SALL3	
ENST00000278302	ENSG00000121236	TRIM6	filled
ENST00000380097	ENSG00000121236	TRIM6	filled
ENST00000445329	ENSG00000121236	TRIM6	filled
ENST00000515022	ENSG00000121236	TRIM6	filled
ENST00000506134	ENSG00000121236	TRIM6	filled
ENST00000618326	ENSG00000121236	TRIM6	filled
ENST00000528457	ENSG00000134744	ZCCHC11	filled
ENST00000527941	ENSG00000134744	ZCCHC11	filled
ENST00000469810	ENSG00000134744	ZCCHC11	filled
ENST00000528642	ENSG00000134744	ZCCHC11	filled
ENST00000484723	ENSG00000134744	ZCCHC11	filled
ENST00000371541	ENSG00000134744	ZCCHC11	filled
ENST00000473856	ENSG00000134744	ZCCHC11	filled
ENST00000343366	ENSG00000187492	CDHR4	filled
ENST00000487256	ENSG00000187492	CDHR4	filled
ENST00000420470	ENSG00000236699	ARHGEF38	filled
ENST00000503289	ENSG00000236699	ARHGEF38	
ENST00000508961	ENSG00000236699	ARHGEF38	

ENST00000299441	ENSG00000166341	DCHS1	filled
ENST00000511828	ENSG00000248713	RP11-766F14.2	
ENST00000508247	ENSG00000040275	SPDL1	
ENST00000512028	ENSG00000040275	SPDL1	
ENST00000388913	ENSG00000180921	FAM83H	
ENST00000395103	ENSG00000180921	FAM83H	
ENST00000560036	ENSG00000166415	WDR72	filled
ENST00000380274	ENSG00000145945	FAM50B	
ENST00000380272	ENSG00000145945	FAM50B	
ENST00000594099	ENSG00000132002	DNAJB1	filled
ENST00000601533	ENSG00000132002	DNAJB1	filled
ENST00000598692	ENSG00000132002	DNAJB1	filled
ENST00000512033	ENSG00000164253	WDR41	
ENST00000509971	ENSG00000164253	WDR41	filled
ENST00000510119	ENSG00000158402	CDC25C	
ENST00000482518	ENSG00000153823	PID1	filled
ENST00000559177	ENSG00000134160	TRPM1	filled
ENST00000558445	ENSG00000134160	TRPM1	filled
ENST00000542188	ENSG00000134160	TRPM1	filled
ENST00000611255	ENSG00000148655	C10orf11	filled
ENST00000593817	ENSG00000148655	C10orf11	filled
ENST00000593699	ENSG00000148655	C10orf11	filled
ENST00000372499	ENSG00000148655	C10orf11	filled
ENST00000428670	ENSG00000070961	ATP2B1	filled
ENST00000551310	ENSG00000070961	ATP2B1	filled
ENST00000493050	ENSG00000153902	LGI4	
ENST00000460948	ENSG00000186787	SPIN2B	
ENST00000273666	ENSG00000145087	STXBP5L	filled
ENST00000471454	ENSG00000145087	STXBP5L	filled
ENST00000472879	ENSG00000145087	STXBP5L	filled
ENST00000497029	ENSG00000145087	STXBP5L	filled
ENST00000492541	ENSG00000145087	STXBP5L	filled
ENST00000471262	ENSG00000145087	STXBP5L	filled
ENST00000514170	ENSG00000198515	CNGA1	filled
ENST00000560374	ENSG00000103942	HOMER2	
ENST00000500334	ENSG00000103942	HOMER2	
ENST00000619240	ENSG00000103942	HOMER2	
ENST00000222690	ENSG00000105968	H2AFV	
ENST00000455869	ENSG00000228163	HLA-DPA1	
ENST00000497897	ENSG00000106077	ABHD11	
ENST00000435124	ENSG00000196189	SEMA4A	filled
ENST00000414683	ENSG00000196189	SEMA4A	filled
ENST00000485575	ENSG00000196189	SEMA4A	
ENST00000451800	ENSG00000171097	CCBL1	filled
ENST00000416084	ENSG00000171097	CCBL1	
ENST00000322310	ENSG00000176101	SSNA1	filled
ENST00000459860	ENSG00000176101	SSNA1	filled
ENST00000464553	ENSG00000176101	SSNA1	filled
ENST00000438723	ENSG00000136828	RALGPS1	
ENST00000416134	ENSG00000235715	PSMB8	filled
ENST00000508978	ENSG00000174136	RGMB	
ENST00000530378	ENSG00000110693	SOX6	filled
ENST00000533658	ENSG00000110693	SOX6	filled
ENST00000356970	ENSG00000121940	CLCC1	filled
ENST00000473062	ENSG00000121940	CLCC1	filled
ENST00000482889	ENSG00000121940	CLCC1	filled
ENST00000369969	ENSG00000121940	CLCC1	filled
ENST00000369968	ENSG00000121940	CLCC1	filled
ENST00000383270	ENSG00000206315	PBX2	filled
ENST00000280614	ENSG00000151014	CCRN4L	filled

ENST00000402157	ENSG00000166436	TRIM66	filled
ENST00000481014	ENSG00000166436	TRIM66	filled
ENST00000529211	ENSG00000166436	TRIM66	
ENST00000531498	ENSG00000166436	TRIM66	
ENST00000299550	ENSG00000166436	TRIM66	filled
ENST00000470189	ENSG00000197223	C1D	
ENST00000483149	ENSG00000188938	FAM120AOS	
ENST00000520470	ENSG00000188938	FAM120AOS	
ENST00000483056	ENSG00000188938	FAM120AOS	
ENST00000428152	ENSG00000188938	FAM120AOS	
ENST00000565068	ENSG00000167178	ISLR2	
ENST00000355809	ENSG00000134744	ZCCHC11	filled
ENST00000470626	ENSG00000134744	ZCCHC11	filled
ENST00000524582	ENSG00000134744	ZCCHC11	filled
ENST00000582471	ENSG00000263809	KRBA2	filled
ENST00000463470	ENSG00000163159	VPS72	
ENST00000392690	ENSG00000172318	B3GALT1	
ENST00000492191	ENSG00000175166	PSMD2	
ENST00000476461	ENSG00000175166	PSMD2	
ENST00000599704	ENSG00000142227	EMP3	
ENST00000492067	ENSG00000117543	DPH5	filled
ENST00000370109	ENSG00000117543	DPH5	filled
ENST00000488789	ENSG00000117543	DPH5	filled
ENST00000427040	ENSG00000117543	DPH5	filled
ENST00000326974	ENSG00000124702	KLHDC3	filled
ENST00000244670	ENSG00000124702	KLHDC3	filled
ENST00000332245	ENSG00000124702	KLHDC3	filled
ENST00000612084	ENSG00000277909	SPC25	
ENST00000616584	ENSG00000277909	SPC25	filled
ENST00000304191	ENSG00000169612	FAM103A1	filled
ENST00000420843	ENSG00000137486	ARRB1	filled
ENST00000360025	ENSG00000137486	ARRB1	filled
ENST00000532525	ENSG00000137486	ARRB1	
ENST00000524400	ENSG00000137486	ARRB1	
ENST00000533255	ENSG00000137486	ARRB1	filled
ENST00000532790	ENSG00000166801	FAM111A	
ENST00000293842	ENSG00000161970	RPL26	
ENST00000406134	ENSG00000095002	MSH2	filled
ENST00000461394	ENSG00000095002	MSH2	filled
ENST00000467323	ENSG00000095002	MSH2	
ENST00000428675	ENSG00000115896	PLCL1	filled
ENST00000435320	ENSG00000115896	PLCL1	filled
ENST00000487695	ENSG00000115896	PLCL1	filled
ENST00000399607	ENSG00000243594	PSMB9	
ENST00000399619	ENSG00000243594	PSMB9	filled
ENST00000538597	ENSG00000149231	CCDC82	
ENST00000530203	ENSG00000149231	CCDC82	
ENST00000560202	ENSG00000140455	USP3	
ENST00000514019	ENSG00000270386	UGT2A1	filled
ENST00000468441	ENSG00000116885	OSCP1	filled
ENST00000243918	ENSG00000204070	SYS1	
ENST00000426004	ENSG00000204070	SYS1	
ENST00000493809	ENSG00000227600	PPT2	
ENST00000465812	ENSG00000136689	IL1RN	
ENST00000463073	ENSG00000136689	IL1RN	
ENST00000409052	ENSG00000136689	IL1RN	filled
ENST00000361779	ENSG00000136689	IL1RN	filled
ENST00000259206	ENSG00000136689	IL1RN	filled
ENST00000354115	ENSG00000136689	IL1RN	filled
ENST00000590320	ENSG00000267534	S1PR2	

ENST00000423128	ENSG00000205356	TECPR1	
ENST00000420697	ENSG00000205356	TECPR1	
ENST00000367727	ENSG00000180999	C1orf105	filled
ENST00000460397	ENSG00000180999	C1orf105	filled
ENST00000488100	ENSG00000180999	C1orf105	filled
ENST00000545467	ENSG00000134333	LDHA	filled
ENST00000430553	ENSG00000134333	LDHA	
ENST00000545215	ENSG00000134333	LDHA	
ENST00000555436	ENSG00000139899	CBLN3	filled
ENST00000609686	ENSG00000273079	GRIN2B	filled
ENST00000562067	ENSG00000140400	MAN2C1	
ENST00000564929	ENSG00000140400	MAN2C1	
ENST00000561693	ENSG00000140400	MAN2C1	
ENST00000564785	ENSG00000140400	MAN2C1	filled
ENST00000492026	ENSG00000119636	CCDC176	filled
ENST00000592854	ENSG00000214212	C19orf38	
ENST00000361297	ENSG00000086015	MAST2	filled
ENST00000295628	ENSG00000163428	LRRC58	
ENST00000512175	ENSG00000249693	THEGL	filled
ENST00000521826	ENSG00000164609	SLU7	
ENST00000485562	ENSG00000167103	PIP5KL1	filled
ENST00000313324	ENSG00000178397	FAM220A	
ENST00000578372	ENSG00000178397	FAM220A	
ENST00000616824	ENSG00000178397	FAM220A	
ENST00000518164	ENSG00000156482	RPL30	
ENST00000396070	ENSG00000156482	RPL30	
ENST00000503039	ENSG00000113761	ZNF346	filled
ENST00000576114	ENSG00000181222	POLR2A	
ENST00000560021	ENSG00000104059	FAM189A1	
ENST00000560082	ENSG00000104059	FAM189A1	filled
ENST00000560050	ENSG00000104059	FAM189A1	filled
ENST00000507279	ENSG00000113108	APBB3	filled
ENST00000531778	ENSG00000109956	B3GAT1	
ENST00000312527	ENSG00000109956	B3GAT1	
ENST00000524765	ENSG00000109956	B3GAT1	
ENST00000531510	ENSG00000109956	B3GAT1	
ENST00000392580	ENSG00000109956	B3GAT1	
ENST00000294635	ENSG00000162621	LRRC53	
ENST00000394455	ENSG00000072121	ZFYVE26	filled
ENST00000378182	ENSG00000204977	TRIM13	
ENST00000461459	ENSG00000227460	SYNGAP1	filled
ENST00000395071	ENSG00000227460	SYNGAP1	filled
ENST00000484671	ENSG00000227460	SYNGAP1	
ENST00000429817	ENSG00000223609	HBD	
ENST00000436575	ENSG00000164535	DAGLB	filled
ENST00000338222	ENSG00000188021	UBQLN2	
ENST00000421374	ENSG00000100014	SPECC1L	filled
ENST00000314328	ENSG00000100014	SPECC1L	filled
ENST00000440893	ENSG00000100014	SPECC1L	filled
ENST00000437398	ENSG00000100014	SPECC1L	filled
ENST00000509143	ENSG00000113328	CCNG1	
ENST00000532502	ENSG00000197601	FAR1	
ENST00000484239	ENSG00000196660	SLC30A10	filled
ENST00000399275	ENSG00000120693	SMAD9	
ENST00000350148	ENSG00000120693	SMAD9	filled
ENST00000379826	ENSG00000120693	SMAD9	filled
ENST00000261693	ENSG00000073060	SCARB1	filled
ENST00000535005	ENSG00000073060	SCARB1	filled
ENST00000541661	ENSG00000073060	SCARB1	filled
ENST00000568294	ENSG00000166503	HDGFRP3	filled

ENST00000370081	ENSG00000112695	COX7A2	
ENST00000420128	ENSG00000226124	FTCDNL1	
ENST00000622774	ENSG00000226124	FTCDNL1	
ENST00000607252	ENSG00000171467	ZNF318	filled
ENST00000605935	ENSG00000171467	ZNF318	filled
ENST00000606599	ENSG00000171467	ZNF318	
ENST00000510594	ENSG00000186314	PRELID2	filled
ENST00000515161	ENSG00000186314	PRELID2	filled
ENST00000303786	ENSG00000169618	PROKR1	filled
ENST00000274456	ENSG00000145779	TNFAIP8	filled
ENST00000388882	ENSG00000145779	TNFAIP8	filled
ENST00000556155	ENSG00000166888	STAT6	filled
ENST00000553499	ENSG00000166888	STAT6	filled
ENST00000530278	ENSG00000170322	NFRKB	
ENST00000531318	ENSG00000170322	NFRKB	
ENST00000480486	ENSG00000159259	CHAF1B	filled
ENST00000367674	ENSG00000116147	TNR	filled
ENST00000263525	ENSG00000116147	TNR	filled
ENST00000284290	ENSG00000154144	TBRG1	
ENST00000441174	ENSG00000154144	TBRG1	
ENST00000288350	ENSG00000157578	LCA5L	filled
ENST00000358268	ENSG00000157578	LCA5L	filled
ENST00000491625	ENSG00000157578	LCA5L	filled
ENST00000468009	ENSG00000157578	LCA5L	
ENST00000497401	ENSG00000182902	SLC25A18	
ENST00000616321	ENSG00000172725	CORO1B	
ENST00000525047	ENSG00000149972	CNTN5	filled
ENST00000528727	ENSG00000149972	CNTN5	filled
ENST00000527185	ENSG00000149972	CNTN5	filled
ENST00000528682	ENSG00000149972	CNTN5	filled
ENST00000524871	ENSG00000149972	CNTN5	filled
ENST00000418526	ENSG00000149972	CNTN5	filled
ENST00000530458	ENSG00000149972	CNTN5	
ENST00000361060	ENSG00000198380	GFPT1	filled
ENST00000357308	ENSG00000198380	GFPT1	filled
ENST00000533540	ENSG00000109971	HSPA8	
ENST00000533238	ENSG00000109971	HSPA8	
ENST00000528292	ENSG00000109971	HSPA8	
ENST00000525463	ENSG00000109971	HSPA8	
ENST00000322716	ENSG00000178567	EPM2AIP1	
ENST00000623924	ENSG00000178567	EPM2AIP1	
ENST00000528282	ENSG00000079819	EPB41L2	filled
ENST00000445890	ENSG00000079819	EPB41L2	filled
ENST00000337057	ENSG00000079819	EPB41L2	filled
ENST00000392427	ENSG00000079819	EPB41L2	filled
ENST00000368128	ENSG00000079819	EPB41L2	filled
ENST00000527411	ENSG00000079819	EPB41L2	filled
ENST00000525271	ENSG00000079819	EPB41L2	filled
ENST00000528179	ENSG00000079819	EPB41L2	filled
ENST00000526333	ENSG00000079819	EPB41L2	filled
ENST00000529709	ENSG00000079819	EPB41L2	
ENST00000532499	ENSG00000079819	EPB41L2	
ENST00000531356	ENSG00000079819	EPB41L2	
ENST00000530707	ENSG00000079819	EPB41L2	filled
ENST00000399344	ENSG00000206279	DAXX	
ENST00000383194	ENSG00000206279	DAXX	
ENST00000471954	ENSG00000206279	DAXX	
ENST00000494562	ENSG00000206279	DAXX	
ENST00000496639	ENSG00000206279	DAXX	
ENST00000429531	ENSG00000206279	DAXX	
ENST00000438332	ENSG00000206279	DAXX	filled
ENST00000494997	ENSG00000206279	DAXX	
ENST00000419051	ENSG00000206279	DAXX	
ENST00000497962	ENSG00000206279	DAXX	
ENST00000617660	ENSG00000206279	DAXX	
ENST00000619421	ENSG00000206279	DAXX	
ENST00000481585	ENSG00000206199	ANKUB1	filled
ENST00000473672	ENSG00000206199	ANKUB1	
ENST00000556278	ENSG00000258728	GALT	
ENST00000371222	ENSG00000177606	JUN	
ENST00000397450	ENSG00000184459	BPIFC	
ENST00000588952	ENSG00000130816	DNMT1	filled
ENST00000588118	ENSG00000130816	DNMT1	
ENST00000592342	ENSG00000130816	DNMT1	filled
ENST00000586800	ENSG00000130816	DNMT1	
ENST00000271139	ENSG00000142961	MOB3C	
ENST00000371940	ENSG00000142961	MOB3C	
ENST00000614757	ENSG00000277726	TARDBP	filled
ENST00000611136	ENSG00000277726	TARDBP	
ENST00000612542	ENSG00000277726	TARDBP	
ENST00000622108	ENSG00000277726	TARDBP	
ENST00000620028	ENSG00000277726	TARDBP	
ENST00000609590	ENSG00000272980	CCR6	filled
ENST00000451208	ENSG00000173681	CXorf23	
ENST00000456553	ENSG00000173681	CXorf23	
ENST00000482825	ENSG00000143418	CERS2	
ENST00000561294	ENSG00000143418	CERS2	
ENST00000467588	ENSG00000134256	CD101	filled
ENST00000579366	ENSG00000183010	PYCR1	
ENST00000573708	ENSG00000161920	MED11	filled
ENST00000433140	ENSG00000138080	EMILIN1	
ENST00000589898	ENSG00000067596	DHX8	
ENST00000512760	ENSG00000163291	PAQR3	filled
ENST00000342820	ENSG00000163291	PAQR3	filled
ENST00000511594	ENSG00000163291	PAQR3	filled
ENST00000512733	ENSG00000163291	PAQR3	filled
ENST00000515541	ENSG00000163291	PAQR3	filled
ENST00000503343	ENSG00000163291	PAQR3	filled
ENST00000489002	ENSG00000135845	PIGC	
ENST00000465374	ENSG00000135845	PIGC	
ENST00000475059	ENSG00000135845	PIGC	
ENST00000557161	ENSG00000213927	CCL27	
ENST00000256951	ENSG00000134531	EMP1	
ENST00000431267	ENSG00000134531	EMP1	
ENST00000544053	ENSG00000134531	EMP1	
ENST00000542289	ENSG00000134531	EMP1	
ENST00000553597	ENSG00000128739	SNRPN	
ENST00000569236	ENSG00000169189	NSMCE1	filled
ENST00000562039	ENSG00000169189	NSMCE1	
ENST00000567710	ENSG00000169189	NSMCE1	filled
ENST00000563512	ENSG00000169189	NSMCE1	filled
ENST00000596918	ENSG00000268193	AC002985.3	
ENST00000593484	ENSG00000268193	AC002985.3	
ENST00000414301	ENSG00000001617	SEMA3F	
ENST00000426511	ENSG00000001617	SEMA3F	
ENST00000624676	ENSG00000185022	MAFF	
ENST00000304916	ENSG00000168672	FAM84B	
ENST00000517458	ENSG00000168672	FAM84B	
ENST00000484437	ENSG00000163807	KIAA1143	
ENST00000296121	ENSG00000163807	KIAA1143	

ENST00000498051	ENSG00000187955	COL14A1	filled
ENST00000373020	ENSG00000000003	TSPAN6	
ENST00000494424	ENSG00000000003	TSPAN6	
ENST00000612152	ENSG00000000003	TSPAN6	
ENST00000366978	ENSG00000117697	NSL1	
ENST00000396267	ENSG00000184619	KRBA2	filled
ENST00000331336	ENSG00000184619	KRBA2	filled
ENST00000339859	ENSG00000166348	USP54	filled
ENST00000451492	ENSG00000166348	USP54	filled
ENST00000413442	ENSG00000166348	USP54	filled
ENST00000433394	ENSG00000166348	USP54	filled
ENST00000498213	ENSG00000166348	USP54	
ENST00000475035	ENSG00000182004	SNRPE	
ENST00000469451	ENSG00000182004	SNRPE	
ENST00000380599	ENSG00000174282	ZBTB4	filled
ENST00000311403	ENSG00000174282	ZBTB4	filled
ENST00000608456	ENSG00000111906	HDDC2	filled
ENST00000609477	ENSG00000111906	HDDC2	filled
ENST00000507126	ENSG00000174780	SRP72	
ENST00000602841	ENSG00000163156	SCNM1	
ENST00000615958	ENSG00000151023	ENKUR	filled
ENST00000262839	ENSG00000072315	TRPC5	filled
ENST00000478555	ENSG00000174514	MFSD4	
ENST00000467303	ENSG00000145016	KIAA0226	filled
ENST00000592858	ENSG00000129932	DOHH	
ENST00000593124	ENSG00000105401	CDC37	filled
ENST00000588847	ENSG00000078687	TNRC6C	filled
ENST00000588061	ENSG00000078687	TNRC6C	filled
ENST00000368854	ENSG00000121898	CPXM2	filled
ENST00000241305	ENSG00000121898	CPXM2	filled
ENST00000615851	ENSG00000121898	CPXM2	filled
ENST00000371220	ENSG00000153294	GPR115	
ENST00000593999	ENSG00000268434	AC011530.4	
ENST00000596586	ENSG00000268434	AC011530.4	
ENST00000597712	ENSG00000268434	AC011530.4	
ENST00000595946	ENSG00000268434	AC011530.4	
ENST00000510771	ENSG00000170180	GYPA	
ENST00000418460	ENSG00000212916	MAP10	
ENST00000275461	ENSG00000146618	FERD3L	
ENST00000399371	ENSG00000240118	PSMB9	
ENST00000399381	ENSG00000240118	PSMB9	filled
ENST00000513724	ENSG00000163293	NIPAL1	filled
ENST00000511123	ENSG00000163293	NIPAL1	filled
ENST00000490060	ENSG00000075790	BCAP29	
ENST00000421797	ENSG00000128585	MKLN1	filled
ENST00000416992	ENSG00000128585	MKLN1	filled
ENST00000360299	ENSG00000111540	RAB5B	
ENST00000548068	ENSG00000111540	RAB5B	
ENST00000549915	ENSG00000111540	RAB5B	
ENST00000551459	ENSG00000111540	RAB5B	
ENST00000550283	ENSG00000111540	RAB5B	filled
ENST00000464516	ENSG00000125944	HNRNPR	filled
ENST00000448504	ENSG00000141337	ARSG	filled
ENST00000578726	ENSG00000141337	ARSG	filled
ENST00000622383	ENSG00000119919	NKX2-3	
ENST00000582678	ENSG00000159640	ACE	filled
ENST00000494827	ENSG00000070087	PFN2	filled
ENST00000375863	ENSG00000204428	LY6G5C	
ENST00000474395	ENSG00000204428	LY6G5C	
ENST00000467098	ENSG00000204428	LY6G5C	

ENST00000440956	ENSG00000231974	APOM	
ENST00000443975	ENSG00000231974	APOM	
ENST00000419959	ENSG00000165092	ALDH1A1	filled
ENST00000446946	ENSG00000165092	ALDH1A1	filled
ENST00000370435	ENSG00000119900	OGFRL1	
ENST00000617675	ENSG00000144597	EAF1	
ENST00000458404	ENSG00000196632	WNK3	
ENST00000531705	ENSG00000149792	MRPL49	filled
ENST00000404895	ENSG00000156687	UNC5D	filled
ENST00000420357	ENSG00000156687	UNC5D	filled
ENST00000287272	ENSG00000156687	UNC5D	filled
ENST00000416672	ENSG00000156687	UNC5D	filled
ENST00000299415	ENSG00000166329	CCDC182	
ENST00000486100	ENSG00000196504	PRPF40A	
ENST00000512180	ENSG00000120709	FAM53C	
ENST00000511024	ENSG00000120709	FAM53C	filled
ENST00000393437	ENSG00000170500	LONRF2	
ENST00000336576	ENSG00000135924	DNAJB2	
ENST00000425450	ENSG00000135924	DNAJB2	
ENST00000392086	ENSG00000135924	DNAJB2	
ENST00000480537	ENSG00000135924	DNAJB2	
ENST00000529191	ENSG00000167996	FTH1	filled
ENST00000529631	ENSG00000167996	FTH1	filled
ENST00000530019	ENSG00000167996	FTH1	filled
ENST00000289431	ENSG00000158480	SPATA2	
ENST00000422556	ENSG00000158480	SPATA2	
ENST00000249861	ENSG00000129028	THAP10	filled
ENST00000560604	ENSG00000129028	THAP10	filled
ENST00000517748	ENSG00000176571	CNBD1	
ENST00000523299	ENSG00000176571	CNBD1	filled
ENST00000620117	ENSG00000244025	KRTAP19-3	
ENST00000538862	ENSG00000111665	CDCA3	
ENST00000596271	ENSG00000179134	SAMD4B	
ENST00000568315	ENSG00000103061	SLC7A6OS	
ENST00000263997	ENSG00000103061	SLC7A6OS	filled
ENST00000561933	ENSG00000103061	SLC7A6OS	filled
ENST00000375043	ENSG00000213654	GPSM3	
ENST00000373266	ENSG00000142687	KIAA0319L	filled
ENST00000392927	ENSG00000139351	SYCP3	
ENST00000502534	ENSG00000109475	RPL34	
ENST00000394668	ENSG00000109475	RPL34	
ENST00000528273	ENSG00000159314	ARHGAP27	
ENST00000475242	ENSG00000254806	SYS1-DBNDD2	
ENST00000458187	ENSG00000254806	SYS1-DBNDD2	
ENST00000583676	ENSG00000258890	CEP95	filled
ENST00000483209	ENSG00000121578	B4GALT4	filled
ENST00000467604	ENSG00000121578	B4GALT4	filled
ENST00000393765	ENSG00000121578	B4GALT4	filled
ENST00000480814	ENSG00000121578	B4GALT4	filled
ENST00000491906	ENSG00000121578	B4GALT4	
ENST00000475803	ENSG00000121578	B4GALT4	
ENST00000479150	ENSG00000121578	B4GALT4	
ENST00000470111	ENSG00000121578	B4GALT4	
ENST00000473887	ENSG00000121578	B4GALT4	
ENST00000459778	ENSG00000121578	B4GALT4	
ENST00000460395	ENSG00000121578	B4GALT4	
ENST00000340096	ENSG00000046651	OFD1	filled
ENST00000380567	ENSG00000046651	OFD1	filled
ENST00000490265	ENSG00000046651	OFD1	filled
ENST00000398395	ENSG00000046651	OFD1	filled

ENST00000380550	ENSG00000046651	OFD1	filled
ENST00000537098	ENSG00000153815	CMIP	
ENST00000502669	ENSG00000125089	SH3TC1	
ENST00000245105	ENSG00000125089	SH3TC1	
ENST00000515682	ENSG00000125089	SH3TC1	
ENST00000244020	ENSG00000124193	SRSF6	filled
ENST00000420986	ENSG00000150776	C11orf57	
ENST00000532163	ENSG00000150776	C11orf57	
ENST00000280352	ENSG00000150776	C11orf57	
ENST00000393047	ENSG00000150776	C11orf57	
ENST00000465440	ENSG00000143178	TBX19	
ENST00000555829	ENSG00000187097	ENTPD5	
ENST00000245458	ENSG00000213741	RPS29	
ENST00000428180	ENSG00000206291	HLA-DPA1	
ENST00000607320	ENSG00000131378	RFTN1	filled
ENST00000360473	ENSG00000198553	KCNRG	
ENST00000312942	ENSG00000198553	KCNRG	
ENST00000375608	ENSG00000126217	MCF2L	filled
ENST00000397030	ENSG00000126217	MCF2L	filled
ENST00000569649	ENSG00000178573	MAF	
ENST00000477917	ENSG00000135924	DNAJB2	
ENST00000392087	ENSG00000135924	DNAJB2	
ENST00000433948	ENSG00000167566	NCKAP5L	
ENST00000468956	ENSG00000126767	ELK1	
ENST00000531207	ENSG00000179240	RP11-111M22.2	
ENST00000530460	ENSG00000179240	RP11-111M22.2	
ENST00000529331	ENSG00000179240	RP11-111M22.2	
ENST00000534586	ENSG00000179240	RP11-111M22.2	
ENST00000369727	ENSG00000156384	SFR1	
ENST00000287878	ENSG00000106617	PRKAG2	filled
ENST00000392801	ENSG00000106617	PRKAG2	filled
ENST00000382252	ENSG00000205903	ZNF316	filled
ENST00000427912	ENSG00000205903	ZNF316	
ENST00000219204	ENSG00000102931	ARL2BP	
ENST00000564698	ENSG00000137842	TMEM62	
ENST00000239231	ENSG00000120137	PANK3	
ENST00000475483	ENSG00000069849	ATP1B3	filled
ENST00000286371	ENSG00000069849	ATP1B3	filled
ENST00000465172	ENSG00000069849	ATP1B3	filled
ENST00000476353	ENSG00000223680	DDR1	
ENST00000397633	ENSG00000214140	PRCD	
ENST00000465808	ENSG00000214140	PRCD	
ENST00000397630	ENSG00000214140	PRCD	
ENST00000592340	ENSG00000214140	PRCD	
ENST00000587289	ENSG00000214140	PRCD	
ENST00000591317	ENSG00000214140	PRCD	
ENST00000525730	ENSG00000110013	SIAE	
ENST00000445860	ENSG00000171405	XAGE5	
ENST00000521548	ENSG00000214050	FBXO16	filled
ENST00000380254	ENSG00000214050	FBXO16	filled
ENST00000522609	ENSG00000214050	FBXO16	
ENST00000346498	ENSG00000214050	FBXO16	filled
ENST00000518734	ENSG00000214050	FBXO16	filled
ENST00000551598	ENSG00000187778	MCRS1	
ENST00000549743	ENSG00000167548	KMT2D	filled
ENST00000547610	ENSG00000167548	KMT2D	
ENST00000542218	ENSG00000137310	TCF19	
ENST00000375123	ENSG00000178919	FOXE1	filled
ENST00000551074	ENSG00000139287	TPH2	filled
ENST00000573524	ENSG00000273086	TAS2R9	

ENST00000521585	ENSG00000170091	GPIHBP1	filled
ENST00000468388	ENSG00000198830	HMGN2	
ENST00000479815	ENSG00000198830	HMGN2	
ENST00000460563	ENSG00000198830	HMGN2	
ENST00000464888	ENSG00000198830	HMGN2	
ENST00000463817	ENSG00000198830	HMGN2	
ENST00000467700	ENSG00000198830	HMGN2	
ENST00000361427	ENSG00000198830	HMGN2	filled
ENST00000466194	ENSG00000198830	HMGN2	
ENST00000493418	ENSG00000198830	HMGN2	
ENST00000619352	ENSG00000198830	HMGN2	filled
ENST00000453996	ENSG00000174177	CTU2	filled
ENST00000312060	ENSG00000174177	CTU2	filled
ENST00000603967	ENSG00000270647	TAF15	filled
ENST00000604195	ENSG00000270647	TAF15	
ENST00000522135	ENSG00000132561	MATN2	
ENST00000316950	ENSG00000196437	ZNF569	filled
ENST00000577830	ENSG00000180891	CUEDC1	
ENST00000272233	ENSG00000143878	RHOB	
ENST00000571047	ENSG00000132510	KDM6B	
ENST00000254846	ENSG00000132510	KDM6B	
ENST00000448097	ENSG00000132510	KDM6B	
ENST00000572030	ENSG00000132510	KDM6B	
ENST00000575521	ENSG00000132510	KDM6B	
ENST00000570632	ENSG00000132510	KDM6B	
ENST00000552901	ENSG00000172819	RARG	
ENST00000375406	ENSG00000117148	ACTL8	
ENST00000333924	ENSG00000186666	BCDIN3D	
ENST00000358743	ENSG00000183963	SMTN	filled
ENST00000347557	ENSG00000183963	SMTN	filled
ENST00000333137	ENSG00000183963	SMTN	filled
ENST00000489337	ENSG00000183963	SMTN	filled
ENST00000520381	ENSG00000066777	ARFGEF1	
ENST00000522878	ENSG00000066777	ARFGEF1	
ENST00000262215	ENSG00000066777	ARFGEF1	filled
ENST00000519436	ENSG00000066777	ARFGEF1	filled
ENST00000616266	ENSG00000269313	MAGIX	
ENST00000339824	ENSG00000171435	KSR2	filled
ENST00000545002	ENSG00000171435	KSR2	filled
ENST00000543793	ENSG00000171435	KSR2	
ENST00000425217	ENSG00000171435	KSR2	filled
ENST00000561551	ENSG00000136404	TM6SF1	
ENST00000595676	ENSG00000268465	CTC-273B12.7	
ENST00000354209	ENSG00000132356	PRKAA1	filled
ENST00000419706	ENSG00000183826	BTBD9	filled
ENST00000328403	ENSG00000183826	BTBD9	filled
ENST00000497373	ENSG00000183826	BTBD9	filled
ENST00000498633	ENSG00000183826	BTBD9	filled
ENST00000408958	ENSG00000183826	BTBD9	filled
ENST00000579794	ENSG00000141433	ADCYAP1	
ENST00000581602	ENSG00000141433	ADCYAP1	
ENST00000489094	ENSG00000006704	GTF2IRD1	filled
ENST00000500323	ENSG00000146094	DOK3	
ENST00000508270	ENSG00000170482	SLC23A1	
ENST00000420041	ENSG00000233490	GPSM3	
ENST00000404933	ENSG00000102172	SMS	filled
ENST00000379404	ENSG00000102172	SMS	filled
ENST00000585851	ENSG00000118046	STK11	
ENST00000586358	ENSG00000118046	STK11	
ENST00000401705	ENSG00000213889	PPM1N	filled

ENST00000532489	ENSG00000126457	PRMT1	filled
ENST00000527382	ENSG00000126457	PRMT1	
ENST00000528623	ENSG00000126457	PRMT1	
ENST00000526224	ENSG00000126457	PRMT1	
ENST00000306378	ENSG00000171634	BPTF	filled
ENST00000424123	ENSG00000171634	BPTF	filled
ENST00000577770	ENSG00000171634	BPTF	filled
ENST00000321892	ENSG00000171634	BPTF	filled
ENST00000549856	ENSG00000187510	PLEKHG7	
ENST00000486895	ENSG00000176124	DLEU1	filled
ENST00000483444	ENSG00000176124	DLEU1	filled
ENST00000463357	ENSG00000176124	DLEU1	filled
ENST00000489542	ENSG00000176124	DLEU1	
ENST00000483169	ENSG00000176124	DLEU1	
ENST00000469095	ENSG00000176124	DLEU1	
ENST00000409572	ENSG00000153234	NR4A2	filled
ENST00000476844	ENSG00000189283	FHIT	filled
ENST00000492590	ENSG00000189283	FHIT	filled
ENST00000466788	ENSG00000189283	FHIT	filled
ENST00000468189	ENSG00000189283	FHIT	filled
ENST00000488467	ENSG00000189283	FHIT	filled
ENST00000490952	ENSG00000189283	FHIT	filled
ENST00000465330	ENSG00000189283	FHIT	
ENST00000506466	ENSG00000066427	ATXN3	
ENST00000445326	ENSG00000234508	GPSM3	
ENST00000543605	ENSG00000080189	SLC35C2	
ENST00000372227	ENSG00000080189	SLC35C2	
ENST00000474362	ENSG00000095794	CREM	
ENST00000374721	ENSG00000095794	CREM	
ENST00000461968	ENSG00000095794	CREM	
ENST00000490460	ENSG00000095794	CREM	
ENST00000466251	ENSG00000095794	CREM	
ENST00000495960	ENSG00000095794	CREM	
ENST00000429130	ENSG00000095794	CREM	
ENST00000460270	ENSG00000095794	CREM	
ENST00000354759	ENSG00000095794	CREM	
ENST00000345491	ENSG00000095794	CREM	
ENST00000487132	ENSG00000095794	CREM	
ENST00000337656	ENSG00000095794	CREM	
ENST00000374734	ENSG00000095794	CREM	
ENST00000479070	ENSG00000095794	CREM	
ENST00000348787	ENSG00000095794	CREM	
ENST00000464475	ENSG00000095794	CREM	
ENST00000374728	ENSG00000095794	CREM	
ENST00000439705	ENSG00000095794	CREM	
ENST00000436628	ENSG00000232575	TUBB	
ENST00000437490	ENSG00000232575	TUBB	
ENST00000602836	ENSG00000270181	BIVM-ERCC5	
ENST00000552023	ENSG00000123066	MED13L	
ENST00000548743	ENSG00000123066	MED13L	
ENST00000598878	ENSG00000206562	METTL6	filled
ENST00000307169	ENSG00000168348	INSM2	
ENST00000580241	ENSG00000179761	PIPOX	filled
ENST00000580383	ENSG00000179761	PIPOX	filled
ENST00000583215	ENSG00000179761	PIPOX	filled
ENST00000578748	ENSG00000179761	PIPOX	filled
ENST00000509093	ENSG00000138663	COPS4	filled
ENST00000509317	ENSG00000138663	COPS4	
ENST00000240691	ENSG00000121381	TAS2R9	
ENST00000523651	ENSG00000174705	SH3PXD2B	

ENST00000518522	ENSG00000174705	SH3PXD2B	filled
ENST00000308910	ENSG00000174500	GCSAM	
ENST00000585477	ENSG00000228075	BOD1L2	
ENST00000546744	ENSG00000185920	PTCH1	filled
ENST00000619327	ENSG00000261221	ZNF865	filled
ENST00000448750	ENSG00000132341	RAN	
ENST00000589284	ENSG00000072062	PRKACA	filled
ENST00000450402	ENSG00000171621	SPSB1	filled
ENST00000519913	ENSG00000146007	ZMAT2	
ENST00000548458	ENSG00000235162	C12orf75	filled
ENST00000562197	ENSG00000261594	TPBGL	
ENST00000367240	ENSG00000143847	PPFIA4	filled
ENST00000515488	ENSG00000087206	UIMC1	filled
ENST00000340110	ENSG00000188691	OR56A5	filled
ENST00000588790	ENSG00000183401	CCDC159	
ENST00000458408	ENSG00000183401	CCDC159	
ENST00000511542	ENSG00000156269	NAA11	filled
ENST00000513733	ENSG00000156269	NAA11	filled
ENST00000306773	ENSG00000171246	NPTX1	
ENST00000550603	ENSG00000177425	PAWR	
ENST00000470844	ENSG00000154222	CC2D1B	
ENST00000564884	ENSG00000040199	PHLPP2	
ENST00000592996	ENSG00000173581	CCDC106	filled
ENST00000594663	ENSG00000269035	CTD-2521M24.10	
ENST00000621736	ENSG00000278570	NR2E3	filled
ENST00000563709	ENSG00000278570	NR2E3	
ENST00000404922	ENSG00000163520	FBLN2	filled
ENST00000595618	ENSG00000099331	MYO9B	filled
ENST00000594824	ENSG00000099331	MYO9B	filled
ENST00000496132	ENSG00000196990	FAM163B	
ENST00000356873	ENSG00000196990	FAM163B	
ENST00000371795	ENSG00000152778	IFIT5	
ENST00000592740	ENSG00000266953	RP11-618P17.4	
ENST00000606020	ENSG00000266953	RP11-618P17.4	
ENST00000429238	ENSG00000249209	AP000304.12	filled
ENST00000404076	ENSG00000163531	NFASC	filled
ENST00000360049	ENSG00000163531	NFASC	filled
ENST00000339876	ENSG00000163531	NFASC	filled
ENST00000539706	ENSG00000163531	NFASC	filled
ENST00000556810	ENSG00000070269	TMEM260	filled
ENST00000395018	ENSG00000213578	CPLX3	
ENST00000421362	ENSG00000174038	C9orf131	
ENST00000354479	ENSG00000174038	C9orf131	
ENST00000312292	ENSG00000174038	C9orf131	
ENST00000378745	ENSG00000174038	C9orf131	
ENST00000416537	ENSG00000174038	C9orf131	
ENST00000317623	ENSG00000126773	PCNXL4	filled
ENST00000406949	ENSG00000126773	PCNXL4	filled
ENST00000567873	ENSG00000133121	STARD13	filled
ENST00000439831	ENSG00000133121	STARD13	
ENST00000523477	ENSG00000169427	KCNK9	
ENST00000519923	ENSG00000169427	KCNK9	
ENST00000522781	ENSG00000188916	FAM196A	
ENST00000614311	ENSG00000188916	FAM196A	
ENST00000562663	ENSG00000205517	RGL3	
ENST00000568628	ENSG00000205517	RGL3	
ENST00000533753	ENSG00000168060	NAALADL1	
ENST00000473014	ENSG00000066056	TIE1	filled
ENST00000557577	ENSG00000133961	NUMB	
ENST00000557581	ENSG00000133961	NUMB	

ENST00000438370	ENSG00000164051	CCDC51	
ENST00000328880	ENSG00000182782	HCAR2	
ENST00000604213	ENSG00000183060	LYSMD4	
ENST00000496108	ENSG00000183060	LYSMD4	
ENST00000528880	ENSG00000255398	HCAR3	
ENST00000308811	ENSG00000172748	ZNF596	
ENST00000521270	ENSG00000172748	ZNF596	
ENST00000320552	ENSG00000172748	ZNF596	
ENST00000398612	ENSG00000172748	ZNF596	
ENST00000592881	ENSG00000213015	ZNF580	
ENST00000451199	ENSG00000205111	CDKL4	filled
ENST00000489878	ENSG00000143479	DYRK3	filled
ENST00000412482	ENSG00000151690	MFSD6	
ENST00000374583	ENSG00000181191	PJA1	
ENST00000374584	ENSG00000181191	PJA1	
ENST00000298943	ENSG00000165985	C1QL3	filled
ENST00000598766	ENSG00000167619	TMEM145	filled
ENST00000323534	ENSG00000181467	RAP2B	filled
ENST00000564376	ENSG00000102934	PLLP	
ENST00000531293	ENSG00000170290	SLN	filled
ENST00000525934	ENSG00000170290	SLN	filled
ENST00000565290	ENSG00000213648	SULT1A4	filled
ENST00000562941	ENSG00000213648	SULT1A4	filled
ENST00000589280	ENSG00000142065	ZFP14	
ENST00000497081	ENSG00000166482	MFAP4	
ENST00000395592	ENSG00000166482	MFAP4	
ENST00000299610	ENSG00000166482	MFAP4	
ENST00000311459	ENSG00000125375	ATP5S	filled
ENST00000623038	ENSG00000237787	C3orf79	filled
ENST00000446603	ENSG00000237787	C3orf79	filled
ENST00000432879	ENSG00000099901	RANBP1	
ENST00000464093	ENSG00000180098	TRNAU1AP	filled
ENST00000261994	ENSG00000140093	SERPINA10	
ENST00000570293	ENSG00000259900	RP11-343C2.7	
ENST00000564737	ENSG00000259900	RP11-343C2.7	
ENST00000509146	ENSG00000183876	ARSI	filled
ENST00000458416	ENSG00000232237	ASCL5	
ENST00000267176	ENSG00000139697	SBNO1	filled
ENST00000602398	ENSG00000139697	SBNO1	filled
ENST00000548258	ENSG00000094914	AAAS	filled
ENST00000551724	ENSG00000094914	AAAS	filled
ENST00000333891	ENSG00000186472	PCLO	filled
ENST00000423517	ENSG00000186472	PCLO	filled
ENST00000461143	ENSG00000186472	PCLO	
ENST00000481367	ENSG00000134330	IAH1	filled
ENST00000490621	ENSG00000134330	IAH1	
ENST00000513208	ENSG00000155011	DKK2	filled
ENST00000510463	ENSG00000155011	DKK2	filled
ENST00000402284	ENSG00000100029	PES1	
ENST00000335214	ENSG00000100029	PES1	
ENST00000406208	ENSG00000100029	PES1	
ENST00000573493	ENSG00000261884	CTC-479C5.12	
ENST00000428074	ENSG00000157214	STEAP2	filled
ENST00000323319	ENSG00000177300	CLDN22	
ENST00000460604	ENSG00000127124	HIVEP3	
ENST00000491442	ENSG00000127124	HIVEP3	filled
ENST00000331925	ENSG00000184009	ACTG1	
ENST00000574671	ENSG00000184009	ACTG1	
ENST00000576209	ENSG00000184009	ACTG1	
ENST00000572105	ENSG00000184009	ACTG1	filled

ENST00000576214	ENSG00000184009	ACTG1	filled
ENST00000571691	ENSG00000184009	ACTG1	filled
ENST00000575659	ENSG00000184009	ACTG1	
ENST00000570382	ENSG00000184009	ACTG1	
ENST00000615544	ENSG00000184009	ACTG1	filled
ENST00000398399	ENSG00000206538	VGLL3	
ENST00000378693	ENSG00000198944	SOWAHA	
ENST00000512940	ENSG00000168916	ZNF608	
ENST00000546873	ENSG00000111666	CHPT1	filled
ENST00000589656	ENSG00000115268	RPS15	
ENST00000585665	ENSG00000115268	RPS15	
ENST00000403058	ENSG00000106536	POU6F2	filled
ENST00000451021	ENSG00000106536	POU6F2	filled
ENST00000416452	ENSG00000106536	POU6F2	filled
ENST00000619133	ENSG00000272398	CD24	
ENST00000615659	ENSG00000272398	CD24	
ENST00000610952	ENSG00000272398	CD24	
ENST00000621311	ENSG00000272398	CD24	
ENST00000606017	ENSG00000272398	CD24	
ENST00000299004	ENSG00000166025	AMOTL1	filled
ENST00000418500	ENSG00000123500	COL10A1	
ENST00000587250	ENSG00000108830	RND2	
ENST00000558132	ENSG00000182718	ANXA2	
ENST00000559113	ENSG00000182718	ANXA2	
ENST00000558558	ENSG00000182718	ANXA2	
ENST00000449959	ENSG00000172322	CLEC12A	
ENST00000487463	ENSG00000102316	MAGED2	filled
ENST00000513720	ENSG00000081051	AFP	
ENST00000303004	ENSG00000172216	CEBPB	
ENST00000509262	ENSG00000163633	C4orf36	
ENST00000503159	ENSG00000163633	C4orf36	
ENST00000465275	ENSG00000188738	FSIP2	
ENST00000469367	ENSG00000188738	FSIP2	
ENST00000416611	ENSG00000011332	DPF1	
ENST00000418517	ENSG00000011332	DPF1	
ENST00000456296	ENSG00000011332	DPF1	
ENST00000412732	ENSG00000011332	DPF1	
ENST00000367701	ENSG00000185278	ZBTB37	filled
ENST00000398417	ENSG00000141646	SMAD4	filled
ENST00000589076	ENSG00000141646	SMAD4	
ENST00000590061	ENSG00000141646	SMAD4	
ENST00000591914	ENSG00000141646	SMAD4	
ENST00000592911	ENSG00000141646	SMAD4	
ENST00000589941	ENSG00000141646	SMAD4	
ENST00000342988	ENSG00000141646	SMAD4	filled
ENST00000512905	ENSG00000080819	CPOX	
ENST00000591369	ENSG00000187775	DNAH17	filled
ENST00000585328	ENSG00000187775	DNAH17	filled
ENST00000586052	ENSG00000187775	DNAH17	filled
ENST00000592152	ENSG00000187775	DNAH17	
ENST00000389840	ENSG00000187775	DNAH17	filled
ENST00000247226	ENSG00000126822	PLEKHG3	filled
ENST00000554088	ENSG00000126822	PLEKHG3	filled
ENST00000554499	ENSG00000126822	PLEKHG3	
ENST00000393359	ENSG00000135966	TGFBRAP1	
ENST00000453056	ENSG00000156931	VPS8	
ENST00000475580	ENSG00000156931	VPS8	
ENST00000471655	ENSG00000156931	VPS8	
ENST00000492449	ENSG00000156931	VPS8	filled
ENST00000623295	ENSG00000168746	C20orf62	

ENST00000372910	ENSG00000168746	C20orf62	
ENST00000473112	ENSG00000128928	IVD	filled
ENST00000481262	ENSG00000128928	IVD	filled
ENST00000491554	ENSG00000128928	IVD	filled
ENST00000466756	ENSG00000128928	IVD	
ENST00000267377	ENSG00000139874	SSTR1	
ENST00000369697	ENSG00000171385	KCND3	filled
ENST00000315987	ENSG00000171385	KCND3	filled
ENST00000302127	ENSG00000171385	KCND3	filled
ENST00000451794	ENSG00000163121	NEURL3	
ENST00000435380	ENSG00000163121	NEURL3	
ENST00000444588	ENSG00000163121	NEURL3	
ENST00000392653	ENSG00000241794	SPRR2A	filled
ENST00000339241	ENSG00000164056	SPRY1	
ENST00000394339	ENSG00000164056	SPRY1	
ENST00000508849	ENSG00000164056	SPRY1	
ENST00000610581	ENSG00000164056	SPRY1	
ENST00000622283	ENSG00000164056	SPRY1	
ENST00000422730	ENSG00000229637	PRAC2	
ENST00000515852	ENSG00000164292	RHOBTB3	filled
ENST00000510226	ENSG00000164292	RHOBTB3	filled
ENST00000504949	ENSG00000164292	RHOBTB3	filled
ENST00000251195	ENSG00000092853	CLSPN	filled
ENST00000518740	ENSG00000197763	TXNRD3	
ENST00000564377	ENSG00000163637	PRICKLE2	filled
ENST00000455915	ENSG00000100401	RANGAP1	filled
ENST00000309285	ENSG00000173578	XCR1	
ENST00000372141	ENSG00000185737	NRG3	filled
ENST00000404547	ENSG00000185737	NRG3	filled
ENST00000372142	ENSG00000185737	NRG3	filled
ENST00000602794	ENSG00000185737	NRG3	filled
ENST00000555784	ENSG00000185737	NRG3	filled
ENST00000404576	ENSG00000185737	NRG3	filled
ENST00000556918	ENSG00000185737	NRG3	filled
ENST00000591107	ENSG00000141458	NPC1	
ENST00000590723	ENSG00000141458	NPC1	
ENST00000586150	ENSG00000141458	NPC1	
ENST00000269228	ENSG00000141458	NPC1	filled
ENST00000540608	ENSG00000141458	NPC1	filled
ENST00000497522	ENSG00000143224	PPOX	
ENST00000495483	ENSG00000143224	PPOX	
ENST00000535223	ENSG00000143224	PPOX	
ENST00000534099	ENSG00000166402	TUB	filled
ENST00000464611	ENSG00000075624	ACTB	
ENST00000452676	ENSG00000197937	ZNF347	filled
ENST00000369724	ENSG00000013392	RWDD2A	
ENST00000551788	ENSG00000159256	MORC3	filled
ENST00000551367	ENSG00000159256	MORC3	filled
ENST00000547657	ENSG00000159256	MORC3	filled
ENST00000520956	ENSG00000239704	CDRT4	filled
ENST00000421743	ENSG00000117152	RGS4	
ENST00000367909	ENSG00000117152	RGS4	
ENST00000367908	ENSG00000117152	RGS4	
ENST00000491263	ENSG00000117152	RGS4	
ENST00000389010	ENSG00000138756	BMP2K	filled
ENST00000502871	ENSG00000138756	BMP2K	filled
ENST00000335016	ENSG00000138756	BMP2K	filled
ENST00000419792	ENSG00000235067	TUBB	
ENST00000436335	ENSG00000235067	TUBB	
ENST00000329271	ENSG00000164284	GRPEL2	filled

ENST00000507562	ENSG00000164284	GRPEL2	filled
ENST00000619297	ENSG00000273706	LHX1	
ENST00000621767	ENSG00000273706	LHX1	
ENST00000310961	ENSG00000033122	LRRC7	filled
ENST00000035383	ENSG00000033122	LRRC7	filled
ENST00000588515	ENSG00000033122	LRRC7	filled
ENST00000609498	ENSG00000033122	LRRC7	
ENST00000608815	ENSG00000033122	LRRC7	
ENST00000608744	ENSG00000033122	LRRC7	
ENST00000609072	ENSG00000033122	LRRC7	
ENST00000608470	ENSG00000033122	LRRC7	
ENST00000607936	ENSG00000033122	LRRC7	
ENST00000565615	ENSG00000033122	LRRC7	
ENST00000441830	ENSG00000033122	LRRC7	
ENST00000415775	ENSG00000033122	LRRC7	filled
ENST00000259006	ENSG00000136490	LIMD2	
ENST00000584645	ENSG00000136490	LIMD2	
ENST00000588188	ENSG00000108946	PRKAR1A	
ENST00000276461	ENSG00000147475	ERLIN2	
ENST00000573035	ENSG00000263001	GTF2I	filled
ENST00000614986	ENSG00000263001	GTF2I	filled
ENST00000621734	ENSG00000263001	GTF2I	filled
ENST00000620879	ENSG00000263001	GTF2I	filled
ENST00000432143	ENSG00000263001	GTF2I	
ENST00000495084	ENSG00000082438	COBLL1	filled
ENST00000537147	ENSG00000213088	ACKR1	
ENST00000463043	ENSG00000162413	KLHL21	
ENST00000404115	ENSG00000158290	CUL4B	filled
ENST00000588860	ENSG00000141646	SMAD4	
ENST00000469537	ENSG00000160785	SLC25A44	filled
ENST00000482737	ENSG00000160785	SLC25A44	
ENST00000529773	ENSG00000172409	CLP1	
ENST00000367267	ENSG00000143858	SYT2	filled
ENST00000545700	ENSG00000139192	TAPBPL	
ENST00000373913	ENSG00000080845	DLGAP4	filled
ENST00000373907	ENSG00000080845	DLGAP4	filled
ENST00000401952	ENSG00000080845	DLGAP4	filled
ENST00000339266	ENSG00000080845	DLGAP4	filled
ENST00000556539	ENSG00000170581	STAT2	
ENST00000312422	ENSG00000174982	OR4S2	
ENST00000525071	ENSG00000110660	SLC35F2	filled
ENST00000344293	ENSG00000165632	TAF3	filled
ENST00000497343	ENSG00000163590	PPM1L	filled
ENST00000498165	ENSG00000163590	PPM1L	filled
ENST00000577257	ENSG00000167733	HSD11B1L	
ENST00000581893	ENSG00000167733	HSD11B1L	
ENST00000582346	ENSG00000167733	HSD11B1L	
ENST00000584904	ENSG00000167733	HSD11B1L	
ENST00000579562	ENSG00000167733	HSD11B1L	
ENST00000342970	ENSG00000167733	HSD11B1L	
ENST00000422535	ENSG00000167733	HSD11B1L	
ENST00000578167	ENSG00000167733	HSD11B1L	
ENST00000577701	ENSG00000167733	HSD11B1L	
ENST00000339423	ENSG00000167733	HSD11B1L	
ENST00000578832	ENSG00000167733	HSD11B1L	
ENST00000577920	ENSG00000167733	HSD11B1L	
ENST00000530149	ENSG00000177963	RIC8A	
ENST00000513458	ENSG00000153006	SREK1IP1	filled
ENST00000495198	ENSG00000153006	SREK1IP1	filled
ENST00000510616	ENSG00000153006	SREK1IP1	filled

ENST00000506252	ENSG00000153006	SREK1IP1	filled
ENST00000622404	ENSG00000153006	SREK1IP1	filled
ENST00000427576	ENSG00000061938	TNK2	
ENST00000552301	ENSG00000110844	PRPF40B	
ENST00000547764	ENSG00000110844	PRPF40B	
ENST00000551063	ENSG00000110844	PRPF40B	
ENST00000527253	ENSG00000110844	PRPF40B	
ENST00000508736	ENSG00000110844	PRPF40B	
ENST00000419318	ENSG00000196466	ZNF799	filled
ENST00000254301	ENSG00000131981	LGALS3	
ENST00000467203	ENSG00000066136	NFYC	filled
ENST00000424419	ENSG00000066136	NFYC	filled
ENST00000427410	ENSG00000066136	NFYC	filled
ENST00000447388	ENSG00000066136	NFYC	filled
ENST00000425457	ENSG00000066136	NFYC	filled
ENST00000453631	ENSG00000066136	NFYC	filled
ENST00000456393	ENSG00000066136	NFYC	filled
ENST00000372654	ENSG00000066136	NFYC	filled
ENST00000534399	ENSG00000066136	NFYC	filled
ENST00000372669	ENSG00000066136	NFYC	filled
ENST00000372652	ENSG00000066136	NFYC	filled
ENST00000372651	ENSG00000066136	NFYC	filled
ENST00000531464	ENSG00000066136	NFYC	filled
ENST00000620728	ENSG00000066136	NFYC	filled
ENST00000542011	ENSG00000176834	VSIG10	filled
ENST00000438826	ENSG00000175106	TVP23C	filled
ENST00000428082	ENSG00000175106	TVP23C	filled
ENST00000518321	ENSG00000175106	TVP23C	filled
ENST00000590221	ENSG00000132471	WBP2	
ENST00000356151	ENSG00000168297	PXK	filled
ENST00000468776	ENSG00000168297	PXK	filled
ENST00000463280	ENSG00000168297	PXK	filled
ENST00000383715	ENSG00000168297	PXK	filled
ENST00000484288	ENSG00000168297	PXK	filled
ENST00000479241	ENSG00000168297	PXK	filled
ENST00000477308	ENSG00000168297	PXK	filled
ENST00000491164	ENSG00000168297	PXK	filled
ENST00000383716	ENSG00000168297	PXK	filled
ENST00000302779	ENSG00000168297	PXK	filled
ENST00000605932	ENSG00000154814	OXNAD1	filled
ENST00000435829	ENSG00000154814	OXNAD1	filled
ENST00000606098	ENSG00000154814	OXNAD1	filled
ENST00000458234	ENSG00000196498	NCOR2	filled
ENST00000420698	ENSG00000196498	NCOR2	filled
ENST00000542565	ENSG00000196498	NCOR2	filled
ENST00000520044	ENSG00000270024	C8orf44-SGK3	filled
ENST00000519289	ENSG00000270024	C8orf44-SGK3	filled
ENST00000252071	ENSG00000106526	ACTR3C	filled
ENST00000342526	ENSG00000187546	AGMO	filled
ENST00000227756	ENSG00000110328	GALNT18	filled
ENST00000526064	ENSG00000110328	GALNT18	filled
ENST00000452098	ENSG00000151093	OXSM	
ENST00000449808	ENSG00000151093	OXSM	
ENST00000488998	ENSG00000135205	CCDC146	filled
ENST00000290374	ENSG00000159248	GJD2	
ENST00000432071	ENSG00000118640	VAMP8	
ENST00000367807	ENSG00000117475	BLZF1	
ENST00000367808	ENSG00000117475	BLZF1	
ENST00000329281	ENSG00000117475	BLZF1	
ENST00000420531	ENSG00000117475	BLZF1	

ENST00000426663	ENSG00000117475	BLZF1	
ENST00000510679	ENSG00000172062	SMN1	filled
ENST00000491839	ENSG00000172986	GXYLT2	filled
ENST00000403625	ENSG00000069020	MAST4	filled
ENST00000490016	ENSG00000069020	MAST4	filled
ENST00000403666	ENSG00000069020	MAST4	filled
ENST00000405643	ENSG00000069020	MAST4	filled
ENST00000432426	ENSG00000069020	MAST4	filled
ENST00000371080	ENSG00000185483	ROR1	filled
ENST00000371079	ENSG00000185483	ROR1	filled
ENST00000482426	ENSG00000185483	ROR1	filled
ENST00000509851	ENSG00000163749	CCDC158	filled
ENST00000504868	ENSG00000163749	CCDC158	filled
ENST00000351989	ENSG00000128191	DGCR8	
ENST00000383024	ENSG00000128191	DGCR8	
ENST00000327720	ENSG00000184302	SIX6	
ENST00000497505	ENSG00000144426	NBEAL1	filled
ENST00000441007	ENSG00000156976	EIF4A2	
ENST00000426808	ENSG00000156976	EIF4A2	
ENST00000440191	ENSG00000156976	EIF4A2	
ENST00000443963	ENSG00000156976	EIF4A2	
ENST00000421879	ENSG00000149485	FADS1	filled
ENST00000448607	ENSG00000149485	FADS1	filled
ENST00000426755	ENSG00000163964	PIGX	filled
ENST00000502288	ENSG00000080822	CLDND1	
ENST00000507874	ENSG00000080822	CLDND1	
ENST00000506927	ENSG00000080822	CLDND1	
ENST00000371225	ENSG00000184292	TACSTD2	
ENST00000326665	ENSG00000178965	ERICH3	filled
ENST00000433746	ENSG00000178965	ERICH3	
ENST00000547408	ENSG00000092841	MYL6	filled
ENST00000546845	ENSG00000092841	MYL6	filled
ENST00000551954	ENSG00000092841	MYL6	
ENST00000529805	ENSG00000170903	MSANTD4	
ENST00000461923	ENSG00000204889	KRT40	
ENST00000398486	ENSG00000204889	KRT40	
ENST00000355035	ENSG00000161944	ASGR2	filled
ENST00000254850	ENSG00000161944	ASGR2	filled
ENST00000446679	ENSG00000161944	ASGR2	filled
ENST00000523949	ENSG00000104765	BNIP3L	filled
ENST00000570914	ENSG00000141504	SAT2	
ENST00000374469	ENSG00000165124	SVEP1	filled
ENST00000401783	ENSG00000165124	SVEP1	filled
ENST00000290295	ENSG00000159184	HOXB13	
ENST00000338465	ENSG00000135913	USP37	filled
ENST00000399084	ENSG00000179344	HLA-DQB1	
ENST00000563128	ENSG00000166947	EPB42	filled
ENST00000539745	ENSG00000197837	HIST4H4	
ENST00000546783	ENSG00000173588	CEP83	filled
ENST00000297071	ENSG00000164548	TRA2A	
ENST00000494255	ENSG00000164548	TRA2A	
ENST00000474586	ENSG00000164548	TRA2A	
ENST00000490942	ENSG00000164548	TRA2A	
ENST00000448549	ENSG00000164548	TRA2A	
ENST00000392502	ENSG00000164548	TRA2A	
ENST00000621813	ENSG00000164548	TRA2A	
ENST00000538367	ENSG00000164548	TRA2A	
ENST00000541806	ENSG00000165376	CLDN2	
ENST00000481461	ENSG00000106397	PLOD3	
ENST00000494455	ENSG00000124251	TP53TG5	

ENST00000488588	ENSG00000124251	TP53TG5	
ENST00000468375	ENSG00000124251	TP53TG5	
ENST00000478216	ENSG00000056586	RC3H2	
ENST00000519715	ENSG00000170458	CD14	
ENST00000512545	ENSG00000170458	CD14	
ENST00000200453	ENSG00000087074	PPP1R15A	filled
ENST00000298912	ENSG00000165959	CLMN	filled
ENST00000555336	ENSG00000165959	CLMN	filled
ENST00000555615	ENSG00000165959	CLMN	filled
ENST00000371754	ENSG00000124201	ZNFX1	
ENST00000469991	ENSG00000124201	ZNFX1	
ENST00000390658	ENSG00000176396	EID2	
ENST00000283635	ENSG00000153563	CD8A	
ENST00000409511	ENSG00000153563	CD8A	filled
ENST00000553066	ENSG00000196465	MYL6B	
ENST00000550443	ENSG00000196465	MYL6B	
ENST00000551834	ENSG00000196465	MYL6B	
ENST00000605847	ENSG00000255526	NEDD8-MDP1	
ENST00000534348	ENSG00000255526	NEDD8-MDP1	
ENST00000530579	ENSG00000255526	NEDD8-MDP1	
ENST00000604306	ENSG00000255526	NEDD8-MDP1	
ENST00000623677	ENSG00000232258	TMEM114	filled
ENST00000332290	ENSG00000185127	C6orf120	
ENST00000423807	ENSG00000187024	PTRH1	
ENST00000419060	ENSG00000187024	PTRH1	filled
ENST00000369454	ENSG00000155961	RAB39B	
ENST00000409774	ENSG00000152133	GPATCH11	
ENST00000281932	ENSG00000152133	GPATCH11	
ENST00000470671	ENSG00000198034	RPS4X	
ENST00000492695	ENSG00000198034	RPS4X	
ENST00000522111	ENSG00000253368	TRNP1	
ENST00000451963	ENSG00000066923	STAG3	
ENST00000556675	ENSG00000089916	GPATCH2L	filled
ENST00000596459	ENSG00000066044	ELAVL1	
ENST00000351593	ENSG00000066044	ELAVL1	
ENST00000397527	ENSG00000126001	CEP250	
ENST00000587915	ENSG00000064545	TMEM161A	
ENST00000257940	ENSG00000135482	ZC3H10	
ENST00000324000	ENSG00000177511	ST8SIA3	
ENST00000586360	ENSG00000177511	ST8SIA3	
ENST00000419452	ENSG00000235844	HLA-DPA1	
ENST00000377982	ENSG00000138071	ACTR2	
ENST00000511051	ENSG00000152990	GPR125	filled
ENST00000552377	ENSG00000133773	CCDC59	filled
ENST00000612688	ENSG00000275052	SMEK2	
ENST00000534831	ENSG00000013588	GPRC5A	
ENST00000440599	ENSG00000151704	KCNJ1	filled
ENST00000324036	ENSG00000151704	KCNJ1	filled
ENST00000392664	ENSG00000151704	KCNJ1	
ENST00000324003	ENSG00000151704	KCNJ1	filled
ENST00000531562	ENSG00000151704	KCNJ1	filled
ENST00000392666	ENSG00000151704	KCNJ1	filled
ENST00000569080	ENSG00000062038	CDH3	
ENST00000475997	ENSG00000277877	RP11-11N7.5	
ENST00000489705	ENSG00000277877	RP11-11N7.5	
ENST00000286437	ENSG00000155966	AFF2	
ENST00000517980	ENSG00000044115	CTNNA1	filled
ENST00000522227	ENSG00000044115	CTNNA1	filled
ENST00000524127	ENSG00000044115	CTNNA1	filled
ENST00000518910	ENSG00000044115	CTNNA1	filled
ENST00000520520	ENSG00000044115	CTNNA1	
ENST00000597498	ENSG00000086967	MYBPC2	
ENST00000289672	ENSG00000158683	PKD1L1	filled
ENST00000304411	ENSG00000170837	GPR27	
ENST00000373368	ENSG00000072080	SPP2	filled
ENST00000168148	ENSG00000072080	SPP2	filled
ENST00000492481	ENSG00000072080	SPP2	filled
ENST00000383472	ENSG00000088756	ARHGAP28	filled
ENST00000583410	ENSG00000088756	ARHGAP28	
ENST00000584387	ENSG00000088756	ARHGAP28	
ENST00000532723	ENSG00000088756	ARHGAP28	
ENST00000262227	ENSG00000088756	ARHGAP28	
ENST00000560916	ENSG00000172345	STARD5	
ENST00000551790	ENSG00000139641	ESYT1	
ENST00000563730	ENSG00000129636	ITFG1	
ENST00000367876	ENSG00000143157	POGK	
ENST00000367875	ENSG00000143157	POGK	
ENST00000494626	ENSG00000175029	CTBP2	
ENST00000433011	ENSG00000196440	ARMCX4	filled
ENST00000430461	ENSG00000196440	ARMCX4	
ENST00000453574	ENSG00000196440	ARMCX4	
ENST00000452188	ENSG00000196440	ARMCX4	filled
ENST00000455331	ENSG00000196440	ARMCX4	
ENST00000354842	ENSG00000196440	ARMCX4	filled
ENST00000584689	ENSG00000141526	SLC16A3	
ENST00000583237	ENSG00000141526	SLC16A3	
ENST00000580098	ENSG00000141526	SLC16A3	
ENST00000583025	ENSG00000141526	SLC16A3	
ENST00000392339	ENSG00000141526	SLC16A3	
ENST00000392341	ENSG00000141526	SLC16A3	
ENST00000340635	ENSG00000113448	PDE4D	filled
ENST00000360047	ENSG00000113448	PDE4D	filled
ENST00000507116	ENSG00000113448	PDE4D	filled
ENST00000502484	ENSG00000113448	PDE4D	filled
ENST00000309641	ENSG00000113448	PDE4D	filled
ENST00000502575	ENSG00000113448	PDE4D	filled
ENST00000512069	ENSG00000113448	PDE4D	filled
ENST00000509368	ENSG00000113448	PDE4D	filled
ENST00000509355	ENSG00000113448	PDE4D	filled
ENST00000504624	ENSG00000113448	PDE4D	
ENST00000506510	ENSG00000113448	PDE4D	filled
ENST00000511382	ENSG00000113448	PDE4D	filled
ENST00000505507	ENSG00000113448	PDE4D	filled
ENST00000514552	ENSG00000113448	PDE4D	filled
ENST00000515835	ENSG00000113448	PDE4D	filled
ENST00000531674	ENSG00000167207	NOD2	filled
ENST00000300589	ENSG00000167207	NOD2	filled
ENST00000524712	ENSG00000167207	NOD2	
ENST00000527052	ENSG00000167207	NOD2	
ENST00000460710	ENSG00000153246	PLA2R1	
ENST00000554908	ENSG00000197045	GMFB	
ENST00000358056	ENSG00000197045	GMFB	
ENST00000554163	ENSG00000197045	GMFB	
ENST00000616146	ENSG00000197045	GMFB	
ENST00000472748	ENSG00000115840	SLC25A12	filled
ENST00000484227	ENSG00000115840	SLC25A12	filled
ENST00000464063	ENSG00000115840	SLC25A12	filled
ENST00000604922	ENSG00000109686	SH3D19	filled
ENST00000604030	ENSG00000109686	SH3D19	filled
ENST00000512351	ENSG00000168214	RBPJ	filled

ENST00000510778	ENSG00000168214	RBPJ	filled
ENST00000278947	ENSG00000149575	SCN2B	
ENST00000396081	ENSG00000122557	HERPUD2	filled
ENST00000311350	ENSG00000122557	HERPUD2	filled
ENST00000438224	ENSG00000122557	HERPUD2	
ENST00000413517	ENSG00000122557	HERPUD2	
ENST00000427455	ENSG00000122557	HERPUD2	
ENST00000416904	ENSG00000139651	ZNF740	
ENST00000479309	ENSG00000152253	SPC25	
ENST00000282074	ENSG00000152253	SPC25	filled
ENST00000418597	ENSG00000130413	STK33	filled
ENST00000422559	ENSG00000130413	STK33	filled
ENST00000526360	ENSG00000130413	STK33	filled
ENST00000370552	ENSG00000172987	HPSE2	filled
ENST00000370549	ENSG00000172987	HPSE2	filled
ENST00000370546	ENSG00000172987	HPSE2	filled
ENST00000614306	ENSG00000172987	HPSE2	filled
ENST00000529577	ENSG00000087365	SF3B2	
ENST00000471446	ENSG00000105829	BET1	
ENST00000576342	ENSG00000261992	GATAD2B	filled
ENST00000470166	ENSG00000121905	HPCA	
ENST00000373467	ENSG00000121905	HPCA	
ENST00000459874	ENSG00000121905	HPCA	
ENST00000470896	ENSG00000121905	HPCA	
ENST00000555677	ENSG00000119650	IFT43	filled
ENST00000596341	ENSG00000083838	ZNF446	
ENST00000569294	ENSG00000205084	TMEM231	
ENST00000524171	ENSG00000234511	C5orf58	
ENST00000557430	ENSG00000151812	SLC35F4	
ENST00000556568	ENSG00000151812	SLC35F4	filled
ENST00000554648	ENSG00000151812	SLC35F4	
ENST00000546655	ENSG00000182544	MFSD5	
ENST00000551660	ENSG00000182544	MFSD5	
ENST00000506207	ENSG00000169567	HINT1	filled
ENST00000286307	ENSG00000155858	LSM11	filled
ENST00000461237	ENSG00000126860	EVI2A	
ENST00000462804	ENSG00000126860	EVI2A	
ENST00000561945	ENSG00000197912	SPG7	
ENST00000563218	ENSG00000197912	SPG7	
ENST00000561911	ENSG00000197912	SPG7	
ENST00000592349	ENSG00000141431	ASXL3	
ENST00000586948	ENSG00000141431	ASXL3	
ENST00000431946	ENSG00000135596	MICAL1	
ENST00000523454	ENSG00000184661	CDCA2	
ENST00000332378	ENSG00000182591	KRTAP11-1	
ENST00000450537	ENSG00000196700	ZNF512B	filled
ENST00000529580	ENSG00000175467	SART1	
ENST00000400546	ENSG00000154654	NCAM2	filled
ENST00000311083	ENSG00000175183	CSRP2	
ENST00000546966	ENSG00000175183	CSRP2	
ENST00000520138	ENSG00000226257	STK19	filled
ENST00000272520	ENSG00000144119	C1QL2	
ENST00000520720	ENSG00000069206	ADAM7	filled
ENST00000556799	ENSG00000179008	C14orf39	
ENST00000488423	ENSG00000154813	DPH3	filled
ENST00000383775	ENSG00000154813	DPH3	filled
ENST00000305432	ENSG00000168959	GRM5	filled
ENST00000305447	ENSG00000168959	GRM5	filled
ENST00000393294	ENSG00000168959	GRM5	
ENST00000455756	ENSG00000168959	GRM5	filled

ENST00000487831	ENSG00000112159	MDN1	
ENST00000383238	ENSG00000206298	PSMB8	filled
ENST00000560241	ENSG00000172349	IL16	filled
ENST00000360547	ENSG00000172349	IL16	filled
ENST00000559383	ENSG00000172349	IL16	filled
ENST00000394660	ENSG00000172349	IL16	filled
ENST00000428084	ENSG00000128512	DOCK4	filled
ENST00000437633	ENSG00000128512	DOCK4	filled
ENST00000476846	ENSG00000128512	DOCK4	filled
ENST00000557349	ENSG00000259024	TVP23C-CDRT4	filled
ENST00000481756	ENSG00000259024	TVP23C-CDRT4	filled
ENST00000518506	ENSG00000259024	TVP23C-CDRT4	filled
ENST00000597927	ENSG00000197134	ZNF257	filled
ENST00000600162	ENSG00000197134	ZNF257	filled
ENST00000486108	ENSG00000128271	ADORA2A	filled
ENST00000607039	ENSG00000143079	CTTNBP2NL	
ENST00000530047	ENSG00000185803	SLC52A2	
ENST00000527078	ENSG00000185803	SLC52A2	
ENST00000534725	ENSG00000185803	SLC52A2	
ENST00000532887	ENSG00000185803	SLC52A2	
ENST00000329994	ENSG00000185803	SLC52A2	
ENST00000230882	ENSG00000112964	GHR	filled
ENST00000505006	ENSG00000112964	GHR	filled
ENST00000615111	ENSG00000112964	GHR	filled
ENST00000620156	ENSG00000112964	GHR	filled
ENST00000548938	ENSG00000061273	HDAC7	
ENST00000427332	ENSG00000061273	HDAC7	filled
ENST00000422254	ENSG00000061273	HDAC7	filled
ENST00000447463	ENSG00000061273	HDAC7	filled
ENST00000434070	ENSG00000061273	HDAC7	filled
ENST00000421231	ENSG00000061273	HDAC7	filled
ENST00000433685	ENSG00000061273	HDAC7	filled
ENST00000367903	ENSG00000143248	RGS5	
ENST00000531954	ENSG00000143248	RGS5	filled
ENST00000534288	ENSG00000143248	RGS5	filled
ENST00000618415	ENSG00000143248	RGS5	filled
ENST00000521808	ENSG00000129691	ASH2L	filled
ENST00000295190	ENSG00000163053	SLC16A14	filled
ENST00000457406	ENSG00000163053	SLC16A14	filled
ENST00000412034	ENSG00000163053	SLC16A14	filled
ENST00000436869	ENSG00000163053	SLC16A14	
ENST00000397996	ENSG00000242550	SERPINB10	
ENST00000418725	ENSG00000242550	SERPINB10	
ENST00000590722	ENSG00000267699	RP11-729L2.2	filled
ENST00000444495	ENSG00000145191	EIF2B5	filled
ENST00000306897	ENSG00000169750	RAC3	
ENST00000415796	ENSG00000206306	HLA-DRB1	filled
ENST00000502658	ENSG00000164182	NDUFAF2	
ENST00000320354	ENSG00000176692	FOXC2	filled
ENST00000294661	ENSG00000162642	C1orf52	filled
ENST00000259455	ENSG00000136928	GABBR2	filled
ENST00000477471	ENSG00000136928	GABBR2	filled
ENST00000378670	ENSG00000164405	UQCRQ	
ENST00000218004	ENSG00000101888	NXT2	
ENST00000372106	ENSG00000101888	NXT2	
ENST00000434752	ENSG00000228672	PROB1	
ENST00000382952	ENSG00000159692	CTBP1	
ENST00000290921	ENSG00000159692	CTBP1	
ENST00000513420	ENSG00000159692	CTBP1	filled
ENST00000524833	ENSG00000075239	ACAT1	

ENST00000299355	ENSG00000075239	ACAT1	
ENST00000560117	ENSG00000021776	AQR	filled
ENST00000393215	ENSG00000188523	C9orf171	filled
ENST00000508780	ENSG00000173221	GLRX	
ENST00000575403	ENSG00000108641	B9D1	filled
ENST00000477478	ENSG00000108641	B9D1	filled
ENST00000487415	ENSG00000108641	B9D1	
ENST00000478136	ENSG00000183662	FAM19A1	filled
ENST00000496687	ENSG00000183662	FAM19A1	filled
ENST00000377560	ENSG00000173064	HECTD4	filled
ENST00000550724	ENSG00000173064	HECTD4	filled
ENST00000550722	ENSG00000173064	HECTD4	filled
ENST00000368750	ENSG00000203785	SPRR2E	filled
ENST00000602566	ENSG00000134250	NOTCH2	filled
ENST00000612822	ENSG00000134250	NOTCH2	filled
ENST00000579475	ENSG00000134250	NOTCH2	filled
ENST00000572145	ENSG00000167193	CRK	filled
ENST00000521162	ENSG00000155975	VPS37A	
ENST00000451916	ENSG00000162994	CLHC1	filled
ENST00000463300	ENSG00000162994	CLHC1	filled
ENST00000403506	ENSG00000162994	CLHC1	
ENST00000465656	ENSG00000188033	ZNF490	filled
ENST00000614979	ENSG00000143195	ILDR2	
ENST00000271417	ENSG00000143195	ILDR2	filled
ENST00000528703	ENSG00000143195	ILDR2	filled
ENST00000525740	ENSG00000143195	ILDR2	filled
ENST00000529387	ENSG00000143195	ILDR2	filled
ENST00000469934	ENSG00000143195	ILDR2	filled
ENST00000529071	ENSG00000143195	ILDR2	filled
ENST00000526687	ENSG00000143195	ILDR2	filled
ENST00000522578	ENSG00000131773	KHDRBS3	
ENST00000358654	ENSG00000258555	SPECC1L-ADORA	filled
ENST00000377767	ENSG00000083520	DIS3	
ENST00000521228	ENSG00000159398	CES5A	
ENST00000521992	ENSG00000159398	CES5A	filled
ENST00000319165	ENSG00000159398	CES5A	
ENST00000518005	ENSG00000159398	CES5A	filled
ENST00000290567	ENSG00000159398	CES5A	
ENST00000520435	ENSG00000159398	CES5A	
ENST00000541580	ENSG00000159398	CES5A	filled
ENST00000418331	ENSG00000149177	PTPRJ	filled
ENST00000534219	ENSG00000149177	PTPRJ	filled
ENST00000613246	ENSG00000149177	PTPRJ	filled
ENST00000615445	ENSG00000149177	PTPRJ	filled
ENST00000596049	ENSG00000104872	PIH1D1	
ENST00000599366	ENSG00000104872	PIH1D1	
ENST00000597415	ENSG00000104872	PIH1D1	
ENST00000601825	ENSG00000104872	PIH1D1	
ENST00000402676	ENSG00000131016	AKAP12	filled
ENST00000453961	ENSG00000237344	PBX2	filled
ENST00000489088	ENSG00000120699	EXOSC8	filled
ENST00000395878	ENSG00000103353	UBFD1	
ENST00000567212	ENSG00000103353	UBFD1	
ENST00000563366	ENSG00000103353	UBFD1	
ENST00000565634	ENSG00000103353	UBFD1	
ENST00000564106	ENSG00000103353	UBFD1	
ENST00000566136	ENSG00000103353	UBFD1	
ENST00000383657	ENSG00000206527	PTPLB	
ENST00000469317	ENSG00000206527	PTPLB	filled
ENST00000618916	ENSG00000196072	BLOC1S2	filled

ENST00000473119	ENSG00000133114	GPALPP1	
ENST00000440025	ENSG00000005471	ABCB4	
ENST00000473795	ENSG00000005471	ABCB4	
ENST00000583912	ENSG00000177885	GRB2	filled
ENST00000374694	ENSG00000177283	FZD8	
ENST00000315251	ENSG00000016391	CHDH	filled
ENST00000309352	ENSG00000204316	MRPL38	filled
ENST00000486101	ENSG00000204316	MRPL38	filled
ENST00000480203	ENSG00000204316	MRPL38	filled
ENST00000588620	ENSG00000204316	MRPL38	filled
ENST00000483393	ENSG00000204316	MRPL38	filled
ENST00000477371	ENSG00000204316	MRPL38	filled
ENST00000477023	ENSG00000204316	MRPL38	filled
ENST00000511112	ENSG00000138650	PCDH10	filled
ENST00000472944	ENSG00000134717	BTF3L4	
ENST00000305892	ENSG00000171936	OR10H3	
ENST00000478300	ENSG00000163630	SYNPR	filled
ENST00000450542	ENSG00000163630	SYNPR	filled
ENST00000468110	ENSG00000163630	SYNPR	filled
ENST00000496889	ENSG00000163630	SYNPR	filled
ENST00000460142	ENSG00000163630	SYNPR	filled
ENST00000493532	ENSG00000163630	SYNPR	filled
ENST00000514089	ENSG00000159210	SNF8	
ENST00000576353	ENSG00000159210	SNF8	
ENST00000507302	ENSG00000159210	SNF8	
ENST00000447351	ENSG00000075240	GRAMD4	filled
ENST00000486777	ENSG00000135631	RAB11FIP5	
ENST00000482554	ENSG00000135631	RAB11FIP5	
ENST00000593625	ENSG00000176678	FOXL1	
ENST00000434040	ENSG00000198646	NCOA6	
ENST00000593786	ENSG00000198646	NCOA6	
ENST00000550668	ENSG00000161791	FMNL3	
ENST00000435848	ENSG00000214253	FIS1	filled
ENST00000529359	ENSG00000147535	PPAPDC1B	filled
ENST00000424479	ENSG00000147535	PPAPDC1B	filled
ENST00000530193	ENSG00000147535	PPAPDC1B	filled
ENST00000566914	ENSG00000140859	KIFC3	
ENST00000563028	ENSG00000140859	KIFC3	filled
ENST00000331683	ENSG00000144451	SPAG16	filled
ENST00000406979	ENSG00000144451	SPAG16	filled
ENST00000452556	ENSG00000144451	SPAG16	filled
ENST00000451561	ENSG00000144451	SPAG16	filled
ENST00000480494	ENSG00000144451	SPAG16	filled
ENST00000401432	ENSG00000084710	EFR3B	filled
ENST00000403714	ENSG00000084710	EFR3B	filled
ENST00000402191	ENSG00000084710	EFR3B	filled
ENST00000515393	ENSG00000175471	MCTP1	filled
ENST00000503301	ENSG00000175471	MCTP1	filled
ENST00000513695	ENSG00000175471	MCTP1	filled
ENST00000404622	ENSG00000197632	SERPINB2	
ENST00000567871	ENSG00000178814	OPLAH	
ENST00000515763	ENSG00000127184	COX7C	
ENST00000465142	ENSG00000114405	C3orf14	
ENST00000521943	ENSG00000198586	TLK1	filled
ENST00000556241	ENSG00000119686	FLVCR2	filled
ENST00000553587	ENSG00000119686	FLVCR2	filled
ENST00000555529	ENSG00000100629	CEP128	filled
ENST00000323482	ENSG00000154930	ACSS1	filled
ENST00000432802	ENSG00000154930	ACSS1	filled
ENST00000462024	ENSG00000160908	ZNF394	

ENST00000396193	ENSG00000154678	PDE1C	filled
ENST00000510962	ENSG00000131446	MGAT1	
ENST00000402506	ENSG00000106003	LFNG	
ENST00000577616	ENSG00000146872	TLK2	filled
ENST00000460934	ENSG00000158715	SLC45A3	
ENST00000565786	ENSG00000005194	CIAPIN1	
ENST00000252329	ENSG00000157778	PSMG3	filled
ENST00000481617	ENSG00000133138	TBC1D8B	filled
ENST00000589938	ENSG00000197213	ZSCAN5B	filled
ENST00000518342	ENSG00000174226	SNX31	
ENST00000483386	ENSG00000176273	SLC35G1	
ENST00000603665	ENSG00000176273	SLC35G1	
ENST00000476718	ENSG00000162576	MXRA8	
ENST00000464351	ENSG00000162576	MXRA8	
ENST00000327245	ENSG00000118322	ATP10B	filled
ENST00000521033	ENSG00000118322	ATP10B	filled
ENST00000518411	ENSG00000118322	ATP10B	filled
ENST00000549481	ENSG00000063046	EIF4B	
ENST00000552490	ENSG00000063046	EIF4B	
ENST00000358387	ENSG00000113621	TXNDC15	
ENST00000511070	ENSG00000113621	TXNDC15	
ENST00000506916	ENSG00000113621	TXNDC15	
ENST00000508779	ENSG00000113621	TXNDC15	
ENST00000506350	ENSG00000113621	TXNDC15	
ENST00000505174	ENSG00000113621	TXNDC15	
ENST00000509954	ENSG00000113621	TXNDC15	
ENST00000578584	ENSG00000265118	CTD-2370N5.3	
ENST00000578021	ENSG00000265118	CTD-2370N5.3	
ENST00000584948	ENSG00000265118	CTD-2370N5.3	
ENST00000581884	ENSG00000265118	CTD-2370N5.3	
ENST00000504376	ENSG00000154162	CDH12	filled
ENST00000382254	ENSG00000154162	CDH12	filled
ENST00000468300	ENSG00000012048	BRCA1	filled
ENST00000552430	ENSG00000050426	LETMD1	filled
ENST00000550755	ENSG00000050426	LETMD1	filled
ENST00000547008	ENSG00000050426	LETMD1	filled
ENST00000601953	ENSG00000182472	CAPN12	
ENST00000506823	ENSG00000174473	GALNTL6	filled
ENST00000508122	ENSG00000174473	GALNTL6	filled
ENST00000457021	ENSG00000174473	GALNTL6	filled
ENST00000445009	ENSG00000231617	DAXX	
ENST00000433482	ENSG00000231617	DAXX	
ENST00000485010	ENSG00000231617	DAXX	
ENST00000467554	ENSG00000231617	DAXX	
ENST00000453931	ENSG00000231617	DAXX	filled
ENST00000469788	ENSG00000231617	DAXX	
ENST00000468906	ENSG00000231617	DAXX	
ENST00000454197	ENSG00000231617	DAXX	
ENST00000469277	ENSG00000231617	DAXX	
ENST00000451280	ENSG00000231617	DAXX	
ENST00000622655	ENSG00000231617	DAXX	
ENST00000538780	ENSG00000139438	FAM222A	filled
ENST00000543653	ENSG00000139438	FAM222A	filled
ENST00000358906	ENSG00000139438	FAM222A	filled
ENST00000532400	ENSG00000104529	EEF1D	
ENST00000526838	ENSG00000104529	EEF1D	
ENST00000528610	ENSG00000104529	EEF1D	
ENST00000533833	ENSG00000104529	EEF1D	
ENST00000529007	ENSG00000104529	EEF1D	
ENST00000531621	ENSG00000104529	EEF1D	

ENST00000534377	ENSG00000104529	EEF1D	
ENST00000529516	ENSG00000104529	EEF1D	
ENST00000556137	ENSG00000139926	FRMD6	filled
ENST00000356218	ENSG00000139926	FRMD6	filled
ENST00000555952	ENSG00000139926	FRMD6	
ENST00000554745	ENSG00000139926	FRMD6	filled
ENST00000395718	ENSG00000139926	FRMD6	filled
ENST00000554778	ENSG00000139926	FRMD6	
ENST00000622874	ENSG00000143194	MAEL	
ENST00000478530	ENSG00000106785	TRIM14	
ENST00000517522	ENSG00000038945	MSR1	
ENST00000477068	ENSG00000135899	SP110	
ENST00000622985	ENSG00000275763	C18orf65	
ENST00000618178	ENSG00000275763	C18orf65	
ENST00000522056	ENSG00000134824	FADS2	filled
ENST00000410046	ENSG00000138311	ZNF365	filled
ENST00000598018	ENSG00000104888	SLC17A7	filled
ENST00000401710	ENSG00000179915	NRXN1	filled
ENST00000404971	ENSG00000179915	NRXN1	filled
ENST00000406316	ENSG00000179915	NRXN1	filled
ENST00000405472	ENSG00000179915	NRXN1	filled
ENST00000401669	ENSG00000179915	NRXN1	filled
ENST00000331040	ENSG00000179915	NRXN1	filled
ENST00000405581	ENSG00000179915	NRXN1	filled
ENST00000496792	ENSG00000179915	NRXN1	filled
ENST00000320460	ENSG00000180481	GLIPR1L2	filled
ENST00000538846	ENSG00000182450	KCNK4	
ENST00000538767	ENSG00000182450	KCNK4	
ENST00000422670	ENSG00000182450	KCNK4	
ENST00000260443	ENSG00000137876	RSL24D1	
ENST00000492177	ENSG00000231737	PPP1R10	
ENST00000537792	ENSG00000118557	PMFBP1	
ENST00000540440	ENSG00000118557	PMFBP1	
ENST00000346991	ENSG00000137812	CASC5	filled
ENST00000399668	ENSG00000137812	CASC5	filled
ENST00000526913	ENSG00000137812	CASC5	filled
ENST00000532406	ENSG00000137812	CASC5	filled
ENST00000528967	ENSG00000137812	CASC5	
ENST00000539463	ENSG00000139726	DENR	filled
ENST00000537955	ENSG00000139726	DENR	
ENST00000605860	ENSG00000007001	UPP2	filled
ENST00000460456	ENSG00000007001	UPP2	filled
ENST00000005756	ENSG00000007001	UPP2	filled
ENST00000489438	ENSG00000007001	UPP2	filled
ENST00000452403	ENSG00000115594	IL1R1	filled
ENST00000409929	ENSG00000115594	IL1R1	filled
ENST00000424272	ENSG00000115594	IL1R1	filled
ENST00000409329	ENSG00000115594	IL1R1	filled
ENST00000428279	ENSG00000115594	IL1R1	filled
ENST00000442590	ENSG00000115594	IL1R1	filled
ENST00000458229	ENSG00000155380	SLC16A1	filled
ENST00000429288	ENSG00000155380	SLC16A1	filled
ENST00000538576	ENSG00000155380	SLC16A1	filled
ENST00000521096	ENSG00000104381	GDAP1	
ENST00000520797	ENSG00000104381	GDAP1	
ENST00000524195	ENSG00000104381	GDAP1	filled
ENST00000562635	ENSG00000140367	UBE2Q2	
ENST00000563966	ENSG00000140367	UBE2Q2	filled
ENST00000373874	ENSG00000118707	TGIF2	
ENST00000373872	ENSG00000118707	TGIF2	

ENST00000611732	ENSG00000118707	TGIF2	
ENST00000560313	ENSG00000090470	PDCD7	
ENST00000495879	ENSG00000144724	PTPRG	filled
ENST00000475527	ENSG00000144724	PTPRG	filled
ENST00000295874	ENSG00000144724	PTPRG	filled
ENST00000474889	ENSG00000144724	PTPRG	filled
ENST00000534289	ENSG00000143228	NUF2	filled
ENST00000522613	ENSG00000184672	RALYL	filled
ENST00000522455	ENSG00000184672	RALYL	filled
ENST00000521695	ENSG00000184672	RALYL	filled
ENST00000521268	ENSG00000184672	RALYL	filled
ENST00000518566	ENSG00000184672	RALYL	filled
ENST00000517988	ENSG00000184672	RALYL	filled
ENST00000517638	ENSG00000184672	RALYL	filled
ENST00000522647	ENSG00000184672	RALYL	filled
ENST00000563310	ENSG00000166233	ARIH1	
ENST00000591728	ENSG00000081923	ATP8B1	filled
ENST00000536015	ENSG00000081923	ATP8B1	filled
ENST00000382564	ENSG00000205981	DNAJC19	
ENST00000469657	ENSG00000205981	DNAJC19	
ENST00000491873	ENSG00000205981	DNAJC19	
ENST00000569223	ENSG00000103966	EHD4	filled
ENST00000441120	ENSG00000116791	CRYZ	
ENST00000374580	ENSG00000204217	BMPR2	filled
ENST00000374574	ENSG00000204217	BMPR2	filled
ENST00000379607	ENSG00000173674	EIF1AX	
ENST00000360000	ENSG00000180964	TCEAL8	
ENST00000372685	ENSG00000180964	TCEAL8	
ENST00000451678	ENSG00000180964	TCEAL8	
ENST00000400000	ENSG00000101558	VAPA	filled
ENST00000340541	ENSG00000101558	VAPA	filled
ENST00000585042	ENSG00000101558	VAPA	filled
ENST00000321639	ENSG00000181291	TMEM132E	filled
ENST00000441551	ENSG00000167210	LOXHD1	filled
ENST00000536736	ENSG00000167210	LOXHD1	filled
ENST00000498382	ENSG00000143786	CNIH3	filled
ENST00000474165	ENSG00000144815	NXPE3	filled
ENST00000491511	ENSG00000144815	NXPE3	filled
ENST00000358487	ENSG00000066468	FGFR2	filled
ENST00000356226	ENSG00000066468	FGFR2	filled
ENST00000369060	ENSG00000066468	FGFR2	filled
ENST00000604236	ENSG00000066468	FGFR2	filled
ENST00000369059	ENSG00000066468	FGFR2	filled
ENST00000346997	ENSG00000066468	FGFR2	filled
ENST00000457416	ENSG00000066468	FGFR2	filled
ENST00000369058	ENSG00000066468	FGFR2	filled
ENST00000336553	ENSG00000066468	FGFR2	filled
ENST00000490349	ENSG00000066468	FGFR2	filled
ENST00000613324	ENSG00000066468	FGFR2	filled
ENST00000611527	ENSG00000066468	FGFR2	filled
ENST00000491111	ENSG00000066468	FGFR2	filled
ENST00000357555	ENSG00000066468	FGFR2	filled
ENST00000369061	ENSG00000066468	FGFR2	filled
ENST00000613048	ENSG00000066468	FGFR2	filled
ENST00000351936	ENSG00000066468	FGFR2	filled
ENST00000371947	ENSG00000184719	RNLS	filled
ENST00000331772	ENSG00000184719	RNLS	filled
ENST00000466945	ENSG00000184719	RNLS	filled
ENST00000481793	ENSG00000184719	RNLS	filled
ENST00000437752	ENSG00000184719	RNLS	

ENST00000471770	ENSG00000128609	NDUFA5	
ENST00000281589	ENSG00000151846	PABPC3	
ENST00000373187	ENSG00000196090	PTPRT	filled
ENST00000356100	ENSG00000196090	PTPRT	filled
ENST00000373184	ENSG00000196090	PTPRT	filled
ENST00000373190	ENSG00000196090	PTPRT	filled
ENST00000373198	ENSG00000196090	PTPRT	filled
ENST00000373201	ENSG00000196090	PTPRT	filled
ENST00000373193	ENSG00000196090	PTPRT	filled
ENST00000617474	ENSG00000196090	PTPRT	filled
ENST00000620410	ENSG00000196090	PTPRT	filled
ENST00000467125	ENSG00000047932	GOPC	filled
ENST00000256640	ENSG00000134245	WNT2B	filled
ENST00000369686	ENSG00000134245	WNT2B	
ENST00000306085	ENSG00000169871	TRIM56	
ENST00000506502	ENSG00000275740	RP11-449H3.3	filled
ENST00000598753	ENSG00000269755	CTD-3105H18.18	filled
ENST00000456542	ENSG00000079263	SP140	filled
ENST00000520635	ENSG00000104231	ZFAND1	
ENST00000520604	ENSG00000104231	ZFAND1	
ENST00000523361	ENSG00000104231	ZFAND1	
ENST00000521742	ENSG00000104231	ZFAND1	
ENST00000517353	ENSG00000104231	ZFAND1	
ENST00000521885	ENSG00000104231	ZFAND1	
ENST00000520941	ENSG00000104231	ZFAND1	
ENST00000572182	ENSG00000129214	SHBG	
ENST00000574539	ENSG00000129214	SHBG	
ENST00000576728	ENSG00000129214	SHBG	
ENST00000575314	ENSG00000129214	SHBG	
ENST00000570547	ENSG00000129214	SHBG	
ENST00000572262	ENSG00000129214	SHBG	
ENST00000576478	ENSG00000129214	SHBG	
ENST00000521740	ENSG00000240694	PNMA2	
ENST00000518212	ENSG00000240694	PNMA2	
ENST00000522764	ENSG00000240694	PNMA2	
ENST00000414997	ENSG00000239521	GATS	
ENST00000549600	ENSG00000205420	KRT6A	filled
ENST00000549754	ENSG00000205420	KRT6A	
ENST00000481842	ENSG00000135537	LACE1	
ENST00000496835	ENSG00000102309	PIN4	filled
ENST00000439980	ENSG00000102309	PIN4	filled
ENST00000423432	ENSG00000102309	PIN4	filled
ENST00000534079	ENSG00000166012	TAF1D	
ENST00000529435	ENSG00000166012	TAF1D	
ENST00000529900	ENSG00000166012	TAF1D	
ENST00000533794	ENSG00000166012	TAF1D	
ENST00000529794	ENSG00000166012	TAF1D	
ENST00000527068	ENSG00000166012	TAF1D	
ENST00000379992	ENSG00000174482	LINGO2	filled
ENST00000308675	ENSG00000174482	LINGO2	filled
ENST00000613945	ENSG00000174482	LINGO2	filled
ENST00000622810	ENSG00000131389	SLC6A6	filled
ENST00000610642	ENSG00000131389	SLC6A6	filled
ENST00000613060	ENSG00000131389	SLC6A6	filled
ENST00000618278	ENSG00000131389	SLC6A6	filled
ENST00000613930	ENSG00000131389	SLC6A6	filled
ENST00000452775	ENSG00000131389	SLC6A6	
ENST00000622186	ENSG00000131389	SLC6A6	filled
ENST00000527834	ENSG00000085788	DDHD2	
ENST00000526071	ENSG00000085788	DDHD2	filled

ENST00000529872	ENSG00000085788	DDHD2	filled
ENST00000378641	ENSG00000182621	PLCB1	filled
ENST00000338037	ENSG00000182621	PLCB1	filled
ENST00000378637	ENSG00000182621	PLCB1	filled
ENST00000404098	ENSG00000182621	PLCB1	filled
ENST00000487210	ENSG00000182621	PLCB1	filled
ENST00000510929	ENSG00000162458	FBLIM1	
ENST00000502638	ENSG00000162458	FBLIM1	
ENST00000546310	ENSG00000165462	PHOX2A	
ENST00000234091	ENSG00000115738	ID2	
ENST00000531243	ENSG00000090621	PABPC4	filled
ENST00000480820	ENSG00000174891	RSRC1	filled
ENST00000494002	ENSG00000174891	RSRC1	filled
ENST00000295930	ENSG00000174891	RSRC1	filled
ENST00000471994	ENSG00000174891	RSRC1	filled
ENST00000464171	ENSG00000174891	RSRC1	filled
ENST00000312179	ENSG00000174891	RSRC1	filled
ENST00000475278	ENSG00000174891	RSRC1	filled
ENST00000480119	ENSG00000174891	RSRC1	filled
ENST00000468344	ENSG00000174891	RSRC1	filled
ENST00000471911	ENSG00000174891	RSRC1	filled
ENST00000476899	ENSG00000174891	RSRC1	filled
ENST00000477788	ENSG00000174891	RSRC1	filled
ENST00000482822	ENSG00000174891	RSRC1	filled
ENST00000496268	ENSG00000174891	RSRC1	filled
ENST00000611884	ENSG00000174891	RSRC1	filled
ENST00000421473	ENSG00000224156	TUBB	
ENST00000413547	ENSG00000224156	TUBB	
ENST00000392910	ENSG00000159840	ZYX	
ENST00000520401	ENSG00000258864	CTC-554D6.1	
ENST00000374810	ENSG00000078804	TP53INP2	
ENST00000374809	ENSG00000078804	TP53INP2	
ENST00000532359	ENSG00000166441	RPL27A	
ENST00000319595	ENSG00000179097	HTR1F	
ENST00000444908	ENSG00000170379	FAM115C	
ENST00000518791	ENSG00000170379	FAM115C	
ENST00000357344	ENSG00000170379	FAM115C	
ENST00000441159	ENSG00000170379	FAM115C	filled
ENST00000411935	ENSG00000170379	FAM115C	
ENST00000425618	ENSG00000170379	FAM115C	
ENST00000555463	ENSG00000119640	ACYP1	
ENST00000594769	ENSG00000268083	AC104534.3	
ENST00000602021	ENSG00000268083	AC104534.3	
ENST00000439554	ENSG00000147234	FRMPD3	filled
ENST00000477796	ENSG00000147234	FRMPD3	
ENST00000276185	ENSG00000147234	FRMPD3	filled
ENST00000621525	ENSG00000258644	SYNJ2BP-COX16	filled
ENST00000617196	ENSG00000258644	SYNJ2BP-COX16	filled
ENST00000617124	ENSG00000258644	SYNJ2BP-COX16	filled
ENST00000618385	ENSG00000258644	SYNJ2BP-COX16	filled
ENST00000292524	ENSG00000160959	LRRC14	filled
ENST00000530242	ENSG00000160959	LRRC14	
ENST00000514667	ENSG00000273217	CTC-432M15.3	filled
ENST00000620913	ENSG00000017483	SLC38A5	filled
ENST00000329654	ENSG00000184588	PDE4B	filled
ENST00000341517	ENSG00000184588	PDE4B	filled
ENST00000423207	ENSG00000184588	PDE4B	filled
ENST00000531358	ENSG00000184588	PDE4B	
ENST00000532040	ENSG00000184588	PDE4B	filled
ENST00000526666	ENSG00000184588	PDE4B	filled

ENST00000412480	ENSG00000184588	PDE4B	filled
ENST00000577184	ENSG00000166342	NETO1	
ENST00000541752	ENSG00000165458	INPPL1	filled
ENST00000320683	ENSG00000165458	INPPL1	filled
ENST00000517834	ENSG00000178125	PPP1R42	filled
ENST00000437579	ENSG00000107954	NEURL1	filled
ENST00000455386	ENSG00000107954	NEURL1	filled
ENST00000324607	ENSG00000172197	MBOAT1	filled
ENST00000413292	ENSG00000068971	PPP2R5B	filled
ENST00000526559	ENSG00000068971	PPP2R5B	filled
ENST00000423981	ENSG00000178171	AMER3	
ENST00000434248	ENSG00000128000	ZNF780B	
ENST00000617676	ENSG00000128000	ZNF780B	
ENST00000465178	ENSG00000113790	EHHADH	
ENST00000534151	ENSG00000119703	ZC2HC1C	
ENST00000524913	ENSG00000119703	ZC2HC1C	
ENST00000380548	ENSG00000178031	ADAMTSL1	filled
ENST00000380559	ENSG00000178031	ADAMTSL1	filled
ENST00000489062	ENSG00000178031	ADAMTSL1	
ENST00000556674	ENSG00000119699	TGFB3	
ENST00000429978	ENSG00000121964	GTDC1	filled
ENST00000361961	ENSG00000139116	KIF21A	filled
ENST00000544797	ENSG00000139116	KIF21A	filled
ENST00000361418	ENSG00000139116	KIF21A	filled
ENST00000541463	ENSG00000139116	KIF21A	filled
ENST00000519049	ENSG00000185942	NKAIN3	filled
ENST00000523367	ENSG00000185942	NKAIN3	filled
ENST00000375370	ENSG00000198176	TFDP1	filled
ENST00000453989	ENSG00000198176	TFDP1	filled
ENST00000615042	ENSG00000139405	RITA1	
ENST00000483894	ENSG00000162613	FUBP1	
ENST00000480673	ENSG00000162613	FUBP1	
ENST00000474632	ENSG00000162613	FUBP1	
ENST00000489495	ENSG00000162613	FUBP1	
ENST00000488814	ENSG00000162613	FUBP1	
ENST00000492405	ENSG00000162613	FUBP1	
ENST00000492724	ENSG00000162613	FUBP1	filled
ENST00000548861	ENSG00000257411	RP11-603J24.9	
ENST00000570308	ENSG00000087245	MMP2	filled
ENST00000271588	ENSG00000143341	HMCN1	filled
ENST00000511901	ENSG00000164128	NPY1R	
ENST00000295092	ENSG00000162981	FAM84A	
ENST00000331243	ENSG00000162981	FAM84A	
ENST00000434031	ENSG00000174748	RPL15	
ENST00000413699	ENSG00000174748	RPL15	
ENST00000490223	ENSG00000174748	RPL15	
ENST00000435882	ENSG00000174748	RPL15	
ENST00000436146	ENSG00000174748	RPL15	
ENST00000464771	ENSG00000148773	MKI67	
ENST00000302165	ENSG00000170604	IRF2BP1	filled
ENST00000425467	ENSG00000008869	HEATR5B	
ENST00000495109	ENSG00000007341	ST7L	
ENST00000360016	ENSG00000141867	BRD4	filled
ENST00000597315	ENSG00000141867	BRD4	filled
ENST00000514985	ENSG00000250722	SEPP1	
ENST00000511224	ENSG00000250722	SEPP1	
ENST00000506577	ENSG00000250722	SEPP1	filled
ENST00000507920	ENSG00000250722	SEPP1	
ENST00000509276	ENSG00000250722	SEPP1	
ENST00000514218	ENSG00000250722	SEPP1	filled

ENST00000513303	ENSG00000250722	SEPP1	
ENST00000514403	ENSG00000250722	SEPP1	
ENST00000505309	ENSG00000250722	SEPP1	
ENST00000506078	ENSG00000250722	SEPP1	
ENST00000515626	ENSG00000250722	SEPP1	
ENST00000508937	ENSG00000250722	SEPP1	
ENST00000373826	ENSG00000188060	RAB42	
ENST00000287461	ENSG00000156853	ZNF689	
ENST00000476116	ENSG00000214827	MTCP1	
ENST00000362018	ENSG00000214827	MTCP1	
ENST00000540363	ENSG00000149380	P4HA3	
ENST00000524388	ENSG00000149380	P4HA3	filled
ENST00000320912	ENSG00000179010	MRFAP1	
ENST00000617365	ENSG00000179010	MRFAP1	
ENST00000622277	ENSG00000147164	SNX12	filled
ENST00000568749	ENSG00000180209	MYLPF	
ENST00000524150	ENSG00000112038	OPRM1	filled
ENST00000337049	ENSG00000112038	OPRM1	filled
ENST00000564663	ENSG00000260916	CCPG1	
ENST00000566794	ENSG00000140829	DHX38	
ENST00000566489	ENSG00000140829	DHX38	
ENST00000341618	ENSG00000156265	MAP3K7CL	
ENST00000399935	ENSG00000156265	MAP3K7CL	
ENST00000399934	ENSG00000156265	MAP3K7CL	
ENST00000399947	ENSG00000156265	MAP3K7CL	
ENST00000339024	ENSG00000156265	MAP3K7CL	
ENST00000372391	ENSG00000151208	DLG5	filled
ENST00000541421	ENSG00000256162	SMLR1	
ENST00000581487	ENSG00000183723	CMTM4	
ENST00000623028	ENSG00000144560	VGLL4	filled
ENST00000465821	ENSG00000167106	FAM102A	
ENST00000373084	ENSG00000167106	FAM102A	filled
ENST00000300434	ENSG00000167106	FAM102A	filled
ENST00000439581	ENSG00000068366	ACSL4	
ENST00000505075	ENSG00000068366	ACSL4	
ENST00000418265	ENSG00000261796	ISY1-RAB43	filled
ENST00000556265	ENSG00000119685	TTLL5	filled
ENST00000554132	ENSG00000119685	TTLL5	filled
ENST00000586102	ENSG00000126259	KIRREL2	
ENST00000589143	ENSG00000018869	ZNF582	
ENST00000296955	ENSG00000164465	DCBLD1	filled
ENST00000533453	ENSG00000164465	DCBLD1	filled
ENST00000435168	ENSG00000084070	SMAP2	filled
ENST00000592910	ENSG00000189114	BLOC1S3	
ENST00000272224	ENSG00000143869	GDF7	filled
ENST00000521085	ENSG00000083307	GRHL2	
ENST00000510118	ENSG00000071242	RPS6KA2	filled
ENST00000503859	ENSG00000071242	RPS6KA2	filled
ENST00000512860	ENSG00000071242	RPS6KA2	filled
ENST00000507371	ENSG00000071242	RPS6KA2	filled
ENST00000506565	ENSG00000071242	RPS6KA2	filled
ENST00000438936	ENSG00000179846	NKPD1	filled
ENST00000317951	ENSG00000179846	NKPD1	filled
ENST00000463670	ENSG00000163472	TMEM79	
ENST00000295694	ENSG00000163472	TMEM79	
ENST00000357501	ENSG00000163472	TMEM79	
ENST00000485135	ENSG00000163472	TMEM79	
ENST00000334306	ENSG00000186212	SOWAHB	
ENST00000296641	ENSG00000164220	F2RL2	
ENST00000490909	ENSG00000106333	PCOLCE	

ENST00000519093	ENSG00000147526	TACC1	filled
ENST00000587853	ENSG00000141622	RNF165	
ENST00000590330	ENSG00000141622	RNF165	
ENST00000586604	ENSG00000141622	RNF165	filled
ENST00000269439	ENSG00000141622	RNF165	filled
ENST00000543885	ENSG00000141622	RNF165	filled
ENST00000480968	ENSG00000198715	C1orf85	filled
ENST00000482579	ENSG00000198715	C1orf85	
ENST00000481050	ENSG00000198715	C1orf85	
ENST00000267984	ENSG00000140406	MESDC1	
ENST00000426362	ENSG00000140830	TXNL4B	
ENST00000569767	ENSG00000140830	TXNL4B	
ENST00000506447	ENSG00000101639	CEP192	filled
ENST00000325971	ENSG00000101639	CEP192	filled
ENST00000474753	ENSG00000152292	SH2D6	
ENST00000465936	ENSG00000165629	ATP5C1	
ENST00000509736	ENSG00000145425	RPS3A	
ENST00000512690	ENSG00000145425	RPS3A	
ENST00000515792	ENSG00000145425	RPS3A	
ENST00000590993	ENSG00000181007	ZFP82	
ENST00000310298	ENSG00000073417	PDE8A	
ENST00000557957	ENSG00000073417	PDE8A	
ENST00000339708	ENSG00000073417	PDE8A	
ENST00000478717	ENSG00000073417	PDE8A	
ENST00000559742	ENSG00000073417	PDE8A	
ENST00000394553	ENSG00000073417	PDE8A	
ENST00000394287	ENSG00000125686	MED1	filled
ENST00000300651	ENSG00000125686	MED1	filled
ENST00000577831	ENSG00000125686	MED1	filled
ENST00000581334	ENSG00000125686	MED1	
ENST00000551944	ENSG00000165805	C12orf50	filled
ENST00000357635	ENSG00000178761	FAM219B	
ENST00000566132	ENSG00000178761	FAM219B	
ENST00000563671	ENSG00000178761	FAM219B	
ENST00000563119	ENSG00000178761	FAM219B	
ENST00000569524	ENSG00000178761	FAM219B	
ENST00000566894	ENSG00000178761	FAM219B	
ENST00000399285	ENSG00000214943	GPR33	filled
ENST00000589133	ENSG00000004777	ARHGAP33	
ENST00000475115	ENSG00000169554	ZEB2	
ENST00000481822	ENSG00000160767	FAM189B	filled
ENST00000395676	ENSG00000130487	KLHDC7B	filled
ENST00000277225	ENSG00000148143	ZNF462	filled
ENST00000428390	ENSG00000214336	FOXI3	
ENST00000566946	ENSG00000168488	ATXN2L	
ENST00000562583	ENSG00000168488	ATXN2L	
ENST00000566007	ENSG00000168488	ATXN2L	
ENST00000564162	ENSG00000168488	ATXN2L	
ENST00000567024	ENSG00000168488	ATXN2L	
ENST00000400633	ENSG00000215595	C20orf202	filled
ENST00000381898	ENSG00000125818	PSMF1	
ENST00000418246	ENSG00000125818	PSMF1	
ENST00000542210	ENSG00000090975	PITPNM2	filled
ENST00000304056	ENSG00000170852	KBTBD2	filled
ENST00000485611	ENSG00000170852	KBTBD2	filled
ENST00000452926	ENSG00000170852	KBTBD2	filled
ENST00000453627	ENSG00000170852	KBTBD2	filled
ENST00000564692	ENSG00000178802	MPI	
ENST00000507438	ENSG00000113360	DROSHA	
ENST00000504592	ENSG00000153064	BANK1	filled

ENST00000565355	ENSG00000149925	ALDOA	
ENST00000342683	ENSG00000189227	C15orf61	
ENST00000425856	ENSG00000226215	APOM	
ENST00000422771	ENSG00000226215	APOM	
ENST00000464233	ENSG00000169855	ROBO1	filled
ENST00000470245	ENSG00000171169	NAIF1	
ENST00000373078	ENSG00000171169	NAIF1	
ENST00000488519	ENSG00000171169	NAIF1	filled
ENST00000466139	ENSG00000171169	NAIF1	
ENST00000582444	ENSG00000170412	GPRC5C	
ENST00000392628	ENSG00000170412	GPRC5C	
ENST00000581590	ENSG00000170412	GPRC5C	
ENST00000591817	ENSG00000161270	NPHS1	
ENST00000404039	ENSG00000130226	DPP6	filled
ENST00000406326	ENSG00000130226	DPP6	filled
ENST00000377770	ENSG00000130226	DPP6	filled
ENST00000462622	ENSG00000130226	DPP6	filled
ENST00000332007	ENSG00000130226	DPP6	filled
ENST00000496611	ENSG00000130226	DPP6	filled
ENST00000427557	ENSG00000130226	DPP6	filled
ENST00000619756	ENSG00000130226	DPP6	filled
ENST00000405995	ENSG00000130723	PRRC2B	filled
ENST00000414371	ENSG00000156500	FAM122C	
ENST00000347606	ENSG00000198185	ZNF334	filled
ENST00000457685	ENSG00000198185	ZNF334	filled
ENST00000593880	ENSG00000198185	ZNF334	filled
ENST00000596323	ENSG00000198185	ZNF334	filled
ENST00000615481	ENSG00000198185	ZNF334	
ENST00000434230	ENSG00000123562	MORF4L2	
ENST00000418819	ENSG00000123562	MORF4L2	
ENST00000309495	ENSG00000175105	ZNF654	
ENST00000398189	ENSG00000175336	APOF	
ENST00000306507	ENSG00000184492	FOXD4L1	
ENST00000473240	ENSG00000115271	GCA	
ENST00000446271	ENSG00000115271	GCA	
ENST00000563849	ENSG00000140955	ADAD2	
ENST00000369207	ENSG00000188316	ENO4	
ENST00000550514	ENSG00000196091	MYBPC1	filled
ENST00000485070	ENSG00000135248	FAM71F1	
ENST00000484425	ENSG00000135248	FAM71F1	
ENST00000493738	ENSG00000135248	FAM71F1	
ENST00000469348	ENSG00000135248	FAM71F1	
ENST00000537638	ENSG00000166986	MARS	filled
ENST00000262027	ENSG00000166986	MARS	filled
ENST00000546481	ENSG00000166986	MARS	
ENST00000545888	ENSG00000166986	MARS	filled
ENST00000547062	ENSG00000166986	MARS	
ENST00000548714	ENSG00000166986	MARS	
ENST00000551842	ENSG00000166986	MARS	
ENST00000553162	ENSG00000166986	MARS	
ENST00000547501	ENSG00000166986	MARS	
ENST00000548146	ENSG00000166986	MARS	
ENST00000553123	ENSG00000166986	MARS	
ENST00000467013	ENSG00000154928	EPHB1	
ENST00000332142	ENSG00000184611	KCNH7	filled
ENST00000328032	ENSG00000184611	KCNH7	filled
ENST00000566024	ENSG00000083799	CYLD	
ENST00000568744	ENSG00000083799	CYLD	
ENST00000382227	ENSG00000163900	TMEM41A	
ENST00000475480	ENSG00000163900	TMEM41A	

ENST00000608007	ENSG00000125462	C1orf61	
ENST00000400991	ENSG00000125462	C1orf61	
ENST00000497824	ENSG00000125462	C1orf61	
ENST00000462458	ENSG00000125462	C1orf61	
ENST00000486517	ENSG00000125462	C1orf61	
ENST00000310027	ENSG00000125462	C1orf61	
ENST00000489918	ENSG00000125462	C1orf61	
ENST00000476966	ENSG00000125462	C1orf61	
ENST00000464203	ENSG00000125462	C1orf61	
ENST00000469813	ENSG00000125462	C1orf61	filled
ENST00000495000	ENSG00000125462	C1orf61	
ENST00000367474	ENSG00000203727	SAMD5	filled
ENST00000566741	ENSG00000203727	SAMD5	filled
ENST00000468155	ENSG00000176601	MAP3K19	filled
ENST00000414343	ENSG00000176601	MAP3K19	filled
ENST00000486077	ENSG00000176601	MAP3K19	filled
ENST00000401867	ENSG00000072682	P4HA2	
ENST00000379086	ENSG00000072682	P4HA2	
ENST00000166534	ENSG00000072682	P4HA2	
ENST00000360568	ENSG00000072682	P4HA2	
ENST00000506807	ENSG00000072682	P4HA2	
ENST00000417528	ENSG00000072682	P4HA2	
ENST00000431054	ENSG00000072682	P4HA2	filled
ENST00000439698	ENSG00000072682	P4HA2	
ENST00000395164	ENSG00000072682	P4HA2	
ENST00000453286	ENSG00000072682	P4HA2	
ENST00000428369	ENSG00000072682	P4HA2	
ENST00000418055	ENSG00000072682	P4HA2	
ENST00000478055	ENSG00000072682	P4HA2	
ENST00000416053	ENSG00000072682	P4HA2	
ENST00000428841	ENSG00000072682	P4HA2	
ENST00000379100	ENSG00000072682	P4HA2	
ENST00000379104	ENSG00000072682	P4HA2	
ENST00000392039	ENSG00000135898	GPR55	filled
ENST00000438398	ENSG00000135898	GPR55	
ENST00000504572	ENSG00000113580	NR3C1	filled
ENST00000505058	ENSG00000113580	NR3C1	
ENST00000503701	ENSG00000113580	NR3C1	
ENST00000343796	ENSG00000113580	NR3C1	filled
ENST00000572329	ENSG00000175866	BAIAP2	filled
ENST00000571530	ENSG00000175866	BAIAP2	filled
ENST00000559067	ENSG00000140538	NTRK3	filled
ENST00000265260	ENSG00000081154	PCNP	
ENST00000460231	ENSG00000081154	PCNP	
ENST00000469941	ENSG00000081154	PCNP	
ENST00000486406	ENSG00000081154	PCNP	
ENST00000580716	ENSG00000169727	GPS1	
ENST00000578168	ENSG00000169727	GPS1	
ENST00000583486	ENSG00000169727	GPS1	
ENST00000547666	ENSG00000120868	APAF1	
ENST00000616478	ENSG00000153721	CNKSR3	filled
ENST00000433368	ENSG00000096006	CRISP3	filled
ENST00000448480	ENSG00000003400	CASP10	filled
ENST00000484926	ENSG00000003400	CASP10	
ENST00000494598	ENSG00000114805	PLCH1	filled
ENST00000424440	ENSG00000173699	SPATA3	
ENST00000433428	ENSG00000173699	SPATA3	
ENST00000455816	ENSG00000173699	SPATA3	
ENST00000440792	ENSG00000173699	SPATA3	
ENST00000423134	ENSG00000173699	SPATA3	

ENST00000612823	ENSG00000173699	SPATA3	
ENST00000565560	ENSG00000140939	NOL3	
ENST00000564992	ENSG00000140939	NOL3	
ENST00000268605	ENSG00000140939	NOL3	
ENST00000373640	ENSG00000204128	C2orf72	
ENST00000463834	ENSG00000204128	C2orf72	
ENST00000394236	ENSG00000184500	PROS1	filled
ENST00000348159	ENSG00000116604	MEF2D	filled
ENST00000489057	ENSG00000116604	MEF2D	filled
ENST00000561067	ENSG00000092295	TGM1	
ENST00000514747	ENSG00000146067	FAM193B	filled
ENST00000506955	ENSG00000146067	FAM193B	filled
ENST00000508298	ENSG00000146067	FAM193B	filled
ENST00000510429	ENSG00000146067	FAM193B	
ENST00000506432	ENSG00000146067	FAM193B	
ENST00000507587	ENSG00000146067	FAM193B	filled
ENST00000600940	ENSG00000131864	USP29	
ENST00000377813	ENSG00000125844	RRBP1	filled
ENST00000360807	ENSG00000125844	RRBP1	filled
ENST00000455029	ENSG00000125844	RRBP1	filled
ENST00000398782	ENSG00000125844	RRBP1	
ENST00000377807	ENSG00000125844	RRBP1	filled
ENST00000541970	ENSG00000071994	PDCD2	
ENST00000167218	ENSG00000071994	PDCD2	
ENST00000392090	ENSG00000071994	PDCD2	
ENST00000545869	ENSG00000071994	PDCD2	
ENST00000542896	ENSG00000071994	PDCD2	
ENST00000453163	ENSG00000071994	PDCD2	
ENST00000537445	ENSG00000071994	PDCD2	
ENST00000614056	ENSG00000071994	PDCD2	
ENST00000486712	ENSG00000114738	MAPKAPK3	filled
ENST00000470153	ENSG00000166997	CNPY4	filled
ENST00000561621	ENSG00000196123	KIAA0895L	
ENST00000290881	ENSG00000196123	KIAA0895L	
ENST00000563902	ENSG00000196123	KIAA0895L	
ENST00000563831	ENSG00000196123	KIAA0895L	
ENST00000564423	ENSG00000196123	KIAA0895L	
ENST00000560009	ENSG00000137841	PLCB2	filled
ENST00000559671	ENSG00000137841	PLCB2	
ENST00000559618	ENSG00000137841	PLCB2	
ENST00000512055	ENSG00000196353	CPNE4	filled
ENST00000512332	ENSG00000196353	CPNE4	filled
ENST00000511604	ENSG00000196353	CPNE4	filled
ENST00000502818	ENSG00000196353	CPNE4	filled
ENST00000505881	ENSG00000196353	CPNE4	filled
ENST00000514999	ENSG00000196353	CPNE4	filled
ENST00000505957	ENSG00000196353	CPNE4	filled
ENST00000429747	ENSG00000196353	CPNE4	filled
ENST00000617767	ENSG00000196353	CPNE4	filled
ENST00000470622	ENSG00000074706	IPCEF1	
ENST00000044462	ENSG00000041357	PSMA4	
ENST00000599996	ENSG00000269547	CTC-360G5.8	filled
ENST00000367754	ENSG00000117507	FMO6P	filled
ENST00000236166	ENSG00000117507	FMO6P	filled
ENST00000286298	ENSG00000155850	SLC26A2	
ENST00000503336	ENSG00000155850	SLC26A2	
ENST00000580749	ENSG00000182938	OTOP3	
ENST00000328801	ENSG00000182938	OTOP3	
ENST00000357731	ENSG00000172260	NEGR1	filled
ENST00000306821	ENSG00000172260	NEGR1	filled

ENST00000467479	ENSG00000172260	NEGR1	filled
ENST00000434200	ENSG00000172260	NEGR1	filled
ENST00000490820	ENSG00000137504	CREBZF	
ENST00000528561	ENSG00000137504	CREBZF	
ENST00000260058	ENSG00000137504	CREBZF	
ENST00000531515	ENSG00000137504	CREBZF	
ENST00000534224	ENSG00000137504	CREBZF	
ENST00000527529	ENSG00000137504	CREBZF	
ENST00000525639	ENSG00000137504	CREBZF	
ENST00000527447	ENSG00000137504	CREBZF	
ENST00000528889	ENSG00000137504	CREBZF	
ENST00000568844	ENSG00000186187	ZNRF1	
ENST00000505790	ENSG00000113430	IRX4	
ENST00000613726	ENSG00000113430	IRX4	
ENST00000506111	ENSG00000174123	TLR10	filled
ENST00000598691	ENSG00000130529	TRPM4	
ENST00000594568	ENSG00000130529	TRPM4	filled
ENST00000599459	ENSG00000130529	TRPM4	
ENST00000597316	ENSG00000130529	TRPM4	
ENST00000465920	ENSG00000083444	PLOD1	filled
ENST00000486206	ENSG00000152430	BOLL	
ENST00000544582	ENSG00000076053	RBM7	
ENST00000296736	ENSG00000164296	TIGD6	
ENST00000515406	ENSG00000164296	TIGD6	
ENST00000532987	ENSG00000164296	TIGD6	
ENST00000568323	ENSG00000103091	WDR59	filled
ENST00000562331	ENSG00000103091	WDR59	filled
ENST00000611716	ENSG00000140479	PCSK6	filled
ENST00000541657	ENSG00000139725	RHOF	
ENST00000545544	ENSG00000139725	RHOF	
ENST00000493340	ENSG00000059377	TBXAS1	filled
ENST00000493858	ENSG00000127922	SHFM1	
ENST00000619259	ENSG00000127922	SHFM1	filled
ENST00000617133	ENSG00000127922	SHFM1	filled
ENST00000417009	ENSG00000127922	SHFM1	filled
ENST00000444799	ENSG00000127922	SHFM1	filled
ENST00000449279	ENSG00000127922	SHFM1	filled
ENST00000413065	ENSG00000127922	SHFM1	filled
ENST00000615352	ENSG00000127922	SHFM1	
ENST00000356686	ENSG00000127922	SHFM1	
ENST00000471602	ENSG00000196510	ANAPC7	filled
ENST00000486773	ENSG00000169291	SHE	
ENST00000555188	ENSG00000169291	SHE	
ENST00000367469	ENSG00000111961	SASH1	filled
ENST00000282276	ENSG00000247626	MARS2	
ENST00000254101	ENSG00000131791	PRKAB2	
ENST00000287474	ENSG00000156869	FRRS1	filled
ENST00000330493	ENSG00000184774	MGAT4EP	
ENST00000623000	ENSG00000279049	AL353898.1	
ENST00000624707	ENSG00000279272	AC109925.1	filled
ENST00000616190	ENSG00000276217	AC006518.1	
ENST00000624398	ENSG00000280249	AL691477.1	
ENST00000624984	ENSG00000280004	AC005086.2	
ENST00000625173	ENSG00000278947	AC011385.1	
ENST00000625131	ENSG00000279336	AL353662.2	
ENST00000624425	ENSG00000279966	AC008626.1	filled
ENST00000623044	ENSG00000280454	AC007347.1	
ENST00000624227	ENSG00000279975	AL353662.3	
ENST00000560651	ENSG00000218052	ADAMTS7P4	
ENST00000493696	ENSG00000239435	KCNMB3P1	

ENST00000380331	ENSG00000186312	CA5BP1	filled
ENST00000447500	ENSG00000230092	RP11-206L10.8	filled
ENST00000484361	ENSG00000237621	OR9A1P	
ENST00000497195	ENSG00000227835	CARM1P1	
ENST00000611153	ENSG00000275002	RP11-187E13.3	
ENST00000613699	ENSG00000275002	RP11-187E13.3	
ENST00000437462	ENSG00000224479	AC136289.1	
ENST00000436687	ENSG00000224479	AC136289.1	
ENST00000435541	ENSG00000161912	ADCY10P1	
ENST00000485041	ENSG00000243910	TUBA4B	filled
ENST00000473885	ENSG00000243910	TUBA4B	filled
ENST00000490341	ENSG00000243910	TUBA4B	filled
ENST00000533341	ENSG00000254667	AP000783.1	filled
ENST00000623141	ENSG00000254667	AP000783.1	
ENST00000587192	ENSG00000237732	AC010980.2	
ENST00000555502	ENSG00000237732	AC010980.2	
ENST00000439237	ENSG00000237732	AC010980.2	
ENST00000606619	ENSG00000243406	MRPS31P5	
ENST00000447201	ENSG00000236155	RP11-231P20.2	
ENST00000561384	ENSG00000259479	SORD2P	filled
ENST00000514503	ENSG00000250654	RP11-834C11.7	
ENST00000607809	ENSG00000250654	RP11-834C11.7	
ENST00000505259	ENSG00000250654	RP11-834C11.7	
ENST00000504756	ENSG00000170089	RP11-423H2.1	
ENST00000578564	ENSG00000266714	MYO15B	
ENST00000582561	ENSG00000266714	MYO15B	
ENST00000581612	ENSG00000266714	MYO15B	
ENST00000581866	ENSG00000266714	MYO15B	
ENST00000583140	ENSG00000266714	MYO15B	
ENST00000577296	ENSG00000266714	MYO15B	
ENST00000605100	ENSG00000250878	METTL21EP	
ENST00000508429	ENSG00000230578	RP13-228J13.5	
ENST00000453508	ENSG00000230578	RP13-228J13.5	
ENST00000382965	ENSG00000206159	GYG2P1	
ENST00000438139	ENSG00000234761	RP11-38O14.5	
ENST00000417729	ENSG00000234761	RP11-38O14.5	
ENST00000483225	ENSG00000187893	CXXC1P1	
ENST00000589302	ENSG00000187893	CXXC1P1	filled
ENST00000329395	ENSG00000179766	ATP8B5P	filled
ENST00000423138	ENSG00000179766	ATP8B5P	filled
ENST00000422009	ENSG00000242507	RP3-477O4.5	
ENST00000450773	ENSG00000242507	RP3-477O4.5	
ENST00000432650	ENSG00000242507	RP3-477O4.5	
ENST00000446499	ENSG00000232022	FAAHP1	
ENST00000606395	ENSG00000232022	FAAHP1	
ENST00000607656	ENSG00000232022	FAAHP1	
ENST00000429784	ENSG00000232022	FAAHP1	
ENST00000412192	ENSG00000229150	CRYGEP	
ENST00000440809	ENSG00000229150	CRYGEP	
ENST00000515154	ENSG00000226074	PRSS44	
ENST00000413293	ENSG00000272779	LL22NC03-80A10.(	filled
ENST00000514295	ENSG00000250026	TMPRSS11BNL	
ENST00000513714	ENSG00000250026	TMPRSS11BNL	
ENST00000514420	ENSG00000250026	TMPRSS11BNL	
ENST00000504453	ENSG00000250026	TMPRSS11BNL	filled
ENST00000568403	ENSG00000261741	CTD-2014E2.5	
ENST00000565692	ENSG00000261741	CTD-2014E2.5	filled
ENST00000451043	ENSG00000182048	TRPC2	filled
ENST00000575421	ENSG00000262583	RP11-77K12.5	
ENST00000423435	ENSG00000224353	ACE3P	

ENST00000340135	ENSG00000189332	RP11-113D6.10	
ENST00000534640	ENSG00000189332	RP11-113D6.10	
ENST00000527059	ENSG00000189332	RP11-113D6.10	filled
ENST00000463099	ENSG00000243064	ABCC13	filled
ENST00000442370	ENSG00000272135	RP11-107C16.2	
ENST00000606028	ENSG00000272135	RP11-107C16.2	
ENST00000435645	ENSG00000234814	SVILP1	
ENST00000450249	ENSG00000204787	REG1P	
ENST00000529026	ENSG00000255085	AF186192.5	
ENST00000480264	ENSG00000253882	RP11-61L23.2	
ENST00000525030	ENSG00000176654	NANOGP1	
ENST00000507787	ENSG00000206149	HERC2P9	
ENST00000458319	ENSG00000233167	EEF1A1P26	
ENST00000557651	ENSG00000258786	RP11-928F19.3	
ENST00000433573	ENSG00000233026	MTCO1P5	
ENST00000445763	ENSG00000225384	RP13-643D4.1	
ENST00000604305	ENSG00000270739	RP11-608O8.2	
ENST00000486188	ENSG00000241950	RPL29P23	
ENST00000432491	ENSG00000233309	RPS6P12	
ENST00000439300	ENSG00000237833	RP11-432N13.2	
ENST00000573021	ENSG00000262090	RP11-23E10.5	
ENST00000604770	ENSG00000271195	RP11-162I7.2	
ENST00000556746	ENSG00000259086	RP11-134E15.2	
ENST00000591298	ENSG00000267178	PHF5CP	
ENST00000605098	ENSG00000271177	RP11-575G13.3	
ENST00000527643	ENSG00000255444	CYCSP27	
ENST00000487566	ENSG00000244451	RPL34P21	
ENST00000515049	ENSG00000249176	RP11-304F15.5	
ENST00000495613	ENSG00000232150	ST13P4	
ENST00000524889	ENSG00000254974	RP11-702H23.2	
ENST00000490840	ENSG00000240156	COX6CP6	
ENST00000438468	ENSG00000225443	AC004938.5	
ENST00000547227	ENSG00000258251	YPEL5P3	
ENST00000578535	ENSG00000266520	RAC1P1	
ENST00000605515	ENSG00000270973	SMARCE1P7	
ENST00000514691	ENSG00000251195	RP11-378N18.1	
ENST00000450258	ENSG00000227992	AC108463.2	
ENST00000484185	ENSG00000226729	RPL35AP30	
ENST00000605507	ENSG00000270766	RP11-627K11.5	
ENST00000569247	ENSG00000260184	RP11-42I10.3	
ENST00000604143	ENSG00000271025	RP11-708L7.9	
ENST00000400856	ENSG00000215760	TAF9BP2	
ENST00000579571	ENSG00000266809	RP11-449D8.3	
ENST00000586289	ENSG00000267664	RPL17P45	
ENST00000439324	ENSG00000227875	RP11-426M1.2	
ENST00000455437	ENSG00000214651	RP11-477J21.2	
ENST00000553815	ENSG00000258493	RP11-73E17.3	
ENST00000585581	ENSG00000267471	RP11-1058G23.1	
ENST00000403053	ENSG00000216360	RP1-182O16.2	
ENST00000527756	ENSG00000255445	RP11-573M3.3	
ENST00000584490	ENSG00000265453	RP11-173M1.3	
ENST00000555184	ENSG00000258996	CTD-2315A10.1	
ENST00000447155	ENSG00000229384	HMGB1P16	
ENST00000451754	ENSG00000223724	RAD17P2	filled
ENST00000432315	ENSG00000226765	RP11-694D5.1	
ENST00000559372	ENSG00000259228	HNRNPA1P62	
ENST00000423238	ENSG00000224401	RPL7P57	
ENST00000605318	ENSG00000271563	HIRAP1	
ENST00000438196	ENSG00000230451	RPS29P6	
ENST00000472109	ENSG00000240305	RP11-1072C15.1	

ENST00000425667	ENSG00000224431	AC063976.7	
ENST00000421987	ENSG00000205716	FAM183DP	
ENST00000487828	ENSG00000242140	RP11-262M14.2	
ENST00000604064	ENSG00000271150	CTD-2571L23.9	
ENST00000457883	ENSG00000234765	ELL2P3	
ENST00000573303	ENSG00000262048	RP1-232L24.4	
ENST00000524791	ENSG00000255296	RP11-757C15.4	
ENST00000407020	ENSG00000219361	RP11-250B2.2	
ENST00000420841	ENSG00000233161	DHFRP2	
ENST00000326971	ENSG00000181705	RP11-11L12.2	
ENST00000435950	ENSG00000226468	AC018641.7	
ENST00000620521	ENSG00000274727	RP11-476I15.6	filled
ENST00000434717	ENSG00000226255	UBE2V2P1	
ENST00000426582	ENSG00000118903	BTF3P11	
ENST00000467595	ENSG00000242752	NMTRQ-TTG12-1	
ENST00000544832	ENSG00000256723	RP11-167N4.4	
ENST00000432247	ENSG00000235182	RP11-348H3.4	
ENST00000430506	ENSG00000237554	RPL7AP7	
ENST00000550979	ENSG00000258016	HIGD1AP1	
ENST00000406493	ENSG00000218793	RP3-382I10.3	
ENST00000448700	ENSG00000233557	NFU1P2	
ENST00000582031	ENSG00000265883	RP11-169I9.2	
ENST00000427294	ENSG00000227883	ATP6V0E1P4	
ENST00000433415	ENSG00000231120	BTF3P10	
ENST00000458090	ENSG00000235325	AC009963.6	
ENST00000417217	ENSG00000229616	RP11-369J21.11	
ENST00000469035	ENSG00000241307	RP11-674E16.1	
ENST00000604895	ENSG00000270268	RP1-164L12.1	
ENST00000458495	ENSG00000234107	TPT1P1	
ENST00000566966	ENSG00000261232	RP11-331H13.1	
ENST00000495857	ENSG00000239317	RP11-449H3.2	
ENST00000534985	ENSG00000256399	RP11-274J7.3	
ENST00000444513	ENSG00000256211	RP11-1018J11.1	
ENST00000426992	ENSG00000224690	UBE2D3P3	
ENST00000415778	ENSG00000213187	COPS5P1	
ENST00000452014	ENSG00000224686	TCEB1P23	
ENST00000441148	ENSG00000225251	RPL5P25	
ENST00000429986	ENSG00000226902	CHCHD2P1	
ENST00000511296	ENSG00000248867	CTD-2128F4.1	
ENST00000489849	ENSG00000242729	RP11-124G5.1	
ENST00000406185	ENSG00000219222	RP11-480N24.3	
ENST00000497325	ENSG00000213862	CTD-2270N23.1	
ENST00000439003	ENSG00000230012	HTATSF1P	
ENST00000446931	ENSG00000227211	RP5-1100E15.4	
ENST00000416198	ENSG00000237005	HIGD1AP15	
ENST00000400214	ENSG00000215399	HMGB3P7	
ENST00000515565	ENSG00000248854	HNRNPH1P3	
ENST00000590816	ENSG00000267634	RPL7L1P5	
ENST00000489727	ENSG00000240518	RP11-515C16.1	
ENST00000508153	ENSG00000251162	RP11-75A5.1	
ENST00000604334	ENSG00000271629	RP11-79J24.1	
ENST00000426371	ENSG00000212961	HNRNPA1P40	filled
ENST00000510182	ENSG00000250307	TUBB8P3	
ENST00000448026	ENSG00000264497	RP11-640N20.5	
ENST00000472460	ENSG00000240174	RP11-851M3.1	
ENST00000525475	ENSG00000254678	RP11-109L13.5	
ENST00000511924	ENSG00000227814	MRPS17P9	
ENST00000452483	ENSG00000224551	HMGB3P21	
ENST00000614164	ENSG00000278824	RP11-1315F8.2	
ENST00000564298	ENSG00000260689	HNRNPA3P11	

ENST00000491913	ENSG00000242135	RPL17P2
ENST00000565911	ENSG00000261356	RP11-46D6.5
ENST00000604049	ENSG00000271428	RP1-224A6.8
ENST00000474156	ENSG00000239683	RP11-322E11.1
ENST00000447672	ENSG00000229753	RP11-28P17.3
ENST00000446380	ENSG00000230016	DHFRP2
ENST00000398929	ENSG00000278476	AC009414.1
ENST00000555623	ENSG00000258439	RP11-173A8.2
ENST00000613427	ENSG00000277979	RP11-157L3.6
ENST00000429873	ENSG00000235115	CHCHD2P8
ENST00000405355	ENSG00000219559	RP11-346C16.4
ENST00000441858	ENSG00000226515	AC004386.3
ENST00000502376	ENSG00000250562	RPL38P4
ENST00000545001	ENSG00000256293	RP11-114F3.2
ENST00000536546	ENSG00000256192	RP11-290I21.2
ENST00000489757	ENSG00000243687	RP11-432B6.1
ENST00000497800	ENSG00000214903	RPS15AP3
ENST00000552666	ENSG00000257957	QRSL1P3
ENST00000520250	ENSG00000253654	CTD-2210A23.1
ENST00000405924	ENSG00000218893	SUMO2P12
ENST00000605266	ENSG00000270627	RP11-216F5.1
ENST00000622677	ENSG00000277646	RP11-275A14.1
ENST00000404707	ENSG00000217612	RP1-28C20.1
ENST00000446654	ENSG00000236776	RPL21P23
ENST00000395943	ENSG00000230256	FGFR1OP2P1
ENST00000442812	ENSG00000225475	RP11-377K22.2
ENST00000508858	ENSG00000250194	RP11-257I8.1
ENST00000522682	ENSG00000253655	IGJP1
ENST00000422816	ENSG00000229944	EIF4EP2
ENST00000603797	ENSG00000271257	CTD-2117L12.3
ENST00000464109	ENSG00000244134	RPS12P20
ENST00000465478	ENSG00000233778	RP11-777J24.1
ENST00000569492	ENSG00000261620	HMGN2P41
ENST00000572768	ENSG00000262892	RP11-429H5.1
ENST00000603022	ENSG00000270791	DPRXP6
ENST00000399411	ENSG00000214998	CCNB2P1
ENST00000604477	ENSG00000271496	SNRPGP20
ENST00000447108	ENSG00000223746	TCEB1P30
ENST00000303758	ENSG00000172186	HMGN1P35
ENST00000449240	ENSG00000227470	AC073415.2
ENST00000562529	ENSG00000261725	RP11-480G7.3
ENST00000563247	ENSG00000260472	CTD-2358C21.2
ENST00000534163	ENSG00000255222	SETP17
ENST00000444159	ENSG00000215236	RPL7P33
ENST00000532834	ENSG00000255035	SDHCP4
ENST00000540463	ENSG00000256021	TCEB1P31
ENST00000429407	ENSG00000225893	RP11-6J24.3
ENST00000444468	ENSG00000226339	RPS26P56
ENST00000413955	ENSG00000235425	RPS7P13
ENST00000452590	ENSG00000236863	RPL23AP23
ENST00000423841	ENSG00000231684	EIF1P3
ENST00000605789	ENSG00000271088	RP11-497J7.4
ENST00000425552	ENSG00000234075	RPL35AP
ENST00000504217	ENSG00000250293	CRYZP2
ENST00000451924	ENSG00000233565	RP11-42D20.1
ENST00000620067	ENSG00000275598	CMB9-14B22.1
ENST00000435137	ENSG00000234982	RP11-280O24.3
ENST00000559765	ENSG00000259304	CTD-2014N11.3
ENST00000404812	ENSG00000228292	RP11-220I1.4
ENST00000443543	ENSG00000233648	AC010095.6

ENST00000431406	ENSG00000235289	BRD7P6
ENST00000412883	ENSG00000231144	EEF1A1P40
ENST00000406786	ENSG00000217041	RP11-346C16.1
ENST00000417134	ENSG00000213149	CNN2P9
ENST00000480448	ENSG00000242573	ACTR3P3
ENST00000477071	ENSG00000242697	RPL5P12
ENST00000407268	ENSG00000223967	RP11-505P4.5
ENST00000441206	ENSG00000234743	EIF5AP4
ENST00000603444	ENSG00000270995	RP11-29B11.5
ENST00000607399	ENSG00000271846	CTD-3113P16.9
ENST00000513977	ENSG00000249019	RP11-539G18.1
ENST00000415651	ENSG00000237629	UQCRHP2
ENST00000435997	ENSG00000232678	SPTLC1P4
ENST00000411559	ENSG00000231124	UBE2V1P11
ENST00000417306	ENSG00000237901	AC009161.1
ENST00000412164	ENSG00000232875	HMGN2P35
ENST00000463110	ENSG00000240231	RPS27P29
ENST00000458289	ENSG00000236530	AC097711.1
ENST00000552027	ENSG00000258133	RP11-340M11.1
ENST00000542687	ENSG00000256037	MRPL40P1
ENST00000535885	ENSG00000256355	NTAN1P3
ENST00000548858	ENSG00000258271	RP11-356O22.4
ENST00000438279	ENSG00000226066	AC009541.1
ENST00000414021	ENSG00000234272	RPL30P2
ENST00000407436	ENSG00000217181	RP11-360O19.5
ENST00000598428	ENSG00000268080	RP11-388K12.3
ENST00000440352	ENSG00000227638	HNRNPA1P14
ENST00000482895	ENSG00000242970	AC068522.4
ENST00000551910	ENSG00000257662	EIF4A1P12
ENST00000409926	ENSG00000224334	AP000357.4
ENST00000579720	ENSG00000265545	RP11-183C12.1
ENST00000412199	ENSG00000236722	RP11-359I18.1
ENST00000604554	ENSG00000271464	DPRXP7
ENST00000467028	ENSG00000243516	RP11-19F9.1
ENST00000514359	ENSG00000248851	AC006427.2
ENST00000407538	ENSG00000216548	RP11-471B18.1
ENST00000504623	ENSG00000249795	RP11-18O11.2
ENST00000438988	ENSG00000231725	VN1R110P
ENST00000615621	ENSG00000274823	RP11-348M3.6
ENST00000586584	ENSG00000267433	CTD-2057D4.2
ENST00000498782	ENSG00000244199	RP11-139K4.4
ENST00000482859	ENSG00000241741	RPL7AP30
ENST00000404330	ENSG00000219547	RPL17P25
ENST00000443510	ENSG00000234901	MTND6P13
ENST00000469911	ENSG00000228766	RPL7L1P8
ENST00000447970	ENSG00000226499	RP5-882O7.1
ENST00000434731	ENSG00000233880	RP11-497J7.2
ENST00000455287	ENSG00000228364	SAR1P1
ENST00000542626	ENSG00000256917	RP11-81H14.4
ENST00000317902	ENSG00000232370	AF241726.2
ENST00000428051	ENSG00000224339	AL121578.5
ENST00000419827	ENSG00000229091	HSPA8P8
ENST00000605814	ENSG00000271237	RP11-140C5.3
ENST00000443781	ENSG00000228366	RP11-18B3.3
ENST00000489253	ENSG00000228938	SNRPD2P1
ENST00000529375	ENSG00000255376	RP11-360K13.2
ENST00000603614	ENSG00000271277	RP11-82O2.2
ENST00000412619	ENSG00000230283	RPS12P24
ENST00000425207	ENSG00000237729	AC002075.4
ENST00000402768	ENSG00000219806	ATP5F1P6

ENST00000381279	ENSG00000205695	RP11-15H7.2	
ENST00000604867	ENSG00000270549	RP11-293K19.1	
ENST00000432461	ENSG00000231020	RP11-357J22.1	
ENST00000508230	ENSG00000250214	RP13-488H8.1	
ENST00000473326	ENSG00000235459	RPS26P31	
ENST00000498103	ENSG00000212664	RP11-592N21.1	
ENST00000573044	ENSG00000263029	RP11-517A5.6	
ENST00000603090	ENSG00000270822	RP11-730B22.1	
ENST00000473054	ENSG00000240210	RP11-204K16.1	
ENST00000493832	ENSG00000242941	CTD-2325P2.2	
ENST00000478775	ENSG00000240074	RPL9P30	
ENST00000502445	ENSG00000250540	RP11-58H15.3	
ENST00000510511	ENSG00000249976	RP11-580P21.1	
ENST00000559695	ENSG00000259192	AC109631.1	
ENST00000426038	ENSG00000225904	MORF4L1P7	
ENST00000403118	ENSG00000219039	AC005102.1	filled
ENST00000619984	ENSG00000275423	RP11-429P7.1	
ENST00000406305	ENSG00000218173	RP11-427E4.1	
ENST00000479840	ENSG00000243423	RP5-837J1.1	
ENST00000416693	ENSG00000227845	RP11-184B22.2	
ENST00000489560	ENSG00000241354	RP11-1042B17.2	
ENST00000470927	ENSG00000214062	RPL7P17	
ENST00000445734	ENSG00000227988	TDGF1P1	
ENST00000420997	ENSG00000234349	GLUD1P9	
ENST00000405812	ENSG00000216811	RP1-69B13.2	
ENST00000494006	ENSG00000242198	CTD-2235C13.1	
ENST00000436391	ENSG00000232149	FERP1	
ENST00000495884	ENSG00000240870	RPL19P14	
ENST00000419975	ENSG00000213260	YWHAZP5	
ENST00000546846	ENSG00000257905	RP3-432I18.1	
ENST00000556889	ENSG00000258494	OR11J5P	
ENST00000431298	ENSG00000231035	RPL7L1P9	
ENST00000426556	ENSG00000223498	RP11-716A19.3	
ENST00000458411	ENSG00000238259	AC067940.1	
ENST00000494861	ENSG00000242169	RP11-219G10.1	
ENST00000402778	ENSG00000220446	RP3-520B18.1	
ENST00000603750	ENSG00000271166	RP11-409O11.3	
ENST00000449897	ENSG00000227309	AC140076.1	
ENST00000421072	ENSG00000231780	AC090960.1	
ENST00000422311	ENSG00000233578	RP5-1022P6.3	
ENST00000611595	ENSG00000273536	RP11-74M13.6	filled
ENST00000366255	ENSG00000203437	RP11-820K3.3	
ENST00000442505	ENSG00000228110	ST13P19	
ENST00000402231	ENSG00000220132	AF238380.8	
ENST00000431429	ENSG00000230611	HMGB1P27	
ENST00000603598	ENSG00000271027	RP11-463M14.1	
ENST00000449348	ENSG00000234853	NDUFA5P3	
ENST00000405921	ENSG00000216781	RP1-290I10.2	
ENST00000478982	ENSG00000239288	RP11-59E19.3	
ENST00000399435	ENSG00000215016	RPL24P7	
ENST00000428741	ENSG00000243659	RP11-4K3_A.3	
ENST00000563903	ENSG00000261282	SOD1P2	
ENST00000560431	ENSG00000259173	RP11-30K9.7	
ENST00000463059	ENSG00000236111	RP11-630I5.1	
ENST00000401900	ENSG00000216359	RP11-360O19.1	
ENST00000577737	ENSG00000266771	RP11-19P22.10	
ENST00000543361	ENSG00000256221	AE000661.36	
ENST00000447766	ENSG00000228751	AC004022.8	
ENST00000441137	ENSG00000234732	RPEP5	
ENST00000587055	ENSG00000267591	RP11-689E3.2	

ENST00000418453	ENSG00000237109	RPL21P111
ENST00000509113	ENSG00000250703	CTD-2533K21.1
ENST00000447049	ENSG00000228341	CTB-20D2.1
ENST00000415008	ENSG00000224007	AC019070.1
ENST00000434028	ENSG00000223568	RPL3P11
ENST00000475062	ENSG00000240163	RP11-745A24.1
ENST00000421173	ENSG00000234748	RP11-183K14.1
ENST00000520383	ENSG00000253697	RP11-1081K18.1
ENST00000446870	ENSG00000226388	ELL2P4
ENST00000404109	ENSG00000217030	RP11-428J1.2
ENST00000603098	ENSG00000271558	RP11-445P19.2
ENST00000475330	ENSG00000231516	CBX1P5
ENST00000412705	ENSG00000228289	RP4-640E24.1
ENST00000477483	ENSG00000239320	RPS29P26
ENST00000511084	ENSG00000213488	CTB-85P21.1
ENST00000469402	ENSG00000240436	RP11-95I19.1
ENST00000605154	ENSG00000270990	RP11-632K21.6
ENST00000616637	ENSG00000278215	RP11-712L6.8
ENST00000579952	ENSG00000264054	RPL31P9
ENST00000420390	ENSG00000230119	AC123900.2
ENST00000457739	ENSG00000231913	RARRES2P3
ENST00000399029	ENSG00000214812	RP5-1053E7.3
ENST00000605824	ENSG00000271332	RP11-797J4.1
ENST00000555980	ENSG00000258587	RP11-316E14.2
ENST00000496412	ENSG00000241739	CTD-2290C23.2
ENST00000526403	ENSG00000254944	ATP5F1P5
ENST00000520175	ENSG00000254112	KB-1205A7.1
ENST00000517879	ENSG00000253299	RP11-316J7.4
ENST00000413929	ENSG00000224692	LL0XNC01-177E8.2
ENST00000462872	ENSG00000244249	RP11-441M10.1
ENST00000485389	ENSG00000243116	RP11-564C24.1
ENST00000459863	ENSG00000214560	RPL21P41
ENST00000479876	ENSG00000240047	RPS3AP32
ENST00000567221	ENSG00000231169	EEF1B2P1
ENST00000505332	ENSG00000248106	AC005609.2
ENST00000408034	ENSG00000217585	RP1-13D10.5
ENST00000517893	ENSG00000253907	RP11-380I10.3
ENST00000468519	ENSG00000232830	RP11-144C15.1
ENST00000434498	ENSG00000214429	CYCSP6
ENST00000421119	ENSG00000237137	RP11-408A13.2
ENST00000560646	ENSG00000259433	CTD-2651B20.4
ENST00000604342	ENSG00000271597	RP11-85D18.1
ENST00000433468	ENSG00000228781	RP11-336N8.3
ENST00000513715	ENSG00000248188	RP11-442P12.2
ENST00000618593	ENSG00000274844	RP11-325M4.2
ENST00000518744	ENSG00000253346	CTD-3107M8.1
ENST00000502591	ENSG00000248349	RP11-79C6.1
ENST00000507850	ENSG00000249649	MRPS33P2
ENST00000584401	ENSG00000266048	CTD-2206N4.1
ENST00000476891	ENSG00000239263	RBM43P1
ENST00000463445	ENSG00000196933	RPS26P11
ENST00000549779	ENSG00000257503	CYB5AP5
ENST00000556323	ENSG00000259020	RP11-529H20.3
ENST00000479856	ENSG00000244604	RP11-713H12.1
ENST00000265259	ENSG00000213561	RP11-386I14.2
ENST00000498068	ENSG00000242882	RPL5P11
ENST00000604027	ENSG00000270665	HNRNPA1P67
ENST00000605229	ENSG00000270606	RP11-713H12.2
ENST00000457712	ENSG00000230407	AC018717.2
ENST00000441083	ENSG00000225191	RP11-97C18.1

ENST00000537570	ENSG00000256664	RP11-611O2.3	
ENST00000603703	ENSG00000271052	RP11-479I16.2	
ENST00000604734	ENSG00000270314	GS1-165B14.2	
ENST00000451535	ENSG00000225728	RP6-191P20.3	
ENST00000532878	ENSG00000213333	NPM1P50	
ENST00000413162	ENSG00000227048	RP11-134G8.2	
ENST00000456151	ENSG00000226671	AC005008.3	
ENST00000458303	ENSG00000227505	RP11-434J24.2	
ENST00000423946	ENSG00000237317	RP4-809F4.1	
ENST00000615893	ENSG00000277899	MLECP1	
ENST00000457263	ENSG00000226396	RPS14P3	
ENST00000610379	ENSG00000274024	RP13-210D15.8	
ENST00000603490	ENSG00000271194	RNF138P2	
ENST00000464337	ENSG00000213238	AC008155.2	
ENST00000401435	ENSG00000218748	DBIP1	
ENST00000398565	ENSG00000214549	SDHCP2	
ENST00000525775	ENSG00000254965	RP11-113K21.2	
ENST00000427945	ENSG00000229794	RP11-522L3.11	
ENST00000445574	ENSG00000225154	RP11-184J23.2	
ENST00000436223	ENSG00000237016	AC013410.1	
ENST00000429867	ENSG00000223570	RP11-275I14.2	
ENST00000603085	ENSG00000270934	RP11-346C16.6	
ENST00000421048	ENSG00000233071	RP4-635G19.1	
ENST00000584299	ENSG00000265240	RP11-638L3.2	
ENST00000421444	ENSG00000225116	RP11-137F15.1	
ENST00000430910	ENSG00000240122	FABP5P11	
ENST00000458382	ENSG00000226110	RP11-272G22.2	
ENST00000436691	ENSG00000231930	AF228730.5	
ENST00000471467	ENSG00000240411	RPL5P16	
ENST00000457611	ENSG00000227649	RP11-522L3.9	
ENST00000450968	ENSG00000229010	RP5-979D14.1	
ENST00000604232	ENSG00000270585	RP11-568G11.4	
ENST00000431445	ENSG00000237888	AC087650.1	filled
ENST00000422627	ENSG00000237617	AC013410.2	
ENST00000426018	ENSG00000234886	MTND5P26	
ENST00000426107	ENSG00000232144	PSAT1P2	filled
ENST00000432008	ENSG00000223457	HTATSF1P	
ENST00000497334	ENSG00000242602	CTD-2339M3.1	
ENST00000558213	ENSG00000259512	HNRNPA1P5	
ENST00000511559	ENSG00000248200	RP11-115A14.1	
ENST00000603161	ENSG00000270296	STX8P1	
ENST00000431587	ENSG00000232750	RP11-177A2.5	
ENST00000415533	ENSG00000214074	RPL23AP39	
ENST00000479477	ENSG00000239510	RPL9P5	
ENST00000456025	ENSG00000228551	SNRPGP9	
ENST00000482506	ENSG00000243711	RPL21P116	
ENST00000531560	ENSG00000254954	SLC2A13P1	
ENST00000546853	ENSG00000258148	AC007115.3	
ENST00000546707	ENSG00000258116	RP11-493L12.6	
ENST00000416042	ENSG00000228098	AC110620.1	
ENST00000452925	ENSG00000234682	AC110620.2	
ENST00000419089	ENSG00000225071	GS1-184P14.2	
ENST00000423896	ENSG00000234071	RP11-420H19.1	
ENST00000480199	ENSG00000239198	RPL5P22	
ENST00000491037	ENSG00000234005	GAPDHP22	
ENST00000622243	ENSG00000275400	RP4-756H11.5	
ENST00000426407	ENSG00000215197	PGAM4P1	
ENST00000505836	ENSG00000248196	RP11-476C8.2	
ENST00000520784	ENSG00000253114	RP11-550I15.1	
ENST00000426454	ENSG00000236973	GAPDHP51	

ENST00000417082	ENSG00000229256	ST13P13	
ENST00000572295	ENSG00000262767	GEMIN2P1	
ENST00000377927	ENSG00000204915	RP6-99M1.1	
ENST00000504007	ENSG00000249372	ATP6V1G1P6	
ENST00000603149	ENSG00000271269	RP11-317P15.6	
ENST00000605108	ENSG00000271175	RP11-434J24.3	
ENST00000438916	ENSG00000235616	ST13P2	
ENST00000517577	ENSG00000237264	FTH1P11	
ENST00000603096	ENSG00000271693	CTD-2194M22.1	
ENST00000407097	ENSG00000219986	BTF3P7	
ENST00000416048	ENSG00000238070	SAR1P1	
ENST00000412537	ENSG00000225364	ATP6V0E1P1	
ENST00000460876	ENSG00000223877	RP11-2F9.3	
ENST00000605443	ENSG00000270981	RP11-550I24.3	
ENST00000480875	ENSG00000244538	RP11-10G12.1	
ENST00000416788	ENSG00000235163	RP11-410C4.1	
ENST00000510683	ENSG00000250219	LTV1P1	
ENST00000522790	ENSG00000253383	RP11-116B19.2	
ENST00000439608	ENSG00000231222	ARF4P4	
ENST00000436312	ENSG00000224630	RP1-169P22.2	
ENST00000576223	ENSG00000262994	RP11-216N14.10	
ENST00000481963	ENSG00000230507	RPL7AP8	
ENST00000567049	ENSG00000260113	RP11-146F11.2	
ENST00000603972	ENSG00000270458	RP11-644F5.17	
ENST00000604389	ENSG00000270243	RP11-779O18.4	
ENST00000530974	ENSG00000255266	RP11-734C14.2	
ENST00000424795	ENSG00000236998	RP11-203L2.3	
ENST00000579735	ENSG00000265417	RP11-288C17.1	filled
ENST00000433923	ENSG00000235224	RP11-236P24.1	
ENST00000604630	ENSG00000271385	RP11-108M11.3	
ENST00000488067	ENSG00000223416	RPS26P15	
ENST00000611920	ENSG00000274567	RPS27P14	
ENST00000506790	ENSG00000251663	SUMO2P5	
ENST00000448737	ENSG00000234564	HSPA8P20	
ENST00000451820	ENSG00000227721	RPSAP64	
ENST00000518368	ENSG00000253480	RP11-39H3.1	
ENST00000584447	ENSG00000265564	PIGPP4	
ENST00000520803	ENSG00000253721	SUMO2P20	
ENST00000605245	ENSG00000271339	RP11-1114A5.6	
ENST00000612976	ENSG00000275647	RP11-209D20.2	
ENST00000464802	ENSG00000230562	FAM133DP	
ENST00000517657	ENSG00000253760	RP11-48B3.2	
ENST00000403074	ENSG00000219666	RP11-174C7.1	
ENST00000485656	ENSG00000243171	RP11-273P3.1	
ENST00000461396	ENSG00000241991	RP11-95I19.2	
ENST00000463176	ENSG00000244730	RP13-1056D16.2	
ENST00000473673	ENSG00000241281	RP11-364M6.1	
ENST00000454464	ENSG00000223628	AC023449.2	
ENST00000447169	ENSG00000215286	RP5-1158E12.2	
ENST00000443851	ENSG00000230249	RP13-824C8.2	
ENST00000424559	ENSG00000235776	AC000089.3	
ENST00000529830	ENSG00000254672	RP5-916O11.3	
ENST00000455641	ENSG00000235684	HTATSF1P	
ENST00000437448	ENSG00000227326	RPL7AP7	
ENST00000490729	ENSG00000241590	RPL17P37	
ENST00000425391	ENSG00000223455	RP11-781P14.3	
ENST00000482972	ENSG00000242208	RPL5P29	
ENST00000449885	ENSG00000229806	RPS15P5	
ENST00000426640	ENSG00000237140	HSPE1P16	
ENST00000556286	ENSG00000259148	RP11-322L17.1	

ENST00000427891	ENSG00000237278	RLIMP2	
ENST00000579008	ENSG00000266613	RFWD2P1	
ENST00000541658	ENSG00000256243	RPL7AP3	
ENST00000611521	ENSG00000273806	RP11-439K3.4	
ENST00000402041	ENSG00000217089	RPS29P13	
ENST00000564266	ENSG00000260255	RP11-185O21.1	
ENST00000438811	ENSG00000224573	AC083863.5	
ENST00000406671	ENSG00000220744	RPL5P18	
ENST00000478778	ENSG00000239528	RPS14P8	
ENST00000555153	ENSG00000258421	FRDAP	
ENST00000483907	ENSG00000242229	RPS3AP14	
ENST00000517442	ENSG00000253225	RP11-1057N3.2	
ENST00000603988	ENSG00000271243	RP11-355B11.1	filled
ENST00000464563	ENSG00000241678	RP11-732A19.1	
ENST00000454246	ENSG00000235015	GEMIN2P1	
ENST00000406301	ENSG00000220125	MRPL32P1	
ENST00000506228	ENSG00000251001	CTD-2193P3.1	
ENST00000442212	ENSG00000231480	SNRPGP13	
ENST00000615912	ENSG00000273569	CTC-320C6.1	
ENST00000529855	ENSG00000254736	RP11-867G23.9	
ENST00000479327	ENSG00000240925	RPS20P31	
ENST00000534107	ENSG00000254601	CYCSP26	
ENST00000413361	ENSG00000225125	RANP4	
ENST00000435591	ENSG00000232142	RP11-367H5.8	
ENST00000405071	ENSG00000219604	RP11-132M7.2	
ENST00000460778	ENSG00000244413	RP11-1057B8.1	
ENST00000445539	ENSG00000237827	RP11-332O19.2	
ENST00000487324	ENSG00000241102	RP11-286H14.2	
ENST00000440331	ENSG00000233278	RPS26P2	
ENST00000605855	ENSG00000271373	NANOGP3	
ENST00000414070	ENSG00000244716	RP11-20O24.4	
ENST00000444364	ENSG00000234624	AC016894.1	
ENST00000407196	ENSG00000218631	RP3-395C13.1	
ENST00000422498	ENSG00000232599	RP1-161N10.1	
ENST00000613393	ENSG00000278317	RP4-655L22.5	
ENST00000555221	ENSG00000258788	RP11-404P21.9	
ENST00000427056	ENSG00000233113	RPL7AP7	
ENST00000506136	ENSG00000250940	RP11-489M13.3	
ENST00000443334	ENSG00000224836	RP11-341G2.3	
ENST00000458300	ENSG00000231328	TPT1P7	
ENST00000412993	ENSG00000229332	PGBD4P8	
ENST00000404881	ENSG00000219619	RP11-126M14.1	
ENST00000425872	ENSG00000238249	HMGN2P17	
ENST00000445592	ENSG00000233462	RPL7AP7	
ENST00000604149	ENSG00000270554	RP11-225N10.3	
ENST00000521343	ENSG00000254195	TPM3P3	
ENST00000554198	ENSG00000258510	RP11-493G17.4	
ENST00000588044	ENSG00000267301	RPL23AP77	
ENST00000422524	ENSG00000223593	AL356806.3	
ENST00000456501	ENSG00000232292	ALDH7A1P2	
ENST00000444514	ENSG00000235369	RPL36AP15	
ENST00000411908	ENSG00000235002	RP11-157D18.2	
ENST00000432585	ENSG00000229982	GTF3AP6	
ENST00000422282	ENSG00000225078	RP11-123N4.4	
ENST00000604597	ENSG00000271511	RP11-712C19.1	
ENST00000446146	ENSG00000233933	RP11-63P12.2	
ENST00000620582	ENSG00000276632	RP11-419N10.6	
ENST00000553854	ENSG00000258841	RP11-226P1.3	
ENST00000509908	ENSG00000250956	CTB-88F18.3	
ENST00000392417	ENSG00000213126	AC092642.1	

ENST00000422587	ENSG00000227207	RPL31P12	
ENST00000448118	ENSG00000236330	RPL5P9	
ENST00000438019	ENSG00000237025	RP1-315G1.1	
ENST00000620422	ENSG00000274506	RP11-1174L13.2	
ENST00000605028	ENSG00000271509	RP11-382A18.3	
ENST00000440420	ENSG00000229949	AC005094.2	
ENST00000457554	ENSG00000234287	RP11-761N21.2	
ENST00000455249	ENSG00000235090	RPL7L1P3	
ENST00000504752	ENSG00000248639	RP11-642E20.2	
ENST00000454879	ENSG00000233562	RP11-548N1.1	
ENST00000492764	ENSG00000241891	RP11-226P1.1	
ENST00000434429	ENSG00000228665	RP11-20O24.1	
ENST00000426826	ENSG00000223945	RP11-458I7.1	
ENST00000437667	ENSG00000229814	RPL35AP21	
ENST00000488297	ENSG00000240083	RPS3AP22	
ENST00000549567	ENSG00000258076	RP11-536C10.14	
ENST00000456044	ENSG00000218198	RPS20P32	
ENST00000611095	ENSG00000274138	HMGN1P12	
ENST00000604431	ENSG00000270683	FAM71BP1	
ENST00000460866	ENSG00000243675	RP11-379F4.1	
ENST00000442718	ENSG00000227430	AC019185.2	
ENST00000548175	ENSG00000257680	RP11-367O10.1	
ENST00000456243	ENSG00000231911	TPRKBP1	
ENST00000524262	ENSG00000213393	RP11-546B8.1	
ENST00000605094	ENSG00000270815	RP11-56O18.1	
ENST00000438127	ENSG00000234268	AP000936.1	
ENST00000397758	ENSG00000214192	UBE2V1P2	
ENST00000393781	ENSG00000213384	EIF4E2P1	
ENST00000581473	ENSG00000264064	RP11-758G22.1	
ENST00000532859	ENSG00000254512	RP11-472I20.2	
ENST00000413370	ENSG00000234466	RP11-266I3.1	
ENST00000584626	ENSG00000264930	RP11-846F4.10	
ENST00000431448	ENSG00000227133	AC011742.5	
ENST00000547218	ENSG00000257998	RP11-361A23.3	
ENST00000528386	ENSG00000255192	NANOGP8	
ENST00000439053	ENSG00000235440	RP11-214J9.1	
ENST00000515267	ENSG00000251220	RFPL4AP3	filled
ENST00000483361	ENSG00000241052	RP11-173D9.1	
ENST00000528260	ENSG00000254792	RP11-119D9.4	
ENST00000603448	ENSG00000271022	RP11-142I2.1	
ENST00000432048	ENSG00000231071	RP5-1068H6.1	
ENST00000416715	ENSG00000232809	VDAC1P10	
ENST00000508874	ENSG00000249286	CTD-2210P15.2	
ENST00000461683	ENSG00000242399	RPS20P23	
ENST00000589465	ENSG00000267678	RP11-15E18.3	
ENST00000481157	ENSG00000243020	RPL7P39	
ENST00000421086	ENSG00000227187	RP11-6C10.1	
ENST00000438801	ENSG00000236407	HMGB1P18	
ENST00000549457	ENSG00000257956	NOP56P3	
ENST00000495117	ENSG00000239659	RPL9P31	
ENST00000439946	ENSG00000228530	RP11-567B20.1	
ENST00000604662	ENSG00000270652	DPRXP5	
ENST00000528228	ENSG00000214366	RP11-138H14.1	
ENST00000416539	ENSG00000229221	HNRNPA1P66	
ENST00000433683	ENSG00000232751	RP11-296O14.1	
ENST00000414815	ENSG00000224669	RP11-561N12.1	
ENST00000509926	ENSG00000251312	AC004062.2	
ENST00000435693	ENSG00000228729	RP11-211A18.2	
ENST00000443069	ENSG00000225254	ARMC8P1	
ENST00000468051	ENSG00000243925	RPS24P18	

ENST00000451989	ENSG00000236554	ASNSP3	
ENST00000398764	ENSG00000214669	RP11-18B16.1	
ENST00000451185	ENSG00000224727	FCF1P7	
ENST00000455505	ENSG00000232334	RP11-47G11.2	
ENST00000496370	ENSG00000244717	RPS27P14	
ENST00000392530	ENSG00000213144	RP11-64B16.2	
ENST00000520029	ENSG00000253623	RP11-346I3.2	
ENST00000424289	ENSG00000223450	RP11-52I18.1	
ENST00000433830	ENSG00000227623	AC073987.2	
ENST00000529959	ENSG00000255254	HIGD1AP5	
ENST00000458678	ENSG00000233615	HNRNPA1P42	
ENST00000616549	ENSG00000277509	RP11-944L7.6	
ENST00000414760	ENSG00000229667	UBE2V1P9	
ENST00000485147	ENSG00000239374	RP11-407P2.1	
ENST00000592379	ENSG00000267135	AD000091.3	
ENST00000401679	ENSG00000220326	RP11-129H15.1	
ENST00000404780	ENSG00000220694	RP3-403A15.1	
ENST00000456388	ENSG00000234044	AC108059.1	
ENST00000492033	ENSG00000244157	RP11-190P13.1	
ENST00000514945	ENSG00000250684	KB-1042C11.1	
ENST00000400275	ENSG00000215414	PSMA6P1	
ENST00000424277	ENSG00000229423	RPL27AP8	
ENST00000482313	ENSG00000241804	RP11-719N22.1	
ENST00000510337	ENSG00000235275	KRT18P16	
ENST00000527837	ENSG00000254455	HIGD1AP10	
ENST00000468292	ENSG00000242261	MGC27345	
ENST00000548110	ENSG00000257609	RP11-275O18.1	
ENST00000474255	ENSG00000239405	TMED10P2	
ENST00000457831	ENSG00000234825	XRCC6P2	filled
ENST00000522230	ENSG00000254331	CKS1BP7	
ENST00000540396	ENSG00000256720	RP11-436I9.6	
ENST00000447284	ENSG00000226003	RP11-312J18.3	
ENST00000430845	ENSG00000225242	COX6B1P7	
ENST00000572651	ENSG00000262986	RP11-785H5.1	
ENST00000552208	ENSG00000257730	LSM6P2	
ENST00000445666	ENSG00000227873	RP3-389A20.2	
ENST00000420608	ENSG00000234524	RPL12P43	
ENST00000514835	ENSG00000248282	RP11-112L18.1	
ENST00000457727	ENSG00000237891	RP11-417D4.1	
ENST00000404020	ENSG00000217495	RP1-95L4.2	
ENST00000526214	ENSG00000212789	ST13P5	
ENST00000416838	ENSG00000214653	HNRNPA3P3	
ENST00000605823	ENSG00000270736	RP6-166C19.19	
ENST00000517470	ENSG00000253638	CTC-369M3.1	
ENST00000426359	ENSG00000228191	RP11-345I18.6	
ENST00000526967	ENSG00000255280	CTD-2530H12.5	
ENST00000519377	ENSG00000253580	RP11-790J24.2	
ENST00000553035	ENSG00000257820	MRPS6P4	
ENST00000502546	ENSG00000251436	NUPL1P1	
ENST00000476875	ENSG00000180150	HMGN2P9	
ENST00000555346	ENSG00000258381	RP11-737F10.1	
ENST00000439651	ENSG00000229518	UBE2V1P3	
ENST00000604297	ENSG00000270499	MKI67P1	
ENST00000537926	ENSG00000257087	RP11-136I14.3	
ENST00000605421	ENSG00000270879	RP11-210K20.4	filled
ENST00000619794	ENSG00000276486	RP11-210K20.6	
ENST00000446186	ENSG00000232392	AC002366.3	
ENST00000534518	ENSG00000224829	TOMM20P1	
ENST00000381357	ENSG00000229652	RP11-435P24.2	
ENST00000472609	ENSG00000220831	NDUFA5P9	

ENST00000429025	ENSG00000236132	CTA-440B3.1	
ENST00000465034	ENSG00000244217	RPS4XP10	
ENST00000503819	ENSG00000248791	CTD-2165H16.3	
ENST00000550830	ENSG00000257159	RP11-58A17.3	filled
ENST00000421785	ENSG00000237959	RPL36AP10	
ENST00000407371	ENSG00000218965	NACAP7	
ENST00000411919	ENSG00000241975	TCEB1P19	
ENST00000575927	ENSG00000262870	CYCSP40	
ENST00000547343	ENSG00000257649	METTL7AP1	
ENST00000536166	ENSG00000256827	RP11-214K3.5	
ENST00000534226	ENSG00000135477	KRT87P	filled
ENST00000508551	ENSG00000250959	GLUD1P3	
ENST00000360035	ENSG00000196970	NXF4	
ENST00000570871	ENSG00000262096	PCDHB19P	
ENST00000625133	ENSG00000262096	PCDHB19P	
ENST00000503038	ENSG00000245958	RP11-33B1.1	
ENST00000448860	ENSG00000186163	TRY2P	
ENST00000343738	ENSG00000250251	PKD1P6	filled
ENST00000537668	ENSG00000256594	RP11-705C15.2	
ENST00000532103	ENSG00000254840	RP11-574M7.1	
ENST00000370638	ENSG00000122432	SPATA1	filled
ENST00000542490	ENSG00000177359	RP11-551L14.1	
ENST00000603640	ENSG00000187791	FAM205CP	
ENST00000562418	ENSG00000237223	SULT1C2P1	
ENST00000552999	ENSG00000257896	RP11-210N13.1	
ENST00000549403	ENSG00000257896	RP11-210N13.1	
ENST00000614323	ENSG00000276005	RP11-578F21.1	
ENST00000618089	ENSG00000276005	RP11-578F21.1	
ENST00000431070	ENSG00000229009	TMPRSS11GP	
ENST00000502496	ENSG00000229009	TMPRSS11GP	
ENST00000572770	ENSG00000260734	RP11-510M2.4	
ENST00000526769	ENSG00000254838	GVINP1	
ENST00000531871	ENSG00000254838	GVINP1	
ENST00000601044	ENSG00000269021	CTD-3187F8.12	
ENST00000436375	ENSG00000171658	RP11-443P15.2	filled
ENST00000416764	ENSG00000171658	RP11-443P15.2	filled
ENST00000445507	ENSG00000171658	RP11-443P15.2	filled
ENST00000306399	ENSG00000171658	RP11-443P15.2	filled
ENST00000480251	ENSG00000242595	RP11-648L3.1	
ENST00000567392	ENSG00000261778	RP5-1173P7.1	
ENST00000460165	ENSG00000241362	RPL36AP43	
ENST00000498515	ENSG00000243945	RP11-696F10.1	
ENST00000435020	ENSG00000226093	RPS28P8	
ENST00000433823	ENSG00000227168	RPL35AP4	
ENST00000519012	ENSG00000253997	RP11-200A13.3	
ENST00000486139	ENSG00000240364	RPL31P59	
ENST00000416467	ENSG00000232665	PHBP10	
ENST00000340589	ENSG00000249934	RP11-466G12.3	
ENST00000455244	ENSG00000226549	SCDP1	
ENST00000423991	ENSG00000228337	RP11-804H8.5	
ENST00000604840	ENSG00000271491	RP11-787P24.4	
ENST00000435088	ENSG00000229197	RP11-227H15.7	
ENST00000505438	ENSG00000251473	AC004069.1	
ENST00000486977	ENSG00000240759	RP11-25I15.1	
ENST00000425971	ENSG00000226837	HMGB1P32	
ENST00000605525	ENSG00000271710	RP11-312P12.3	
ENST00000505780	ENSG00000250895	CTD-2158P22.4	
ENST00000407947	ENSG00000219669	BECN1P2	
ENST00000475327	ENSG00000242703	CCT4P1	
ENST00000412007	ENSG00000225644	RPL35AP4	

ENST00000621502	ENSG00000276374	RP11-1E11.1
ENST00000406554	ENSG00000220522	RP1-177A13.1
ENST00000411661	ENSG00000224618	AC096921.2
ENST00000411498	ENSG00000226773	RP4-775D17.1
ENST00000511173	ENSG00000250161	TRMT112P5
ENST00000476981	ENSG00000243607	RPL35AP26
ENST00000525759	ENSG00000255450	CTD-2063L20.1
ENST00000404509	ENSG00000216365	RPL37P15
ENST00000450604	ENSG00000225925	RP13-313G19.2
ENST00000406783	ENSG00000218991	CCNG1P1
ENST00000435808	ENSG00000231060	FARSBP1
ENST00000434169	ENSG00000225176	ATP5LP4
ENST00000475149	ENSG00000243855	RP11-114O13.1
ENST00000332869	ENSG00000183055	FAM133CP
ENST00000567372	ENSG00000260747	RP11-421N8.1
ENST00000551999	ENSG00000257751	RP11-536C10.21
ENST00000439303	ENSG00000229919	TCEB1P3
ENST00000422710	ENSG00000236851	DHFRP2
ENST00000569805	ENSG00000261284	RBM22P13
ENST00000434960	ENSG00000229946	B3GNT2P1
ENST00000556233	ENSG00000258962	RP11-747H7.1
ENST00000447612	ENSG00000224545	AC008264.4
ENST00000622748	ENSG00000276915	RP4-775C13.3
ENST00000444423	ENSG00000235817	RP11-543E8.2
ENST00000507713	ENSG00000225165	CTD-2538A21.1
ENST00000425543	ENSG00000230609	RP11-167P22.4
ENST00000520139	ENSG00000253620	AC144568.4
ENST00000430732	ENSG00000227030	RPL7AP7
ENST00000401791	ENSG00000217231	RP3-340H11.2
ENST00000396959	ENSG00000213943	KRT18P17
ENST00000460666	ENSG00000243759	ST13P15
ENST00000613800	ENSG00000278688	RP11-1E11.2
ENST00000454247	ENSG00000223753	GHc-602D8.2
ENST00000406097	ENSG00000219302	RP11-174C7.3
ENST00000422792	ENSG00000236549	AC079807.3
ENST00000512222	ENSG00000250826	HNRNPA3P13
ENST00000402650	ENSG00000218350	LYPLA1P3
ENST00000603779	ENSG00000270772	RP11-332H21.2
ENST00000451600	ENSG00000233982	RPL7AP7
ENST00000448403	ENSG00000213041	RP11-383G10.3
ENST00000437306	ENSG00000237536	HTATSF1P
ENST00000419124	ENSG00000224443	AC006509.4
ENST00000438685	ENSG00000233716	AC074367.1
ENST00000604659	ENSG00000270328	RP11-33E15.1
ENST00000463499	ENSG00000240167	RPS7P7
ENST00000521306	ENSG00000254310	CTD-2045M21.1
ENST00000453699	ENSG00000228600	POLR2CP
ENST00000439448	ENSG00000250461	RP11-631M6.2
ENST00000448271	ENSG00000229939	RP11-111F16.2
ENST00000456717	ENSG00000226470	RP11-132E11.3
ENST00000431951	ENSG00000234587	MRPL50P1
ENST00000442064	ENSG00000230191	RP4-725G10.3
ENST00000605196	ENSG00000270893	RP11-311B14.1
ENST00000596823	ENSG00000268407	AC006115.6
ENST00000430744	ENSG00000232540	RPL36P19
ENST00000511389	ENSG00000249888	RP11-447H19.2
ENST00000438629	ENSG00000233551	LSM1P1
ENST00000545836	ENSG00000255664	ARL6IP1P1
ENST00000468402	ENSG00000230886	HMGB1P25
ENST00000454851	ENSG00000225286	AC005105.2

ENST00000419363	ENSG00000226491	FTOP1	
ENST00000448352	ENSG00000226132	RPS3AP46	
ENST00000392260	ENSG00000213091	PHBP1	
ENST00000555477	ENSG00000258722	CKAP2P1	
ENST00000441775	ENSG00000226970	RP11-82H13.2	filled
ENST00000545370	ENSG00000256004	RP11-20D14.4	
ENST00000515537	ENSG00000213716	FABP5P5	
ENST00000555940	ENSG00000258980	EIF1AXP2	
ENST00000416876	ENSG00000225658	TAF13P2	
ENST00000493462	ENSG00000216718	RP3-522P13.1	
ENST00000460914	ENSG00000241993	RPL38P1	
ENST00000619013	ENSG00000278275	RP11-55J15.2	
ENST00000582212	ENSG00000266486	FAM106CP	
ENST00000427711	ENSG00000213987	RP11-399E6.2	
ENST00000546640	ENSG00000257773	ST13P3	
ENST00000502313	ENSG00000248909	HMGB1P21	
ENST00000560574	ENSG00000259722	RP11-94P14.1	
ENST00000452981	ENSG00000230112	RP11-56H7.2	
ENST00000602494	ENSG00000269907	RP11-330M2.4	
ENST00000505367	ENSG00000251591	CTD-2197M16.1	
ENST00000435639	ENSG00000230405	RPS3AP52	
ENST00000443050	ENSG00000225711	RP11-345I18.4	
ENST00000603201	ENSG00000271021	RP5-878I13.2	
ENST00000424262	ENSG00000223640	RPL30P3	
ENST00000448218	ENSG00000237752	RP13-75G22.1	
ENST00000467217	ENSG00000242123	RP11-51L5.1	
ENST00000538746	ENSG00000256079	RP11-318G8.2	
ENST00000565251	ENSG00000260483	RP11-151H2.1	
ENST00000604059	ENSG00000271211	RP6-166C19.20	
ENST00000587507	ENSG00000267558	RP11-618K16.4	
ENST00000507834	ENSG00000248725	RP11-218C23.1	
ENST00000603134	ENSG00000271286	LL0XNC01-39B3.1	
ENST00000603395	ENSG00000271047	RP11-760D2.12	
ENST00000434191	ENSG00000219870	RP11-430A19.2	
ENST00000411777	ENSG00000229906	SNRPGP11	
ENST00000603734	ENSG00000270343	UNGP3	
ENST00000401429	ENSG00000226790	HNRNPA3P1	
ENST00000421404	ENSG00000236570	RAD23BP1	
ENST00000501221	ENSG00000247763	RP11-447H19.1	
ENST00000465511	ENSG00000243431	RPL5P30	
ENST00000424932	ENSG00000178556	CKS1BP6	
ENST00000495704	ENSG00000241429	EEF1A1P25	
ENST00000429211	ENSG00000226814	RP3-419C19.2	
ENST00000605375	ENSG00000271657	RP11-270B14.1	
ENST00000487630	ENSG00000242321	RPL23AP40	
ENST00000429983	ENSG00000179460	EEF1A1P27	
ENST00000531054	ENSG00000254621	SETP16	
ENST00000604344	ENSG00000270524	QTRT1P1	
ENST00000427962	ENSG00000224800	RP11-235D19.2	
ENST00000427200	ENSG00000233752	AC009414.1	
ENST00000422893	ENSG00000236870	RP1-223D17.1	
ENST00000490892	ENSG00000220556	RP1-202I21.3	
ENST00000442667	ENSG00000229029	CDCA4P1	
ENST00000463344	ENSG00000237490	RP13-926M18.1	
ENST00000570155	ENSG00000261047	AC137527.2	
ENST00000412936	ENSG00000237801	AMDP1	
ENST00000493346	ENSG00000244652	RP11-93B21.1	
ENST00000435255	ENSG00000232114	AC018693.5	
ENST00000604497	ENSG00000270682	KB-1907C4.2	
ENST00000446973	ENSG00000228667	RP11-129O7.2	

ENST00000446889	ENSG00000231490	RPL7L1P2	
ENST00000604318	ENSG00000271537	RP11-365F18.6	
ENST00000435130	ENSG00000224685	RP11-84O12.3	
ENST00000405166	ENSG00000218803	GSTM2P1	
ENST00000559021	ENSG00000119660	DPPA5P4	
ENST00000603130	ENSG00000270507	CTA-21C21.1	
ENST00000415219	ENSG00000230053	RP11-76N22.1	
ENST00000456945	ENSG00000229530	RP11-62C3.8	
ENST00000456549	ENSG00000232834	RP11-12D5.3	
ENST00000402485	ENSG00000217379	RP11-254A17.1	
ENST00000406512	ENSG00000218213	FTH1P26	
ENST00000616705	ENSG00000278541	RP11-467N20.8	
ENST00000514075	ENSG00000250471	GMPSP1	
ENST00000495378	ENSG00000241782	RP11-91P24.1	
ENST00000416146	ENSG00000233829	AC017078.1	
ENST00000411748	ENSG00000223514	AC004866.1	
ENST00000496058	ENSG00000240669	RP11-555K12.2	
ENST00000498296	ENSG00000239780	RPLP0P11	
ENST00000485809	ENSG00000243355	RP11-57G22.1	
ENST00000457414	ENSG00000226538	RPL35AP4	
ENST00000560892	ENSG00000259710	NUTF2P6	
ENST00000509054	ENSG00000250319	CTD-2272G21.3	
ENST00000426040	ENSG00000223968	AC098614.1	
ENST00000604478	ENSG00000271543	RP11-692M12.5	
ENST00000522976	ENSG00000253271	RP11-1059L18.1	
ENST00000508597	ENSG00000248956	HMGB1P44	
ENST00000424041	ENSG00000213979	RPL7AP14	
ENST00000428657	ENSG00000237260	AC073069.2	
ENST00000480579	ENSG00000242206	RPS26P35	
ENST00000453325	ENSG00000231750	NANOGP10	
ENST00000495837	ENSG00000241112	RPL29P14	
ENST00000455312	ENSG00000237141	DNAJC19P1	
ENST00000604565	ENSG00000270425	CTA-369K23.1	
ENST00000530091	ENSG00000254915	RP11-263C24.3	
ENST00000583992	ENSG00000264186	SNRPCP4	
ENST00000436983	ENSG00000224584	RP11-365D23.2	
ENST00000404107	ENSG00000220181	RP11-486M3.2	
ENST00000423552	ENSG00000229090	RP1-232L22_A.1	
ENST00000477943	ENSG00000242071	RPL7AP6	
ENST00000448158	ENSG00000237249	RP1-102G20.2	
ENST00000604518	ENSG00000271184	RP4-800O15.3	
ENST00000523157	ENSG00000253777	RP11-758M4.3	
ENST00000406538	ENSG00000218549	OR4K12P	
ENST00000490908	ENSG00000243720	RPL21P127	
ENST00000415070	ENSG00000228499	TMSB10P1	
ENST00000438345	ENSG00000234276	BX664724.4	
ENST00000447425	ENSG00000234513	AC073072.7	
ENST00000576545	ENSG00000262495	RP11-46I8.1	
ENST00000470205	ENSG00000212829	RPS26P3	
ENST00000418190	ENSG00000226795	SAR1P1	
ENST00000572683	ENSG00000262819	RP11-58A17.3	filled
ENST00000573097	ENSG00000262082	RP11-58A17.2	
ENST00000473808	ENSG00000244527	RP11-254G11.1	
ENST00000605601	ENSG00000271508	RP11-73B8.3	
ENST00000582646	ENSG00000264189	CTD-2533G20.1	
ENST00000438855	ENSG00000226579	RP11-351K23.3	
ENST00000442570	ENSG00000234889	RP13-130D24.1	
ENST00000486904	ENSG00000242431	RP11-731J8.1	
ENST00000405705	ENSG00000218536	AP002530.2	
ENST00000517342	ENSG00000253541	SEPT10P1	

ENST00000520478	ENSG00000254193	RP11-585F1.8	
ENST00000521633	ENSG00000254190	RP11-267M23.5	
ENST00000584353	ENSG00000264373	RP11-227G15.6	
ENST00000454899	ENSG00000235730	OR2AF1P	
ENST00000434035	ENSG00000237041	AC007679.4	
ENST00000514243	ENSG00000251014	RP11-159K7.1	
ENST00000603116	ENSG00000270916	RP4-741O10.1	
ENST00000414863	ENSG00000236243	RPL6P29	
ENST00000481681	ENSG00000224993	RPL29P12	filled
ENST00000422868	ENSG00000236594	RPS27AP14	
ENST00000510606	ENSG00000248387	RP11-781M16.1	
ENST00000603803	ENSG00000270510	RP11-777F6.3	
ENST00000603137	ENSG00000270528	RP11-674I16.2	
ENST00000404531	ENSG00000217078	RP1-13D10.3	
ENST00000582896	ENSG00000263729	RP11-746M1.8	
ENST00000438001	ENSG00000226345	AC083822.2	
ENST00000612100	ENSG00000275953	RP11-753G20.2	
ENST00000611308	ENSG00000273595	RP11-707E21.1	
ENST00000548906	ENSG00000257643	RP11-278C7.2	
ENST00000418231	ENSG00000232928	DDX3YP1	
ENST00000468834	ENSG00000235962	RP11-462L8.2	
ENST00000426795	ENSG00000231592	SAR1P1	
ENST00000422184	ENSG00000234163	RP11-479G22.6	
ENST00000616480	ENSG00000273997	RP11-361F15.5	
ENST00000454105	ENSG00000229629	RP11-302K17.4	
ENST00000534879	ENSG00000255995	HPRTP4	
ENST00000456802	ENSG00000231376	HMGN2P16	
ENST00000605879	ENSG00000271075	RP11-589M4.4	
ENST00000427776	ENSG00000179131	RP11-458F8.3	
ENST00000414224	ENSG00000228432	DHFRP2	
ENST00000600955	ENSG00000227311	RP1-228H13.1	
ENST00000425328	ENSG00000235951	AC009965.1	
ENST00000404520	ENSG00000217377	AK4P5	
ENST00000603879	ENSG00000271322	RP11-390F10.3	
ENST00000555741	ENSG00000259046	RP11-857B24.2	
ENST00000485187	ENSG00000241905	RP11-305O4.1	
ENST00000425086	ENSG00000225304	AC106901.1	
ENST00000605576	ENSG00000271588	LARP7P1	
ENST00000430476	ENSG00000237182	RP11-402P6.3	
ENST00000604690	ENSG00000270324	RP11-180P8.4	
ENST00000606304	ENSG00000272164	RP11-180P8.5	
ENST00000470544	ENSG00000242083	RPL7AP31	
ENST00000418634	ENSG00000235264	RPL5P28	
ENST00000442175	ENSG00000228847	ATP5G2P4	
ENST00000373465	ENSG00000231235	RP11-75A9.1	
ENST00000437678	ENSG00000229800	ATP8A2P2	
ENST00000522981	ENSG00000254253	RP11-343B22.2	
ENST00000440153	ENSG00000230755	RP11-343J3.7	
ENST00000561045	ENSG00000259266	RP11-183E24.1	
ENST00000580616	ENSG00000263748	EXOGP1	
ENST00000449233	ENSG00000233727	RP11-380I20.2	
ENST00000584143	ENSG00000266003	RP11-267C16.2	
ENST00000490943	ENSG00000231643	GHc-210E9.2	
ENST00000299783	ENSG00000235945	AC002543.2	
ENST00000529668	ENSG00000255377	DUXAP5	
ENST00000494644	ENSG00000220349	RP3-431A14.4	
ENST00000559378	ENSG00000259246	HMGN2P47	
ENST00000603845	ENSG00000271468	RP11-14N4.1	
ENST00000605353	ENSG00000270771	RP6-166C19.12	
ENST00000486611	ENSG00000243730	RPL29P3	

ENST00000604447	ENSG00000271459	RP4-560B9.6
ENST00000493810	ENSG00000240471	PHBP8
ENST00000603287	ENSG00000270914	RP5-1077B9.5
ENST00000432828	ENSG00000223777	AC092162.2
ENST00000422551	ENSG00000229032	RP11-91A18.1
ENST00000438507	ENSG00000225622	PPIAP17
ENST00000411632	ENSG00000226880	XX-2136C48.7
ENST00000446414	ENSG00000233473	RAD1P2
ENST00000439038	ENSG00000213854	CNN2P6
ENST00000550105	ENSG00000257146	RP11-813P10.1
ENST00000423226	ENSG00000214035	AC073310.4
ENST00000326586	ENSG00000181524	RPL24P4
ENST00000521552	ENSG00000254200	RPL7AP33
ENST00000478270	ENSG00000214405	RAP1BP2
ENST00000415667	ENSG00000225701	EIF4A1P13
ENST00000519284	ENSG00000253814	MRPS36P3
ENST00000406535	ENSG00000218813	RP11-59D5_B.3
ENST00000430062	ENSG00000231341	VDAC1P6
ENST00000428087	ENSG00000233940	RP11-248I9.2
ENST00000423216	ENSG00000237749	RP3-423B22.5
ENST00000460608	ENSG00000240729	CTD-2301A4.3
ENST00000522241	ENSG00000254091	RP11-10A14.8
ENST00000430685	ENSG00000234017	RP11-214N15.5
ENST00000428620	ENSG00000228825	LAMTOR3P1
ENST00000400121	ENSG00000215357	HSPD1P8
ENST00000415692	ENSG00000227805	RP11-248J23.5
ENST00000475771	ENSG00000240511	MED28P2
ENST00000604050	ENSG00000270352	RP11-111N20.4
ENST00000431784	ENSG00000230003	RP11-217F16.1
ENST00000437256	ENSG00000230671	NDUFS5P5
ENST00000418056	ENSG00000233263	AC009518.2
ENST00000403169	ENSG00000216364	MRPL42P2
ENST00000465295	ENSG00000242747	RP11-30K9.1
ENST00000605239	ENSG00000270775	AP000436.4
ENST00000453859	ENSG00000237178	AC019080.1
ENST00000439110	ENSG00000237000	PTMAP6
ENST00000437364	ENSG00000238145	RP11-346M5.1
ENST00000508796	ENSG00000249520	RP11-386B13.1
ENST00000551909	ENSG00000257927	MRPS36P5
ENST00000436597	ENSG00000224061	AC092106.1
ENST00000322792	ENSG00000215900	SEPW1P
ENST00000414330	ENSG00000225934	RP4-566D2.1
ENST00000456632	ENSG00000234322	ST13P18
ENST00000447781	ENSG00000237008	AL591704.5
ENST00000603059	ENSG00000270813	NANOGNBP3
ENST00000446367	ENSG00000237148	HIGD1AP2
ENST00000418991	ENSG00000227864	ARL5AP1
ENST00000446395	ENSG00000235061	UBE2V1P7
ENST00000604803	ENSG00000270411	RP13-379L11.3
ENST00000433254	ENSG00000236121	HAUS6P2
ENST00000605544	ENSG00000271609	CTB-31N19.4
ENST00000620999	ENSG00000273763	RP11-420C9.1
ENST00000433725	ENSG00000230820	AC000362.1
ENST00000421400	ENSG00000234881	PIGFP2
ENST00000605022	ENSG00000270923	TAS2R6P
ENST00000434165	ENSG00000230495	RP11-462D18.2
ENST00000415208	ENSG00000226665	SSR1P2
ENST00000456830	ENSG00000224121	ATG12P2
ENST00000426223	ENSG00000225843	NIPA2P1
ENST00000426436	ENSG00000237226	RPS3AP39

ENST00000407035	ENSG00000219712	RP11-532F6.2
ENST00000310599	ENSG00000174572	RP11-209A2.1
ENST00000436166	ENSG00000234251	HTATSF1P
ENST00000605253	ENSG00000270646	RP6-166C19.15
ENST00000321498	ENSG00000180019	AC079741.2
ENST00000621061	ENSG00000275432	CTD-3093B17.2
ENST00000456719	ENSG00000213486	AC108039.1
ENST00000605250	ENSG00000270323	AP000860.2
ENST00000603920	ENSG00000270917	RP11-27I1.6
ENST00000522430	ENSG00000225259	ST13P6
ENST00000441193	ENSG00000234639	AC009263.2
ENST00000622603	ENSG00000274901	LL0XNC01-220B11.1
ENST00000434771	ENSG00000231931	AC024082.4
ENST00000482905	ENSG00000244582	RPL21P120
ENST00000603171	ENSG00000271232	CARSP1
ENST00000512377	ENSG00000248375	RP11-177B4.1
ENST00000567026	ENSG00000261234	RP11-356O24.1
ENST00000614532	ENSG00000274256	RP1-229K20.8
ENST00000453148	ENSG00000225027	IFNWP4
ENST00000618821	ENSG00000275980	RP11-113D19.9
ENST00000552900	ENSG00000258262	RP11-693J15.3
ENST00000532188	ENSG00000254784	LRRC6P1
ENST00000425216	ENSG00000235803	RP11-343J3.6
ENST00000438336	ENSG00000227645	RPL7P54
ENST00000612158	ENSG00000278745	RP1-229K20.9
ENST00000489238	ENSG00000242911	RP11-118N24.2
ENST00000451124	ENSG00000205433	SNRPGP6
ENST00000420113	ENSG00000237549	AC092570.3
ENST00000468671	ENSG00000241932	RP11-14K2.1
ENST00000452117	ENSG00000232556	AC092570.4
ENST00000429076	ENSG00000229288	RP11-1E11.2
ENST00000517555	ENSG00000254358	CYCSP22
ENST00000449183	ENSG00000236783	RPS15AP27
ENST00000366529	ENSG00000213025	COX20P1
ENST00000558299	ENSG00000259324	OR11K1P
ENST00000399891	ENSG00000215263	AC025750.7
ENST00000554547	ENSG00000258721	OR11H3P
ENST00000561335	ENSG00000259231	CTD-2014N11.2
ENST00000529541	ENSG00000228901	HMGN2P36
ENST00000605362	ENSG00000271242	SLMO2P2
ENST00000605804	ENSG00000270497	RP1-290F12.3
ENST00000588490	ENSG00000175886	RPL7AP66
ENST00000456484	ENSG00000224340	RP11-496H15.2
ENST00000576277	ENSG00000262990	CTC-281F24.2
ENST00000445880	ENSG00000230478	RP1-52D1.1
ENST00000407792	ENSG00000219757	RP11-793L10.1
ENST00000584430	ENSG00000264179	RP11-672L10.5
ENST00000505179	ENSG00000251050	RP11-168A11.4
ENST00000538792	ENSG00000256171	GCSHP4
ENST00000578682	ENSG00000265869	RP11-76K13.2
ENST00000517332	ENSG00000254131	RP11-1007J8.1
ENST00000472363	ENSG00000240873	RPS29P22
ENST00000526365	ENSG00000254582	PSMA2P1
ENST00000428299	ENSG00000224001	RP11-270F18.2
ENST00000453847	ENSG00000183586	HMGN3P1
ENST00000517591	ENSG00000254336	AC008694.2
ENST00000620894	ENSG00000273849	ATP5A1P10
ENST00000546447	ENSG00000257648	CYCSP30
ENST00000478069	ENSG00000241103	RP11-398J16.1
ENST00000434465	ENSG00000227062	RP11-115A15.4

ENST00000438601	ENSG00000229820	RP1-308E4.1	
ENST00000397729	ENSG00000243014	PTMAP8	
ENST00000392131	ENSG00000213069	KRT8P40	
ENST00000444678	ENSG00000223375	RP11-224O19.5	
ENST00000449328	ENSG00000232508	MRPL45P1	
ENST00000496941	ENSG00000239327	RP11-14J7.1	
ENST00000498779	ENSG00000244043	RPS27P27	
ENST00000490136	ENSG00000242285	RPL6P8	
ENST00000507884	ENSG00000251668	RP11-466P24.5	
ENST00000506772	ENSG00000249014	HMGN2P4	
ENST00000603247	ENSG00000271625	PSMA6P4	
ENST00000505398	ENSG00000250324	MRPL22P1	
ENST00000395416	ENSG00000213548	AC005522.6	
ENST00000504890	ENSG00000248120	RP11-301A5.2	
ENST00000458172	ENSG00000176268	CYCSP34	
ENST00000603762	ENSG00000270625	RP4-555N2.4	
ENST00000418311	ENSG00000213183	RPS7P8	
ENST00000511690	ENSG00000251553	CTC-463N11.1	filled
ENST00000603209	ENSG00000270289	RP11-512G4.1	
ENST00000605620	ENSG00000270865	KB-1836B5.3	
ENST00000529697	ENSG00000254957	RP11-179A10.2	
ENST00000432156	ENSG00000238013	RP11-427P5.2	
ENST00000446953	ENSG00000229700	RP1-130G2.1	filled
ENST00000591692	ENSG00000267005	AC002984.2	
ENST00000584914	ENSG00000263505	RP11-78F17.3	
ENST00000558620	ENSG00000259601	RP11-568G20.2	filled
ENST00000522871	ENSG00000253536	RP11-1174L13.2	
ENST00000436573	ENSG00000249947	XBP1P1	
ENST00000438784	ENSG00000228744	RPS3AP30	
ENST00000603741	ENSG00000270734	RP11-719K4.7	
ENST00000438690	ENSG00000228464	RP11-617O8.1	
ENST00000568401	ENSG00000261391	RP11-586K12.7	
ENST00000451332	ENSG00000229822	RP5-1174J21.2	
ENST00000508068	ENSG00000251638	AC006390.4	
ENST00000508958	ENSG00000248400	ST13P12	
ENST00000444831	ENSG00000237024	RPL21P33	
ENST00000430567	ENSG00000228611	HNF4GP1	
ENST00000423801	ENSG00000184423	AC090043.1	
ENST00000610373	ENSG00000276507	RP11-769N22.2	
ENST00000451503	ENSG00000224162	UQCRHP3	
ENST00000428627	ENSG00000231198	AC004987.10	
ENST00000528859	ENSG00000255138	GLTPP1	
ENST00000458741	ENSG00000226222	RP11-753E22.3	
ENST00000503783	ENSG00000250678	CTB-161M19.1	
ENST00000486538	ENSG00000242445	RPL7AP11	
ENST00000555161	ENSG00000259105	RPS3AP4	
ENST00000397383	ENSG00000226693	NXNP1	
ENST00000441918	ENSG00000227592	PIGFP3	
ENST00000619270	ENSG00000275506	CTD-2062A1.2	
ENST00000484754	ENSG00000224085	RP11-4H14.1	
ENST00000512187	ENSG00000249213	RP11-98P2.1	
ENST00000445789	ENSG00000226657	SAR1P1	
ENST00000497656	ENSG00000243920	RPS26P24	
ENST00000525311	ENSG00000213785	RP11-449L13.2	
ENST00000424779	ENSG00000224072	RP11-75A9.3	
ENST00000436861	ENSG00000228663	PSMD10P1	
ENST00000509365	ENSG00000249600	RP11-510I6.3	
ENST00000444622	ENSG00000225949	RP3-358H7.1	
ENST00000469685	ENSG00000243859	RPL5P17	
ENST00000488313	ENSG00000239686	RP11-665C16.1	

ENST00000612290	ENSG00000273813	RP11-11A9.2	
ENST00000494583	ENSG00000240043	RPS27P26	
ENST00000492984	ENSG00000244630	RP11-241J12.1	
ENST00000465655	ENSG00000244432	RPL39P28	
ENST00000419030	ENSG00000227887	RPS26P13	
ENST00000468501	ENSG00000241385	RP11-25D10.1	
ENST00000530827	ENSG00000255286	RP11-708L7.7	
ENST00000445465	ENSG00000230785	AC006326.3	
ENST00000428525	ENSG00000224553	AC008065.1	
ENST00000464297	ENSG00000237107	RP11-256G5.1	
ENST00000412724	ENSG00000226536	SETP15	
ENST00000443304	ENSG00000237555	RP5-862K6.4	
ENST00000449607	ENSG00000224589	RP11-12D5.6	
ENST00000611241	ENSG00000277711	RP11-378I6.3	
ENST00000539977	ENSG00000255642	PABPC1P4	filled
ENST00000417777	ENSG00000236736	UQCRC2P1	
ENST00000473404	ENSG00000240027	RP11-415I12.1	
ENST00000448027	ENSG00000226556	NPM1P49	
ENST00000509786	ENSG00000248288	CTD-2194F4.2	
ENST00000437728	ENSG00000237818	RPS3AP29	
ENST00000546669	ENSG00000257128	RP11-2A1.1	
ENST00000529891	ENSG00000255381	AP001258.5	
ENST00000433580	ENSG00000225712	ATP5G2P1	
ENST00000510278	ENSG00000251105	RP11-571I18.2	
ENST00000392009	ENSG00000213051	RPL5P5	
ENST00000421414	ENSG00000223490	CHCHD2P5	
ENST00000418052	ENSG00000233465	RPL7AP7	
ENST00000432257	ENSG00000228523	RP4-763G1.1	
ENST00000509878	ENSG00000251019	HIGD1AP13	
ENST00000432666	ENSG00000227000	HSPD1P14	
ENST00000545905	ENSG00000255976	RAB11AP2	
ENST00000499125	ENSG00000247911	HMGN1P12	
ENST00000470304	ENSG00000241130	RP11-507J18.1	
ENST00000612354	ENSG00000275827	RP11-1O2.2	
ENST00000411645	ENSG00000236942	RP11-6B6.3	
ENST00000394513	ENSG00000223905	RP11-335E14.1	
ENST00000617815	ENSG00000278744	CTA-444M12.3	
ENST00000443385	ENSG00000224947	VN1R25P	
ENST00000604465	ENSG00000271164	RP4-789D17.4	
ENST00000604500	ENSG00000271697	RP11-1012A1.10	
ENST00000454802	ENSG00000236004	RP4-800M22.4	
ENST00000456449	ENSG00000229179	SAR1P1	
ENST00000379591	ENSG00000205318	GCNT6	
ENST00000450119	ENSG00000220585	DDX18P6	filled
ENST00000423240	ENSG00000236060	HSPB1P1	
ENST00000433407	ENSG00000227950	RP11-312B8.2	
ENST00000415988	ENSG00000230018	RP11-481H12.1	
ENST00000554853	ENSG00000258906	CTD-2552B11.3	
ENST00000575091	ENSG00000262974	RP11-457I16.4	
ENST00000476029	ENSG00000241947	HNRNPA1P24	
ENST00000433335	ENSG00000238215	RP11-167P22.5	
ENST00000611756	ENSG00000277315	RP1-209B9.2	
ENST00000603871	ENSG00000271081	RP11-465K4.4	
ENST00000430903	ENSG00000228241	MOB1AP1	
ENST00000407851	ENSG00000219139	RP11-304C16.2	
ENST00000514311	ENSG00000248915	ACTR6P1	
ENST00000434488	ENSG00000231978	RP11-132G19.3	
ENST00000415214	ENSG00000223995	RPL32P35	
ENST00000437690	ENSG00000213964	CHCHD4P4	
ENST00000562497	ENSG00000261527	RP11-343C2.10	

ENST00000439659	ENSG00000227905	MED6P1	
ENST00000411508	ENSG00000229977	AC073264.10	
ENST00000475156	ENSG00000212994	RPS26P6	
ENST00000526466	ENSG00000213234	ST13P10	
ENST00000398541	ENSG00000214541	RPS4XP3	
ENST00000416471	ENSG00000241790	ENO1P4	
ENST00000455141	ENSG00000213421	RP11-84O12.2	
ENST00000519666	ENSG00000253861	SLC2A3P1	
ENST00000451120	ENSG00000234566	RPL7AP71	
ENST00000415815	ENSG00000228134	AC092578.1	
ENST00000442645	ENSG00000225816	GTF3AP5	
ENST00000514125	ENSG00000248209	RP11-18O11.1	
ENST00000492004	ENSG00000239256	RPL35AP35	
ENST00000604227	ENSG00000271222	RP11-1072C15.7	
ENST00000407590	ENSG00000218490	FCF1P10	
ENST00000570582	ENSG00000261866	AC092566.1	
ENST00000464146	ENSG00000241673	RPS27P12	
ENST00000604736	ENSG00000270677	RP11-294L11.1	
ENST00000418090	ENSG00000233934	RPL21P38	
ENST00000454451	ENSG00000238051	ISCUP1	
ENST00000511727	ENSG00000249950	CTC-394G3.1	
ENST00000427559	ENSG00000225496	AC104651.2	
ENST00000524621	ENSG00000255309	RP11-756D7.1	
ENST00000589286	ENSG00000235028	HMGN1P30	
ENST00000572223	ENSG00000262503	RP11-530N7.2	
ENST00000469206	ENSG00000227586	RP11-162A23.5	
ENST00000551090	ENSG00000258012	RP11-603K19.1	
ENST00000415292	ENSG00000229932	YWHAZP3	
ENST00000457574	ENSG00000229083	PSMA6P2	
ENST00000582221	ENSG00000264503	RP11-856M7.7	
ENST00000605428	ENSG00000271041	RP6-166C19.18	
ENST00000605291	ENSG00000271502	RP6-166C19.17	
ENST00000481845	ENSG00000240454	RPL39P26	
ENST00000447415	ENSG00000230721	RP4-612B15.2	
ENST00000604857	ENSG00000270762	FXYD6P1	
ENST00000465341	ENSG00000240991	RPL23AP67	
ENST00000429528	ENSG00000231879	TXNL1P1	
ENST00000469899	ENSG00000213343	RPL21P18	
ENST00000520205	ENSG00000253401	VTA1P2	
ENST00000431854	ENSG00000215085	RP11-471G13.2	
ENST00000434240	ENSG00000230638	RP11-486B10.4	
ENST00000550710	ENSG00000258104	HIGD1AP9	
ENST00000485426	ENSG00000240776	RP11-435F17.1	
ENST00000428027	ENSG00000234981	RP11-534L20.4	
ENST00000531576	ENSG00000255460	ZDHHC20P3	
ENST00000521763	ENSG00000253512	CTC-535M15.1	
ENST00000604421	ENSG00000270904	CTC-224D3.1	
ENST00000418085	ENSG00000226183	RANP7	
ENST00000455007	ENSG00000232503	CYCSP8	
ENST00000424739	ENSG00000235579	AC007283.4	
ENST00000452636	ENSG00000235013	COX20P2	
ENST00000488873	ENSG00000239212	RPL6P7	
ENST00000533382	ENSG00000255356	RP11-277E18.2	
ENST00000455326	ENSG00000227952	RPL18AP17	
ENST00000422522	ENSG00000226324	RP11-115M14.1	
ENST00000502332	ENSG00000206120	EGFEM1P	
ENST00000431685	ENSG00000206120	EGFEM1P	filled
ENST00000506760	ENSG00000206120	EGFEM1P	filled
ENST00000510136	ENSG00000206120	EGFEM1P	
ENST00000488647	ENSG00000206120	EGFEM1P	

ENST00000482245	ENSG00000206120	EGFEM1P	
ENST00000461151	ENSG00000206120	EGFEM1P	
ENST00000491953	ENSG00000206120	EGFEM1P	
ENST00000382864	ENSG00000206120	EGFEM1P	
ENST00000486295	ENSG00000206120	EGFEM1P	
ENST00000423971	ENSG00000234667	ACTBP13	filled
ENST00000466706	ENSG00000179073	TAAR3	
ENST00000481759	ENSG00000179073	TAAR3	
ENST00000525689	ENSG00000227160	THEM7P	filled
ENST00000436555	ENSG00000181201	HIST3H2BA	
ENST00000461341	ENSG00000240216	CPHL1P	filled
ENST00000446052	ENSG00000230086	VN1R96P	
ENST00000604015	ENSG00000242267	SKINTL	filled
ENST00000307165	ENSG00000230484	OR51A10P	
ENST00000418979	ENSG00000232528	RP4-673D20.3	
ENST00000458670	ENSG00000232528	RP4-673D20.3	
ENST00000493279	ENSG00000242111	TOPORSLP	
ENST00000431943	ENSG00000227742	CALR4P	
ENST00000499077	ENSG00000246082	NUDT16P1	filled
ENST00000498923	ENSG00000246082	NUDT16P1	filled
ENST00000400371	ENSG00000215452	ZNF663P	
ENST00000472805	ENSG00000215452	ZNF663P	
ENST00000612615	ENSG00000213471	TTLL13	
ENST00000417104	ENSG00000236083	OR13E1P	
ENST00000418074	ENSG00000230927	TMBIM7P	
ENST00000460880	ENSG00000172900	FLJ42102	
ENST00000415975	ENSG00000224709	OR11M1P	
ENST00000485590	ENSG00000123201	GUCY1B2	
ENST00000471276	ENSG00000182796	TMEM198B	
ENST00000487582	ENSG00000182796	TMEM198B	
ENST00000507007	ENSG00000248449	PCDHGB8P	filled
ENST00000417674	ENSG00000228211	HYALP1	
ENST00000446181	ENSG00000235661	MIR670HG	
ENST00000533531	ENSG00000235661	MIR670HG	
ENST00000476232	ENSG00000242193	RP11-568K15.1	
ENST00000462729	ENSG00000242193	RP11-568K15.1	
ENST00000512906	ENSG00000242193	RP11-568K15.1	
ENST00000474419	ENSG00000232871	SEC1P	
ENST00000420263	ENSG00000233228	LPCAT2BP	
ENST00000438496	ENSG00000214279	SCART1	
ENST00000615766	ENSG00000203799	CCDC162P	
ENST00000368966	ENSG00000203799	CCDC162P	filled
ENST00000508210	ENSG00000203799	CCDC162P	
ENST00000506861	ENSG00000203799	CCDC162P	filled
ENST00000424240	ENSG00000229186	ADAM1A	
ENST00000602903	ENSG00000229186	ADAM1A	
ENST00000556349	ENSG00000259074	PSMB7P1	
ENST00000487147	ENSG00000230524	COL6A4P1	
ENST00000508169	ENSG00000230524	COL6A4P1	
ENST00000399070	ENSG00000257267	ZNF271P	
ENST00000435735	ENSG00000233217	MROH3P	filled
ENST00000548527	ENSG00000214198	GNN	filled
ENST00000548897	ENSG00000214198	GNN	filled
ENST00000388789	ENSG00000214198	GNN	filled
ENST00000404786	ENSG00000218233	NEPNP	
ENST00000454683	ENSG00000226469	ADAM1B	
ENST00000486547	ENSG00000243995	RP1-300I2.2	
ENST00000619161	ENSG00000211451	GNRHR2	filled
ENST00000478379	ENSG00000240280	TCAM1P	
ENST00000467633	ENSG00000244301	AOX3P	

ENST00000437305	ENSG00000236409	NRADDP	
ENST00000602548	ENSG00000223591	CENPVP1	
ENST00000579400	ENSG00000265460	RP11-690G19.4	
ENST00000621658	ENSG00000179038	RP11-157K17.5	
ENST00000407767	ENSG00000220660	RP3-406A7.3	
ENST00000423039	ENSG00000269721	RPL23AP51	
ENST00000572284	ENSG00000220600	RP3-509I19.6	
ENST00000564938	ENSG00000261281	RP11-361M10.4	
ENST00000494591	ENSG00000243175	RP11-58H15.1	filled
ENST00000583091	ENSG00000266876	RP11-1148O4.1	filled
ENST00000493028	ENSG00000240531	RPL21P123	
ENST00000430569	ENSG00000224593	RP11-30B1.1	
ENST00000437804	ENSG00000232862	RP4-665N4.4	
ENST00000489875	ENSG00000180764	PIPSL	
ENST00000612052	ENSG00000244306	LINC01296	
ENST00000423837	ENSG00000235101	SETP9	
ENST00000618570	ENSG00000276380	UBE2NL	
ENST00000427198	ENSG00000224892	RPS4XP16	
ENST00000548011	ENSG00000257150	PGAM1P5	
ENST00000401830	ENSG00000223361	FTH1P10	
ENST00000492786	ENSG00000243742	RPLP0P2	filled
ENST00000426579	ENSG00000225727	RP11-71C5.2	filled
ENST00000545609	ENSG00000134297	PLEKHA8P1	
ENST00000553912	ENSG00000258505	RP11-90P16.1	
ENST00000554881	ENSG00000259019	RP11-305B6.2	
ENST00000424505	ENSG00000244433	PGBD4P7	
ENST00000610807	ENSG00000277783	RP1-232L24.1	
ENST00000339545	ENSG00000226999	AC073325.2	
ENST00000398779	ENSG00000214684	AC003045.1	filled
ENST00000453682	ENSG00000230691	MRPL35P4	
ENST00000430731	ENSG00000224806	ARL5AP4	
ENST00000604810	ENSG00000271492	MEMO1P5	
ENST00000477176	ENSG00000244740	RP11-463H24.1	
ENST00000576526	ENSG00000262000	CTD-2526A2.5	filled
ENST00000581715	ENSG00000264564	RP11-61D1.2	
ENST00000467090	ENSG00000242176	RPL39P31	
ENST00000417893	ENSG00000232337	AC009313.2	
ENST00000429574	ENSG00000213120	LIN28AP1	
ENST00000452524	ENSG00000229612	SUMO1P2	
ENST00000596405	ENSG00000268884	CTD-2550O8.7	
ENST00000547375	ENSG00000258142	RP11-18O15.1	
ENST00000547971	ENSG00000257287	RP11-98E6.1	
ENST00000437555	ENSG00000228783	RP11-147I11.1	
ENST00000432596	ENSG00000226299	RP6-29D12.4	
ENST00000604983	ENSG00000270397	RP6-166C19.16	
ENST00000604053	ENSG00000270196	RP11-550A18.1	
ENST00000427219	ENSG00000234354	RPS26P47	
ENST00000553000	ENSG00000257979	SNRPGP18	
ENST00000528866	ENSG00000254824	RP11-37O16.8	
ENST00000503825	ENSG00000248926	RP11-777B9.1	
ENST00000604279	ENSG00000271018	RP11-60E8.2	
ENST00000567995	ENSG00000259918	NDUFA5P11	
ENST00000489069	ENSG00000241261	RPL17P19	
ENST00000562293	ENSG00000260012	RP11-329J18.4	
ENST00000605160	ENSG00000271330	CTA-298G8.2	
ENST00000604488	ENSG00000271096	SUMO2P14	
ENST00000526024	ENSG00000254697	COPS8P3	
ENST00000405131	ENSG00000219993	RP11-288G3.3	
ENST00000511702	ENSG00000251464	RPL7L1P13	
ENST00000422457	ENSG00000236439	RP11-175B9.3	

ENST00000497954	ENSG00000240828	RPL21P4	
ENST00000603019	ENSG00000271214	RP11-9M16.3	
ENST00000437229	ENSG00000232664	LARP1BP3	
ENST00000445748	ENSG00000235240	AC026185.1	
ENST00000614375	ENSG00000273675	RP11-406H23.5	
ENST00000492447	ENSG00000239840	RPL23AP72	
ENST00000553864	ENSG00000258577	SNRPGP1	
ENST00000403786	ENSG00000219391	AC019129.1	filled
ENST00000526949	ENSG00000254771	RP11-50B3.1	
ENST00000472521	ENSG00000239483	RPS15AP16	
ENST00000480365	ENSG00000242992	FTH1P4	
ENST00000610385	ENSG00000275320	RP3-347M6.3	
ENST00000458353	ENSG00000234790	NUTF2P4	
ENST00000423835	ENSG00000227412	STK33P1	
ENST00000444546	ENSG00000224494	HNRNPA3P14	
ENST00000478134	ENSG00000240774	RP11-359H3.4	
ENST00000604022	ENSG00000270209	RP4-592A1.4	
ENST00000546955	ENSG00000258154	RP11-44N21.2	
ENST00000611633	ENSG00000276214	CTD-2146O16.1	
ENST00000518873	ENSG00000253639	SUMO2P18	
ENST00000475257	ENSG00000232174	CTB-164L20.1	
ENST00000604244	ENSG00000270218	RP11-325L12.5	
ENST00000585464	ENSG00000267692	TAF9P3	
ENST00000510378	ENSG00000251374	RPS23P5	
ENST00000446897	ENSG00000234335	RPS4XP11	
ENST00000581802	ENSG00000265273	PGDP1	
ENST00000507583	ENSG00000248977	RP11-395I6.1	
ENST00000402023	ENSG00000218459	RPS27P15	
ENST00000404250	ENSG00000220131	RP4-570O12.2	
ENST00000411911	ENSG00000225507	AC069282.6	
ENST00000549998	ENSG00000258206	RP13-619I2.2	
ENST00000603046	ENSG00000270258	DUXAP4	
ENST00000396055	ENSG00000213747	RP13-278H16.2	
ENST00000422983	ENSG00000236290	EEF1GP7	
ENST00000397288	ENSG00000214025	ATP5F1P4	
ENST00000441945	ENSG00000224004	ATP5C1P1	
ENST00000503440	ENSG00000248820	DYNLL1P6	
ENST00000443436	ENSG00000233307	AC109815.2	
ENST00000469026	ENSG00000213152	RPL7AP60	
ENST00000422812	ENSG00000232089	AC011233.2	
ENST00000440968	ENSG00000226188	HNRNPA1P3	
ENST00000481016	ENSG00000240522	RPL7AP10	
ENST00000449274	ENSG00000237620	GCNT1P5	
ENST00000471084	ENSG00000240568	RP11-1008M1.1	
ENST00000420392	ENSG00000237417	XRCC6P1	filled
ENST00000423857	ENSG00000226573	CTA-85E5.7	
ENST00000611327	ENSG00000273994	RP11-45O22.3	
ENST00000605126	ENSG00000270512	RP5-1136G2.1	
ENST00000414193	ENSG00000234557	RP1-164F3.8	
ENST00000323496	ENSG00000223551	TMSB4XP4	
ENST00000417179	ENSG00000229858	AC016831.5	
ENST00000507315	ENSG00000249050	RP11-375N15.1	
ENST00000598302	ENSG00000269742	BNIP3P29	
ENST00000521432	ENSG00000213985	AC078899.1	
ENST00000606668	ENSG00000272006	RP11-583F2.6	
ENST00000603189	ENSG00000270192	MRPS35P3	
ENST00000493631	ENSG00000229048	DUTP1	
ENST00000511857	ENSG00000250403	RP11-15B17.2	
ENST00000447000	ENSG00000225118	RP11-477L16.2	
ENST00000394301	ENSG00000237806	FAUP2	

ENST00000443733	ENSG00000226338	AC079150.2	
ENST00000566722	ENSG00000261614	YBX3P1	
ENST00000407837	ENSG00000219532	RP3-323K23.3	
ENST00000484315	ENSG00000214759	CTD-2509G16.1	
ENST00000438508	ENSG00000224070	HMGN1P6	
ENST00000416102	ENSG00000238180	AC017079.4	
ENST00000458157	ENSG00000229593	SUCLA2P3	
ENST00000521318	ENSG00000254255	CTD-2008O4.1	
ENST00000553226	ENSG00000258284	POLR2KP1	
ENST00000437724	ENSG00000237551	AC096775.2	
ENST00000547280	ENSG00000257852	RP11-843B15.1	
ENST00000604322	ENSG00000270588	RP11-810F22.1	
ENST00000588959	ENSG00000267652	RP11-188I24.1	
ENST00000444288	ENSG00000227692	MED28P3	
ENST00000398690	ENSG00000214628	RP11-392M9.2	
ENST00000429993	ENSG00000226473	AC079807.1	
ENST00000416706	ENSG00000227004	AC108032.1	
ENST00000470806	ENSG00000243538	CTB-55B8.1	
ENST00000619891	ENSG00000277143	RP11-113A11.3	
ENST00000488305	ENSG00000242837	RPL21P13	
ENST00000562403	ENSG00000260010	ZNF720P1	
ENST00000413892	ENSG00000229087	RP11-32P22.1	
ENST00000467688	ENSG00000243592	RPL17P22	
ENST00000440222	ENSG00000229398	RP4-620E11.5	
ENST00000605745	ENSG00000270378	RP11-108K3.5	
ENST00000407291	ENSG00000220447	RP1-276J11.2	
ENST00000521355	ENSG00000253137	RP11-509P12.1	
ENST00000469688	ENSG00000243404	RPL35AP32	
ENST00000598115	ENSG00000268144	NIFKP6	filled
ENST00000449851	ENSG00000232994	RPL7P14	
ENST00000614561	ENSG00000275550	RP4-744I24.4	
ENST00000413752	ENSG00000229554	RPL21P24	
ENST00000448956	ENSG00000236571	RP6-29D12.3	
ENST00000510152	ENSG00000250568	RP11-333E13.2	filled
ENST00000507982	ENSG00000250473	DUTP7	
ENST00000454989	ENSG00000234413	RP6-29D12.2	
ENST00000514909	ENSG00000248950	LDHBP3	
ENST00000412010	ENSG00000230284	RP11-402G3.4	
ENST00000581726	ENSG00000266648	SETP3	
ENST00000605001	ENSG00000270437	RP11-511I11.2	
ENST00000414323	ENSG00000235299	MRPL53P1	
ENST00000612950	ENSG00000274919	RP11-428F18.2	
ENST00000604298	ENSG00000270241	RP4-657M3.2	
ENST00000393490	ENSG00000204652	RPS26P8	
ENST00000604757	ENSG00000270385	GS1-21A4.2	
ENST00000622839	ENSG00000276703	RP1-188K17.2	
ENST00000519475	ENSG00000253579	SUMO2P16	
ENST00000577786	ENSG00000264971	PRR13P4	
ENST00000554037	ENSG00000259121	RP11-545M17.2	
ENST00000551552	ENSG00000257658	RP3-521E19.3	
ENST00000484182	ENSG00000243314	RP11-538P18.1	
ENST00000571144	ENSG00000261970	RP11-342L8.2	
ENST00000505795	ENSG00000249619	HMGN1P13	
ENST00000457046	ENSG00000225094	SETP20	
ENST00000497294	ENSG00000242634	RPS24P16	
ENST00000558606	ENSG00000259413	RP11-5O23.1	
ENST00000556558	ENSG00000259165	DDX18P1	
ENST00000395181	ENSG00000213601	KRT18P19	
ENST00000414612	ENSG00000231602	RPL35AP4	
ENST00000555505	ENSG00000258578	RP11-98L12.2	

ENST00000485829	ENSG00000242727	RP11-123J14.1	
ENST00000487608	ENSG00000239413	RPS27P23	
ENST00000448300	ENSG00000237319	RP1-136J15.1	
ENST00000417617	ENSG00000213798	AC004129.9	
ENST00000429083	ENSG00000230482	ATP5G2P3	
ENST00000527291	ENSG00000255010	RP11-113K21.3	
ENST00000579765	ENSG00000264150	RP11-715F3.1	
ENST00000469001	ENSG00000243053	RPL31P58	
ENST00000605336	ENSG00000270306	RP3-437C15.2	
ENST00000603536	ENSG00000271142	YWHAQP7	
ENST00000438138	ENSG00000230469	RPL5P26	
ENST00000403720	ENSG00000227541	RP3-525N14.2	
ENST00000429708	ENSG00000237757	EEF1A1P30	
ENST00000447495	ENSG00000231849	UBE2V2P4	
ENST00000435230	ENSG00000237163	RP4-771M4.2	
ENST00000484753	ENSG00000239323	CTD-2213F21.1	
ENST00000478876	ENSG00000241045	RPL7P43	
ENST00000548926	ENSG00000258090	RP11-734E19.1	
ENST00000416077	ENSG00000232702	RP3-437C15.1	
ENST00000476607	ENSG00000243483	RP11-572M11.2	
ENST00000394996	ENSG00000213574	LDHAP5	
ENST00000456494	ENSG00000229322	RP5-937E21.1	
ENST00000514799	ENSG00000214244	SETP21	
ENST00000514578	ENSG00000249721	RP11-83M16.4	
ENST00000458108	ENSG00000234737	KRT18P15	
ENST00000407180	ENSG00000219703	RAP1BP3	
ENST00000498718	ENSG00000239924	RPL29P22	
ENST00000508612	ENSG00000251656	CTD-2201E18.4	
ENST00000434149	ENSG00000232778	RPL23AP50	
ENST00000506347	ENSG00000231707	PABPC1P1	filled
ENST00000412942	ENSG00000232687	RPL12P9	
ENST00000473394	ENSG00000243303	RP11-268I9.1	
ENST00000603429	ENSG00000270335	RP11-642D6.1	
ENST00000431135	ENSG00000237360	CHCHD4P2	
ENST00000442640	ENSG00000213669	RP11-476B13.2	
ENST00000450718	ENSG00000227922	SPTLC1P5	
ENST00000604718	ENSG00000271346	RP1-321E8.4	
ENST00000605040	ENSG00000270456	RP6-166C19.14	
ENST00000403408	ENSG00000219940	SPTLC1P3	
ENST00000450381	ENSG00000235308	RP11-82K18.2	
ENST00000431191	ENSG00000227529	SAR1P1	
ENST00000587313	ENSG00000267336	RP11-773H22.2	
ENST00000438615	ENSG00000235190	RP1-197O17.3	
ENST00000603687	ENSG00000271500	RP11-288K12.1	
ENST00000411782	ENSG00000228780	RP1-50A13.1	
ENST00000425607	ENSG00000235382	MTND4P31	filled
ENST00000505442	ENSG00000248577	RP11-122C5.2	
ENST00000452821	ENSG00000213609	RP11-170M17.2	
ENST00000414613	ENSG00000237522	NONOP2	
ENST00000520293	ENSG00000253237	RP11-775E10.1	
ENST00000621124	ENSG00000274867	RP4-724P12.2	
ENST00000602729	ENSG00000228948	SLC25A6P5	
ENST00000603184	ENSG00000271118	RP11-175E9.2	
ENST00000445859	ENSG00000230980	RPL36AP39	
ENST00000441256	ENSG00000230879	RBMX2P4	
ENST00000434751	ENSG00000236062	GSTM5P1	
ENST00000451357	ENSG00000243085	RP11-392E22.3	
ENST00000453086	ENSG00000229324	NUTF2P7	
ENST00000547726	ENSG00000257851	HNRNPA3P10	
ENST00000418670	ENSG00000234187	AIMP1P1	

ENST00000398059	ENSG00000228209	AC073091.2	
ENST00000510257	ENSG00000250922	ATP5EP1	
ENST00000419932	ENSG00000226958	RNA28S5	
ENST00000494303	ENSG00000240674	RP11-366M4.1	
ENST00000494902	ENSG00000241917	RP11-512N21.1	
ENST00000588924	ENSG00000267203	SNRPGP4	
ENST00000551462	ENSG00000258288	NENFP2	
ENST00000424793	ENSG00000227395	EIF2AP4	
ENST00000398297	ENSG00000215120	AL590763.5	filled
ENST00000547512	ENSG00000257246	RP11-416A17.6	
ENST00000406473	ENSG00000218089	DNAJA1P4	
ENST00000393907	ENSG00000213403	CISD1P1	
ENST00000469695	ENSG00000242088	AC090602.2	
ENST00000604752	ENSG00000270251	RP6-166C19.13	
ENST00000531501	ENSG00000255259	ZNF123P	
ENST00000395709	ENSG00000213698	RP1-172N19.4	
ENST00000466725	ENSG00000243494	CTC-550B14.1	
ENST00000404926	ENSG00000220506	RP3-415N12.1	
ENST00000622571	ENSG00000274493	MRPS17P5	
ENST00000559810	ENSG00000259535	RPL21P12	filled
ENST00000416921	ENSG00000227361	RPS24P7	filled
ENST00000423267	ENSG00000204399	AC012306.2	
ENST00000617434	ENSG00000275488	RP11-793H13.12	
ENST00000423444	ENSG00000229878	RP11-479G22.7	
ENST00000604731	ENSG00000271159	GS1-200K5.1	
ENST00000457984	ENSG00000229827	AC093899.3	
ENST00000436365	ENSG00000224656	RP1-212G6.4	
ENST00000441973	ENSG00000224188	UBE2D3P4	
ENST00000434943	ENSG00000232728	PHB2P1	
ENST00000455606	ENSG00000237242	BTF3P15	
ENST00000434567	ENSG00000232163	RPLP1P13	
ENST00000417974	ENSG00000237916	RP11-537E18.1	
ENST00000492168	ENSG00000239465	RP11-330L19.2	
ENST00000483963	ENSG00000243403	RP11-330L19.1	
ENST00000433994	ENSG00000236655	AC023347.2	
ENST00000519073	ENSG00000253109	RP11-619L12.1	
ENST00000506854	ENSG00000249312	ARL2BPP4	
ENST00000441891	ENSG00000234061	AC007969.4	
ENST00000493817	ENSG00000242276	RPL5P3	
ENST00000435859	ENSG00000235334	RP1-203C2.3	
ENST00000558741	ENSG00000259622	RP11-597K23.1	
ENST00000604328	ENSG00000270826	RP1-60G11.1	
ENST00000603753	ENSG00000270678	RP6-166C19.21	
ENST00000463604	ENSG00000242807	RPL26P31	
ENST00000425889	ENSG00000236930	RP11-852E15.1	
ENST00000491091	ENSG00000240236	HNRNPA1P23	
ENST00000605180	ENSG00000270521	RP11-24C14.1	
ENST00000619218	ENSG00000275940	MRP63P3	
ENST00000437739	ENSG00000218632	RPL7P28	
ENST00000441999	ENSG00000233908	RP11-425D10.1	
ENST00000418220	ENSG00000224129	DPPA2P2	
ENST00000452670	ENSG00000233158	RPS24P6	
ENST00000605372	ENSG00000271146	RP11-479F13.1	
ENST00000466859	ENSG00000244295	RPS20P21	
ENST00000493002	ENSG00000213527	RP11-591L14.1	
ENST00000414476	ENSG00000234192	RP11-57C13.5	
ENST00000519036	ENSG00000253670	RP11-429O2.1	
ENST00000455722	ENSG00000237717	RP11-402P6.4	
ENST00000427639	ENSG00000227663	RPL7P2	
ENST00000503502	ENSG00000249302	FTH1P24	

ENST00000511094	ENSG00000249418	AC005741.2	
ENST00000529418	ENSG00000254890	RP11-10A7.1	
ENST00000395517	ENSG00000225419	RPL21P3	
ENST00000455621	ENSG00000213058	RP4-765C7.2	
ENST00000437729	ENSG00000224263	CYCSP12	
ENST00000473913	ENSG00000243697	RP11-463O9.1	
ENST00000602999	ENSG00000271532	RP11-426K3.1	
ENST00000457882	ENSG00000230161	RP11-62L10.1	
ENST00000433054	ENSG00000231735	RP11-1O7.1	
ENST00000454743	ENSG00000227536	SOCS5P4	
ENST00000454064	ENSG00000226718	SNRPGP8	
ENST00000452903	ENSG00000231802	AC009502.1	
ENST00000467018	ENSG00000241464	RPL39P38	
ENST00000448883	ENSG00000224219	AC074290.1	
ENST00000488538	ENSG00000214988	RPL7AP26	
ENST00000440996	ENSG00000232050	CTA-125H2.3	
ENST00000551532	ENSG00000257668	RP11-58A17.2	
ENST00000495392	ENSG00000227081	RP11-543P15.1	
ENST00000445825	ENSG00000233910	GTF2F2P2	
ENST00000418010	ENSG00000232622	RP11-484D4.3	
ENST00000484463	ENSG00000244422	RPL38P3	
ENST00000463867	ENSG00000242307	RPS26P52	
ENST00000436102	ENSG00000224092	RP11-443B9.1	
ENST00000441288	ENSG00000236327	RP11-759F5.1	
ENST00000451203	ENSG00000225416	AC104843.3	
ENST00000618587	ENSG00000278102	RP11-14J16.1	
ENST00000404856	ENSG00000217414	RP11-528A10.2	filled
ENST00000471495	ENSG00000242036	RP11-603B13.1	
ENST00000423288	ENSG00000238040	SALL4P2	
ENST00000513990	ENSG00000251342	CTC-235G5.2	
ENST00000409159	ENSG00000233111	RAB1C	
ENST00000422268	ENSG00000224052	HTATSF1P	
ENST00000558669	ENSG00000259440	NPM1P43	
ENST00000415693	ENSG00000233373	AC106874.3	
ENST00000506459	ENSG00000250076	RP11-141P6.1	
ENST00000406229	ENSG00000219592	NCSTNP1	
ENST00000492041	ENSG00000241499	RP11-85G20.2	
ENST00000605279	ENSG00000270493	RP6-166C19.22	
ENST00000404247	ENSG00000218870	SLC25A6P6	
ENST00000420569	ENSG00000229948	B3GALNT1P1	
ENST00000583290	ENSG00000265939	UBE2CP2	
ENST00000400616	ENSG00000215586	RP5-836E8.1	
ENST00000463581	ENSG00000241526	RP11-144C9.1	
ENST00000440214	ENSG00000229831	AC108039.3	
ENST00000403956	ENSG00000216519	RP3-406A7.1	
ENST00000493565	ENSG00000240201	RP11-269C4.1	
ENST00000568623	ENSG00000260414	RP11-1437A8.2	
ENST00000603389	ENSG00000271315	WI2-2221J1.1	
ENST00000453429	ENSG00000213880	RPL7AP7	
ENST00000519957	ENSG00000253393	RANP9	
ENST00000532310	ENSG00000255454	RP11-46P12.1	
ENST00000502867	ENSG00000213608	SLC25A14P1	
ENST00000507985	ENSG00000248611	RP11-158C21.3	
ENST00000454070	ENSG00000173810	PPIAP7	
ENST00000505749	ENSG00000248955	RP11-114H7.3	
ENST00000588843	ENSG00000267800	RP11-49K24.5	
ENST00000616254	ENSG00000277795	AC005752.10	
ENST00000590286	ENSG00000267799	MAN1A2P1	
ENST00000407776	ENSG00000217680	RP11-415D17.1	
ENST00000437188	ENSG00000214484	AC074008.1	

ENST00000567176	ENSG00000260344	RP11-56L13.6	
ENST00000418216	ENSG00000225583	RP4-630C24.1	
ENST00000523033	ENSG00000253612	WBP1LP3	
ENST00000452808	ENSG00000229635	RP4-713B5.2	
ENST00000431607	ENSG00000230734	RPL10P3	
ENST00000566875	ENSG00000260602	HMGN2P40	
ENST00000440115	ENSG00000225310	DNAJC19P4	
ENST00000411430	ENSG00000229733	RP5-1189B24.1	
ENST00000589357	ENSG00000267319	CTD-2528L19.3	
ENST00000404414	ENSG00000220311	RPL35AP18	
ENST00000611630	ENSG00000276412	RP11-157L3.9	
ENST00000434965	ENSG00000229016	RP11-224O19.4	
ENST00000603591	ENSG00000271350	CTD-2384B9.1	
ENST00000614861	ENSG00000277213	RP11-286O1.2	
ENST00000621938	ENSG00000275381	RP11-401N16.2	
ENST00000406203	ENSG00000219736	RP11-560O20.1	
ENST00000605661	ENSG00000270702	RP11-107F6.4	
ENST00000604330	ENSG00000271154	RP6-166C19.23	
ENST00000504014	ENSG00000248271	PGAM1P1	
ENST00000416730	ENSG00000232168	RP11-180D15.1	
ENST00000546187	ENSG00000256346	RP11-72J9.1	
ENST00000544546	ENSG00000227760	RP11-298E10.1	
ENST00000427282	ENSG00000223505	RP11-397P13.7	
ENST00000605565	ENSG00000271298	RP5-1012F16.2	
ENST00000419887	ENSG00000231254	PCED1CP	
ENST00000535821	ENSG00000255763	MRPS18CP4	
ENST00000527568	ENSG00000255463	RP11-16F15.4	
ENST00000417973	ENSG00000232901	CYCSP10	
ENST00000619538	ENSG00000275103	SCb-64M4.1	filled
ENST00000436521	ENSG00000227154	MKRNP2	
ENST00000604816	ENSG00000270620	RP1-134O19.3	
ENST00000618727	ENSG00000277171	RP11-191H23.1	
ENST00000503430	ENSG00000250979	RP11-656G20.1	filled
ENST00000616451	ENSG00000277876	RP11-467P22.5	
ENST00000616055	ENSG00000274601	WI2-88277B6.1	
ENST00000612939	ENSG00000275768	RP11-11P22.1	
ENST00000439100	ENSG00000225567	OR2AD1P	
ENST00000402365	ENSG00000220721	OR1F12	
ENST00000416478	ENSG00000228304	OR4K6P	
ENST00000619934	ENSG00000274666	CTD-2566J3.2	
ENST00000618630	ENSG00000276362	RP11-241M13.2	
ENST00000610750	ENSG00000275314	RP11-32A1.2	
ENST00000405929	ENSG00000219392	RP1-265C24.5	
ENST00000553271	ENSG00000258816	RP11-74M13.3	
ENST00000590718	ENSG00000267482	RNY4P13	
ENST00000620602	ENSG00000277984	RP11-735A19.3	
ENST00000433767	ENSG00000225491	UBE2Q2P4Y	
ENST00000526939	ENSG00000255077	OR4X7P	
ENST00000514684	ENSG00000248642	OR10J2P	
ENST00000611491	ENSG00000278683	RP11-132A1.6	
ENST00000527724	ENSG00000255514	OR4B2P	
ENST00000568600	ENSG00000198555	RP11-598D12.4	filled
ENST00000511039	ENSG00000250207	AC079776.6	
ENST00000424402	ENSG00000237860	RP11-232D9.4	
ENST00000412173	ENSG00000236205	RP3-406C18.1	
ENST00000452394	ENSG00000233563	OR52E7P	
ENST00000612320	ENSG00000274642	CH17-472G23.1	
ENST00000521465	ENSG00000254608	RP11-56A10.1	filled
ENST00000554039	ENSG00000258625	OR11H5P	
ENST00000533878	ENSG00000254832	OR4A40P	

ENST00000441183	ENSG00000230546	RP11-393I23.3	
ENST00000588632	ENSG00000267310	OR4G1P	
ENST00000532318	ENSG00000255401	RP11-15D14.2	
ENST00000403446	ENSG00000218274	RP3-407E4.3	
ENST00000423388	ENSG00000197866	OR5M12P	
ENST00000402490	ENSG00000217004	RP3-329A5.1	
ENST00000425337	ENSG00000237334	HLA-Z	
ENST00000618994	ENSG00000273704	RP11-573C10.1	
ENST00000616145	ENSG00000274923	GS1-214D18.3	
ENST00000313342	ENSG00000215861	WI2-1896O14.1	
ENST00000413867	ENSG00000223636	UBE2Q2P5Y	
ENST00000445188	ENSG00000233053	HLA-Z	
ENST00000503365	ENSG00000251590	NIFKP7	filled
ENST00000453277	ENSG00000232398	TMPRSS11CP	
ENST00000504659	ENSG00000251686	OR10J8P	
ENST00000553483	ENSG00000258597	SERPINA2	
ENST00000618654	ENSG00000276205	RP11-529O20.2	
ENST00000427293	ENSG00000223447	HLA-DPA3	
ENST00000534196	ENSG00000254770	OR4D7P	
ENST00000424920	ENSG00000231895	HLA-DPA3	
ENST00000595155	ENSG00000213753	CENPBD1P1	
ENST00000580773	ENSG00000215486	ARL2BPP3	
ENST00000421226	ENSG00000215486	ARL2BPP3	
ENST00000515019	ENSG00000248924	RP11-481K16.2	
ENST00000512366	ENSG00000248924	RP11-481K16.2	filled
ENST00000464216	ENSG00000241431	RPL37P6	
ENST00000580031	ENSG00000265713	RP11-82O19.2	
ENST00000531034	ENSG00000254727	PTP4A1P6	
ENST00000503807	ENSG00000226306	NPY6R	
ENST00000494130	ENSG00000240443	RPS10P20	
ENST00000458482	ENSG00000239995	TPT1P11	
ENST00000552582	ENSG00000257501	RP11-1016B18.1	
ENST00000550221	ENSG00000257501	RP11-1016B18.1	
ENST00000552615	ENSG00000257501	RP11-1016B18.1	
ENST00000563752	ENSG00000236253	SLC25A3P1	
ENST00000566100	ENSG00000236253	SLC25A3P1	
ENST00000569142	ENSG00000236253	SLC25A3P1	
ENST00000595299	ENSG00000249065	PCNAP1	
ENST00000505363	ENSG00000249065	PCNAP1	
ENST00000392735	ENSG00000241129	RPL22P19	
ENST00000412546	ENSG00000231478	FCF1P9	
ENST00000562267	ENSG00000261127	RP11-17M15.2	
ENST00000437960	ENSG00000241735	FABP5P3	
ENST00000610068	ENSG00000229036	VDAC1P8	
ENST00000438118	ENSG00000229036	VDAC1P8	
ENST00000619849	ENSG00000229036	VDAC1P8	
ENST00000415586	ENSG00000229036	VDAC1P8	
ENST00000591189	ENSG00000229036	VDAC1P8	
ENST00000593045	ENSG00000229036	VDAC1P8	
ENST00000590703	ENSG00000229036	VDAC1P8	
ENST00000593175	ENSG00000229036	VDAC1P8	
ENST00000612298	ENSG00000229036	VDAC1P8	
ENST00000611810	ENSG00000229036	VDAC1P8	
ENST00000589489	ENSG00000229036	VDAC1P8	
ENST00000616580	ENSG00000275026	RP11-15E1.1	
ENST00000514718	ENSG00000249149	RP11-79P5.3	
ENST00000562726	ENSG00000224578	HNRNPA1P48	
ENST00000447646	ENSG00000223428	RP11-399K21.5	
ENST00000443827	ENSG00000242412	DBIL5P2	
ENST00000393073	ENSG00000214182	PTMAP5	

ENST00000409454	ENSG00000179362	HMGN2P46	filled
ENST00000396644	ENSG00000179362	HMGN2P46	filled
ENST00000619210	ENSG00000225210	DUXAP10	
ENST00000622815	ENSG00000225210	DUXAP10	
ENST00000456786	ENSG00000271672	DUXAP8	
ENST00000578523	ENSG00000264529	RP11-556O9.3	
ENST00000414782	ENSG00000231322	RPL13AP17	
ENST00000574864	ENSG00000213109	RP11-55K22.2	
ENST00000485827	ENSG00000196656	AC004057.1	
ENST00000427230	ENSG00000237065	NANOGP4	
ENST00000527374	ENSG00000254506	RP11-748H22.1	
ENST00000565928	ENSG00000235217	TSPY26P	filled
ENST00000476365	ENSG00000235217	TSPY26P	filled
ENST00000608660	ENSG00000226937	CEP164P1	
ENST00000447996	ENSG00000215875	ST13P20	
ENST00000557014	ENSG00000258966	GTF3AP2	
ENST00000569208	ENSG00000260258	RP11-467J12.2	
ENST00000602826	ENSG00000260258	RP11-467J12.2	
ENST00000600311	ENSG00000258856	CTD-2325K12.1	
ENST00000602631	ENSG00000229052	RP11-386I23.1	
ENST00000418249	ENSG00000229052	RP11-386I23.1	
ENST00000498671	ENSG00000240480	RPL29P2	
ENST00000552735	ENSG00000257512	RP11-486A14.1	
ENST00000393388	ENSG00000242444	RP11-320N7.1	
ENST00000513994	ENSG00000251429	RP11-597D13.7	
ENST00000591758	ENSG00000224631	RP11-51O6.1	
ENST00000406795	ENSG00000151963	RP11-775A3.1	
ENST00000392760	ENSG00000213182	OR10D5P	
ENST00000560644	ENSG00000259633	UBE2Q2P8	
ENST00000431706	ENSG00000214992	AKAP17BP	
ENST00000434556	ENSG00000232845	TRAPPC2P9	
ENST00000616045	ENSG00000277708	PGBD4P2	
ENST00000615543	ENSG00000274691	RP11-310E22.6	
ENST00000619632	ENSG00000278496	RP11-144H23.2	
ENST00000436962	ENSG00000234842	AC007163.8	
ENST00000458618	ENSG00000228785	HLA-Z	
ENST00000454657	ENSG00000227626	OR2AD1P	
ENST00000579654	ENSG00000263870	CTD-2350C19.5	
ENST00000440621	ENSG00000235432	RP5-930J4.5	
ENST00000612448	ENSG00000277608	RP11-321L2.3	
ENST00000447093	ENSG00000234522	RP11-572P18.2	
ENST00000617638	ENSG00000274572	CTD-2025J6.1	
ENST00000492842	ENSG00000240361	OR4G11P	
ENST00000438437	ENSG00000225459	HLA-Z	
ENST00000546924	ENSG00000257994	RP11-626I20.2	
ENST00000418459	ENSG00000232799	CRYGFP	
ENST00000417020	ENSG00000234099	MTND4P11	
ENST00000433774	ENSG00000235794	RP5-837O21.6	
ENST00000430791	ENSG00000232752	RP5-837O21.5	
ENST00000438531	ENSG00000229842	RP5-837O21.4	
ENST00000506803	ENSG00000249081	OR5M14P	
ENST00000419780	ENSG00000236689	RP5-837O21.3	
ENST00000434543	ENSG00000224299	AC007282.6	
ENST00000436045	ENSG00000230231	FMO7P	filled
ENST00000553311	ENSG00000258438	OR11K2P	
ENST00000419366	ENSG00000227841	MTND5P31	
ENST00000452205	ENSG00000224590	AC007163.10	
ENST00000554847	ENSG00000258564	OR4N1P	
ENST00000612874	ENSG00000277000	DUSP12P1	
ENST00000610667	ENSG00000275015	RP11-129G17.2	

ENST00000557871	ENSG00000259556	RP11-56B16.2	
ENST00000613287	ENSG00000276538	RP11-545G3.2	
ENST00000506013	ENSG00000251508	RP5-862P8.3	
ENST00000531671	ENSG00000255019	OR5D15P	
ENST00000432847	ENSG00000237871	HLA-Z	
ENST00000566879	ENSG00000261835	RP11-429P3.2	
ENST00000432003	ENSG00000228498	HLA-Z	
ENST00000554269	ENSG00000258780	BMS1P15	filled
ENST00000428250	ENSG00000228827	RP11-234P3.2	
ENST00000518369	ENSG00000253630	CTC-370J7.1	
ENST00000580232	ENSG00000265340	OR4K7P	
ENST00000414479	ENSG00000229568	AC010146.1	
ENST00000587562	ENSG00000267771	CTC-260E6.7	
ENST00000419107	ENSG00000227556	RP11-472F19.1	
ENST00000427165	ENSG00000225418	AKR1C5P	filled
ENST00000549699	ENSG00000258273	RP11-370I10.4	
ENST00000506703	ENSG00000248978	RP11-98H4.1	
ENST00000291129	ENSG00000159860	FAM115D	
ENST00000611221	ENSG00000276828	RP11-88L20.1	
ENST00000416634	ENSG00000226960	RP5-837O21.1	
ENST00000550410	ENSG00000257408	RP11-645C24.4	
ENST00000529831	ENSG00000254613	OR6M2P	
ENST00000502880	ENSG00000235991	RP11-143H17.1	
ENST00000611632	ENSG00000278193	RP11-313C15.2	
ENST00000393988	ENSG00000225438	KRT41P	
ENST00000609258	ENSG00000273234	OR2A13P	
ENST00000431701	ENSG00000233135	RPS27P18	
ENST00000482585	ENSG00000241026	CTD-2036J7.1	
ENST00000612091	ENSG00000276928	RP11-483E23.9	
ENST00000619761	ENSG00000274532	RP11-483E23.10	
ENST00000556991	ENSG00000258541	OR4K4P	
ENST00000485242	ENSG00000242214	MTND3P6	
ENST00000613847	ENSG00000277500	RP11-456J20.2	
ENST00000611186	ENSG00000278110	XXbac-B33L19.10	
ENST00000355343	ENSG00000196403	OR10D1P	filled
ENST00000289731	ENSG00000158731	OR10J6P	
ENST00000620045	ENSG00000277056	RP11-65E10.1	
ENST00000605130	ENSG00000270975	RP11-723P16.3	
ENST00000412857	ENSG00000223603	CRPP1	
ENST00000519383	ENSG00000253273	RP11-99A14.1	
ENST00000529003	ENSG00000254752	OR5M2P	
ENST00000525935	ENSG00000254475	OR2AT1P	filled
ENST00000511351	ENSG00000249486	RP11-215I16.1	
ENST00000612314	ENSG00000278283	RP5-884M20.4	
ENST00000427283	ENSG00000231827	RP11-216N14.5	
ENST00000525712	ENSG00000254743	OR10V3P	
ENST00000528485	ENSG00000254403	OR10Y1P	
ENST00000452651	ENSG00000243831	RP1-81D8.4	
ENST00000617915	ENSG00000273933	RP11-48O20.5	
ENST00000622094	ENSG00000276940	GS1-383H3.7	
ENST00000448848	ENSG00000231586	RPS23P9	
ENST00000622239	ENSG00000275614	RP11-12A20.12	
ENST00000555180	ENSG00000258467	PHKBP2	
ENST00000617319	ENSG00000275318	RP11-526D8.11	
ENST00000446352	ENSG00000231195	IFNA11P	
ENST00000443734	ENSG00000237043	OR2AD1P	
ENST00000507585	ENSG00000250762	RP1-313L4.4	
ENST00000419907	ENSG00000233650	OR2AD1P	
ENST00000547875	ENSG00000257757	OR6C7P	
ENST00000439459	ENSG00000232781	OR2AD1P	

ENST00000622303	ENSG00000277883	RP6-191P20.5	filled
ENST00000611760	ENSG00000277555	CTA-38K21.5	
ENST00000443754	ENSG00000237238	BMS1P10	filled
ENST00000533932	ENSG00000255030	OR8B5P	
ENST00000615015	ENSG00000278244	CTA-38K21.3	
ENST00000619131	ENSG00000278504	CTA-38K21.4	
ENST00000575506	ENSG00000261963	RP11-74E22.4	
ENST00000401898	ENSG00000217707	SERPINB8P1	
ENST00000485382	ENSG00000204790	CBWD6	
ENST00000419756	ENSG00000229386	OR8B9P	
ENST00000529692	ENSG00000254646	OR8B10P	
ENST00000528904	ENSG00000255315	OR8A3P	
ENST00000618332	ENSG00000277549	RP11-359P14.3	
ENST00000603616	ENSG00000270552	DUXAP3	
ENST00000619723	ENSG00000275315	RP11-143N13.4	
ENST00000413827	ENSG00000232261	RP4-560B9.5	
ENST00000426849	ENSG00000227616	AC063976.1	
ENST00000413596	ENSG00000227023	OR51A3P	
ENST00000522108	ENSG00000253179	CALCP	filled
ENST00000434920	ENSG00000235301	HLA-Z	
ENST00000610003	ENSG00000272559	OR51F3P	
ENST00000583145	ENSG00000265298	RP13-104F24.3	
ENST00000572320	ENSG00000262299	RP11-216N14.5	
ENST00000446214	ENSG00000237171	GS1-466O4.5	
ENST00000608682	ENSG00000272569	OR5BL1P	
ENST00000611406	ENSG00000277479	AL022345.10	
ENST00000606307	ENSG00000272387	AL022345.8	
ENST00000413739	ENSG00000228940	RP11-348A7.1	
ENST00000603274	ENSG00000271581	XXbac-BPG248L24	filled
ENST00000441814	ENSG00000224366	AC138472.4	
ENST00000316512	ENSG00000273762	VN1R76P	
ENST00000556075	ENSG00000258684	BMS1P16	filled
ENST00000424327	ENSG00000203758	OR10T1P	
ENST00000328113	ENSG00000183909	OR4G2P	
ENST00000425977	ENSG00000225538	OR5BE1P	
ENST00000612768	ENSG00000275675	RP11-1021O19.2	
ENST00000509872	ENSG00000250599	AC064853.3	
ENST00000616908	ENSG00000278319	RP11-266I3.8	
ENST00000439389	ENSG00000227493	RP11-104D21.1	
ENST00000455649	ENSG00000232068	AF003529.2	
ENST00000427395	ENSG00000231547	RP11-38O14.6	
ENST00000569468	ENSG00000260165	CTD-2323K18.3	
ENST00000524992	ENSG00000255386	OR5BR1P	
ENST00000611813	ENSG00000276099	LILRP1	
ENST00000436125	ENSG00000225048	OR2AD1P	
ENST00000563970	ENSG00000261365	RP11-483E23.5	
ENST00000611461	ENSG00000274814	RP11-536P16.5	
ENST00000572799	ENSG00000262040	AC003958.1	
ENST00000448910	ENSG00000226691	HLA-Z	
ENST00000414737	ENSG00000236070	OR2AD1P	
ENST00000551839	ENSG00000257392	RP11-554D14.5	
ENST00000621416	ENSG00000276929	RP11-266I3.9	
ENST00000549599	ENSG00000257426	RP11-554D14.3	
ENST00000531655	ENSG00000225101	OR52K3P	
ENST00000568867	ENSG00000260109	RP11-883G10.1	
ENST00000522984	ENSG00000253166	RP11-585F1.6	
ENST00000441992	ENSG00000223677	OR2AD1P	
ENST00000403057	ENSG00000220291	RP3-455E7.1	
ENST00000598203	ENSG00000269458	CTD-2579N5.4	
ENST00000440828	ENSG00000234731	AC007163.11	

Supplemental table 4. Statistics of gene annotations in the original genome (MGSAC) sequence.

	Ensembl annotations	marmoset cDNA	human cDNA	ab initio by AUGUSTUS	RNA-seq
# of transcripts	53,427	45,517	116,454	32,161	79,281

Supplemental table 5. The total length of each chromosome in MGSAC genome and the improved (CIEA) genome.

	chr1	chr2	chr3	chr4
MGSAC	210,400,635	204,313,951	190,850,796	171,630,274
CIEA	207,769,690	202,061,623	188,915,795	169,636,824
	chr5	chr6	chr7	chr8
	159,171,411	158,406,734	155,834,243	128,169,293
	156,926,887	156,541,257	153,861,075	126,746,118
	chr9	chr10	chr11	chr12
	124,281,992	132,174,527	130,397,257	121,768,101
	122,668,591	130,530,602	128,799,695	120,127,041
	chr13	chr14	chr15	chr16
	117,903,854	108,792,865	98,464,013	96,796,970
	116,575,745	107,535,158	97,356,171	95,698,484
	chr17	chr18	chr19	chr20
	74,750,902	47,448,759	49,578,535	44,557,958
	73,868,652	46,865,366	49,025,376	43,957,344
	chr21	chr22	chrX	chrY
	50,472,720	49,145,316	142,054,208	2,853,901
	49,901,391	48,126,568	140,450,746	2,824,998

A reason that the length of every chromosome in CIEA genome becomes shorter than the one in MGSAC is that the insert lengths of scaffolds in the MGSCA genome were overestimated and the lengths were modified to the correct lengths by gap filling in the CIEA genome.

Pedigree information about animals in CIEA:

The marmoset colony at CIEA was introduced mainly from Imperial Chemical Industries (ICI, London, England) in 1983. Then the breeding was carried out at CLEA Japan Inc.. “I2075 male” was born in September 22, 2000 from the pair of “I028 female” and “I021 male”, and came to CIEA at February 5, 2008. The BAC library was constructed from a marmoset embryonic stem (ES) cell line (Sasaki et al., Stem Cells, 2005). The ES cell line was established from an embryo obtained from a pair of animals of “I992 female” and “IH554 male”.

According to the pedigree information, there was no lineal consanguinity among “I2075 male”, two animals “I992 female” and “IH554 male” for the embryonic stem cells, and their ancestors within three generations.
